# Gut virome-wide association analysis identifies cross-population viral signatures for inflammatory bowel disease

**DOI:** 10.1186/s40168-024-01832-x

**Published:** 2024-07-18

**Authors:** Xiangge Tian, Shenghui Li, Chao Wang, Yanyan Zhang, Xiaoying Feng, Qiulong Yan, Ruochun Guo, Fan Wu, Chunxue Wu, Yan Wang, Xiaokui Huo, Xiaochi Ma

**Affiliations:** 1 Second Affiliated Hospital, Dalian Medical University, Dalian, 116023 China; 2 Dalian Key Laboratory of Metabolic Target Characterization and Traditional Chinese Medicine Intervention, College of Basic Medical Sciences, Dalian Medical University, Dalian, 116044 China; 3 Puensum Genetech Institute, Wuhan, 430076 China

**Keywords:** Gut virome, Inflammatory bowel disease, Viral-like particle virome, Bulk virome, Viral signatures, IBD-characterized viruses

## Abstract

**Background:**

The gut virome has been implicated in inflammatory bowel disease (IBD), yet a full understanding of the gut virome in IBD patients, especially across diverse geographic populations, is lacking.

**Results:**

In this study, we conducted a comprehensive gut virome-wide association study in a Chinese cohort of 71 IBD patients (15 with Crohn’s disease and 56 with ulcerative colitis) and 77 healthy controls via viral-like particle (VLP) and bulk virome sequencing of their feces. By utilizing an integrated gut virus catalog tailored to the IBD virome, we revealed fundamental alterations in the gut virome in IBD patients. These characterized 139 differentially abundant viral signatures, including elevated phages predicted to infect *Escherichia*, *Klebsiella*, *Enterococcus_B*, *Streptococcus*, and *Veillonella species*, as well as IBD-depleted phages targeting *Prevotella*, *Ruminococcus_E*, *Bifidobacterium*, and *Blautia species*. Remarkably, these viral signatures demonstrated high consistency across diverse populations such as those in Europe and the USA, emphasizing their significance and broad relevance in the disease context. Furthermore, fecal virome transplantation experiments verified that the colonization of these IBD-characterized viruses can modulate experimental colitis in mouse models.

**Conclusions:**

Building upon these insights into the IBD gut virome, we identified potential biomarkers for prognosis and therapy in IBD patients, laying the foundation for further exploration of viromes in related conditions.

Video Abstract

**Supplementary Information:**

The online version contains supplementary material available at 10.1186/s40168-024-01832-x.

## Background

The human gut virome represents a dynamic community of viruses inhabiting the gastrointestinal tract, primarily comprising bacteriophages with limited eukaryotic and archaeal viral presence. Factors influencing gut virome composition and diversity include genetics, diet, antibiotic use, and environmental exposures [1–4]. This complex virome has been implicated in the pathogenesis of several human disorders. Notably, it was first associated with inflammatory bowel disease (IBD) [5], and subsequent research linked the gut virome to conditions such as obesity, diabetes [6], metabolic syndrome [7], colorectal cancer [8], autoimmune diseases [9–11], and liver disease [12, 13]. These interactions occur within the context of the gut microbiome and the host immune system and are crucial for maintaining gut homeostasis and defending against pathogens [14].

The study of the gut virome predominantly employs two approaches: bulk metagenome sequencing and virus-like particle (VLP) enrichment followed by sequencing. While bulk metagenome analysis has long been used to characterize the entire microbial community (i.e., bacteriome, archaeome, and mycobiome) in the gut [15], its use in predicting viral genomes is relatively recent, with viral reference databases still evolving [16]. In contrast, VLP enrichment-based technology has emerged as a promising tool for studying the gut virome in the past few years [17, 18], yielding a greater proportion of viral sequences and simplifying the identification of viruses in complex microbiota. Nevertheless, VLP enrichment has its challenges, including incomplete databases and potential methodological biases, requiring resolution to enhance the understanding of viral characteristics linked to diseases.

Both VLP and bulk virome approaches have been used to investigate the characteristics of the gut virome in patients with IBD, including Crohn’s disease (CD) and ulcerative colitis (UC). Early VLP-based studies revealed a significant expansion of *Caudovirales* bacteriophages in CD and UC patients [5]. A subsequent study expanded on this by identifying IBD-specific virome changes and found increased temperate phages in CD patients [19]. Moreover, bulk virome analysis revealed significant alterations in eukaryotic viruses in IBD [20], consistent with findings in children with IBD [21, 22]. Experimental advances have demonstrated that fecal virus-like particles isolated from UC patients exacerbate colitis severity in mice [23], and viromes from IBD colon tissue promote inflammation in mice [24]. However, further research is needed to obtain detailed information about gut viral structural changes and their impact on IBD. Comprehensive technologies and reference databases are essential for the universal and streamlined identification of these associated changes.

In this study, we designed and carried out a virome-wide association study based on VLP and bulk metagenome sequencing and analysis of fecal samples from an IBD patient and healthy control cohort (total *n* = 148). We established an integrated gut virus catalog tailored to the IBD virome, facilitating the identification of numerous viral signatures. Notably, these signatures displayed remarkable consistency across diverse populations, including those in Europe and the USA. Moreover, we conducted fecal virome transplantation (FVT) experiments using human fecal VLPs in a mouse model, confirming the roles of IBD viral signatures in disease progression. Our findings provide valuable insights into the IBD gut virome, offering a range of reproducible biomarkers with potential implications for prognostic and therapeutic strategies, and establish a framework for exploring virome landscapes in other relevant disorders.

## Results

### Subject characteristics

Our cohort was composed of 71 patients with IBD (including 15 with CD and 56 with UC) and 77 healthy controls. The phenotypic characteristics of all the subjects are summarized in Supplementary Table 1. There were no significant differences in age, sex, or body mass index (BMI) between IBD patients and healthy controls, and these characteristics did not differ between patients with CD and those with UC. The disease activity of IBD patients was determined by the simplified Crohn’s Disease Activity Index (sCDAI) [25], which revealed that 66.7% of CD patients and 82.1% of UC patients experienced moderate or severe activity periods.

### Gut virome sequencing and virus catalog construction

**Fig. 1:**
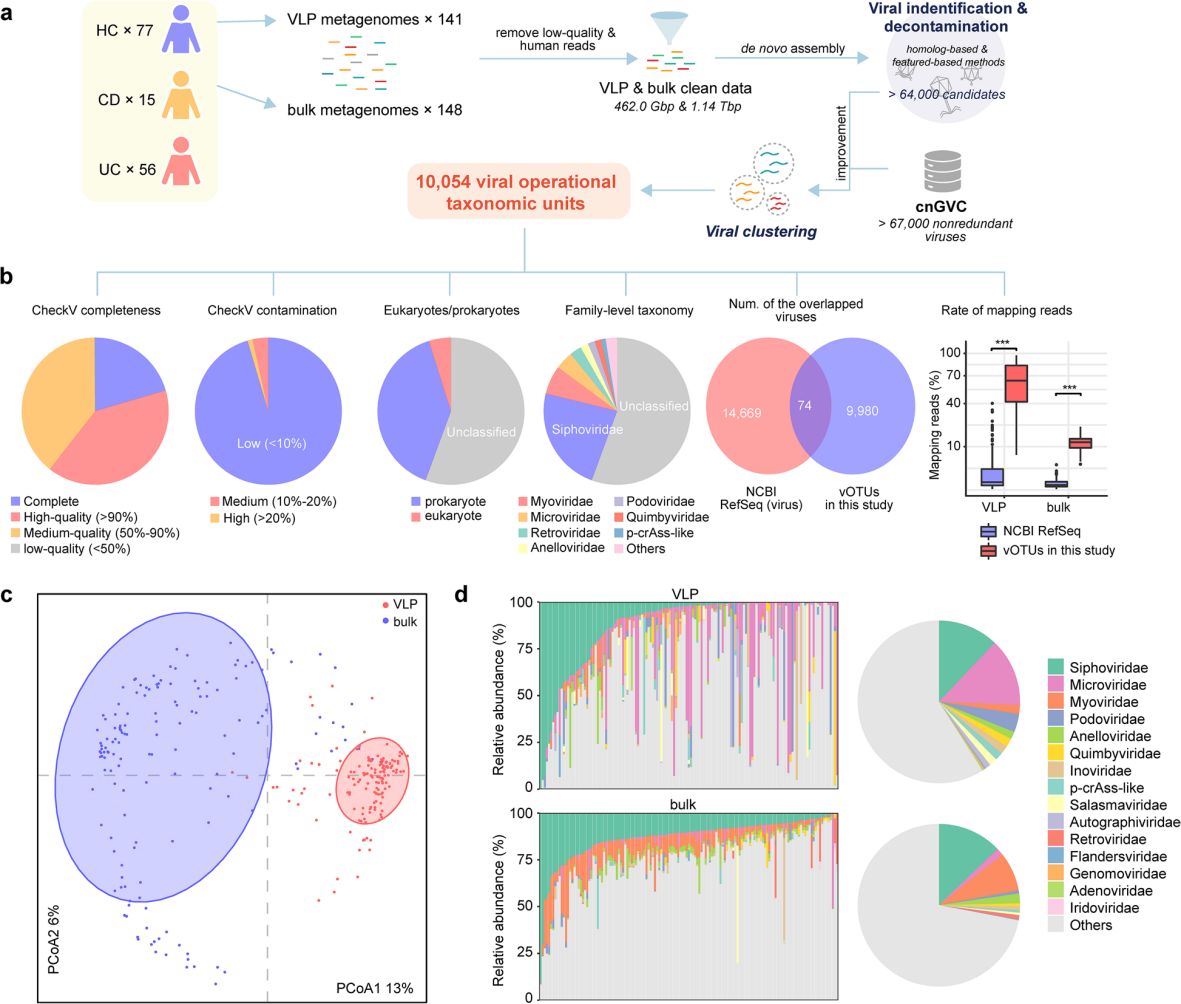
Overview of the study design and the integrated gut virus catalog. **a** Workflow of the construction of the nonredundant gut virus catalog from IBD patients and healthy subjects. **b** Statistics of the gut virus catalog. Pie plots show the completeness, contamination, proportions of eukaryotic and prokaryotic viruses, and family-level taxonomic classification of the virus catalog. Venn diagram shows the overlap between the integrated gut virus catalog and the viruses from the NCBI RefSeq database. Boxplot shows the fraction of mapped metagenomic reads to the integrated gut virus catalog and the viruses from the NCBI RefSeq database. Wilcoxon rank-sum test: ***p < 0.001. **c** Principal coordinate analysis (PCoA) reveals the difference between the VLP virome and bulk virome (141 paired samples were included in the analysis). Samples are shown at the first and second principal coordinates (PCoA1 and PCoA2), and the variance explained (%) by these two PCoAs is displayed. Ellipses represent a 95% confidence interval surrounding each group. **d** Comparison of the gut viral structure between VLPs and bulk viromes. Bar plots show the family-level composition of each sample from the VLP and bulk viromes. Pie plots show the overall proportions

To characterize IBD virome, we processed and analyzed fecal samples from 148 subjects using VLP metagenomic and bulk metagenomic sequencing technologies (detailed in “Methods”). This generated a total of 462.0 Gbp (VLP clean data, averaging 3.3 ± 1.9 Gbp per sample) and 1.14 Tbp (bulk clean data, average 7.7 ± 5.2 Gbp per sample) of high-quality nonhuman data (Fig. 1a). The metagenomic data of each sample were individually de novo assembled, and the viral sequences (representing > 64,000 candidates for all samples) were identified from these assembled contigs using an integrated pipeline detailed in the “Methods” section, consistent with our prior studies [18, 26, 27]. As a considerable number of viral sequences in this study were potentially fully assembled in other Chinese metagenomes, we incorporated ~ 17,000 viral genomes from the comprehensive Chinese gut virus catalog (cnGVC) [26], which exhibited high similarity and coverage with our current viral candidates. Next, we combined all viral sequences and removed duplicates with > 95% nucleotide identity across > 70% of the sequences, resulting in a catalog of 10,054 viral operational taxonomic units (vOTUs) for subsequent analyses (Supplementary Table 2). Utilizing CheckV [28], it was estimated that the majority (60.6%) of vOTUs possessed complete or high-quality (≥ 90% completeness) genomes, with over 90% of vOTUs showing low contamination (< 10%) (Fig. 1b). Taxonomically, 39.5% and 4.9% of vOTUs could be assigned to known prokaryotic or eukaryotic viral families, respectively, while the remaining vOTUs (55.6% of all vOTUs) remained unclassified at the family level. Consistent with previous studies [18, 19, 29], we found that the most dominant classifiable vOTU families were *Siphoviridae*, *Myoviridae*, *Microviridae*, and *Podoviridae*. Significantly, only 74 vOTUs (< 1% of all vOTUs) exhibited species-level homology with viral genomes from the NCBI RefSeq database (Fig. 1b), highlighting the extraordinary novelty of our catalog. Moreover, an average of 59.8% and 11.7% of the metagenomic reads from the VLP and bulk datasets, respectively, were captured by our vOTU catalog, which was more than an order of magnitude greater than that of the RefSeq viruses (3.1% and 0.3%, respectively; Fig. 1b). This underscores the broader and more comprehensive profiling capability of our viral genome catalog in studying IBD and healthy viromes.

Principal coordinate analysis (PCoA) based on the Bray–Curtis distance at the vOTU level clearly separated the VLP and bulk viromes (permutational multivariate analysis of variance [PERMANOVA] *p* < 0.001; Fig. 1c). This pattern was consistent with the family-level viral profile (Supplementary Fig. 1a). Almost all families exhibited significantly different relative abundances between various samples obtained from the two technologies, especially in the case of two predominant families, *Microviridae* (average relative abundance 13.5% vs. 1.4% in VLP and bulk viromes, respectively, Wilcoxon rank-sum test *q* = 2.5 × 10^–11^) and *Myoviridae* (1.8% vs. 7.8%, *q* = 1.6 × 10^–16^) (Fig. 1d). Notably, of the 401 *Microviridae* vOTUs present in the VLP metagenomes, only 61.8% (248/401) had reads captured by the bulk metagenomic samples (Supplementary Fig. 1b). These results concur with prior studies highlighting the substantial differences between VLPs and bulk technologies in virome capture and profiling [26, 30], emphasizing the need to integrate both methods to comprehensively study entire gut viral communities.

### Eukaryotic and prokaryotic virome diversity in IBD patients and healthy subjects

**Fig. 2:**
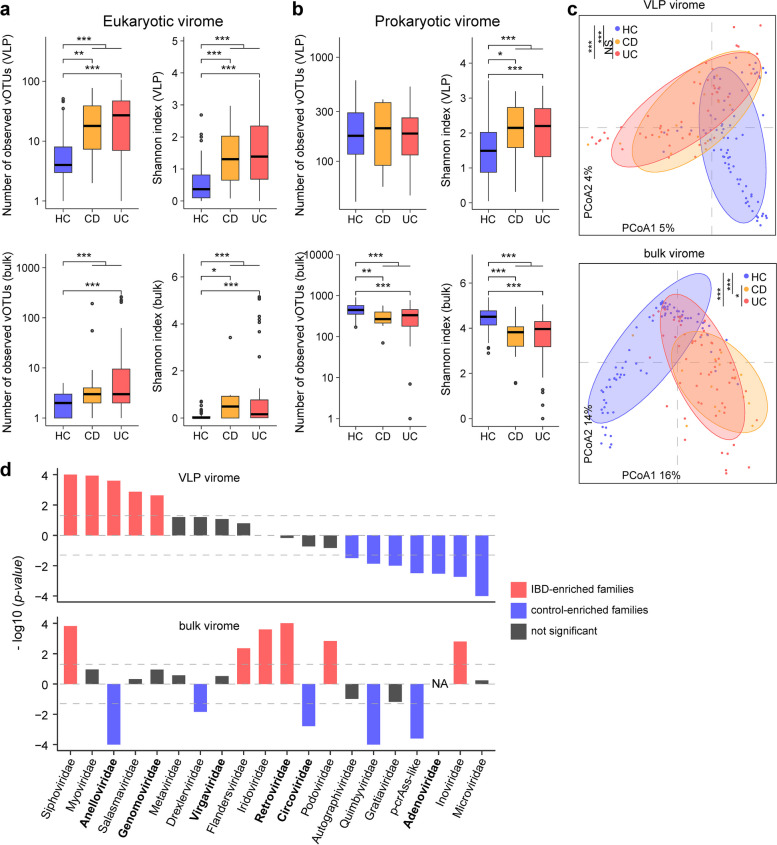
Diversity and structure of the gut virome associated with IBD. **a–b** Boxplot showing the comparison of eukaryotic (**a**) and prokaryotic (**b**) viromes between IBD patients and healthy controls. Boxes represent the interquartile range between the first and third quartiles and the median (internal line). Whiskers denote the lowest and highest values within 1.5 times the range of the first and third quartiles, respectively; dots represent outlier samples beyond the whiskers. Wilcoxon rank-sum test: *p < 0.05; **p < 0.01; ***p < 0.001. HC, healthy controls. **c** Principal coordinate analysis (PCoA) reveals the differences in the VLP (upper panel) and bulk (bottom panel) viromes of IBD patients and healthy controls. The samples are shown at the first and second principal coordinates (PCoA1 and PCoA2), and the ratio of variance contributed by these two PCs is shown. PERMANOVA *adonis* test: ***p < 0.001; ns not significant. The ellipses represent a 95% confidence interval surrounding each group. **d** Family-level comparison of the VLP and bulk viromes of patients and controls. The bar plot shows *p*-values from the Wilcoxon rank-sum test for comparison between two groups. The direction (positive or negative) of the bars indicates how the average relative abundance of each family differs between the patient and control groups. The names of the eukaryotic viruses are bolded

To gauge the gut virome diversity concerning IBD, we conducted a comparative analysis of the observed index (reflecting viral richness) and Shannon index (representing viral diversity) within the viromes of IBD patients and healthy controls. The examination was carried out separately for eukaryotic and prokaryotic viromes due to disparities in the results obtained (as elaborated below). In the eukaryotic virome, we observed a significant increase in both the observed and Shannon indices for IBD patients in comparison to healthy controls. This pattern held true for both the VLP and bulk metagenomic datasets (Fig. 2a). The elevation in eukaryotic viruses, which can infect host cells in the gut, aligns with earlier research suggesting that the heightened presence of eukaryotic viruses may serve as a hallmark in IBD patients, particularly those with UC [20]. In contrast, when considering the prokaryotic virome, we observed that diversity indices (especially the Shannon index) were greater in IBD patients than in healthy controls in the VLP dataset. However, a reversal of this trend was observed in the bulk dataset, where the indices were significantly lower in IBD patients than in controls (Fig. 2b). This discrepancy may stem from the preference of the VLP technique for free viral particles, which tends to increase in IBD patients. In contrast, bulk metagenomes predominantly capture actively infecting viruses or integrated prophages [30], and these are diminished in IBD patients along with some bacteria. As a corroborative point, we found that gut bacteriome diversity significantly decreased in IBD patients in comparison to controls. Importantly, this bacteriome diversity exhibited a stronger positive correlation with the prokaryotic virome diversity in the bulk dataset than in the VLP dataset (Supplementary Fig. 2).

### Gut virome structure associated with IBD

PCoA analysis of the gut virome structure indicated a clear demarcation between IBD patients and healthy subjects, irrespective of whether VLP or bulk viromes were considered (Fig. 2c), underscoring a profound alteration in the viromes of IBD patients. Intriguingly, no significant difference was noted between the gut viromes of CD and UC patients in the PCoA plot. In contrast to IBD-control differentiation, which accounted for 1.9% (PERMANOVA *p* < 0.001) and 7.2% (*p* < 0.001) of the gut virome variance in the VLP and bulk datasets, respectively, the effect of CD-UC differentiation was substantially lower at 0.8% (PERMANOVA *p* = 0.513) and 1.7% (*p* = 0.029). Furthermore, demographic parameters such as age, sex, BMI, and disease activity had minimal impacts on gut virome variance (effect size < 0.5%, PERMANOVA *p* > 0.05 for all parameters).

Given the similarity in gut virome diversity and structure between CD patients and UC patients, we proceeded with a case–control comparison of the gut viral profiles for all IBD patients against those of healthy controls. This analysis focused on the 20 most abundant viral families, representing 99.7% of the total relative abundance of known viral families in both the VLP and bulk viromes. The results revealed that 11 out of the 20 viral families in the VLP metagenomes displayed trends of change consistent with those observed in the bulk metagenomes (Fig. 2d; Supplementary Table 3). Among these viral families, *Siphoviridae*, the predominant viral family in the human gut, displayed a mean relative abundance that was 250% greater (Wilcoxon rank-sum test *q* = 4.0 × 10^–5^) in the VLP dataset and 89% greater (*q* = 6.2 × 10^–4^) in the bulk dataset of IBD patients compared to controls. However, in the IBD gut virome, *Myoviridae* and *Salasmaviridae* were significantly enriched in the VLP dataset but not in the bulk dataset, while *Podoviridae* and *Inoviridae* were enriched in the bulk dataset but not in the VLP dataset. In contrast, *crAss-like* and *Quimbyviridae* viruses experienced a significant reduction in relative abundance in both datasets of IBD patients. Reduction in *Microviridae*, *Adenoviridae*, *Gratiaviridae*, and *Autographiviridae* was exclusively observed in the VLP dataset and not in the bulk dataset. *Anelloviridae* exhibited conspicuous enrichment among IBD patients in the VLP dataset but a marked reduction in the bulk dataset, whereas *Inoviridae* exhibited the opposite trend. (Fig. 2d). At the vOTU level, almost all *Anelloviridae* vOTUs demonstrated increased relative abundance in IBD patients, regardless of whether they were detected in the VLP or bulk datasets (Supplementary Fig. 3). Furthermore, consistent with prior studies [5, 21], it is noteworthy that the relative abundance of *Caudovirales* (primarily composed of *Siphoviridae* and *Myoviridae*) significantly increased in IBD patients compared to controls in both datasets, while that of *Petitvirales* (mainly composed of *Microviridae*) significantly decreased (Supplementary Fig. 4).

### Identification of IBD-associated viral signatures

**Fig. 3:**
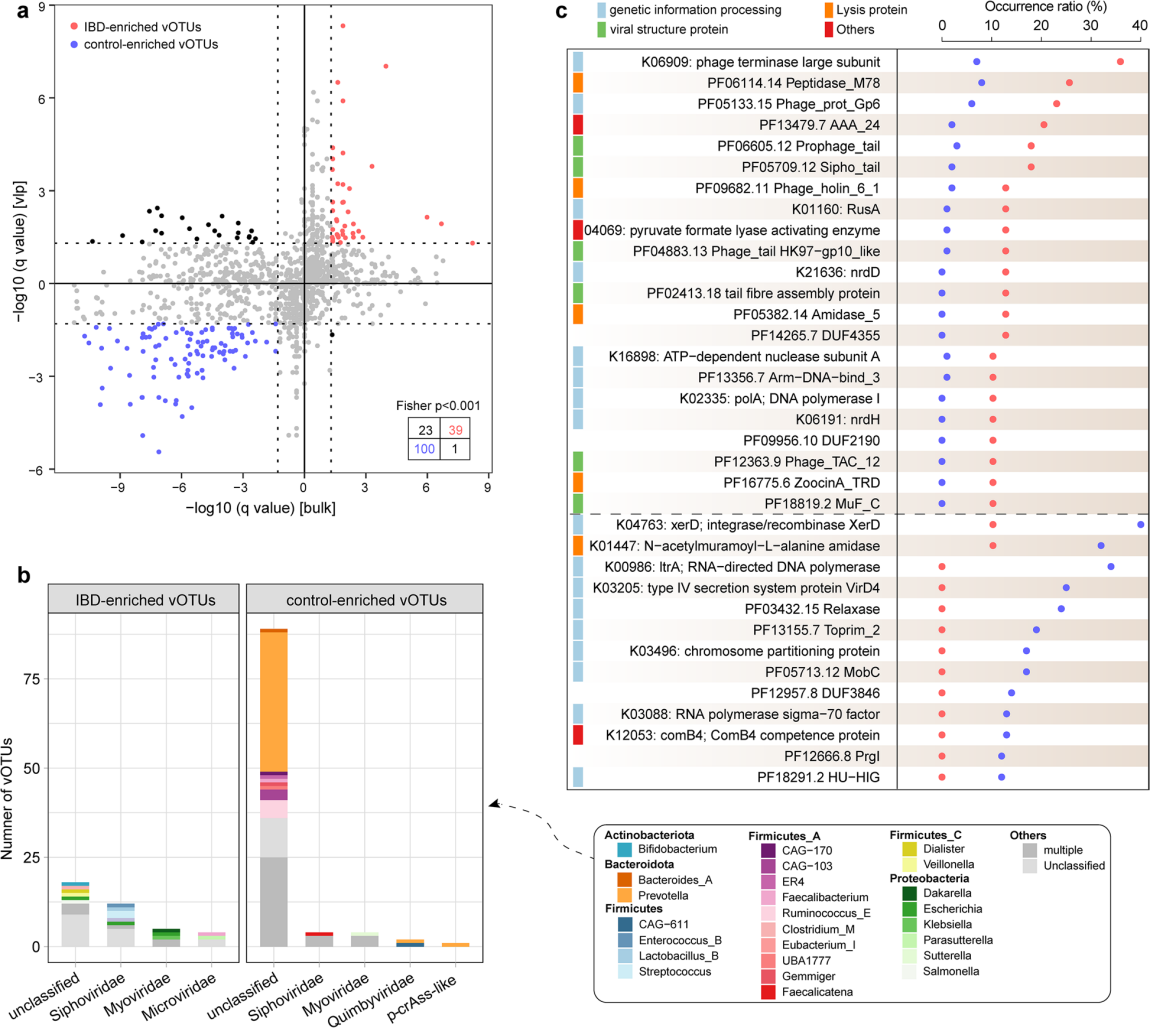
Identification of IBD-associated vOTUs and exploration of their prokaryotic hosts and potential functions. **a** Scatter plot of *q*-values of vOTUs in the VLP and bulk datasets. Red and blue nodes represent the vOTUs that are significantly enriched in the viromes of IBD patients and healthy controls, respectively, with the same tendency in the VLP and bulk datasets. Statistical significance is determined using the Wilcoxon rank-sum test with Benjamini–Hochberg adjustment. **b** Distribution of the taxonomic annotation and host assignment of the IBD-enriched and control-enriched vOTUs. The vOTUs are grouped at the family level, and the prokaryotic host taxa are also shown at the family level. **c** Occurrence rates of 40 differential functions in IBD-enriched and control-enriched vOTUs. Red and blue nodes represent the IBD-enriched and control-enriched vOTUs, respectively. Statistical significance is determined using Fisher’s exact test with Benjamini–Hochberg adjustment, and a *q* < 0.05 was considered statistically significant

To determine the viral species associated with IBD, we conducted a case–control comparison of the vOTU composition in both the VLP and bulk viromes using the Wilcoxon rank‐sum test with Benjamini–Hochberg adjustment. This analysis identified a total of 139 vOTUs that displayed significant differences in relative abundances and consistent trends in case–control comparisons in both datasets (Fig. 3a; Supplementary Table 4); these vOTUs were designated IBD-associated gut viral signatures. Notably, a Fisher’s exact test analysis indicated a high level of agreement in the identification of differential vOTUs between the two datasets (odds ratio = 163; *p* < 0.001), signifying that these IBD-associated markers were not randomly identified. Among these 139 IBD-associated markers, 39 were IBD-enriched vOTUs, representing members of the *Siphoviridae*, *Myoviridae*, and *Microviridae* families, as well as unclassified viruses (Fig. 3b). In contrast, the majority of control-enriched vOTUs (89 out of 100) were from unknown families, with a few originating from the *Siphoviridae*, *Myoviridae*, *Quimbyviridae*, and *crAss-like* families.

To investigate the prokaryotic hosts infected by these viral markers associated with IBD, we performed virus-host prediction based on the comprehensive Unified Human Gastrointestinal Genome (UHGG) collection containing 4644 gut prokaryotes [31] (see “Methods” for details). Approximately, 80% of the viral markers (112/139) were assigned to at least one prokaryotic host, with these hosts primarily belonging to the Bacteroidota, Firmicutes_A, Firmicutes, and Proteobacteria phyla (Supplementary Fig. 5). A notable difference in host preference was observed between IBD- and control-enriched vOTUs (Fig. 3b). For instance, IBD-enriched vOTUs frequently include genera containing common bacterial pathogens, such as *Escherichia*, *Klebsiella*, and *Streptococcus*. Moreover, the genus *Veillonella*, which was identified as an IBD-enriched bacteria in previous studies [32], was also infected by IBD-enriched vOTUs. In contrast, there was a high percentage of control-enriched vOTUs with predicted hosts (54%) infected with species from Bacteroidota, particularly *Prevotella* species, with most of these vOTUs lacking annotations from known viral families. A few control-enriched vOTUs were predicted to infect species from *Ruminococcus_E* and *Faecalibacterium*.

Furthermore, we conducted a functional comparison between IBD- and control-enriched vOTUs. Gene prediction of these vOTUs resulted in 8466 protein-coding genes, 49.2% of which were annotated as functional orthologs within the Kyoto Encyclopedia of Genes and Genomes (KEGG) and Pfam databases [33, 34], spanning 1747 functional families. PCoA and PERMANOVA revealed a significant difference in the functional profiles between IBD-enriched and control-enriched vOTUs (PERMANOVA* p* < 0.001; Supplementary Fig. 6). Using Fisher’s exact test with Benjamini–Hochberg adjustment, we identified 35 functional families that significantly differed in the occurrence rate between IBD-enriched and control-enriched vOTUs (*q* < 0.05; Fig. 3c). Functions related to viral structure proteins, particularly those involved in phage tail assembly (*n* = 6), were notably prevalent in IBD-enriched vOTUs but largely absent in control-enriched vOTUs. This suggests that the control-enriched vOTUs were more likely to belong to non-tailed phages. Moreover, four lysis proteins (PF06114.14, PF09682.11, PF05382.14, and PF16775.6), which interfere with the growth of host bacteria, were also found to be more commonly present in IBD-enriched vOTUs. Conversely, control-enriched vOTUs more frequently encoded genes associated with genetic information processing, indicating a greater focus on genetic replication and expression. Notably, more than 1/3 of the control-enriched vOTUs possessed the reverse transcriptase gene (K00986). These viruses were not annotated as *Retroviridae*, which predominantly infect human and animal vertebrates but were more likely to infect members of Bacteroidota. Numerous studies have demonstrated the presence of reverse transcriptase homologous genes in bacterial genomes, known as retrons, which function as components of the bacterial defense system against phages [35, 36]. Interestingly, we observed that, in addition to carrying the reverse transcriptase gene, several control-enriched vOTUs also contained typical viral protein sequences such as capsid and tape measure proteins (Supplementary Fig. 7). This suggests that these reverse transcriptase genes may not originate from host bacterial genomes but rather from the phages infecting them.

### The IBD viral signatures are consistent across global cohorts

**Fig. 4:**
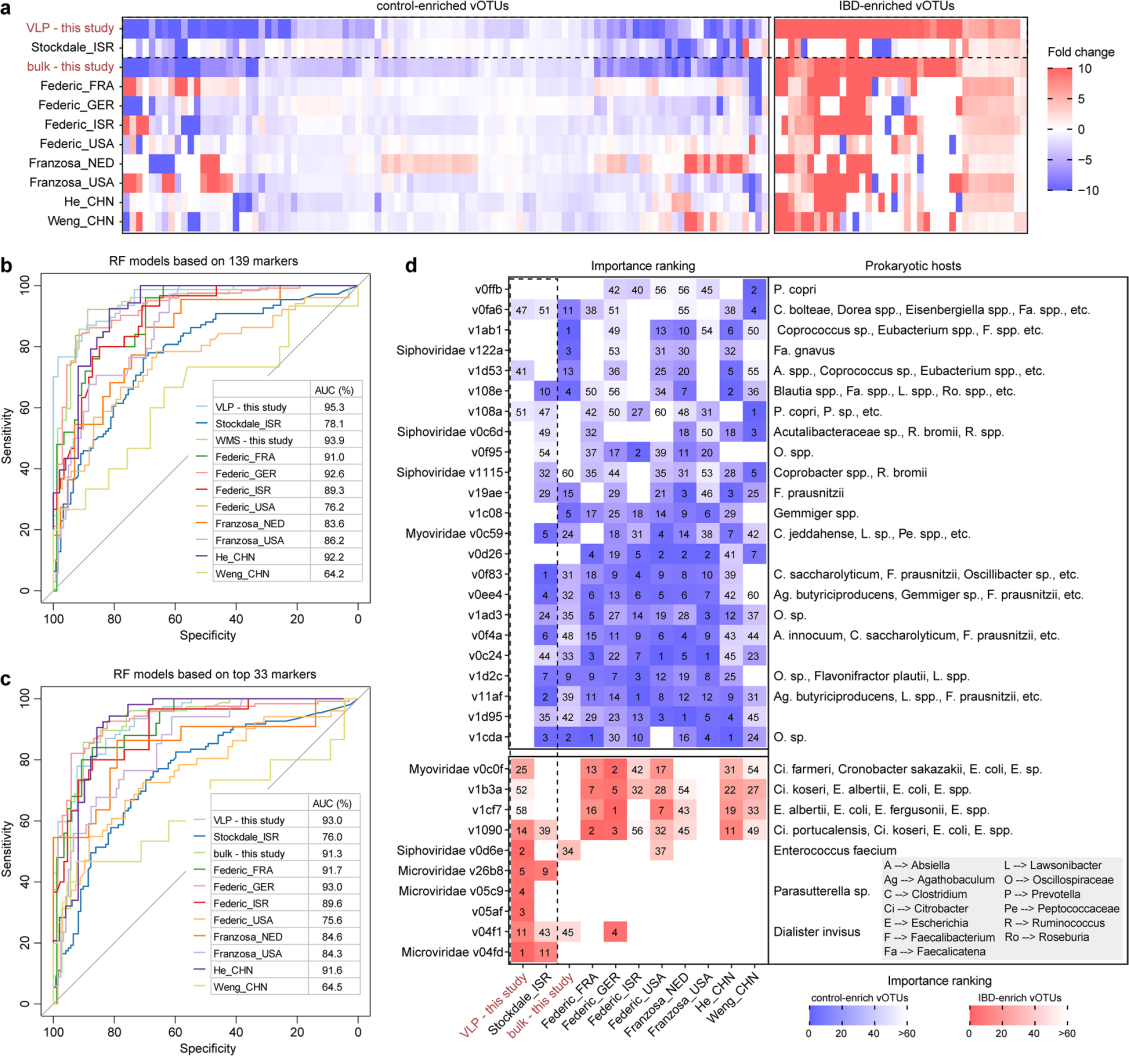
Alterations in IBD-associated vOTUs in the external validation cohort. **a** Heatmap showing the fold changes in IBD-associated vOTUs in the VLP and bulk datasets of this study and external validation cohorts. Fold change > 0, enriched in patients; fold change < 0, enriched in controls. **b–c** Receiver operating characteristic (ROC) analysis of the classification of IBD/control status in each cohort using the random forest model trained by 139 IBD-associated vOTUs (**b**) and 34 top-ranking vOTUs (**c**). The classification performance of the model was assessed by the area under the ROC curve (AUC). **d** Heatmap showing the 34 most important vOTUs in the random forest models and their host information

To assess the reliability and consistency of the 139 IBD-associated vOTUs identified in this study, we validated them in publicly available IBD cohorts from diverse geographic origins. We collected 1171 gut VLP and bulk metagenomic sequencing samples from 9 case–control datasets, including data from the USA, Europe, and China (Supplementary Table 5). We processed these samples by filtering out low-quality and human reads, mapping them to our integrated gut virus catalog containing 10,054 vOTUs, and calculating the relative abundance of each vOTU. PCoA revealed a substantial difference in viral composition between IBD patients and healthy controls among all samples from the 11 datasets (including the 9 public datasets and the VLP and bulk datasets from this study) (PERMANOVA *p* < 0.001; Supplementary Fig. 8), indicating the presence of common signatures in different populations. We then examined the direction of enrichment in the relative abundance of the 139 IBD-associated vOTUs between IBD patients and healthy controls in each cohort. Impressively, the direction of enrichment of these vOTUs displayed remarkable consistency across cohorts (average consistency rate of 70.0 ± 10.4% among the nine public datasets), despite a few exceptions in individual cohorts (Fig. 4a). This finding underscores the robustness of the IBD-associated vOTUs identified in this study in other public databases.

We built random forest (RF) models using the 139 IBD-associated vOTUs to evaluate their potential for classifying IBD patients from healthy controls in each dataset, including both the VLP and bulk metagenomic datasets from this study and the 9 public datasets. The models achieved an average intra-dataset area under the curve (AUC) of 0.857 (ranging from 0.642 to 0.936) in these datasets, with five datasets exhibiting an AUC of over 0.90 (Fig. 4b), supporting the generalizability and reliability of our markers across different datasets. Additionally, we developed RF models based on a subset of 33 IBD-associated vOTUs that integrated the top 5 most important features in the model for each dataset. The average intra-dataset AUC of these models was found to be high at 0.850 (ranging from 0.645 to 0.930), which was comparable to the performance of the RF models based on all markers (Fig. 4c). The 33 IBD-associated vOTUs consisted of 23 control-enriched vOTUs and 10 IBD-enriched vOTUs (Fig. 4d). Notably, some of the control-enriched vOTUs were predicted to infect butyrate-producing bacteria, such as *Agathobaculum butyriciproducens,* and *Faecalibacterium prausnitzii*. However, although three control-enriched vOTUs, v0c24 v0d26, and v1d95, were crucial to the prediction accuracy of most classification models, their taxonomic annotation and host preference remain completely unknown. On the other hand, the ten IBD-enriched vOTUs were found to frequently infect opportunistic pathogens, including *Citrobacter farmeri*, *Escherichia coli*, and *Escherichia fergusonii* (Fig. 4d).

We also performed cross-dataset prediction based on these 33 IBD-associated vOTUs (Supplementary Fig. 9). The cross-dataset RF models achieved an average AUC of 0.758 (ranging from 0.496 to 0.922). Considering the variation in sample sizes across datasets, we performed a leave-one-dataset-out (LODO) analysis, where each dataset was treated as a test dataset in turn. The LODO AUCs ranged from 0.599 to 0.919, with an average AUC of 0.799 (Supplementary Fig. 9). Collectively, these results demonstrate the robustness of the IBD-associated viral signatures identified in this study across diverse datasets, highlighting their potential as reliable targets for future microbiota intervention studies in IBD patients.

### Colonization of IBD-associated viruses regulates experimental colitis in mice

**Fig. 5:**
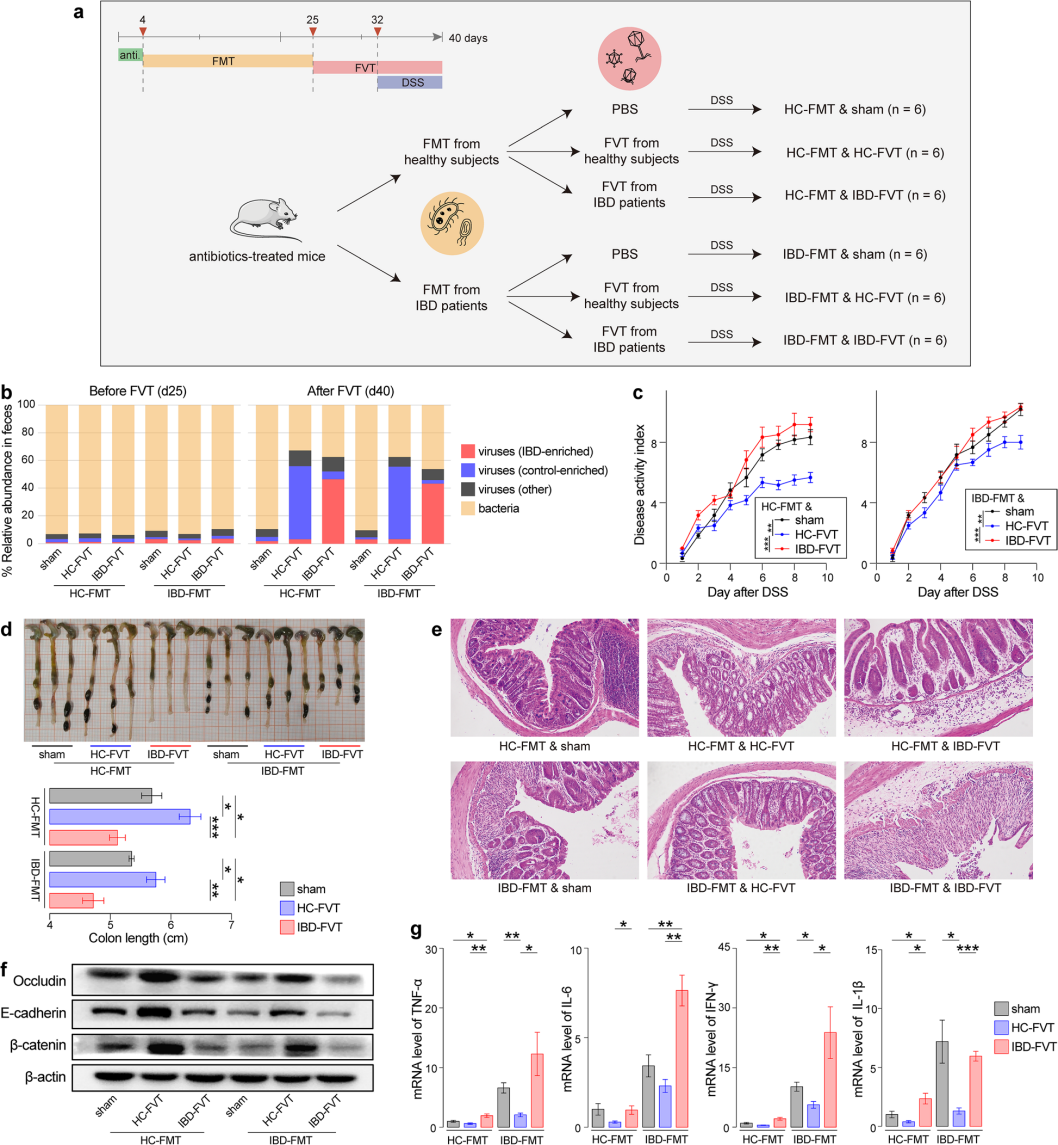
Biological effect evaluation of the gut virome on DSS-induced colitis in mice. **a** Schematic diagram of the construction of the DSS-induced mouse model of colitis. **b** Bar plot showing the microbial composition of the feces of mice before and after FVT. **c** Evaluation of the disease activity index in colitis mice that received gut viromes from IBD patients and healthy controls. **d** Colon length of mice with colitis. **e** Representative images of hematoxylin and eosin (HE) staining of the mouse colon. **f** The expression levels of adhesion and tight junction-related proteins in the mice with colitis. **g** Levels of inflammatory cytokines in the colon of colitis mice. *N* = 6. Statistical tests were performed using Student’s *t*-test: *p < 0.05; **p < 0.01; ***p < 0.001

Although IBD-associated gut viral signatures have been identified, experimental evidence regarding the influence of these viruses on IBD is limited. To address this, we conducted fecal microbiome transplantation (FMT) from either IBD patients or healthy subjects into broad spectrum antibiotic-treated mice to generate human microbiota-associated (HMA) mice, followed by high-dose FVT from patients and controls into these HMA mice and the administration of 2% DSS in their drinking water to induce experimental colitis (Fig. 5a). The donors for these experiments, including eight IBD patients (three CD patients and five UC patients) and seven healthy individuals, were randomly selected from our original cohort, and their fresh feces and fecal virus-like particles were pooled for the FMT and FVT procedures, respectively (see “Methods” for details). We then assessed the viral and bacterial composition of mouse feces before and after the FVT procedures using shotgun metagenomic sequencing. Low levels of viruses were detectable in the HMA mice before FVT, possibly reflecting the baseline proportion of viruses in their feces (Fig. 5b). Following FVT and DSS-induced colitis, we observed that the mice receiving the fecal virome from healthy donors (referred to as “HC-FVT mice”) showed a significant expansion of control-enriched vOTUs compared to their pre-FVT state, with average relative abundances increasing from 2.0% to 52.4% (Student’s *t*-test *p* = 2.5 × 10^–8^). Similarly, the mice that received the virome from IBD patients (referred to as “IBD-FVT mice”) exhibited a substantial expansion of IBD-enriched vOTUs, with average relative abundances increasing from 2.3% to 44.9% (*p* = 9.1 × 10^–8^). We also estimated the virus-to-bacterium ratio of mouse feces and found that this ratio reached 51–1087 (average 366 in HC-FVT mice and 272 in IBD-FVT mice) in post-FVT samples (Supplementary Fig. 10), suggesting that the overgrowth of these viruses may have overwhelmed the existing bacterial content in the mouse gut microbiota. These findings demonstrate that the recipient mice efficiently recapitulated the viral features of the patient or control donors virome.

Next, we compared colitis-related symptoms in mice from the HC-FVT, IBD-FVT, and sham FVT groups. After DSS induction, the mice exhibited a colitis phenotype characterized by body weight loss, diarrhea, rectal bleeding, colon shortening, pathological changes, increased levels of inflammatory factors, and decreased expression of tight junction proteins, indicating that DSS induced colonic inflammation and impaired gut barrier function. Regardless of whether the initial FMT was from IBD patients or healthy donors, we found that compared with the sham group, the HC-FVT group exhibited reduced colitis-related symptoms, including a significant reduction in the disease activity index (DAI), an elongated colon length, and less severe pathological colon injury (Fig. 5c-e). In contrast, the IBD-FVT group displayed exacerbated colitis, with greater DAI scores, more severe pathological injuries, and shorter colons than both the HC-FVT and sham groups (Fig. 5c-e and Supplementary Fig. 11). Moreover, the expression levels of adhesion and tight junction-related proteins, such as E-cadherin, β-catenin, and occludin, were upregulated in the HF-FVT group and downregulated in the IBD-FVT group (Fig. 5f and Supplementary Fig. 12), indicating improved gut permeability in the HF-FVT group and worsened gut permeability in the IBD-FVT group. Additionally, the IBD-FVT group, irrespective of whether the HMA mice received microbiota from IBD patients or healthy donors, exhibited significantly greater levels of inflammatory cytokines, including TNF-α, IL-6, IFN-γ, and IL-1β, than the HC-FVT or sham groups (Fig. 5g). In contrast, the HC-FVT group exhibited reduced levels of certain cytokines such as IL-6, IFN-γ, and IL-1β in IBD HMA mice compared with those in the sham group (Fig. 5g). Taken together, these findings indicate that the colonization of viruses from healthy donors (HC-FVT) has a mitigating effect on colitis in a mouse model, whereas that of viruses from IBD patients (IBD-FVT) exacerbates colitis with a substantial expansion of IBD-enriched vOTUs in the mouse gut microbiota.

## Discussion

### Improved approaches for investigating the IBD gut virome

IBD is a severe gastrointestinal disorder that is increasingly attributed to dysbiosis of the gut microbiota, leading to immune dysfunction and the onset of the disease [37, 38]. While extensive research has identified the significant roles of gut bacteria and even fungi in IBD pathology, there has been a notable lack of information regarding the involvement of viruses. The limitations in studying the gut virome within the context of specific diseases can be attributed to two primary technical challenges. First, detection methods for viral communities have often been restricted to either VLP or bulk metagenomics, lacking comprehensive cross-validation. Second, despite the construction of several large viral genome reference databases, there remains a substantial reservoir of unexplored viral sequences within the human gut [16, 26]. This reliance on reference databases for gut virome analysis significantly restricts the scope of viral investigations. In this study, we sought to overcome these challenges by employing both VLP and bulk metagenomics sequencing methods in a cohort of individuals to conduct an extensive investigation into the “whole gut virome” associated with IBD. Notably, these two sequencing approaches demonstrated distinctions in their capacity to capture viruses. In alignment with prior assumptions [19, 30], VLP sequencing exhibited a preference for capturing free viral particles, particularly those from the *Microviridae* family, whereas bulk sequencing was more adept at reconstructing viral sequences that had partly integrated into bacterial hosts. Furthermore, we constructed a reference gut viral catalog that represented more than 10,000 nonredundant vOTUs based on our high-depth sequenced samples. The majority (61.2%) of vOTUs in this study were not present in existing gut virus catalogs (i.e., the Gut Virome Database (GVD) [30], Gut Phage Database (GPD) [39], or Metagenomic Gut Virus (MGV) [40]) (Supplementary Fig. 13), and almost all vOTUs had no homology with the viruses in the NCBI RefSeq database. These findings suggest that our catalog substantially supplements the deficiencies of previous viral references, allowing for a more comprehensive examination of the IBD gut virome. Overall, our results underscore the significance of utilizing complementary sequencing techniques and viral reference databases to fully explore the diversity of viruses within the human gut. This study presents a paradigm for future virome-wide studies of other relevant disorders.

### Characteristics of the IBD gut virome and viral signatures

The results from both the VLP and bulk datasets consistently indicated an increase in eukaryotic virome richness and evenness in IBD patients compared to healthy controls. In particular, two eukaryotic viral families, *Retroviridae* and *Genomoviridae*, were found to be enriched in IBD patients. Although the overgrowth of the *Retroviridae* family in the gut has been implicated in several diseases including CD [26, 41], its specific influence on IBD remains unclear. Our analysis showed that VLP data supported the enrichment of *Anelloviridae* in IBD patients, whereas bulk data yielded the opposite result. Considering that at the vOTU level, the majority of *Anelloviridae* vOTUs in both VLP and bulk samples were enriched in patients, we believe that the enrichment of *Anelloviridae* in patients appears to be more credible. The enrichment of *Anelloviridae* was also previously observed in a study of onset CD [22], possibly related to long-term immune therapy [42, 43]. Notably, another study focusing on the gut mucosa virome found significantly greater levels of eukaryotic *Hepadnaviridae* transcripts in patients with UC than in both healthy controls and CD patients [20]. However, this phenomenon was not observed in our datasets.

In terms of the prokaryotic virome, the alpha diversity results for the VLP and bulk datasets revealed diametrically opposite findings. In the bulk dataset, we observed a significant decrease in prokaryotic virome diversity in IBD patients compared with the controls, and it exhibited a significant positive correlation with bacteriome diversity. Previous studies have extensively reported a substantial decrease in bacterial diversity in IBD patients [44–46]. These findings demonstrated a strong dependency of the bacterial community on the prokaryotic viral community in the bulk dataset. In contrast, the VLP dataset showed a significant increase in prokaryotic virome diversity in the IBD group, which was entirely unrelated to the bacteriome. Combining these findings with previous research suggesting that VLPs may preferentially capture free-floating viruses outside host cells, while bulk analysis captures a greater proportion of intracellular viral communities. These observations suggest a more frequent occurrence of prokaryotic cell lysis within the gut of IBD patients.

At the family level, we found that *Siphoviridae* and *Myoviridae* were significantly enriched in IBD patients, while *crAss-like* and *Quimbyviridae* were decreased. *Siphoviridae* and *Myoviridae* constitute the major viral communities in the human gut [1]; most members of these two families are temperate viruses, but their functions in the IBD virome remain unknown. Additionally, both *Siphoviridae* and *Myoviridae* belong to the order Caudovirales, and their enrichment was consistent with previous studies of the IBD virome [5, 47]. At the vOTU level, we also identified numerous IBD-enriched *Siphoviridae* and *Myoviridae* vOTUs. These vOTUs contained a large proportion of phages infecting bacteria such as *Escherichia*, *Klebsiella*, *Enterococcus_B*, *Streptococcus*, and *Veillonella*. *Escherichia*, *Klebsiella*, and *Enterococcus* spp. are typical opportunistic pathogens capable of inducing inflammation and triggering various diseases [48–50], and their pathogenicity in IBD has been recently reported [51, 52]. Overproliferation of *Escherichia* phages and other Enterobacteriaceae phages has also been observed in mucosal viromes of UC patients [53]. *Streptococcus* and *Veillonella* are commensal bacteria in the gut that have proinflammatory properties and are associated with diseases such as cirrhosis and obesity [54]. Although *Streptococcus* phages have been reported to be associated with metabolic and autoimmune diseases [55], their role in IBD has not been reported. In addition, our results showed that IBD-enriched vOTUs were more frequently associated with IBD-related pathogenic bacteria as their hosts, suggesting their involvement in the development of IBD through their prokaryotic hosts, as observed in immune diseases [10, 56]. Regarding IBD-depleted viruses, *crAss-like* and *Quimbyviridae* are newly identified viral clades that tend to infect Bacteroidetes, which are major polysaccharide degraders in the gut [57]. Similarly, at the vOTU level, numerous unclassified control-enriched vOTUs are predicted to infect Bacteroidetes, particularly *Prevotella* species, which are known to widely participate in plant polysaccharide utilization [58]. Studies have shown that these two clades of viruses, *crAss-like* and *Quimbyviridae*, have larger genomes and a substantial number of functional auxiliary genes, especially carbohydrate-active enzymes involved in polysaccharide metabolism [59, 60]. Therefore, the decrease in *crAss-like* and *Quimbyviridae* viruses and *Prevotella* phages may imply the loss of certain core viral functions, such as polysaccharide metabolism, in the gut virome of IBD patients.

### Universality and disease relevance of the IBD-associated viral signatures

Recent meta-analyses of the gut virome in colorectal cancer patients have revealed distinctive viral signatures among diverse populations and suggested that their virome characteristics may operate independently from the bacteriome [8]. Similarly, by studying fecal samples from populations spanning Europe, the USA, Israel, and China, we found that despite population heterogeneity and differences in sample treatment methods, most of the IBD-associated viral signatures identified in our current study could be validated in other datasets. This indicates that our dataset and analyses effectively captured the universal signal characteristics in the IBD virome. The consistency of gut viral signatures in transcontinental datasets may not only be correlated with common changes in the bacterial microbiome but also suggest that some viruses can independently influence IBD in a consistent manner across geographic populations, potentially through mechanisms such as immunoregulation or viral infection [61]. Furthermore, we discovered that the classifying models with only 33 viral markers could effectively distinguish between the IBD and control groups in nearly all datasets (average AUC > 0.80), suggesting their potential diagnostic value for IBD and relevant diseases.

Previous animal experiments have confirmed that FVT from UC patients can exacerbate the severity of DSS-induced colitis [23]. Here, we performed a similar FVT experiment by transplanting VLPs from IBD patients and healthy controls into an HMA mouse model of experimental colitis. Surprisingly, we observed that IBD-associated viruses (both IBD-enriched and control-enriched viruses) could colonize the mice at very high levels after FVT, with an average relative abundance exceeding 50% and more than 200 times the number compared to bacteria. Accompanying the colonization of IBD-associated viruses, mice exhibited significant changes in colitis, pathological injury, and intestinal permeability. Isolation and direct functional investigation of these IBD-associated viruses may be needed to address causality between the gut virome and IBD progression in future research. Importantly, in contrast to previous studies focusing on the exacerbation of disease by IBD-FVT, our research found that HC-FVT effectively improved intestinal permeability and inflammatory markers in mice with colitis. This finding suggests the potential of HC-FVT as a therapeutic approach for the IBD population.

Furthermore, we also found that compared with mice with IBD-FMT, mice with HC-FMT exhibited an alleviated IBD phenotype. The improvement derived from the HC-FMT was comparable to that of the HC-FVT. It would be interesting to differentiate the contributions of FMT and FVT to IBD treatment. However, this was impossible in the present study because the mice received FMT and FVT simultaneously. The use of an antibiotic cocktail before the establishment of an HMA mouse model is another point that requires attention. Broad-spectrum antibiotic regimens can induce a 10,000-fold reduction in the gut bacterial load, leading to a significant increase in the diversity and relative abundance of the gut fungal microbiota, and a decrease in gut virome diversity [3, 62, 63]. Additional studies using germ-free mice or clinical trials may be needed to further clarify the role of FVT in IBD progression in the future.

In discussing the limitations of our study, it is important to acknowledge that while we made efforts to ensure age, sex, and BMI matching between the control and disease groups during sample collection, and established strict exclusion criteria such as excluding individuals with liver diseases, kidney diseases, cardiometabolic diseases, and cancers, there are potential confounding factors related to participants’ income levels, dietary habits, exercise patterns, and lifestyles. These differences among subjects may have influenced the study outcomes. Therefore, we advocate that future research exercise greater caution in selecting control groups, striving to align demographic, socioeconomic, and lifestyle factors across different cohorts to enhance the reliability and applicability of the research findings. Additionally, the presence of potential impurities within VLPs is a limitation of this study. These impurities, including large glycoprotein molecules, lipoprotein molecules, outer membrane vesicles, bacterial fragments, fungal fragments, and even trace amounts of secondary metabolites and free nucleic acid, could influence our research outcomes. Subsequent research efforts aimed at further separating phages or eukaryotic viruses exerting effects will overcome this issue.

## Conclusions

Through integrated VLP and bulk virome studies, we demonstrated a significant disruption in the gut virome of IBD patients and identified 139 IBD-associated vOTUs. Furthermore, we substantiated that these IBD viral signatures can be validated across diverse human population datasets and possess potential modulatory features in regulating disease severity in animal models. This study provides a detailed analysis of the gut virome landscape for IBD, and the results and resources provided may promote future mechanistic and therapeutic work.

## Methods

### Subjects and sample collection

Ethical approval for this study was obtained from the Ethics Committee of Xinhua Hospital affiliated with Dalian University (approval no. XH2020A008), and written informed consent was obtained from all participants. All subjects involved in this study were recruited from Dalian, Liaoning province, China, and were rigorously screened to exclude individuals with liver diseases, kidney diseases, cardiometabolic diseases (e.g., diabetes, moderate to severe hypertension), and cancers. Additionally, none of the participants had taken antibiotics within 1 month or antiviral drugs within 3 months prior to sample collection. The IBD patients were enrolled with inclusion criteria necessitating a clear diagnosis by a licensed physician in strict accordance with the ECCO-ESGAR Guideline for Diagnostic Assessment in Inflammatory Bowel Disease [64]. The Simplified Crohn’s Disease Activity Index (sCDAI) integrates clinical symptoms, laboratory parameters, medication utilization, and the presence of perianal disease to offer a comprehensive assessment of disease severity. Healthy participants were matched with IBD patients in terms of age, sex, and BMI to ensure consistency in the results. More than 20 g of fresh fecal specimen per individual were collected from both IBD patients and healthy controls, using sterile fecal collection containers. Fecal specimens were collected and transported to the laboratory within 2 h. One portion of the samples was directly frozen at − 80 °C for subsequent NGS sequencing, while another portion was subjected to processing for VLPs enrichment. For the latter, a parallel set of samples was supplemented with glycerol as a protective agent before being frozen at − 80 °C. All samples for VLPs enrichment were stored frozen for less than 1 month before processing.

### VLP enrichment and metagenomic sequencing

The procedures of VLP enrichment, viral DNA extraction, and metagenomic sequencing were conducted following our previously established protocols with minor adjustments [18, 26]. Briefly, 0.17 g of fecal material for each specimen was mixed with 1 mL of Hank’s balanced salt solution (HBSS, devoid of phenol red) and vigorously homogenized using a vortex mixer (with pulses lasting a minimum of 15 s). Subsequently, the samples underwent centrifugation at 10,000 × *g* for 10 min at 4 ℃. The resulting supernatant was then successively filtered through 0.45-μm and 0.2-μm filters. Afterward, the samples were subjected to ultracentrifugation at 750,000 × *g* for 60 min at 8 ℃, and the resulting pellet was resuspended in 500 μL of HBSS; 120 μL of this resuspension was transferred and treated with a mixture of nucleases (comprising 2.4 μL of TURBO DNase (4.8 U, Invitrogen), 8 μL of RNase A/T1 Mix (16 μg RNase A, 40 U RNase T1, Thermo Scientific), and 1 μL of Benzonase (5 U, EMD Millipore)) for 120 min at 37 ℃. Following this, nucleic acids were immediately extracted using the TIANamp Viral Genome DNA/RNA extraction kit (TIANGEN, China), following the manufacturer’s instructions. Subsequently, a DNA sequencing library was constructed utilizing the NEB Next Ultra DNA Library Prep Kit (NEB, USA) in accordance with the manufacturer’s guidelines, with unique index codes assigned to each sample. The library’s quality was confirmed using an Agilent 2100 instrument. Index-coded samples were then clustered using the Illumina PE Cluster Kit (Illumina, USA) on a cBot Cluster Generation System, following the manufacturer’s protocols. Following cluster generation, the DNA libraries were subjected to metagenome shotgun sequencing on the Illumina NovaSeq platform, resulting in the generation of 150 bp paired-end reads.

### Bulk metagenomic sequencing

For bulk metagenomic analyses, each fecal specimen from IBD patients or healthy controls was subjected to microbial DNA extraction using the TIANamp Stool DNA Kit (TIANGEN, China). DNA quality was assessed using the Qubit 2.0 system, and the extracted DNA samples were stored at − 80 ℃ until needed. After that, the DNA library and bulk metagenomic sequencing were consistent with the operations of the VLP samples.

### Quality control and assembly of metagenomic datasets

Raw reads from the VLP and bulk metagenomic datasets were preprocessed using fastp v.0.23.2 [65] with adapter and polyG tail trimming, and low-quality reads were removed if the length was less than 60 bp or over more than 30% of them had an average Phred quality score of less than 20. The preprocessed reads were then aligned to the human genome (GRCh38) and the *Escherichia* phage phiX174 genome (NCBI accession NC_001422.1). Any reads that mapped to these genomes were excluded, and the remaining reads were recognized as clean reads for each sample. Next, the clean reads from each sample were subjected to contig assembly using MEGAHIT v1.2.967 [66] with the parameters: “–k-list 21,41,61,81,101,121,141”.

### Identification and clustering of viral sequences

We identified viral sequences using a well-established method described in our previous studies [18, 26]. Only contigs exceeding a length of 2000 bp were used for this analysis. Firstly, these contigs were assessed using CheckV v0.7.0 [28]. Those containing more than ten host genes, exceeding five times the number of viral genes, were excluded from further consideration. Among the remaining contigs, potential viral sequences were identified if they met any of the following criteria: (1) contigs with a higher count of viral genes in comparison to host genes, as determined by CheckV v0.7.0 [28]; (2) contigs with a score > 0.90 and a *p*-value < 0.01 in DeepVirFinder v1.0 [67]; and (3) contigs that were identified as viruses using default parameters in VIBRANT v1.2.1 [68]. To minimize non-viral sequence contamination, we conducted a search for bacterial universal single-copy orthologs (BUSCOs) within the potential viral sequences using hmmsearch [69]. We then calculated the BUSCO ratio, which measures the ratio of BUSCO counts to the total gene counts within each viral sequence, to assess potential contamination levels. Any sequence exhibiting ≥ 5% BUSCO ratio was then removed from the analysis. The remaining sequences, which possessed a CheckV-estimated completeness exceeding 50%, were assigned as the final viral genomes. To further improve the quality of the viral genomes and deduplicate genomes, we utilized a large gut viral genome database known as cnGVC [26]. In short, we clustered the viral genomes identified in our study with those in cnGVC based on criteria of 95% identity and 70% coverage. Only clusters containing at least one viral genome identified in our study were retained. Within each cluster, the longest sequence was selected as the reference sequence, which was referred to as the vOTU. In the end, we conducted a quality assessment of the genomes for all vOTUs through CheckV v0.7.0.

### Taxonomic and functional annotation and host prediction for viral sequences

The taxonomic annotation of vOTUs was conducted by aligning their protein sequences against a comprehensive database. This database was constructed by integrating proteins from various sources, including Virus-Host DB (acquired in May 2021) [70], crAss-like proteins from Guerin’s study [71], as well as viral proteins from Benler’s and Ye’s studies [71, 72]. To predict protein-coding sequences within vOTUs, we employed Prodigal v2.6.3 with the “meta” parameter [73]. Subsequently, we queried these protein sequences against the combined database using diamond v2.0.13.151 with the following parameters: “–id 30 –query-cover 50 –subject-cover 50 –min-score 50” [74]. For small vOTUs containing fewer than 30 genes, we assigned them to a known viral family if more than one-fifth of their proteins matched to a certain family. Conversely, for large vOTUs with 30 or more genes, we assigned them to a known viral family if at least 10 of their proteins matched to the same family.

To elucidate the functions of putative proteins, we used diamond v2.0.13.151 for searches against the KEGG (Kyoto Encyclopedia of Genes and Genomes) database [75], applying criteria of > 50% query coverage and > 60% score. For proteins that remained unassigned to a specific KEGG ortholog (KO), we conducted additional investigations by utilizing the PfamScan tool against Pfam database version 33.1 [34, 76].

We conducted host matching for vOTUs using two criteria within the pool of 4644 prokaryotic species from the Unified Human Gastrointestinal Genome (UHGG) database [31]. Firstly, for prokaryotic genomes, we predicted CRISPR spacer sequences using MinCED v0.4.2 with the parameter “-minNR 2” [77]. When a CRISPR spacer sequence from the host displayed a BLASTn match to a viral genome with a bit-score of 45 or greater, we assigned that virus to the corresponding host. Secondly, the virus was linked to a host if its sequence demonstrated ≥ 90% nucleotide identity and ≥ 30% viral coverage when compared to the host genome.

### Taxonomic composition of metagenomic samples

In order to mitigate the issue of false positives, we have taken three steps to regenerate the relative abundance table of vOTUs. The specific steps for generating relative abundances of vOTUs for a metagenomic sample are as follows: (1) we used Bowtie 2 with the options “–end-to-end –fast” to align all clean reads to the 10,054 vOTUs and then utilized Samtools to obtain the sequencing depth of each base position for every vOTU (including positions with a sequencing depth of 0). For each vOTU, all base positions were sorted based on their sequencing depths from lowest to highest, excluding positions in the lowest and highest 10% of depth ranges. Subsequently, the mean depth of the remaining base positions was calculated as the sequencing depth for this vOTU. (2) Additionally, all clean reads were aligned to the 10,054 vOTUs using Kraken with option “–confidence 0.1”. If no reads were specifically assigned to a particular vOTU, its sequencing depth obtained in the first step was set to 0. (3) Finally, the relative abundance of each vOTU was calculated by dividing its sequencing depth by the sum of the sequencing depths of all vOTUs. This calculation determined the relative abundance of each vOTU. For the family-level profiles, we aggregated the relative abundances of vOTUs that shared the same family-level annotation.

### Statistical analysis

R language (version 4.2.3) was employed for all statistical tests and data visualization in this study.

#### Alpha diversity

We quantified the number of observed vOTUs per sample by counting those vOTUs that exhibited relative abundances not equal to 0. We derived the Shannon index from the vOTU-level relative abundance profile by applying the *diversity* function with the “index = shannon” parameter. Notably, in computing alpha diversity indexes for the eukaryotic or prokaryotic virome, we exclusively rely on the relative abundance profile of vOTUs assigned to the eukaryotic or prokaryotic families.

#### Beta diversity

The vOTU-level relative abundance profile was first subjected to square root transformation. Subsequently, Bray–Curtis distances between samples were computed based on the transformed data using the *vegdist* function. Using this distance matrix, we conducted a PERMANOVA using the *adonis* function. Similarly, based on the same distance matrix, PCoA was carried out using the *pcoa* function from the *ape* package. The PCoA plot was generated using the *ggplot* function. Within the plot, we applied the *stat_ellipse* function to include ellipses around each group’s centroid, displaying an 80% confidence interval.

#### Statistical test

To compare the alpha diversity indexes and relative abundances of the viral community between groups, we conducted a Wilcoxon rank-sum test using the *wilcox.test* function. The resulting *p*-values were adjusted for multiple comparisons using the *p.adjust* function with the “method = B-H” parameter (Benjamini–Hochberg method). Spearman correlation analysis was conducted using the *cor.test* function with the “spearman” parameter to evaluate the association between the alpha diversity index of the gut virome and bacteriome. In the functional comparison between IBD-enriched and control-enriched vOTUs, we first calculated the occurrence ratio of each function within each group of viruses. This occurrence ratio is defined as the number of vOTUs possessing the corresponding functional gene divided by the total number of vOTUs within that specific group. Fisher’s exact test was applied to compare the occurrence ratios of each function between IBD-enriched and control-enriched vOTUs using the *fisher.test* function.

#### Random forest model

We additionally downloaded data from nine cohorts, including eight bulk datasets and one VLP dataset. Random forest model was constructed separately for each of the 11 datasets, including our 2 datasets, to perform classification predictions for IBD patients and healthy controls. The model, along with five repeats of fivefold cross-validation, was built based on IBD-associated vOTUs using the *randomForest* function. The importance of IBD-associated vOTUs was assessed using the mean decrease in accuracy (MDA) index, which was obtained using the *importance* function. Subsequently, the importance rank of IBD-associated vOTUs was determined based on the average of MDA indexes derived from the results of five repeats of fivefold cross-validation. To assess the performance of the model, we calculated the AUC using the *roc* function from the *pROC* package. Additionally, we assessed the performance of the cross-cohort model for disease classification. This involved training the model on one dataset and validating it on another dataset. For the leave-one-dataset-out analysis (LODO), we built the model using a training dataset that consisted of data from ten cohorts and subsequently assessed its performance on the remaining cohort.

### Animal experiments

All animal studies were approved by the Ethical Committee of Experimental Animal Care of Dalian Medical University (AEE19044).

#### Preparation of FVT materials and counting of VLPs

Three grams of pooled fecal samples were placed into a sterile 50-mL centrifuge tube. Thirty milliliters of SM buffer were added to create a fecal suspension. The suspension was left to stand on ice for 2 h with two intermittent shakes during this period. Afterward, centrifugation was performed at 5000 × *g* for 5 min at 4 °C. The supernatant was then transferred to a new sterile 50 mL-centrifuge tube and subjected to centrifugation at 12,000 × *g* for 15 min at 4 °C, and the supernatant was transferred to a new sterile 50-mL centrifuge tube. This step was repeated. The samples were then filtered through 0.45-μm and 0.22-μm filter membranes. The samples were then subjected to ultracentrifugation (750,000 × *g*, 60 min), followed by resuspension in saline solution. For counting the virus component prepared in the previous step, fixation was carried out using formalin. Subsequently, filtration was performed using a 0.02-μm filter (Whatman). Afterward, staining was done using SYBR Gold (Thermo Scientific) dye, and images were captured using an epifluorescence microscope for counting. Based on these results, the virus particle concentration for FVT was adjusted to approximately 1 × 10^9^/mL. For animal experiments, 200 μL was administered by gavage each time per mouse.

#### DSS-induced colitis model

Healthy male C57BL/6 mice were maintained under SPF conditions for 1 week prior to the experiment. Mice received an antibiotics cocktail in the drinking water for 5 days (neomycin 1 mg/mL, streptomycin 1 mg/mL, and bacitracin 1 mg/mL) to deplete gut microbiota. Then, mice received FMT from either IBD patients or healthy controls for 21 days to generate HMA mice, followed by a high-dose FVT for 5 more days. After FMT and FVT, colitis was induced by 2% (w/v) DSS in the drinking water for 7 days, followed by 2 days of regular drinking water before sacrifice. Throughout the experiment, consistent daily dosing was maintained, and changes in the mice’s body weight, diarrhea, and rectal bleeding were recorded. At the end of the experiment, the mice were anesthetized, and a colonoscopy was performed. Subsequently, the mice were euthanized, and the colon were collected and the colon length was measured. Approximately 1 cm segments of the colon were placed in formaldehyde fixation solution for histological analysis, while the remaining portions were placed in centrifuge tubes and stored at − 80 °C for subsequent experiments.

#### Microbial compositional analysis

Fecal specimens of mice were collected before and after the FVT procedures. Each fecal samples underwent for microbial DNA extraction, DNA library preparation, shotgun metagenomic sequencing, and data quality control using the aforementioned methods employed for bulk metagenomic sequencing of human feces. To determinate the viral composition of the mouse fecal samples, sequencing reads were mapped to the vOTU catalog of this study using Bowtie 2, and the read count for each vOTU were generated. For each sample, the relative abundances of IBD-enriched and control-enriched vOTUs were calculated by summing the read count of each type of vOTUs and then dividing by the total number of reads of that sample. Similarly, the bacterial composition was determined by mapping the sequencing reads to the UHGG database. Due to the substantial difference in genome size between viruses and bacteria, directly comparing their relative abundances in samples cannot adequately reflect their differences. Therefore, we used the virus-to-bacterium ratio to reflect the difference of “cell count” between viruses and bacteria in each fecal sample. For each virus or bacterium, the number of mapped reads was divided by its genome length to obtain an estimate of “cell count”. The sum of “cell counts” for all viruses was divided by the sum of “cell counts” for all bacteria to obtain the virus-to-bacterium ratio.

#### DAI scores

The disease activity index (DAI), including body weight loss, diarrhea, and rectal bleeding, was assigned scores on a 0–4 scale. Body weight: 0, body weight loss ≤ 0; 1, body weight loss ≤ 5; 2, body weight loss ≤ 10; 3, body weight loss ≤ 15; and 4, body weight loss > 15. Diarrhea: 0, normal stool; 1, soft but still formed stool; 2, soft stool; 3, very soft and wet stool; and 4, watery stool. Rectal bleeding: 0, no bleeding; 1, positive hemoccult; 2, visible blood traces in stool; 3, visible blood traces that adhered to the anus; and 4, gross bleeding.

#### Hematoxylin and eosin (HE) staining

Fixed colon segments were fixed in 10% buffered formaldehyde solution, embedded in paraffin, and sectioned. Sections were stained with HE for pathological lesion assessment. Pathological injures (crypt damage, inflammation, and ulceration) were assigned scores on a 0–3 scale by experienced and blinded researchers used a pathology scoring method as follows [78]. Inflammation was scored as follows: 0 = none; 1 = slight; 2 = moderate; and 3 = severe. Crypt damage was assigned scores as follows: 0, normal; 1, mild to moderate crypt loss (basal 1/3 damage); 2, severe crypt loss (basal 2/3 damage); and 3, complete crypt loss. Ulceration was assigned scores as follows: 0, normal; 1, destruction of mucosa; 2, destruction of musularis mucosa layer; and 3, destruction of submucosa.

#### qPCR

The levels of inflammatory cytokines (TNFα, IL1β, IL6, IFNγ) in the colon were determined by qPCR assays. Approximately 25 mg of colon tissue was placed in 500 μL of Trizol to obtain total RNA. A total of 1000 ng of total RNA was reverse-transcribed into cDNA using a reverse transcription kit according to the manufacturer’s instructions. The primers used for qPCR were designed as follows: IL1β: Forward 5'-TGCCACCTTTTGACAGTGATG-3',  Reverse 5'-TGATGTGCTGCTGCGAGATT-3'; IL6: Forward 5'-GGGACTGATGCTGGTGACAA-3', Reverse 5'-ACAGGTCTGTTGGGAGTGGT-3'; TNFα:  Forward 5'-TAGCCCACGTCGTAGCAAAC-3', Reverse 5'-TGTCTTTGAGATCCATGCCGT-3'; INFγ: Forward 5'-GGAGGAACTGGCAAAAGGATG-3',  Reverse 5'-GTTGCTGATGGCCTGATTGT-3'; β-actin: Forward 5'-CACCATGTACCCAGGCATTG-3', Reverse 5'-CCTGCTTGCTGATCCACATC-3'. Cycle threshold (Ct) values were recorded and normalized to the levels of internal reference β-actin.

#### Western blot

The protein levels of adhesion and tight junction-related proteins (occludin, E-cadherin, β-catenin) were determined by Western blot. Approximately 25 mg of colon tissue was mixed with cell lysis buffer containing PMSF and protease cocktail to obtain the total proteins. The protein concentration was determined using a BCA kit. Subsequently, protein samples were loaded onto the sodium dodecyl sulfate–polyacrylamide gel electrophoresis (SDS-PAGE) gel to undergo electrophoresis. Then, the interesting gel was transferred into PVDF membranes and incubated with primary antibody and secondary antibody sequentially. Finally, the bands were visualized using the Tanon 5200 ECL detection system (Tanon, China) and then the semiquantitative analyses were carried out using the Image J software.

### Supplementary Information

Additional File 1: Supplementary Figure 1. Comparison of gut viromes between VLP and bulk datasets. (a) Principal coordinate analysis (PCoA) reveals the difference between the VLP virome and bulk virome at the family level. Samples are shown at the first and second principal coordinates (PCoA1 and PCoA2), and the ratio of variance contributed by these two PCs is shown. (b) Observation of Microviridae vOTUs in the VLP and bulk datasets. Supplementary Figure 2. Correlation analysis (Spearman correlation test) of the gut bacteriome diversity and VLP/bulk virome diversity. For each panel, scatter plot shows the samples and the fitting line are formed based on the diversity indexes in the bacteriome and virome for all samples. Supplementary Figure 3. Comparison of Anelloviridae vOTUs between IBD patients and healthy controls. Barplot showing the number of vOTUs enriched in IBD patients or healthy controls. Dark red or blue indicate vOTUs with a p-value of less than 0.05 from the Wilcoxon rank-sum test comparing the two groups. Supplementary Figure 4. Comparison of gut viral composition between IBD patients and healthy controls at the order level. Wilcoxon rank-sum test: *, *p*<0.05; **, *p*<0.01; ***, *p*<0.001. HC, healthy controls. Supplementary Figure 5. Distribution of prokaryotic hosts of the IBD-associated vOTUs. Supplementary Figure 6. Comparison of functions between IBD-enriched and control-enriched vOTUs. Principal coordinate analysis (PCoA) reveals the differences in functional profiles between IBD-enriched and controls vOTUs. vOTUs are shown at the first and second principal coordinates (PCoA1 and PCoA2), and the ratio of variance contributed by these two PCs is shown. Statistical significance was obtained by PERMANOVA analysis. Supplementary Figure 7. Partial gene structures of several control-enriched vOTUs with the RNA-dependent DNA polymerase (RdDp) gene. The left-hand side text provides the family-level taxonomic annotation and vOTU ID number corresponding to each vOTU, along with the predicted prokaryotic host for each one. Supplementary Figure 8. Viral community variation among 9 public datasets and the VLP and bulk datasets in this study. Principal coordinates analysis (PCoA) based on the Bray-Curtis distance at the vOTU level. Effect size (R2) and statistical significance were obtained by PERMANOVA analysis. Cross-dataset IBD prediction based on 34 IBD-associated vOTUs. Supplementary Figure 9. Heatmap shows the performance assessed as AUC scores of intra-dataset and cross-dataset IBD predictions using random forest models trained based on 34 IBD-associated vOTUs. The models of intra-dataset prediction (diagonal) are validated using five repeats of five-fold cross-validations. The models of cross-dataset prediction (off-diagonal) are built on the dataset corresponding to each row and validated on the dataset corresponding to each column. The LOCO row refers to leave-one-cohort-out analysis in which models are built on ten datasets combined and validated on the remaining one corresponding to each column. Supplementary Figure 10. Bar plot showing the virus-to-bacterium ratio of feces of mouse before and after FVT. Supplementary Figure 11. Colitis scores of HE staining of the colon in Figure 5e. Supplementary Figure 12. Quantitative analysis of the western blot of Figure 5f. Supplementary Figure 13. Venn plot showing the overlap between our gut virus catalog and the existing gut virus catalogs.

Additional File 2: Table S1. Phenotypic characteristics of 71 IBD patients and 77 healthy controls recruited in this study. Table S2. Detailed information of 10,054 viral operational taxonomic units (vOTUs). Table S3. Comparison of the gut virome between IBD patients and healthy controls at the family level. Table S4. Detailed information of 139 IBD-associated vOTUs. Table S5. External validation cohorts for this study.

## Data Availability

The metagenomic sequencing data of human feces have been deposited in the European Nucleotide Archive (ENA) under the accession IDs PRJEB67456 (https://www.ebi.ac.uk/ena/browser/view/PRJEB67456). The analysis and visualization codes used in this study have been uploaded into the GitHub repository, accessible at: https://github.com/lish2/ibd_virome.
