# Supplementary material for: Gut virome-wide association analysis identifies cross-population viral signatures for inflammatory bowel disease
Source: Microbiome. 2024 Jul 18;12:130. doi: 10.1186/s40168-024-01832-x (PMC11256409; doi:10.1186/s40168-024-01832-x)
Supplement: Supplementary file 2 — Additional File 1: Supplementary Figure 1. Comparison of gut viromes between VLP and bulk datasets. (a) Principal coordinate analysis (PCoA) reveals the difference between the VLP virome and bulk virome at the family level. Samples are shown at the first and second principal coordinates (PCoA1 and PCoA2), and the ratio of variance contributed by these two PCs is shown. (b) Observation of Microviridae vOTUs in the VLP and bulk datasets. Supplementary Figure 2. Correlation analysis (Spearman correlation test) of the gut bacteriome diversity and VLP/bulk virome diversity. For each panel, scatter plot shows the samples and the fitting line are formed based on the diversity indexes in the bacteriome and virome for all samples. Supplementary Figure 3. Comparison of Anelloviridae vOTUs between IBD patients and healthy controls. Barplot showing the number of vOTUs enriched in IBD patients or healthy controls. Dark red or blue indicate vOTUs with a p-value of less than 0.05 from the Wilcoxon rank-sum test comparing the two groups. Supplementary Figure 4. Comparison of gut viral composition between IBD patients and healthy controls at the order level. Wilcoxon rank-sum test: *, p<0.05; **, p<0.01; ***, p<0.001. HC, healthy controls. Supplementary Figure 5. Distribution of prokaryotic hosts of the IBD-associated vOTUs. Supplementary Figure 6. Comparison of functions between IBD-enriched and control-enriched vOTUs. Principal coordinate analysis (PCoA) reveals the differences in functional profiles between IBD-enriched and controls vOTUs. vOTUs are shown at the first and second principal coordinates (PCoA1 and PCoA2), and the ratio of variance contributed by these two PCs is shown. Statistical significance was obtained by PERMANOVA analysis. Supplementary Figure 7. Partial gene structures of several control-enriched vOTUs with the RNA-dependent DNA polymerase (RdDp) gene. The left-hand side text provides the family-level taxonomic annotation and vOTU ID number corresponding to e [file 40168_2024_1832_MOESM1_ESM.docx]

**Gut virome-wide association analysis identifies cross-population viral signatures for inflammatory bowel disease**

Xiangge Tian ^1,2,+^, Shenghui Li ^3,+^, Chao Wang ^2,+^, Yanyan Zhang ^1,2,+^, Xiaoying Feng ^1^, Qiulong Yan ^1,2,*^, Ruochun Guo ^3^, Fan Wu ^1^, Chunxue Wu ^1^, Yan Wang ^1^, Xiaokui Huo ^1,2,*^, Xiaochi Ma ^1,2,*^

1, Second Affiliated Hospital, Dalian Medical University, Dalian 116023, China.

2, Dalian Key Laboratory of Metabolic Target Characterization and Traditional Chinese Medicine Intervention, College of Basic Medical Sciences, Dalian Medical University, Dalian 116044, China.

3, Puensum Genetech Institute, Wuhan 430076, China.

+ These authors contributed equally to this work.

* Correspondence: qiulongy1988@163.com (Q.Y.), huoxiaokui@163.com (X.H.), and maxc1978@163.com (X.M.)

**Supplementary Figures**


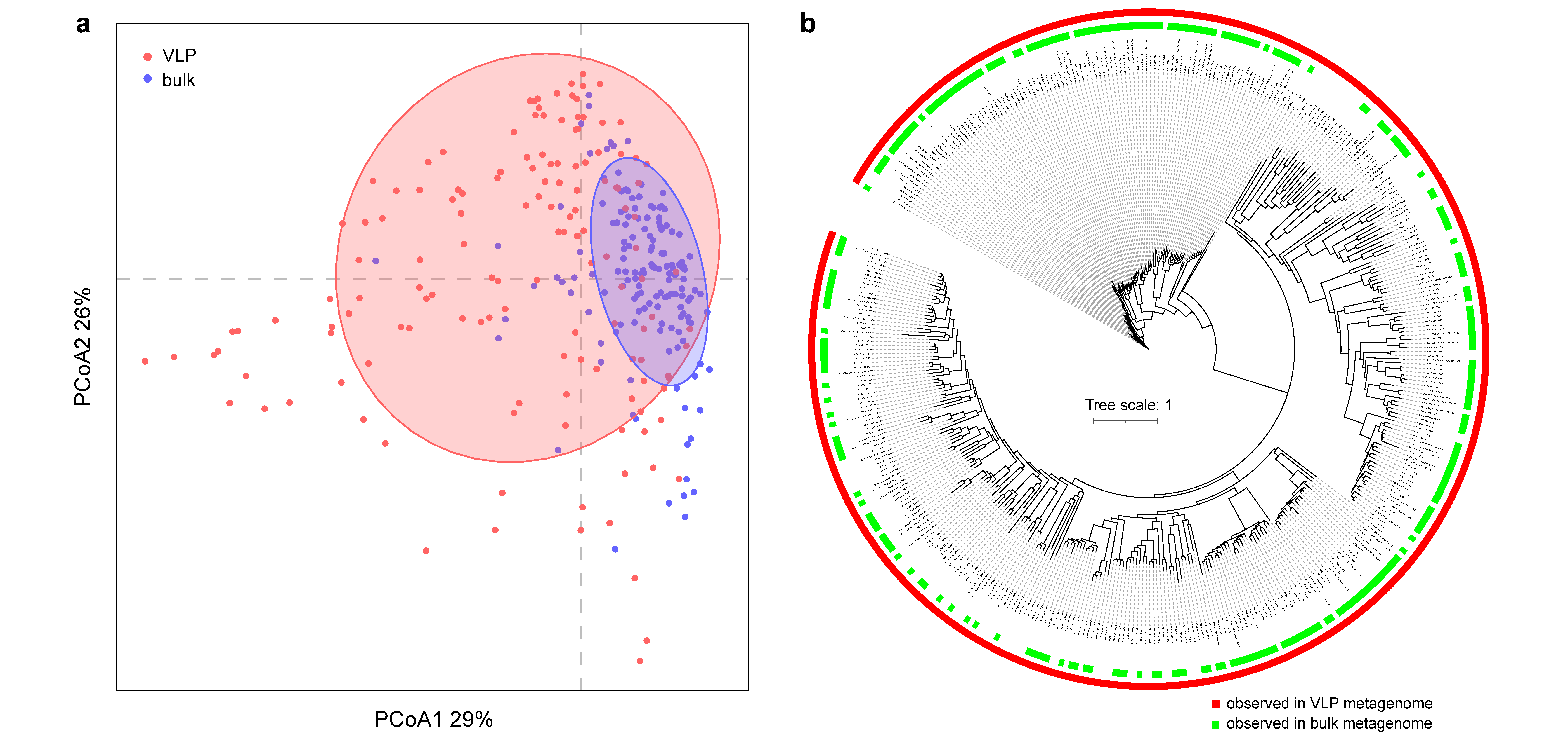


**Supplementary Figure 1| Comparison of gut viromes between VLP and bulk datasets. (a)** Principal coordinate analysis (PCoA) reveals the difference between the VLP virome and bulk virome at the family level. Samples are shown at the first and second principal coordinates (PCoA1 and PCoA2), and the ratio of variance contributed by these two PCs is shown. **(b)** Observation of *Microviridae* vOTUs in the VLP and bulk datasets.


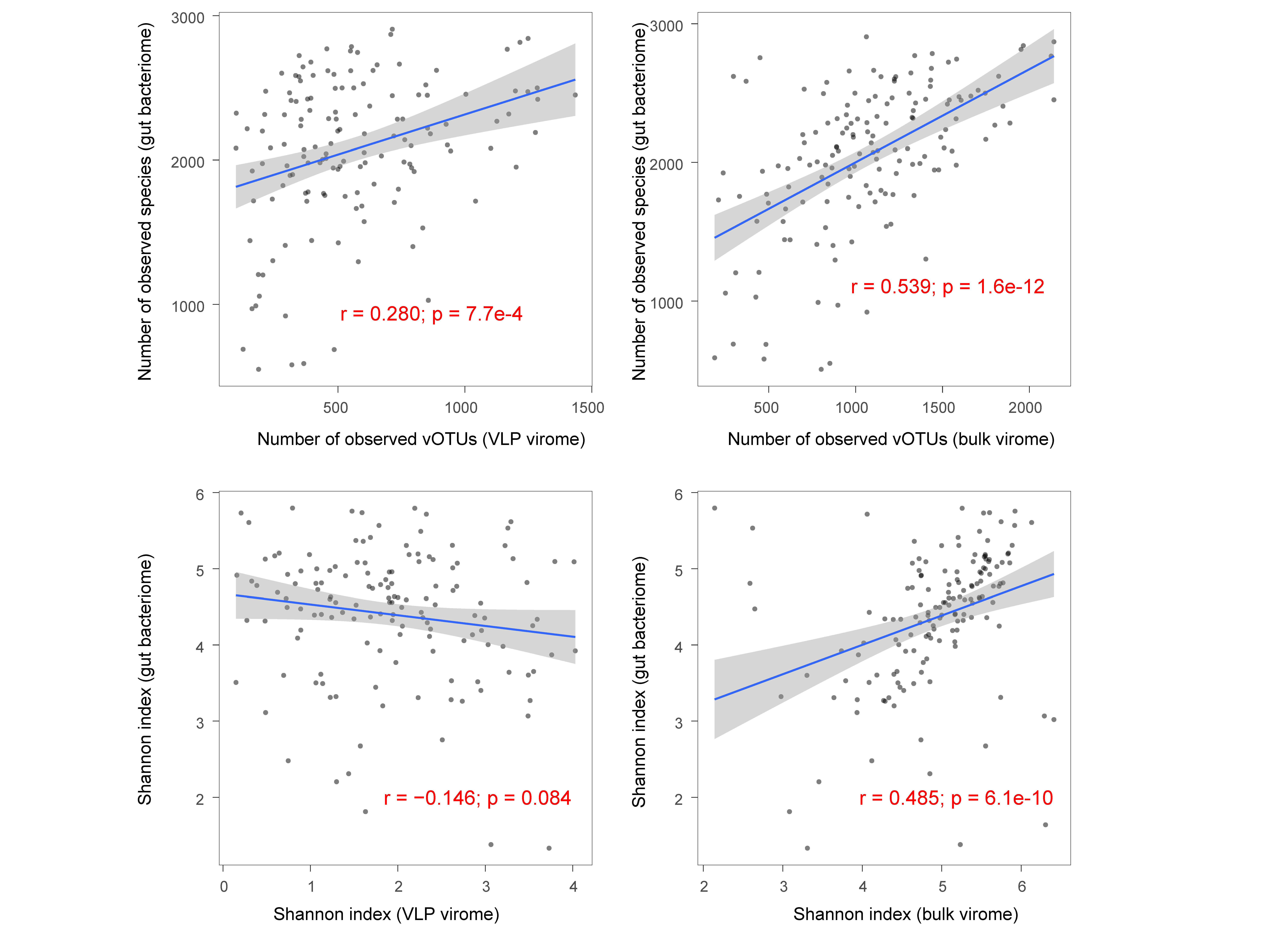


**Supplementary Figure 2| Correlation analysis (Spearman correlation test) of the gut bacteriome diversity and VLP/bulk virome diversity.** For each panel, scatter plot shows the samples and the fitting line are formed based on the diversity indexes in the bacteriome and virome for all samples.


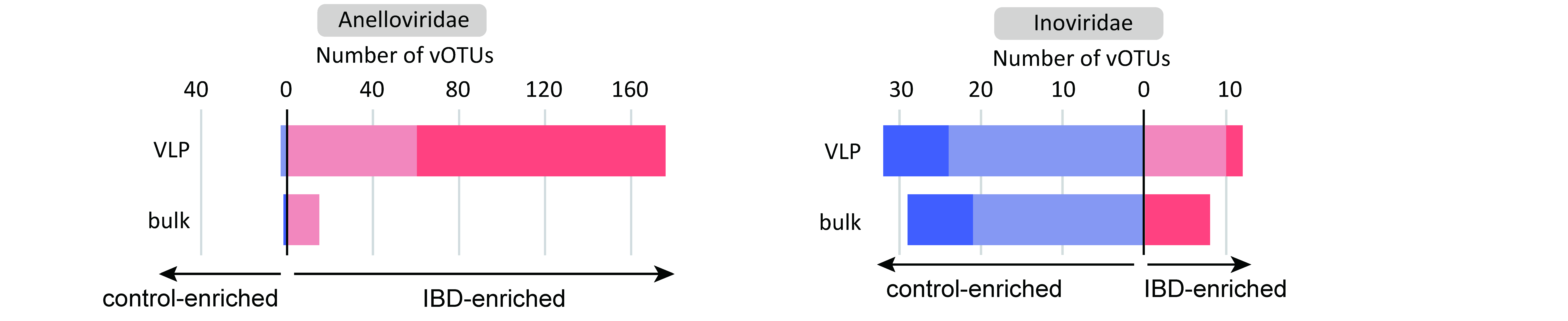


**Supplementary Figure 3|** Comparison of Anelloviridae and Inoviridae vOTUs between IBD patients and healthy controls. Barplot showing the number of vOTUs enriched in IBD patients or healthy controls. Dark red or blue indicate vOTUs with a p-value of less than 0.05 from the Wilcoxon rank-sum test comparing the two groups.


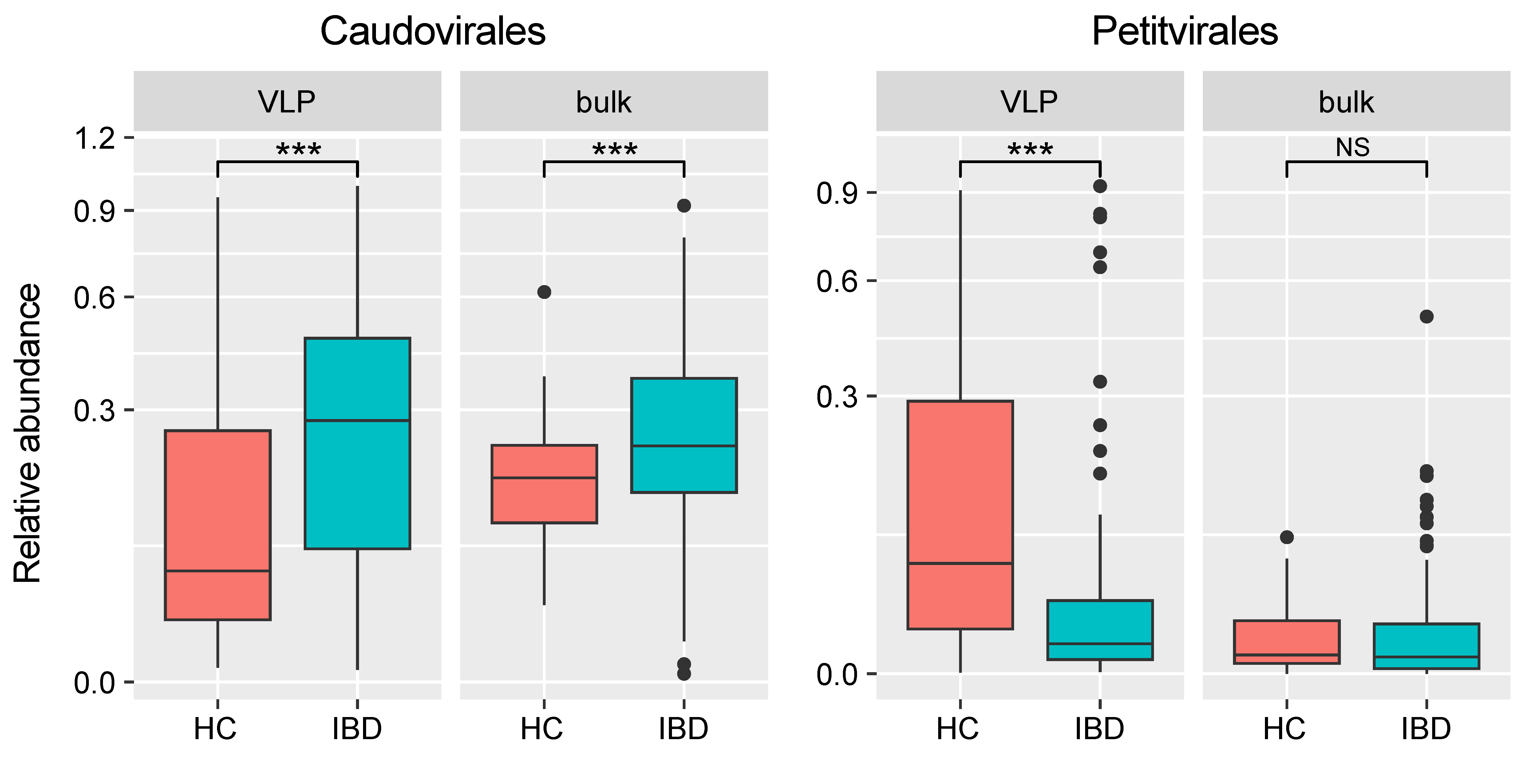


**Supplementary Figure 4|** Comparison of gut viral composition between IBD patients and healthy controls at the order level. Wilcoxon rank-sum test. *, p<0.05; **, p<0.01; ***, p<0.001. HC, healthy controls.


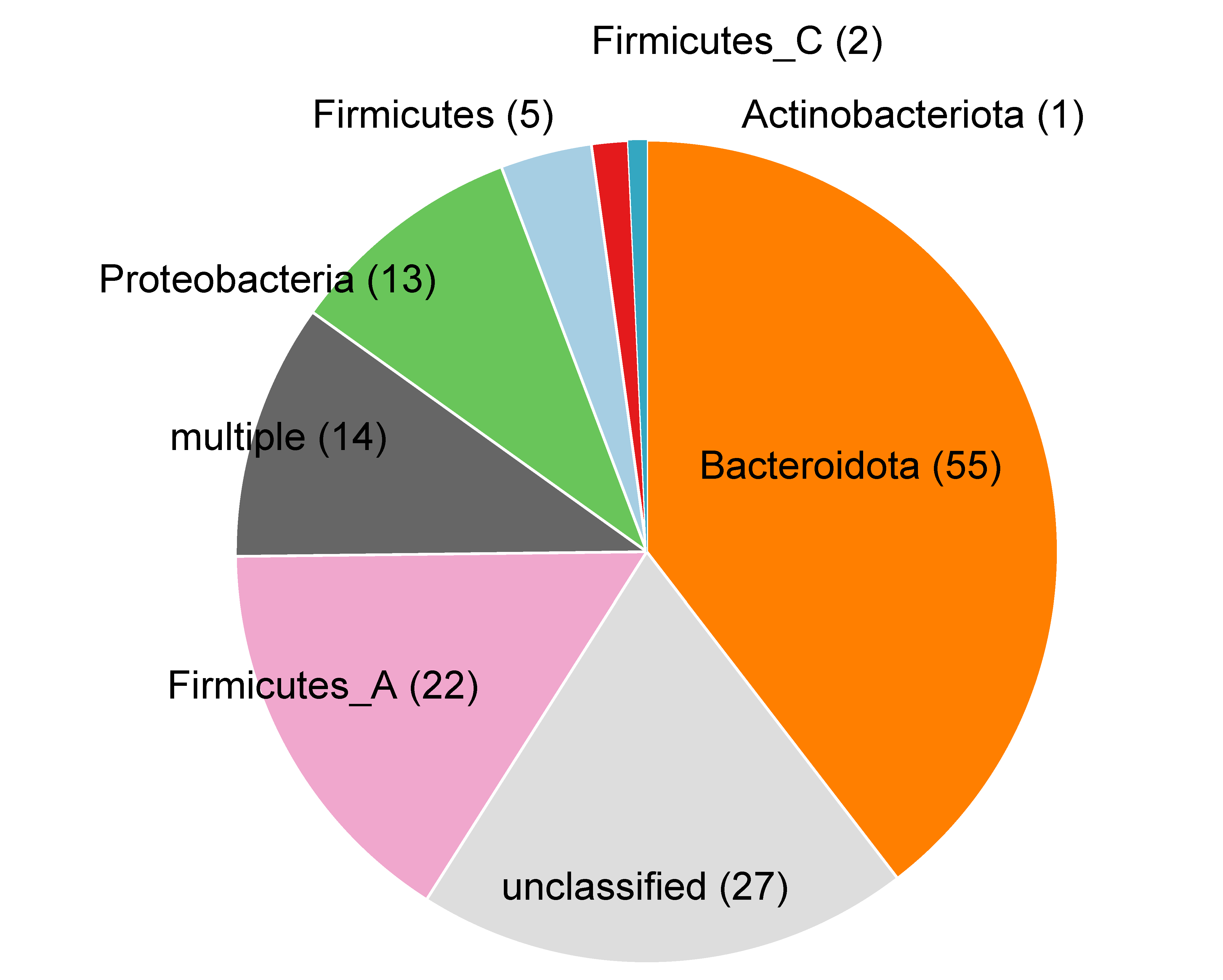


**Supplementary Figure 5| Distribution of prokaryotic hosts of the IBD-associated vOTUs.**


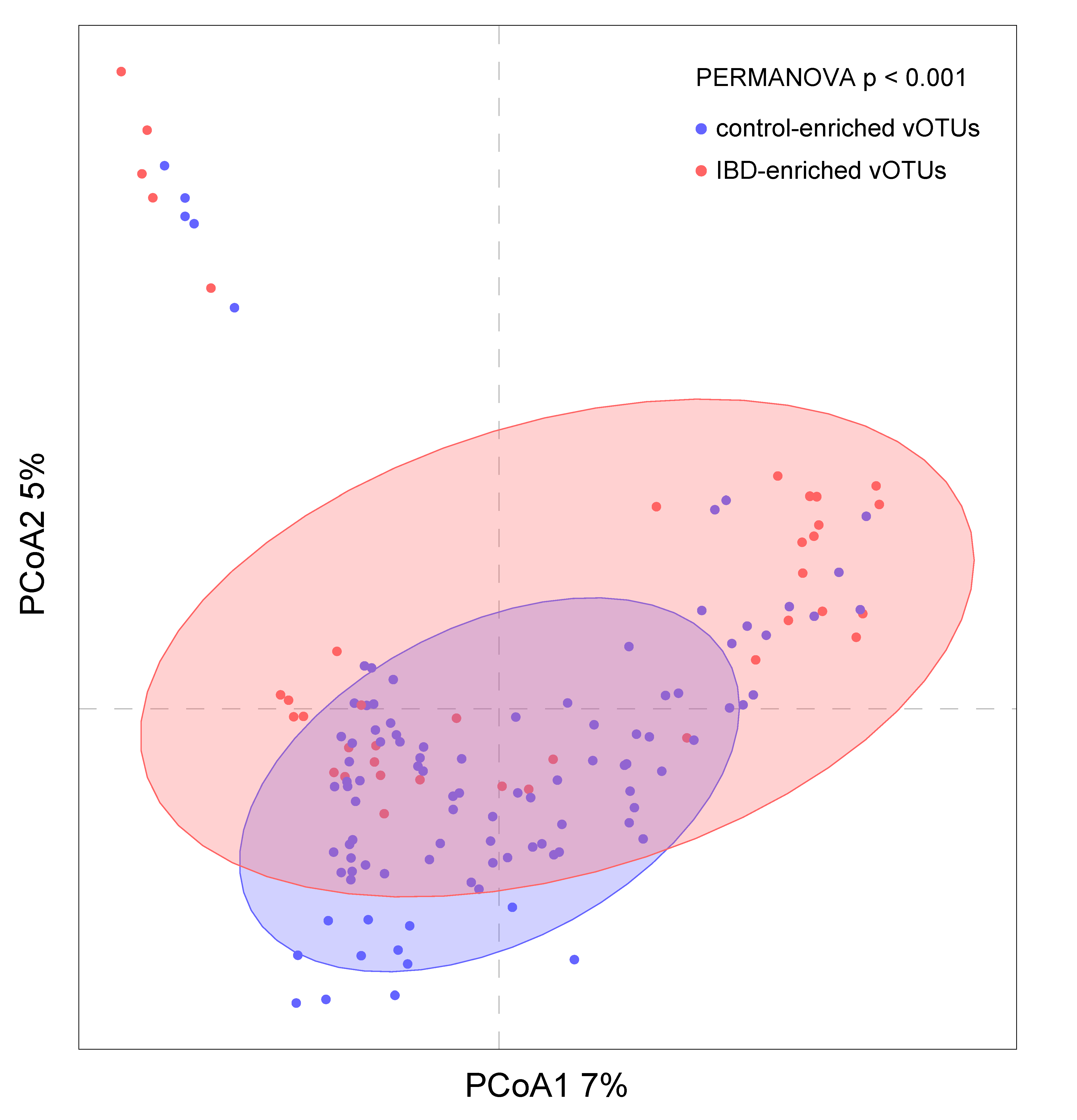


**Supplementary Figure 6| Comparison of functions between IBD-enriched and control-enriched vOTUs.** Principal coordinate analysis (PCoA) reveals the differences in functional profiles between IBD-enriched and controls vOTUs. vOTUs are shown at the first and second principal coordinates (PCoA1 and PCoA2), and the ratio of variance contributed by these two PCs is shown. Statistical significance was obtained by PERMANOVA analysis.


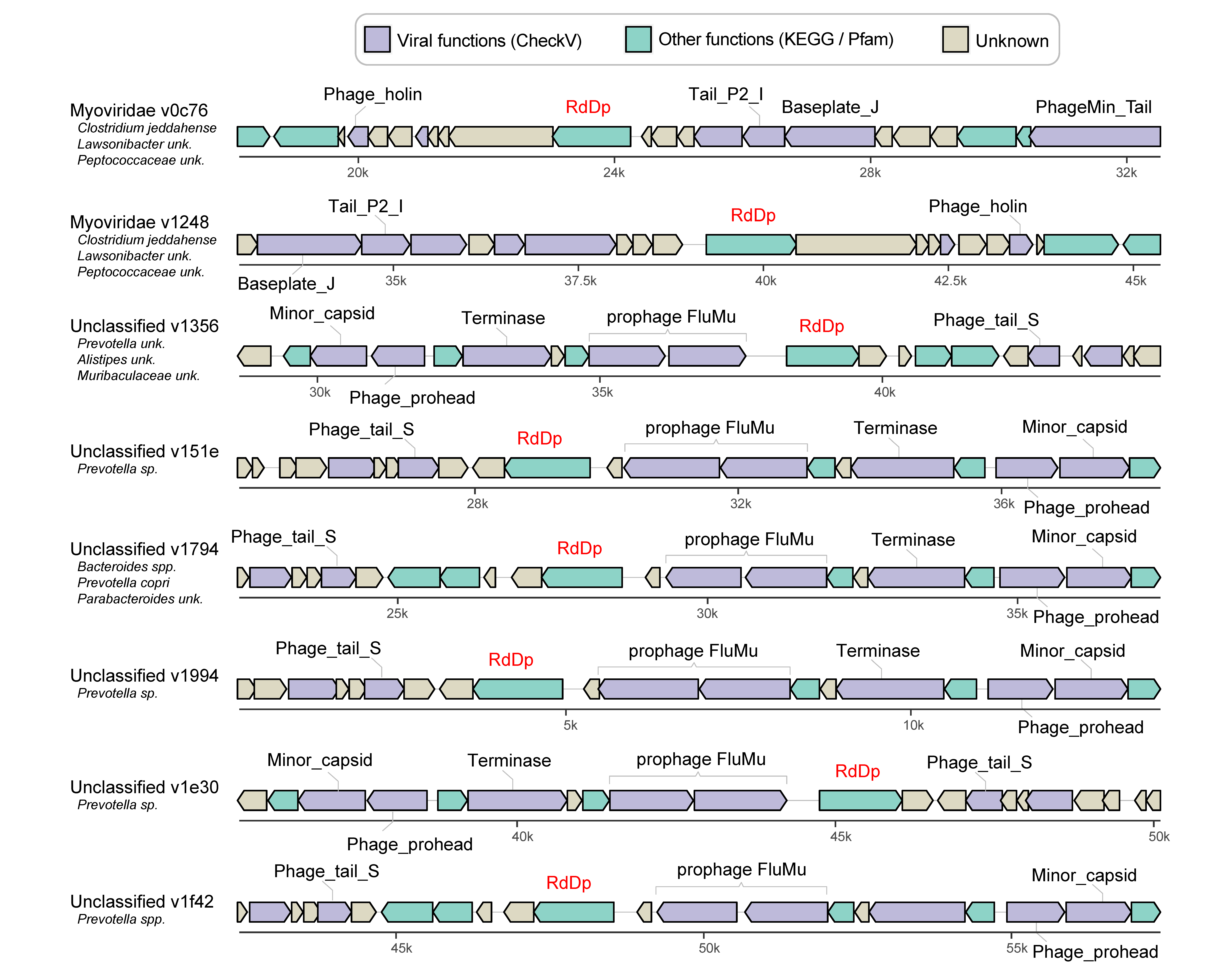


**Supplementary Figure 7| Partial gene structures of several control-enriched vOTUs with the RNA-dependent DNA polymerase (RdDp) gene.** The left-hand side text provides the family-level taxonomic annotation and vOTU ID number corresponding to each vOTU, along with the predicted prokaryotic host for each one.


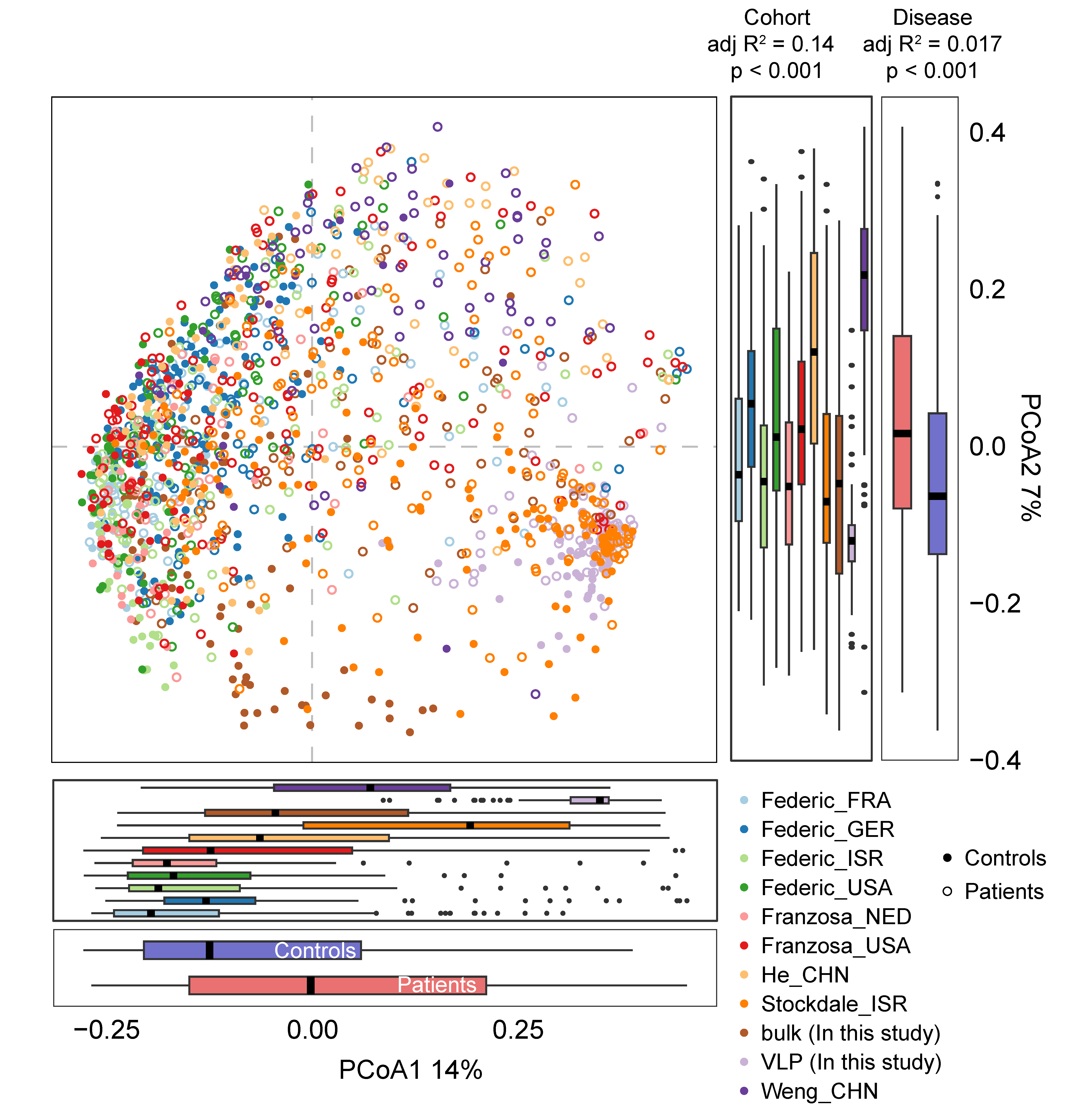


**Supplementary Figure 8| Viral community variation among 9 public datasets and the VLP and bulk datasets in this study.** Principal coordinates analysis (PCoA) based on the Bray-Curtis distance at the vOTU level. Effect size (R^2^) and statistical significance were obtained by PERMANOVA analysis.


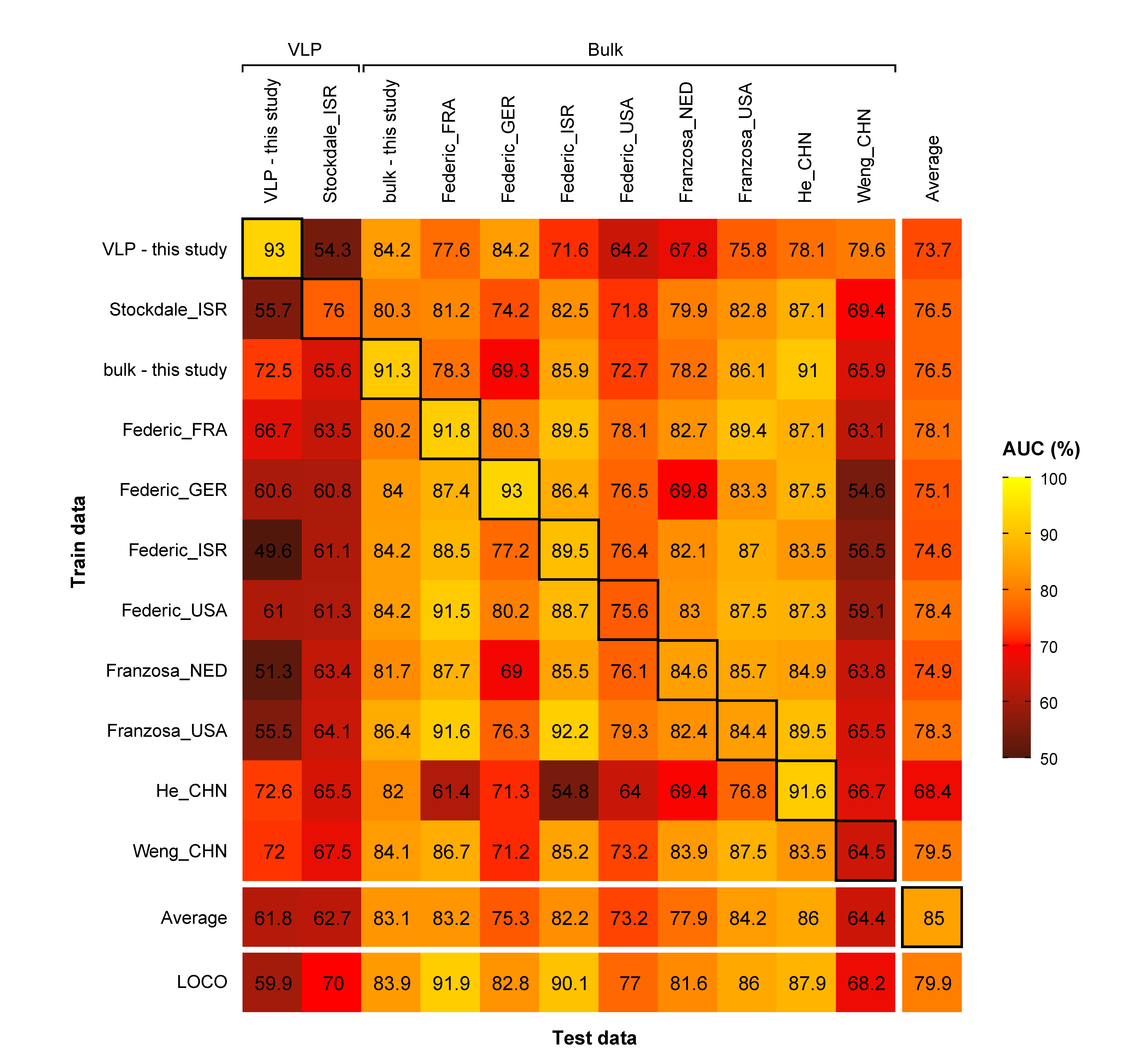


**Supplementary Figure 9| Cross-dataset IBD prediction based on 34 IBD-associated vOTUs.** Heatmap shows the performance assessed as AUC scores of intra-dataset and cross-dataset IBD predictions using random forest models trained based on 34 IBD-associated vOTUs. The models of intra-dataset prediction (diagonal) are validated using five repeats of five-fold cross-validations. The models of cross-dataset prediction (off-diagonal) are built on the dataset corresponding to each row and validated on the dataset corresponding to each column. The LOCO row refers to leave-one-cohort-out analysis in which models are built on ten datasets combined and validated on the remaining one corresponding to each column.


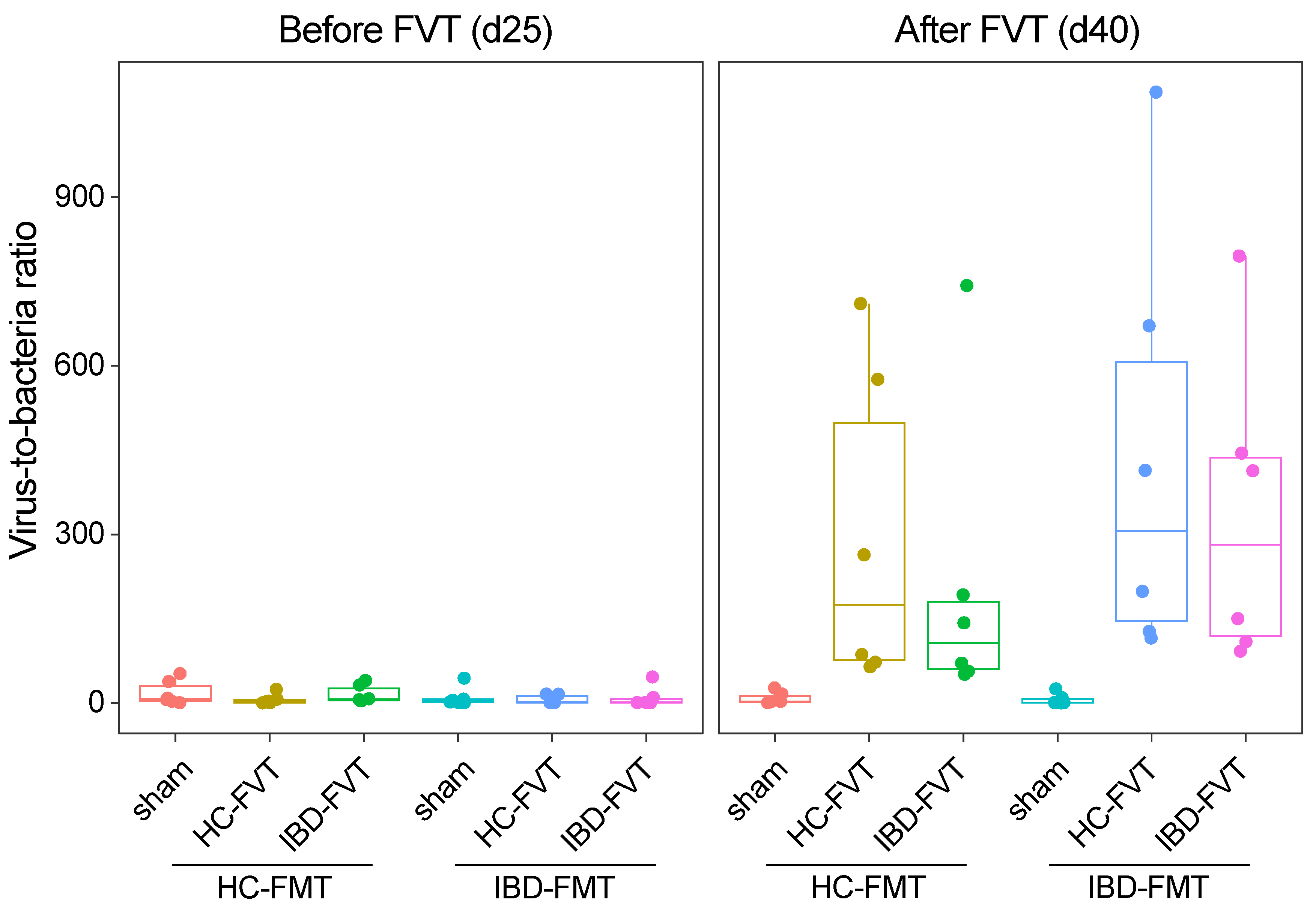


**Supplementary Figure 10| Barplot showing the virus-to-bacteria ratio of feces of mouse before and after FVT.**

**Supplementary Figure 11| Colitis scores of HE staining of the colon in Figure 5e.** Pathological injures (crypt damage, inflammation, and ulceration) were assigned scores on a 0-3 scale. N = 6. Statistical test was performed using Student’s t-test: *, p < 0.05; ***, p < 0.001.


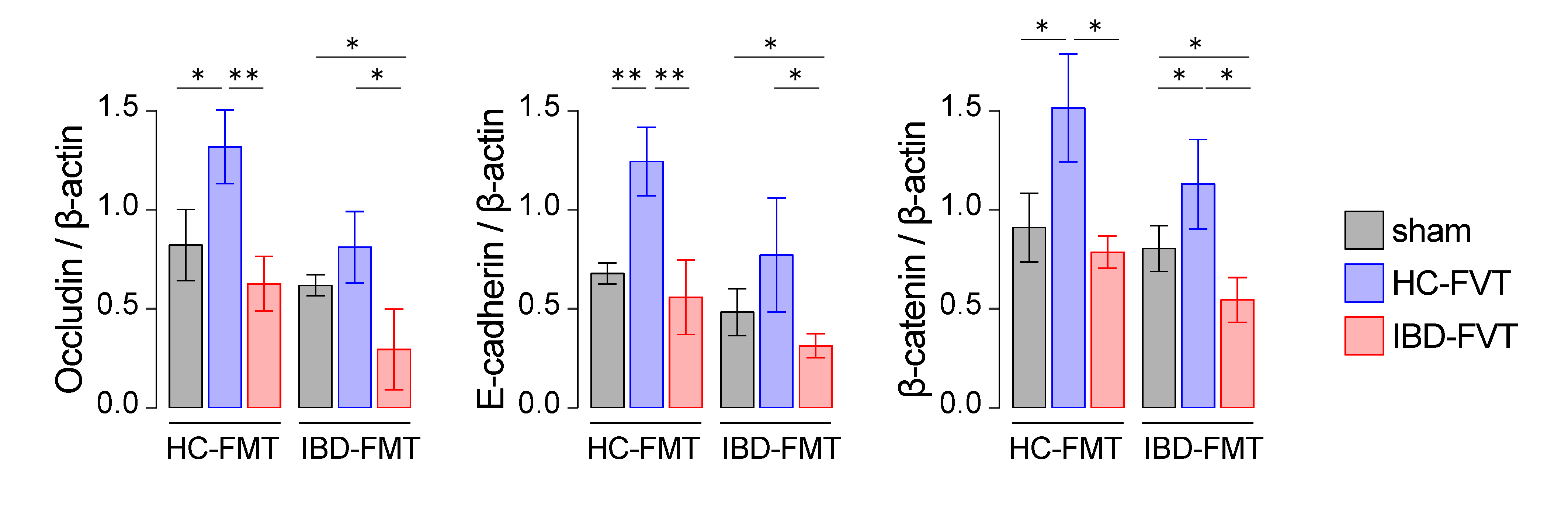


**Supplementary Figure 12| Quantitative analysis of the western blot of Figure 5f.** The data was the densitometry ratio of the interest protein and β-actin. N = 3. Statistical test was performed using Student’s t-test: *, p < 0.05; **, p < 0.01.


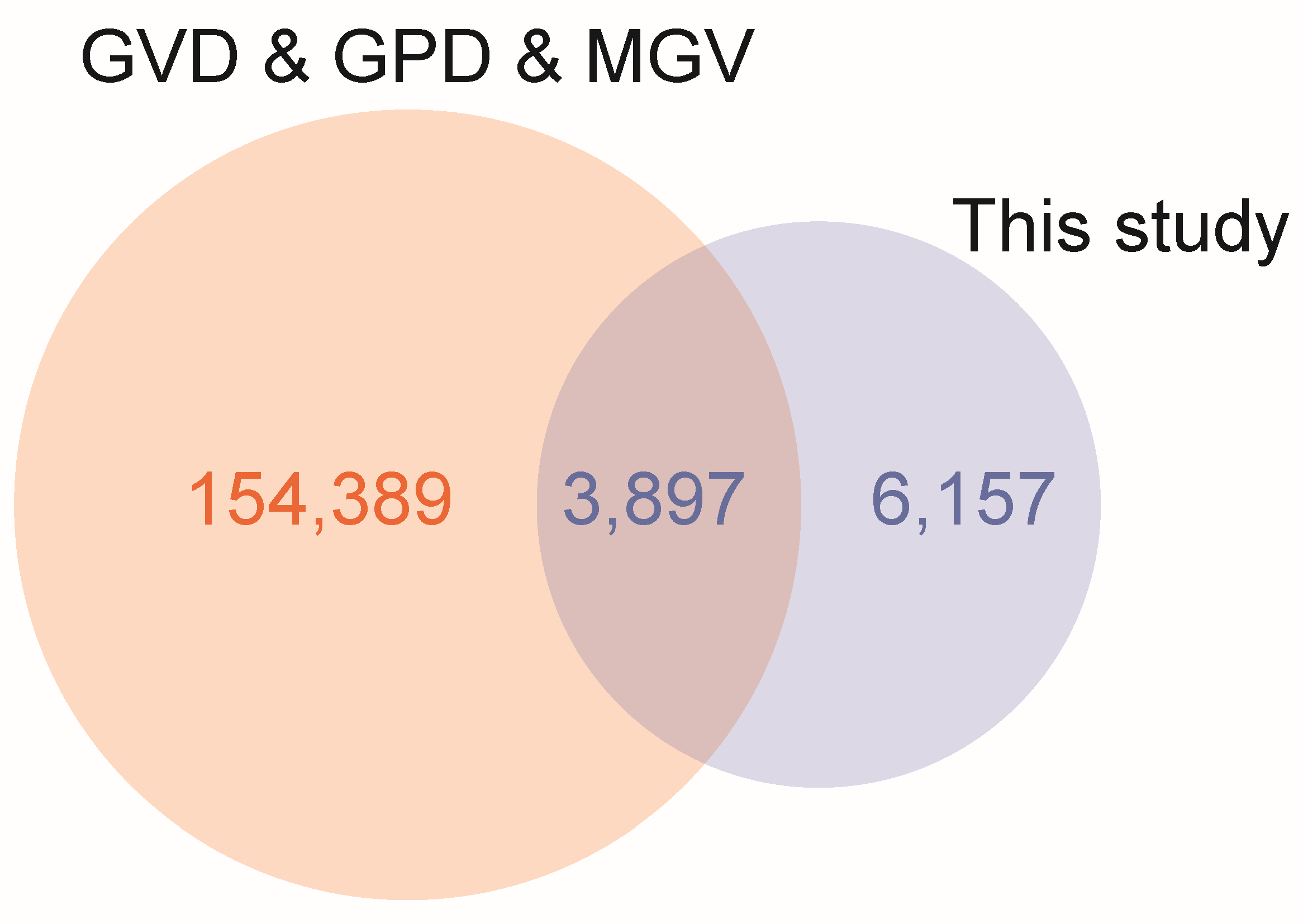


**Supplementary Figure 13| Venn plot showing the overlap between our gut virus catalogue and the existing gut virus catalogues.**
