# Supplementary material for: Gut virome-wide association analysis identifies cross-population viral signatures for inflammatory bowel disease
Source: Microbiome. 2024 Jul 18;12:130. doi: 10.1186/s40168-024-01832-x (PMC11256409; doi:10.1186/s40168-024-01832-x)
Supplement: Supplementary file 3 — Additional File 2: Table S1. Phenotypic characteristics of 71 IBD patients and 77 healthy controls recruited in this study. Table S2. Detailed information of 10,054 viral operational taxonomic units (vOTUs). Table S3. Comparison of the gut virome between IBD patients and healthy controls at the family level. Table S4. Detailed information of 139 IBD-associated vOTUs. Table S5. External validation cohorts for this study. [file 40168_2024_1832_MOESM2_ESM.pdf]

Supplementary Table 1| Phenotypic characteristics of 71 IBD patients and 77 healthy controls recruited in this study.

	Healthy controls	Patients with CD	Patients with UC	p-value (HC vs. IBD)	p-value (CD vs. UC)
**Number of samples (n)**	77	15	56	/	/
**Age, years (mean ± s.d.)**	51.9 ± 13.8	53.3 ± 14.4	52.3 ± 16.6	0.749	0.841
**Gender (% female)**	41.6%	40.0%	48.2%	0.62	0.772
**Body mass index, kg/m^2 (mean ± s.d.)**	21.45 ± 2.71	22.53 ± 2.52	23.02 ± 3.05	0.191	0.573
**Montreal classification (n)**	/	L1, n = 7<br>L2, n = 3<br>L3, n = 5	E1, n = 21<br>E2, n = 10<br>E3, n = 25	/	/
**CDAI group (n)**	/	remission, n = 5<br>moderate activity, n = 3<br>severe activity, n = 6	remission, n = 9<br>moderate activity, n = 34<br>severe activity, n = 12<br>unrecorded, n = 1	/	/

Supplementary Table 2| Detailed information of 10,054 viral operational taxonomic units (vOTUs).

vOTU ID	Length (bp)	Number of genes	Number of viral genes	Number of microbial genes	CheckV quality	% Completeness	% Contamination	Taxonomic assignment (family level)	Eukaryotic or prokaryotic virus
v0001	18644	19	18	0	Medium-quality	53.34	0	Adenoviridae	eukaryote
v0002	6378	6	3	0	Complete	100	0	unclassified	unclassified
v0003	2347	3	2	0	Complete	100	0	Circoviridae	eukaryote
v0004	5996	10	4	0	Complete	100	0	Microviridae	prokaryote
v0005	6696	8	2	0	Complete	100	0	Microviridae	prokaryote
v0006	6430	9	3	0	High-quality	100	0	unclassified	unclassified
v0007	2726	2	2	0	High-quality	93.97	0	Anelloviridae	eukaryote
v0008	2223	4	2	0	High-quality	100	0	Genomoviridae	eukaryote
v0009	6190	9	2	0	Complete	100	0	unclassified	unclassified
v000a	36039	60	20	0	High-quality	90.08	0	Siphoviridae	prokaryote
v000b	3374	4	4	0	Medium-quality	70.83	0	Microviridae	prokaryote
v000c	5864	8	1	0	Complete	100	0	unclassified	unclassified
v000d	5559	9	4	0	Medium-quality	86.95	0	Microviridae	prokaryote
v000e	4189	7	4	0	Medium-quality	75.13	0	unclassified	unclassified
v000f	30673	40	10	0	Complete	100	0	unclassified	unclassified
v0010	2721	4	4	0	Medium-quality	55.8	0	Microviridae	prokaryote
v0011	6530	9	2	0	High-quality	100	0	unclassified	unclassified
v0012	3558	3	2	0	High-quality	95.24	0	unclassified	unclassified
v0013	58783	89	27	0	High-quality	96.78	0	Siphoviridae	prokaryote
v0014	4951	9	6	0	Complete	100	0	Microviridae	prokaryote
v0015	4154	9	6	0	Medium-quality	84.66	0	Microviridae	prokaryote
v0016	41445	62	26	1	High-quality	98.82	0	Myoviridae	prokaryote
v0017	22935	24	5	0	Medium-quality	67.4	0	unclassified	unclassified
v0018	3469	4	1	0	Medium-quality	54.53	0	Microviridae	prokaryote
v0019	3604	4	2	0	Medium-quality	57.81	0	unclassified	unclassified
v001a	9269	12	3	0	High-quality	100	0	unclassified	unclassified
v001b	3041	6	5	0	Medium-quality	61.89	0	Microviridae	prokaryote
v001c	16712	19	4	0	Medium-quality	56.04	0	unclassified	unclassified
v001d	19325	26	8	1	High-quality	100	0	Podoviridae	prokaryote
v001e	49226	82	24	1	High-quality	97.99	0	unclassified	unclassified
v001f	7321	9	3	0	High-quality	100	0	unclassified	unclassified
v0020	9063	10	3	0	High-quality	100	0	unclassified	unclassified
v0021	3482	4	2	0	Medium-quality	59.54	0	unclassified	unclassified
v0022	4445	9	0	0	Medium-quality	71.85	0	unclassified	unclassified
v0023	5798	9	4	0	Complete	100	0	Microviridae	prokaryote

v0024	35008	48	19	1	High-quality	93.95	0	Siphoviridae	prokaryote
v0025	43496	60	18	0	High-quality	100	0	unclassified	unclassified
v0026	6858	6	4	0	Complete	100	0	unclassified	unclassified
v0027	90605	137	12	2	Complete	100	0	Quimbyviridae	prokaryote
v0028	26931	49	14	0	Medium-quality	72.52	0	unclassified	unclassified
v0029	97805	111	14	0	High-quality	99.15	0	Podoviridae_crAss-like	prokaryote
v002a	5880	6	3	0	Complete	100	0	Microviridae	prokaryote
v002b	41884	68	23	0	High-quality	100	0	Siphoviridae	prokaryote
v002c	5325	8	5	0	Complete	100	0	Microviridae	prokaryote
v002d	33149	58	12	1	High-quality	99.7	0	unclassified	unclassified
v002e	3907	3	1	0	Medium-quality	62.04	0	Microviridae	prokaryote
v002f	47156	68	20	2	Complete	100	0	Siphoviridae	prokaryote
v0030	7277	10	3	0	Complete	100	0	unclassified	unclassified
v0031	10130	12	5	0	Medium-quality	86.07	0	Podoviridae	prokaryote
v0032	30663	39	6	0	Medium-quality	71.21	0	unclassified	unclassified
v0033	18492	26	8	0	High-quality	100	0	Podoviridae	prokaryote
v0034	6331	7	3	0	Complete	100	0	unclassified	unclassified
v0035	23627	27	8	0	Medium-quality	52.25	0	unclassified	unclassified
v0036	6566	9	4	0	Complete	100	0	Microviridae	prokaryote
v0037	6691	8	3	0	High-quality	97.66	0	unclassified	unclassified
v0038	40193	75	13	1	High-quality	99.38	0	unclassified	unclassified
v0039	19288	25	6	0	Medium-quality	87.38	0	Podoviridae	prokaryote
v003a	3632	6	1	0	Medium-quality	55.62	0	unclassified	unclassified
v003b	6124	8	2	0	Complete	100	0	unclassified	unclassified
v003c	4943	8	6	0	Complete	100	0	Microviridae	prokaryote
v003d	10772	18	3	0	Medium-quality	63.76	0	unclassified	unclassified
v003e	4667	7	2	0	Medium-quality	72.91	0	unclassified	unclassified
v003f	5963	9	3	0	High-quality	100	0	unclassified	unclassified
v0040	34407	45	21	0	Medium-quality	57.19	0	Siphoviridae	prokaryote
v0041	5279	8	0	0	Complete	100	0	Inoviridae	prokaryote
v0042	5971	6	2	0	Complete	100	0	unclassified	unclassified
v0043	3401	6	5	0	Medium-quality	67.27	0	Microviridae	prokaryote
v0044	6529	9	4	0	Complete	100	0	unclassified	unclassified
v0045	34627	75	11	1	Medium-quality	60.55	0	unclassified	unclassified
v0046	41360	75	17	0	High-quality	98.09	0	unclassified	unclassified
v0047	2654	4	2	0	Medium-quality	52	0	Microviridae	prokaryote
v0048	5414	11	0	0	High-quality	100	0	Inoviridae	prokaryote
v0049	41256	67	17	0	Complete	100	0	unclassified	unclassified
v004a	152840	227	15	1	Medium-quality	78.93	0	unclassified	unclassified
v004b	6739	7	2	0	Complete	100	0	unclassified	unclassified

v004c	32311	63	14	0	Medium-quality	80.05	0	Siphoviridae	prokaryote
v004d	39034	52	6	0	High-quality	93.99	0	unclassified	unclassified
v004e	4320	10	0	0	Medium-quality	70.7	0	Inoviridae	prokaryote
v004f	38697	54	7	0	High-quality	100	0	unclassified	unclassified
v0050	5954	10	5	0	Complete	100	0	Microviridae	prokaryote
v0051	5090	5	3	0	Medium-quality	83.44	0	unclassified	unclassified
v0052	16273	19	9	0	Medium-quality	89.4	0	Rountreeviridae	prokaryote
v0053	5099	4	3	0	Medium-quality	79.49	0	unclassified	unclassified
v0054	79860	116	15	6	High-quality	100	0	unclassified	unclassified
v0055	42046	72	18	0	Complete	100	0	unclassified	unclassified
v0056	4637	9	7	0	High-quality	94.38	0	Microviridae	prokaryote
v0057	35640	52	24	0	Medium-quality	69.43	0	Myoviridae	prokaryote
v0058	45357	62	16	0	High-quality	93.51	0	unclassified	unclassified
v0059	4493	6	6	0	Medium-quality	88.33	0	Microviridae	prokaryote
v005a	62635	76	16	2	Complete	100	0	unclassified	unclassified
v005b	17094	26	10	0	Medium-quality	74.14	0	unclassified	unclassified
v005c	6226	7	3	0	Complete	100	0	Microviridae	prokaryote
v005d	100592	158	9	1	Complete	100	0	Podoviridae_crAss-like	prokaryote
v005e	5045	7	6	0	Complete	100	0	Microviridae	prokaryote
v005f	32111	45	14	0	Medium-quality	84.39	0	unclassified	unclassified
v0060	79231	124	13	2	Complete	100	0	unclassified	unclassified
v0061	43877	73	14	0	Complete	100	0	unclassified	unclassified
v0062	43515	74	16	0	Complete	100	0	unclassified	unclassified
v0063	4927	4	3	0	Medium-quality	80.79	0	unclassified	unclassified
v0064	10289	11	0	0	Medium-quality	66.71	0	unclassified	unclassified
v0065	4282	4	2	0	Medium-quality	68.96	0	unclassified	unclassified
v0066	5029	9	7	0	Complete	100	0	Microviridae	prokaryote
v0067	5129	8	0	0	Complete	100	0	Inoviridae	prokaryote
v0068	2993	5	1	0	Complete	100	0	Circoviridae	eukaryote
v0069	45251	72	16	0	Complete	100	0	unclassified	unclassified
v006a	5974	10	4	0	Complete	100	0	Microviridae	prokaryote
v006b	52258	74	8	2	High-quality	92.62	0	unclassified	unclassified
v006c	58069	99	19	1	Complete	100	0	unclassified	unclassified
v006d	14768	18	8	0	Medium-quality	63.86	0	unclassified	unclassified
v006e	37725	61	28	0	High-quality	100	0	Siphoviridae	prokaryote
v006f	17664	22	8	0	Medium-quality	76.75	0	unclassified	unclassified
v0070	5923	8	3	0	Complete	100	0	unclassified	unclassified
v0071	42186	45	5	0	High-quality	91.97	0	unclassified	unclassified
v0072	18882	28	8	0	High-quality	98.58	0	unclassified	unclassified
v0073	38884	58	19	1	Complete	100	0	Siphoviridae	prokaryote

v0074	7035	8	3	0	Complete	100	0	unclassified	unclassified
v0075	39828	50	11	1	High-quality	91.75	0	unclassified	unclassified
v0076	27353	38	13	0	Medium-quality	67.75	0	unclassified	unclassified
v0077	7664	8	4	0	High-quality	100	0	unclassified	unclassified
v0078	5212	9	3	0	High-quality	100	0	Microviridae	prokaryote
v0079	11481	14	5	0	High-quality	97.66	0	Podoviridae	prokaryote
v007a	42969	63	18	2	Medium-quality	85.62	0	unclassified	unclassified
v007b	42212	52	6	0	Complete	100	0	Siphoviridae	prokaryote
v007c	5649	9	6	0	Complete	100	0	Microviridae	prokaryote
v007d	5589	9	7	0	Complete	100	0	Microviridae	prokaryote
v007e	5333	8	4	0	Complete	100	0	Microviridae	prokaryote
v007f	57843	55	7	3	Medium-quality	83.09	0	unclassified	unclassified
v0080	150715	189	41	4	High-quality	100	0	unclassified	unclassified
v0081	80673	107	11	2	Complete	100	0	Quimbyviridae	prokaryote
v0082	32325	46	15	0	Medium-quality	71.01	0	unclassified	unclassified
v0083	17516	23	6	0	Medium-quality	58.96	0	unclassified	unclassified
v0084	103220	153	28	1	Complete	100	0	Podoviridae_crAss-like	prokaryote
v0085	16623	22	6	0	Medium-quality	76.3	0	unclassified	unclassified
v0086	34238	60	12	0	Complete	100	0	unclassified	unclassified
v0087	25972	39	9	0	Medium-quality	58.76	0	unclassified	unclassified
v0088	23737	35	12	0	Medium-quality	65.69	0	Siphoviridae	prokaryote
v0089	6242	6	3	0	Complete	100	0	Microviridae	prokaryote
v008a	6156	7	3	0	Complete	100	0	unclassified	unclassified
v008b	94548	96	17	0	High-quality	92.76	0	Podoviridae_crAss-like	prokaryote
v008c	105566	133	18	2	High-quality	100	0	Gratiaviridae	prokaryote
v008d	40601	44	9	1	High-quality	92.06	0	unclassified	unclassified
v008e	6409	7	3	0	Complete	100	0	Microviridae	prokaryote
v008f	5006	7	6	0	High-quality	98.96	0	Microviridae	prokaryote
v0090	19220	23	7	0	Complete	100	0	Podoviridae	prokaryote
v0091	46451	73	17	0	Complete	100	0	unclassified	unclassified
v0092	86610	129	19	2	Complete	100	0	Quimbyviridae	prokaryote
v0093	12730	17	8	0	Medium-quality	67.99	0	Salasmaviridae	prokaryote
v0094	19035	21	8	0	Medium-quality	56.48	0	unclassified	unclassified
v0095	16683	21	8	0	High-quality	90.35	0	unclassified	unclassified
v0096	31098	52	10	1	Medium-quality	69.83	0	unclassified	unclassified
v0097	48901	67	7	2	Medium-quality	84.86	0	unclassified	unclassified
v0098	33831	68	10	1	Complete	100	0	unclassified	unclassified
v0099	4199	6	2	0	Medium-quality	71.91	0	unclassified	unclassified
v009a	35332	39	11	0	Medium-quality	71.31	0	Siphoviridae	prokaryote
v009b	5597	10	5	0	Complete	100	0	Microviridae	prokaryote

v009c	16737	21	7	0	Medium-quality	53.32	0	unclassified	unclassified
v009d	6055	9	6	0	High-quality	100	0	Microviridae	prokaryote
v009e	37157	72	20	1	High-quality	94.02	0	unclassified	unclassified
v009f	3312	5	3	0	Medium-quality	63.23	0	Microviridae	prokaryote
v00a0	20748	27	13	1	Medium-quality	52.31	0	Siphoviridae	prokaryote
v00a1	43256	68	9	2	Medium-quality	55.11	0	unclassified	unclassified
v00a2	6438	7	2	0	Complete	100	0	unclassified	unclassified
v00a3	5418	10	4	0	Complete	100	0	Microviridae	prokaryote
v00a4	77919	125	15	0	Medium-quality	59.76	0	unclassified	unclassified
v00a5	15022	21	9	0	Medium-quality	71.64	0	unclassified	unclassified
v00a6	6199	10	4	0	High-quality	100	0	unclassified	unclassified
v00a7	76947	116	18	2	Complete	100	0	unclassified	unclassified
v00a8	31430	50	18	0	Medium-quality	69.05	0	unclassified	unclassified
v00a9	7126	9	3	0	Complete	100	0	unclassified	unclassified
v00aa	87752	144	23	2	Complete	100	0	unclassified	unclassified
v00ab	41205	55	10	1	Complete	100	0	unclassified	unclassified
v00ac	5663	10	5	0	Complete	100	0	Microviridae	prokaryote
v00ad	5668	7	2	0	Complete	100	0	unclassified	unclassified
v00ae	37647	53	40	0	Complete	100	0	Siphoviridae	prokaryote
v00af	38928	54	15	0	High-quality	100	0	Siphoviridae	prokaryote
v00b0	67024	97	20	1	High-quality	94.48	0	unclassified	unclassified
v00b1	14925	26	7	0	Medium-quality	76.64	0	unclassified	unclassified
v00b2	4623	8	2	0	High-quality	100	0	Microviridae	prokaryote
v00b3	81129	99	24	1	Complete	100	0	unclassified	unclassified
v00b4	58041	79	12	0	Complete	100	0	unclassified	unclassified
v00b5	42411	61	10	1	Medium-quality	64.41	0	unclassified	unclassified
v00b6	8756	10	3	0	Medium-quality	63.04	0	unclassified	unclassified
v00b7	19708	27	6	1	Medium-quality	53.72	0	unclassified	unclassified
v00b8	139459	211	37	2	Medium-quality	86.03	0	unclassified	unclassified
v00b9	46007	74	16	1	High-quality	100	0	unclassified	unclassified
v00ba	104894	155	30	1	Complete	100	0	Podoviridae_crAss-like	prokaryote
v00bb	37964	58	20	0	High-quality	99.84	0	Siphoviridae	prokaryote
v00bc	33391	51	13	0	Complete	100	0	unclassified	unclassified
v00bd	8724	18	3	0	Medium-quality	52.55	0	unclassified	unclassified
v00be	24681	31	11	0	Medium-quality	54.76	0	unclassified	unclassified
v00bf	45211	69	15	1	Complete	100	0	unclassified	unclassified
v00c0	2601	4	3	0	Medium-quality	50.59	0	Microviridae	prokaryote
v00c1	54261	73	11	2	Medium-quality	67.87	0	unclassified	unclassified
v00c2	6781	8	3	0	Complete	100	0	unclassified	unclassified
v00c3	6438	7	2	0	Complete	100	0	unclassified	unclassified

v00c4	24598	39	13	1	Medium-quality	82.88	0	Siphoviridae	prokaryote
v00c5	33778	40	8	1	High-quality	100	0	unclassified	unclassified
v00c6	38276	52	15	0	Complete	100	0	unclassified	unclassified
v00c7	29848	34	9	0	Medium-quality	51.81	0	unclassified	unclassified
v00c8	3952	8	0	0	Medium-quality	77.11	0	Inoviridae	prokaryote
v00c9	77275	115	23	1	Medium-quality	81.32	0	unclassified	unclassified
v00ca	42187	58	12	1	High-quality	94.19	0	unclassified	unclassified
v00cb	19571	29	12	0	Medium-quality	53.29	0	Siphoviridae	prokaryote
v00cc	33615	41	12	0	Medium-quality	77.06	0	unclassified	unclassified
v00cd	5237	7	3	0	Complete	100	0	unclassified	unclassified
v00ce	110659	163	30	3	High-quality	100	0	unclassified	unclassified
v00cf	17694	24	8	0	Complete	100	0	Podoviridae	prokaryote
v00d0	14136	17	4	0	Medium-quality	50.2	0	unclassified	unclassified
v00d1	37020	45	10	1	Medium-quality	86.43	0	unclassified	unclassified
v00d2	100695	151	14	2	Complete	100	0	Podoviridae_crAss-like	prokaryote
v00d3	15580	16	7	0	Medium-quality	67.05	0	Salasmaviridae	prokaryote
v00d4	7289	10	3	0	Complete	100	0	unclassified	unclassified
v00d5	42024	71	16	0	Complete	100	0	unclassified	unclassified
v00d6	24477	36	8	0	Medium-quality	60.69	0	Siphoviridae	prokaryote
v00d7	17385	28	6	0	High-quality	91.09	0	unclassified	unclassified
v00d8	12971	21	4	0	Complete	100	0	unclassified	unclassified
v00d9	43973	82	15	2	Complete	100	0	unclassified	unclassified
v00da	17548	24	9	0	Complete	100	0	unclassified	unclassified
v00db	5103	9	7	0	Complete	100	0	Microviridae	prokaryote
v00dc	6293	6	3	0	Complete	100	0	unclassified	unclassified
v00dd	31629	56	20	0	Complete	100	0	Siphoviridae	prokaryote
v00de	5243	8	6	0	Complete	100	0	Microviridae	prokaryote
v00df	33593	62	16	1	Complete	100	0	unclassified	unclassified
v00e0	12034	14	0	0	Medium-quality	73.97	0	unclassified	unclassified
v00e1	60812	94	32	1	High-quality	100	0	Siphoviridae	prokaryote
v00e2	73684	97	24	1	High-quality	100	0	unclassified	unclassified
v00e3	13105	20	5	0	Complete	100	0	unclassified	unclassified
v00e4	13647	19	4	0	High-quality	100	0	unclassified	unclassified
v00e5	37656	62	28	0	High-quality	93.63	0	Siphoviridae	prokaryote
v00e6	15643	19	7	0	Complete	100	0	unclassified	unclassified
v00e7	5396	9	7	0	Complete	100	0	Microviridae	prokaryote
v00e8	32243	52	23	2	Complete	100	0	Siphoviridae	prokaryote
v00e9	5362	9	7	0	Complete	100	0	Microviridae	prokaryote
v00ea	7339	8	3	0	High-quality	100	0	unclassified	unclassified
v00eb	5027	8	7	0	Complete	100	0	Microviridae	prokaryote

v00ec	18131	28	8	0	High-quality	93.78	0	unclassified	unclassified
v00ed	5019	6	3	0	Complete	100	0	Microviridae	prokaryote
v00ee	12273	20	6	0	Medium-quality	65.58	0	unclassified	unclassified
v00ef	5287	8	6	0	Complete	100	0	Microviridae	prokaryote
v00f0	33928	60	19	0	Complete	100	0	unclassified	unclassified
v00f1	31574	53	9	1	High-quality	95.11	0	unclassified	unclassified
v00f2	19528	36	15	1	Medium-quality	58.58	0	Siphoviridae	prokaryote
v00f3	17569	19	9	0	Medium-quality	82.31	0	unclassified	unclassified
v00f4	47180	76	5	0	Medium-quality	67.25	0	Quimbyviridae	prokaryote
v00f5	104691	165	30	1	Complete	100	0	Podoviridae_crAss-like	prokaryote
v00f6	2891	3	3	0	Medium-quality	50.65	0	Microviridae	prokaryote
v00f7	6497	12	0	0	High-quality	100	0	Inoviridae	prokaryote
v00f8	12355	12	1	0	Medium-quality	77.54	0	unclassified	unclassified
v00f9	5923	7	2	0	Complete	100	0	unclassified	unclassified
v00fa	37973	37	6	1	Medium-quality	78.87	0	unclassified	unclassified
v00fb	16597	21	7	0	Medium-quality	77.34	0	unclassified	unclassified
v00fc	5244	7	0	0	Complete	100	0	Inoviridae	prokaryote
v00fd	5549	10	5	0	Complete	100	0	Microviridae	prokaryote
v00fe	6479	9	3	0	Complete	100	0	Microviridae	prokaryote
v00ff	5791	9	2	0	High-quality	96.92	0	unclassified	unclassified
v0100	5440	8	7	0	High-quality	100	0	Microviridae	prokaryote
v0101	46931	55	7	2	Medium-quality	83.55	0	unclassified	unclassified
v0102	46458	65	10	1	Medium-quality	65.98	0	unclassified	unclassified
v0103	18150	28	7	0	Complete	100	0	unclassified	unclassified
v0104	5249	9	0	0	Medium-quality	85.63	0	unclassified	unclassified
v0105	5380	9	6	0	Complete	100	0	Microviridae	prokaryote
v0106	19532	23	8	0	Medium-quality	57.95	0	unclassified	unclassified
v0107	5455	9	6	0	Complete	100	0	Microviridae	prokaryote
v0108	5626	9	4	0	Complete	100	0	Microviridae	prokaryote
v0109	16288	25	8	0	Medium-quality	70.99	0	unclassified	unclassified
v010a	6187	7	4	0	Complete	100	0	unclassified	unclassified
v010b	19843	45	4	1	Medium-quality	59.73	0	unclassified	unclassified
v010c	6321	8	2	0	Complete	100	0	unclassified	unclassified
v010d	38499	52	9	1	High-quality	92.3	0	unclassified	unclassified
v010e	12861	18	5	0	Complete	100	0	unclassified	unclassified
v010f	17102	24	6	0	Complete	100	0	unclassified	unclassified
v0110	32900	47	7	0	Complete	100	0	unclassified	unclassified
v0111	60285	86	11	4	Medium-quality	72.53	0	unclassified	unclassified
v0112	59491	91	34	0	Complete	100	0	Siphoviridae	prokaryote
v0113	97573	123	50	0	Medium-quality	60.83	0	unclassified	unclassified

v0114	161050	304	20	3	High-quality	93.21	0	unclassified	unclassified
v0115	5101	8	7	0	Complete	100	0	Microviridae	prokaryote
v0116	31941	58	14	0	High-quality	100	0	unclassified	unclassified
v0117	4650	8	3	0	Complete	100	0	Microviridae	prokaryote
v0118	5814	11	6	0	Complete	100	0	unclassified	unclassified
v0119	2559	6	0	0	Medium-quality	53.83	0	unclassified	unclassified
v011a	88038	128	27	1	High-quality	99.35	0	unclassified	unclassified
v011b	5619	9	5	0	Complete	100	0	Microviridae	prokaryote
v011c	5412	9	6	0	Complete	100	0	Microviridae	prokaryote
v011d	5978	9	4	0	Complete	100	0	Microviridae	prokaryote
v011e	3229	5	4	0	Medium-quality	61.32	0	Microviridae	prokaryote
v011f	5347	8	6	0	Complete	100	0	Microviridae	prokaryote
v0120	13914	19	5	0	High-quality	100	0	Podoviridae	prokaryote
v0121	43759	72	17	1	Complete	100	0	unclassified	unclassified
v0122	5533	7	3	0	High-quality	100	0	unclassified	unclassified
v0123	6696	8	4	0	Complete	100	0	Microviridae	prokaryote
v0124	5674	8	6	0	Complete	100	0	Microviridae	prokaryote
v0125	11232	14	5	0	Complete	100	0	Podoviridae	prokaryote
v0126	33750	55	16	1	High-quality	98.27	0	Siphoviridae	prokaryote
v0127	3425	4	2	0	Medium-quality	51.93	0	unclassified	unclassified
v0128	13135	24	5	0	High-quality	91.27	0	unclassified	unclassified
v0129	97383	180	14	2	High-quality	99.37	0	Podoviridae_crAss-like	prokaryote
v012a	30498	47	10	0	Medium-quality	79.88	0	unclassified	unclassified
v012b	4833	5	4	0	Complete	100	0	Microviridae	prokaryote
v012c	45242	84	17	0	Complete	100	0	unclassified	unclassified
v012d	102321	153	16	1	Complete	100	0	Podoviridae_crAss-like	prokaryote
v012e	31493	37	5	1	Medium-quality	75.96	0	unclassified	unclassified
v012f	40259	41	1	15	Medium-quality	62.87	0	unclassified	unclassified
v0130	6173	6	3	0	Complete	100	0	unclassified	unclassified
v0131	5042	8	6	0	Complete	100	0	Microviridae	prokaryote
v0132	108579	168	16	3	Medium-quality	85.25	0	unclassified	unclassified
v0133	48770	39	2	3	Medium-quality	50.82	0	Podoviridae_crAss-like	prokaryote
v0134	53426	73	33	0	Medium-quality	88.23	0	Siphoviridae	prokaryote
v0135	63294	87	14	1	Medium-quality	69.07	0	unclassified	unclassified
v0136	5226	8	6	0	High-quality	100	0	Microviridae	prokaryote
v0137	161435	205	20	1	Complete	100	0	unclassified	unclassified
v0138	16426	19	8	0	Medium-quality	68.17	0	unclassified	unclassified
v0139	170857	302	29	0	Complete	100	0	unclassified	unclassified
v013a	3189	8	0	0	Medium-quality	55.14	0	Inoviridae	prokaryote
v013b	20778	34	10	0	Medium-quality	64.13	0	unclassified	unclassified

v013c	108896	197	28	1	Medium-quality	70.2	0	unclassified	unclassified
v013d	8554	14	10	0	High-quality	100	0	Microviridae	prokaryote
v013e	42079	70	13	0	High-quality	100	0	unclassified	unclassified
v013f	4875	9	6	0	Complete	100	0	Microviridae	prokaryote
v0140	139902	187	41	0	Medium-quality	83.21	0	unclassified	unclassified
v0141	54654	90	17	0	High-quality	99.05	0	unclassified	unclassified
v0142	20849	27	10	0	Medium-quality	57.29	0	unclassified	unclassified
v0143	37624	61	11	0	Medium-quality	73.15	0	unclassified	unclassified
v0144	5841	10	4	0	Complete	100	0	unclassified	unclassified
v0145	27440	46	6	0	Medium-quality	73.95	0	unclassified	unclassified
v0146	5439	7	5	0	Complete	100	0	Microviridae	prokaryote
v0147	5081	8	7	0	Complete	100	0	Microviridae	prokaryote
v0148	15965	22	7	0	Medium-quality	63.65	0	Podoviridae	prokaryote
v0149	4696	7	3	0	Complete	100	0	Microviridae	prokaryote
v014a	4901	7	7	0	Complete	100	0	Microviridae	prokaryote
v014b	49441	63	14	1	Medium-quality	59.91	0	Quimbyviridae	prokaryote
v014c	6419	7	2	0	Complete	100	0	unclassified	unclassified
v014d	87834	133	15	4	Complete	100	0	Quimbyviridae	prokaryote
v014e	6443	9	4	0	Complete	100	0	unclassified	unclassified
v014f	4896	8	5	0	High-quality	96.46	0	Microviridae	prokaryote
v0150	4345	4	2	0	Medium-quality	68.85	0	unclassified	unclassified
v0151	81841	116	20	1	Complete	100	0	unclassified	unclassified
v0152	5084	6	6	0	Complete	100	0	Microviridae	prokaryote
v0153	109761	175	26	4	Medium-quality	65.12	0	unclassified	unclassified
v0154	66153	70	1	26	High-quality	100	0	unclassified	unclassified
v0155	5254	10	6	0	High-quality	100	0	Microviridae	prokaryote
v0156	62237	90	17	1	Complete	100	0	unclassified	unclassified
v0157	130279	158	36	3	Complete	100	0	unclassified	unclassified
v0158	2570	2	1	0	Medium-quality	61.76	0	unclassified	unclassified
v0159	4941	6	3	0	Medium-quality	70.44	0	unclassified	unclassified
v015a	4888	7	5	0	Complete	100	0	Microviridae	prokaryote
v015b	62052	102	32	0	High-quality	100	0	Siphoviridae	prokaryote
v015c	138447	186	28	3	Medium-quality	89.58	0	unclassified	unclassified
v015d	24424	31	17	0	Medium-quality	58.79	0	Siphoviridae	prokaryote
v015e	128194	224	27	4	Medium-quality	69.72	0	unclassified	unclassified
v015f	57598	43	3	0	Medium-quality	60.29	0	Podoviridae_crAss-like	prokaryote
v0160	4957	6	3	0	Complete	100	0	Microviridae	prokaryote
v0161	4571	9	3	0	Complete	100	0	Microviridae	prokaryote
v0162	5251	9	5	0	Complete	100	0	Microviridae	prokaryote
v0163	5054	5	2	0	Complete	100	0	Microviridae	prokaryote

v0164	50931	75	34	0	Complete	100	0	Myoviridae	prokaryote
v0165	5163	8	7	0	Complete	100	0	Microviridae	prokaryote
v0166	5995	9	2	0	Complete	100	0	unclassified	unclassified
v0167	21563	29	14	0	Medium-quality	65.61	0	Myoviridae	prokaryote
v0168	70905	106	29	2	High-quality	91.11	0	unclassified	unclassified
v0169	3440	3	1	0	Medium-quality	52.29	0	Microviridae	prokaryote
v016a	37351	44	7	0	High-quality	94.79	0	unclassified	unclassified
v016b	43261	64	17	1	Medium-quality	87	0	unclassified	unclassified
v016c	5831	8	3	0	Complete	100	0	unclassified	unclassified
v016d	47086	82	19	0	High-quality	100	0	unclassified	unclassified
v016e	39673	61	15	0	Complete	100	0	Siphoviridae	prokaryote
v016f	18381	22	8	0	Medium-quality	59.75	0	unclassified	unclassified
v0170	4456	8	7	0	High-quality	90.67	0	Microviridae	prokaryote
v0171	12728	15	5	0	High-quality	100	0	Podoviridae	prokaryote
v0172	31144	60	13	0	Medium-quality	58.17	0	unclassified	unclassified
v0173	6609	8	3	0	Complete	100	0	Microviridae	prokaryote
v0174	21985	31	10	1	Medium-quality	57.01	0	unclassified	unclassified
v0175	6372	6	3	0	Complete	100	0	Microviridae	prokaryote
v0176	17049	30	14	0	Medium-quality	67.32	0	Siphoviridae	prokaryote
v0177	6065	9	3	0	Complete	100	0	unclassified	unclassified
v0178	4288	9	0	0	Medium-quality	50.36	0	Myoviridae	prokaryote
v0179	38173	65	18	2	High-quality	100	0	unclassified	unclassified
v017a	6102	9	3	0	Complete	100	0	unclassified	unclassified
v017b	16727	21	8	0	Medium-quality	73.54	0	unclassified	unclassified
v017c	5987	8	2	0	Complete	100	0	unclassified	unclassified
v017d	16026	19	6	0	Medium-quality	73.09	0	Salasmaviridae	prokaryote
v017e	36098	52	7	0	Medium-quality	86.14	0	unclassified	unclassified
v017f	27928	41	17	2	Medium-quality	83.05	0	Siphoviridae	prokaryote
v0180	44382	81	17	1	Complete	100	0	unclassified	unclassified
v0181	20196	30	8	0	High-quality	100	0	unclassified	unclassified
v0182	19094	26	11	0	Medium-quality	57.05	0	unclassified	unclassified
v0183	38831	53	15	1	Medium-quality	78.59	0	unclassified	unclassified
v0184	14224	19	6	0	High-quality	100	0	unclassified	unclassified
v0185	12406	17	4	0	Complete	100	0	unclassified	unclassified
v0186	16801	22	7	0	Medium-quality	51.68	0	unclassified	unclassified
v0187	64071	92	16	0	Complete	100	0	unclassified	unclassified
v0188	3867	8	0	0	Medium-quality	75.4	0	Inoviridae	prokaryote
v0189	7137	7	3	0	Complete	100	0	unclassified	unclassified
v018a	5134	9	0	0	Complete	100	0	Inoviridae	prokaryote
v018b	21383	25	6	1	Medium-quality	62.08	0	unclassified	unclassified

v018c	6338	7	2	0	Complete	100	0	unclassified	unclassified
v018d	7334	13	2	0	Complete	100	0	Inoviridae	prokaryote
v018e	13640	15	4	0	High-quality	100	0	Podoviridae	prokaryote
v018f	28280	52	11	0	Medium-quality	60.88	0	unclassified	unclassified
v0190	41128	72	18	0	Complete	100	0	unclassified	unclassified
v0191	78736	107	18	1	Medium-quality	78.18	0	unclassified	unclassified
v0192	5069	9	7	0	Complete	100	0	Microviridae	prokaryote
v0193	5905	9	4	0	Complete	100	0	Microviridae	prokaryote
v0194	5327	8	6	0	Complete	100	0	Microviridae	prokaryote
v0195	40553	73	20	0	Complete	100	0	unclassified	unclassified
v0196	54536	93	24	1	High-quality	100	0	unclassified	unclassified
v0197	6050	9	1	0	Complete	100	0	unclassified	unclassified
v0198	25207	43	15	0	Medium-quality	55.94	0	unclassified	unclassified
v0199	49882	47	9	1	Medium-quality	57.2	0	Quimbyviridae	prokaryote
v019a	20728	27	10	0	Medium-quality	71.84	0	unclassified	unclassified
v019b	100901	118	8	2	High-quality	96.75	0	unclassified	unclassified
v019c	5148	9	6	0	Complete	100	0	Microviridae	prokaryote
v019d	15016	19	7	1	Complete	100	0	unclassified	unclassified
v019e	5007	8	5	0	Complete	100	0	Microviridae	prokaryote
v019f	15319	18	7	0	Medium-quality	78.88	0	Salasmaviridae	prokaryote
v01a0	6463	8	3	0	High-quality	100	0	Microviridae	prokaryote
v01a1	6242	10	3	0	Complete	100	0	unclassified	unclassified
v01a2	33896	56	43	0	Medium-quality	86.3	0	Podoviridae	prokaryote
v01a3	6473	10	0	0	High-quality	100	0	Inoviridae	prokaryote
v01a4	5793	8	3	0	Complete	100	0	unclassified	unclassified
v01a5	5947	9	4	0	Complete	100	0	Microviridae	prokaryote
v01a6	15862	15	6	0	Medium-quality	53.39	0	unclassified	unclassified
v01a7	63894	103	15	0	Complete	100	0	Quimbyviridae	prokaryote
v01a8	6565	8	3	0	Complete	100	0	Microviridae	prokaryote
v01a9	6497	8	4	0	Complete	100	0	unclassified	unclassified
v01aa	6581	9	4	0	Complete	100	0	unclassified	unclassified
v01ab	40887	66	24	1	High-quality	100	0	Siphoviridae	prokaryote
v01ac	47013	56	41	0	Complete	100	0	Autographiviridae	prokaryote
v01ad	42116	62	8	0	Complete	100	0	unclassified	unclassified
v01ae	5983	8	2	0	Complete	100	0	unclassified	unclassified
v01af	24364	36	21	0	Medium-quality	57.37	0	Myoviridae	prokaryote
v01b0	37793	59	22	1	Medium-quality	66.39	0	Siphoviridae	prokaryote
v01b1	4989	8	6	0	Complete	100	0	Microviridae	prokaryote
v01b2	2573	3	3	0	Medium-quality	52.61	0	Microviridae	prokaryote
v01b3	3275	4	4	0	Medium-quality	64.5	0	Microviridae	prokaryote

v01b4	4733	8	3	0	High-quality	97.18	0	Microviridae	prokaryote
v01b5	16774	18	6	0	Medium-quality	51.49	0	unclassified	unclassified
v01b6	4502	7	2	0	High-quality	95.48	0	Microviridae	prokaryote
v01b7	21573	27	6	0	High-quality	100	0	unclassified	unclassified
v01b8	2638	3	1	0	Medium-quality	63.39	0	Circoviridae	eukaryote
v01b9	2467	3	2	0	High-quality	95.1	0	Smacoviridae	eukaryote
v01ba	3630	5	4	0	Medium-quality	74.33	0	Microviridae	prokaryote
v01bb	50321	70	8	1	Medium-quality	69.13	0	unclassified	unclassified
v01bc	11278	12	4	0	High-quality	96.1	0	Podoviridae	prokaryote
v01bd	13683	14	4	0	High-quality	100	0	Podoviridae	prokaryote
v01be	4648	5	4	0	Medium-quality	80.09	0	unclassified	unclassified
v01bf	9228	7	3	0	Complete	100	0	unclassified	unclassified
v01c0	4712	6	2	0	High-quality	100	0	Microviridae	prokaryote
v01c1	30730	57	13	1	Medium-quality	72.03	0	unclassified	unclassified
v01c2	9134	11	4	0	Medium-quality	77.62	0	Podoviridae	prokaryote
v01c3	11163	15	3	0	Complete	100	0	Podoviridae	prokaryote
v01c4	30403	37	18	0	Medium-quality	50.43	0	Siphoviridae	prokaryote
v01c5	16230	21	8	0	Medium-quality	81.6	0	unclassified	unclassified
v01c6	19904	33	14	1	Medium-quality	51.03	0	Siphoviridae	prokaryote
v01c7	12829	16	3	0	Complete	100	0	Podoviridae	prokaryote
v01c8	22374	52	12	0	Medium-quality	70.38	0	unclassified	unclassified
v01c9	16991	23	3	0	Medium-quality	55.51	0	unclassified	unclassified
v01ca	7051	9	4	0	High-quality	100	0	unclassified	unclassified
v01cb	5571	8	3	0	High-quality	100	0	unclassified	unclassified
v01cc	4129	3	2	0	Medium-quality	77.66	0	Microviridae	prokaryote
v01cd	6284	6	4	0	High-quality	100	0	unclassified	unclassified
v01ce	2622	5	1	0	Medium-quality	63.01	0	Circoviridae	eukaryote
v01cf	5102	6	5	0	Complete	100	0	Microviridae	prokaryote
v01d0	14357	17	5	0	Medium-quality	50.11	0	unclassified	unclassified
v01d1	6002	4	3	0	High-quality	94.59	0	Microviridae	prokaryote
v01d2	3751	6	6	0	Medium-quality	74.82	0	Microviridae	prokaryote
v01d3	2628	4	4	0	Medium-quality	51.67	0	Microviridae	prokaryote
v01d4	5439	7	3	0	Complete	100	0	unclassified	unclassified
v01d5	3443	8	2	0	Medium-quality	56.01	0	unclassified	unclassified
v01d6	23763	31	17	0	Medium-quality	58.51	0	unclassified	unclassified
v01d7	31549	41	22	1	Medium-quality	50.97	0	Siphoviridae	prokaryote
v01d8	12092	14	4	0	Complete	100	0	unclassified	unclassified
v01d9	9372	11	7	0	Medium-quality	50.86	0	Salasmaviridae	prokaryote
v01da	3025	4	1	0	Medium-quality	72.69	0	unclassified	unclassified
v01db	16230	22	7	0	Medium-quality	68.77	0	unclassified	unclassified

v01dc	19233	29	7	1	Medium-quality	59.86	0	unclassified	unclassified
v01dd	32587	45	9	0	Complete	100	0	unclassified	unclassified
v01de	6762	8	3	0	High-quality	100	0	unclassified	unclassified
v01df	6081	7	4	0	Complete	100	0	unclassified	unclassified
v01e0	5582	10	6	0	Complete	100	0	Microviridae	prokaryote
v01e1	6097	6	3	0	Complete	100	0	unclassified	unclassified
v01e2	6571	9	3	0	Complete	100	0	Microviridae	prokaryote
v01e3	6594	8	3	0	Complete	100	0	unclassified	unclassified
v01e4	3551	7	1	0	Medium-quality	59.33	0	unclassified	unclassified
v01e5	7773	9	4	0	High-quality	100	0	unclassified	unclassified
v01e6	6730	8	3	0	Complete	100	0	unclassified	unclassified
v01e7	3389	3	2	0	Medium-quality	53.45	0	unclassified	unclassified
v01e8	4035	10	1	0	Medium-quality	67.42	0	unclassified	unclassified
v01e9	6077	8	2	0	Complete	100	0	unclassified	unclassified
v01ea	34885	59	18	0	Complete	100	0	unclassified	unclassified
v01eb	35381	46	24	0	Medium-quality	58.48	0	Siphoviridae	prokaryote
v01ec	4693	7	2	0	Medium-quality	76.84	0	unclassified	unclassified
v01ed	39424	69	16	0	Complete	100	0	unclassified	unclassified
v01ee	41816	80	16	0	Complete	100	0	unclassified	unclassified
v01ef	5602	9	7	0	Complete	100	0	Microviridae	prokaryote
v01f0	6306	10	4	0	Complete	100	0	unclassified	unclassified
v01f1	6089	9	2	0	Complete	100	0	unclassified	unclassified
v01f2	4808	8	5	0	Complete	100	0	Microviridae	prokaryote
v01f3	43582	61	8	1	Complete	100	0	unclassified	unclassified
v01f4	15102	17	1	0	Complete	100	0	unclassified	unclassified
v01f5	22800	30	9	0	Medium-quality	63.91	0	unclassified	unclassified
v01f6	52299	83	23	1	Complete	100	0	unclassified	unclassified
v01f7	4972	9	6	0	Complete	100	0	Microviridae	prokaryote
v01f8	3768	7	2	0	Medium-quality	58.88	0	unclassified	unclassified
v01f9	6080	10	3	0	Complete	100	0	unclassified	unclassified
v01fa	3398	4	2	0	Medium-quality	53.81	0	unclassified	unclassified
v01fb	6100	10	5	0	Complete	100	0	Microviridae	prokaryote
v01fc	5928	6	2	0	Complete	100	0	unclassified	unclassified
v01fd	6338	8	4	0	Complete	100	0	unclassified	unclassified
v01fe	32196	32	9	1	Medium-quality	77.2	0	unclassified	unclassified
v01ff	6498	9	4	0	Complete	100	0	Microviridae	prokaryote
v0200	5906	7	2	0	Complete	100	0	unclassified	unclassified
v0201	15672	21	7	0	Medium-quality	67.06	0	unclassified	unclassified
v0202	6707	9	4	0	Complete	100	0	Microviridae	prokaryote
v0203	6257	9	3	0	Complete	100	0	unclassified	unclassified

v0204	4645	6	2	0	Complete	100	0	Microviridae	prokaryote
v0205	5271	8	7	0	Complete	100	0	Microviridae	prokaryote
v0206	5825	6	3	0	High-quality	95.38	0	unclassified	unclassified
v0207	4397	5	2	0	Medium-quality	73.68	0	unclassified	unclassified
v0208	56731	100	20	1	High-quality	96.25	0	unclassified	unclassified
v0209	6411	10	3	0	High-quality	100	0	unclassified	unclassified
v020a	10941	16	12	0	High-quality	100	0	Microviridae	prokaryote
v020b	6134	9	3	0	Complete	100	0	unclassified	unclassified
v020c	5693	6	2	0	Complete	100	0	unclassified	unclassified
v020d	6564	7	3	0	High-quality	100	0	unclassified	unclassified
v020e	42958	76	24	1	Complete	100	0	unclassified	unclassified
v020f	13368	20	5	0	High-quality	96.25	0	unclassified	unclassified
v0210	6340	6	3	0	Complete	100	0	unclassified	unclassified
v0211	6006	9	3	0	Complete	100	0	unclassified	unclassified
v0212	5528	8	5	0	Complete	100	0	Microviridae	prokaryote
v0213	5200	9	2	0	Medium-quality	86.93	0	unclassified	unclassified
v0214	6132	9	2	0	High-quality	100	0	unclassified	unclassified
v0215	6565	8	3	0	Complete	100	0	unclassified	unclassified
v0216	46472	80	48	0	Complete	100	0	Siphoviridae	prokaryote
v0217	34133	50	15	0	Complete	100	0	unclassified	unclassified
v0218	5935	8	4	0	Complete	100	0	Microviridae	prokaryote
v0219	7394	10	3	0	High-quality	100	0	unclassified	unclassified
v021a	6177	9	4	0	Medium-quality	88.94	0	unclassified	unclassified
v021b	3770	8	3	0	Medium-quality	64.71	0	unclassified	unclassified
v021c	44946	74	45	0	High-quality	95.66	0	Siphoviridae	prokaryote
v021d	6500	10	3	0	Complete	100	0	Microviridae	prokaryote
v021e	6281	7	3	0	High-quality	100	0	unclassified	unclassified
v021f	6677	8	4	0	Complete	100	0	unclassified	unclassified
v0220	6382	6	3	0	Complete	100	0	Microviridae	prokaryote
v0221	11693	15	5	0	Complete	100	0	Podoviridae	prokaryote
v0222	5655	7	5	0	Complete	100	0	Microviridae	prokaryote
v0223	5074	5	2	0	Medium-quality	89.99	0	unclassified	unclassified
v0224	104897	157	30	1	Complete	100	0	Podoviridae_crAss-like	prokaryote
v0225	26654	44	4	1	Medium-quality	60.46	0	unclassified	unclassified
v0226	44521	57	49	0	Complete	100	0	Autographiviridae	prokaryote
v0227	103301	110	5	32	High-quality	93.81	0	unclassified	unclassified
v0228	43503	67	47	0	High-quality	100	0	Podoviridae	prokaryote
v0229	32052	38	15	0	Medium-quality	64.45	0	Siphoviridae	prokaryote
v022a	6326	9	4	0	High-quality	100	0	unclassified	unclassified
v022b	5413	9	4	0	Complete	100	0	Microviridae	prokaryote

v022c	51300	72	11	0	Medium-quality	88.89	0	unclassified	unclassified
v022d	39745	64	11	1	High-quality	97.72	0	unclassified	unclassified
v022e	6011	10	4	0	Complete	100	0	Microviridae	prokaryote
v022f	4952	7	6	0	Complete	100	0	Microviridae	prokaryote
v0230	12458	19	4	0	Complete	100	0	unclassified	unclassified
v0231	4717	9	0	0	Complete	100	0	Inoviridae	prokaryote
v0232	8034	9	4	0	High-quality	100	0	unclassified	unclassified
v0233	33476	60	12	1	Medium-quality	79.28	0	unclassified	unclassified
v0234	6927	8	3	0	Complete	100	0	unclassified	unclassified
v0235	4603	6	2	0	Medium-quality	78.25	0	unclassified	unclassified
v0236	5027	9	7	0	Complete	100	0	Microviridae	prokaryote
v0237	6604	7	3	0	Complete	100	0	unclassified	unclassified
v0238	6302	6	3	0	Complete	100	0	unclassified	unclassified
v0239	5682	9	4	0	Complete	100	0	Microviridae	prokaryote
v023a	3218	6	3	0	Medium-quality	56.96	0	unclassified	unclassified
v023b	19332	26	7	1	Medium-quality	60.17	0	unclassified	unclassified
v023c	15500	21	7	0	Medium-quality	84.15	0	unclassified	unclassified
v023d	23648	45	14	0	Medium-quality	52.53	0	unclassified	unclassified
v023e	13880	18	5	0	High-quality	100	0	unclassified	unclassified
v023f	6503	6	3	0	High-quality	100	0	unclassified	unclassified
v0240	6559	9	4	0	Complete	100	0	Microviridae	prokaryote
v0241	6186	7	2	0	Complete	100	0	unclassified	unclassified
v0242	17808	22	7	0	High-quality	100	0	unclassified	unclassified
v0243	9271	12	5	0	High-quality	100	0	unclassified	unclassified
v0244	6393	11	3	0	High-quality	100	0	unclassified	unclassified
v0245	5668	8	2	0	Complete	100	0	unclassified	unclassified
v0246	5058	8	7	0	Complete	100	0	Microviridae	prokaryote
v0247	39248	38	1	19	Medium-quality	73.16	0	unclassified	unclassified
v0248	5525	7	3	0	High-quality	93.25	0	unclassified	unclassified
v0249	15267	22	8	0	Medium-quality	78.96	0	unclassified	unclassified
v024a	5529	7	3	0	Complete	100	0	unclassified	unclassified
v024b	38568	56	10	1	Medium-quality	66.96	0	unclassified	unclassified
v024c	15730	21	8	0	Medium-quality	87.08	0	unclassified	unclassified
v024d	6069	9	3	0	Complete	100	0	unclassified	unclassified
v024e	6095	7	4	0	Complete	100	0	unclassified	unclassified
v024f	6508	8	4	0	Complete	100	0	Microviridae	prokaryote
v0250	6340	7	4	0	Complete	100	0	unclassified	unclassified
v0251	5662	10	6	0	Complete	100	0	Microviridae	prokaryote
v0252	18405	25	8	0	High-quality	100	0	Podoviridae	prokaryote
v0253	6797	9	3	0	Complete	100	0	unclassified	unclassified

v0254	6075	8	2	0	High-quality	100	0	unclassified	unclassified
v0255	18414	25	6	0	Complete	100	0	Podoviridae	prokaryote
v0256	16370	22	6	0	High-quality	100	0	unclassified	unclassified
v0257	3179	4	2	0	Medium-quality	54.11	0	unclassified	unclassified
v0258	5896	8	3	0	Complete	100	0	unclassified	unclassified
v0259	5487	7	3	0	High-quality	99.15	0	unclassified	unclassified
v025a	5774	7	3	0	High-quality	91.39	0	unclassified	unclassified
v025b	4469	7	2	0	High-quality	99.85	0	Microviridae	prokaryote
v025c	5733	9	3	0	High-quality	94.85	0	unclassified	unclassified
v025d	5755	8	3	0	Complete	100	0	unclassified	unclassified
v025e	4340	8	2	0	Medium-quality	72.51	0	unclassified	unclassified
v025f	43067	58	4	1	Complete	100	0	unclassified	unclassified
v0260	5827	11	4	0	Complete	100	0	unclassified	unclassified
v0261	15917	27	9	0	Medium-quality	70.36	0	unclassified	unclassified
v0262	5670	9	4	0	Complete	100	0	Microviridae	prokaryote
v0263	6464	10	3	0	Complete	100	0	unclassified	unclassified
v0264	44882	71	19	0	Complete	100	0	unclassified	unclassified
v0265	5835	10	4	0	High-quality	100	0	Microviridae	prokaryote
v0266	3069	4	1	0	Complete	100	0	unclassified	unclassified
v0267	3288	2	2	0	Medium-quality	52.87	0	unclassified	unclassified
v0268	2924	3	1	0	Medium-quality	55.9	0	Microviridae	prokaryote
v0269	20493	24	8	0	Medium-quality	60.26	0	unclassified	unclassified
v026a	6405	7	4	0	Complete	100	0	unclassified	unclassified
v026b	9781	17	4	0	Medium-quality	50.7	0	Podoviridae	prokaryote
v026c	2955	4	2	0	Medium-quality	59.03	0	Microviridae	prokaryote
v026d	5941	10	4	0	Complete	100	0	Microviridae	prokaryote
v026e	13172	20	5	0	High-quality	94.84	0	unclassified	unclassified
v026f	3445	6	0	0	Medium-quality	67.16	0	Inoviridae	prokaryote
v0270	16030	21	9	0	Medium-quality	69.12	0	unclassified	unclassified
v0271	5377	8	6	0	Complete	100	0	Microviridae	prokaryote
v0272	35769	47	31	0	Complete	100	0	Siphoviridae	prokaryote
v0273	17156	26	6	0	Medium-quality	89.64	0	unclassified	unclassified
v0274	48827	76	23	1	Complete	100	0	unclassified	unclassified
v0275	5308	8	7	0	Complete	100	0	Microviridae	prokaryote
v0276	5092	8	7	0	Complete	100	0	Microviridae	prokaryote
v0277	6398	8	3	0	Complete	100	0	unclassified	unclassified
v0278	6364	9	2	0	Complete	100	0	unclassified	unclassified
v0279	6904	8	3	0	Complete	100	0	unclassified	unclassified
v027a	6037	8	2	0	Complete	100	0	unclassified	unclassified
v027b	5217	8	7	0	Complete	100	0	Microviridae	prokaryote

v027c	12722	18	4	1	High-quality	91.6	0	unclassified	unclassified
v027d	6063	8	2	0	Complete	100	0	unclassified	unclassified
v027e	20376	28	8	0	High-quality	100	0	unclassified	unclassified
v027f	5911	10	2	0	Complete	100	0	unclassified	unclassified
v0280	16803	22	8	0	High-quality	90.1	0	unclassified	unclassified
v0281	5680	11	6	0	Complete	100	0	Microviridae	prokaryote
v0282	5349	6	2	0	Medium-quality	89.51	0	unclassified	unclassified
v0283	15033	20	7	0	High-quality	95.01	0	unclassified	unclassified
v0284	16227	23	7	0	Medium-quality	86.87	0	unclassified	unclassified
v0285	5795	8	2	0	High-quality	98.52	0	unclassified	unclassified
v0286	16918	25	8	0	Medium-quality	89.34	0	unclassified	unclassified
v0287	6658	8	3	0	High-quality	100	0	unclassified	unclassified
v0288	5663	8	4	0	Complete	100	0	unclassified	unclassified
v0289	46869	73	18	0	Complete	100	0	unclassified	unclassified
v028a	5165	9	6	0	Complete	100	0	Microviridae	prokaryote
v028b	3964	4	1	0	Medium-quality	66.35	0	unclassified	unclassified
v028c	6157	8	3	0	High-quality	100	0	unclassified	unclassified
v028d	6553	9	4	0	Complete	100	0	Microviridae	prokaryote
v028e	6149	9	3	0	Complete	100	0	unclassified	unclassified
v028f	39434	63	21	1	Complete	100	0	Siphoviridae	prokaryote
v0290	6144	9	3	0	Complete	100	0	unclassified	unclassified
v0291	6113	7	3	0	High-quality	100	0	unclassified	unclassified
v0292	8095	10	4	0	Medium-quality	58.28	0	unclassified	unclassified
v0293	30082	45	29	0	Medium-quality	61.96	0	Myoviridae	prokaryote
v0294	193933	213	25	5	High-quality	100	0	unclassified	unclassified
v0295	6600	8	3	0	Complete	100	0	unclassified	unclassified
v0296	50414	85	65	0	Complete	100	0	Drexlerviridae	prokaryote
v0297	5024	7	6	0	Complete	100	0	Microviridae	prokaryote
v0298	16532	23	6	0	Medium-quality	70.59	0	unclassified	unclassified
v0299	6118	9	3	0	Complete	100	0	unclassified	unclassified
v029a	6651	8	4	0	Complete	100	0	unclassified	unclassified
v029b	12986	16	6	0	Complete	100	0	unclassified	unclassified
v029c	3183	3	2	0	Medium-quality	52.19	0	unclassified	unclassified
v029d	5811	9	4	0	Complete	100	0	Microviridae	prokaryote
v029e	6146	10	4	0	Complete	100	0	unclassified	unclassified
v029f	6040	10	2	0	High-quality	100	0	unclassified	unclassified
v02a0	6101	8	4	0	Complete	100	0	unclassified	unclassified
v02a1	5870	9	4	0	High-quality	100	0	unclassified	unclassified
v02a2	6980	7	2	0	Complete	100	0	unclassified	unclassified
v02a3	2932	4	3	0	Medium-quality	61.01	0	Microviridae	prokaryote

v02a4	37014	54	16	0	Medium-quality	78.65	0	Myoviridae	prokaryote
v02a5	40641	75	13	0	High-quality	93.22	0	unclassified	unclassified
v02a6	42598	57	10	1	Medium-quality	67.15	0	unclassified	unclassified
v02a7	26790	44	17	0	Medium-quality	80.73	0	Siphoviridae	prokaryote
v02a8	45897	70	24	2	Complete	100	0	Siphoviridae	prokaryote
v02a9	27369	41	15	1	Medium-quality	71.73	0	unclassified	unclassified
v02aa	22304	29	3	0	Medium-quality	53.44	0	unclassified	unclassified
v02ab	56243	74	1	25	Medium-quality	69.61	0	unclassified	unclassified
v02ac	50785	51	4	15	Medium-quality	60.71	0	unclassified	unclassified
v02ad	34846	51	14	0	High-quality	99.24	0	unclassified	unclassified
v02ae	26860	38	20	0	Medium-quality	66.6	0	Siphoviridae	prokaryote
v02af	40114	40	6	4	Medium-quality	65.46	0	unclassified	unclassified
v02b0	44952	82	16	0	Complete	100	0	unclassified	unclassified
v02b1	43398	72	22	2	Medium-quality	87.59	0	Siphoviridae	prokaryote
v02b2	41341	58	15	0	Complete	100	0	unclassified	unclassified
v02b3	52505	54	3	23	High-quality	100	0	unclassified	unclassified
v02b4	41432	63	17	0	Complete	100	0	unclassified	unclassified
v02b5	29608	39	3	1	Medium-quality	86.5	0	unclassified	unclassified
v02b6	48811	66	18	0	Complete	100	0	unclassified	unclassified
v02b7	43171	62	38	0	Complete	100	0	Autographiviridae	prokaryote
v02b8	32618	52	20	0	Complete	100	0	Siphoviridae	prokaryote
v02b9	41493	68	53	0	Complete	100	0	Siphoviridae	prokaryote
v02ba	22074	37	10	0	Medium-quality	58.28	0	unclassified	unclassified
v02bb	41699	59	25	0	High-quality	100	0	Siphoviridae	prokaryote
v02bc	33614	54	18	0	High-quality	98.6	0	Siphoviridae	prokaryote
v02bd	6123	10	0	0	Complete	100	0	Inoviridae	prokaryote
v02be	40233	54	22	2	Complete	100	0	Siphoviridae	prokaryote
v02bf	5175	5	3	0	Complete	100	0	Microviridae	prokaryote
v02c0	26066	50	9	0	Medium-quality	62.54	0	unclassified	unclassified
v02c1	43283	73	16	0	High-quality	100	0	unclassified	unclassified
v02c2	44972	80	15	2	Medium-quality	78.04	0	unclassified	unclassified
v02c3	35149	64	21	0	High-quality	100	0	unclassified	unclassified
v02c4	105949	128	9	2	Complete	100	0	unclassified	unclassified
v02c5	28895	29	8	2	Medium-quality	55.21	0	unclassified	unclassified
v02c6	17694	32	4	0	Medium-quality	50	0	unclassified	unclassified
v02c7	46144	85	21	0	Complete	100	0	unclassified	unclassified
v02c8	24380	33	11	1	Medium-quality	59.55	0	Siphoviridae	prokaryote
v02c9	49864	67	9	2	Medium-quality	66.64	0	unclassified	unclassified
v02ca	7656	15	0	0	Medium-quality	51	0	unclassified	unclassified
v02cb	19880	33	10	0	Medium-quality	61.14	0	Siphoviridae	prokaryote

v02cc	50930	73	33	1	Complete	100	0	unclassified	unclassified
v02cd	13177	17	6	0	Medium-quality	70.67	0	Salasmaviridae	prokaryote
v02ce	18097	18	7	0	Medium-quality	77.18	0	Podoviridae	prokaryote
v02cf	26191	35	14	0	Medium-quality	66.73	0	Siphoviridae	prokaryote
v02d0	3708	10	1	0	Medium-quality	50.51	0	unclassified	unclassified
v02d1	34125	45	21	0	Medium-quality	56.69	0	Siphoviridae	prokaryote
v02d2	45042	81	16	0	Complete	100	0	unclassified	unclassified
v02d3	39546	59	33	0	High-quality	100	0	Siphoviridae	prokaryote
v02d4	41535	61	12	1	Complete	100	0	unclassified	unclassified
v02d5	44011	71	19	0	Complete	100	0	unclassified	unclassified
v02d6	95811	171	16	1	Complete	100	0	Podoviridae_crAss-like	prokaryote
v02d7	7033	9	2	0	Complete	100	0	unclassified	unclassified
v02d8	6618	8	3	0	Complete	100	0	unclassified	unclassified
v02d9	19412	30	8	0	High-quality	91.88	0	unclassified	unclassified
v02da	111375	139	36	5	High-quality	99.92	0	unclassified	unclassified
v02db	47020	81	8	1	Medium-quality	53.46	0	Quimbyviridae	prokaryote
v02dc	49827	48	1	24	Medium-quality	77.81	0	unclassified	unclassified
v02dd	30371	36	19	0	Medium-quality	86.42	0	Siphoviridae	prokaryote
v02de	39608	52	19	1	High-quality	100	0	Siphoviridae	prokaryote
v02df	18518	32	11	0	Medium-quality	52.39	0	Siphoviridae	prokaryote
v02e0	91988	137	11	2	Complete	100	0	Quimbyviridae	prokaryote
v02e1	50623	50	1	8	High-quality	100	0	unclassified	unclassified
v02e2	19785	9	1	4	Medium-quality	57.06	0	unclassified	unclassified
v02e3	45387	78	20	0	Complete	100	0	unclassified	unclassified
v02e4	42192	75	15	0	High-quality	97.43	0	unclassified	unclassified
v02e5	45312	75	19	0	Complete	100	0	unclassified	unclassified
v02e6	34126	33	1	12	Medium-quality	53.29	0	unclassified	unclassified
v02e7	57278	101	22	0	Complete	100	0	unclassified	unclassified
v02e8	25148	38	16	1	Medium-quality	63.81	0	Siphoviridae	prokaryote
v02e9	18892	26	8	0	Complete	100	0	Podoviridae	prokaryote
v02ea	77140	118	19	3	Complete	100	0	unclassified	unclassified
v02eb	5038	6	6	0	Complete	100	0	Microviridae	prokaryote
v02ec	46568	76	17	1	Complete	100	0	unclassified	unclassified
v02ed	5185	8	6	0	Complete	100	0	Microviridae	prokaryote
v02ee	6031	11	1	0	Complete	100	0	Inoviridae	prokaryote
v02ef	59629	102	17	2	Complete	100	0	unclassified	unclassified
v02f0	6109	6	3	0	Complete	100	0	unclassified	unclassified
v02f1	4045	9	0	0	Medium-quality	63.48	0	unclassified	unclassified
v02f2	21350	25	18	0	Medium-quality	53.72	0	Siphoviridae	prokaryote
v02f3	29979	37	15	1	Medium-quality	75.99	0	Siphoviridae	prokaryote

v02f4	2055	3	3	0	Complete	100	0	Circoviridae	eukaryote
v02f5	2112	5	0	0	High-quality	100	0	unclassified	unclassified
v02f6	36595	63	24	0	High-quality	100	0	Siphoviridae	prokaryote
v02f7	33540	34	1	15	Medium-quality	54.29	0	unclassified	unclassified
v02f8	61306	104	19	0	Complete	100	0	unclassified	unclassified
v02f9	3119	6	0	0	High-quality	100	0	unclassified	unclassified
v02fa	18956	30	7	0	Medium-quality	69.48	0	unclassified	unclassified
v02fb	2766	3	3	0	High-quality	98.27	0	Circoviridae	eukaryote
v02fc	2548	4	2	0	Complete	100	0	Circoviridae	eukaryote
v02fd	3414	4	1	0	Complete	100	0	Geminiviridae	eukaryote
v02fe	40322	60	25	1	Complete	100	0	Siphoviridae	prokaryote
v02ff	4406	5	2	0	High-quality	98.1	0	unclassified	unclassified
v0300	2598	4	2	0	High-quality	100	0	Circoviridae	eukaryote
v0301	30360	36	4	1	Medium-quality	62.8	0	unclassified	unclassified
v0302	35840	47	4	1	Medium-quality	85.6	0	unclassified	unclassified
v0303	123545	180	32	1	Medium-quality	70.38	0	unclassified	unclassified
v0304	44158	68	30	1	High-quality	97.63	0	Myoviridae	prokaryote
v0305	2000	2	1	0	Medium-quality	68.03	0	unclassified	unclassified
v0306	6039	8	2	0	High-quality	100	0	Microviridae	prokaryote
v0307	2946	4	4	0	Medium-quality	64.16	0	Microviridae	prokaryote
v0308	28488	20	1	8	Medium-quality	64.48	0	unclassified	unclassified
v0309	36653	57	15	1	High-quality	100	0	unclassified	unclassified
v030a	33490	48	21	1	Medium-quality	83.53	0	Siphoviridae	prokaryote
v030b	2168	2	2	0	Medium-quality	67.69	0	Circoviridae	eukaryote
v030c	80262	66	2	20	High-quality	98.21	0	unclassified	unclassified
v030d	34805	57	20	0	Complete	100	0	unclassified	unclassified
v030e	17822	25	17	0	Medium-quality	51.6	0	Siphoviridae	prokaryote
v030f	2265	4	0	0	High-quality	100	0	unclassified	unclassified
v0310	35967	47	25	0	Medium-quality	59.78	0	Siphoviridae	prokaryote
v0311	22078	23	18	0	Medium-quality	55.56	0	Siphoviridae	prokaryote
v0312	35874	35	1	11	Medium-quality	71.26	0	unclassified	unclassified
v0313	43343	66	31	0	Complete	100	0	unclassified	unclassified
v0314	37596	60	13	0	Complete	100	0	unclassified	unclassified
v0315	68838	91	17	0	High-quality	100	0	unclassified	unclassified
v0316	43968	63	30	0	Complete	100	0	Myoviridae	prokaryote
v0317	60881	89	21	1	Complete	100	0	unclassified	unclassified
v0318	3406	5	1	0	Complete	100	0	Circoviridae	eukaryote
v0319	3558	5	2	0	Medium-quality	50.08	0	unclassified	unclassified
v031a	2049	3	1	0	Complete	100	0	Circoviridae	eukaryote
v031b	4312	6	3	0	Complete	100	0	Microviridae	prokaryote

v031c	2012	3	1	0	Complete	100	0	Circoviridae	eukaryote
v031d	2667	4	2	0	Medium-quality	64.09	0	Circoviridae	eukaryote
v031e	2616	4	2	0	Complete	100	0	Circoviridae	eukaryote
v031f	2086	3	1	0	Medium-quality	63.68	0	Circoviridae	eukaryote
v0320	6494	7	4	0	Complete	100	0	unclassified	unclassified
v0321	17668	27	7	0	High-quality	92.53	0	unclassified	unclassified
v0322	5007	9	6	0	Complete	100	0	Microviridae	prokaryote
v0323	19498	27	12	0	Medium-quality	83.36	0	Salasmaviridae	prokaryote
v0324	17307	23	7	0	Complete	100	0	unclassified	unclassified
v0325	16558	21	8	0	Medium-quality	89.65	0	unclassified	unclassified
v0326	6346	9	4	0	Complete	100	0	unclassified	unclassified
v0327	6503	9	4	0	Complete	100	0	Microviridae	prokaryote
v0328	5281	10	6	0	Complete	100	0	Microviridae	prokaryote
v0329	5231	7	5	0	Complete	100	0	Microviridae	prokaryote
v032a	6148	9	4	0	Complete	100	0	unclassified	unclassified
v032b	5852	9	4	0	Complete	100	0	Microviridae	prokaryote
v032c	6068	9	3	0	Complete	100	0	unclassified	unclassified
v032d	5458	9	7	0	Complete	100	0	Microviridae	prokaryote
v032e	5305	8	6	0	Complete	100	0	Microviridae	prokaryote
v032f	6445	8	3	0	Complete	100	0	unclassified	unclassified
v0330	5522	9	6	0	Complete	100	0	Microviridae	prokaryote
v0331	5687	8	3	0	Complete	100	0	unclassified	unclassified
v0332	6324	8	3	0	Complete	100	0	unclassified	unclassified
v0333	3519	4	0	0	High-quality	100	0	unclassified	unclassified
v0334	2204	4	3	0	High-quality	100	0	Genomoviridae	eukaryote
v0335	4338	4	3	0	Medium-quality	71.09	0	unclassified	unclassified
v0336	43800	52	3	2	High-quality	92.91	0	unclassified	unclassified
v0337	6182	8	2	0	Complete	100	0	unclassified	unclassified
v0338	6108	9	2	0	Complete	100	0	unclassified	unclassified
v0339	5911	9	5	0	High-quality	100	0	Microviridae	prokaryote
v033a	48003	80	52	0	Complete	100	0	Siphoviridae	prokaryote
v033b	43275	65	19	0	Complete	100	0	unclassified	unclassified
v033c	2454	3	2	0	High-quality	100	0	unclassified	unclassified
v033d	3303	3	2	0	Medium-quality	50.78	0	unclassified	unclassified
v033e	4718	7	1	0	Medium-quality	78.86	0	unclassified	unclassified
v033f	10097	13	5	1	Medium-quality	84.35	0	Podoviridae	prokaryote
v0340	5600	8	5	0	High-quality	98.96	0	Microviridae	prokaryote
v0341	13499	18	5	0	High-quality	98.58	0	unclassified	unclassified
v0342	6697	9	4	0	Complete	100	0	unclassified	unclassified
v0343	6025	7	3	0	Complete	100	0	unclassified	unclassified

v0344	5989	9	4	0	Complete	100	0	Microviridae	prokaryote
v0345	6604	8	4	0	Complete	100	0	unclassified	unclassified
v0346	7146	10	4	0	High-quality	100	0	unclassified	unclassified
v0347	6011	9	3	0	Complete	100	0	unclassified	unclassified
v0348	44224	80	16	0	Complete	100	0	unclassified	unclassified
v0349	6037	8	3	0	Complete	100	0	unclassified	unclassified
v034a	62103	94	17	4	High-quality	94.7	0	Quimbyviridae	prokaryote
v034b	5907	7	3	0	Complete	100	0	unclassified	unclassified
v034c	6626	9	4	0	Complete	100	0	unclassified	unclassified
v034d	6030	9	3	0	Complete	100	0	unclassified	unclassified
v034e	5666	8	3	0	Complete	100	0	unclassified	unclassified
v034f	6240	6	3	0	Complete	100	0	Microviridae	prokaryote
v0350	2485	2	2	0	Complete	100	0	Circoviridae	eukaryote
v0351	6168	10	3	0	Complete	100	0	unclassified	unclassified
v0352	6158	8	4	0	Complete	100	0	unclassified	unclassified
v0353	6453	7	4	0	Complete	100	0	unclassified	unclassified
v0354	6010	9	3	0	Complete	100	0	unclassified	unclassified
v0355	32702	58	22	0	Complete	100	0	Siphoviridae	prokaryote
v0356	6638	11	4	0	Complete	100	0	Microviridae	prokaryote
v0357	5278	9	7	0	Complete	100	0	Microviridae	prokaryote
v0358	5751	10	7	0	Complete	100	0	Microviridae	prokaryote
v0359	13718	18	5	0	High-quality	100	0	unclassified	unclassified
v035a	6114	10	3	0	Complete	100	0	unclassified	unclassified
v035b	5875	10	5	0	Complete	100	0	Microviridae	prokaryote
v035c	5378	9	6	0	Complete	100	0	Microviridae	prokaryote
v035d	3807	7	1	0	Medium-quality	58.18	0	unclassified	unclassified
v035e	5898	9	5	0	Complete	100	0	Microviridae	prokaryote
v035f	5492	8	7	0	Complete	100	0	Microviridae	prokaryote
v0360	6068	8	3	0	Complete	100	0	unclassified	unclassified
v0361	2312	3	2	0	Complete	100	0	unclassified	unclassified
v0362	3575	3	2	0	Medium-quality	50.32	0	unclassified	unclassified
v0363	6516	9	3	0	Complete	100	0	Microviridae	prokaryote
v0364	15467	26	8	0	Medium-quality	55.55	0	unclassified	unclassified
v0365	6170	10	3	0	Complete	100	0	unclassified	unclassified
v0366	6148	10	3	0	High-quality	100	0	unclassified	unclassified
v0367	14447	21	7	0	Medium-quality	63.05	0	unclassified	unclassified
v0368	4973	7	5	0	Complete	100	0	Microviridae	prokaryote
v0369	45693	61	13	1	Complete	100	0	Siphoviridae	prokaryote
v036a	34605	53	9	0	Medium-quality	52.68	0	unclassified	unclassified
v036b	9423	11	3	0	High-quality	100	0	unclassified	unclassified

v036c	5163	6	3	0	Medium-quality	88.57	0	unclassified	unclassified
v036d	5747	9	4	0	Complete	100	0	Microviridae	prokaryote
v036e	6972	12	3	0	High-quality	100	0	unclassified	unclassified
v036f	15453	21	7	0	Medium-quality	68.37	0	unclassified	unclassified
v0370	21964	32	11	0	Medium-quality	64.63	0	unclassified	unclassified
v0371	5496	9	6	0	Complete	100	0	Microviridae	prokaryote
v0372	2474	4	3	0	Medium-quality	52.24	0	Microviridae	prokaryote
v0373	15370	18	5	0	Medium-quality	53.65	0	unclassified	unclassified
v0374	16957	25	8	0	Medium-quality	50.56	0	unclassified	unclassified
v0375	6121	8	3	0	Complete	100	0	unclassified	unclassified
v0376	4630	5	3	0	Medium-quality	75.77	0	unclassified	unclassified
v0377	6196	10	2	0	Complete	100	0	unclassified	unclassified
v0378	17177	22	9	0	High-quality	93.05	0	unclassified	unclassified
v0379	32334	55	19	0	Medium-quality	62.49	0	Myoviridae	prokaryote
v037a	40522	70	21	0	Complete	100	0	unclassified	unclassified
v037b	24869	41	9	1	Medium-quality	75.03	0	unclassified	unclassified
v037c	6667	9	4	0	Complete	100	0	Microviridae	prokaryote
v037d	8035	11	3	0	Medium-quality	58.85	0	unclassified	unclassified
v037e	58207	103	19	1	Complete	100	0	unclassified	unclassified
v037f	15427	18	7	0	Medium-quality	66.63	0	unclassified	unclassified
v0380	41396	55	10	0	Complete	100	0	Siphoviridae	prokaryote
v0381	45728	69	17	1	High-quality	100	0	unclassified	unclassified
v0382	19306	23	7	0	Medium-quality	66.61	0	unclassified	unclassified
v0383	5666	8	2	0	Complete	100	0	unclassified	unclassified
v0384	3244	6	0	0	Medium-quality	52.37	0	unclassified	unclassified
v0385	4053	4	3	0	Medium-quality	73.25	0	unclassified	unclassified
v0386	78083	106	10	1	Complete	100	0	Flandersviridae	prokaryote
v0387	6042	13	0	0	Complete	100	0	Inoviridae	prokaryote
v0388	5936	8	4	0	Complete	100	0	Microviridae	prokaryote
v0389	5591	11	6	0	Complete	100	0	Microviridae	prokaryote
v038a	6028	9	2	0	Complete	100	0	unclassified	unclassified
v038b	4808	8	6	0	Complete	100	0	Microviridae	prokaryote
v038c	5826	8	5	0	Complete	100	0	Microviridae	prokaryote
v038d	6692	9	3	0	Complete	100	0	unclassified	unclassified
v038e	6278	11	3	0	Complete	100	0	unclassified	unclassified
v038f	3587	3	2	0	Medium-quality	58.82	0	unclassified	unclassified
v0390	14301	20	6	0	Medium-quality	64.29	0	Salasmaviridae	prokaryote
v0391	12582	17	4	0	High-quality	92.08	0	unclassified	unclassified
v0392	77896	130	16	5	Complete	100	0	unclassified	unclassified
v0393	41153	78	17	0	Complete	100	0	Siphoviridae	prokaryote

v0394	3579	5	3	0	Medium-quality	74.61	0	Microviridae	prokaryote
v0395	11630	13	5	0	Medium-quality	73.5	0	unclassified	unclassified
v0396	3618	5	0	0	Medium-quality	56.34	0	Inoviridae	prokaryote
v0397	4988	6	5	0	High-quality	100	0	Microviridae	prokaryote
v0398	47349	83	51	0	Complete	100	0	Siphoviridae	prokaryote
v0399	97298	146	36	1	High-quality	100	0	unclassified	unclassified
v039a	50892	76	26	0	Medium-quality	84.97	0	Siphoviridae	prokaryote
v039b	37799	62	29	1	High-quality	91.58	0	Myoviridae	prokaryote
v039c	13399	17	4	0	Complete	100	0	unclassified	unclassified
v039d	6461	10	2	0	High-quality	100	0	unclassified	unclassified
v039e	6503	8	3	0	Complete	100	0	Microviridae	prokaryote
v039f	6288	7	4	0	Complete	100	0	unclassified	unclassified
v03a0	5387	9	3	0	Complete	100	0	Microviridae	prokaryote
v03a1	130368	171	15	2	High-quality	100	0	unclassified	unclassified
v03a2	3522	5	2	0	Medium-quality	54.19	0	unclassified	unclassified
v03a3	39770	66	14	0	Medium-quality	88.44	0	unclassified	unclassified
v03a4	41068	68	20	1	Complete	100	0	Siphoviridae	prokaryote
v03a5	4675	6	1	0	High-quality	100	0	unclassified	unclassified
v03a6	5871	9	2	0	Complete	100	0	unclassified	unclassified
v03a7	43514	71	13	0	High-quality	100	0	unclassified	unclassified
v03a8	3271	5	1	0	Medium-quality	50.23	0	unclassified	unclassified
v03a9	158788	212	49	5	High-quality	100	0	unclassified	unclassified
v03aa	54985	88	24	1	High-quality	100	0	unclassified	unclassified
v03ab	42153	66	26	0	High-quality	93.43	0	unclassified	unclassified
v03ac	39568	63	30	1	Complete	100	0	Siphoviridae	prokaryote
v03ad	63434	86	19	0	Complete	100	0	unclassified	unclassified
v03ae	60019	101	22	1	Complete	100	0	unclassified	unclassified
v03af	5233	8	7	0	Complete	100	0	Microviridae	prokaryote
v03b0	5631	8	4	0	Complete	100	0	Microviridae	prokaryote
v03b1	5115	8	6	0	Complete	100	0	Microviridae	prokaryote
v03b2	40093	70	16	0	Complete	100	0	unclassified	unclassified
v03b3	5144	8	6	0	Complete	100	0	Microviridae	prokaryote
v03b4	5525	7	3	0	Complete	100	0	unclassified	unclassified
v03b5	6409	6	3	0	Complete	100	0	unclassified	unclassified
v03b6	6224	9	3	0	Complete	100	0	unclassified	unclassified
v03b7	6557	9	4	0	Complete	100	0	Microviridae	prokaryote
v03b8	6064	9	3	0	Complete	100	0	unclassified	unclassified
v03b9	6070	10	4	0	Complete	100	0	unclassified	unclassified
v03ba	4634	7	2	0	Complete	100	0	Microviridae	prokaryote
v03bb	5718	8	5	0	Complete	100	0	Microviridae	prokaryote

v03bc	5865	8	4	0	High-quality	96.05	0	unclassified	unclassified
v03bd	13965	20	4	0	Complete	100	0	unclassified	unclassified
v03be	41963	71	19	0	High-quality	99.28	0	unclassified	unclassified
v03bf	72447	59	5	0	Medium-quality	66.61	0	Podoviridae_crAss-like	prokaryote
v03c0	5928	7	2	0	High-quality	99.18	0	unclassified	unclassified
v03c1	6115	7	2	0	Complete	100	0	unclassified	unclassified
v03c2	10958	17	5	0	High-quality	100	0	unclassified	unclassified
v03c3	4746	7	3	0	Complete	100	0	Microviridae	prokaryote
v03c4	6192	10	4	0	Complete	100	0	unclassified	unclassified
v03c5	6311	7	3	0	Complete	100	0	unclassified	unclassified
v03c6	5371	9	7	0	Complete	100	0	Microviridae	prokaryote
v03c7	6441	9	3	0	Complete	100	0	Microviridae	prokaryote
v03c8	6267	8	3	0	Complete	100	0	unclassified	unclassified
v03c9	43699	82	18	0	Complete	100	0	unclassified	unclassified
v03ca	8636	9	4	0	High-quality	100	0	unclassified	unclassified
v03cb	49667	77	15	1	High-quality	100	0	unclassified	unclassified
v03cc	16177	21	10	0	Medium-quality	83.98	0	unclassified	unclassified
v03cd	6434	8	3	0	Complete	100	0	unclassified	unclassified
v03ce	14479	22	8	0	Medium-quality	75.76	0	unclassified	unclassified
v03cf	4668	6	4	0	High-quality	97.3	0	Microviridae	prokaryote
v03d0	8224	15	1	0	High-quality	100	0	Inoviridae	prokaryote
v03d1	2984	10	0	0	Medium-quality	55.32	0	Inoviridae	prokaryote
v03d2	99736	129	22	4	High-quality	100	0	unclassified	unclassified
v03d3	32399	48	11	0	Medium-quality	70.7	0	unclassified	unclassified
v03d4	20348	38	9	0	Medium-quality	51.97	0	unclassified	unclassified
v03d5	11283	17	4	0	High-quality	100	0	unclassified	unclassified
v03d6	49817	77	68	0	High-quality	100	0	Drexlerviridae	prokaryote
v03d7	111943	139	8	3	Complete	100	0	unclassified	unclassified
v03d8	48137	68	18	0	Complete	100	0	Siphoviridae	prokaryote
v03d9	6137	8	4	0	Complete	100	0	unclassified	unclassified
v03da	174074	288	39	1	High-quality	98.7	0	unclassified	unclassified
v03db	37260	63	28	0	Complete	100	0	unclassified	unclassified
v03dc	40645	49	33	0	Medium-quality	84.82	0	Siphoviridae	prokaryote
v03dd	166979	284	244	0	Complete	100	0	Myoviridae	prokaryote
v03de	70730	88	61	0	Complete	100	0	Schitoviridae	prokaryote
v03df	6402	8	4	0	Complete	100	0	unclassified	unclassified
v03e0	33296	43	22	0	Medium-quality	55.28	0	Siphoviridae	prokaryote
v03e1	48227	80	21	1	Complete	100	0	unclassified	unclassified
v03e2	6240	9	3	0	Complete	100	0	unclassified	unclassified
v03e3	5777	9	5	0	Complete	100	0	Microviridae	prokaryote

v03e4	6507	9	3	0	Complete	100	0	Microviridae	prokaryote
v03e5	6027	9	2	0	Complete	100	0	unclassified	unclassified
v03e6	42853	52	45	0	Complete	100	0	Autographiviridae	prokaryote
v03e7	6612	9	3	0	Complete	100	0	Microviridae	prokaryote
v03e8	5320	6	3	0	Medium-quality	87.13	0	unclassified	unclassified
v03e9	5610	7	4	0	High-quality	91.85	0	unclassified	unclassified
v03ea	5238	7	6	0	Complete	100	0	Microviridae	prokaryote
v03eb	5058	2	2	0	High-quality	100	0	Parvoviridae	eukaryote
v03ec	24173	34	15	2	Medium-quality	68.25	0	Myoviridae	prokaryote
v03ed	87198	96	29	0	Medium-quality	60.5	0	unclassified	unclassified
v03ee	54888	95	13	2	Complete	100	0	unclassified	unclassified
v03ef	52622	81	26	2	Complete	100	0	unclassified	unclassified
v03f0	27596	38	14	1	Medium-quality	73.42	0	unclassified	unclassified
v03f1	6737	10	3	0	High-quality	100	0	Microviridae	prokaryote
v03f2	4165	3	1	0	Medium-quality	64.75	0	Microviridae	prokaryote
v03f3	6095	8	3	0	High-quality	100	0	unclassified	unclassified
v03f4	58873	85	20	3	Complete	100	0	Quimbyviridae	prokaryote
v03f5	6275	6	4	0	High-quality	100	0	unclassified	unclassified
v03f6	18141	24	9	0	High-quality	90.23	0	unclassified	unclassified
v03f7	42046	75	19	2	High-quality	97.26	0	unclassified	unclassified
v03f8	102137	106	19	0	High-quality	100	0	Podoviridae_crAss-like	prokaryote
v03f9	12977	19	5	0	Complete	100	0	unclassified	unclassified
v03fa	20892	26	15	0	Medium-quality	58.07	0	Siphoviridae	prokaryote
v03fb	16610	24	7	0	Medium-quality	87.73	0	unclassified	unclassified
v03fc	55324	86	16	1	Complete	100	0	unclassified	unclassified
v03fd	14689	22	1	2	Medium-quality	69.89	0	unclassified	unclassified
v03fe	86297	134	24	3	Complete	100	0	unclassified	unclassified
v03ff	8243	14	1	0	Complete	100	0	Inoviridae	prokaryote
v0400	5063	8	7	0	Complete	100	0	Microviridae	prokaryote
v0401	10241	16	1	0	Complete	100	0	unclassified	unclassified
v0402	6649	9	3	0	Complete	100	0	unclassified	unclassified
v0403	6083	7	3	0	Complete	100	0	unclassified	unclassified
v0404	5231	8	7	0	Complete	100	0	Microviridae	prokaryote
v0405	4883	9	4	0	Complete	100	0	Microviridae	prokaryote
v0406	6075	9	4	0	Complete	100	0	unclassified	unclassified
v0407	6470	8	4	0	Complete	100	0	Microviridae	prokaryote
v0408	4810	8	5	0	Complete	100	0	Microviridae	prokaryote
v0409	5683	8	4	0	Complete	100	0	Microviridae	prokaryote
v040a	5761	7	3	0	High-quality	94.4	0	unclassified	unclassified
v040b	4409	2	1	0	Medium-quality	62.78	0	Metaviridae	eukaryote

v040c	5101	8	7	0	Complete	100	0	Microviridae	prokaryote
v040d	4276	4	2	0	Medium-quality	71.99	0	unclassified	unclassified
v040e	34039	57	25	1	High-quality	100	0	Myoviridae	prokaryote
v040f	6673	10	3	0	Complete	100	0	unclassified	unclassified
v0410	7094	8	4	0	High-quality	100	0	Microviridae	prokaryote
v0411	42803	68	16	1	High-quality	100	0	unclassified	unclassified
v0412	40071	61	22	3	High-quality	100	0	Siphoviridae	prokaryote
v0413	42623	69	17	0	Complete	100	0	unclassified	unclassified
v0414	4270	6	5	0	Medium-quality	88.95	0	Microviridae	prokaryote
v0415	75315	73	2	44	Medium-quality	81.88	0	unclassified	unclassified
v0416	14459	21	2	0	High-quality	98.88	0	unclassified	unclassified
v0417	4162	4	2	0	Medium-quality	71.21	0	unclassified	unclassified
v0418	179444	290	44	2	Complete	100	0	unclassified	unclassified
v0419	5921	8	3	0	High-quality	97	0	unclassified	unclassified
v041a	47796	63	8	1	Complete	100	0	unclassified	unclassified
v041b	5760	9	4	0	Complete	100	0	unclassified	unclassified
v041c	6590	8	3	0	Complete	100	0	unclassified	unclassified
v041d	3119	3	1	0	Medium-quality	50.32	0	Metaviridae	eukaryote
v041e	3983	4	2	0	Medium-quality	65.25	0	unclassified	unclassified
v041f	6598	6	3	0	Complete	100	0	unclassified	unclassified
v0420	104977	109	19	2	High-quality	100	0	unclassified	unclassified
v0421	5886	10	4	0	Complete	100	0	Microviridae	prokaryote
v0422	6142	8	3	0	Complete	100	0	unclassified	unclassified
v0423	6762	9	3	0	Complete	100	0	unclassified	unclassified
v0424	5613	8	3	0	Complete	100	0	unclassified	unclassified
v0425	6936	8	3	0	Complete	100	0	unclassified	unclassified
v0426	5714	9	6	0	Complete	100	0	Microviridae	prokaryote
v0427	9242	17	1	0	Complete	100	0	unclassified	unclassified
v0428	6377	9	4	0	Complete	100	0	unclassified	unclassified
v0429	95157	84	1	69	Medium-quality	61.57	0	unclassified	unclassified
v042a	6183	10	2	0	High-quality	100	0	unclassified	unclassified
v042b	5094	8	6	0	Complete	100	0	Microviridae	prokaryote
v042c	16858	26	8	0	Medium-quality	89.1	0	unclassified	unclassified
v042d	5451	9	4	0	Complete	100	0	Microviridae	prokaryote
v042e	6040	8	2	0	Complete	100	0	unclassified	unclassified
v042f	6090	8	2	0	Complete	100	0	unclassified	unclassified
v0430	2010	3	1	0	Complete	100	0	unclassified	unclassified
v0431	16726	21	8	0	Medium-quality	89.68	0	unclassified	unclassified
v0432	6327	9	4	0	Complete	100	0	unclassified	unclassified
v0433	6650	7	3	0	Complete	100	0	unclassified	unclassified

v0434	19366	22	10	0	High-quality	100	0	unclassified	unclassified
v0435	72514	90	15	1	Medium-quality	83.12	0	unclassified	unclassified
v0436	34100	52	22	1	Complete	100	0	Siphoviridae	prokaryote
v0437	6734	10	3	0	Complete	100	0	unclassified	unclassified
v0438	140200	200	24	5	Medium-quality	81.06	0	unclassified	unclassified
v0439	5497	10	6	0	Complete	100	0	Microviridae	prokaryote
v043a	2842	3	2	0	Medium-quality	56.08	0	Microviridae	prokaryote
v043b	21563	32	14	1	Medium-quality	56.55	0	unclassified	unclassified
v043c	6285	8	3	0	Complete	100	0	unclassified	unclassified
v043d	2332	3	2	0	Complete	100	0	Genomoviridae	eukaryote
v043e	6267	10	4	0	Complete	100	0	unclassified	unclassified
v043f	5992	10	2	0	Complete	100	0	unclassified	unclassified
v0440	5157	8	6	0	Complete	100	0	Microviridae	prokaryote
v0441	42370	62	26	1	Complete	100	0	Siphoviridae	prokaryote
v0442	40512	69	17	4	High-quality	97.59	0	Siphoviridae	prokaryote
v0443	4579	6	3	0	Medium-quality	75	0	unclassified	unclassified
v0444	62374	99	15	3	High-quality	100	0	unclassified	unclassified
v0445	6123	9	2	0	Complete	100	0	unclassified	unclassified
v0446	53164	73	47	0	High-quality	92.47	0	Siphoviridae	prokaryote
v0447	3096	3	2	0	Medium-quality	89.59	0	Anelloviridae	eukaryote
v0448	80754	120	22	1	Complete	100	0	unclassified	unclassified
v0449	43346	76	21	1	Complete	100	0	unclassified	unclassified
v044a	5574	11	4	0	Complete	100	0	Microviridae	prokaryote
v044b	6318	5	3	0	Complete	100	0	unclassified	unclassified
v044c	5766	8	2	0	High-quality	100	0	unclassified	unclassified
v044d	12543	17	6	0	High-quality	100	0	unclassified	unclassified
v044e	52389	88	23	1	Complete	100	0	unclassified	unclassified
v044f	4293	7	6	0	Medium-quality	89.4	0	Microviridae	prokaryote
v0450	6548	12	8	0	High-quality	100	0	Microviridae	prokaryote
v0451	6552	9	4	0	Complete	100	0	Microviridae	prokaryote
v0452	6227	12	0	0	High-quality	99.84	0	unclassified	unclassified
v0453	6249	8	3	0	Complete	100	0	unclassified	unclassified
v0454	6180	8	3	0	High-quality	100	0	unclassified	unclassified
v0455	42958	70	30	0	Complete	100	0	Myoviridae	prokaryote
v0456	2506	6	2	0	High-quality	100	0	Genomoviridae	eukaryote
v0457	37831	64	22	0	High-quality	93.51	0	Siphoviridae	prokaryote
v0458	6143	13	0	0	High-quality	97.34	0	unclassified	unclassified
v0459	20262	17	2	4	Medium-quality	52.82	0	unclassified	unclassified
v045a	105662	87	2	27	Medium-quality	60.29	0	unclassified	unclassified
v045b	65275	81	11	1	High-quality	100	0	unclassified	unclassified

v045c	3646	7	1	0	Medium-quality	60.97	0	unclassified	unclassified
v045d	25285	43	17	0	Medium-quality	52.7	0	Siphoviridae	prokaryote
v045e	2756	5	4	0	High-quality	100	0	Genomoviridae	eukaryote
v045f	16749	24	10	0	Medium-quality	68.49	0	unclassified	unclassified
v0460	6188	2	1	0	Medium-quality	86.07	0	unclassified	unclassified
v0461	13303	20	4	0	High-quality	95.78	0	unclassified	unclassified
v0462	45489	62	19	0	Complete	100	0	unclassified	unclassified
v0463	3743	5	3	0	Medium-quality	63.78	0	unclassified	unclassified
v0464	15691	21	6	0	Medium-quality	52.81	0	unclassified	unclassified
v0465	9204	16	3	0	Medium-quality	69.65	0	unclassified	unclassified
v0466	6551	9	4	0	Complete	100	0	Microviridae	prokaryote
v0467	15048	21	6	0	Medium-quality	66.21	0	unclassified	unclassified
v0468	40049	56	18	0	Medium-quality	80.54	0	unclassified	unclassified
v0469	5575	9	7	0	High-quality	100	0	Microviridae	prokaryote
v046a	6311	7	4	0	Complete	100	0	unclassified	unclassified
v046b	58895	58	1	16	Medium-quality	56.39	0	unclassified	unclassified
v046c	35669	60	21	0	High-quality	100	0	Siphoviridae	prokaryote
v046d	17773	27	4	1	Medium-quality	53.42	0	unclassified	unclassified
v046e	5860	9	4	0	High-quality	100	0	unclassified	unclassified
v046f	6011	8	3	0	High-quality	100	0	unclassified	unclassified
v0470	61318	105	25	0	High-quality	100	0	unclassified	unclassified
v0471	44919	67	15	1	High-quality	100	0	unclassified	unclassified
v0472	5094	10	0	0	Complete	100	0	unclassified	unclassified
v0473	117204	150	31	3	Complete	100	0	unclassified	unclassified
v0474	5287	9	7	0	High-quality	100	0	Microviridae	prokaryote
v0475	6304	8	3	0	Complete	100	0	unclassified	unclassified
v0476	5954	7	4	0	Complete	100	0	unclassified	unclassified
v0477	16339	22	8	0	Medium-quality	85.76	0	unclassified	unclassified
v0478	24106	34	8	1	Medium-quality	53.88	0	Siphoviridae	prokaryote
v0479	16281	24	7	0	Medium-quality	84.5	0	unclassified	unclassified
v047a	51448	79	67	0	Complete	100	0	Drexlerviridae	prokaryote
v047b	6608	8	3	0	Complete	100	0	unclassified	unclassified
v047c	6291	8	3	0	Complete	100	0	unclassified	unclassified
v047d	6116	7	3	0	Complete	100	0	unclassified	unclassified
v047e	5957	7	2	0	Complete	100	0	unclassified	unclassified
v047f	6447	8	3	0	High-quality	100	0	unclassified	unclassified
v0480	6685	8	4	0	High-quality	100	0	unclassified	unclassified
v0481	14980	17	7	0	Medium-quality	78.52	0	Salasmaviridae	prokaryote
v0482	4499	6	3	0	Medium-quality	73.7	0	unclassified	unclassified
v0483	6101	11	3	0	Complete	100	0	unclassified	unclassified

v0484	5963	9	3	0	Complete	100	0	unclassified	unclassified
v0485	5219	8	6	0	Complete	100	0	Microviridae	prokaryote
v0486	5449	9	7	0	Complete	100	0	Microviridae	prokaryote
v0487	17354	21	15	0	Medium-quality	51.37	0	Myoviridae	prokaryote
v0488	5999	8	2	0	Complete	100	0	unclassified	unclassified
v0489	80810	130	25	2	High-quality	100	0	unclassified	unclassified
v048a	6544	7	4	0	Complete	100	0	unclassified	unclassified
v048b	6545	8	4	0	Complete	100	0	unclassified	unclassified
v048c	2452	2	1	0	Medium-quality	51.11	0	Microviridae	prokaryote
v048d	6705	7	4	0	High-quality	100	0	unclassified	unclassified
v048e	13066	16	4	0	High-quality	95.88	0	unclassified	unclassified
v048f	3406	5	3	0	Medium-quality	55.8	0	unclassified	unclassified
v0490	15913	20	6	0	Medium-quality	70.72	0	Salasmaviridae	prokaryote
v0491	75326	127	19	4	Complete	100	0	Quimbyviridae	prokaryote
v0492	5807	8	3	0	Complete	100	0	unclassified	unclassified
v0493	6169	8	2	0	Complete	100	0	unclassified	unclassified
v0494	6241	8	3	0	Complete	100	0	Microviridae	prokaryote
v0495	8369	16	2	0	Medium-quality	56.71	0	Tectiviridae	prokaryote
v0496	46652	70	30	2	Medium-quality	74.34	0	unclassified	unclassified
v0497	39908	49	39	0	Complete	100	0	Podoviridae	prokaryote
v0498	13308	17	6	0	High-quality	95.82	0	unclassified	unclassified
v0499	6761	8	3	0	Complete	100	0	unclassified	unclassified
v049a	13050	20	5	0	Complete	100	0	unclassified	unclassified
v049b	5682	10	1	0	Complete	100	0	Inoviridae	prokaryote
v049c	5559	9	5	0	Complete	100	0	Microviridae	prokaryote
v049d	6478	9	4	0	Complete	100	0	Microviridae	prokaryote
v049e	6155	9	2	0	Complete	100	0	unclassified	unclassified
v049f	7707	11	2	0	High-quality	100	0	unclassified	unclassified
v04a0	5866	9	3	0	Complete	100	0	unclassified	unclassified
v04a1	5984	8	4	0	Complete	100	0	unclassified	unclassified
v04a2	5998	8	2	0	Complete	100	0	unclassified	unclassified
v04a3	5220	6	6	0	High-quality	100	0	Microviridae	prokaryote
v04a4	6189	10	2	0	Complete	100	0	unclassified	unclassified
v04a5	7568	8	4	0	High-quality	100	0	Microviridae	prokaryote
v04a6	5817	10	6	0	Complete	100	0	Microviridae	prokaryote
v04a7	6351	7	3	0	Complete	100	0	unclassified	unclassified
v04a8	6177	8	3	0	High-quality	100	0	unclassified	unclassified
v04a9	5178	9	0	0	High-quality	100	0	Inoviridae	prokaryote
v04aa	5344	8	6	0	Complete	100	0	Microviridae	prokaryote
v04ab	5945	9	4	0	Complete	100	0	Microviridae	prokaryote

v04ac	5817	9	5	0	Complete	100	0	Microviridae	prokaryote
v04ad	5674	8	6	0	Complete	100	0	Microviridae	prokaryote
v04ae	6299	7	3	0	Complete	100	0	unclassified	unclassified
v04af	5617	9	5	0	High-quality	100	0	Microviridae	prokaryote
v04b0	5598	8	6	0	Complete	100	0	Microviridae	prokaryote
v04b1	6006	8	3	0	High-quality	99.39	0	unclassified	unclassified
v04b2	3816	9	4	0	Medium-quality	66.25	0	Microviridae	prokaryote
v04b3	7600	7	3	0	High-quality	100	0	unclassified	unclassified
v04b4	55640	75	13	0	High-quality	92.35	0	unclassified	unclassified
v04b5	47622	67	19	0	High-quality	100	0	unclassified	unclassified
v04b6	6137	8	2	0	Complete	100	0	unclassified	unclassified
v04b7	6243	7	4	0	Complete	100	0	unclassified	unclassified
v04b8	6160	7	3	0	Complete	100	0	unclassified	unclassified
v04b9	31169	46	12	1	Medium-quality	54.53	0	unclassified	unclassified
v04ba	6436	7	4	0	High-quality	100	0	unclassified	unclassified
v04bb	6191	8	4	0	Complete	100	0	unclassified	unclassified
v04bc	15092	17	0	0	Complete	100	0	unclassified	unclassified
v04bd	4857	7	2	0	Complete	100	0	Microviridae	prokaryote
v04be	6140	9	2	0	Complete	100	0	unclassified	unclassified
v04bf	6055	11	1	0	Complete	100	0	unclassified	unclassified
v04c0	6183	6	3	0	Complete	100	0	unclassified	unclassified
v04c1	4925	7	5	0	Complete	100	0	Microviridae	prokaryote
v04c2	4891	6	3	0	Complete	100	0	unclassified	unclassified
v04c3	7109	8	3	0	Complete	100	0	unclassified	unclassified
v04c4	5655	12	6	0	Complete	100	0	Microviridae	prokaryote
v04c5	3366	6	1	0	Medium-quality	54.21	0	unclassified	unclassified
v04c6	15740	21	6	0	Medium-quality	68.51	0	unclassified	unclassified
v04c7	34925	49	12	0	High-quality	97.51	0	unclassified	unclassified
v04c8	3182	6	0	1	Medium-quality	52.95	0	unclassified	unclassified
v04c9	4281	8	2	0	Medium-quality	65.74	0	unclassified	unclassified
v04ca	6391	12	1	1	High-quality	100	0	unclassified	unclassified
v04cb	4245	7	2	0	Medium-quality	87.38	0	unclassified	unclassified
v04cc	4978	7	6	0	Complete	100	0	Microviridae	prokaryote
v04cd	6336	10	3	0	High-quality	100	0	unclassified	unclassified
v04ce	10372	11	6	0	Medium-quality	53.08	0	Salasmaviridae	prokaryote
v04cf	30459	45	9	0	High-quality	90.67	0	Podoviridae	prokaryote
v04d0	6098	8	2	0	Complete	100	0	unclassified	unclassified
v04d1	31057	48	14	0	Medium-quality	68.21	0	Siphoviridae	prokaryote
v04d2	44670	71	36	0	High-quality	95.11	0	Myoviridae	prokaryote
v04d3	67250	89	14	2	Complete	100	0	unclassified	unclassified

v04d4	36859	50	23	0	High-quality	99.83	0	Siphoviridae	prokaryote
v04d5	36210	55	37	0	High-quality	95.01	0	Siphoviridae	prokaryote
v04d6	58292	71	27	0	High-quality	98.86	0	Siphoviridae	prokaryote
v04d7	46261	74	26	1	Complete	100	0	unclassified	unclassified
v04d8	37787	61	35	0	Medium-quality	80.45	0	Siphoviridae	prokaryote
v04d9	19137	25	17	0	Medium-quality	54.14	0	Siphoviridae	prokaryote
v04da	22464	27	14	0	Medium-quality	52.5	0	Siphoviridae	prokaryote
v04db	19235	20	6	0	Medium-quality	63.15	0	unclassified	unclassified
v04dc	11167	14	11	0	Medium-quality	75.8	0	Siphoviridae	prokaryote
v04dd	40581	59	30	0	Medium-quality	67.46	0	Siphoviridae	prokaryote
v04de	26803	36	30	0	Medium-quality	70.13	0	Siphoviridae	prokaryote
v04df	34347	56	8	1	Medium-quality	59.11	0	unclassified	unclassified
v04e0	24574	35	7	1	Medium-quality	76.38	0	Siphoviridae	prokaryote
v04e1	21811	21	17	0	Medium-quality	51.55	0	Siphoviridae	prokaryote
v04e2	42857	52	30	0	High-quality	99.68	0	Autographiviridae	prokaryote
v04e3	15579	27	5	0	Medium-quality	50.26	0	Siphoviridae	prokaryote
v04e4	15596	27	6	1	Medium-quality	51.91	0	Siphoviridae	prokaryote
v04e5	22730	27	20	0	Medium-quality	69.22	0	Siphoviridae	prokaryote
v04e6	35994	54	17	0	Complete	100	0	Siphoviridae	prokaryote
v04e7	27723	32	8	0	Medium-quality	81.24	0	unclassified	unclassified
v04e8	82905	130	15	1	Medium-quality	85.39	0	Podoviridae_crAss-like	prokaryote
v04e9	38908	56	33	0	Medium-quality	85.39	0	Siphoviridae	prokaryote
v04ea	48531	59	24	0	Complete	100	0	Siphoviridae	prokaryote
v04eb	40759	60	32	0	High-quality	97.21	0	Myoviridae	prokaryote
v04ec	38588	63	49	0	Complete	100	0	Podoviridae	prokaryote
v04ed	4581	5	3	0	High-quality	97.82	0	Microviridae	prokaryote
v04ee	140581	192	68	1	Complete	100	0	Herelleviridae	prokaryote
v04ef	38240	55	43	0	Complete	100	0	Siphoviridae	prokaryote
v04f0	6910	7	4	0	Complete	100	0	unclassified	unclassified
v04f1	89361	91	2	37	High-quality	100	0	unclassified	unclassified
v04f2	30893	38	3	1	Medium-quality	63.89	0	unclassified	unclassified
v04f3	64531	90	21	1	High-quality	100	0	Myoviridae	prokaryote
v04f4	5828	8	2	0	High-quality	100	0	unclassified	unclassified
v04f5	28977	36	13	0	Medium-quality	58.27	0	Siphoviridae	prokaryote
v04f6	42690	56	19	1	Complete	100	0	Siphoviridae	prokaryote
v04f7	40131	51	26	0	Medium-quality	66.58	0	Siphoviridae	prokaryote
v04f8	6185	6	3	0	High-quality	90.58	0	unclassified	unclassified
v04f9	46150	72	26	0	High-quality	94.59	0	unclassified	unclassified
v04fa	38889	59	42	0	Medium-quality	71.99	0	Myoviridae	prokaryote
v04fb	9947	12	4	0	Complete	100	0	Podoviridae	prokaryote

v04fc	67433	81	16	0	Complete	100	0	unclassified	unclassified
v04fd	5893	9	4	0	Complete	100	0	Microviridae	prokaryote
v04fe	29951	40	1	11	Medium-quality	53.72	0	Myoviridae	prokaryote
v04ff	21522	25	16	0	Medium-quality	79.91	0	Siphoviridae	prokaryote
v0500	38049	49	32	1	Complete	100	0	Myoviridae	prokaryote
v0501	41305	65	30	0	Complete	100	0	Siphoviridae	prokaryote
v0502	6318	8	3	0	Complete	100	0	unclassified	unclassified
v0503	11149	13	3	2	High-quality	93.34	0	unclassified	unclassified
v0504	16277	21	7	0	Medium-quality	51.09	0	unclassified	unclassified
v0505	6376	8	3	0	Complete	100	0	unclassified	unclassified
v0506	38043	55	21	1	Complete	100	0	Siphoviridae	prokaryote
v0507	7159	7	3	0	Medium-quality	52.46	0	unclassified	unclassified
v0508	2998	4	3	0	Medium-quality	62.47	0	Microviridae	prokaryote
v0509	12359	15	5	1	High-quality	90.6	0	unclassified	unclassified
v050a	3146	3	0	2	Medium-quality	55.89	0	unclassified	unclassified
v050b	6257	10	2	0	Complete	100	0	unclassified	unclassified
v050c	2473	4	0	0	High-quality	100	0	unclassified	unclassified
v050d	42660	78	15	0	Complete	100	0	unclassified	unclassified
v050e	5236	8	7	0	Complete	100	0	Microviridae	prokaryote
v050f	6728	8	3	0	Complete	100	0	unclassified	unclassified
v0510	5438	9	6	0	High-quality	100	0	Microviridae	prokaryote
v0511	16137	22	8	0	Medium-quality	84.01	0	unclassified	unclassified
v0512	6148	10	3	0	Complete	100	0	unclassified	unclassified
v0513	53088	75	12	1	Medium-quality	69.69	0	unclassified	unclassified
v0514	4516	4	1	0	Medium-quality	69.02	0	unclassified	unclassified
v0515	4040	5	2	0	Medium-quality	66.73	0	unclassified	unclassified
v0516	14057	20	8	0	High-quality	100	0	unclassified	unclassified
v0517	12004	17	3	0	Complete	100	0	unclassified	unclassified
v0518	14986	16	4	0	Medium-quality	50.26	0	unclassified	unclassified
v0519	6196	7	4	0	Complete	100	0	unclassified	unclassified
v051a	16432	22	7	1	Medium-quality	70.96	0	unclassified	unclassified
v051b	5380	9	7	0	High-quality	100	0	Microviridae	prokaryote
v051c	17772	22	6	2	Medium-quality	59.81	0	unclassified	unclassified
v051d	5617	6	3	0	Complete	100	0	unclassified	unclassified
v051e	4962	7	7	0	Complete	100	0	Microviridae	prokaryote
v051f	15942	21	6	0	High-quality	95.38	0	unclassified	unclassified
v0520	3343	10	1	0	Medium-quality	57.47	0	Inoviridae	prokaryote
v0521	6306	9	3	0	Complete	100	0	unclassified	unclassified
v0522	3012	5	2	0	Medium-quality	51.39	0	Microviridae	prokaryote
v0523	5666	8	2	0	Complete	100	0	unclassified	unclassified

v0524	3278	7	2	0	Medium-quality	52.8	0	unclassified	unclassified
v0525	5941	6	2	0	Complete	100	0	unclassified	unclassified
v0526	5374	9	8	0	Complete	100	0	Microviridae	prokaryote
v0527	21027	26	1	13	High-quality	100	0	unclassified	unclassified
v0528	5230	8	6	0	Complete	100	0	Microviridae	prokaryote
v0529	13070	15	5	0	High-quality	100	0	unclassified	unclassified
v052a	5125	9	6	0	Complete	100	0	Microviridae	prokaryote
v052b	7295	7	4	0	Complete	100	0	unclassified	unclassified
v052c	14744	21	4	1	High-quality	100	0	unclassified	unclassified
v052d	50324	63	16	0	Complete	100	0	Siphoviridae	prokaryote
v052e	3284	5	4	0	Medium-quality	68.5	0	Microviridae	prokaryote
v052f	4099	3	2	0	Medium-quality	61.71	0	unclassified	unclassified
v0530	2923	4	1	0	Medium-quality	51.32	0	unclassified	unclassified
v0531	26401	39	1	19	Medium-quality	52.24	0	unclassified	unclassified
v0532	84159	129	28	6	High-quality	100	0	unclassified	unclassified
v0533	43523	48	28	0	Complete	100	0	Autographiviridae	prokaryote
v0534	6551	10	3	0	Complete	100	0	unclassified	unclassified
v0535	29774	50	12	1	Medium-quality	80.72	0	unclassified	unclassified
v0536	6085	11	2	0	Complete	100	0	unclassified	unclassified
v0537	39251	54	8	3	High-quality	100	0	unclassified	unclassified
v0538	20613	20	4	0	Medium-quality	52.23	0	unclassified	unclassified
v0539	6685	9	3	0	Complete	100	0	unclassified	unclassified
v053a	37093	59	18	0	High-quality	93.16	0	Siphoviridae	prokaryote
v053b	22712	35	16	2	Medium-quality	64.38	0	unclassified	unclassified
v053c	5639	8	3	0	Complete	100	0	unclassified	unclassified
v053d	5754	9	6	0	Complete	100	0	Microviridae	prokaryote
v053e	3748	2	2	0	High-quality	100	0	Anelloviridae	eukaryote
v053f	40802	54	41	0	Complete	100	0	Podoviridae	prokaryote
v0540	44307	73	25	2	Complete	100	0	unclassified	unclassified
v0541	5139	6	6	0	Complete	100	0	Microviridae	prokaryote
v0542	9197	14	1	0	Complete	100	0	unclassified	unclassified
v0543	13400	20	5	0	High-quality	98.28	0	unclassified	unclassified
v0544	13438	19	5	0	Complete	100	0	unclassified	unclassified
v0545	33668	27	1	13	Medium-quality	72.42	0	unclassified	unclassified
v0546	20199	18	2	0	Medium-quality	58.03	0	unclassified	unclassified
v0547	35906	68	18	1	Medium-quality	82.96	0	unclassified	unclassified
v0548	9528	8	0	1	Medium-quality	50.82	0	unclassified	unclassified
v0549	6217	12	0	0	Complete	100	0	unclassified	unclassified
v054a	5258	8	5	0	Complete	100	0	Microviridae	prokaryote
v054b	3604	5	1	0	Medium-quality	60.76	0	Microviridae	prokaryote

v054c	14983	22	11	0	High-quality	100	0	Siphoviridae	prokaryote
v054d	30828	41	24	0	Medium-quality	81.14	0	Siphoviridae	prokaryote
v054e	5122	9	7	0	Complete	100	0	Microviridae	prokaryote
v054f	40425	58	37	0	Complete	100	0	Siphoviridae	prokaryote
v0550	6082	7	3	0	High-quality	99.68	0	unclassified	unclassified
v0551	20715	23	11	0	Medium-quality	50.19	0	unclassified	unclassified
v0552	3298	2	1	0	High-quality	100	0	Circoviridae	eukaryote
v0553	30418	38	4	1	Medium-quality	62.92	0	unclassified	unclassified
v0554	19222	21	19	0	Medium-quality	50.05	0	Siphoviridae	prokaryote
v0555	38596	64	37	0	High-quality	98.84	0	Siphoviridae	prokaryote
v0556	33115	54	18	0	High-quality	100	0	Siphoviridae	prokaryote
v0557	37304	58	22	0	Complete	100	0	unclassified	unclassified
v0558	2880	2	1	0	Medium-quality	77.79	0	Anelloviridae	eukaryote
v0559	15316	27	4	0	Medium-quality	56.56	0	unclassified	unclassified
v055a	5836	8	4	0	High-quality	95.67	0	unclassified	unclassified
v055b	16834	20	6	0	Medium-quality	51.68	0	unclassified	unclassified
v055c	10285	13	4	0	Medium-quality	87.24	0	Podoviridae	prokaryote
v055d	81423	122	57	1	Medium-quality	79.73	0	Myoviridae	prokaryote
v055e	7059	9	3	0	Medium-quality	59.64	0	Podoviridae	prokaryote
v055f	75938	107	51	0	Complete	100	0	Schitoviridae	prokaryote
v0560	48691	78	60	0	Complete	100	0	Drexlerviridae	prokaryote
v0561	44503	63	16	1	High-quality	99.04	0	unclassified	unclassified
v0562	34648	36	24	0	Medium-quality	74.61	0	Autographiviridae	prokaryote
v0563	24177	31	26	0	Medium-quality	63.28	0	Siphoviridae	prokaryote
v0564	66131	68	1	31	High-quality	100	0	unclassified	unclassified
v0565	27605	28	27	0	Medium-quality	68.23	0	Autographiviridae	prokaryote
v0566	42463	64	8	1	Complete	100	0	unclassified	unclassified
v0567	22467	25	9	0	Medium-quality	56.24	0	unclassified	unclassified
v0568	6036	9	2	0	Complete	100	0	unclassified	unclassified
v0569	6831	8	4	0	High-quality	100	0	unclassified	unclassified
v056a	20792	29	8	2	Medium-quality	56.56	0	Siphoviridae	prokaryote
v056b	2498	3	2	0	Medium-quality	70.95	0	Anelloviridae	eukaryote
v056c	36098	48	25	0	Medium-quality	88.95	0	Myoviridae	prokaryote
v056d	5646	6	3	0	Complete	100	0	unclassified	unclassified
v056e	6352	10	4	0	High-quality	100	0	Microviridae	prokaryote
v056f	15576	20	9	0	Medium-quality	67.4	0	Salasmaviridae	prokaryote
v0570	10115	20	2	0	Medium-quality	76.16	0	unclassified	unclassified
v0571	6518	9	3	0	Complete	100	0	Microviridae	prokaryote
v0572	6145	9	4	0	Complete	100	0	unclassified	unclassified
v0573	6245	8	3	0	Complete	100	0	unclassified	unclassified

v0574	45321	72	18	1	Complete	100	0	unclassified	unclassified
v0575	5801	9	4	0	Complete	100	0	Microviridae	prokaryote
v0576	34525	50	15	0	Complete	100	0	unclassified	unclassified
v0577	30891	45	26	0	Medium-quality	81.45	0	Siphoviridae	prokaryote
v0578	18763	26	9	0	High-quality	96.02	0	Salasmaviridae	prokaryote
v0579	42741	53	30	0	Medium-quality	71.01	0	Siphoviridae	prokaryote
v057a	34952	51	17	0	High-quality	100	0	unclassified	unclassified
v057b	66461	71	2	30	Medium-quality	69.06	0	unclassified	unclassified
v057c	6191	10	2	0	Complete	100	0	unclassified	unclassified
v057d	36435	47	11	1	Complete	100	0	unclassified	unclassified
v057e	5230	7	2	0	Complete	100	0	unclassified	unclassified
v057f	41162	58	26	0	Medium-quality	68.34	0	Siphoviridae	prokaryote
v0580	6591	7	2	0	High-quality	98.14	0	unclassified	unclassified
v0581	4224	7	3	0	Medium-quality	71.12	0	unclassified	unclassified
v0582	53888	35	2	0	Medium-quality	53.64	0	Podoviridae_crAss-like	prokaryote
v0583	21367	23	7	0	Medium-quality	54.79	0	Siphoviridae	prokaryote
v0584	2918	1	1	0	High-quality	98.09	0	Anelloviridae	eukaryote
v0585	48065	71	40	1	Complete	100	0	Siphoviridae	prokaryote
v0586	6572	6	4	0	Complete	100	0	unclassified	unclassified
v0587	6837	8	2	0	Complete	100	0	unclassified	unclassified
v0588	5822	8	3	0	Complete	100	0	unclassified	unclassified
v0589	6050	7	2	0	Complete	100	0	unclassified	unclassified
v058a	16809	19	7	0	Complete	100	0	Salasmaviridae	prokaryote
v058b	6381	8	4	0	Complete	100	0	unclassified	unclassified
v058c	40604	63	44	0	Complete	100	0	Podoviridae	prokaryote
v058d	57069	76	51	0	High-quality	97.1	0	Siphoviridae	prokaryote
v058e	6123	7	4	0	Complete	100	0	unclassified	unclassified
v058f	6385	8	3	0	Complete	100	0	unclassified	unclassified
v0590	35082	50	11	0	Medium-quality	77.27	0	Siphoviridae	prokaryote
v0591	41997	53	36	0	Complete	100	0	Podoviridae	prokaryote
v0592	39362	64	14	0	Complete	100	0	Quimbyviridae	prokaryote
v0593	6178	10	2	0	Complete	100	0	unclassified	unclassified
v0594	92821	91	9	15	Complete	100	0	unclassified	unclassified
v0595	3589	2	2	0	Complete	100	0	unclassified	unclassified
v0596	2483	1	1	0	Medium-quality	67.06	0	Anelloviridae	eukaryote
v0597	42067	61	22	0	Complete	100	0	unclassified	unclassified
v0598	2906	2	1	0	Complete	100	0	Anelloviridae	eukaryote
v0599	40387	49	19	0	High-quality	98.13	0	Siphoviridae	prokaryote
v059a	3085	3	2	0	Complete	100	0	Anelloviridae	eukaryote
v059b	3089	3	2	0	Medium-quality	87.43	0	Anelloviridae	eukaryote

v059c	34041	46	11	0	Complete	100	0	unclassified	unclassified
v059d	38641	57	6	0	High-quality	100	0	unclassified	unclassified
v059e	40030	60	42	0	High-quality	100	0	Podoviridae	prokaryote
v059f	43228	66	15	0	Complete	100	0	unclassified	unclassified
v05a0	3413	2	2	0	High-quality	98.33	0	unclassified	unclassified
v05a1	2333	1	1	0	Medium-quality	65.38	0	unclassified	unclassified
v05a2	3432	3	2	0	Complete	100	0	Anelloviridae	eukaryote
v05a3	2561	2	2	0	Medium-quality	72.57	0	Anelloviridae	eukaryote
v05a4	2419	1	1	0	Medium-quality	82.88	0	Anelloviridae	eukaryote
v05a5	2980	2	1	0	Medium-quality	81.25	0	Anelloviridae	eukaryote
v05a6	3091	3	2	0	Medium-quality	87.4	0	Anelloviridae	eukaryote
v05a7	3156	3	1	0	Medium-quality	85.26	0	Anelloviridae	eukaryote
v05a8	2988	4	2	0	Medium-quality	85.95	0	Anelloviridae	eukaryote
v05a9	41213	55	20	0	High-quality	99.82	0	unclassified	unclassified
v05aa	39821	56	20	0	High-quality	100	0	Siphoviridae	prokaryote
v05ab	2641	2	1	0	Medium-quality	74.78	0	Anelloviridae	eukaryote
v05ac	6583	12	2	0	High-quality	100	0	unclassified	unclassified
v05ad	6149	9	4	0	Complete	100	0	unclassified	unclassified
v05ae	13389	19	4	0	High-quality	97.69	0	unclassified	unclassified
v05af	6284	7	4	0	High-quality	100	0	unclassified	unclassified
v05b0	2873	6	0	0	Medium-quality	54.05	0	unclassified	unclassified
v05b1	6381	7	4	0	Complete	100	0	unclassified	unclassified
v05b2	5620	11	5	0	Complete	100	0	unclassified	unclassified
v05b3	83928	137	13	2	Medium-quality	54.52	0	unclassified	unclassified
v05b4	196881	234	31	8	High-quality	100	0	unclassified	unclassified
v05b5	11619	18	5	0	Medium-quality	83.66	0	unclassified	unclassified
v05b6	6099	9	3	0	Complete	100	0	unclassified	unclassified
v05b7	50737	75	32	0	High-quality	100	0	Myoviridae	prokaryote
v05b8	30533	27	1	12	High-quality	100	0	unclassified	unclassified
v05b9	5913	9	3	0	High-quality	93.6	0	unclassified	unclassified
v05ba	24164	44	6	0	Medium-quality	55.31	0	unclassified	unclassified
v05bb	39133	52	26	0	Medium-quality	64.97	0	Siphoviridae	prokaryote
v05bc	161918	164	76	0	Medium-quality	57.99	0	Myoviridae	prokaryote
v05bd	15744	23	6	0	Medium-quality	67.48	0	unclassified	unclassified
v05be	16060	21	7	0	Medium-quality	67.89	0	unclassified	unclassified
v05bf	5218	9	7	0	Complete	100	0	Microviridae	prokaryote
v05c0	7056	6	3	0	High-quality	100	0	unclassified	unclassified
v05c1	16792	21	7	0	High-quality	98.6	0	unclassified	unclassified
v05c2	3356	4	3	0	Complete	100	0	Hepadnaviridae	eukaryote
v05c3	5805	13	2	0	High-quality	96.99	0	unclassified	unclassified

v05c4	3910	8	2	0	Medium-quality	52.08	0	Siphoviridae	prokaryote
v05c5	3649	5	2	0	Medium-quality	64.81	0	Microviridae	prokaryote
v05c6	6133	8	3	0	Complete	100	0	unclassified	unclassified
v05c7	2311	4	4	0	Complete	100	0	Genomoviridae	eukaryote
v05c8	46429	63	27	0	Complete	100	0	Siphoviridae	prokaryote
v05c9	3193	3	2	0	Medium-quality	50.05	0	Microviridae	prokaryote
v05ca	14884	22	11	0	High-quality	100	0	Siphoviridae	prokaryote
v05cb	2361	1	1	0	Medium-quality	68.79	0	Anelloviridae	eukaryote
v05cc	53634	74	28	1	High-quality	100	0	unclassified	unclassified
v05cd	2424	1	1	0	Medium-quality	67.93	0	Anelloviridae	eukaryote
v05ce	41028	48	32	0	High-quality	95.93	0	Autographiviridae	prokaryote
v05cf	3004	3	2	0	Medium-quality	86.34	0	Anelloviridae	eukaryote
v05d0	3032	2	2	0	Medium-quality	89.43	0	Anelloviridae	eukaryote
v05d1	53740	65	41	1	Complete	100	0	Siphoviridae	prokaryote
v05d2	9239	14	5	0	Medium-quality	57.48	0	Siphoviridae	prokaryote
v05d3	22755	27	21	0	Medium-quality	58.4	0	Siphoviridae	prokaryote
v05d4	20143	23	8	0	Medium-quality	52.65	0	Siphoviridae	prokaryote
v05d5	3097	3	1	0	Medium-quality	87.57	0	Anelloviridae	eukaryote
v05d6	2651	1	1	0	Medium-quality	74.04	0	Anelloviridae	eukaryote
v05d7	2929	2	2	0	Medium-quality	78.79	0	Anelloviridae	eukaryote
v05d8	196784	224	74	0	Medium-quality	69.57	0	Myoviridae	prokaryote
v05d9	41446	52	28	0	Medium-quality	68.77	0	Siphoviridae	prokaryote
v05da	36911	33	15	0	Medium-quality	61.74	0	unclassified	unclassified
v05db	41882	57	34	1	Complete	100	0	Myoviridae	prokaryote
v05dc	40507	48	37	0	Complete	100	0	Podoviridae	prokaryote
v05dd	3474	3	2	0	High-quality	100	0	Anelloviridae	eukaryote
v05de	3139	2	2	0	Complete	100	0	Anelloviridae	eukaryote
v05df	33605	39	27	0	Complete	100	0	Siphoviridae	prokaryote
v05e0	6870	7	4	0	Complete	100	0	unclassified	unclassified
v05e1	24438	30	19	0	Medium-quality	59.57	0	Podoviridae	prokaryote
v05e2	46844	65	21	0	High-quality	94.2	0	unclassified	unclassified
v05e3	22558	22	3	0	Medium-quality	66.95	0	unclassified	unclassified
v05e4	42192	46	19	0	Complete	100	0	unclassified	unclassified
v05e5	2868	1	1	0	Medium-quality	77.49	0	Anelloviridae	eukaryote
v05e6	18305	26	12	0	High-quality	99.68	0	Rountreeviridae	prokaryote
v05e7	31548	49	8	0	Complete	100	0	unclassified	unclassified
v05e8	215962	316	256	0	Complete	100	0	Demerecviridae	prokaryote
v05e9	15289	19	5	0	Medium-quality	81.9	0	unclassified	unclassified
v05ea	45667	60	11	1	High-quality	92.24	0	unclassified	unclassified
v05eb	41348	59	24	0	High-quality	91.85	0	Siphoviridae	prokaryote

v05ec	30653	52	25	0	Medium-quality	88.61	0	Siphoviridae	prokaryote
v05ed	15062	16	5	1	Medium-quality	52.75	0	unclassified	unclassified
v05ee	30553	37	4	1	Medium-quality	63.19	0	unclassified	unclassified
v05ef	38883	50	12	2	High-quality	100	0	unclassified	unclassified
v05f0	22913	28	7	0	Medium-quality	76.27	0	unclassified	unclassified
v05f1	55737	95	21	2	High-quality	98.06	0	unclassified	unclassified
v05f2	101755	104	13	1	High-quality	100	0	Podoviridae_crAss-like	prokaryote
v05f3	5522	10	7	0	High-quality	100	0	Microviridae	prokaryote
v05f4	3249	6	2	0	Medium-quality	56.49	0	unclassified	unclassified
v05f5	12757	17	5	0	Complete	100	0	unclassified	unclassified
v05f6	28738	36	3	6	High-quality	100	0	unclassified	unclassified
v05f7	2362	3	2	0	High-quality	100	0	Circoviridae	eukaryote
v05f8	13246	16	4	0	High-quality	99.15	0	unclassified	unclassified
v05f9	6622	9	4	0	Complete	100	0	Microviridae	prokaryote
v05fa	5121	9	7	0	Complete	100	0	Microviridae	prokaryote
v05fb	4971	6	5	0	High-quality	100	0	Microviridae	prokaryote
v05fc	5965	10	6	0	High-quality	100	0	Microviridae	prokaryote
v05fd	10302	8	1	1	Medium-quality	74.17	0	unclassified	unclassified
v05fe	36031	59	24	0	High-quality	100	0	Myoviridae	prokaryote
v05ff	6587	8	4	0	High-quality	100	0	unclassified	unclassified
v0600	6938	8	3	0	High-quality	100	0	unclassified	unclassified
v0601	18943	26	7	0	High-quality	100	0	Podoviridae	prokaryote
v0602	54972	100	19	2	Complete	100	0	unclassified	unclassified
v0603	3697	4	2	0	Medium-quality	61.83	0	unclassified	unclassified
v0604	53062	92	11	2	Medium-quality	65.46	0	Quimbyviridae	prokaryote
v0605	158671	207	17	5	Complete	100	0	unclassified	unclassified
v0606	6678	11	5	1	High-quality	100	0	Microviridae	prokaryote
v0607	2845	2	1	0	Medium-quality	59.17	0	unclassified	unclassified
v0608	6147	8	3	0	Complete	100	0	unclassified	unclassified
v0609	59301	74	17	1	High-quality	100	0	Siphoviridae	prokaryote
v060a	7797	9	4	0	High-quality	100	0	unclassified	unclassified
v060b	77125	75	16	0	Medium-quality	75.69	0	Podoviridae_crAss-like	prokaryote
v060c	58417	91	36	1	Complete	100	0	Siphoviridae	prokaryote
v060d	2146	2	2	0	Medium-quality	69.52	0	unclassified	unclassified
v060e	6093	9	2	0	Complete	100	0	unclassified	unclassified
v060f	3532	4	1	0	Medium-quality	65.13	0	unclassified	unclassified
v0610	2581	2	2	0	Medium-quality	75.02	0	Anelloviridae	eukaryote
v0611	49148	91	21	1	Complete	100	0	unclassified	unclassified
v0612	2186	2	2	0	Medium-quality	65.36	0	Anelloviridae	eukaryote
v0613	6189	6	3	0	Complete	100	0	unclassified	unclassified

v0614	2438	2	2	0	Medium-quality	85.5	0	Anelloviridae	eukaryote
v0615	2111	3	1	0	Medium-quality	59.87	0	Anelloviridae	eukaryote
v0616	3598	1	1	0	High-quality	100	0	unclassified	unclassified
v0617	3336	3	2	0	High-quality	95.12	0	Anelloviridae	eukaryote
v0618	6062	10	5	0	High-quality	100	0	Microviridae	prokaryote
v0619	4359	7	3	0	Medium-quality	80.41	0	unclassified	unclassified
v061a	42489	49	25	0	High-quality	98.66	0	Autographiviridae	prokaryote
v061b	5671	11	5	0	Complete	100	0	unclassified	unclassified
v061c	20869	34	7	0	High-quality	90.12	0	unclassified	unclassified
v061d	2668	1	1	0	Medium-quality	82.66	0	Anelloviridae	eukaryote
v061e	2610	1	1	0	Medium-quality	74.14	0	Anelloviridae	eukaryote
v061f	3545	3	1	0	High-quality	99.41	0	Myoviridae	prokaryote
v0620	31044	44	14	0	Medium-quality	56.91	0	unclassified	unclassified
v0621	37766	50	13	2	High-quality	100	0	Myoviridae	prokaryote
v0622	33772	56	17	1	Medium-quality	61.98	0	unclassified	unclassified
v0623	18142	23	9	0	Medium-quality	82.35	0	unclassified	unclassified
v0624	33799	61	17	0	High-quality	100	0	unclassified	unclassified
v0625	3336	4	2	0	High-quality	98.95	0	Anelloviridae	eukaryote
v0626	2064	1	1	0	Medium-quality	74.28	0	Anelloviridae	eukaryote
v0627	3305	2	2	0	High-quality	92.64	0	Anelloviridae	eukaryote
v0628	2992	3	2	0	Medium-quality	86.4	0	Anelloviridae	eukaryote
v0629	2910	2	2	0	High-quality	90.39	0	Anelloviridae	eukaryote
v062a	2763	3	2	0	High-quality	96.63	0	Anelloviridae	eukaryote
v062b	2407	2	2	0	Medium-quality	75.25	0	Anelloviridae	eukaryote
v062c	2266	2	2	0	Medium-quality	71.18	0	Anelloviridae	eukaryote
v062d	2387	2	1	0	Medium-quality	57.36	0	Circoviridae	eukaryote
v062e	33043	44	20	1	Medium-quality	77.4	0	Siphoviridae	prokaryote
v062f	5944	7	4	0	High-quality	100	0	unclassified	unclassified
v0630	14932	18	6	0	Medium-quality	50.26	0	unclassified	unclassified
v0631	37788	45	10	2	High-quality	95.74	0	unclassified	unclassified
v0632	5311	9	7	0	Complete	100	0	Microviridae	prokaryote
v0633	12248	11	6	0	High-quality	100	0	unclassified	unclassified
v0634	2585	3	3	0	Medium-quality	79.59	0	Anelloviridae	eukaryote
v0635	43894	56	32	0	High-quality	100	0	Siphoviridae	prokaryote
v0636	28231	54	18	0	High-quality	100	0	Siphoviridae	prokaryote
v0637	30794	46	11	0	Complete	100	0	unclassified	unclassified
v0638	2487	2	2	0	Medium-quality	77.68	0	Anelloviridae	eukaryote
v0639	2103	2	1	0	Medium-quality	66.34	0	Anelloviridae	eukaryote
v063a	2735	2	2	0	Medium-quality	78.36	0	Anelloviridae	eukaryote
v063b	3906	5	1	0	Medium-quality	58.16	0	unclassified	unclassified

v063c	4833	7	5	0	Complete	100	0	Microviridae	prokaryote
v063d	6395	8	3	0	Complete	100	0	unclassified	unclassified
v063e	2333	2	2	0	Medium-quality	72.83	0	unclassified	unclassified
v063f	2482	2	2	0	Medium-quality	77.51	0	Anelloviridae	eukaryote
v0640	6585	9	4	0	Complete	100	0	Microviridae	prokaryote
v0641	60609	78	15	0	Complete	100	0	unclassified	unclassified
v0642	5379	10	2	0	Medium-quality	89.93	0	unclassified	unclassified
v0643	5652	10	0	0	High-quality	96.82	0	unclassified	unclassified
v0644	22918	34	5	1	Medium-quality	50.73	0	Siphoviridae	prokaryote
v0645	6155	11	6	0	High-quality	100	0	Microviridae	prokaryote
v0646	6070	9	3	0	Complete	100	0	unclassified	unclassified
v0647	19473	23	14	0	Medium-quality	56.18	0	Myoviridae	prokaryote
v0648	16162	23	7	0	Medium-quality	68.63	0	unclassified	unclassified
v0649	2332	1	1	0	Medium-quality	65.84	0	Anelloviridae	eukaryote
v064a	12847	17	4	0	Complete	100	0	unclassified	unclassified
v064b	6133	7	2	0	Complete	100	0	unclassified	unclassified
v064c	41145	68	17	0	Complete	100	0	unclassified	unclassified
v064d	5220	8	7	0	Complete	100	0	Microviridae	prokaryote
v064e	3092	4	2	0	Medium-quality	53.19	0	unclassified	unclassified
v064f	4763	8	3	0	Medium-quality	84.9	0	unclassified	unclassified
v0650	4166	8	3	0	High-quality	94.79	0	Microviridae	prokaryote
v0651	2955	5	2	0	High-quality	98.21	0	Circoviridae	eukaryote
v0652	5992	10	5	0	Complete	100	0	Microviridae	prokaryote
v0653	44568	73	47	0	Complete	100	0	Siphoviridae	prokaryote
v0654	61010	93	36	0	High-quality	99.8	0	Siphoviridae	prokaryote
v0655	2788	7	0	0	Medium-quality	52.46	0	unclassified	unclassified
v0656	26689	49	16	0	Medium-quality	81	0	Siphoviridae	prokaryote
v0657	5071	7	4	0	Complete	100	0	Microviridae	prokaryote
v0658	43509	75	53	0	Complete	100	0	Siphoviridae	prokaryote
v0659	59566	100	21	1	High-quality	99.11	0	unclassified	unclassified
v065a	59738	63	1	20	High-quality	100	0	unclassified	unclassified
v065b	34529	54	14	0	High-quality	99.17	0	Siphoviridae	prokaryote
v065c	41309	50	48	0	Complete	100	0	Autographiviridae	prokaryote
v065d	70319	67	1	26	High-quality	100	0	unclassified	unclassified
v065e	54231	58	1	14	Medium-quality	79.02	0	unclassified	unclassified
v065f	44446	71	42	0	Complete	100	0	Siphoviridae	prokaryote
v0660	2889	2	2	0	Medium-quality	89.31	0	unclassified	unclassified
v0661	2414	1	1	0	Medium-quality	65.95	0	Anelloviridae	eukaryote
v0662	2736	3	3	0	Medium-quality	70.12	0	Anelloviridae	eukaryote
v0663	2500	2	1	0	Medium-quality	87.97	0	Anelloviridae	eukaryote

v0664	2500	3	2	0	Medium-quality	88.88	0	Anelloviridae	eukaryote
v0665	2370	3	3	0	Medium-quality	82.8	0	Anelloviridae	eukaryote
v0666	41525	56	18	0	High-quality	100	0	unclassified	unclassified
v0667	40720	51	6	0	Medium-quality	65.38	0	unclassified	unclassified
v0668	38227	61	31	0	Complete	100	0	Siphoviridae	prokaryote
v0669	2547	1	1	0	High-quality	90.49	0	Anelloviridae	eukaryote
v066a	2834	3	3	0	High-quality	98	0	Anelloviridae	eukaryote
v066b	2630	1	1	0	High-quality	93.12	0	Anelloviridae	eukaryote
v066c	47110	68	35	1	Complete	100	0	Siphoviridae	prokaryote
v066d	2711	1	1	0	Medium-quality	86.02	0	unclassified	unclassified
v066e	18009	20	6	0	Medium-quality	60.62	0	unclassified	unclassified
v066f	44810	66	34	1	High-quality	100	0	Siphoviridae	prokaryote
v0670	22276	30	13	0	Medium-quality	70.02	0	unclassified	unclassified
v0671	3166	2	2	0	Medium-quality	89.12	0	unclassified	unclassified
v0672	2526	3	3	0	Medium-quality	84.91	0	Anelloviridae	eukaryote
v0673	43320	82	40	0	Complete	100	0	Myoviridae	prokaryote
v0674	2330	2	2	0	Medium-quality	65.79	0	Anelloviridae	eukaryote
v0675	2570	2	2	0	Medium-quality	71.28	0	Anelloviridae	eukaryote
v0676	2752	4	3	0	Medium-quality	70.53	0	Anelloviridae	eukaryote
v0677	6186	11	3	0	Complete	100	0	unclassified	unclassified
v0678	3074	3	2	0	Medium-quality	83.59	0	Anelloviridae	eukaryote
v0679	2741	4	2	0	High-quality	97.99	0	Anelloviridae	eukaryote
v067a	2736	1	1	0	High-quality	95.65	0	Anelloviridae	eukaryote
v067b	2068	1	1	0	Medium-quality	71	0	Anelloviridae	eukaryote
v067c	2668	3	2	0	Medium-quality	87.93	0	Anelloviridae	eukaryote
v067d	2287	2	1	0	Medium-quality	77.74	0	Anelloviridae	eukaryote
v067e	3236	2	2	0	High-quality	91.72	0	Anelloviridae	eukaryote
v067f	34020	47	12	0	Complete	100	0	unclassified	unclassified
v0680	42117	47	19	0	Complete	100	0	unclassified	unclassified
v0681	45101	58	46	0	High-quality	100	0	Siphoviridae	prokaryote
v0682	2623	2	2	0	Medium-quality	81.95	0	Anelloviridae	eukaryote
v0683	38411	51	27	0	High-quality	93.97	0	Siphoviridae	prokaryote
v0684	18701	24	10	0	Medium-quality	63.59	0	Siphoviridae	prokaryote
v0685	2738	2	1	0	Medium-quality	71.45	0	Anelloviridae	eukaryote
v0686	41989	57	27	1	High-quality	100	0	Myoviridae	prokaryote
v0687	2447	2	1	0	Medium-quality	69.88	0	Anelloviridae	eukaryote
v0688	3061	2	2	0	Medium-quality	86.31	0	Anelloviridae	eukaryote
v0689	2717	2	2	0	Medium-quality	82.86	0	unclassified	unclassified
v068a	2563	3	2	0	Medium-quality	72.83	0	Anelloviridae	eukaryote
v068b	2172	3	2	0	Medium-quality	68.1	0	unclassified	unclassified

v068c	2522	1	1	0	High-quality	90.83	0	Anelloviridae	eukaryote
v068d	2510	3	2	0	Medium-quality	78.21	0	unclassified	unclassified
v068e	2022	2	2	0	Medium-quality	61.58	0	Anelloviridae	eukaryote
v068f	3064	2	1	0	Medium-quality	86.85	0	unclassified	unclassified
v0690	2642	3	2	0	High-quality	93.22	0	Anelloviridae	eukaryote
v0691	2527	2	2	0	Medium-quality	71.84	0	Anelloviridae	eukaryote
v0692	2554	2	2	0	Medium-quality	79.82	0	unclassified	unclassified
v0693	2688	2	2	0	Medium-quality	82.29	0	unclassified	unclassified
v0694	2450	3	2	0	Medium-quality	71.38	0	Anelloviridae	eukaryote
v0695	2528	3	3	0	Medium-quality	78.56	0	Anelloviridae	eukaryote
v0696	2058	1	1	0	Medium-quality	62.51	0	unclassified	unclassified
v0697	2595	2	2	0	Medium-quality	80.96	0	unclassified	unclassified
v0698	2235	3	2	0	Medium-quality	66.88	0	Anelloviridae	eukaryote
v0699	2485	2	1	0	Medium-quality	69.52	0	unclassified	unclassified
v069a	2374	1	1	0	Medium-quality	74.08	0	Anelloviridae	eukaryote
v069b	2316	2	2	0	Medium-quality	70.84	0	Anelloviridae	eukaryote
v069c	59694	103	16	2	High-quality	100	0	unclassified	unclassified
v069d	2603	2	2	0	Medium-quality	81.3	0	Anelloviridae	eukaryote
v069e	6306	9	3	0	Complete	100	0	unclassified	unclassified
v069f	2669	2	2	0	Medium-quality	83.05	0	unclassified	unclassified
v06a0	2095	2	2	0	Medium-quality	76.12	0	Anelloviridae	eukaryote
v06a1	2484	2	2	0	Medium-quality	77.3	0	unclassified	unclassified
v06a2	2710	2	2	0	Medium-quality	84.67	0	Anelloviridae	eukaryote
v06a3	2388	1	1	0	Medium-quality	81.07	0	unclassified	unclassified
v06a4	3223	2	2	0	High-quality	90.47	0	Anelloviridae	eukaryote
v06a5	2711	1	1	0	Medium-quality	76.83	0	Anelloviridae	eukaryote
v06a6	158302	193	51	5	High-quality	93.57	0	unclassified	unclassified
v06a7	36639	49	24	0	Medium-quality	60.81	0	Siphoviridae	prokaryote
v06a8	23012	23	4	0	Medium-quality	67.55	0	unclassified	unclassified
v06a9	45416	69	31	2	Complete	100	0	Myoviridae	prokaryote
v06aa	37616	60	9	2	High-quality	100	0	unclassified	unclassified
v06ab	36165	60	24	3	High-quality	100	0	Siphoviridae	prokaryote
v06ac	3839	8	1	0	Medium-quality	64.16	0	unclassified	unclassified
v06ad	29412	42	16	0	High-quality	93.54	0	Siphoviridae	prokaryote
v06ae	21983	22	5	0	Medium-quality	55.33	0	unclassified	unclassified
v06af	3544	4	0	0	Medium-quality	55.21	0	Inoviridae	prokaryote
v06b0	38172	60	17	0	High-quality	100	0	unclassified	unclassified
v06b1	36680	57	17	3	Complete	100	0	Siphoviridae	prokaryote
v06b2	3069	8	0	0	Medium-quality	56.18	0	Inoviridae	prokaryote
v06b3	5620	6	2	0	Complete	100	0	unclassified	unclassified

v06b4	7508	9	4	0	High-quality	100	0	unclassified	unclassified
v06b5	38380	49	15	0	Complete	100	0	Siphoviridae	prokaryote
v06b6	4978	6	6	0	Complete	100	0	Microviridae	prokaryote
v06b7	4976	7	5	0	Complete	100	0	Microviridae	prokaryote
v06b8	6183	9	3	0	Complete	100	0	unclassified	unclassified
v06b9	6164	6	2	0	Complete	100	0	unclassified	unclassified
v06ba	20826	26	22	0	Medium-quality	55.15	0	Siphoviridae	prokaryote
v06bb	25326	30	14	0	Medium-quality	59.45	0	Siphoviridae	prokaryote
v06bc	8063	9	5	0	Medium-quality	50.96	0	unclassified	unclassified
v06bd	6761	8	4	0	High-quality	100	0	unclassified	unclassified
v06be	38688	57	29	0	High-quality	96.58	0	Siphoviridae	prokaryote
v06bf	46424	63	13	0	High-quality	100	0	Siphoviridae	prokaryote
v06c0	29739	44	16	3	Medium-quality	79.88	0	Siphoviridae	prokaryote
v06c1	2823	4	2	0	Medium-quality	52.83	0	Microviridae	prokaryote
v06c2	38628	68	31	0	High-quality	99.7	0	Siphoviridae	prokaryote
v06c3	11306	14	5	0	Medium-quality	58.46	0	Podoviridae	prokaryote
v06c4	18814	23	8	0	Medium-quality	62.32	0	Siphoviridae	prokaryote
v06c5	65792	84	18	0	High-quality	100	0	unclassified	unclassified
v06c6	5810	9	5	0	Complete	100	0	Microviridae	prokaryote
v06c7	15916	22	9	0	Medium-quality	70.2	0	unclassified	unclassified
v06c8	59234	99	34	0	High-quality	100	0	Siphoviridae	prokaryote
v06c9	16944	19	10	0	High-quality	93.24	0	Rountreeviridae	prokaryote
v06ca	37913	54	18	1	Complete	100	0	Siphoviridae	prokaryote
v06cb	5379	8	3	0	Complete	100	0	Microviridae	prokaryote
v06cc	5778	7	1	0	Complete	100	0	unclassified	unclassified
v06cd	23203	43	8	0	Medium-quality	56.62	0	unclassified	unclassified
v06ce	31185	47	24	0	Medium-quality	78.3	0	Siphoviridae	prokaryote
v06cf	21920	26	14	0	Medium-quality	51.22	0	Siphoviridae	prokaryote
v06d0	39285	62	25	0	High-quality	100	0	Siphoviridae	prokaryote
v06d1	18224	26	7	1	Medium-quality	56.72	0	unclassified	unclassified
v06d2	7026	9	3	0	Complete	100	0	unclassified	unclassified
v06d3	5659	8	3	0	High-quality	100	0	unclassified	unclassified
v06d4	3488	4	3	0	Medium-quality	58.6	0	unclassified	unclassified
v06d5	40989	53	28	0	Medium-quality	68.09	0	Siphoviridae	prokaryote
v06d6	44601	64	27	1	High-quality	98.98	0	Siphoviridae	prokaryote
v06d7	28386	60	13	0	Medium-quality	62.91	0	unclassified	unclassified
v06d8	30434	38	4	0	High-quality	96.15	0	unclassified	unclassified
v06d9	2649	2	1	0	Medium-quality	82.47	0	Anelloviridae	eukaryote
v06da	2420	2	1	0	Medium-quality	66.53	0	Anelloviridae	eukaryote
v06db	2584	2	2	0	High-quality	90.31	0	Anelloviridae	eukaryote

v06dc	2263	2	2	0	Medium-quality	70.71	0	unclassified	unclassified
v06dd	2812	2	1	0	Medium-quality	79.96	0	Anelloviridae	eukaryote
v06de	2722	1	1	0	Medium-quality	75.37	0	Anelloviridae	eukaryote
v06df	3031	2	1	0	Medium-quality	86.75	0	Anelloviridae	eukaryote
v06e0	2825	2	2	0	Medium-quality	79.53	0	Anelloviridae	eukaryote
v06e1	3018	2	1	0	Medium-quality	87.07	0	unclassified	unclassified
v06e2	3102	2	2	0	Medium-quality	87	0	Anelloviridae	eukaryote
v06e3	3240	5	2	0	Medium-quality	53.09	0	unclassified	unclassified
v06e4	2231	2	2	0	Medium-quality	64.39	0	Anelloviridae	eukaryote
v06e5	3341	3	3	0	High-quality	100	0	Anelloviridae	eukaryote
v06e6	2562	2	2	0	Medium-quality	70.48	0	unclassified	unclassified
v06e7	2210	1	1	0	Medium-quality	60.23	0	Anelloviridae	eukaryote
v06e8	2625	1	1	0	High-quality	94.51	0	Anelloviridae	eukaryote
v06e9	2133	2	1	0	Medium-quality	58.91	0	Anelloviridae	eukaryote
v06ea	4747	2	2	0	High-quality	100	0	Anelloviridae	eukaryote
v06eb	2894	2	1	0	Complete	100	0	Anelloviridae	eukaryote
v06ec	2593	1	1	0	Medium-quality	72.67	0	unclassified	unclassified
v06ed	2499	2	1	0	Medium-quality	70.67	0	Anelloviridae	eukaryote
v06ee	2588	2	2	0	High-quality	90.12	0	unclassified	unclassified
v06ef	3003	2	2	0	Medium-quality	85.35	0	Anelloviridae	eukaryote
v06f0	2646	4	2	0	Medium-quality	69.05	0	Anelloviridae	eukaryote
v06f1	2808	2	2	0	High-quality	98.51	0	Anelloviridae	eukaryote
v06f2	2801	2	2	0	Medium-quality	73.21	0	unclassified	unclassified
v06f3	3019	3	3	0	Complete	100	0	Anelloviridae	eukaryote
v06f4	38962	49	20	0	High-quality	100	0	Siphoviridae	prokaryote
v06f5	2367	2	2	0	Medium-quality	74.02	0	Anelloviridae	eukaryote
v06f6	2143	1	1	0	Medium-quality	60.81	0	Anelloviridae	eukaryote
v06f7	2340	1	1	0	Medium-quality	66.63	0	Anelloviridae	eukaryote
v06f8	3501	4	1	0	High-quality	98.94	0	Anelloviridae	eukaryote
v06f9	3118	1	1	0	Medium-quality	87.88	0	Anelloviridae	eukaryote
v06fa	41201	56	32	1	High-quality	98.15	0	Siphoviridae	prokaryote
v06fb	29590	39	27	0	Medium-quality	72.86	0	Siphoviridae	prokaryote
v06fc	15088	20	10	0	Medium-quality	50.8	0	Siphoviridae	prokaryote
v06fd	39112	51	16	0	High-quality	97.59	0	unclassified	unclassified
v06fe	33841	58	26	0	Complete	100	0	Siphoviridae	prokaryote
v06ff	37987	60	37	0	Complete	100	0	Siphoviridae	prokaryote
v0700	3005	6	2	0	Medium-quality	58.93	0	unclassified	unclassified
v0701	45056	66	35	0	High-quality	100	0	Siphoviridae	prokaryote
v0702	4872	4	2	0	Medium-quality	84.87	0	unclassified	unclassified
v0703	3197	3	1	0	Complete	100	0	Redondoviridae	eukaryote

v0704	37221	60	27	0	Complete	100	0	Myoviridae	prokaryote
v0705	2388	1	1	0	Medium-quality	68.64	0	unclassified	unclassified
v0706	2228	4	1	0	High-quality	100	0	Genomoviridae	eukaryote
v0707	35002	57	24	0	High-quality	96.76	0	Siphoviridae	prokaryote
v0708	64200	66	9	0	Complete	100	0	unclassified	unclassified
v0709	7258	10	3	0	Complete	100	0	unclassified	unclassified
v070a	11034	14	4	0	High-quality	93.16	0	Podoviridae	prokaryote
v070b	2677	1	1	0	High-quality	93.93	0	Anelloviridae	eukaryote
v070c	18348	20	7	0	Medium-quality	88.93	0	unclassified	unclassified
v070d	57093	82	49	0	Complete	100	0	Siphoviridae	prokaryote
v070e	41890	59	41	0	Complete	100	0	Siphoviridae	prokaryote
v070f	43718	86	44	1	Complete	100	0	Myoviridae	prokaryote
v0710	37303	57	34	0	High-quality	95.01	0	Siphoviridae	prokaryote
v0711	4805	8	3	0	High-quality	94.09	0	Microviridae	prokaryote
v0712	62338	95	18	0	Complete	100	0	unclassified	unclassified
v0713	43099	65	22	1	High-quality	100	0	Siphoviridae	prokaryote
v0714	5072	8	7	0	Complete	100	0	Microviridae	prokaryote
v0715	91877	142	14	3	Complete	100	0	Quimbyviridae	prokaryote
v0716	40835	71	21	0	High-quality	91.75	0	Myoviridae	prokaryote
v0717	2293	4	3	0	High-quality	100	0	Genomoviridae	eukaryote
v0718	17245	20	8	0	High-quality	95.99	0	Podoviridae	prokaryote
v0719	39598	55	37	0	Complete	100	0	Podoviridae	prokaryote
v071a	14319	19	8	0	Medium-quality	73.24	0	Salasmaviridae	prokaryote
v071b	6718	8	3	0	High-quality	100	0	unclassified	unclassified
v071c	51365	82	57	0	Complete	100	0	Siphoviridae	prokaryote
v071d	2346	2	1	0	Medium-quality	65.22	0	Anelloviridae	eukaryote
v071e	38696	60	16	2	High-quality	100	0	Siphoviridae	prokaryote
v071f	26986	26	0	6	Medium-quality	62.21	0	unclassified	unclassified
v0720	33226	50	37	0	Medium-quality	82.21	0	Podoviridae	prokaryote
v0721	48263	80	18	0	High-quality	97.08	0	unclassified	unclassified
v0722	43299	74	25	0	Complete	100	0	Myoviridae	prokaryote
v0723	3417	5	1	0	Medium-quality	58.82	0	Microviridae	prokaryote
v0724	23745	26	12	0	Medium-quality	69.61	0	Siphoviridae	prokaryote
v0725	37973	53	26	1	Medium-quality	54.52	0	Siphoviridae	prokaryote
v0726	45450	75	17	0	Complete	100	0	Siphoviridae	prokaryote
v0727	44487	69	15	0	Complete	100	0	unclassified	unclassified
v0728	6189	4	4	0	High-quality	95.34	0	Virgaviridae	eukaryote
v0729	40357	66	28	1	Complete	100	0	Siphoviridae	prokaryote
v072a	58418	65	1	11	High-quality	100	0	unclassified	unclassified
v072b	112276	158	22	9	Medium-quality	67.01	0	unclassified	unclassified

v072c	6383	13	1	0	Complete	100	0	unclassified	unclassified
v072d	23630	43	17	0	Medium-quality	71.69	0	Siphoviridae	prokaryote
v072e	41469	61	36	0	High-quality	100	0	Siphoviridae	prokaryote
v072f	2249	1	1	0	Medium-quality	64.54	0	Anelloviridae	eukaryote
v0730	37933	59	40	0	High-quality	99.59	0	Siphoviridae	prokaryote
v0731	25462	27	10	0	Medium-quality	63.17	0	unclassified	unclassified
v0732	33420	46	22	0	High-quality	100	0	Siphoviridae	prokaryote
v0733	55244	71	29	0	High-quality	97.53	0	Siphoviridae	prokaryote
v0734	45613	47	13	0	Medium-quality	78.73	0	unclassified	unclassified
v0735	42337	55	11	1	Medium-quality	87.59	0	unclassified	unclassified
v0736	42415	52	29	0	Medium-quality	70.56	0	Siphoviridae	prokaryote
v0737	31689	50	30	0	Medium-quality	89.47	0	Myoviridae	prokaryote
v0738	81722	82	2	33	High-quality	100	0	unclassified	unclassified
v0739	39880	55	29	0	Medium-quality	66.31	0	Siphoviridae	prokaryote
v073a	31371	46	25	0	High-quality	94.02	0	Siphoviridae	prokaryote
v073b	28620	26	14	0	Complete	100	0	unclassified	unclassified
v073c	21361	34	20	0	Complete	100	0	Siphoviridae	prokaryote
v073d	38373	60	44	0	Complete	100	0	Siphoviridae	prokaryote
v073e	40530	60	11	0	Complete	100	0	unclassified	unclassified
v073f	41075	68	14	3	High-quality	100	0	Siphoviridae	prokaryote
v0740	54754	73	22	3	Complete	100	0	Siphoviridae	prokaryote
v0741	33950	45	15	0	Complete	100	0	unclassified	unclassified
v0742	34530	50	28	0	Complete	100	0	Siphoviridae	prokaryote
v0743	30391	37	13	0	Medium-quality	70.35	0	Siphoviridae	prokaryote
v0744	14060	21	8	0	Complete	100	0	unclassified	unclassified
v0745	53394	76	26	0	Medium-quality	88.49	0	Siphoviridae	prokaryote
v0746	32013	46	27	1	Medium-quality	81.82	0	Siphoviridae	prokaryote
v0747	52708	52	10	2	Complete	100	0	unclassified	unclassified
v0748	22967	43	12	0	Medium-quality	77.78	0	Siphoviridae	prokaryote
v0749	42428	49	20	0	Complete	100	0	unclassified	unclassified
v074a	31629	38	22	0	Medium-quality	52.38	0	Siphoviridae	prokaryote
v074b	32634	54	18	0	High-quality	95.66	0	Siphoviridae	prokaryote
v074c	9153	13	4	0	Medium-quality	77.78	0	Podoviridae	prokaryote
v074d	17964	32	19	0	Medium-quality	80.98	0	Siphoviridae	prokaryote
v074e	41308	44	16	0	Complete	100	0	Autographiviridae	prokaryote
v074f	22071	34	16	0	Medium-quality	57.6	0	Siphoviridae	prokaryote
v0750	10621	13	5	0	High-quality	90.23	0	Podoviridae	prokaryote
v0751	45636	63	25	0	High-quality	100	0	Myoviridae	prokaryote
v0752	45253	50	1	9	Medium-quality	68.27	0	unclassified	unclassified
v0753	38179	56	23	1	Medium-quality	66.17	0	Siphoviridae	prokaryote

v0754	40394	52	18	0	Complete	100	0	Siphoviridae	prokaryote
v0755	169476	238	37	6	Complete	100	0	unclassified	unclassified
v0756	31805	31	1	4	Medium-quality	79.19	0	unclassified	unclassified
v0757	17036	20	7	0	Medium-quality	54.4	0	unclassified	unclassified
v0758	5903	10	3	0	Complete	100	0	unclassified	unclassified
v0759	28012	36	25	0	Medium-quality	70.41	0	Podoviridae	prokaryote
v075a	39390	50	28	1	Complete	100	0	Podoviridae	prokaryote
v075b	5405	9	6	0	Complete	100	0	Microviridae	prokaryote
v075c	30058	46	36	0	Medium-quality	63.28	0	Siphoviridae	prokaryote
v075d	24246	38	14	0	Medium-quality	67.53	0	Siphoviridae	prokaryote
v075e	47617	78	47	0	Complete	100	0	Siphoviridae	prokaryote
v075f	41493	63	23	1	Complete	100	0	Siphoviridae	prokaryote
v0760	21731	32	8	1	Medium-quality	75.03	0	unclassified	unclassified
v0761	20087	24	14	0	Medium-quality	51.08	0	Siphoviridae	prokaryote
v0762	3081	2	2	0	High-quality	90.9	0	Anelloviridae	eukaryote
v0763	6168	10	3	0	Medium-quality	62.41	0	Inoviridae	prokaryote
v0764	2712	2	2	0	Medium-quality	77.58	0	unclassified	unclassified
v0765	34632	57	22	0	High-quality	100	0	Siphoviridae	prokaryote
v0766	4540	6	5	0	High-quality	100	0	Microviridae	prokaryote
v0767	2753	2	1	0	Medium-quality	77.99	0	Anelloviridae	eukaryote
v0768	41524	54	39	0	High-quality	100	0	Siphoviridae	prokaryote
v0769	2520	2	2	0	Medium-quality	68.7	0	Anelloviridae	eukaryote
v076a	5551	9	6	0	Complete	100	0	Microviridae	prokaryote
v076b	41075	54	29	0	Complete	100	0	Myoviridae	prokaryote
v076c	2636	1	1	0	Medium-quality	72.01	0	Anelloviridae	eukaryote
v076d	2708	2	1	0	Medium-quality	76.93	0	Anelloviridae	eukaryote
v076e	3063	2	1	0	Medium-quality	89.45	0	Anelloviridae	eukaryote
v076f	43559	59	47	0	High-quality	99.79	0	Autographiviridae	prokaryote
v0770	3648	2	2	0	High-quality	100	0	unclassified	unclassified
v0771	2321	1	1	0	Medium-quality	65.82	0	Anelloviridae	eukaryote
v0772	2257	2	1	0	Medium-quality	64.68	0	Anelloviridae	eukaryote
v0773	3013	3	3	0	High-quality	90.29	0	Anelloviridae	eukaryote
v0774	2594	2	1	0	Medium-quality	71.41	0	Anelloviridae	eukaryote
v0775	17158	19	17	0	Medium-quality	51.01	0	Siphoviridae	prokaryote
v0776	2840	2	2	0	Medium-quality	78.05	0	Anelloviridae	eukaryote
v0777	22968	49	6	0	Medium-quality	63.06	0	unclassified	unclassified
v0778	5362	5	3	0	Medium-quality	84.95	0	unclassified	unclassified
v0779	37559	54	20	0	Complete	100	0	Siphoviridae	prokaryote
v077a	56458	86	31	0	High-quality	100	0	unclassified	unclassified
v077b	2580	2	2	0	Medium-quality	73.12	0	Anelloviridae	eukaryote

v077c	41544	46	22	0	Complete	100	0	unclassified	unclassified
v077d	44021	56	42	0	High-quality	100	0	Siphoviridae	prokaryote
v077e	2020	1	1	0	Medium-quality	57.4	0	Anelloviridae	eukaryote
v077f	3319	3	2	0	High-quality	94.69	0	Anelloviridae	eukaryote
v0780	2024	1	1	0	Medium-quality	56.66	0	unclassified	unclassified
v0781	2396	2	1	0	Medium-quality	82.8	0	Anelloviridae	eukaryote
v0782	2235	1	1	0	Medium-quality	68.93	0	unclassified	unclassified
v0783	2702	2	2	0	High-quality	98.47	0	Anelloviridae	eukaryote
v0784	3419	1	1	0	High-quality	97.83	0	Anelloviridae	eukaryote
v0785	17964	24	8	0	High-quality	99.99	0	Podoviridae	prokaryote
v0786	3027	2	2	0	Medium-quality	86.79	0	Anelloviridae	eukaryote
v0787	52730	97	25	1	Complete	100	0	unclassified	unclassified
v0788	6172	11	4	0	Complete	100	0	unclassified	unclassified
v0789	2533	2	2	0	Medium-quality	86.09	0	Anelloviridae	eukaryote
v078a	2976	2	2	0	Medium-quality	83.62	0	Anelloviridae	eukaryote
v078b	2833	1	1	0	Medium-quality	83.03	0	Anelloviridae	eukaryote
v078c	2040	1	1	0	Medium-quality	60.3	0	Anelloviridae	eukaryote
v078d	2541	1	1	0	High-quality	90.14	0	Anelloviridae	eukaryote
v078e	2624	4	3	0	High-quality	92.81	0	Anelloviridae	eukaryote
v078f	2621	3	1	0	Medium-quality	63.42	0	Circoviridae	eukaryote
v0790	2818	3	3	0	Medium-quality	88	0	Anelloviridae	eukaryote
v0791	36590	58	15	2	High-quality	94.58	0	unclassified	unclassified
v0792	3681	3	3	0	High-quality	100	0	unclassified	unclassified
v0793	2496	1	1	0	Medium-quality	73.25	0	Anelloviridae	eukaryote
v0794	2056	2	2	0	Medium-quality	63.09	0	Anelloviridae	eukaryote
v0795	2645	3	3	0	High-quality	92.96	0	Anelloviridae	eukaryote
v0796	2374	2	1	0	Medium-quality	66.45	0	unclassified	unclassified
v0797	2570	2	2	0	Medium-quality	78.8	0	Anelloviridae	eukaryote
v0798	42010	48	12	0	High-quality	100	0	unclassified	unclassified
v0799	34345	50	22	0	Medium-quality	85.55	0	unclassified	unclassified
v079a	43115	53	41	0	High-quality	100	0	Siphoviridae	prokaryote
v079b	43363	70	13	1	High-quality	100	0	unclassified	unclassified
v079c	22211	30	7	0	Medium-quality	65.09	0	unclassified	unclassified
v079d	39774	57	15	3	Complete	100	0	Siphoviridae	prokaryote
v079e	2916	4	2	0	Medium-quality	62.45	0	Microviridae	prokaryote
v079f	41529	62	23	0	High-quality	96.06	0	Myoviridae	prokaryote
v07a0	35233	54	16	1	Complete	100	0	unclassified	unclassified
v07a1	20800	23	11	0	Medium-quality	58.02	0	Siphoviridae	prokaryote
v07a2	30450	37	12	0	Medium-quality	84.38	0	unclassified	unclassified
v07a3	34662	58	39	0	High-quality	96.77	0	Siphoviridae	prokaryote

v07a4	64735	79	29	0	High-quality	97.26	0	Siphoviridae	prokaryote
v07a5	31029	39	15	0	Medium-quality	51.26	0	Siphoviridae	prokaryote
v07a6	34731	41	21	0	Medium-quality	79.81	0	Siphoviridae	prokaryote
v07a7	40100	58	21	1	Complete	100	0	Siphoviridae	prokaryote
v07a8	35950	51	8	0	Complete	100	0	unclassified	unclassified
v07a9	39431	57	30	0	Medium-quality	86.03	0	Siphoviridae	prokaryote
v07aa	40957	62	44	0	Complete	100	0	Siphoviridae	prokaryote
v07ab	41992	69	25	0	High-quality	100	0	Siphoviridae	prokaryote
v07ac	27420	48	15	0	Medium-quality	83.3	0	unclassified	unclassified
v07ad	28193	35	15	0	Medium-quality	72.42	0	Siphoviridae	prokaryote
v07ae	43640	56	29	0	Medium-quality	72.49	0	Siphoviridae	prokaryote
v07af	31719	45	4	0	Medium-quality	75.71	0	unclassified	unclassified
v07b0	19950	25	5	0	Medium-quality	54.48	0	unclassified	unclassified
v07b1	26327	33	6	0	Medium-quality	68.11	0	unclassified	unclassified
v07b2	41527	52	30	0	Medium-quality	69.18	0	Siphoviridae	prokaryote
v07b3	95485	97	21	0	High-quality	98.48	0	unclassified	unclassified
v07b4	136974	196	41	4	Medium-quality	75.87	0	unclassified	unclassified
v07b5	41348	62	22	0	Complete	100	0	unclassified	unclassified
v07b6	39372	59	20	1	Complete	100	0	Siphoviridae	prokaryote
v07b7	39248	51	17	1	Complete	100	0	Siphoviridae	prokaryote
v07b8	34718	57	18	0	Complete	100	0	unclassified	unclassified
v07b9	5838	7	2	0	Complete	100	0	unclassified	unclassified
v07ba	29699	27	21	0	Medium-quality	51.03	0	Siphoviridae	prokaryote
v07bb	45348	73	35	0	Complete	100	0	Myoviridae	prokaryote
v07bc	169801	240	40	11	High-quality	100	0	unclassified	unclassified
v07bd	17169	23	10	0	Medium-quality	87.84	0	Salasmaviridae	prokaryote
v07be	6319	9	2	0	Complete	100	0	unclassified	unclassified
v07bf	40135	58	17	0	High-quality	98.55	0	unclassified	unclassified
v07c0	9348	10	5	0	High-quality	100	0	unclassified	unclassified
v07c1	15408	24	7	0	Medium-quality	50.92	0	unclassified	unclassified
v07c2	2197	2	1	0	Medium-quality	63.57	0	unclassified	unclassified
v07c3	63487	92	18	2	Complete	100	0	unclassified	unclassified
v07c4	38964	58	45	0	High-quality	99.81	0	Podoviridae	prokaryote
v07c5	13036	16	6	0	Complete	100	0	Podoviridae	prokaryote
v07c6	18036	22	8	0	Medium-quality	86.29	0	unclassified	unclassified
v07c7	34396	63	14	0	Medium-quality	82.62	0	unclassified	unclassified
v07c8	61940	87	16	0	Medium-quality	75.51	0	Quimbyviridae	prokaryote
v07c9	31517	43	35	0	Medium-quality	79.49	0	Siphoviridae	prokaryote
v07ca	13328	20	2	0	Medium-quality	89.73	0	unclassified	unclassified
v07cb	2784	3	2	0	Medium-quality	63.44	0	Microviridae	prokaryote

v07cc	32314	57	23	0	High-quality	94.72	0	Siphoviridae	prokaryote
v07cd	86898	147	24	1	Medium-quality	87.55	0	Podoviridae_crAss-like	prokaryote
v07ce	25674	28	24	0	Medium-quality	67.88	0	Siphoviridae	prokaryote
v07cf	38985	59	31	1	Medium-quality	82.02	0	Myoviridae	prokaryote
v07d0	40680	57	30	0	Medium-quality	67.75	0	Siphoviridae	prokaryote
v07d1	35013	61	19	1	Complete	100	0	Siphoviridae	prokaryote
v07d2	30405	38	6	0	Medium-quality	78.57	0	unclassified	unclassified
v07d3	23438	30	12	0	Medium-quality	53.18	0	Podoviridae	prokaryote
v07d4	31140	48	10	0	High-quality	100	0	unclassified	unclassified
v07d5	6144	7	3	0	Complete	100	0	Microviridae	prokaryote
v07d6	18088	15	3	0	Medium-quality	56.2	0	unclassified	unclassified
v07d7	29361	46	21	2	Medium-quality	87.81	0	Siphoviridae	prokaryote
v07d8	19000	21	19	0	Medium-quality	58.85	0	Siphoviridae	prokaryote
v07d9	44691	71	16	1	High-quality	99.06	0	unclassified	unclassified
v07da	5192	9	5	0	Complete	100	0	Microviridae	prokaryote
v07db	38221	60	36	0	High-quality	100	0	Siphoviridae	prokaryote
v07dc	48213	72	19	0	High-quality	96.94	0	unclassified	unclassified
v07dd	5845	7	3	0	Complete	100	0	unclassified	unclassified
v07de	61164	75	14	0	High-quality	97.07	0	unclassified	unclassified
v07df	6636	6	2	0	High-quality	100	0	unclassified	unclassified
v07e0	13756	18	6	0	High-quality	99.04	0	unclassified	unclassified
v07e1	51720	76	20	2	Complete	100	0	Siphoviridae	prokaryote
v07e2	16915	26	9	0	High-quality	90.43	0	unclassified	unclassified
v07e3	32586	59	16	0	Medium-quality	76.41	0	unclassified	unclassified
v07e4	41246	53	28	0	Medium-quality	68.43	0	Siphoviridae	prokaryote
v07e5	11764	12	5	0	High-quality	100	0	unclassified	unclassified
v07e6	6797	7	3	0	Complete	100	0	unclassified	unclassified
v07e7	5067	8	6	0	Complete	100	0	Microviridae	prokaryote
v07e8	4977	9	0	0	Complete	100	0	Inoviridae	prokaryote
v07e9	29814	44	12	1	Medium-quality	79.22	0	unclassified	unclassified
v07ea	6530	9	4	0	Complete	100	0	Microviridae	prokaryote
v07eb	38122	54	20	0	Complete	100	0	Siphoviridae	prokaryote
v07ec	3115	2	1	0	High-quality	91.19	0	Anelloviridae	eukaryote
v07ed	38632	58	37	0	High-quality	98.38	0	Siphoviridae	prokaryote
v07ee	45890	52	12	1	Medium-quality	75.03	0	unclassified	unclassified
v07ef	72721	65	2	28	High-quality	98.12	0	unclassified	unclassified
v07f0	27071	36	5	1	Medium-quality	55.99	0	unclassified	unclassified
v07f1	24886	35	0	1	Medium-quality	61.38	0	unclassified	unclassified
v07f2	29844	41	10	0	Medium-quality	60.29	0	unclassified	unclassified
v07f3	5133	6	6	0	Complete	100	0	Microviridae	prokaryote

v07f4	19465	28	7	0	Medium-quality	71.75	0	unclassified	unclassified
v07f5	25029	35	10	0	Medium-quality	60.55	0	Siphoviridae	prokaryote
v07f6	28338	34	14	0	Medium-quality	78.68	0	unclassified	unclassified
v07f7	3129	3	2	0	Medium-quality	88.44	0	Anelloviridae	eukaryote
v07f8	43224	65	17	0	Medium-quality	89.41	0	Myoviridae	prokaryote
v07f9	38590	55	42	0	High-quality	100	0	Siphoviridae	prokaryote
v07fa	14972	24	11	0	High-quality	100	0	Siphoviridae	prokaryote
v07fb	2094	2	1	0	Medium-quality	72.74	0	Anelloviridae	eukaryote
v07fc	41499	58	29	1	High-quality	95.57	0	Siphoviridae	prokaryote
v07fd	80258	137	16	3	Medium-quality	55.5	0	unclassified	unclassified
v07fe	6209	4	4	0	High-quality	97.22	0	Virgaviridae	eukaryote
v07ff	34010	48	15	0	Complete	100	0	unclassified	unclassified
v0800	39272	68	17	2	High-quality	100	0	Siphoviridae	prokaryote
v0801	31916	48	20	1	Medium-quality	84.72	0	Siphoviridae	prokaryote
v0802	21119	29	7	0	Medium-quality	68.96	0	unclassified	unclassified
v0803	39661	60	20	1	Complete	100	0	Siphoviridae	prokaryote
v0804	38414	61	7	0	Complete	100	0	unclassified	unclassified
v0805	26774	44	11	4	Medium-quality	73.78	0	Siphoviridae	prokaryote
v0806	29895	53	28	0	Medium-quality	78.32	0	Siphoviridae	prokaryote
v0807	37811	51	35	0	High-quality	95.04	0	Siphoviridae	prokaryote
v0808	38065	58	37	0	Complete	100	0	Siphoviridae	prokaryote
v0809	24431	31	21	0	Medium-quality	62.79	0	Siphoviridae	prokaryote
v080a	38602	55	14	0	Complete	100	0	Siphoviridae	prokaryote
v080b	17437	22	17	0	Medium-quality	50.12	0	Siphoviridae	prokaryote
v080c	26102	31	21	0	Medium-quality	64.51	0	Siphoviridae	prokaryote
v080d	16132	19	5	0	Medium-quality	53.7	0	unclassified	unclassified
v080e	37734	49	27	0	Medium-quality	67.89	0	Siphoviridae	prokaryote
v080f	42645	68	41	0	Medium-quality	61.22	0	Myoviridae	prokaryote
v0810	40204	61	19	0	Complete	100	0	unclassified	unclassified
v0811	38571	51	34	0	Complete	100	0	Siphoviridae	prokaryote
v0812	39911	50	21	0	Medium-quality	88.56	0	Siphoviridae	prokaryote
v0813	2798	3	2	0	Medium-quality	81.9	0	Anelloviridae	eukaryote
v0814	2542	2	2	0	Medium-quality	72.05	0	Anelloviridae	eukaryote
v0815	2286	2	2	0	Medium-quality	78.01	0	Anelloviridae	eukaryote
v0816	2409	2	1	0	Medium-quality	67.57	0	Anelloviridae	eukaryote
v0817	2466	1	1	0	Medium-quality	75.88	0	unclassified	unclassified
v0818	2835	2	2	0	Medium-quality	82.31	0	Anelloviridae	eukaryote
v0819	2503	1	1	0	High-quality	90.21	0	Anelloviridae	eukaryote
v081a	43069	63	27	1	High-quality	100	0	Siphoviridae	prokaryote
v081b	2944	2	2	0	Complete	100	0	Anelloviridae	eukaryote

v081c	2022	3	1	0	Medium-quality	57.27	0	Anelloviridae	eukaryote
v081d	2466	3	3	0	Medium-quality	85.34	0	Anelloviridae	eukaryote
v081e	2242	1	1	0	Complete	100	0	Anelloviridae	eukaryote
v081f	2895	2	1	0	Medium-quality	83.6	0	Anelloviridae	eukaryote
v0820	2107	2	2	0	Medium-quality	60.89	0	Anelloviridae	eukaryote
v0821	2659	2	1	0	Medium-quality	75.84	0	Anelloviridae	eukaryote
v0822	2082	1	1	0	Medium-quality	59.52	0	unclassified	unclassified
v0823	3399	6	1	0	Medium-quality	50.66	0	unclassified	unclassified
v0824	2613	2	1	0	Medium-quality	80.71	0	Anelloviridae	eukaryote
v0825	2607	2	2	0	Medium-quality	73.56	0	Anelloviridae	eukaryote
v0826	2433	1	1	0	Complete	100	0	Anelloviridae	eukaryote
v0827	3094	2	1	0	Medium-quality	86.19	0	Anelloviridae	eukaryote
v0828	38611	62	19	0	High-quality	96.43	0	Siphoviridae	prokaryote
v0829	35040	60	22	2	High-quality	96.69	0	Siphoviridae	prokaryote
v082a	2543	2	1	0	Medium-quality	74.4	0	Anelloviridae	eukaryote
v082b	2524	2	2	0	Medium-quality	71.03	0	Anelloviridae	eukaryote
v082c	2101	1	1	0	Medium-quality	59.59	0	Anelloviridae	eukaryote
v082d	2038	1	1	0	Medium-quality	57.85	0	unclassified	unclassified
v082e	2532	2	1	0	Medium-quality	70.74	0	Anelloviridae	eukaryote
v082f	3052	3	3	0	Medium-quality	88.99	0	Anelloviridae	eukaryote
v0830	2544	3	1	0	Medium-quality	74.01	0	Anelloviridae	eukaryote
v0831	6534	7	3	0	High-quality	100	0	unclassified	unclassified
v0832	2011	2	2	0	Medium-quality	69.29	0	Anelloviridae	eukaryote
v0833	2770	2	2	0	Medium-quality	85.54	0	Anelloviridae	eukaryote
v0834	68509	106	15	1	High-quality	98.7	0	Quimbyviridae	prokaryote
v0835	2451	2	2	0	Medium-quality	69.99	0	Anelloviridae	eukaryote
v0836	2175	1	1	0	Medium-quality	65.58	0	Anelloviridae	eukaryote
v0837	2848	2	1	0	High-quality	97.38	0	Anelloviridae	eukaryote
v0838	2615	1	1	0	Medium-quality	81.28	0	unclassified	unclassified
v0839	2780	2	2	0	Medium-quality	54.18	0	Parvoviridae	eukaryote
v083a	2466	2	1	0	Medium-quality	87.57	0	Anelloviridae	eukaryote
v083b	2117	1	1	0	Medium-quality	65.87	0	Anelloviridae	eukaryote
v083c	5125	10	5	0	High-quality	100	0	Genomoviridae	eukaryote
v083d	2535	2	1	0	Medium-quality	78.9	0	Anelloviridae	eukaryote
v083e	48997	72	30	2	High-quality	100	0	Myoviridae	prokaryote
v083f	14150	20	7	0	Complete	100	0	unclassified	unclassified
v0840	42338	66	35	1	High-quality	100	0	Siphoviridae	prokaryote
v0841	39948	55	25	0	High-quality	99.55	0	Siphoviridae	prokaryote
v0842	29982	40	14	0	High-quality	95.36	0	Siphoviridae	prokaryote
v0843	38499	63	18	0	Complete	100	0	Siphoviridae	prokaryote

v0844	57947	98	18	2	High-quality	96.48	0	unclassified	unclassified
v0845	62629	77	13	0	Complete	100	0	unclassified	unclassified
v0846	117005	142	32	6	High-quality	99.11	0	unclassified	unclassified
v0847	5828	8	5	1	High-quality	94.35	9.95	Microviridae	prokaryote
v0848	89837	154	17	1	High-quality	100	0	unclassified	unclassified
v0849	3220	5	1	0	Medium-quality	88.87	0	Circoviridae	eukaryote
v084a	3991	5	2	1	Medium-quality	50.68	22.07	unclassified	unclassified
v084b	28682	38	6	0	Medium-quality	82.47	0	unclassified	unclassified
v084c	6481	7	4	0	High-quality	100	0	unclassified	unclassified
v084d	7023	8	3	0	High-quality	100	0	unclassified	unclassified
v084e	2936	6	2	0	Medium-quality	52.81	0	unclassified	unclassified
v084f	4427	9	5	0	Medium-quality	78.97	0	Microviridae	prokaryote
v0850	5574	9	4	0	High-quality	98.49	0	Microviridae	prokaryote
v0851	25076	29	4	3	Medium-quality	55.16	36.89	unclassified	unclassified
v0852	33677	58	18	1	Medium-quality	83.44	0	unclassified	unclassified
v0853	43373	65	11	0	High-quality	100	0	unclassified	unclassified
v0854	28215	38	16	0	Medium-quality	76.71	0	Siphoviridae	prokaryote
v0855	34733	50	19	0	High-quality	100	0	Siphoviridae	prokaryote
v0856	35301	59	24	0	High-quality	98.21	0	Siphoviridae	prokaryote
v0857	34942	46	17	0	High-quality	93.05	0	Siphoviridae	prokaryote
v0858	4272	4	2	0	Medium-quality	68.8	0	unclassified	unclassified
v0859	6606	8	4	0	High-quality	100	0	Microviridae	prokaryote
v085a	45124	70	22	0	High-quality	91.24	0	Myoviridae	prokaryote
v085b	6358	6	3	0	High-quality	100	0	Microviridae	prokaryote
v085c	64672	78	44	4	High-quality	100	0	Myoviridae	prokaryote
v085d	5810	7	2	0	High-quality	97.34	0	unclassified	unclassified
v085e	18344	21	8	0	High-quality	96.54	0	unclassified	unclassified
v085f	20446	22	4	3	Medium-quality	71.27	0	unclassified	unclassified
v0860	102791	108	19	0	High-quality	100	0	Podoviridae_crAss-like	prokaryote
v0861	34656	55	25	0	High-quality	90.41	0	Siphoviridae	prokaryote
v0862	35420	59	13	3	High-quality	90.45	0	unclassified	unclassified
v0863	5924	6	3	0	High-quality	92.56	0	unclassified	unclassified
v0864	25900	52	12	0	Medium-quality	71.96	0	unclassified	unclassified
v0865	23065	36	9	0	Medium-quality	58.21	0	unclassified	unclassified
v0866	7069	9	2	0	High-quality	100	0	unclassified	unclassified
v0867	43213	41	7	2	Medium-quality	50.23	0	unclassified	unclassified
v0868	44632	47	4	0	High-quality	98.67	0	unclassified	unclassified
v0869	41074	64	6	1	High-quality	99.2	0	unclassified	unclassified
v086a	36541	55	29	0	High-quality	100	0	Siphoviridae	prokaryote
v086b	43183	57	6	0	Complete	100	0	unclassified	unclassified

v086c	42514	59	28	0	High-quality	100	0	Myoviridae	prokaryote
v086d	46183	70	7	3	Medium-quality	52.38	0	Quimbyviridae	prokaryote
v086e	26357	36	24	0	Medium-quality	58.89	0	Myoviridae	prokaryote
v086f	32716	52	8	0	High-quality	100	0	unclassified	unclassified
v0870	32336	50	19	0	Medium-quality	73.07	0	Myoviridae	prokaryote
v0871	28139	35	6	0	Medium-quality	65.14	0	unclassified	unclassified
v0872	25420	47	6	0	Medium-quality	58.85	0	unclassified	unclassified
v0873	89628	101	9	2	High-quality	90.26	0	unclassified	unclassified
v0874	100765	120	13	3	Complete	100	0	unclassified	unclassified
v0875	20273	40	12	0	Medium-quality	50.62	0	Siphoviridae	prokaryote
v0876	33853	37	8	1	Medium-quality	74.34	0	unclassified	unclassified
v0877	79241	118	11	2	High-quality	90.27	0	Quimbyviridae	prokaryote
v0878	75765	93	9	0	Complete	100	0	unclassified	unclassified
v0879	34406	56	26	0	Complete	100	0	Siphoviridae	prokaryote
v087a	3316	4	1	0	Medium-quality	83.48	0	Circoviridae	eukaryote
v087b	35260	40	9	0	Medium-quality	84.25	0	unclassified	unclassified
v087c	3308	6	1	0	Medium-quality	86.18	0	Circoviridae	eukaryote
v087d	67739	75	15	4	High-quality	100	0	unclassified	unclassified
v087e	37034	55	8	1	Complete	100	0	unclassified	unclassified
v087f	43153	55	11	1	High-quality	99.92	0	unclassified	unclassified
v0880	34851	62	13	0	High-quality	95	0	unclassified	unclassified
v0881	37937	50	22	0	Complete	100	0	Siphoviridae	prokaryote
v0882	30506	33	3	0	Medium-quality	70.59	0	unclassified	unclassified
v0883	63251	109	22	4	High-quality	100	0	unclassified	unclassified
v0884	41666	73	18	1	High-quality	99.46	0	unclassified	unclassified
v0885	58596	81	11	0	Complete	100	0	unclassified	unclassified
v0886	44447	68	15	0	Complete	100	0	unclassified	unclassified
v0887	38465	59	14	2	High-quality	94.45	0	unclassified	unclassified
v0888	46867	53	4	2	High-quality	100	0	unclassified	unclassified
v0889	6097	7	5	0	High-quality	100	0	Microviridae	prokaryote
v088a	294360	342	43	6	Complete	100	0	unclassified	unclassified
v088b	86612	117	13	4	High-quality	100	0	unclassified	unclassified
v088c	99864	158	13	1	High-quality	96.87	0	Podoviridae_crAss-like	prokaryote
v088d	75449	87	29	0	Complete	100	0	Schitoviridae	prokaryote
v088e	33761	38	4	1	Medium-quality	81.94	0	unclassified	unclassified
v088f	7094	9	2	0	High-quality	100	0	unclassified	unclassified
v0890	5629	10	4	0	Complete	100	0	Microviridae	prokaryote
v0891	16045	19	6	0	Medium-quality	54	0	unclassified	unclassified
v0892	7385	16	1	0	Medium-quality	58.06	0	unclassified	unclassified
v0893	23779	30	1	0	Medium-quality	50.51	0	unclassified	unclassified

v0894	17940	25	7	1	High-quality	100	0	unclassified	unclassified
v0895	47353	36	4	1	Medium-quality	75.29	0	unclassified	unclassified
v0896	18833	25	14	0	Medium-quality	53.61	0	unclassified	unclassified
v0897	31926	30	6	0	Medium-quality	76.27	0	unclassified	unclassified
v0898	7672	6	1	0	High-quality	100	0	unclassified	unclassified
v0899	25345	46	13	0	Medium-quality	68.96	0	unclassified	unclassified
v089a	54450	55	8	1	Medium-quality	86.56	0	Quimbyviridae	prokaryote
v089b	44628	34	3	2	Medium-quality	71.73	0	unclassified	unclassified
v089c	45038	59	11	2	High-quality	100	0	unclassified	unclassified
v089d	39801	58	14	0	Complete	100	0	unclassified	unclassified
v089e	4548	7	1	0	High-quality	100	0	unclassified	unclassified
v089f	12116	10	1	0	Medium-quality	78.24	0	unclassified	unclassified
v08a0	36618	70	7	1	Medium-quality	63.53	0	unclassified	unclassified
v08a1	88508	127	14	2	Complete	100	0	Quimbyviridae	prokaryote
v08a2	32103	46	19	0	Medium-quality	88.21	0	Siphoviridae	prokaryote
v08a3	59418	110	23	0	Complete	100	0	unclassified	unclassified
v08a4	20847	27	5	0	Medium-quality	51.74	0	unclassified	unclassified
v08a5	75552	85	25	3	Medium-quality	53.24	0	unclassified	unclassified
v08a6	40461	72	11	1	Complete	100	0	unclassified	unclassified
v08a7	25438	41	11	0	Medium-quality	57.52	0	unclassified	unclassified
v08a8	24381	27	11	0	Medium-quality	61.56	0	unclassified	unclassified
v08a9	41418	59	16	0	High-quality	100	0	unclassified	unclassified
v08aa	42031	54	9	1	High-quality	100	0	unclassified	unclassified
v08ab	44050	65	12	0	High-quality	100	0	unclassified	unclassified
v08ac	58240	80	14	5	Medium-quality	86.55	0	unclassified	unclassified
v08ad	44901	57	12	0	Complete	100	0	unclassified	unclassified
v08ae	101499	116	11	2	High-quality	99	0	unclassified	unclassified
v08af	3973	5	1	0	High-quality	95.47	0	unclassified	unclassified
v08b0	20289	40	10	1	Medium-quality	52.02	0	Siphoviridae	prokaryote
v08b1	50259	75	20	2	Complete	100	0	Siphoviridae	prokaryote
v08b2	4387	7	1	0	High-quality	100	0	unclassified	unclassified
v08b3	163875	293	16	1	Complete	100	0	unclassified	unclassified
v08b4	22686	30	3	2	Medium-quality	50.29	0	unclassified	unclassified
v08b5	51784	79	14	1	High-quality	100	0	unclassified	unclassified
v08b6	35568	44	16	0	High-quality	95.76	0	Siphoviridae	prokaryote
v08b7	48962	85	11	0	Medium-quality	65.89	0	unclassified	unclassified
v08b8	31946	39	21	0	Medium-quality	51.14	0	Siphoviridae	prokaryote
v08b9	21089	30	11	0	Medium-quality	52.78	0	Siphoviridae	prokaryote
v08ba	45365	66	9	3	Medium-quality	59.18	0	unclassified	unclassified
v08bb	96461	167	16	1	Complete	100	0	Podoviridae_crAss-like	prokaryote

v08bc	43768	58	7	1	Complete	100	0	unclassified	unclassified
v08bd	44442	66	15	1	High-quality	100	0	unclassified	unclassified
v08be	126931	161	19	8	Complete	100	0	unclassified	unclassified
v08bf	32986	61	16	0	Complete	100	0	unclassified	unclassified
v08c0	100789	151	32	2	Complete	100	0	unclassified	unclassified
v08c1	43955	60	9	0	Complete	100	0	unclassified	unclassified
v08c2	54618	47	4	3	Medium-quality	69.95	0	unclassified	unclassified
v08c3	41066	41	8	1	Medium-quality	82.47	0	unclassified	unclassified
v08c4	71771	109	18	2	Complete	100	0	unclassified	unclassified
v08c5	56047	83	31	3	High-quality	91.94	0	Siphoviridae	prokaryote
v08c6	5568	8	7	0	Complete	100	0	Microviridae	prokaryote
v08c7	27747	38	22	0	Medium-quality	65.9	0	unclassified	unclassified
v08c8	92043	138	16	3	High-quality	100	0	Quimbyviridae	prokaryote
v08c9	16268	19	5	3	Medium-quality	56.71	0	Siphoviridae	prokaryote
v08ca	52047	74	2	0	Medium-quality	53.06	0	unclassified	unclassified
v08cb	346124	450	55	10	High-quality	92.69	0	unclassified	unclassified
v08cc	28777	38	7	0	Complete	100	0	unclassified	unclassified
v08cd	27219	25	17	0	Medium-quality	64.84	0	Autographiviridae	prokaryote
v08ce	20918	23	3	0	Medium-quality	73.17	0	unclassified	unclassified
v08cf	20791	20	11	0	Medium-quality	51.64	0	Autographiviridae	prokaryote
v08d0	100706	124	9	2	High-quality	96.55	0	unclassified	unclassified
v08d1	75756	100	8	0	High-quality	100	0	unclassified	unclassified
v08d2	15603	17	1	0	High-quality	100	0	unclassified	unclassified
v08d3	43923	75	13	0	Complete	100	0	unclassified	unclassified
v08d4	121961	178	31	2	Complete	100	0	unclassified	unclassified
v08d5	17819	24	17	0	Medium-quality	55.57	0	Myoviridae	prokaryote
v08d6	33583	53	16	0	High-quality	97.61	0	Siphoviridae	prokaryote
v08d7	35233	52	23	0	Medium-quality	83.73	0	Myoviridae	prokaryote
v08d8	66399	118	33	0	High-quality	91.58	0	unclassified	unclassified
v08d9	19087	42	16	0	Medium-quality	84.18	0	Siphoviridae	prokaryote
v08da	59341	91	11	0	High-quality	100	0	unclassified	unclassified
v08db	66263	78	9	2	Complete	100	0	unclassified	unclassified
v08dc	28833	40	14	0	Medium-quality	72.24	0	Siphoviridae	prokaryote
v08dd	35601	47	19	2	Medium-quality	76.95	0	Siphoviridae	prokaryote
v08de	22752	39	15	0	Medium-quality	50.93	0	Siphoviridae	prokaryote
v08df	29734	46	12	0	Medium-quality	70.02	0	unclassified	unclassified
v08e0	36686	52	6	2	High-quality	93.95	0	unclassified	unclassified
v08e1	38595	70	12	2	Complete	100	0	unclassified	unclassified
v08e2	34918	55	12	1	High-quality	91.45	0	unclassified	unclassified
v08e3	51475	96	16	1	Medium-quality	63.91	0	unclassified	unclassified

v08e4	44495	65	26	2	High-quality	100	0	Myoviridae	prokaryote
v08e5	42891	51	13	0	Complete	100	0	unclassified	unclassified
v08e6	15996	21	4	2	Medium-quality	55.75	0	Siphoviridae	prokaryote
v08e7	37723	56	13	0	High-quality	95.01	0	unclassified	unclassified
v08e8	30247	45	15	0	Complete	100	0	Siphoviridae	prokaryote
v08e9	35104	71	13	2	Medium-quality	88.03	0	unclassified	unclassified
v08ea	39283	64	18	0	Medium-quality	88.88	0	unclassified	unclassified
v08eb	23786	25	2	1	Medium-quality	50.53	0	unclassified	unclassified
v08ec	35704	37	4	2	Medium-quality	75.85	0	unclassified	unclassified
v08ed	29020	43	12	6	Medium-quality	67.6	0	unclassified	unclassified
v08ee	34144	53	16	1	Medium-quality	72.52	0	Myoviridae	prokaryote
v08ef	34884	56	22	2	Medium-quality	87.43	0	Siphoviridae	prokaryote
v08f0	53197	86	23	0	High-quality	98.13	0	Myoviridae	prokaryote
v08f1	29851	48	16	0	Medium-quality	74.73	0	unclassified	unclassified
v08f2	55173	100	20	1	Complete	100	0	unclassified	unclassified
v08f3	35187	47	11	0	Complete	100	0	unclassified	unclassified
v08f4	36175	59	9	1	High-quality	98.91	0	unclassified	unclassified
v08f5	57577	99	18	2	Complete	100	0	unclassified	unclassified
v08f6	43339	49	18	3	Medium-quality	86	0	Siphoviridae	prokaryote
v08f7	35258	39	13	3	Medium-quality	65.89	0	Siphoviridae	prokaryote
v08f8	35561	43	7	0	Medium-quality	57.69	0	unclassified	unclassified
v08f9	21878	44	12	0	Medium-quality	61	0	Siphoviridae	prokaryote
v08fa	42134	56	10	0	Complete	100	0	unclassified	unclassified
v08fb	45726	77	19	1	High-quality	100	0	unclassified	unclassified
v08fc	30307	48	3	2	High-quality	100	0	unclassified	unclassified
v08fd	38531	74	18	1	High-quality	95	0	Siphoviridae	prokaryote
v08fe	17723	25	3	1	Medium-quality	60.75	0	Siphoviridae	prokaryote
v08ff	148478	186	45	5	Complete	100	0	unclassified	unclassified
v0900	31064	45	6	3	Medium-quality	68.86	0	unclassified	unclassified
v0901	6193	7	4	0	High-quality	100	0	unclassified	unclassified
v0902	9127	11	3	1	High-quality	100	0	unclassified	unclassified
v0903	7307	10	2	0	Medium-quality	61.18	0	Myoviridae	prokaryote
v0904	45458	76	18	0	Complete	100	0	unclassified	unclassified
v0905	16353	22	9	0	Medium-quality	69.99	0	unclassified	unclassified
v0906	49619	80	21	0	High-quality	94.84	0	unclassified	unclassified
v0907	25104	52	7	1	Medium-quality	65.15	0	unclassified	unclassified
v0908	5132	9	6	0	Complete	100	0	Microviridae	prokaryote
v0909	39968	55	21	1	High-quality	97.86	0	Siphoviridae	prokaryote
v090a	45883	75	20	0	High-quality	94.4	0	unclassified	unclassified
v090b	65059	75	25	3	High-quality	100	0	Siphoviridae	prokaryote

v090c	6102	7	1	0	Complete	100	0	unclassified	unclassified
v090d	39668	67	24	3	Medium-quality	89.2	0	unclassified	unclassified
v090e	3094	4	3	0	Medium-quality	56.15	0	unclassified	unclassified
v090f	40545	64	22	2	High-quality	100	0	Siphoviridae	prokaryote
v0910	32645	50	5	1	Medium-quality	75.89	0	unclassified	unclassified
v0911	36095	42	9	0	Complete	100	0	unclassified	unclassified
v0912	15907	20	7	0	Medium-quality	68.12	0	Salasmaviridae	prokaryote
v0913	40991	73	22	1	Complete	100	0	unclassified	unclassified
v0914	40776	68	20	1	Complete	100	0	Siphoviridae	prokaryote
v0915	43888	78	16	0	High-quality	100	0	unclassified	unclassified
v0916	30285	37	25	1	Medium-quality	74.44	0	Podoviridae	prokaryote
v0917	27582	31	15	3	Medium-quality	58.8	0	Siphoviridae	prokaryote
v0918	49605	65	11	2	Medium-quality	72.72	0	unclassified	unclassified
v0919	37957	33	7	3	Medium-quality	79.93	0	Siphoviridae	prokaryote
v091a	158909	196	18	4	Complete	100	0	unclassified	unclassified
v091b	59035	70	23	2	High-quality	100	0	Siphoviridae	prokaryote
v091c	43620	74	17	0	Complete	100	0	unclassified	unclassified
v091d	10368	17	1	0	High-quality	100	0	Inoviridae	prokaryote
v091e	49763	68	5	1	Medium-quality	58.71	0	Quimbyviridae	prokaryote
v091f	15220	21	6	0	High-quality	90.06	0	unclassified	unclassified
v0920	37216	51	26	1	High-quality	100	0	Siphoviridae	prokaryote
v0921	13334	16	4	0	High-quality	99.81	0	unclassified	unclassified
v0922	10606	17	5	0	Medium-quality	61.33	0	unclassified	unclassified
v0923	16628	18	7	0	High-quality	100	0	unclassified	unclassified
v0924	7387	9	3	0	High-quality	100	0	Microviridae	prokaryote
v0925	13137	17	4	0	High-quality	100	0	Siphoviridae	prokaryote
v0926	53292	63	25	0	High-quality	100	0	Siphoviridae	prokaryote
v0927	29465	41	15	0	Complete	100	0	Siphoviridae	prokaryote
v0928	49715	69	21	1	High-quality	99.94	0	Myoviridae	prokaryote
v0929	35618	59	11	0	Complete	100	0	unclassified	unclassified
v092a	32180	50	24	0	Complete	100	0	Siphoviridae	prokaryote
v092b	8275	10	2	0	Medium-quality	61.94	0	unclassified	unclassified
v092c	27919	36	17	1	Medium-quality	58.99	0	Siphoviridae	prokaryote
v092d	61145	77	20	7	High-quality	100	0	Myoviridae	prokaryote
v092e	33963	51	18	0	Medium-quality	86	0	Siphoviridae	prokaryote
v092f	35738	43	12	1	High-quality	98.98	0	unclassified	unclassified
v0930	33466	46	18	1	High-quality	91.69	0	Siphoviridae	prokaryote
v0931	2842	4	1	0	Medium-quality	71.32	0	unclassified	unclassified
v0932	101971	98	14	1	High-quality	100	0	Podoviridae_crAss-like	prokaryote
v0933	53707	79	21	3	Medium-quality	87.49	0	Siphoviridae	prokaryote

v0934	39159	46	6	5	Medium-quality	80.25	0	unclassified	unclassified
v0935	43782	78	19	0	High-quality	100	0	unclassified	unclassified
v0936	45275	75	16	1	Complete	100	0	unclassified	unclassified
v0937	9304	14	1	0	High-quality	100	0	Inoviridae	prokaryote
v0938	4119	3	1	0	Complete	100	0	unclassified	unclassified
v0939	44408	61	10	1	High-quality	100	0	unclassified	unclassified
v093a	31645	39	5	2	Medium-quality	61.83	0	unclassified	unclassified
v093b	25923	29	5	0	Medium-quality	78.55	0	unclassified	unclassified
v093c	22656	34	15	2	Medium-quality	58.49	0	unclassified	unclassified
v093d	10260	11	1	0	Medium-quality	61.54	0	unclassified	unclassified
v093e	2285	5	1	0	Medium-quality	54.91	0	unclassified	unclassified
v093f	45409	68	9	1	Medium-quality	69.26	0	unclassified	unclassified
v0940	39512	63	14	2	High-quality	98.52	0	Siphoviridae	prokaryote
v0941	2706	2	1	0	Medium-quality	60.2	0	unclassified	unclassified
v0942	44488	67	16	1	Complete	100	0	unclassified	unclassified
v0943	54742	58	29	0	High-quality	100	0	Siphoviridae	prokaryote
v0944	9606	12	1	0	Medium-quality	80.84	0	unclassified	unclassified
v0945	38552	55	29	1	High-quality	93.81	0	Siphoviridae	prokaryote
v0946	39985	64	17	0	High-quality	100	0	unclassified	unclassified
v0947	81958	92	25	2	High-quality	99.55	0	Siphoviridae	prokaryote
v0948	38263	57	8	1	High-quality	98	0	unclassified	unclassified
v0949	15353	20	2	1	Medium-quality	80.56	0	unclassified	unclassified
v094a	5962	9	2	0	Complete	100	0	unclassified	unclassified
v094b	45137	76	23	0	High-quality	100	0	unclassified	unclassified
v094c	31445	32	5	2	Medium-quality	64.96	0	unclassified	unclassified
v094d	42165	71	21	1	Complete	100	0	unclassified	unclassified
v094e	29248	39	12	0	Medium-quality	83.34	0	Siphoviridae	prokaryote
v094f	4533	6	3	0	Medium-quality	74.28	0	unclassified	unclassified
v0950	37740	60	33	0	Medium-quality	82.24	0	Siphoviridae	prokaryote
v0951	21278	33	18	1	Medium-quality	52.89	0	Siphoviridae	prokaryote
v0952	33474	49	26	0	Medium-quality	73.83	0	Myoviridae	prokaryote
v0953	42767	60	17	6	High-quality	100	0	unclassified	unclassified
v0954	28352	42	4	2	Medium-quality	75.04	0	unclassified	unclassified
v0955	14330	20	4	0	High-quality	100	0	unclassified	unclassified
v0956	6304	8	3	0	High-quality	96.43	0	Microviridae	prokaryote
v0957	4732	7	7	0	High-quality	98.67	0	Microviridae	prokaryote
v0958	6960	9	4	0	High-quality	100	0	unclassified	unclassified
v0959	53344	73	18	4	High-quality	100	0	Siphoviridae	prokaryote
v095a	8751	13	2	1	Medium-quality	74.35	0	unclassified	unclassified
v095b	45261	57	46	1	High-quality	100	0	Siphoviridae	prokaryote

v095c	30206	34	28	0	Medium-quality	57.93	0	Siphoviridae	prokaryote
v095d	64362	81	9	7	High-quality	100	0	unclassified	unclassified
v095e	32710	39	30	1	Medium-quality	87.85	0	Myoviridae	prokaryote
v095f	6039	11	4	0	High-quality	100	0	unclassified	unclassified
v0960	3522	3	3	0	Medium-quality	62.35	0	Microviridae	prokaryote
v0961	58331	97	15	2	Complete	100	0	unclassified	unclassified
v0962	57289	67	22	3	High-quality	100	0	Siphoviridae	prokaryote
v0963	6670	11	4	0	High-quality	100	0	unclassified	unclassified
v0964	24025	30	12	1	Medium-quality	63.6	0	Siphoviridae	prokaryote
v0965	34123	50	19	1	Medium-quality	83.77	0	Siphoviridae	prokaryote
v0966	5956	8	2	0	Complete	100	0	unclassified	unclassified
v0967	47606	71	12	5	Medium-quality	59.25	0	unclassified	unclassified
v0968	30284	43	24	0	Medium-quality	83.09	0	Siphoviridae	prokaryote
v0969	5860	9	3	0	High-quality	94.5	0	unclassified	unclassified
v096a	38460	55	5	2	Medium-quality	89.4	0	unclassified	unclassified
v096b	60752	71	17	8	High-quality	100	0	unclassified	unclassified
v096c	27035	44	19	0	Medium-quality	72.01	0	Siphoviridae	prokaryote
v096d	15843	22	3	2	Medium-quality	55.22	0	unclassified	unclassified
v096e	5493	7	1	0	High-quality	91.8	0	unclassified	unclassified
v096f	36488	55	19	1	High-quality	91.93	0	Siphoviridae	prokaryote
v0970	4220	5	2	0	Medium-quality	76.28	0	unclassified	unclassified
v0971	45826	75	19	0	Complete	100	0	unclassified	unclassified
v0972	80181	133	29	4	Complete	100	0	unclassified	unclassified
v0973	37994	65	22	3	High-quality	100	0	unclassified	unclassified
v0974	34113	50	25	0	Medium-quality	75.75	0	Siphoviridae	prokaryote
v0975	109470	132	9	7	High-quality	100	0	unclassified	unclassified
v0976	42063	53	10	0	Complete	100	0	unclassified	unclassified
v0977	3345	4	2	0	Medium-quality	55.91	0	unclassified	unclassified
v0978	47870	66	12	0	Medium-quality	79.55	0	unclassified	unclassified
v0979	4333	7	1	0	Medium-quality	72.46	0	unclassified	unclassified
v097a	6660	9	4	0	Complete	100	0	unclassified	unclassified
v097b	46022	71	13	0	Complete	100	0	unclassified	unclassified
v097c	23723	32	4	1	Complete	100	0	unclassified	unclassified
v097d	33179	51	14	0	Medium-quality	87.73	0	unclassified	unclassified
v097e	39392	69	15	2	High-quality	100	0	unclassified	unclassified
v097f	30776	42	28	0	Medium-quality	87.59	0	Myoviridae	prokaryote
v0980	31677	48	15	2	High-quality	95.52	0	unclassified	unclassified
v0981	6089	10	4	0	High-quality	100	0	unclassified	unclassified
v0982	28312	40	17	0	Medium-quality	68.03	0	Siphoviridae	prokaryote
v0983	95690	108	14	5	Medium-quality	55.12	0	unclassified	unclassified

v0984	21952	34	11	1	Medium-quality	52.92	0	Siphoviridae	prokaryote
v0985	37417	55	15	1	Medium-quality	79.47	0	Myoviridae	prokaryote
v0986	41579	57	16	3	High-quality	100	0	unclassified	unclassified
v0987	25677	43	26	1	Medium-quality	67.99	0	Siphoviridae	prokaryote
v0988	35943	52	17	1	High-quality	91.86	0	Siphoviridae	prokaryote
v0989	28361	44	29	0	Medium-quality	61.52	0	Siphoviridae	prokaryote
v098a	5010	7	7	0	High-quality	100	0	Microviridae	prokaryote
v098b	31206	49	16	1	Medium-quality	88.32	0	Siphoviridae	prokaryote
v098c	24535	33	13	0	Medium-quality	72.16	0	unclassified	unclassified
v098d	75211	127	18	4	High-quality	98.12	0	unclassified	unclassified
v098e	6112	6	3	0	Complete	100	0	unclassified	unclassified
v098f	19073	35	8	0	Medium-quality	55.92	0	unclassified	unclassified
v0990	42009	46	5	3	High-quality	100	0	unclassified	unclassified
v0991	25014	33	13	0	Medium-quality	52.01	0	Siphoviridae	prokaryote
v0992	62092	67	19	4	Medium-quality	59.85	0	Myoviridae	prokaryote
v0993	12877	15	7	0	Medium-quality	67.98	0	Salasmaviridae	prokaryote
v0994	44061	63	27	3	High-quality	100	0	Myoviridae	prokaryote
v0995	26289	37	18	2	Medium-quality	57.78	0	Siphoviridae	prokaryote
v0996	43025	73	16	0	Complete	100	0	unclassified	unclassified
v0997	3557	3	1	0	Medium-quality	77.04	0	unclassified	unclassified
v0998	27767	28	17	1	Medium-quality	56.18	0	Siphoviridae	prokaryote
v0999	44064	62	21	4	Medium-quality	76.43	0	Siphoviridae	prokaryote
v099a	4191	2	1	0	Complete	100	0	unclassified	unclassified
v099b	34642	49	24	0	Medium-quality	75.78	0	Siphoviridae	prokaryote
v099c	8903	12	1	0	High-quality	100	0	unclassified	unclassified
v099d	26201	34	27	0	Medium-quality	69.08	0	Siphoviridae	prokaryote
v099e	35705	53	40	0	Complete	100	0	Siphoviridae	prokaryote
v099f	48880	67	11	1	Complete	100	0	unclassified	unclassified
v09a0	5888	7	2	0	Medium-quality	59.59	0	unclassified	unclassified
v09a1	38650	49	27	2	High-quality	100	0	Siphoviridae	prokaryote
v09a2	18817	23	14	1	Medium-quality	52.43	0	Siphoviridae	prokaryote
v09a3	18041	24	8	0	Complete	100	0	Podoviridae	prokaryote
v09a4	6745	8	4	0	High-quality	100	0	unclassified	unclassified
v09a5	29048	38	12	2	Medium-quality	72.43	0	Siphoviridae	prokaryote
v09a6	48471	79	28	6	Complete	100	0	Siphoviridae	prokaryote
v09a7	3700	6	1	0	Medium-quality	85.72	0	unclassified	unclassified
v09a8	2435	3	1	0	Medium-quality	52.65	0	Circoviridae	eukaryote
v09a9	39684	61	38	0	High-quality	100	0	Myoviridae	prokaryote
v09aa	3682	6	1	0	Medium-quality	77.22	0	unclassified	unclassified
v09ab	35614	51	25	0	Medium-quality	83.43	0	Siphoviridae	prokaryote

v09ac	19810	29	13	0	Medium-quality	53.99	0	Siphoviridae	prokaryote
v09ad	34521	55	17	1	Complete	100	0	Siphoviridae	prokaryote
v09ae	14304	17	6	0	High-quality	100	0	unclassified	unclassified
v09af	24091	37	11	0	Medium-quality	65.61	0	Siphoviridae	prokaryote
v09b0	32190	52	19	3	High-quality	94.78	0	unclassified	unclassified
v09b1	27031	41	18	1	Medium-quality	77.05	0	Siphoviridae	prokaryote
v09b2	65375	98	20	3	High-quality	100	0	unclassified	unclassified
v09b3	17199	24	8	0	High-quality	100	0	Siphoviridae	prokaryote
v09b4	3807	3	1	0	Complete	100	0	unclassified	unclassified
v09b5	36539	58	23	2	Medium-quality	62.08	0	Siphoviridae	prokaryote
v09b6	30966	46	36	0	Medium-quality	58.42	0	Myoviridae	prokaryote
v09b7	25810	33	17	0	Medium-quality	55.17	0	Siphoviridae	prokaryote
v09b8	31362	55	13	2	High-quality	93.3	0	Siphoviridae	prokaryote
v09b9	45970	70	17	0	High-quality	97.75	0	Myoviridae	prokaryote
v09ba	47513	76	4	2	High-quality	100	0	unclassified	unclassified
v09bb	33893	39	20	1	High-quality	97.87	0	Siphoviridae	prokaryote
v09bc	39744	56	14	1	High-quality	100	0	unclassified	unclassified
v09bd	17466	25	4	0	High-quality	100	0	unclassified	unclassified
v09be	24378	32	16	1	Medium-quality	68.31	0	Siphoviridae	prokaryote
v09bf	16054	24	8	0	High-quality	99.5	0	unclassified	unclassified
v09c0	70110	100	21	0	Medium-quality	86.61	0	unclassified	unclassified
v09c1	12797	14	5	0	Medium-quality	66.31	0	Podoviridae	prokaryote
v09c2	34225	48	22	0	Medium-quality	56.84	0	Siphoviridae	prokaryote
v09c3	38658	53	23	2	High-quality	100	0	Siphoviridae	prokaryote
v09c4	14715	23	11	0	High-quality	94.02	0	Siphoviridae	prokaryote
v09c5	2751	3	1	0	Medium-quality	56.01	0	unclassified	unclassified
v09c6	29043	41	17	0	Complete	100	0	unclassified	unclassified
v09c7	16719	29	8	0	Complete	100	0	unclassified	unclassified
v09c8	26036	36	24	0	Medium-quality	68.3	0	Siphoviridae	prokaryote
v09c9	16856	20	7	0	Medium-quality	87.79	0	Salasmaviridae	prokaryote
v09ca	54148	55	6	0	High-quality	98.29	0	unclassified	unclassified
v09cb	41165	59	29	0	Medium-quality	68.51	0	Siphoviridae	prokaryote
v09cc	4086	4	1	0	Medium-quality	87.5	0	unclassified	unclassified
v09cd	34112	55	13	3	Medium-quality	56.13	0	unclassified	unclassified
v09ce	14516	21	5	0	Medium-quality	80.07	0	Siphoviridae	prokaryote
v09cf	96305	156	20	1	High-quality	99.19	0	Podoviridae_crAss-like	prokaryote
v09d0	34060	47	13	0	High-quality	99.8	0	unclassified	unclassified
v09d1	42292	49	27	0	High-quality	100	0	Podoviridae	prokaryote
v09d2	16848	25	3	2	High-quality	100	0	Siphoviridae	prokaryote
v09d3	39760	57	40	0	High-quality	100	0	Myoviridae	prokaryote

v09d4	40380	53	34	1	High-quality	100	0	Siphoviridae	prokaryote
v09d5	6108	13	4	0	Medium-quality	50.58	0	Siphoviridae	prokaryote
v09d6	37200	54	23	0	Medium-quality	82.6	0	Siphoviridae	prokaryote
v09d7	33513	50	29	0	High-quality	100	0	Siphoviridae	prokaryote
v09d8	46472	34	1	0	Medium-quality	88.12	0	unclassified	unclassified
v09d9	4366	9	1	0	Medium-quality	70.41	0	unclassified	unclassified
v09da	3488	4	1	0	Medium-quality	80.5	0	unclassified	unclassified
v09db	11965	15	1	0	High-quality	91.16	0	Siphoviridae	prokaryote
v09dc	48842	66	12	1	High-quality	100	0	unclassified	unclassified
v09dd	41657	69	39	0	Complete	100	0	Myoviridae	prokaryote
v09de	16438	18	8	0	Medium-quality	83.43	0	Salasmaviridae	prokaryote
v09df	20053	28	23	0	Medium-quality	61.16	0	Myoviridae	prokaryote
v09e0	8707	13	2	1	Medium-quality	72.9	0	unclassified	unclassified
v09e1	85149	124	107	0	High-quality	93.61	0	Myoviridae	prokaryote
v09e2	25457	30	23	2	Medium-quality	66.9	0	Siphoviridae	prokaryote
v09e3	19854	33	15	1	Medium-quality	50.73	0	Siphoviridae	prokaryote
v09e4	14318	16	4	0	High-quality	100	0	unclassified	unclassified
v09e5	33383	52	21	0	High-quality	92.76	0	Myoviridae	prokaryote
v09e6	39667	58	19	0	Complete	100	0	Siphoviridae	prokaryote
v09e7	6422	9	1	0	High-quality	100	0	unclassified	unclassified
v09e8	5939	7	2	0	Medium-quality	60.17	0	unclassified	unclassified
v09e9	52504	62	14	0	High-quality	90.66	0	unclassified	unclassified
v09ea	31613	42	12	0	Medium-quality	89.52	0	Siphoviridae	prokaryote
v09eb	3260	4	1	0	Complete	100	0	unclassified	unclassified
v09ec	22537	30	3	1	Complete	100	0	unclassified	unclassified
v09ed	30053	51	28	0	Medium-quality	65.22	0	Siphoviridae	prokaryote
v09ee	23483	28	24	1	Medium-quality	72.73	0	Myoviridae	prokaryote
v09ef	18314	31	8	0	Medium-quality	51.79	0	Siphoviridae	prokaryote
v09f0	56713	79	25	0	High-quality	100	0	Siphoviridae	prokaryote
v09f1	29692	37	18	0	Medium-quality	55.48	0	Siphoviridae	prokaryote
v09f2	27772	40	22	0	Medium-quality	60.82	0	Myoviridae	prokaryote
v09f3	23507	30	19	0	Medium-quality	51.88	0	Siphoviridae	prokaryote
v09f4	24979	31	5	0	High-quality	100	0	unclassified	unclassified
v09f5	30711	44	13	1	Complete	100	0	unclassified	unclassified
v09f6	26660	31	13	0	Medium-quality	66.05	0	Siphoviridae	prokaryote
v09f7	22624	29	8	1	Medium-quality	52.21	0	Myoviridae	prokaryote
v09f8	34397	57	17	0	Medium-quality	88.94	0	unclassified	unclassified
v09f9	23096	32	14	0	Medium-quality	66.71	0	Siphoviridae	prokaryote
v09fa	7264	9	1	0	Medium-quality	65.74	0	Retroviridae	eukaryote
v09fb	6202	6	2	0	Medium-quality	72.75	0	unclassified	unclassified

v09fc	5445	3	1	0	Medium-quality	50.76	0	Retroviridae	eukaryote
v09fd	34387	25	3	0	High-quality	92.81	0	unclassified	unclassified
v09fe	11745	18	1	0	High-quality	100	0	unclassified	unclassified
v09ff	10248	8	2	0	High-quality	100	0	Retroviridae	eukaryote
v0a00	17000	12	1	0	Medium-quality	89.69	0	unclassified	unclassified
v0a01	6847	7	1	0	Medium-quality	63.83	0	unclassified	unclassified
v0a02	6188	13	1	0	Medium-quality	57.69	0	unclassified	unclassified
v0a03	7823	12	3	0	Medium-quality	59.85	0	Retroviridae	eukaryote
v0a04	10174	5	1	0	High-quality	100	0	unclassified	unclassified
v0a05	3470	7	1	0	Medium-quality	55.96	0	unclassified	unclassified
v0a06	5067	5	1	0	Medium-quality	59.44	0	Retroviridae	eukaryote
v0a07	7470	7	1	0	Medium-quality	69.64	0	unclassified	unclassified
v0a08	3143	1	1	0	Medium-quality	50.69	0	Retroviridae	eukaryote
v0a09	8051	12	2	0	High-quality	100	0	unclassified	unclassified
v0a0a	9096	7	3	0	High-quality	97.44	0	unclassified	unclassified
v0a0b	7494	11	1	0	Medium-quality	69.87	0	Retroviridae	eukaryote
v0a0c	7570	6	1	0	Medium-quality	88.8	0	unclassified	unclassified
v0a0d	7245	7	1	0	Medium-quality	67.54	0	Retroviridae	eukaryote
v0a0e	5947	8	1	0	High-quality	95.9	0	unclassified	unclassified
v0a0f	6988	5	3	0	Medium-quality	73.78	0	Retroviridae	eukaryote
v0a10	12745	11	2	0	Medium-quality	76.98	0	unclassified	unclassified
v0a11	4641	6	2	0	Medium-quality	74.84	0	unclassified	unclassified
v0a12	11529	5	2	0	Medium-quality	72.48	0	unclassified	unclassified
v0a13	10531	11	1	0	High-quality	98.18	0	Retroviridae	eukaryote
v0a14	8177	6	1	0	High-quality	95.92	0	unclassified	unclassified
v0a15	7104	5	2	0	Medium-quality	54.35	0	unclassified	unclassified
v0a16	11054	4	1	0	High-quality	96.6	0	unclassified	unclassified
v0a17	5824	4	1	0	Medium-quality	54.3	0	Retroviridae	eukaryote
v0a18	17051	5	1	0	High-quality	100	0	unclassified	unclassified
v0a19	10548	10	2	0	Medium-quality	80.7	0	Retroviridae	eukaryote
v0a1a	6240	8	1	0	Medium-quality	73.2	0	unclassified	unclassified
v0a1b	16812	21	3	0	High-quality	100	0	unclassified	unclassified
v0a1c	15774	8	1	0	High-quality	100	0	unclassified	unclassified
v0a1d	23257	20	3	0	Medium-quality	87.17	0	unclassified	unclassified
v0a1e	10205	10	1	0	Medium-quality	53.84	0	unclassified	unclassified
v0a1f	9297	10	2	0	Medium-quality	60.24	0	Retroviridae	eukaryote
v0a20	10200	11	2	0	High-quality	99.89	0	Retroviridae	eukaryote
v0a21	13791	6	2	0	High-quality	98.66	0	Retroviridae	eukaryote
v0a22	7548	5	1	0	Medium-quality	88.54	0	Retroviridae	eukaryote
v0a23	13780	9	1	0	High-quality	100	0	unclassified	unclassified

v0a24	14544	14	3	0	High-quality	100	0	unclassified	unclassified
v0a25	5698	6	1	0	Medium-quality	55.83	0	unclassified	unclassified
v0a26	9830	10	1	0	Medium-quality	51.86	0	unclassified	unclassified
v0a27	3187	3	1	0	Medium-quality	51.39	0	Retroviridae	eukaryote
v0a28	16329	16	2	0	High-quality	100	0	unclassified	unclassified
v0a29	4404	6	1	0	Medium-quality	71.02	0	unclassified	unclassified
v0a2a	4377	5	2	0	Medium-quality	51.34	0	unclassified	unclassified
v0a2b	19471	13	1	0	High-quality	100	0	unclassified	unclassified
v0a2c	8984	4	1	0	High-quality	100	0	Retroviridae	eukaryote
v0a2d	13010	12	2	0	Medium-quality	77.97	0	unclassified	unclassified
v0a2e	10813	10	1	0	High-quality	100	0	unclassified	unclassified
v0a2f	14680	11	1	0	High-quality	100	0	unclassified	unclassified
v0a30	8723	12	1	0	High-quality	100	0	unclassified	unclassified
v0a31	8665	9	1	0	Medium-quality	66.3	0	unclassified	unclassified
v0a32	38005	41	5	0	High-quality	100	0	unclassified	unclassified
v0a33	10030	5	1	0	Medium-quality	52.92	0	unclassified	unclassified
v0a34	22098	27	3	0	High-quality	100	0	unclassified	unclassified
v0a35	8107	12	1	0	High-quality	100	0	unclassified	unclassified
v0a36	10442	7	2	0	High-quality	100	0	unclassified	unclassified
v0a37	17792	10	1	0	High-quality	100	0	Retroviridae	eukaryote
v0a38	17706	10	1	0	High-quality	93.41	0	unclassified	unclassified
v0a39	8204	10	2	0	Medium-quality	62.77	0	Retroviridae	eukaryote
v0a3a	15257	6	1	0	High-quality	100	0	Retroviridae	eukaryote
v0a3b	13712	7	1	0	Medium-quality	72.34	0	unclassified	unclassified
v0a3c	5476	4	1	0	Medium-quality	51.05	0	Retroviridae	eukaryote
v0a3d	5000	4	1	0	Medium-quality	61.59	0	Retroviridae	eukaryote
v0a3e	6243	5	1	0	Medium-quality	58.2	0	unclassified	unclassified
v0a3f	7174	7	1	0	High-quality	100	0	unclassified	unclassified
v0a40	11351	9	2	0	High-quality	100	0	unclassified	unclassified
v0a41	8986	10	2	0	High-quality	100	0	Retroviridae	eukaryote
v0a42	7084	9	1	0	Medium-quality	54.2	0	unclassified	unclassified
v0a43	16461	9	1	0	High-quality	100	0	unclassified	unclassified
v0a44	7136	7	1	0	Medium-quality	66.53	0	Retroviridae	eukaryote
v0a45	9225	6	3	0	Medium-quality	80.81	0	unclassified	unclassified
v0a46	6193	5	1	0	Medium-quality	57.74	0	Retroviridae	eukaryote
v0a47	15590	11	4	0	High-quality	99.15	0	Retroviridae	eukaryote
v0a48	10529	6	1	0	High-quality	92.24	0	Retroviridae	eukaryote
v0a49	3579	2	1	0	Medium-quality	57.72	0	Retroviridae	eukaryote
v0a4a	8602	6	1	0	High-quality	100	0	unclassified	unclassified
v0a4b	4997	2	1	0	Medium-quality	58.62	0	unclassified	unclassified

v0a4c	5476	4	1	0	Medium-quality	64.24	0	Retroviridae	eukaryote
v0a4d	8217	6	1	0	High-quality	96.39	0	unclassified	unclassified
v0a4e	5431	11	3	0	Medium-quality	63.43	0	Retroviridae	eukaryote
v0a4f	12140	8	2	0	High-quality	100	0	unclassified	unclassified
v0a50	8786	14	1	0	Medium-quality	81.91	0	unclassified	unclassified
v0a51	12571	11	1	0	Medium-quality	66.32	0	unclassified	unclassified
v0a52	17087	13	1	0	High-quality	100	0	unclassified	unclassified
v0a53	8106	7	2	0	Medium-quality	52.53	0	Retroviridae	eukaryote
v0a54	10821	6	1	0	High-quality	100	0	Retroviridae	eukaryote
v0a55	6812	6	1	0	Medium-quality	59.67	0	unclassified	unclassified
v0a56	5374	5	2	0	Medium-quality	50.96	0	Retroviridae	eukaryote
v0a57	4971	6	1	0	Medium-quality	80.16	0	unclassified	unclassified
v0a58	3848	4	1	0	Medium-quality	62.05	0	unclassified	unclassified
v0a59	11134	9	4	0	High-quality	99.23	0	Retroviridae	eukaryote
v0a5a	15847	12	2	0	High-quality	100	0	Retroviridae	eukaryote
v0a5b	8138	11	4	0	Medium-quality	76.11	0	unclassified	unclassified
v0a5c	6478	7	1	0	Medium-quality	60.39	0	Retroviridae	eukaryote
v0a5d	11705	13	1	0	High-quality	100	0	unclassified	unclassified
v0a5e	9576	8	1	0	Medium-quality	89.28	0	unclassified	unclassified
v0a5f	8806	7	2	0	Medium-quality	77.14	0	unclassified	unclassified
v0a60	6392	8	2	0	Medium-quality	74.65	0	Retroviridae	eukaryote
v0a61	13910	8	1	0	High-quality	100	0	unclassified	unclassified
v0a62	3653	7	1	0	Medium-quality	58.91	0	unclassified	unclassified
v0a63	6790	10	2	0	Medium-quality	77.5	0	Retroviridae	eukaryote
v0a64	7337	11	1	0	High-quality	100	0	unclassified	unclassified
v0a65	6440	5	1	0	High-quality	100	0	Retroviridae	eukaryote
v0a66	8122	9	1	0	Medium-quality	75.72	0	unclassified	unclassified
v0a67	6854	3	1	0	Medium-quality	60.04	0	unclassified	unclassified
v0a68	14517	18	1	0	Medium-quality	76.59	0	unclassified	unclassified
v0a69	10264	12	5	0	Medium-quality	89.91	0	unclassified	unclassified
v0a6a	11564	10	1	0	Medium-quality	61.01	0	unclassified	unclassified
v0a6b	11281	11	1	0	High-quality	100	0	Retroviridae	eukaryote
v0a6c	3446	6	1	0	Medium-quality	55.57	0	unclassified	unclassified
v0a6d	6873	9	2	0	Medium-quality	80.62	0	unclassified	unclassified
v0a6e	9414	7	2	0	Medium-quality	61.67	0	Retroviridae	eukaryote
v0a6f	6879	4	1	0	Medium-quality	64.13	0	unclassified	unclassified
v0a70	14424	8	1	0	High-quality	100	0	unclassified	unclassified
v0a71	4831	7	1	0	Medium-quality	77.91	0	Retroviridae	eukaryote
v0a72	9132	12	1	0	High-quality	100	0	unclassified	unclassified
v0a73	22379	22	3	0	High-quality	100	0	unclassified	unclassified

v0a74	15068	13	2	0	High-quality	100	0	unclassified	unclassified
v0a75	9941	12	3	0	High-quality	100	0	Retroviridae	eukaryote
v0a76	9642	4	2	0	High-quality	100	0	Retroviridae	eukaryote
v0a77	9930	10	3	0	High-quality	100	0	Retroviridae	eukaryote
v0a78	12345	13	2	1	Medium-quality	65.13	0	unclassified	unclassified
v0a79	6795	3	1	0	Medium-quality	79.71	0	unclassified	unclassified
v0a7a	18516	14	1	0	High-quality	100	0	Retroviridae	eukaryote
v0a7b	6473	8	5	0	Medium-quality	58.32	0	Retroviridae	eukaryote
v0a7c	6192	7	1	0	High-quality	99.85	0	unclassified	unclassified
v0a7d	11177	12	2	0	Medium-quality	58.97	0	unclassified	unclassified
v0a7e	8318	6	1	0	High-quality	96.3	0	Retroviridae	eukaryote
v0a7f	12861	9	1	0	Medium-quality	67.85	0	unclassified	unclassified
v0a80	4517	5	1	0	Medium-quality	72.84	0	Retroviridae	eukaryote
v0a81	28956	24	1	0	High-quality	100	0	unclassified	unclassified
v0a82	11851	13	3	0	High-quality	100	0	unclassified	unclassified
v0a83	10798	14	1	0	Medium-quality	56.97	0	unclassified	unclassified
v0a84	5714	3	1	0	High-quality	92.15	0	unclassified	unclassified
v0a85	9490	6	1	0	Medium-quality	50.07	0	unclassified	unclassified
v0a86	18516	20	3	0	High-quality	100	0	unclassified	unclassified
v0a87	7546	8	4	0	Medium-quality	66.1	0	Retroviridae	eukaryote
v0a88	5542	5	3	0	Medium-quality	63.78	0	Retroviridae	eukaryote
v0a89	5080	4	1	0	Medium-quality	59.59	0	Iridoviridae	eukaryote
v0a8a	10626	7	1	0	High-quality	99.07	0	unclassified	unclassified
v0a8b	10427	5	1	0	High-quality	100	0	Retroviridae	eukaryote
v0a8c	7337	13	3	0	Medium-quality	56.14	0	Retroviridae	eukaryote
v0a8d	5674	4	1	0	Medium-quality	66.56	0	unclassified	unclassified
v0a8e	13869	13	2	0	High-quality	100	0	unclassified	unclassified
v0a8f	14172	13	1	0	High-quality	100	0	unclassified	unclassified
v0a90	20436	17	1	0	High-quality	100	0	unclassified	unclassified
v0a91	10224	6	1	0	Medium-quality	53.94	0	unclassified	unclassified
v0a92	9426	7	1	0	Medium-quality	82.57	0	unclassified	unclassified
v0a93	10719	6	1	0	High-quality	99.93	0	unclassified	unclassified
v0a94	7228	8	1	0	Medium-quality	67.39	0	unclassified	unclassified
v0a95	11316	9	1	0	Medium-quality	86.98	0	Retroviridae	eukaryote
v0a96	3275	4	1	0	Medium-quality	52.81	0	Retroviridae	eukaryote
v0a97	13397	15	2	1	High-quality	100	0	unclassified	unclassified
v0a98	5244	5	1	0	Medium-quality	61.52	0	unclassified	unclassified
v0a99	46415	65	38	1	High-quality	100	0	Siphoviridae	prokaryote
v0a9a	6046	3	1	0	Medium-quality	70.92	0	unclassified	unclassified
v0a9b	14610	15	3	0	Medium-quality	77.08	0	unclassified	unclassified

v0a9c	7375	9	2	0	Medium-quality	68.76	0	unclassified	unclassified
v0a9d	9832	11	2	0	Medium-quality	82.32	0	unclassified	unclassified
v0a9e	3412	4	1	0	Medium-quality	55.02	0	Retroviridae	eukaryote
v0a9f	3166	6	1	0	Medium-quality	51.06	0	unclassified	unclassified
v0aa0	6618	4	2	0	Medium-quality	57.97	0	unclassified	unclassified
v0aa1	4065	4	1	0	Medium-quality	65.55	0	unclassified	unclassified
v0aa2	5162	3	1	0	Medium-quality	50.58	0	Retroviridae	eukaryote
v0aa3	33912	34	1	0	High-quality	100	0	unclassified	unclassified
v0aa4	6049	2	1	0	Medium-quality	59.27	0	unclassified	unclassified
v0aa5	28571	40	26	0	Medium-quality	66.44	0	Myoviridae	prokaryote
v0aa6	3908	4	1	0	Medium-quality	63.02	0	Retroviridae	eukaryote
v0aa7	10282	8	1	0	Medium-quality	54.25	0	unclassified	unclassified
v0aa8	21343	22	2	0	Medium-quality	68.3	0	unclassified	unclassified
v0aa9	18644	14	1	0	High-quality	98.36	0	unclassified	unclassified
v0aaa	5394	8	1	0	Medium-quality	63.27	0	Retroviridae	eukaryote
v0aab	7907	11	1	0	Medium-quality	73.72	0	unclassified	unclassified
v0aac	9935	7	1	0	High-quality	92.62	0	Retroviridae	eukaryote
v0aad	9409	6	1	0	Medium-quality	87.72	0	unclassified	unclassified
v0aae	3351	6	1	0	Medium-quality	54.04	0	unclassified	unclassified
v0aaf	15760	19	1	0	Medium-quality	83.15	0	unclassified	unclassified
v0ab0	17913	21	2	0	High-quality	100	0	Retroviridae	eukaryote
v0ab1	28722	36	1	0	High-quality	100	0	unclassified	unclassified
v0ab2	5852	7	1	0	Medium-quality	54.56	0	Retroviridae	eukaryote
v0ab3	10617	8	2	0	High-quality	100	0	unclassified	unclassified
v0ab4	8884	6	1	0	High-quality	100	0	unclassified	unclassified
v0ab5	22725	28	2	0	High-quality	100	0	unclassified	unclassified
v0ab6	7217	9	1	0	Medium-quality	67.28	0	unclassified	unclassified
v0ab7	23367	12	1	0	High-quality	100	0	unclassified	unclassified
v0ab8	12883	13	1	0	High-quality	100	0	unclassified	unclassified
v0ab9	7123	3	2	0	Medium-quality	62.4	0	unclassified	unclassified
v0aba	10820	9	1	0	Medium-quality	57.08	0	unclassified	unclassified
v0abb	9131	2	1	0	High-quality	100	0	Retroviridae	eukaryote
v0abc	15368	8	1	0	High-quality	100	0	unclassified	unclassified
v0abd	17028	17	4	0	High-quality	100	0	unclassified	unclassified
v0abe	27607	19	7	0	High-quality	100	0	unclassified	unclassified
v0abf	3378	5	1	0	Medium-quality	54.48	0	unclassified	unclassified
v0ac0	4840	4	2	0	Medium-quality	56.53	0	Retroviridae	eukaryote
v0ac1	6105	5	1	0	High-quality	98.45	0	unclassified	unclassified
v0ac2	12258	4	1	0	High-quality	100	0	Retroviridae	eukaryote
v0ac3	7239	12	1	0	Medium-quality	67.49	0	unclassified	unclassified

v0ac4	28771	19	1	0	High-quality	100	0	unclassified	unclassified
v0ac5	12191	14	1	0	High-quality	100	0	unclassified	unclassified
v0ac6	11840	10	1	0	Medium-quality	62.47	0	unclassified	unclassified
v0ac7	12032	10	1	0	Medium-quality	63.48	0	unclassified	unclassified
v0ac8	17222	8	1	0	Medium-quality	52.87	0	unclassified	unclassified
v0ac9	15639	15	5	0	High-quality	100	0	unclassified	unclassified
v0aca	5261	10	1	0	Medium-quality	84.84	0	unclassified	unclassified
v0acb	17089	17	1	0	High-quality	100	0	unclassified	unclassified
v0acc	14850	11	2	0	Medium-quality	78.35	0	unclassified	unclassified
v0acd	4930	8	1	0	Medium-quality	57.83	0	unclassified	unclassified
v0ace	17909	25	3	0	High-quality	100	0	unclassified	unclassified
v0acf	3934	3	1	0	Medium-quality	63.44	0	Retroviridae	eukaryote
v0ad0	12213	9	4	0	Medium-quality	64.43	0	unclassified	unclassified
v0ad1	7814	9	2	0	Medium-quality	68.45	0	unclassified	unclassified
v0ad2	5194	5	3	0	Medium-quality	50.89	0	Retroviridae	eukaryote
v0ad3	3384	5	1	0	Medium-quality	54.57	0	Retroviridae	eukaryote
v0ad4	4439	4	1	0	Medium-quality	52.07	0	unclassified	unclassified
v0ad5	4543	4	1	0	Medium-quality	53.29	0	unclassified	unclassified
v0ad6	12528	21	7	0	High-quality	98.37	0	Retroviridae	eukaryote
v0ad7	11889	10	2	0	High-quality	99.53	0	Retroviridae	eukaryote
v0ad8	4336	8	1	0	Medium-quality	69.92	0	unclassified	unclassified
v0ad9	42871	58	3	0	High-quality	100	0	unclassified	unclassified
v0ada	6713	8	6	0	Medium-quality	50.94	0	Retroviridae	eukaryote
v0adb	21962	31	2	0	High-quality	100	0	unclassified	unclassified
v0adc	6826	8	1	0	High-quality	100	0	unclassified	unclassified
v0add	6583	4	1	0	Medium-quality	57.53	0	unclassified	unclassified
v0ade	26261	18	3	0	High-quality	100	0	unclassified	unclassified
v0adf	5184	7	1	0	Medium-quality	83.6	0	unclassified	unclassified
v0ae0	7715	11	1	0	High-quality	100	0	unclassified	unclassified
v0ae1	9603	8	1	0	High-quality	100	0	unclassified	unclassified
v0ae2	4110	5	1	0	Medium-quality	66.28	0	Retroviridae	eukaryote
v0ae3	15291	16	2	0	High-quality	90.89	0	unclassified	unclassified
v0ae4	7544	9	3	0	Medium-quality	57.25	0	unclassified	unclassified
v0ae5	3743	3	1	0	Medium-quality	60.36	0	Retroviridae	eukaryote
v0ae6	25251	21	1	0	High-quality	100	0	unclassified	unclassified
v0ae7	18378	22	5	0	High-quality	100	0	unclassified	unclassified
v0ae8	4926	7	1	0	Medium-quality	79.44	0	unclassified	unclassified
v0ae9	8815	11	5	0	Medium-quality	77.22	0	unclassified	unclassified
v0aea	3602	3	1	0	Medium-quality	58.09	0	unclassified	unclassified
v0aeb	8521	11	2	0	High-quality	99.96	0	Retroviridae	eukaryote

v0aec	10096	7	1	0	High-quality	100	0	unclassified	unclassified
v0aed	7114	7	1	0	High-quality	100	0	unclassified	unclassified
v0aee	7608	6	1	0	Medium-quality	66.49	0	unclassified	unclassified
v0aef	5519	5	1	0	Medium-quality	51.45	0	Retroviridae	eukaryote
v0af0	11988	16	1	0	Medium-quality	63.25	0	unclassified	unclassified
v0af1	14160	14	4	0	Medium-quality	82.23	0	Retroviridae	eukaryote
v0af2	11015	12	4	0	High-quality	97.25	0	unclassified	unclassified
v0af3	6385	8	1	0	Medium-quality	55.93	0	unclassified	unclassified
v0af4	5767	2	1	0	Medium-quality	67.65	0	unclassified	unclassified
v0af5	3326	5	2	0	Medium-quality	53.64	0	Retroviridae	eukaryote
v0af6	6254	9	2	0	Medium-quality	54.79	0	unclassified	unclassified
v0af7	12493	12	3	0	High-quality	98.32	0	unclassified	unclassified
v0af8	10087	11	3	0	Medium-quality	65.36	0	Retroviridae	eukaryote
v0af9	9607	11	1	0	Medium-quality	84.16	0	unclassified	unclassified
v0afa	7998	9	1	0	Medium-quality	70.06	0	unclassified	unclassified
v0afb	35723	25	2	0	High-quality	100	0	unclassified	unclassified
v0afc	14799	20	1	0	Medium-quality	78.08	0	unclassified	unclassified
v0afd	6599	5	2	0	Medium-quality	50.49	0	unclassified	unclassified
v0afe	9469	18	1	0	Medium-quality	88.28	0	unclassified	unclassified
v0aff	7844	10	3	0	Medium-quality	89.52	0	unclassified	unclassified
v0b00	5496	7	1	0	Medium-quality	51.24	0	Retroviridae	eukaryote
v0b01	12240	12	1	0	Medium-quality	64.58	0	unclassified	unclassified
v0b02	9573	11	3	0	Medium-quality	83.86	0	unclassified	unclassified
v0b03	7226	10	1	0	High-quality	100	0	unclassified	unclassified
v0b04	5114	10	1	0	Medium-quality	82.47	0	unclassified	unclassified
v0b05	10351	13	4	0	High-quality	99.93	0	Retroviridae	eukaryote
v0b06	14366	16	1	0	High-quality	100	0	unclassified	unclassified
v0b07	7985	7	2	0	Medium-quality	69.95	0	unclassified	unclassified
v0b08	15219	15	2	0	High-quality	100	0	unclassified	unclassified
v0b09	3169	5	1	0	Medium-quality	51.1	0	Retroviridae	eukaryote
v0b0a	3191	5	1	0	Medium-quality	51.46	0	Retroviridae	eukaryote
v0b0b	7013	12	1	0	Medium-quality	82.27	0	unclassified	unclassified
v0b0c	5178	8	1	0	Medium-quality	83.5	0	unclassified	unclassified
v0b0d	13819	16	1	0	Medium-quality	72.91	0	unclassified	unclassified
v0b0e	14193	12	6	0	Medium-quality	74.88	0	unclassified	unclassified
v0b0f	12584	8	1	0	High-quality	100	0	unclassified	unclassified
v0b10	7516	8	4	0	Medium-quality	57.51	0	Retroviridae	eukaryote
v0b11	13317	12	5	0	High-quality	100	0	unclassified	unclassified
v0b12	13699	13	3	0	High-quality	100	0	Retroviridae	eukaryote
v0b13	17192	21	5	0	High-quality	100	0	unclassified	unclassified

v0b14	17150	11	2	0	High-quality	100	0	unclassified	unclassified
v0b15	9998	11	4	0	Medium-quality	76.5	0	unclassified	unclassified
v0b16	14082	13	1	0	High-quality	100	0	unclassified	unclassified
v0b17	8082	3	1	0	Medium-quality	70.63	0	unclassified	unclassified
v0b18	4976	8	1	0	Medium-quality	58.37	0	Retroviridae	eukaryote
v0b19	3599	7	1	0	Medium-quality	58.04	0	unclassified	unclassified
v0b1a	8766	11	2	0	High-quality	100	0	unclassified	unclassified
v0b1b	5183	5	2	0	Medium-quality	60.8	0	Retroviridae	eukaryote
v0b1c	8941	8	1	0	Medium-quality	78.32	0	unclassified	unclassified
v0b1d	11067	7	1	0	High-quality	100	0	unclassified	unclassified
v0b1e	11886	8	1	0	High-quality	100	0	unclassified	unclassified
v0b1f	4841	4	1	0	Medium-quality	60.68	0	Retroviridae	eukaryote
v0b20	3974	7	1	0	Medium-quality	64.09	0	unclassified	unclassified
v0b21	11324	15	3	0	High-quality	99.2	0	unclassified	unclassified
v0b22	7545	11	1	0	Medium-quality	70.34	0	unclassified	unclassified
v0b23	5242	3	1	0	Medium-quality	51.36	0	unclassified	unclassified
v0b24	6321	9	2	0	High-quality	100	0	unclassified	unclassified
v0b25	23237	19	1	0	High-quality	100	0	unclassified	unclassified
v0b26	4807	4	1	0	Medium-quality	56.39	0	Retroviridae	eukaryote
v0b27	7728	11	3	0	Medium-quality	72.95	0	unclassified	unclassified
v0b28	26435	32	3	0	High-quality	100	0	unclassified	unclassified
v0b29	7470	7	2	0	Medium-quality	87.63	0	unclassified	unclassified
v0b2a	8435	9	1	0	High-quality	98.95	0	unclassified	unclassified
v0b2b	8422	8	1	0	High-quality	98.8	0	Retroviridae	eukaryote
v0b2c	6221	5	1	0	Medium-quality	54.5	0	unclassified	unclassified
v0b2d	4259	8	1	0	Medium-quality	68.68	0	unclassified	unclassified
v0b2e	7599	13	1	0	Medium-quality	89.14	0	Retroviridae	eukaryote
v0b2f	3202	4	1	0	Medium-quality	51.64	0	unclassified	unclassified
v0b30	5168	3	1	0	Medium-quality	83.34	0	unclassified	unclassified
v0b31	5696	5	1	0	Medium-quality	53.1	0	Retroviridae	eukaryote
v0b32	6389	5	1	0	High-quality	100	0	Retroviridae	eukaryote
v0b33	15303	21	2	0	High-quality	100	0	unclassified	unclassified
v0b34	5300	5	1	0	Medium-quality	55.95	0	unclassified	unclassified
v0b35	14072	14	2	0	High-quality	100	0	unclassified	unclassified
v0b36	4399	5	1	0	Medium-quality	70.94	0	Retroviridae	eukaryote
v0b37	5957	11	1	0	High-quality	96.07	0	unclassified	unclassified
v0b38	3260	6	1	0	Medium-quality	52.57	0	unclassified	unclassified
v0b39	7901	8	1	0	Medium-quality	73.66	0	unclassified	unclassified
v0b3a	4493	7	1	0	Medium-quality	52.71	0	unclassified	unclassified
v0b3b	6325	3	1	0	Medium-quality	66.78	0	Retroviridae	eukaryote

v0b3c	5482	5	1	0	Medium-quality	51.11	0	Retroviridae	eukaryote
v0b3d	8030	3	2	0	Medium-quality	74.86	0	Retroviridae	eukaryote
v0b3e	12955	7	1	0	Medium-quality	68.35	0	unclassified	unclassified
v0b3f	3254	7	1	0	Medium-quality	52.48	0	unclassified	unclassified
v0b40	6819	6	1	0	Medium-quality	79.99	0	unclassified	unclassified
v0b41	5868	4	1	0	Medium-quality	57.5	0	unclassified	unclassified
v0b42	3855	7	1	0	Medium-quality	62.17	0	unclassified	unclassified
v0b43	4176	8	1	0	Medium-quality	67.34	0	unclassified	unclassified
v0b44	6521	7	2	0	Medium-quality	57.13	0	unclassified	unclassified
v0b45	3606	3	1	0	Medium-quality	58.15	0	Retroviridae	eukaryote
v0b46	50335	74	21	0	High-quality	90.99	0	Myoviridae	prokaryote
v0b47	28879	37	11	1	High-quality	100	0	unclassified	unclassified
v0b48	39968	61	8	2	Medium-quality	88.94	0	unclassified	unclassified
v0b49	21262	29	9	1	Medium-quality	50.32	0	Myoviridae	prokaryote
v0b4a	32351	40	24	0	High-quality	91.61	0	Myoviridae	prokaryote
v0b4b	22557	32	26	1	Medium-quality	54.97	0	Siphoviridae	prokaryote
v0b4c	25939	31	11	0	Medium-quality	64.52	0	unclassified	unclassified
v0b4d	41080	50	15	1	High-quality	100	0	unclassified	unclassified
v0b4e	6461	7	4	0	High-quality	100	0	unclassified	unclassified
v0b4f	40503	57	26	0	Complete	100	0	Siphoviridae	prokaryote
v0b50	33118	46	12	0	Complete	100	0	unclassified	unclassified
v0b51	33533	56	25	0	Complete	100	0	Siphoviridae	prokaryote
v0b52	38737	51	8	1	High-quality	100	0	unclassified	unclassified
v0b53	21792	33	16	0	Medium-quality	64.02	0	unclassified	unclassified
v0b54	40266	54	14	2	High-quality	95.18	0	Siphoviridae	prokaryote
v0b55	35490	51	32	1	Medium-quality	87.47	0	Siphoviridae	prokaryote
v0b56	32457	52	20	1	Medium-quality	83.29	0	Siphoviridae	prokaryote
v0b57	20652	41	8	2	High-quality	100	0	unclassified	unclassified
v0b58	8273	16	6	0	Medium-quality	58.89	0	unclassified	unclassified
v0b59	26018	32	25	0	Medium-quality	70.03	0	Siphoviridae	prokaryote
v0b5a	91260	114	42	1	Medium-quality	87.82	0	Myoviridae	prokaryote
v0b5b	6158	9	2	0	Complete	100	0	unclassified	unclassified
v0b5c	5563	6	3	0	Medium-quality	89.61	0	unclassified	unclassified
v0b5d	3795	3	1	0	Complete	100	0	unclassified	unclassified
v0b5e	5553	6	3	0	Medium-quality	89.45	0	unclassified	unclassified
v0b5f	10907	18	5	1	Medium-quality	81.28	0	Siphoviridae	prokaryote
v0b60	23896	21	18	1	Medium-quality	63.87	0	Siphoviridae	prokaryote
v0b61	41843	70	28	0	High-quality	100	0	Myoviridae	prokaryote
v0b62	8427	14	4	1	Medium-quality	64.46	0	Siphoviridae	prokaryote
v0b63	4514	5	1	0	Complete	100	0	unclassified	unclassified

v0b64	6114	9	2	0	Medium-quality	51.69	0	unclassified	unclassified
v0b65	32335	47	19	0	Medium-quality	83.71	0	Siphoviridae	prokaryote
v0b66	38865	60	33	1	Medium-quality	81.95	0	Siphoviridae	prokaryote
v0b67	5043	8	1	0	Complete	100	0	unclassified	unclassified
v0b68	3542	7	1	0	Medium-quality	85.12	0	unclassified	unclassified
v0b69	6118	10	2	0	Complete	100	0	unclassified	unclassified
v0b6a	24501	33	15	1	Medium-quality	60.09	0	Siphoviridae	prokaryote
v0b6b	24132	35	10	0	Medium-quality	59.17	0	Siphoviridae	prokaryote
v0b6c	33100	57	30	0	Medium-quality	83.79	0	Siphoviridae	prokaryote
v0b6d	29309	34	18	0	Medium-quality	73.39	0	Siphoviridae	prokaryote
v0b6e	29647	48	14	0	High-quality	98.17	0	unclassified	unclassified
v0b6f	15056	19	7	1	Complete	100	0	unclassified	unclassified
v0b70	17931	26	8	0	Medium-quality	77.16	0	unclassified	unclassified
v0b71	5615	7	2	0	Medium-quality	86.36	0	unclassified	unclassified
v0b72	34599	48	13	0	Complete	100	0	unclassified	unclassified
v0b73	37740	56	23	1	High-quality	92.06	0	Siphoviridae	prokaryote
v0b74	18665	18	5	0	Medium-quality	54.99	0	unclassified	unclassified
v0b75	28885	37	6	0	Medium-quality	76.34	0	unclassified	unclassified
v0b76	37267	64	24	1	High-quality	96.77	0	unclassified	unclassified
v0b77	10278	14	4	0	Medium-quality	74	0	unclassified	unclassified
v0b78	17876	25	13	1	Medium-quality	50.98	0	Myoviridae	prokaryote
v0b79	47888	72	16	2	High-quality	100	0	unclassified	unclassified
v0b7a	45923	71	19	0	Complete	100	0	unclassified	unclassified
v0b7b	40090	59	22	0	High-quality	99.54	0	Siphoviridae	prokaryote
v0b7c	33151	37	5	1	High-quality	100	0	unclassified	unclassified
v0b7d	21041	22	8	1	Medium-quality	52.51	0	Podoviridae	prokaryote
v0b7e	36512	51	6	2	Medium-quality	73.75	0	unclassified	unclassified
v0b7f	37976	69	21	0	High-quality	94.6	0	Siphoviridae	prokaryote
v0b80	29185	40	16	0	Medium-quality	83.02	0	unclassified	unclassified
v0b81	17662	29	13	1	Medium-quality	55.35	0	Siphoviridae	prokaryote
v0b82	24936	30	17	0	Medium-quality	57.34	0	unclassified	unclassified
v0b83	31346	38	22	3	Medium-quality	66.43	0	Siphoviridae	prokaryote
v0b84	3569	5	1	0	Medium-quality	57.61	0	Mimiviridae	eukaryote
v0b85	26337	35	2	0	Medium-quality	63.4	0	unclassified	unclassified
v0b86	6722	10	3	0	Complete	100	0	Microviridae	prokaryote
v0b87	4889	8	3	0	Medium-quality	75.08	0	unclassified	unclassified
v0b88	5890	6	3	0	Complete	100	0	unclassified	unclassified
v0b89	72341	88	20	3	High-quality	100	0	unclassified	unclassified
v0b8a	62773	91	20	3	High-quality	100	0	Myoviridae	prokaryote
v0b8b	43260	45	6	3	Medium-quality	89.37	0	unclassified	unclassified

v0b8c	32814	52	29	0	Complete	100	0	Siphoviridae	prokaryote
v0b8d	23744	30	23	0	Medium-quality	61.03	0	Siphoviridae	prokaryote
v0b8e	11138	14	2	0	High-quality	93.25	0	Siphoviridae	prokaryote
v0b8f	4211	5	1	0	Complete	100	0	unclassified	unclassified
v0b90	24213	42	32	0	Medium-quality	56.37	0	Myoviridae	prokaryote
v0b91	23796	30	11	2	Medium-quality	60.93	0	Podoviridae	prokaryote
v0b92	32866	48	13	2	High-quality	100	0	unclassified	unclassified
v0b93	26231	33	20	1	Medium-quality	63.76	0	Siphoviridae	prokaryote
v0b94	38840	63	31	1	Complete	100	0	Siphoviridae	prokaryote
v0b95	2130	3	1	0	Medium-quality	51.18	0	unclassified	unclassified
v0b96	19219	24	22	0	Medium-quality	57.66	0	Siphoviridae	prokaryote
v0b97	27031	43	19	0	Medium-quality	62.04	0	Siphoviridae	prokaryote
v0b98	27609	32	23	0	Medium-quality	52.88	0	Siphoviridae	prokaryote
v0b99	25557	35	30	0	Medium-quality	74.8	0	Siphoviridae	prokaryote
v0b9a	34818	54	15	2	High-quality	100	0	unclassified	unclassified
v0b9b	22946	34	23	0	Medium-quality	68.9	0	Siphoviridae	prokaryote
v0b9c	22930	25	21	0	Medium-quality	52.62	0	Siphoviridae	prokaryote
v0b9d	44221	52	32	1	High-quality	100	0	Siphoviridae	prokaryote
v0b9e	4922	7	1	0	High-quality	100	0	unclassified	unclassified
v0b9f	6234	10	3	0	Complete	100	0	unclassified	unclassified
v0ba0	10999	11	2	0	High-quality	92.09	0	unclassified	unclassified
v0ba1	43441	51	23	4	High-quality	93.22	0	Siphoviridae	prokaryote
v0ba2	12952	13	2	0	High-quality	100	0	unclassified	unclassified
v0ba3	27639	40	18	0	Medium-quality	70.16	0	Siphoviridae	prokaryote
v0ba4	6640	8	3	0	High-quality	100	0	unclassified	unclassified
v0ba5	31053	30	18	0	Medium-quality	65.59	0	Siphoviridae	prokaryote
v0ba6	51003	74	19	1	High-quality	97.94	0	Myoviridae	prokaryote
v0ba7	31485	41	21	0	Medium-quality	52.32	0	Siphoviridae	prokaryote
v0ba8	5127	9	2	0	Medium-quality	86.6	0	unclassified	unclassified
v0ba9	13294	15	4	0	High-quality	100	0	Podoviridae	prokaryote
v0baa	5383	8	2	0	High-quality	91.04	0	unclassified	unclassified
v0bab	5636	9	6	0	High-quality	100	0	Microviridae	prokaryote
v0bac	6137	9	2	0	Complete	100	0	unclassified	unclassified
v0bad	61348	81	19	0	Complete	100	0	unclassified	unclassified
v0bae	5390	10	7	0	Complete	100	0	Microviridae	prokaryote
v0baf	12797	13	1	0	Medium-quality	69.33	0	unclassified	unclassified
v0bb0	63891	59	21	6	High-quality	100	0	Siphoviridae	prokaryote
v0bb1	31981	38	14	0	Medium-quality	78.29	0	Siphoviridae	prokaryote
v0bb2	77910	73	8	6	Complete	100	0	unclassified	unclassified
v0bb3	28309	34	20	1	Medium-quality	68.68	0	Siphoviridae	prokaryote

v0bb4	4357	5	1	0	High-quality	100	0	unclassified	unclassified
v0bb5	3771	5	1	0	Complete	100	0	unclassified	unclassified
v0bb6	39658	55	25	0	Medium-quality	65.87	0	Siphoviridae	prokaryote
v0bb7	24569	28	24	0	Medium-quality	62.3	0	Siphoviridae	prokaryote
v0bb8	48433	63	11	1	Complete	100	0	unclassified	unclassified
v0bb9	35253	54	34	0	High-quality	100	0	Siphoviridae	prokaryote
v0bba	60249	68	16	0	High-quality	90.53	0	unclassified	unclassified
v0bbb	23602	25	13	0	Medium-quality	57.28	0	Siphoviridae	prokaryote
v0bbc	56280	94	14	2	Complete	100	0	unclassified	unclassified
v0bbd	22046	32	4	1	Medium-quality	58.35	0	unclassified	unclassified
v0bbe	39987	59	21	0	High-quality	100	0	Siphoviridae	prokaryote
v0bbf	6423	2	1	0	High-quality	97.58	0	Metaviridae	eukaryote
v0bc0	6169	1	1	0	High-quality	95.92	0	Metaviridae	eukaryote
v0bc1	45884	66	40	0	High-quality	100	0	Siphoviridae	prokaryote
v0bc2	11720	9	1	0	High-quality	100	0	unclassified	unclassified
v0bc3	12182	3	1	0	High-quality	100	0	unclassified	unclassified
v0bc4	5733	2	1	0	Medium-quality	54.58	0	unclassified	unclassified
v0bc5	28315	39	21	1	Medium-quality	65.89	0	Siphoviridae	prokaryote
v0bc6	40377	58	19	1	High-quality	100	0	unclassified	unclassified
v0bc7	22959	21	1	0	High-quality	100	0	unclassified	unclassified
v0bc8	40730	61	29	1	Complete	100	0	Siphoviridae	prokaryote
v0bc9	4639	1	1	0	Medium-quality	70.43	0	Metaviridae	eukaryote
v0bca	14063	22	4	1	Medium-quality	61.73	0	unclassified	unclassified
v0bcb	14184	6	1	0	Complete	100	0	unclassified	unclassified
v0bcc	5076	9	5	0	High-quality	100	0	Microviridae	prokaryote
v0bcd	30512	45	11	1	Complete	100	0	Podoviridae	prokaryote
v0bce	6681	8	3	0	High-quality	100	0	unclassified	unclassified
v0bcf	9714	15	9	0	High-quality	100	0	Microviridae	prokaryote
v0bd0	5972	8	3	0	High-quality	100	0	unclassified	unclassified
v0bd1	12110	16	6	0	High-quality	100	0	unclassified	unclassified
v0bd2	3696	3	2	0	Medium-quality	60.59	0	unclassified	unclassified
v0bd3	3053	6	3	0	Medium-quality	56.55	0	unclassified	unclassified
v0bd4	3716	4	2	0	Medium-quality	60.84	0	unclassified	unclassified
v0bd5	37775	55	20	2	Medium-quality	84.35	0	Myoviridae	prokaryote
v0bd6	106220	166	29	1	High-quality	100	0	Podoviridae_crAss-like	prokaryote
v0bd7	44412	48	27	3	Medium-quality	86.21	5.93	Siphoviridae	prokaryote
v0bd8	51134	54	7	6	Medium-quality	61.34	27.1	unclassified	unclassified
v0bd9	77462	111	21	17	High-quality	96.28	29.99	Myoviridae	prokaryote
v0bda	18157	23	7	2	Medium-quality	82.77	21.33	unclassified	unclassified
v0bdb	67475	70	18	14	High-quality	100	29.31	unclassified	unclassified

v0bdc	92106	135	12	4	High-quality	100	2.28	Quimbyviridae	prokaryote
v0bdd	51268	73	20	7	Complete	100	23.44	Siphoviridae	prokaryote
v0bde	56445	90	19	4	Medium-quality	79.55	10.94	Myoviridae	prokaryote
v0bdf	48479	71	16	12	Medium-quality	50.02	22.58	unclassified	unclassified
v0be0	53588	72	26	6	High-quality	95.85	25.7	Myoviridae	prokaryote
v0be1	87311	105	33	21	Medium-quality	84.22	31.4	unclassified	unclassified
v0be2	83020	96	30	26	Complete	100	52.98	Siphoviridae	prokaryote
v0be3	29254	27	5	2	Medium-quality	50.77	22.97	unclassified	unclassified
v0be4	41149	64	13	5	High-quality	100	13.44	unclassified	unclassified
v0be5	33885	35	8	2	Medium-quality	86.8	2.95	Siphoviridae	prokaryote
v0be6	72551	92	9	4	Medium-quality	79.6	4.41	Quimbyviridae	prokaryote
v0be7	28886	39	12	5	Medium-quality	56.91	20.99	Siphoviridae	prokaryote
v0be8	42257	72	10	2	Medium-quality	51.28	11.97	unclassified	unclassified
v0be9	43061	61	13	7	Medium-quality	58.69	26.29	Myoviridae	prokaryote
v0bea	33771	43	5	3	Medium-quality	75.93	6.75	unclassified	unclassified
v0beb	28225	44	12	3	Medium-quality	60.32	12.8	Siphoviridae	prokaryote
v0bec	42882	51	10	9	Complete	100	45.55	unclassified	unclassified
v0bed	50129	51	13	6	Complete	100	20.69	Siphoviridae	prokaryote
v0bee	70556	68	25	6	Medium-quality	78.28	26.14	Siphoviridae	prokaryote
v0bef	52647	67	11	5	Complete	100	23.38	unclassified	unclassified
v0bf0	47385	56	13	9	High-quality	92.18	22.24	unclassified	unclassified
v0bf1	77793	112	32	4	High-quality	100	20.92	Siphoviridae	prokaryote
v0bf2	67767	88	20	10	High-quality	96.16	28.84	Myoviridae	prokaryote
v0bf3	84492	122	19	11	High-quality	91.01	14.43	unclassified	unclassified
v0bf4	53092	63	17	11	High-quality	90.51	34.64	unclassified	unclassified
v0bf5	47672	83	15	7	High-quality	97.42	22.53	unclassified	unclassified
v0bf6	45472	70	14	5	Medium-quality	62.74	13.77	unclassified	unclassified
v0bf7	60667	87	22	6	High-quality	100	11	unclassified	unclassified
v0bf8	85408	100	35	26	Complete	100	49.27	Siphoviridae	prokaryote
v0bf9	49844	57	13	6	High-quality	100	10.69	unclassified	unclassified
v0bfa	60287	90	28	12	Medium-quality	62.03	24.09	unclassified	unclassified
v0bfb	71271	105	23	10	Complete	100	18.72	unclassified	unclassified
v0bfc	33711	49	13	3	Medium-quality	58.97	9.55	Myoviridae	prokaryote
v0bfd	53088	53	24	5	Medium-quality	88.62	20.22	Siphoviridae	prokaryote
v0bfe	57881	73	29	11	Medium-quality	89.57	32.81	unclassified	unclassified
v0bff	52562	73	23	6	Medium-quality	64.27	33.7	unclassified	unclassified
v0c00	71788	71	17	14	High-quality	97.46	44.48	unclassified	unclassified
v0c01	48317	54	22	11	Medium-quality	84.01	38.89	Siphoviridae	prokaryote
v0c02	61606	78	21	4	Complete	100	18.8	Myoviridae	prokaryote
v0c03	72054	100	23	14	Complete	100	33.87	Myoviridae	prokaryote

v0c04	65383	85	13	10	Medium-quality	72.26	17.86	unclassified	unclassified
v0c05	58187	76	20	14	Medium-quality	73.61	42.99	Myoviridae	prokaryote
v0c06	23767	37	9	3	Medium-quality	50.37	6.96	unclassified	unclassified
v0c07	69799	74	24	21	Complete	100	55.38	Siphoviridae	prokaryote
v0c08	46927	60	12	9	Medium-quality	71.57	34.3	unclassified	unclassified
v0c09	30841	48	6	3	Medium-quality	60.35	15.47	unclassified	unclassified
v0c0a	6585	9	3	1	Medium-quality	78.99	25.3	unclassified	unclassified
v0c0b	57409	80	29	18	Medium-quality	84.15	32.49	Myoviridae	prokaryote
v0c0c	86435	85	22	18	High-quality	97.53	45.86	Siphoviridae	prokaryote
v0c0d	55738	73	45	6	High-quality	100	25.6	Siphoviridae	prokaryote
v0c0e	46498	52	18	3	High-quality	96.64	26.19	Siphoviridae	prokaryote
v0c0f	67004	81	30	25	Complete	100	50.28	Myoviridae	prokaryote
v0c10	67461	71	25	17	High-quality	100	31.16	unclassified	unclassified
v0c11	42446	56	28	3	Medium-quality	81.23	11.93	Siphoviridae	prokaryote
v0c12	59911	64	30	13	High-quality	93.77	18.24	Siphoviridae	prokaryote
v0c13	28038	42	18	3	Medium-quality	56.2	24.85	Siphoviridae	prokaryote
v0c14	91087	122	21	18	Complete	100	40.48	Myoviridae	prokaryote
v0c15	66708	93	23	12	Complete	100	36.41	Myoviridae	prokaryote
v0c16	56748	64	22	16	Medium-quality	74.24	41.38	Siphoviridae	prokaryote
v0c17	55521	59	23	8	Medium-quality	69.25	17.68	Siphoviridae	prokaryote
v0c18	102748	125	18	10	High-quality	92.01	24.25	Quimbyviridae	prokaryote
v0c19	131391	159	34	8	Complete	100	11.64	unclassified	unclassified
v0c1a	66158	83	29	14	High-quality	94.87	39.44	Myoviridae	prokaryote
v0c1b	33455	41	21	3	Medium-quality	52.58	29.09	Siphoviridae	prokaryote
v0c1c	146015	195	28	15	High-quality	93.53	9.84	unclassified	unclassified
v0c1d	166675	179	21	5	Medium-quality	86.57	2.69	unclassified	unclassified
v0c1e	48438	68	12	3	High-quality	99.07	8.07	Quimbyviridae	prokaryote
v0c1f	77962	96	48	10	High-quality	100	6.77	Myoviridae	prokaryote
v0c20	46605	61	15	8	High-quality	100	21.51	Siphoviridae	prokaryote
v0c21	52773	60	18	9	Medium-quality	78.39	27.35	unclassified	unclassified
v0c22	86547	113	29	18	High-quality	93.21	30.43	unclassified	unclassified
v0c23	51023	65	23	6	Medium-quality	71.47	32.9	Siphoviridae	prokaryote
v0c24	43569	58	16	10	High-quality	100	24.15	unclassified	unclassified
v0c25	56745	76	17	11	Medium-quality	86.6	24.67	unclassified	unclassified
v0c26	70136	90	22	14	Complete	100	31.69	Myoviridae	prokaryote
v0c27	62457	78	22	18	Medium-quality	89.21	48.6	unclassified	unclassified
v0c28	44442	52	18	11	Medium-quality	85.06	32.84	Siphoviridae	prokaryote
v0c29	57122	80	28	10	High-quality	92.12	20.31	unclassified	unclassified
v0c2a	85975	97	35	29	Complete	100	50.89	Siphoviridae	prokaryote
v0c2b	82592	100	30	27	High-quality	100	43.08	unclassified	unclassified

v0c2c	48670	58	32	8	Medium-quality	88.11	22.92	Siphoviridae	prokaryote
v0c2d	39515	54	22	4	Medium-quality	86.97	15.45	unclassified	unclassified
v0c2e	42280	55	25	3	Medium-quality	87.97	20.98	Siphoviridae	prokaryote
v0c2f	59571	73	18	13	Medium-quality	86.39	36.7	Siphoviridae	prokaryote
v0c30	40207	57	23	3	Medium-quality	87.73	8.02	Siphoviridae	prokaryote
v0c31	41797	54	19	6	Medium-quality	56.49	36.22	unclassified	unclassified
v0c32	51716	64	12	7	Medium-quality	74.72	14.49	unclassified	unclassified
v0c33	45419	54	27	4	High-quality	96.45	14.3	Siphoviridae	prokaryote
v0c34	38186	50	13	4	Medium-quality	73.4	34.79	Siphoviridae	prokaryote
v0c35	40430	59	23	5	Medium-quality	72.06	17.15	Myoviridae	prokaryote
v0c36	71069	77	13	6	High-quality	100	2.62	unclassified	unclassified
v0c37	46494	53	7	6	Complete	100	30.69	unclassified	unclassified
v0c38	63720	92	29	6	High-quality	93.03	11.36	Siphoviridae	prokaryote
v0c39	56724	79	32	5	High-quality	100	14.06	Siphoviridae	prokaryote
v0c3a	68602	94	22	13	Complete	100	29.45	Myoviridae	prokaryote
v0c3b	60819	76	12	9	Complete	100	14.98	unclassified	unclassified
v0c3c	57722	78	17	14	High-quality	92.71	37.94	Siphoviridae	prokaryote
v0c3d	72720	93	19	17	Complete	100	31.83	Myoviridae	prokaryote
v0c3e	30201	49	16	5	Medium-quality	64.73	29.24	Siphoviridae	prokaryote
v0c3f	50302	72	19	16	Medium-quality	70.85	43.66	unclassified	unclassified
v0c40	47966	62	19	11	Medium-quality	69.47	39.65	unclassified	unclassified
v0c41	57157	71	21	14	Complete	100	43.89	Siphoviridae	prokaryote
v0c42	80060	115	23	15	Complete	100	24.87	unclassified	unclassified
v0c43	59772	86	21	9	Complete	100	19.03	Myoviridae	prokaryote
v0c44	222593	335	39	30	Complete	100	13.89	unclassified	unclassified
v0c45	47303	63	20	4	High-quality	100	7.85	Siphoviridae	prokaryote
v0c46	39114	40	12	4	Medium-quality	83.27	21.87	unclassified	unclassified
v0c47	82864	118	20	15	High-quality	100	19.85	unclassified	unclassified
v0c48	64489	84	16	6	Medium-quality	73.69	6.14	unclassified	unclassified
v0c49	22164	38	12	3	Medium-quality	51.64	11.09	Siphoviridae	prokaryote
v0c4a	43525	55	18	5	Medium-quality	76.47	22.27	Siphoviridae	prokaryote
v0c4b	42752	57	25	8	High-quality	98.75	14.95	Siphoviridae	prokaryote
v0c4c	50846	72	18	10	Complete	100	38.63	unclassified	unclassified
v0c4d	43790	66	18	8	Medium-quality	74.83	35.3	Myoviridae	prokaryote
v0c4e	69857	113	29	3	High-quality	100	6.4	unclassified	unclassified
v0c4f	65408	79	47	14	Medium-quality	89.83	35.36	Siphoviridae	prokaryote
v0c50	101511	110	24	13	High-quality	100	18.82	unclassified	unclassified
v0c51	85550	120	38	9	Complete	100	22.47	Siphoviridae	prokaryote
v0c52	57009	70	11	7	Medium-quality	88	33.96	unclassified	unclassified
v0c53	48360	49	5	4	High-quality	100	7.07	unclassified	unclassified

v0c54	39658	76	22	2	High-quality	96.13	8.31	Siphoviridae	prokaryote
v0c55	37629	54	20	8	Medium-quality	77.2	28.27	Siphoviridae	prokaryote
v0c56	34256	50	10	3	Medium-quality	71.06	20.87	Siphoviridae	prokaryote
v0c57	41660	56	15	6	High-quality	99.58	15.45	Siphoviridae	prokaryote
v0c58	75130	87	31	19	Complete	100	37.48	Myoviridae	prokaryote
v0c59	55111	75	20	14	Medium-quality	83.13	28.87	Myoviridae	prokaryote
v0c5a	25765	38	17	3	Medium-quality	53.89	17.28	Siphoviridae	prokaryote
v0c5b	53326	64	22	4	High-quality	100	6.95	Siphoviridae	prokaryote
v0c5c	78434	101	31	18	Complete	100	27.29	Siphoviridae	prokaryote
v0c5d	93669	114	25	23	Medium-quality	68.16	41.61	unclassified	unclassified
v0c5e	29014	40	15	4	Medium-quality	53.55	20.21	Siphoviridae	prokaryote
v0c5f	34560	47	18	7	Medium-quality	70.09	28.7	Siphoviridae	prokaryote
v0c60	36512	45	14	10	Medium-quality	63.7	39.1	Siphoviridae	prokaryote
v0c61	60217	75	30	18	Complete	100	35.64	Siphoviridae	prokaryote
v0c62	53469	59	22	16	Medium-quality	67.95	53.11	Siphoviridae	prokaryote
v0c63	46069	52	19	8	Medium-quality	50.63	48.86	Siphoviridae	prokaryote
v0c64	56982	73	20	7	Complete	100	17.09	Myoviridae	prokaryote
v0c65	129438	148	31	13	High-quality	98.13	10.46	unclassified	unclassified
v0c66	54461	67	21	12	Medium-quality	83.44	35.7	Siphoviridae	prokaryote
v0c67	41394	67	23	2	High-quality	96.76	12.41	Siphoviridae	prokaryote
v0c68	68198	84	31	14	High-quality	100	22.47	Siphoviridae	prokaryote
v0c69	38270	52	21	2	High-quality	90.14	4.26	Siphoviridae	prokaryote
v0c6a	16261	20	4	3	Medium-quality	81.02	33.44	unclassified	unclassified
v0c6b	51724	55	15	10	Medium-quality	86.83	39.35	unclassified	unclassified
v0c6c	69639	78	26	19	Complete	100	41.29	unclassified	unclassified
v0c6d	65875	56	22	18	Medium-quality	71.14	45.58	Siphoviridae	prokaryote
v0c6e	38764	54	14	9	Medium-quality	79.32	33.38	Siphoviridae	prokaryote
v0c6f	46799	66	23	4	High-quality	100	14.87	Siphoviridae	prokaryote
v0c70	99540	154	40	5	High-quality	100	3.09	Myoviridae	prokaryote
v0c71	83774	108	24	22	Complete	100	37.89	unclassified	unclassified
v0c72	50278	70	31	11	High-quality	93.39	19.06	Myoviridae	prokaryote
v0c73	42626	60	18	8	Medium-quality	68.27	36.46	Myoviridae	prokaryote
v0c74	47666	67	29	5	Medium-quality	59.7	21.8	Myoviridae	prokaryote
v0c75	101759	125	29	23	Complete	100	40.81	Siphoviridae	prokaryote
v0c76	72805	94	21	18	Complete	100	40.72	Myoviridae	prokaryote
v0c77	41702	53	18	4	Medium-quality	85.91	21.03	Siphoviridae	prokaryote
v0c78	77388	104	29	21	Complete	100	32.02	Myoviridae	prokaryote
v0c79	68278	73	22	12	Medium-quality	65.43	54.96	Siphoviridae	prokaryote
v0c7a	29083	37	10	6	Medium-quality	54.9	26.47	Siphoviridae	prokaryote
v0c7b	49613	58	31	12	Complete	100	33.18	Myoviridae	prokaryote

v0c7c	25577	27	6	4	Medium-quality	87.27	38.41	unclassified	unclassified
v0c7d	42105	59	24	3	High-quality	100	0.91	Siphoviridae	prokaryote
v0c7e	51102	64	23	8	Medium-quality	87.53	30.14	Siphoviridae	prokaryote
v0c7f	50084	61	21	11	Medium-quality	74.83	47.89	Siphoviridae	prokaryote
v0c80	54537	67	19	8	High-quality	100	21.46	Siphoviridae	prokaryote
v0c81	32551	39	21	5	High-quality	90.36	11.75	Myoviridae	prokaryote
v0c82	60250	72	13	9	Medium-quality	71.51	23.41	unclassified	unclassified
v0c83	54546	74	21	6	Complete	100	10.11	Myoviridae	prokaryote
v0c84	45024	61	18	10	Medium-quality	84.22	28.49	Siphoviridae	prokaryote
v0c85	62358	81	19	8	High-quality	100	8.99	unclassified	unclassified
v0c86	38168	46	19	14	Medium-quality	56.83	37.56	Siphoviridae	prokaryote
v0c87	61597	67	26	20	Medium-quality	74.54	42.73	Siphoviridae	prokaryote
v0c88	83651	88	23	19	Medium-quality	88.41	50.58	Siphoviridae	prokaryote
v0c89	46351	56	17	15	Medium-quality	55.82	51.46	unclassified	unclassified
v0c8a	65513	84	24	20	Medium-quality	80.54	45.52	Myoviridae	prokaryote
v0c8b	57828	61	28	3	Medium-quality	76.98	12.66	Siphoviridae	prokaryote
v0c8c	45708	56	20	8	High-quality	92.57	15.42	Siphoviridae	prokaryote
v0c8d	66774	69	19	12	Complete	100	35.47	Siphoviridae	prokaryote
v0c8e	55618	61	21	9	Medium-quality	84.08	46.34	Siphoviridae	prokaryote
v0c8f	59975	68	23	19	High-quality	100	41.28	Siphoviridae	prokaryote
v0c90	78059	110	19	16	Complete	100	22.2	unclassified	unclassified
v0c91	88538	117	22	21	Complete	100	37.08	unclassified	unclassified
v0c92	79908	121	35	12	High-quality	100	18.77	Myoviridae	prokaryote
v0c93	54871	78	12	10	High-quality	100	31.72	unclassified	unclassified
v0c94	49378	65	23	10	High-quality	93.25	19.57	Siphoviridae	prokaryote
v0c95	43451	59	12	6	High-quality	100	7.46	Siphoviridae	prokaryote
v0c96	40557	52	10	7	Medium-quality	74.08	33.73	Myoviridae	prokaryote
v0c97	58663	67	20	7	High-quality	100	9.12	Siphoviridae	prokaryote
v0c98	55287	59	21	10	Medium-quality	54.8	43.3	Siphoviridae	prokaryote
v0c99	74842	104	22	9	High-quality	97.28	13.33	unclassified	unclassified
v0c9a	60276	74	20	13	High-quality	93.61	37.64	Siphoviridae	prokaryote
v0c9b	83567	122	21	14	Medium-quality	78.36	41.6	unclassified	unclassified
v0c9c	71310	86	22	10	High-quality	100	17.49	Myoviridae	prokaryote
v0c9d	54934	79	18	4	High-quality	96.9	8.27	Myoviridae	prokaryote
v0c9e	58734	73	27	12	Medium-quality	61.46	49.96	Myoviridae	prokaryote
v0c9f	29039	38	22	2	Medium-quality	59.33	4.88	unclassified	unclassified
v0ca0	14447	20	5	4	Medium-quality	51.41	58.12	unclassified	unclassified
v0ca1	44836	58	18	6	High-quality	97.74	20.26	Siphoviridae	prokaryote
v0ca2	44318	62	17	7	Medium-quality	87.64	33.54	unclassified	unclassified
v0ca3	41805	62	19	4	High-quality	100	2.11	Siphoviridae	prokaryote

v0ca4	45717	59	16	3	High-quality	98.37	14.36	unclassified	unclassified
v0ca5	58823	85	19	13	High-quality	92.12	21.5	unclassified	unclassified
v0ca6	58071	71	30	23	Complete	100	45.08	Myoviridae	prokaryote
v0ca7	43539	43	8	3	High-quality	94.13	18.03	Siphoviridae	prokaryote
v0ca8	209908	247	43	39	High-quality	95.63	26.73	unclassified	unclassified
v0ca9	75494	93	24	9	Complete	100	23.47	unclassified	unclassified
v0caa	49535	66	11	6	High-quality	100	23.08	unclassified	unclassified
v0cab	77644	83	27	25	Complete	100	50.37	Siphoviridae	prokaryote
v0cac	71584	93	20	18	High-quality	93.74	28.26	Myoviridae	prokaryote
v0cad	184005	276	37	36	Medium-quality	84.66	24.16	unclassified	unclassified
v0cae	54228	59	30	9	Medium-quality	61.02	48.85	Siphoviridae	prokaryote
v0caf	115702	147	24	5	High-quality	99.35	6.61	Siphoviridae	prokaryote
v0cb0	55336	60	18	12	Medium-quality	76.61	44.32	unclassified	unclassified
v0cb1	43380	62	12	4	Medium-quality	70.86	26.3	unclassified	unclassified
v0cb2	56495	79	23	4	Medium-quality	87.77	15.82	Myoviridae	prokaryote
v0cb3	38839	62	19	4	Medium-quality	87.2	12.27	unclassified	unclassified
v0cb4	52737	74	27	7	High-quality	100	27.26	Siphoviridae	prokaryote
v0cb5	211441	240	45	19	Complete	100	18.46	unclassified	unclassified
v0cb6	44464	64	17	11	Medium-quality	78.49	29.68	unclassified	unclassified
v0cb7	93418	96	25	23	Medium-quality	83.66	63.86	unclassified	unclassified
v0cb8	62289	89	21	19	Medium-quality	84.75	33.48	unclassified	unclassified
v0cb9	35988	48	29	7	Medium-quality	73.71	19.23	Siphoviridae	prokaryote
v0cba	59930	76	21	20	Complete	100	46.77	Siphoviridae	prokaryote
v0cbb	66094	86	26	19	Complete	100	52.28	Siphoviridae	prokaryote
v0cbc	44086	49	19	12	Medium-quality	56.97	44.33	Siphoviridae	prokaryote
v0cbd	33218	43	18	2	Medium-quality	86.84	8.31	Myoviridae	prokaryote
v0cbe	35664	38	22	6	Medium-quality	80.2	20.36	Myoviridae	prokaryote
v0cbf	48154	62	16	10	Complete	100	30.42	Siphoviridae	prokaryote
v0cc0	17044	27	9	5	High-quality	100	20.7	Siphoviridae	prokaryote
v0cc1	36153	40	30	4	Medium-quality	83.98	12.91	Siphoviridae	prokaryote
v0cc2	40744	53	22	6	Medium-quality	61.73	30.27	Myoviridae	prokaryote
v0cc3	38283	52	31	2	High-quality	100	14.55	Siphoviridae	prokaryote
v0cc4	43533	48	12	7	Medium-quality	81.85	33.07	unclassified	unclassified
v0cc5	79539	97	16	9	High-quality	100	23.27	unclassified	unclassified
v0cc6	64301	72	17	16	High-quality	100	39.1	unclassified	unclassified
v0cc7	46162	59	20	4	High-quality	100	12.81	Siphoviridae	prokaryote
v0cc8	64672	72	17	16	Medium-quality	80.61	47.99	Siphoviridae	prokaryote
v0cc9	30185	33	12	8	Medium-quality	50.91	32.87	unclassified	unclassified
v0cca	59017	86	17	15	High-quality	100	32.12	unclassified	unclassified
v0ccb	107017	178	40	6	High-quality	100	6.54	Myoviridae	prokaryote

v0ccc	40568	44	12	7	Medium-quality	81.01	16.37	unclassified	unclassified
v0ccd	41653	50	18	13	Medium-quality	56.06	53.01	Siphoviridae	prokaryote
v0cce	23041	28	14	2	Medium-quality	53.5	18.5	Siphoviridae	prokaryote
v0ccf	65702	87	29	22	Medium-quality	56.81	45.09	Myoviridae	prokaryote
v0cd0	75581	98	36	25	Complete	100	47.38	Siphoviridae	prokaryote
v0cd1	45942	64	23	14	Medium-quality	83.4	28.39	Siphoviridae	prokaryote
v0cd2	47342	51	17	15	Medium-quality	50.92	54.88	Siphoviridae	prokaryote
v0cd3	99698	128	25	19	Medium-quality	82.12	48.44	unclassified	unclassified
v0cd4	100199	114	30	26	Medium-quality	76.1	42.57	Siphoviridae	prokaryote
v0cd5	46713	71	34	15	Medium-quality	58.99	40.09	Siphoviridae	prokaryote
v0cd6	50440	57	24	20	Medium-quality	74.51	53.27	Myoviridae	prokaryote
v0cd7	78954	95	21	16	High-quality	100	27.09	Myoviridae	prokaryote
v0cd8	35391	48	16	4	High-quality	91.05	11.17	Siphoviridae	prokaryote
v0cd9	30506	38	16	4	Medium-quality	59.83	25.74	unclassified	unclassified
v0cda	59597	71	16	15	Complete	100	34.73	Siphoviridae	prokaryote
v0cdb	52499	64	25	15	Medium-quality	72.29	38.64	unclassified	unclassified
v0cdc	62559	82	20	2	High-quality	93.65	9.24	unclassified	unclassified
v0cdd	46778	67	18	8	High-quality	90.33	27.68	Siphoviridae	prokaryote
v0cde	36355	56	29	2	Medium-quality	79.16	15.29	Siphoviridae	prokaryote
v0cdf	64963	71	22	6	High-quality	100	26.5	Siphoviridae	prokaryote
v0ce0	53118	75	19	11	Medium-quality	58.36	35.13	Myoviridae	prokaryote
v0ce1	42726	75	39	2	Medium-quality	71.75	9.77	Siphoviridae	prokaryote
v0ce2	72417	83	31	24	High-quality	98.75	49.41	Myoviridae	prokaryote
v0ce3	67326	102	28	19	Complete	100	45.89	Siphoviridae	prokaryote
v0ce4	72255	89	18	9	High-quality	100	20.26	Myoviridae	prokaryote
v0ce5	68252	80	29	23	Medium-quality	74.61	49.76	Siphoviridae	prokaryote
v0ce6	18561	24	17	2	Medium-quality	51.35	11.82	Myoviridae	prokaryote
v0ce7	58597	61	22	13	Complete	100	49.77	Siphoviridae	prokaryote
v0ce8	50875	69	37	6	High-quality	100	17.65	Siphoviridae	prokaryote
v0ce9	44144	52	33	10	High-quality	98.7	20.85	Myoviridae	prokaryote
v0cea	38808	54	21	10	Medium-quality	60.93	32.98	Siphoviridae	prokaryote
v0ceb	45215	64	18	11	Medium-quality	68.46	39.48	Siphoviridae	prokaryote
v0cec	81352	99	29	25	Complete	100	55.24	Siphoviridae	prokaryote
v0ced	50524	80	25	5	High-quality	100	14.83	Siphoviridae	prokaryote
v0cee	65991	83	33	18	Medium-quality	67.94	33.03	Myoviridae	prokaryote
v0cef	61039	76	19	12	Medium-quality	50.2	52.05	unclassified	unclassified
v0cf0	35241	54	15	3	High-quality	100	8.23	unclassified	unclassified
v0cf1	40015	59	15	4	Medium-quality	61.92	21.32	unclassified	unclassified
v0cf2	26491	36	22	4	Medium-quality	55.21	20.51	Siphoviridae	prokaryote
v0cf3	39615	59	17	12	Medium-quality	73.23	30.99	unclassified	unclassified

v0cf4	69040	88	37	24	Complete	100	48.97	Siphoviridae	prokaryote
v0cf5	10504	14	6	2	Medium-quality	50.76	32.37	unclassified	unclassified
v0cf6	48301	65	18	5	High-quality	100	18.92	Siphoviridae	prokaryote
v0cf7	37216	53	32	5	Medium-quality	85.91	17.51	Siphoviridae	prokaryote
v0cf8	32191	39	17	8	Medium-quality	54.16	36.57	unclassified	unclassified
v0cf9	55976	61	24	11	Complete	100	43	Podoviridae	prokaryote
v0cfa	54421	67	20	11	High-quality	91.38	31.94	unclassified	unclassified
v0cfb	164000	212	61	38	Complete	100	30.84	Siphoviridae	prokaryote
v0cfc	33464	51	11	10	Medium-quality	55.11	38.43	unclassified	unclassified
v0cfd	63126	100	26	3	Medium-quality	84.44	4.83	Myoviridae	prokaryote
v0cfe	50298	75	31	11	High-quality	98.52	27.34	Myoviridae	prokaryote
v0cff	75695	90	42	21	High-quality	100	27.25	Siphoviridae	prokaryote
v0d00	31393	35	19	4	Medium-quality	73.32	24.46	Myoviridae	prokaryote
v0d01	136200	215	26	6	High-quality	99.68	2.26	unclassified	unclassified
v0d02	95830	96	29	13	High-quality	100	26.07	Siphoviridae	prokaryote
v0d03	70256	77	15	9	High-quality	100	7.4	unclassified	unclassified
v0d04	63845	85	19	16	High-quality	98.22	39.88	unclassified	unclassified
v0d05	55998	66	21	12	Complete	100	35.54	Siphoviridae	prokaryote
v0d06	57955	69	14	11	Medium-quality	74.33	27.98	unclassified	unclassified
v0d07	71843	84	21	14	Complete	100	32.28	Myoviridae	prokaryote
v0d08	71089	92	20	15	Complete	100	37.96	Myoviridae	prokaryote
v0d09	43694	66	14	8	High-quality	91.86	23.65	unclassified	unclassified
v0d0a	42252	55	22	7	Medium-quality	75.22	19.79	Siphoviridae	prokaryote
v0d0b	36031	52	32	2	High-quality	92.93	5.68	Siphoviridae	prokaryote
v0d0c	48890	67	15	9	Medium-quality	78.49	39.23	unclassified	unclassified
v0d0d	55366	77	37	12	Complete	100	30.06	Siphoviridae	prokaryote
v0d0e	33359	41	13	7	Medium-quality	50.4	39.69	unclassified	unclassified
v0d0f	71929	92	25	19	Medium-quality	77.35	52.67	Myoviridae	prokaryote
v0d10	45706	62	20	7	High-quality	100	21.26	Siphoviridae	prokaryote
v0d11	43580	67	11	10	High-quality	100	8.46	unclassified	unclassified
v0d12	32080	46	9	3	Medium-quality	50.16	34.66	Siphoviridae	prokaryote
v0d13	42585	61	38	4	Complete	100	13.39	Siphoviridae	prokaryote
v0d14	41438	65	43	3	High-quality	100	5.4	Siphoviridae	prokaryote
v0d15	35893	54	35	3	High-quality	100	6.84	Siphoviridae	prokaryote
v0d16	45958	70	41	7	High-quality	100	15.49	Siphoviridae	prokaryote
v0d17	19944	25	6	5	Complete	100	18.79	Siphoviridae	prokaryote
v0d18	38195	52	19	4	High-quality	94.37	10.26	unclassified	unclassified
v0d19	52616	71	20	8	High-quality	92.64	31.27	Siphoviridae	prokaryote
v0d1a	80767	94	39	24	High-quality	100	45.93	Podoviridae	prokaryote
v0d1b	64529	77	30	22	Complete	100	48.07	Myoviridae	prokaryote

v0d1c	47841	64	32	8	Medium-quality	88.81	14.54	Siphoviridae	prokaryote
v0d1d	88077	113	47	34	High-quality	98.87	47.28	Siphoviridae	prokaryote
v0d1e	60333	60	25	20	Medium-quality	82.25	51.57	Myoviridae	prokaryote
v0d1f	46560	56	16	13	Medium-quality	71.67	40.92	unclassified	unclassified
v0d20	57273	70	23	15	Medium-quality	56.17	44.58	unclassified	unclassified
v0d21	57171	60	18	15	Medium-quality	50.04	52.54	Siphoviridae	prokaryote
v0d22	50546	80	29	7	Complete	100	24.55	Siphoviridae	prokaryote
v0d23	80370	92	22	21	Medium-quality	61.39	55.94	unclassified	unclassified
v0d24	48909	76	34	6	Medium-quality	59.28	28.04	Siphoviridae	prokaryote
v0d25	61382	88	20	13	High-quality	100	20.43	Myoviridae	prokaryote
v0d26	47649	62	9	8	Medium-quality	69.93	29.79	unclassified	unclassified
v0d27	23591	37	7	3	High-quality	100	22.22	unclassified	unclassified
v0d28	41198	49	23	17	Medium-quality	70.16	43.03	Siphoviridae	prokaryote
v0d29	37396	52	19	2	High-quality	91.4	14.11	Siphoviridae	prokaryote
v0d2a	34619	54	23	8	Medium-quality	71.08	23.49	Siphoviridae	prokaryote
v0d2b	68746	92	19	14	Medium-quality	74.68	43.91	Myoviridae	prokaryote
v0d2c	59143	75	24	13	Complete	100	32.7	Siphoviridae	prokaryote
v0d2d	62527	69	6	5	High-quality	100	3.96	unclassified	unclassified
v0d2e	6215	9	5	1	High-quality	100	7	Microviridae	prokaryote
v0d2f	115915	152	17	13	Medium-quality	64.3	6.94	unclassified	unclassified
v0d30	50473	76	32	7	Medium-quality	81.24	24.52	Myoviridae	prokaryote
v0d31	112167	148	35	32	High-quality	100	43.38	Siphoviridae	prokaryote
v0d32	42757	66	18	5	Medium-quality	72.59	8.36	unclassified	unclassified
v0d33	10597	9	3	2	High-quality	100	42.7	Microviridae	prokaryote
v0d34	61728	82	27	18	Complete	100	31.03	Siphoviridae	prokaryote
v0d35	29205	41	9	6	Medium-quality	55.75	22.26	Siphoviridae	prokaryote
v0d36	41461	49	15	7	Complete	100	24.59	Siphoviridae	prokaryote
v0d37	43859	64	15	3	High-quality	99.69	12.76	unclassified	unclassified
v0d38	45586	62	19	11	Medium-quality	61.97	39.44	unclassified	unclassified
v0d39	71846	108	29	3	High-quality	100	4.03	unclassified	unclassified
v0d3a	41597	64	29	4	Medium-quality	87.2	5.67	Siphoviridae	prokaryote
v0d3b	85938	150	12	7	Medium-quality	62.86	2.49	unclassified	unclassified
v0d3c	66973	78	23	11	Complete	100	41.41	unclassified	unclassified
v0d3d	56195	80	27	17	High-quality	94.51	31.98	Siphoviridae	prokaryote
v0d3e	206726	292	43	32	Complete	100	23.32	unclassified	unclassified
v0d3f	38019	54	31	2	Medium-quality	72.59	12.71	Siphoviridae	prokaryote
v0d40	61307	77	23	13	Complete	100	40.13	Siphoviridae	prokaryote
v0d41	49600	71	13	7	Complete	100	34.04	unclassified	unclassified
v0d42	46176	65	17	8	Medium-quality	61.85	47.26	Siphoviridae	prokaryote
v0d43	95747	131	37	29	Complete	100	38.72	Siphoviridae	prokaryote

v0d44	56066	65	25	10	Medium-quality	71.04	39.96	Siphoviridae	prokaryote
v0d45	47398	54	22	10	High-quality	95.65	26.24	Siphoviridae	prokaryote
v0d46	44394	65	23	9	Medium-quality	66.23	30	Myoviridae	prokaryote
v0d47	26599	38	11	4	Medium-quality	50.35	28.93	Siphoviridae	prokaryote
v0d48	90001	99	35	31	Complete	100	49.73	Siphoviridae	prokaryote
v0d49	46757	65	19	6	Complete	100	32.39	unclassified	unclassified
v0d4a	42653	61	14	7	High-quality	100	19.25	unclassified	unclassified
v0d4b	34653	47	26	6	Medium-quality	67.28	31.67	Myoviridae	prokaryote
v0d4c	21395	33	28	2	Medium-quality	62.99	3.97	Myoviridae	prokaryote
v0d4d	49710	66	35	9	Medium-quality	76.61	35.71	Myoviridae	prokaryote
v0d4e	48234	63	20	7	High-quality	96.26	22.14	Siphoviridae	prokaryote
v0d4f	82627	92	32	28	Medium-quality	78.23	49.82	Siphoviridae	prokaryote
v0d50	48410	55	27	14	Medium-quality	59.19	44.19	Siphoviridae	prokaryote
v0d51	44046	62	21	6	Medium-quality	65.69	18.33	Myoviridae	prokaryote
v0d52	12013	16	7	3	Medium-quality	67.57	22.63	Siphoviridae	prokaryote
v0d53	39999	58	28	6	Medium-quality	81.78	22.34	Siphoviridae	prokaryote
v0d54	45219	59	15	8	Medium-quality	84.93	24.47	unclassified	unclassified
v0d55	73459	82	23	20	High-quality	100	31.63	Siphoviridae	prokaryote
v0d56	39082	56	21	4	Medium-quality	89.86	15.32	Siphoviridae	prokaryote
v0d57	45833	72	20	3	High-quality	100	11.95	Siphoviridae	prokaryote
v0d58	51087	49	12	10	Medium-quality	83.62	32.5	unclassified	unclassified
v0d59	53647	69	30	6	Complete	100	14.52	Myoviridae	prokaryote
v0d5a	72044	87	39	30	Complete	100	46.56	Siphoviridae	prokaryote
v0d5b	26816	32	17	2	Medium-quality	53.4	19.88	Siphoviridae	prokaryote
v0d5c	28520	32	21	4	Medium-quality	68.28	10.48	Siphoviridae	prokaryote
v0d5d	59070	86	36	16	Complete	100	37.01	Siphoviridae	prokaryote
v0d5e	49510	71	17	13	Medium-quality	88.79	36.43	unclassified	unclassified
v0d5f	45431	62	7	6	Medium-quality	84.97	22.57	unclassified	unclassified
v0d60	55724	59	23	19	Medium-quality	62.71	57.87	Siphoviridae	prokaryote
v0d61	55630	62	14	6	Medium-quality	89.95	41.77	unclassified	unclassified
v0d62	77838	100	32	26	High-quality	96.34	46.64	Siphoviridae	prokaryote
v0d63	62571	80	28	18	Medium-quality	84.28	48.48	Siphoviridae	prokaryote
v0d64	80882	89	23	19	Medium-quality	79.38	53.39	Siphoviridae	prokaryote
v0d65	24984	40	16	3	Medium-quality	59.9	15.39	Myoviridae	prokaryote
v0d66	76844	100	29	19	High-quality	100	35.09	unclassified	unclassified
v0d67	65894	79	27	13	Complete	100	27.33	unclassified	unclassified
v0d68	47566	74	10	4	Medium-quality	64.31	18.11	unclassified	unclassified
v0d69	25287	42	11	2	Medium-quality	58.17	6.64	Siphoviridae	prokaryote
v0d6a	37925	63	14	5	Medium-quality	87.29	16.36	Myoviridae	prokaryote
v0d6b	45945	55	24	9	Medium-quality	84.36	35.58	Siphoviridae	prokaryote

v0d6c	85025	109	30	18	Complete	100	30.55	Siphoviridae	prokaryote
v0d6d	105757	115	23	11	High-quality	93.86	26.93	Siphoviridae	prokaryote
v0d6e	50933	72	28	14	High-quality	92.3	28.75	Siphoviridae	prokaryote
v0d6f	30306	37	18	0	Medium-quality	83.01	0	unclassified	unclassified
v0d70	5194	6	1	0	High-quality	100	0	unclassified	unclassified
v0d71	39791	56	24	0	High-quality	100	0	Siphoviridae	prokaryote
v0d72	38412	47	6	0	High-quality	92.6	0	unclassified	unclassified
v0d73	41536	63	26	1	High-quality	97.74	0	Siphoviridae	prokaryote
v0d74	18852	36	8	0	Medium-quality	65.33	0	unclassified	unclassified
v0d75	35512	39	24	0	Medium-quality	58.88	0	Siphoviridae	prokaryote
v0d76	42012	53	27	0	High-quality	94.53	0	Myoviridae	prokaryote
v0d77	33225	32	11	1	High-quality	100	0	unclassified	unclassified
v0d78	80513	129	15	1	High-quality	100	0	unclassified	unclassified
v0d79	45036	45	4	2	High-quality	100	0	unclassified	unclassified
v0d7a	34524	53	12	1	High-quality	90.48	0	Siphoviridae	prokaryote
v0d7b	15111	22	7	0	Medium-quality	89.09	0	Siphoviridae	prokaryote
v0d7c	45041	66	16	1	High-quality	99.18	0	unclassified	unclassified
v0d7d	29291	29	2	0	Medium-quality	72.32	0	unclassified	unclassified
v0d7e	38300	46	4	0	Medium-quality	84.67	0	unclassified	unclassified
v0d7f	35072	57	15	1	Medium-quality	76.51	0	Myoviridae	prokaryote
v0d80	67703	68	6	2	High-quality	100	0	unclassified	unclassified
v0d81	17029	32	8	1	Medium-quality	53.48	0	Siphoviridae	prokaryote
v0d82	38768	50	29	0	High-quality	90.99	0	Siphoviridae	prokaryote
v0d83	40284	65	30	0	Medium-quality	64.58	0	Myoviridae	prokaryote
v0d84	41832	68	22	1	High-quality	100	0	Siphoviridae	prokaryote
v0d85	41632	57	27	0	Medium-quality	88.48	0	Siphoviridae	prokaryote
v0d86	36224	41	12	0	High-quality	92.97	0	unclassified	unclassified
v0d87	30481	48	15	0	Medium-quality	81.29	0	unclassified	unclassified
v0d88	63757	78	16	1	Medium-quality	75.16	0	Quimbyviridae	prokaryote
v0d89	30305	37	19	0	Medium-quality	81.38	0	Siphoviridae	prokaryote
v0d8a	27511	46	9	3	Medium-quality	81.47	15.71	unclassified	unclassified
v0d8b	28579	40	16	1	Medium-quality	70.56	0	Siphoviridae	prokaryote
v0d8c	32293	45	16	0	Medium-quality	79.36	0	Siphoviridae	prokaryote
v0d8d	10779	23	4	0	Medium-quality	72.74	0	unclassified	unclassified
v0d8e	25453	49	17	0	Medium-quality	79.19	0	Siphoviridae	prokaryote
v0d8f	44834	72	43	0	High-quality	100	0	Myoviridae	prokaryote
v0d90	17543	22	12	0	Medium-quality	52.97	0	Siphoviridae	prokaryote
v0d91	33439	52	21	0	High-quality	92.2	0	Siphoviridae	prokaryote
v0d92	46906	75	28	1	Medium-quality	75.11	0	Myoviridae	prokaryote
v0d93	6001	8	1	0	High-quality	100	0	unclassified	unclassified

v0d94	32124	45	18	0	Medium-quality	55.83	0	Myoviridae	prokaryote
v0d95	31468	61	22	0	Medium-quality	85.73	0	unclassified	unclassified
v0d96	39116	51	17	0	Medium-quality	83.99	0	unclassified	unclassified
v0d97	35241	46	32	0	High-quality	100	0	Myoviridae	prokaryote
v0d98	61032	102	31	3	High-quality	99.33	0	Myoviridae	prokaryote
v0d99	24920	40	17	1	Medium-quality	76.51	0	Siphoviridae	prokaryote
v0d9a	35579	54	21	0	High-quality	91.27	0	Siphoviridae	prokaryote
v0d9b	48056	57	22	2	Medium-quality	71.97	0	Siphoviridae	prokaryote
v0d9c	48031	47	8	1	Medium-quality	72.77	0	unclassified	unclassified
v0d9d	22498	27	5	1	Medium-quality	52.89	0	unclassified	unclassified
v0d9e	45326	60	23	0	High-quality	91.8	0	unclassified	unclassified
v0d9f	89321	112	33	5	High-quality	98.67	0	Siphoviridae	prokaryote
v0da0	28706	56	24	0	Medium-quality	85.48	0	Siphoviridae	prokaryote
v0da1	41380	64	30	1	High-quality	91.94	0	Myoviridae	prokaryote
v0da2	41385	50	18	1	High-quality	100	0	Siphoviridae	prokaryote
v0da3	38469	52	3	1	High-quality	100	0	unclassified	unclassified
v0da4	67994	72	7	0	High-quality	100	0	unclassified	unclassified
v0da5	36113	53	25	0	Medium-quality	89.23	0	Siphoviridae	prokaryote
v0da6	56301	67	26	1	High-quality	100	0	Siphoviridae	prokaryote
v0da7	32515	46	21	0	Medium-quality	79.73	0	unclassified	unclassified
v0da8	31390	50	13	2	Medium-quality	75.61	0	Siphoviridae	prokaryote
v0da9	75806	100	13	9	High-quality	100	5.55	unclassified	unclassified
v0daa	42203	59	29	0	Medium-quality	86.17	0	Myoviridae	prokaryote
v0dab	54425	75	32	0	High-quality	100	0	Siphoviridae	prokaryote
v0dac	35923	54	10	0	Medium-quality	55.58	0	unclassified	unclassified
v0dad	31715	39	21	0	Medium-quality	84.34	0	Siphoviridae	prokaryote
v0dae	50643	84	29	3	High-quality	100	0	Siphoviridae	prokaryote
v0daf	43807	62	28	0	High-quality	96.39	0	Siphoviridae	prokaryote
v0db0	37109	58	17	1	High-quality	90.7	0	unclassified	unclassified
v0db1	40684	57	30	0	Medium-quality	88.73	0	Myoviridae	prokaryote
v0db2	37524	52	25	0	High-quality	100	0	Siphoviridae	prokaryote
v0db3	50840	88	14	6	Medium-quality	69.27	0	unclassified	unclassified
v0db4	11538	18	2	0	Medium-quality	70.31	0	unclassified	unclassified
v0db5	26056	36	22	2	Medium-quality	57.74	0	Myoviridae	prokaryote
v0db6	31623	52	13	0	Medium-quality	83.58	0	unclassified	unclassified
v0db7	14717	17	4	1	Medium-quality	51.3	0	unclassified	unclassified
v0db8	34929	49	14	0	Medium-quality	86.12	0	unclassified	unclassified
v0db9	35448	49	14	0	High-quality	92.2	0	unclassified	unclassified
v0dba	137955	186	31	4	Medium-quality	78.37	0	unclassified	unclassified
v0dbb	20487	46	8	0	Medium-quality	51.03	0	unclassified	unclassified

v0dbc	65974	104	29	0	High-quality	100	0	Siphoviridae	prokaryote
v0dbd	16869	16	3	2	Medium-quality	60.51	0	unclassified	unclassified
v0dbe	28623	52	16	0	Medium-quality	57.93	0	unclassified	unclassified
v0dbf	60392	100	18	0	High-quality	100	0	unclassified	unclassified
v0dc0	36936	62	11	4	High-quality	100	0	unclassified	unclassified
v0dc1	30310	48	20	0	Medium-quality	75.33	0	Siphoviridae	prokaryote
v0dc2	60907	88	29	0	High-quality	98.69	0	Siphoviridae	prokaryote
v0dc3	48201	64	16	0	Medium-quality	59.87	0	unclassified	unclassified
v0dc4	33620	49	23	0	High-quality	93.46	0	Siphoviridae	prokaryote
v0dc5	24783	32	20	0	Medium-quality	76.08	0	Myoviridae	prokaryote
v0dc6	135335	201	30	1	High-quality	100	0	unclassified	unclassified
v0dc7	57356	96	18	4	High-quality	100	0	unclassified	unclassified
v0dc8	76778	101	21	11	High-quality	100	29.9	Myoviridae	prokaryote
v0dc9	115008	134	39	12	High-quality	100	40.79	Myoviridae	prokaryote
v0dca	33538	47	20	0	Medium-quality	86.6	0	Siphoviridae	prokaryote
v0dcb	34735	50	28	1	Medium-quality	76.65	0	Myoviridae	prokaryote
v0dcc	37644	64	21	1	High-quality	100	0	Siphoviridae	prokaryote
v0dcd	34689	54	14	3	Medium-quality	86.05	13.17	Siphoviridae	prokaryote
v0dce	49852	66	20	0	High-quality	96.33	0	Myoviridae	prokaryote
v0dcf	36656	50	19	2	Medium-quality	80.7	10.65	Siphoviridae	prokaryote
v0dd0	33167	37	6	0	Medium-quality	63.5	0	unclassified	unclassified
v0dd1	46622	63	28	1	Medium-quality	89.16	0	Myoviridae	prokaryote
v0dd2	25727	47	16	1	Medium-quality	76.29	0	Siphoviridae	prokaryote
v0dd3	36593	60	29	0	High-quality	90.27	0	Siphoviridae	prokaryote
v0dd4	41506	57	20	3	High-quality	100	0	unclassified	unclassified
v0dd5	45749	80	20	1	High-quality	97.31	0	Myoviridae	prokaryote
v0dd6	156930	232	38	8	High-quality	100	0	unclassified	unclassified
v0dd7	31806	50	20	0	Medium-quality	81.42	0	Siphoviridae	prokaryote
v0dd8	55559	94	21	0	High-quality	100	0	unclassified	unclassified
v0dd9	36422	50	20	0	High-quality	96.7	0	Siphoviridae	prokaryote
v0dda	48098	59	21	7	High-quality	95.42	20.1	Siphoviridae	prokaryote
v0ddb	31533	44	22	0	Medium-quality	77.43	0	Myoviridae	prokaryote
v0ddc	25476	31	6	4	Medium-quality	85.83	43.35	unclassified	unclassified
v0ddd	40015	51	28	3	High-quality	100	7.72	Myoviridae	prokaryote
v0dde	23865	18	4	0	Medium-quality	63.03	0	Siphoviridae	prokaryote
v0ddf	47463	69	23	0	High-quality	100	0	unclassified	unclassified
v0de0	42837	72	17	0	High-quality	100	0	unclassified	unclassified
v0de1	20636	22	13	0	Medium-quality	51.42	0	Podoviridae	prokaryote
v0de2	37098	57	22	0	High-quality	100	0	Siphoviridae	prokaryote
v0de3	19992	37	9	0	Medium-quality	55.83	0	Siphoviridae	prokaryote

v0de4	40449	70	12	1	Medium-quality	66.13	0	unclassified	unclassified
v0de5	38097	57	23	2	High-quality	100	0	unclassified	unclassified
v0de6	41452	56	25	4	High-quality	91.78	17.64	Siphoviridae	prokaryote
v0de7	17789	21	3	1	Medium-quality	62.01	0	unclassified	unclassified
v0de8	53290	59	15	1	High-quality	100	0	unclassified	unclassified
v0de9	40887	64	33	0	High-quality	95.75	0	Myoviridae	prokaryote
v0dea	30345	48	22	0	Medium-quality	57.23	0	Siphoviridae	prokaryote
v0deb	14694	18	3	1	High-quality	100	0	Siphoviridae	prokaryote
v0dec	47529	51	7	2	High-quality	98.19	0	unclassified	unclassified
v0ded	51624	52	10	7	Medium-quality	73.86	31.76	unclassified	unclassified
v0dee	38137	62	23	0	High-quality	100	0	Siphoviridae	prokaryote
v0def	32576	52	23	1	Medium-quality	88.17	0	Siphoviridae	prokaryote
v0df0	126845	197	23	4	Medium-quality	73.31	0	unclassified	unclassified
v0df1	32971	49	15	0	Medium-quality	50.81	0	unclassified	unclassified
v0df2	33077	49	16	1	Medium-quality	88.45	0	Siphoviridae	prokaryote
v0df3	41235	69	16	0	High-quality	100	0	unclassified	unclassified
v0df4	19051	27	9	3	Medium-quality	87.83	38.95	Siphoviridae	prokaryote
v0df5	41740	67	38	0	High-quality	91.1	0	Siphoviridae	prokaryote
v0df6	46848	62	34	1	High-quality	100	0	Siphoviridae	prokaryote
v0df7	28462	37	23	0	High-quality	90.13	0	Myoviridae	prokaryote
v0df8	7185	8	1	0	High-quality	100	0	unclassified	unclassified
v0df9	39201	50	32	5	High-quality	100	10.78	Myoviridae	prokaryote
v0dfa	34671	45	27	0	High-quality	100	0	Myoviridae	prokaryote
v0dfb	64202	69	4	2	Medium-quality	70.03	0	unclassified	unclassified
v0dfc	40664	55	40	0	High-quality	100	0	Podoviridae	prokaryote
v0dfd	32896	47	21	0	High-quality	97.19	0	Siphoviridae	prokaryote
v0dfe	37784	50	32	7	Complete	100	17.25	Myoviridae	prokaryote
v0dff	43988	77	14	0	High-quality	100	0	unclassified	unclassified
v0e00	42651	71	42	0	High-quality	100	0	Siphoviridae	prokaryote
v0e01	33089	46	29	0	Medium-quality	89.12	0	Siphoviridae	prokaryote
v0e02	22324	30	19	0	Medium-quality	55.59	0	Myoviridae	prokaryote
v0e03	30826	47	21	0	High-quality	90.79	0	Siphoviridae	prokaryote
v0e04	42351	64	20	0	High-quality	100	0	Siphoviridae	prokaryote
v0e05	38813	57	14	1	High-quality	100	0	unclassified	unclassified
v0e06	44958	47	4	1	High-quality	100	0	unclassified	unclassified
v0e07	38767	54	34	0	High-quality	98.13	0	Siphoviridae	prokaryote
v0e08	36816	57	32	0	High-quality	98.25	0	Siphoviridae	prokaryote
v0e09	36640	59	23	0	High-quality	97.92	0	Siphoviridae	prokaryote
v0e0a	44823	61	32	0	Medium-quality	85.47	0	Siphoviridae	prokaryote
v0e0b	34306	49	13	0	High-quality	100	0	unclassified	unclassified

v0e0c	26854	38	3	0	Medium-quality	59.37	0	unclassified	unclassified
v0e0d	48991	68	15	0	High-quality	100	0	unclassified	unclassified
v0e0e	44827	67	22	1	High-quality	100	0	unclassified	unclassified
v0e0f	34315	50	14	1	Medium-quality	89.15	0	Siphoviridae	prokaryote
v0e10	39983	45	7	4	High-quality	100	0	unclassified	unclassified
v0e11	28907	27	3	1	Medium-quality	69.45	0	unclassified	unclassified
v0e12	47582	60	6	3	Medium-quality	71.34	0	unclassified	unclassified
v0e13	41318	55	36	2	Medium-quality	88.84	0	Siphoviridae	prokaryote
v0e14	31233	40	27	0	High-quality	90.67	0	Myoviridae	prokaryote
v0e15	15030	19	4	3	Medium-quality	52.39	0	unclassified	unclassified
v0e16	48699	70	12	5	High-quality	100	0	unclassified	unclassified
v0e17	6586	9	2	0	Medium-quality	53.22	0	unclassified	unclassified
v0e18	35282	50	28	0	High-quality	90.4	0	Siphoviridae	prokaryote
v0e19	28149	35	18	0	Medium-quality	66.72	0	Siphoviridae	prokaryote
v0e1a	21928	24	4	0	Medium-quality	52.27	0	unclassified	unclassified
v0e1b	37336	49	7	0	High-quality	98.82	0	unclassified	unclassified
v0e1c	43743	47	13	0	High-quality	93.17	0	Siphoviridae	prokaryote
v0e1d	37839	51	14	0	High-quality	100	0	unclassified	unclassified
v0e1e	44314	60	20	1	High-quality	100	0	Siphoviridae	prokaryote
v0e1f	36439	51	30	1	Medium-quality	80.11	0	Myoviridae	prokaryote
v0e20	68488	96	25	2	High-quality	100	0	Myoviridae	prokaryote
v0e21	15820	22	4	3	Medium-quality	55.14	0	unclassified	unclassified
v0e22	16999	26	9	0	High-quality	100	0	Siphoviridae	prokaryote
v0e23	16300	23	8	0	Medium-quality	86.15	0	unclassified	unclassified
v0e24	23079	25	4	2	Medium-quality	80.45	0	Siphoviridae	prokaryote
v0e25	24875	26	6	3	High-quality	100	0	unclassified	unclassified
v0e26	39930	50	17	7	Medium-quality	80.55	17.96	Siphoviridae	prokaryote
v0e27	48686	60	32	0	High-quality	100	0	Siphoviridae	prokaryote
v0e28	36647	49	20	0	High-quality	92.73	0	Siphoviridae	prokaryote
v0e29	30135	40	10	0	Medium-quality	79.48	0	Siphoviridae	prokaryote
v0e2a	19412	26	15	0	Medium-quality	56.1	0	Siphoviridae	prokaryote
v0e2b	37561	53	16	2	High-quality	100	0	unclassified	unclassified
v0e2c	31985	43	36	0	High-quality	100	0	Myoviridae	prokaryote
v0e2d	8633	11	3	1	Medium-quality	72.28	0	unclassified	unclassified
v0e2e	74234	100	16	5	High-quality	100	0	unclassified	unclassified
v0e2f	42289	54	19	0	Medium-quality	56.78	0	Siphoviridae	prokaryote
v0e30	34007	54	21	0	High-quality	97.36	0	unclassified	unclassified
v0e31	26941	32	26	0	Medium-quality	65.27	0	Siphoviridae	prokaryote
v0e32	46091	53	28	0	High-quality	96.23	0	Siphoviridae	prokaryote
v0e33	39192	53	16	0	Medium-quality	82.21	0	Myoviridae	prokaryote

v0e34	58813	65	27	0	High-quality	100	0	Siphoviridae	prokaryote
v0e35	51554	75	16	0	Medium-quality	88.9	0	unclassified	unclassified
v0e36	41809	53	9	0	High-quality	100	0	unclassified	unclassified
v0e37	35438	46	26	1	High-quality	100	0	Myoviridae	prokaryote
v0e38	32337	39	27	0	High-quality	99.27	0	Myoviridae	prokaryote
v0e39	18712	20	10	0	Medium-quality	50.65	0	unclassified	unclassified
v0e3a	6039	7	2	0	Medium-quality	50.56	0	unclassified	unclassified
v0e3b	35737	46	35	4	High-quality	96.1	11.84	Myoviridae	prokaryote
v0e3c	112296	171	36	2	High-quality	100	0	unclassified	unclassified
v0e3d	42895	50	31	4	High-quality	100	6.04	Myoviridae	prokaryote
v0e3e	35749	53	22	0	Medium-quality	71.42	0	unclassified	unclassified
v0e3f	36510	56	19	1	High-quality	91.41	0	Siphoviridae	prokaryote
v0e40	36012	41	23	3	Medium-quality	60.04	22.03	Siphoviridae	prokaryote
v0e41	147322	204	24	14	High-quality	100	0	unclassified	unclassified
v0e42	24117	31	21	2	Medium-quality	70.25	0	Myoviridae	prokaryote
v0e43	36090	39	24	0	High-quality	100	0	Myoviridae	prokaryote
v0e44	31628	38	21	0	Medium-quality	76.29	0	Siphoviridae	prokaryote
v0e45	44910	51	27	6	Medium-quality	89.42	20.18	Podoviridae	prokaryote
v0e46	28372	30	20	0	Medium-quality	70.02	0	Siphoviridae	prokaryote
v0e47	35273	43	31	0	High-quality	100	0	Myoviridae	prokaryote
v0e48	17448	18	2	0	High-quality	100	0	unclassified	unclassified
v0e49	34680	53	32	0	High-quality	98.14	0	Myoviridae	prokaryote
v0e4a	80463	85	26	10	High-quality	100	29.97	Siphoviridae	prokaryote
v0e4b	25606	34	21	0	Medium-quality	58.54	0	Siphoviridae	prokaryote
v0e4c	52186	66	12	3	High-quality	100	0	Siphoviridae	prokaryote
v0e4d	37022	56	16	1	High-quality	100	0	unclassified	unclassified
v0e4e	24667	26	7	1	Medium-quality	62.24	0	unclassified	unclassified
v0e4f	8715	13	5	0	Medium-quality	50.01	0	Siphoviridae	prokaryote
v0e50	92951	105	23	4	High-quality	100	0	Siphoviridae	prokaryote
v0e51	57123	106	22	0	High-quality	93.88	0	unclassified	unclassified
v0e52	35912	47	15	1	High-quality	90.38	0	Siphoviridae	prokaryote
v0e53	34437	43	20	1	High-quality	98.27	0	Siphoviridae	prokaryote
v0e54	39588	72	19	0	High-quality	100	0	unclassified	unclassified
v0e55	47543	46	8	4	High-quality	100	10.85	unclassified	unclassified
v0e56	30474	51	15	1	Medium-quality	85.05	0	unclassified	unclassified
v0e57	29624	38	12	2	Medium-quality	59.62	0	Siphoviridae	prokaryote
v0e58	20158	31	12	0	High-quality	100	0	Siphoviridae	prokaryote
v0e59	40973	57	22	2	Medium-quality	86.35	6.63	Myoviridae	prokaryote
v0e5a	37963	50	27	2	High-quality	100	0	Myoviridae	prokaryote
v0e5b	26892	56	11	0	Medium-quality	59.7	0	unclassified	unclassified

v0e5c	37238	63	22	0	High-quality	100	0	Siphoviridae	prokaryote
v0e5d	42720	50	22	7	High-quality	96.31	18.47	Siphoviridae	prokaryote
v0e5e	41409	54	17	1	High-quality	97.47	0	Siphoviridae	prokaryote
v0e5f	41329	61	23	0	High-quality	97.59	0	Myoviridae	prokaryote
v0e60	198813	295	34	3	High-quality	100	0	unclassified	unclassified
v0e61	35737	53	20	1	High-quality	96.05	0	Siphoviridae	prokaryote
v0e62	32631	51	20	0	Medium-quality	82.19	0	unclassified	unclassified
v0e63	38796	48	9	3	Medium-quality	59.26	0	unclassified	unclassified
v0e64	38177	58	27	0	High-quality	93.12	0	Siphoviridae	prokaryote
v0e65	38362	51	20	4	Medium-quality	86.29	21.33	Siphoviridae	prokaryote
v0e66	79091	98	26	2	High-quality	100	0	Myoviridae	prokaryote
v0e67	14631	20	3	1	High-quality	100	0	Siphoviridae	prokaryote
v0e68	63628	102	37	1	High-quality	100	0	Siphoviridae	prokaryote
v0e69	22976	28	8	3	High-quality	99.2	27.33	unclassified	unclassified
v0e6a	40541	58	10	1	High-quality	99.7	0	unclassified	unclassified
v0e6b	40500	62	24	0	High-quality	100	0	Siphoviridae	prokaryote
v0e6c	37600	55	16	1	High-quality	97.35	0	Siphoviridae	prokaryote
v0e6d	39997	59	12	0	High-quality	90.84	0	unclassified	unclassified
v0e6e	75744	94	11	0	High-quality	100	0	Quimbyviridae	prokaryote
v0e6f	36251	52	18	1	High-quality	93.6	0	unclassified	unclassified
v0e70	40008	61	23	1	High-quality	96.53	0	Siphoviridae	prokaryote
v0e71	30864	38	22	1	Medium-quality	61.5	0	Siphoviridae	prokaryote
v0e72	48027	71	9	2	High-quality	100	0	unclassified	unclassified
v0e73	36187	49	14	2	Medium-quality	56.78	0	unclassified	unclassified
v0e74	33341	61	15	4	High-quality	91.98	0	unclassified	unclassified
v0e75	26427	31	6	0	Medium-quality	65.51	0	unclassified	unclassified
v0e76	7623	9	2	0	Medium-quality	61.18	0	Siphoviridae	prokaryote
v0e77	49403	67	21	0	High-quality	100	0	Myoviridae	prokaryote
v0e78	126510	166	10	5	High-quality	91.77	0	unclassified	unclassified
v0e79	37189	55	21	0	High-quality	100	0	Siphoviridae	prokaryote
v0e7a	33955	53	37	0	Medium-quality	69.69	0	Siphoviridae	prokaryote
v0e7b	36863	45	30	1	High-quality	100	0	Myoviridae	prokaryote
v0e7c	42385	50	20	11	Medium-quality	63.26	24.39	Siphoviridae	prokaryote
v0e7d	43376	62	35	0	Medium-quality	77.93	0	Myoviridae	prokaryote
v0e7e	37273	62	18	0	High-quality	92.12	0	unclassified	unclassified
v0e7f	30727	50	20	0	Medium-quality	75.88	0	Siphoviridae	prokaryote
v0e80	31335	46	34	0	High-quality	95.57	0	Myoviridae	prokaryote
v0e81	40743	67	33	0	High-quality	100	0	Siphoviridae	prokaryote
v0e82	40815	64	17	0	High-quality	100	0	Siphoviridae	prokaryote
v0e83	19952	24	13	0	Medium-quality	55.92	0	Siphoviridae	prokaryote

v0e84	44871	47	22	3	High-quality	95.24	0	Siphoviridae	prokaryote
v0e85	27487	43	15	2	Medium-quality	77.11	0	Siphoviridae	prokaryote
v0e86	53882	77	21	0	Medium-quality	88.1	0	unclassified	unclassified
v0e87	65396	73	20	6	High-quality	100	0	unclassified	unclassified
v0e88	53877	71	20	3	High-quality	100	0	Siphoviridae	prokaryote
v0e89	43361	69	17	0	Medium-quality	89.17	0	Myoviridae	prokaryote
v0e8a	24193	25	20	0	Medium-quality	68.08	0	Siphoviridae	prokaryote
v0e8b	44361	76	19	1	High-quality	100	0	unclassified	unclassified
v0e8c	38728	52	22	5	Medium-quality	85.67	30.63	Siphoviridae	prokaryote
v0e8d	35135	49	25	1	Medium-quality	81.31	0	Siphoviridae	prokaryote
v0e8e	9105	16	6	0	Medium-quality	65.78	0	Siphoviridae	prokaryote
v0e8f	5063	8	1	0	High-quality	100	0	unclassified	unclassified
v0e90	30391	38	22	0	Medium-quality	86.24	0	Siphoviridae	prokaryote
v0e91	24289	35	8	0	High-quality	100	0	unclassified	unclassified
v0e92	41050	51	19	0	High-quality	100	0	Siphoviridae	prokaryote
v0e93	41532	59	33	0	High-quality	97.8	0	Myoviridae	prokaryote
v0e94	41772	60	37	0	High-quality	100	0	Siphoviridae	prokaryote
v0e95	35562	49	23	2	Medium-quality	84.64	11.24	Siphoviridae	prokaryote
v0e96	53748	53	28	1	High-quality	100	0	Siphoviridae	prokaryote
v0e97	36862	64	27	0	Medium-quality	84.1	0	Siphoviridae	prokaryote
v0e98	28597	40	21	0	Medium-quality	75.44	0	Siphoviridae	prokaryote
v0e99	39840	51	25	0	High-quality	93.98	0	Siphoviridae	prokaryote
v0e9a	58460	71	5	10	High-quality	100	0	unclassified	unclassified
v0e9b	56075	62	0	16	High-quality	100	0	unclassified	unclassified
v0e9c	36388	38	2	10	Low-quality	44.52	0	unclassified	unclassified
v0e9d	26657	43	4	5	Medium-quality	65.46	0	unclassified	unclassified
v0e9e	24395	22	0	4	Medium-quality	54.73	0	unclassified	unclassified
v0e9f	52168	41	2	9	High-quality	100	0	unclassified	unclassified
v0ea0	108546	102	3	34	Medium-quality	79.74	0	unclassified	unclassified
v0ea1	51422	72	4	6	Medium-quality	78.18	0	unclassified	unclassified
v0ea2	5431	10	0	0	Medium-quality	88.43	0	Inoviridae	prokaryote
v0ea3	151935	153	7	48	High-quality	95.87	0	unclassified	unclassified
v0ea4	7305	9	0	3	Medium-quality	61.44	0	unclassified	unclassified
v0ea5	44507	46	9	5	High-quality	100	0	unclassified	unclassified
v0ea6	17099	24	6	0	Medium-quality	89.77	0	unclassified	unclassified
v0ea7	148345	205	40	7	High-quality	99.95	0	unclassified	unclassified
v0ea8	103358	140	23	12	High-quality	100	0	Myoviridae	prokaryote
v0ea9	44375	67	19	1	Medium-quality	85.36	0	unclassified	unclassified
v0eaa	38399	43	19	1	High-quality	93.3	0	Myoviridae	prokaryote
v0eab	47654	64	24	0	High-quality	100	0	Siphoviridae	prokaryote

v0eac	49688	57	20	4	High-quality	100	0	Siphoviridae	prokaryote
v0ead	44973	62	24	1	High-quality	100	0	Myoviridae	prokaryote
v0eae	163239	241	34	6	High-quality	100	0	unclassified	unclassified
v0eaf	36365	56	22	0	High-quality	97.38	0	Siphoviridae	prokaryote
v0eb0	6970	7	2	1	Medium-quality	88.83	0	unclassified	unclassified
v0eb1	42575	53	21	0	Medium-quality	84.54	0	unclassified	unclassified
v0eb2	9235	8	3	1	High-quality	100	16.66	unclassified	unclassified
v0eb3	27520	38	24	0	Medium-quality	58.14	0	Siphoviridae	prokaryote
v0eb4	42250	40	26	0	Medium-quality	80.65	0	Siphoviridae	prokaryote
v0eb5	43907	71	15	0	High-quality	97.4	0	unclassified	unclassified
v0eb6	39245	56	23	0	High-quality	93.04	0	Siphoviridae	prokaryote
v0eb7	42666	67	30	0	High-quality	100	0	Siphoviridae	prokaryote
v0eb8	124659	169	31	2	Medium-quality	70.44	0	unclassified	unclassified
v0eb9	39157	64	41	0	High-quality	91.3	0	Myoviridae	prokaryote
v0eba	11581	16	3	0	High-quality	100	0	unclassified	unclassified
v0ebb	43303	57	14	4	High-quality	100	0	unclassified	unclassified
v0ebc	43586	62	40	0	High-quality	94.06	0	Siphoviridae	prokaryote
v0ebd	22806	27	23	0	Medium-quality	59.95	0	Siphoviridae	prokaryote
v0ebe	30936	30	16	0	Medium-quality	64.26	0	unclassified	unclassified
v0ebf	41958	57	10	1	Medium-quality	72.35	0	unclassified	unclassified
v0ec0	62115	75	9	3	Medium-quality	70.58	0	Quimbyviridae	prokaryote
v0ec1	69627	100	29	2	High-quality	100	0	Myoviridae	prokaryote
v0ec2	41946	71	18	1	Medium-quality	80.93	0	unclassified	unclassified
v0ec3	33612	50	14	0	High-quality	98.35	0	unclassified	unclassified
v0ec4	55155	77	17	2	Medium-quality	68.42	0	unclassified	unclassified
v0ec5	78957	80	5	13	High-quality	100	0	unclassified	unclassified
v0ec6	74663	77	1	19	High-quality	100	0	unclassified	unclassified
v0ec7	54456	83	15	7	High-quality	100	0	Myoviridae	prokaryote
v0ec8	32429	37	29	0	Medium-quality	79.94	0	Myoviridae	prokaryote
v0ec9	53101	72	14	3	Medium-quality	89.71	0	unclassified	unclassified
v0eca	42934	73	16	0	High-quality	95.16	0	unclassified	unclassified
v0ecb	30573	45	25	0	Medium-quality	85.14	0	Siphoviridae	prokaryote
v0ecc	38992	51	18	1	Medium-quality	85.96	0	Siphoviridae	prokaryote
v0ecd	64449	100	32	3	High-quality	100	0	Siphoviridae	prokaryote
v0ece	26878	40	10	0	Medium-quality	75.69	0	unclassified	unclassified
v0ecf	43514	33	0	5	High-quality	93.6	0	unclassified	unclassified
v0ed0	61241	71	0	10	Medium-quality	74.93	0	unclassified	unclassified
v0ed1	36429	33	0	8	Medium-quality	82.76	0	unclassified	unclassified
v0ed2	52239	58	4	9	High-quality	100	0	unclassified	unclassified
v0ed3	64043	66	2	17	High-quality	96.3	0	unclassified	unclassified

v0ed4	47994	51	2	13	Medium-quality	50.78	0	unclassified	unclassified
v0ed5	14432	21	2	4	Medium-quality	59.58	0	unclassified	unclassified
v0ed6	37292	59	43	0	High-quality	92.13	0	Podoviridae	prokaryote
v0ed7	35259	46	17	1	High-quality	99.73	0	Siphoviridae	prokaryote
v0ed8	54937	65	8	5	Medium-quality	85.13	0	unclassified	unclassified
v0ed9	43475	61	35	0	High-quality	100	0	Siphoviridae	prokaryote
v0eda	36836	45	25	2	Medium-quality	89.74	0	Siphoviridae	prokaryote
v0edb	35194	49	33	0	High-quality	100	0	Myoviridae	prokaryote
v0edc	20061	25	1	1	High-quality	100	0	unclassified	unclassified
v0edd	9377	12	1	1	Medium-quality	51.06	0	unclassified	unclassified
v0ede	105787	120	12	37	High-quality	99.37	0	unclassified	unclassified
v0edf	69934	73	1	10	Medium-quality	70.45	0	unclassified	unclassified
v0ee0	28661	26	1	8	Low-quality	7.85	0	unclassified	unclassified
v0ee1	8887	14	1	2	High-quality	100	0	unclassified	unclassified
v0ee2	41279	26	1	9	High-quality	91.25	0	unclassified	unclassified
v0ee3	59971	60	1	19	Medium-quality	74.23	0	unclassified	unclassified
v0ee4	56089	59	3	16	Medium-quality	68.05	0	unclassified	unclassified
v0ee5	44624	42	1	14	Medium-quality	81.6	0	unclassified	unclassified
v0ee6	26274	25	0	8	Medium-quality	62.65	0	unclassified	unclassified
v0ee7	62772	64	6	7	High-quality	100	0	unclassified	unclassified
v0ee8	152285	138	6	29	High-quality	100	0	unclassified	unclassified
v0ee9	42801	52	24	1	Medium-quality	72.03	0	Siphoviridae	prokaryote
v0eea	57751	70	12	0	High-quality	100	0	unclassified	unclassified
v0eeb	43504	74	19	0	High-quality	100	0	Siphoviridae	prokaryote
v0eec	32643	47	26	0	Medium-quality	64.79	0	unclassified	unclassified
v0eed	66621	97	22	3	High-quality	100	0	Myoviridae	prokaryote
v0eee	39930	57	16	1	High-quality	100	0	Siphoviridae	prokaryote
v0eef	43572	63	24	2	High-quality	99.7	0	Myoviridae	prokaryote
v0ef0	106567	112	7	14	Medium-quality	83.81	0	unclassified	unclassified
v0ef1	33108	53	30	0	High-quality	94.79	0	Myoviridae	prokaryote
v0ef2	35029	61	26	0	Medium-quality	85.57	0	unclassified	unclassified
v0ef3	49831	73	12	2	High-quality	90.44	0	unclassified	unclassified
v0ef4	39657	45	10	5	Medium-quality	71.39	48.07	unclassified	unclassified
v0ef5	37397	53	25	0	High-quality	92.15	0	Myoviridae	prokaryote
v0ef6	38518	57	17	0	High-quality	99.01	0	unclassified	unclassified
v0ef7	31346	50	22	0	Medium-quality	80.15	0	Siphoviridae	prokaryote
v0ef8	40250	69	19	0	High-quality	100	0	unclassified	unclassified
v0ef9	45229	72	14	0	High-quality	99.22	0	unclassified	unclassified
v0efa	78104	118	17	2	High-quality	98.18	0	unclassified	unclassified
v0efb	65826	97	23	1	High-quality	100	0	unclassified	unclassified

v0efc	59491	54	1	16	High-quality	90.02	0	unclassified	unclassified
v0efd	65429	62	1	17	High-quality	100	0	unclassified	unclassified
v0efe	52404	65	4	7	High-quality	100	0	unclassified	unclassified
v0eff	49452	59	1	17	Medium-quality	77.22	0	unclassified	unclassified
v0f00	32183	41	17	2	Medium-quality	68.53	0	Siphoviridae	prokaryote
v0f01	36583	49	25	1	Medium-quality	80.8	0	Myoviridae	prokaryote
v0f02	101861	173	32	5	High-quality	100	0	Myoviridae	prokaryote
v0f03	35401	42	17	0	High-quality	100	0	Siphoviridae	prokaryote
v0f04	42430	58	8	1	High-quality	100	0	unclassified	unclassified
v0f05	12705	15	0	1	Medium-quality	62	0	unclassified	unclassified
v0f06	31945	38	0	11	Medium-quality	61.3	0	unclassified	unclassified
v0f07	77563	82	2	26	Medium-quality	73.7	0	unclassified	unclassified
v0f08	76720	87	6	8	Complete	100	0	unclassified	unclassified
v0f09	42865	46	4	4	Medium-quality	64.77	0	unclassified	unclassified
v0f0a	61300	82	9	3	Medium-quality	69.37	0	unclassified	unclassified
v0f0b	70318	121	9	1	Medium-quality	82.87	0	unclassified	unclassified
v0f0c	24368	25	3	0	Medium-quality	53.87	0	unclassified	unclassified
v0f0d	38688	53	8	4	Medium-quality	57.16	0	unclassified	unclassified
v0f0e	49054	73	22	0	High-quality	90.53	0	Myoviridae	prokaryote
v0f0f	41788	56	9	1	High-quality	100	0	unclassified	unclassified
v0f10	24848	36	14	0	Medium-quality	59.33	0	Siphoviridae	prokaryote
v0f11	40566	62	18	0	High-quality	100	0	unclassified	unclassified
v0f12	43134	62	17	1	High-quality	100	0	Siphoviridae	prokaryote
v0f13	43672	71	25	0	High-quality	100	0	Myoviridae	prokaryote
v0f14	25086	26	3	2	Medium-quality	61.46	0	unclassified	unclassified
v0f15	95616	88	8	8	High-quality	100	0	unclassified	unclassified
v0f16	70739	73	6	11	High-quality	100	0	unclassified	unclassified
v0f17	31114	25	1	5	Medium-quality	69.26	0	unclassified	unclassified
v0f18	138321	145	6	18	High-quality	100	0	unclassified	unclassified
v0f19	73224	71	1	18	High-quality	100	0	unclassified	unclassified
v0f1a	116921	135	7	31	High-quality	92.54	0	unclassified	unclassified
v0f1b	219396	236	6	32	High-quality	100	0	unclassified	unclassified
v0f1c	46741	66	7	11	Medium-quality	87.97	0	Myoviridae	prokaryote
v0f1d	25872	24	0	7	Medium-quality	55.65	0	unclassified	unclassified
v0f1e	159499	171	2	32	High-quality	99.47	0	unclassified	unclassified
v0f1f	77649	118	43	7	High-quality	100	0	Myoviridae	prokaryote
v0f20	110390	172	54	16	High-quality	100	0	Myoviridae	prokaryote
v0f21	34136	47	21	0	High-quality	95.57	0	Siphoviridae	prokaryote
v0f22	39035	45	15	0	High-quality	100	0	unclassified	unclassified
v0f23	35998	50	12	0	High-quality	93.68	0	unclassified	unclassified

v0f24	65007	104	21	0	High-quality	100	0	unclassified	unclassified
v0f25	28809	45	14	0	Medium-quality	80.61	0	unclassified	unclassified
v0f26	47397	63	18	5	High-quality	92.25	29.97	Siphoviridae	prokaryote
v0f27	77582	101	49	12	High-quality	94.38	57.24	Myoviridae	prokaryote
v0f28	34929	52	26	1	Medium-quality	88.76	0	Siphoviridae	prokaryote
v0f29	39722	50	27	2	High-quality	100	10.68	Siphoviridae	prokaryote
v0f2a	68041	89	12	0	High-quality	100	0	unclassified	unclassified
v0f2b	59984	104	9	3	Medium-quality	53.79	0	unclassified	unclassified
v0f2c	50748	48	4	0	Medium-quality	80.69	0	unclassified	unclassified
v0f2d	42027	51	15	11	Medium-quality	59.08	40.45	Siphoviridae	prokaryote
v0f2e	37096	58	27	0	High-quality	92.17	0	Siphoviridae	prokaryote
v0f2f	30240	46	18	0	Medium-quality	86.35	0	Siphoviridae	prokaryote
v0f30	59679	86	18	0	High-quality	100	0	unclassified	unclassified
v0f31	52400	69	36	0	High-quality	100	0	Siphoviridae	prokaryote
v0f32	24420	38	4	0	Medium-quality	61.57	0	unclassified	unclassified
v0f33	266982	299	18	24	High-quality	93.85	0	unclassified	unclassified
v0f34	177190	191	11	42	High-quality	100	0	unclassified	unclassified
v0f35	45605	61	16	0	High-quality	100	0	unclassified	unclassified
v0f36	50111	71	30	1	High-quality	100	0	unclassified	unclassified
v0f37	38574	56	8	2	High-quality	93.21	0	unclassified	unclassified
v0f38	37765	55	5	7	Medium-quality	79.97	0	unclassified	unclassified
v0f39	42839	41	1	6	Medium-quality	73.88	0	unclassified	unclassified
v0f3a	71101	71	1	18	High-quality	100	0	unclassified	unclassified
v0f3b	9183	14	0	0	Complete	100	0	Inoviridae	prokaryote
v0f3c	45911	64	36	0	High-quality	99.33	0	Siphoviridae	prokaryote
v0f3d	29963	34	10	2	Medium-quality	51.23	0	unclassified	unclassified
v0f3e	37976	40	24	2	High-quality	99.1	0	Myoviridae	prokaryote
v0f3f	35262	49	27	0	Medium-quality	82.66	0	Siphoviridae	prokaryote
v0f40	43368	57	6	1	High-quality	100	0	unclassified	unclassified
v0f41	26768	32	7	1	Medium-quality	53.82	0	unclassified	unclassified
v0f42	30776	46	8	0	Medium-quality	52.23	0	unclassified	unclassified
v0f43	42879	73	19	1	High-quality	100	0	unclassified	unclassified
v0f44	42241	46	20	0	High-quality	100	0	unclassified	unclassified
v0f45	61138	64	13	1	Medium-quality	66.2	0	unclassified	unclassified
v0f46	31338	33	8	0	Medium-quality	82.94	0	unclassified	unclassified
v0f47	48144	69	27	1	High-quality	100	0	Myoviridae	prokaryote
v0f48	162905	254	33	6	High-quality	99.51	0	unclassified	unclassified
v0f49	89264	93	1	28	Medium-quality	56.55	0	unclassified	unclassified
v0f4a	47869	48	3	13	Medium-quality	52.14	0	unclassified	unclassified
v0f4b	61798	60	0	19	Medium-quality	77.34	0	unclassified	unclassified

v0f4c	51018	52	1	1	Medium-quality	51.57	0	unclassified	unclassified
v0f4d	44667	40	1	14	High-quality	100	0	unclassified	unclassified
v0f4e	64304	70	1	18	High-quality	100	0	unclassified	unclassified
v0f4f	41871	40	0	8	Medium-quality	76.56	0	unclassified	unclassified
v0f50	36491	50	15	0	High-quality	92.55	0	Siphoviridae	prokaryote
v0f51	39618	46	19	1	High-quality	96.3	0	Siphoviridae	prokaryote
v0f52	23813	42	17	0	Medium-quality	61.87	0	Siphoviridae	prokaryote
v0f53	27269	31	25	0	Medium-quality	67.62	0	Podoviridae	prokaryote
v0f54	53240	68	29	1	High-quality	100	0	Myoviridae	prokaryote
v0f55	35559	63	20	3	High-quality	97	0	unclassified	unclassified
v0f56	69338	93	26	2	High-quality	100	0	Siphoviridae	prokaryote
v0f57	159802	224	41	5	Medium-quality	83.59	0	unclassified	unclassified
v0f58	72498	93	10	0	High-quality	97.57	0	unclassified	unclassified
v0f59	37038	50	20	2	Medium-quality	89.86	9.45	Siphoviridae	prokaryote
v0f5a	31057	56	22	0	Medium-quality	69.79	0	Myoviridae	prokaryote
v0f5b	29691	48	16	0	High-quality	100	0	Siphoviridae	prokaryote
v0f5c	33340	47	23	0	Medium-quality	68.5	0	unclassified	unclassified
v0f5d	31978	52	26	2	High-quality	94.15	0	Siphoviridae	prokaryote
v0f5e	151748	202	35	4	High-quality	100	0	unclassified	unclassified
v0f5f	36593	57	18	0	High-quality	100	0	unclassified	unclassified
v0f60	16138	21	4	1	Medium-quality	56.25	0	Siphoviridae	prokaryote
v0f61	41677	50	17	1	High-quality	100	0	unclassified	unclassified
v0f62	169142	242	56	2	High-quality	100	0	Myoviridae	prokaryote
v0f63	61617	108	22	3	High-quality	100	0	unclassified	unclassified
v0f64	42438	49	1	15	High-quality	100	0	unclassified	unclassified
v0f65	11561	16	0	1	Complete	100	0	unclassified	unclassified
v0f66	35878	52	16	1	High-quality	94.05	0	Siphoviridae	prokaryote
v0f67	18875	19	1	5	High-quality	100	0	unclassified	unclassified
v0f68	171916	236	46	5	High-quality	100	0	unclassified	unclassified
v0f69	35152	57	18	2	High-quality	97.47	0	Siphoviridae	prokaryote
v0f6a	56068	85	26	1	High-quality	100	0	Myoviridae	prokaryote
v0f6b	153983	242	41	3	High-quality	96.65	0	unclassified	unclassified
v0f6c	42842	49	0	17	Medium-quality	54.01	0	unclassified	unclassified
v0f6d	50671	70	19	1	High-quality	100	0	Myoviridae	prokaryote
v0f6e	21168	28	7	0	Medium-quality	53.12	0	Siphoviridae	prokaryote
v0f6f	79654	107	31	1	High-quality	100	0	Siphoviridae	prokaryote
v0f70	92966	105	1	24	High-quality	100	0	unclassified	unclassified
v0f71	23670	31	19	0	Medium-quality	56.01	0	Myoviridae	prokaryote
v0f72	32749	52	27	0	Medium-quality	83.11	0	Siphoviridae	prokaryote
v0f73	34146	44	17	0	High-quality	94.59	0	Siphoviridae	prokaryote

v0f74	84156	121	34	0	High-quality	100	0	Myoviridae	prokaryote
v0f75	36082	60	15	1	High-quality	93.18	0	unclassified	unclassified
v0f76	65938	80	3	17	High-quality	95.82	0	unclassified	unclassified
v0f77	75314	117	17	1	High-quality	100	0	Quimbyviridae	prokaryote
v0f78	65387	86	13	9	Medium-quality	86.32	0	unclassified	unclassified
v0f79	35788	46	21	0	High-quality	100	0	Siphoviridae	prokaryote
v0f7a	50388	94	17	1	High-quality	100	0	unclassified	unclassified
v0f7b	82232	128	21	0	High-quality	100	0	unclassified	unclassified
v0f7c	60125	96	18	2	Medium-quality	83.24	0	unclassified	unclassified
v0f7d	33026	64	22	2	High-quality	91.13	0	Siphoviridae	prokaryote
v0f7e	34948	60	17	0	High-quality	100	0	Siphoviridae	prokaryote
v0f7f	45739	50	24	0	High-quality	97.06	0	Siphoviridae	prokaryote
v0f80	115057	163	17	5	Medium-quality	56.02	0	unclassified	unclassified
v0f81	41396	57	14	1	High-quality	100	0	unclassified	unclassified
v0f82	5745	12	6	0	High-quality	100	0	Microviridae	prokaryote
v0f83	64731	68	3	13	High-quality	100	0	unclassified	unclassified
v0f84	40942	48	16	1	High-quality	97.64	0	unclassified	unclassified
v0f85	44958	68	16	1	Medium-quality	82.89	0	Myoviridae	prokaryote
v0f86	79621	69	1	24	High-quality	100	0	unclassified	unclassified
v0f87	42453	67	48	0	High-quality	100	0	Podoviridae	prokaryote
v0f88	50438	57	5	1	Medium-quality	74.59	0	unclassified	unclassified
v0f89	39958	60	11	0	Medium-quality	63.85	0	unclassified	unclassified
v0f8a	44289	69	34	0	High-quality	99.32	0	Myoviridae	prokaryote
v0f8b	96712	155	23	1	Medium-quality	55.56	0	unclassified	unclassified
v0f8c	25032	40	19	0	Medium-quality	61.33	0	Siphoviridae	prokaryote
v0f8d	31245	34	0	9	Medium-quality	52.19	0	unclassified	unclassified
v0f8e	38181	48	26	1	High-quality	94.67	0	Siphoviridae	prokaryote
v0f8f	28025	46	15	2	Medium-quality	73.75	0	unclassified	unclassified
v0f90	39354	57	15	2	High-quality	100	0	Siphoviridae	prokaryote
v0f91	79153	86	3	28	Medium-quality	53.99	0	unclassified	unclassified
v0f92	41230	55	21	0	High-quality	100	0	Siphoviridae	prokaryote
v0f93	45194	55	0	19	Medium-quality	53.6	0	unclassified	unclassified
v0f94	53057	53	1	11	Medium-quality	59.56	0	unclassified	unclassified
v0f95	96003	98	4	12	High-quality	100	0	unclassified	unclassified
v0f96	87959	80	6	23	High-quality	100	0	unclassified	unclassified
v0f97	217294	212	12	57	High-quality	100	0	unclassified	unclassified
v0f98	33885	36	1	4	Medium-quality	56.6	0	unclassified	unclassified
v0f99	80571	79	2	17	Medium-quality	69.37	0	unclassified	unclassified
v0f9a	33691	31	1	10	Medium-quality	81.1	0	unclassified	unclassified
v0f9b	195450	220	4	64	High-quality	100	0	unclassified	unclassified

v0f9c	15229	20	1	4	High-quality	100	0	unclassified	unclassified
v0f9d	23602	24	1	1	Medium-quality	56.81	0	unclassified	unclassified
v0f9e	165207	202	13	30	High-quality	93.25	0	unclassified	unclassified
v0f9f	37514	44	1	12	Medium-quality	89.6	0	unclassified	unclassified
v0fa0	9723	17	0	1	High-quality	100	0	unclassified	unclassified
v0fa1	144631	139	16	31	High-quality	100	0	unclassified	unclassified
v0fa2	46722	51	1	12	Medium-quality	78.04	0	unclassified	unclassified
v0fa3	21377	23	0	5	Medium-quality	50.38	0	unclassified	unclassified
v0fa4	53887	52	6	18	High-quality	100	0	unclassified	unclassified
v0fa5	30614	34	4	9	Medium-quality	67.21	0	Siphoviridae	prokaryote
v0fa6	34774	38	1	10	Medium-quality	52.62	0	unclassified	unclassified
v0fa7	95685	94	1	30	High-quality	100	0	unclassified	unclassified
v0fa8	109509	131	9	30	Medium-quality	73.27	0	unclassified	unclassified
v0fa9	46272	73	25	0	Medium-quality	58.88	0	Siphoviridae	prokaryote
v0faa	119126	173	28	1	Medium-quality	89.54	0	unclassified	unclassified
v0fab	59787	96	28	1	High-quality	99.08	0	unclassified	unclassified
v0fac	45650	69	23	1	High-quality	100	0	Siphoviridae	prokaryote
v0fad	81563	127	47	2	High-quality	100	0	Siphoviridae	prokaryote
v0fae	42424	65	47	0	High-quality	100	0	Podoviridae	prokaryote
v0faf	59286	91	13	2	Medium-quality	76.13	0	Quimbyviridae	prokaryote
v0fb0	35507	40	23	2	Medium-quality	75.92	0	Siphoviridae	prokaryote
v0fb1	172174	235	44	6	High-quality	100	0	unclassified	unclassified
v0fb2	20923	36	25	0	Medium-quality	56.61	0	Siphoviridae	prokaryote
v0fb3	34325	51	45	0	Medium-quality	86.78	0	Podoviridae	prokaryote
v0fb4	40805	59	29	0	High-quality	99.28	0	Siphoviridae	prokaryote
v0fb5	16459	21	4	2	Medium-quality	57.37	0	unclassified	unclassified
v0fb6	37913	50	13	2	High-quality	100	0	Myoviridae	prokaryote
v0fb7	35319	59	8	0	Medium-quality	57.42	0	unclassified	unclassified
v0fb8	33942	45	7	5	Medium-quality	53.78	37.17	Siphoviridae	prokaryote
v0fb9	41034	57	20	0	High-quality	93.35	0	Siphoviridae	prokaryote
v0fba	41643	69	21	0	High-quality	100	0	Siphoviridae	prokaryote
v0fbb	102706	144	47	13	High-quality	100	31.94	Siphoviridae	prokaryote
v0fbc	34029	41	16	1	Medium-quality	88.12	0	Siphoviridae	prokaryote
v0fbd	29663	48	11	0	Medium-quality	79.05	0	Siphoviridae	prokaryote
v0fbe	37549	55	31	0	Medium-quality	83.86	0	Siphoviridae	prokaryote
v0fbf	35023	51	37	0	High-quality	98.74	0	Siphoviridae	prokaryote
v0fc0	41136	67	19	0	High-quality	100	0	Siphoviridae	prokaryote
v0fc1	43337	63	18	0	High-quality	100	0	Siphoviridae	prokaryote
v0fc2	94473	118	19	6	High-quality	100	0	Myoviridae	prokaryote
v0fc3	37807	51	30	1	Medium-quality	89.79	0	Siphoviridae	prokaryote

v0fc4	36768	58	18	1	High-quality	99.1	0	unclassified	unclassified
v0fc5	44386	50	21	4	High-quality	100	25.86	Siphoviridae	prokaryote
v0fc6	36479	56	28	0	Medium-quality	83.19	0	unclassified	unclassified
v0fc7	83598	85	9	9	High-quality	100	0	unclassified	unclassified
v0fc8	38339	52	23	0	High-quality	100	0	Siphoviridae	prokaryote
v0fc9	23255	38	13	0	Medium-quality	60.63	0	unclassified	unclassified
v0fca	119905	171	25	1	Medium-quality	89.78	0	unclassified	unclassified
v0fcb	48123	68	40	0	High-quality	100	0	Siphoviridae	prokaryote
v0fcc	34747	51	13	0	Medium-quality	84.77	0	Siphoviridae	prokaryote
v0fcd	37502	46	23	0	Medium-quality	83.53	0	Siphoviridae	prokaryote
v0fce	41159	47	18	1	High-quality	99.09	0	Myoviridae	prokaryote
v0fcf	19934	22	2	0	High-quality	100	0	unclassified	unclassified
v0fd0	38111	54	27	0	High-quality	95.92	0	Siphoviridae	prokaryote
v0fd1	91499	136	41	0	High-quality	100	0	Siphoviridae	prokaryote
v0fd2	34273	50	20	1	High-quality	91.03	0	Siphoviridae	prokaryote
v0fd3	36699	53	18	2	Medium-quality	88.66	0	Siphoviridae	prokaryote
v0fd4	120874	184	49	0	High-quality	100	0	unclassified	unclassified
v0fd5	45432	64	25	2	Medium-quality	68.24	0	Myoviridae	prokaryote
v0fd6	80129	126	23	4	High-quality	99.81	0	unclassified	unclassified
v0fd7	31606	48	19	1	Medium-quality	87.27	0	Siphoviridae	prokaryote
v0fd8	33023	54	22	0	High-quality	100	0	Siphoviridae	prokaryote
v0fd9	37453	43	11	1	Medium-quality	80.59	0	Siphoviridae	prokaryote
v0fda	42385	53	18	0	High-quality	100	0	Siphoviridae	prokaryote
v0fdb	20873	29	12	1	Medium-quality	57.09	0	Siphoviridae	prokaryote
v0fdc	123240	157	32	5	High-quality	100	0	unclassified	unclassified
v0fdd	39883	53	5	0	High-quality	99.49	0	unclassified	unclassified
v0fde	35935	61	22	0	Medium-quality	80.88	0	Siphoviridae	prokaryote
v0fdf	232117	255	18	10	High-quality	100	5.06	unclassified	unclassified
v0fe0	52647	89	21	0	Medium-quality	86.22	0	unclassified	unclassified
v0fe1	32110	55	24	1	High-quality	90.03	0	Siphoviridae	prokaryote
v0fe2	37317	55	26	0	High-quality	100	0	Siphoviridae	prokaryote
v0fe3	30554	42	26	0	Medium-quality	74.75	0	Siphoviridae	prokaryote
v0fe4	56210	89	12	1	High-quality	94.94	0	unclassified	unclassified
v0fe5	36370	49	16	1	High-quality	98.78	0	Siphoviridae	prokaryote
v0fe6	28616	53	19	0	Medium-quality	88.21	0	unclassified	unclassified
v0fe7	44505	38	9	1	Medium-quality	50.23	0	unclassified	unclassified
v0fe8	46160	73	15	2	High-quality	100	0	unclassified	unclassified
v0fe9	45447	73	20	0	High-quality	100	0	Myoviridae	prokaryote
v0fea	89334	119	54	5	High-quality	100	5.32	Myoviridae	prokaryote
v0feb	49877	60	12	6	High-quality	94.61	24.17	unclassified	unclassified

v0fec	37001	48	20	0	High-quality	96.22	0	Siphoviridae	prokaryote
v0fed	10736	15	7	1	Medium-quality	79.28	0	Siphoviridae	prokaryote
v0fee	27829	37	6	2	High-quality	100	0	unclassified	unclassified
v0fef	45621	61	30	1	High-quality	100	0	Siphoviridae	prokaryote
v0ff0	39070	53	32	0	High-quality	94.23	0	Siphoviridae	prokaryote
v0ff1	36742	56	16	1	High-quality	92.67	0	Siphoviridae	prokaryote
v0ff2	38581	53	31	0	High-quality	93.5	0	Siphoviridae	prokaryote
v0ff3	30752	47	15	0	Medium-quality	83.71	0	Siphoviridae	prokaryote
v0ff4	38210	43	1	12	Medium-quality	83.3	0	unclassified	unclassified
v0ff5	37874	49	1	11	Medium-quality	70.64	0	Myoviridae	prokaryote
v0ff6	38761	36	1	10	Medium-quality	58.65	0	unclassified	unclassified
v0ff7	104444	107	1	21	Medium-quality	53.92	0	unclassified	unclassified
v0ff8	41968	47	1	13	Medium-quality	63.5	0	Myoviridae	prokaryote
v0ff9	73872	73	5	12	High-quality	100	0	unclassified	unclassified
v0ffa	32565	42	2	3	Medium-quality	78.04	0	unclassified	unclassified
v0ffb	87069	77	1	23	High-quality	100	0	unclassified	unclassified
v0ffc	23645	26	1	6	Medium-quality	51.54	0	unclassified	unclassified
v0ffd	54125	64	1	18	Medium-quality	60.76	0	unclassified	unclassified
v0ffe	81060	88	8	16	Medium-quality	69.12	0	Quimbyviridae	prokaryote
v0fff	67930	64	1	20	High-quality	100	0	unclassified	unclassified
v1000	51475	37	3	3	Medium-quality	81.71	0	unclassified	unclassified
v1001	57570	63	3	20	Medium-quality	83.95	0	unclassified	unclassified
v1002	41010	36	0	11	Low-quality	35.91	0	unclassified	unclassified
v1003	78263	78	2	22	High-quality	100	0	unclassified	unclassified
v1004	69366	65	1	18	High-quality	100	0	unclassified	unclassified
v1005	71640	71	1	18	High-quality	100	0	unclassified	unclassified
v1006	276895	295	20	89	High-quality	100	0	unclassified	unclassified
v1007	97225	118	8	14	High-quality	100	0	unclassified	unclassified
v1008	9091	15	0	0	High-quality	100	0	Inoviridae	prokaryote
v1009	85348	85	2	27	High-quality	100	0	unclassified	unclassified
v100a	77541	71	7	26	High-quality	100	0	unclassified	unclassified
v100b	27546	34	1	6	Medium-quality	58.84	0	unclassified	unclassified
v100c	28350	35	1	7	Medium-quality	58.12	0	unclassified	unclassified
v100d	66095	63	1	19	Medium-quality	78.7	0	unclassified	unclassified
v100e	40177	52	17	0	High-quality	97.47	0	unclassified	unclassified
v100f	50047	60	25	1	High-quality	100	0	Siphoviridae	prokaryote
v1010	40591	64	28	0	Medium-quality	86.56	0	Myoviridae	prokaryote
v1011	38678	37	6	1	Medium-quality	64.73	0	unclassified	unclassified
v1012	21610	44	5	0	Medium-quality	51.46	0	unclassified	unclassified
v1013	73666	87	14	5	High-quality	100	0	Myoviridae	prokaryote

v1014	38036	62	22	0	High-quality	95.34	0	Siphoviridae	prokaryote
v1015	35654	55	16	0	Medium-quality	88.86	0	Siphoviridae	prokaryote
v1016	26742	36	16	0	Medium-quality	83.81	0	unclassified	unclassified
v1017	36193	54	43	0	High-quality	91.26	0	Podoviridae	prokaryote
v1018	37408	57	37	0	High-quality	93.29	0	Siphoviridae	prokaryote
v1019	47651	62	24	0	Medium-quality	58.84	0	Siphoviridae	prokaryote
v101a	106035	138	21	5	Medium-quality	59.02	0	unclassified	unclassified
v101b	34892	31	8	0	Medium-quality	52.93	0	unclassified	unclassified
v101c	165008	209	40	7	High-quality	94.73	0	unclassified	unclassified
v101d	39463	57	17	2	High-quality	100	0	Siphoviridae	prokaryote
v101e	89009	122	15	3	High-quality	100	0	Quimbyviridae	prokaryote
v101f	37424	58	45	0	Medium-quality	76.62	0	Myoviridae	prokaryote
v1020	116219	145	12	15	High-quality	100	0	unclassified	unclassified
v1021	62937	72	12	4	High-quality	96.65	0	unclassified	unclassified
v1022	35603	46	28	0	High-quality	100	0	Myoviridae	prokaryote
v1023	88220	99	13	9	High-quality	100	0	unclassified	unclassified
v1024	58684	66	36	0	High-quality	100	0	Siphoviridae	prokaryote
v1025	40337	62	24	0	High-quality	100	0	Siphoviridae	prokaryote
v1026	75793	104	13	6	High-quality	100	0	unclassified	unclassified
v1027	29937	40	5	0	Medium-quality	72.36	0	unclassified	unclassified
v1028	41324	45	20	0	High-quality	98.92	0	Siphoviridae	prokaryote
v1029	80316	102	13	2	High-quality	100	0	unclassified	unclassified
v102a	30376	52	11	0	Medium-quality	75.33	0	unclassified	unclassified
v102b	14809	21	3	0	Medium-quality	51.62	0	unclassified	unclassified
v102c	106996	118	8	19	Medium-quality	72.93	67.01	unclassified	unclassified
v102d	54178	59	5	2	Medium-quality	85.95	0	unclassified	unclassified
v102e	20560	24	8	1	Medium-quality	51.77	0	Podoviridae	prokaryote
v102f	44313	44	12	4	Medium-quality	53.03	21.7	unclassified	unclassified
v1030	9602	10	3	0	High-quality	100	0	Microviridae	prokaryote
v1031	69303	101	30	0	High-quality	100	0	Myoviridae	prokaryote
v1032	40132	64	18	0	High-quality	100	0	Siphoviridae	prokaryote
v1033	10406	13	3	0	High-quality	100	0	unclassified	unclassified
v1034	39955	56	25	1	High-quality	99.95	0	Siphoviridae	prokaryote
v1035	53499	50	22	4	Medium-quality	83.72	19	Siphoviridae	prokaryote
v1036	60965	103	26	1	High-quality	97.66	0	unclassified	unclassified
v1037	14228	16	7	4	High-quality	100	42.75	Microviridae	prokaryote
v1038	54667	61	23	2	High-quality	100	0	Siphoviridae	prokaryote
v1039	47429	88	18	0	High-quality	91.49	0	unclassified	unclassified
v103a	42308	74	19	0	High-quality	94.85	0	unclassified	unclassified
v103b	35736	48	19	2	Medium-quality	76.5	0	Siphoviridae	prokaryote

v103c	55298	78	16	2	High-quality	92.77	0	Quimbyviridae	prokaryote
v103d	39403	61	46	0	Medium-quality	71.89	0	Myoviridae	prokaryote
v103e	32908	42	10	0	High-quality	98.85	0	unclassified	unclassified
v103f	75513	99	31	3	High-quality	100	0	Siphoviridae	prokaryote
v1040	39711	48	35	0	Medium-quality	82.91	0	Siphoviridae	prokaryote
v1041	71959	94	16	5	High-quality	100	0	unclassified	unclassified
v1042	54542	52	5	2	High-quality	100	0	unclassified	unclassified
v1043	37249	49	8	2	Low-quality	47.69	0	unclassified	unclassified
v1044	38566	53	29	0	Medium-quality	55.61	0	Siphoviridae	prokaryote
v1045	38341	43	4	2	Medium-quality	52.17	0	unclassified	unclassified
v1046	32060	50	16	0	Medium-quality	88.56	0	unclassified	unclassified
v1047	102281	150	50	1	High-quality	100	0	Siphoviridae	prokaryote
v1048	69271	106	16	5	High-quality	100	0	unclassified	unclassified
v1049	38040	58	25	0	Medium-quality	81.08	0	Myoviridae	prokaryote
v104a	50039	64	9	2	High-quality	100	0	unclassified	unclassified
v104b	64675	105	21	1	High-quality	100	0	unclassified	unclassified
v104c	22959	51	7	1	Medium-quality	50.39	0	unclassified	unclassified
v104d	46667	51	30	11	Medium-quality	89.65	37.56	Myoviridae	prokaryote
v104e	48635	65	29	1	High-quality	100	0	Myoviridae	prokaryote
v104f	40857	65	44	0	High-quality	96.65	0	Myoviridae	prokaryote
v1050	41904	55	17	1	High-quality	100	0	unclassified	unclassified
v1051	55035	70	22	2	High-quality	90.99	0	Siphoviridae	prokaryote
v1052	145752	129	4	48	Complete	100	0	unclassified	unclassified
v1053	53195	59	3	6	High-quality	100	0	unclassified	unclassified
v1054	54556	59	4	10	Medium-quality	60.68	0	unclassified	unclassified
v1055	71371	64	1	15	High-quality	100	0	unclassified	unclassified
v1056	27755	30	1	5	Medium-quality	59.7	0	unclassified	unclassified
v1057	43197	46	1	11	Medium-quality	65.36	0	unclassified	unclassified
v1058	28421	42	0	8	Medium-quality	65.11	0	Siphoviridae	prokaryote
v1059	23804	14	1	4	Medium-quality	52.62	0	unclassified	unclassified
v105a	50030	51	0	11	Medium-quality	65.26	0	unclassified	unclassified
v105b	27615	26	0	9	Medium-quality	56.03	0	unclassified	unclassified
v105c	21504	23	1	2	Medium-quality	50	0	unclassified	unclassified
v105d	100260	101	1	30	High-quality	100	0	unclassified	unclassified
v105e	20075	21	1	3	High-quality	100	0	unclassified	unclassified
v105f	75018	77	2	22	Medium-quality	89.32	0	unclassified	unclassified
v1060	44415	49	1	10	Medium-quality	67.21	0	unclassified	unclassified
v1061	35200	30	0	5	Medium-quality	75.72	0	unclassified	unclassified
v1062	121060	100	4	25	High-quality	100	0	unclassified	unclassified
v1063	36496	38	1	9	Medium-quality	55.84	0	unclassified	unclassified

v1064	31140	39	2	4	Medium-quality	68.8	0	unclassified	unclassified
v1065	23394	24	2	8	Medium-quality	57.33	0	unclassified	unclassified
v1066	37890	47	25	0	High-quality	93.87	0	Siphoviridae	prokaryote
v1067	39679	52	27	0	High-quality	97.41	0	Siphoviridae	prokaryote
v1068	83522	101	26	7	High-quality	100	0	Siphoviridae	prokaryote
v1069	53784	56	21	3	High-quality	91.04	0	Siphoviridae	prokaryote
v106a	33947	49	5	0	Medium-quality	87.14	0	unclassified	unclassified
v106b	40459	53	12	0	High-quality	100	0	unclassified	unclassified
v106c	15692	23	2	0	Medium-quality	82.34	0	unclassified	unclassified
v106d	30451	51	17	0	Medium-quality	72.39	0	Siphoviridae	prokaryote
v106e	38823	53	23	2	High-quality	93.98	0	Siphoviridae	prokaryote
v106f	37523	57	21	0	High-quality	95.71	0	Siphoviridae	prokaryote
v1070	59643	52	7	0	High-quality	94.75	0	unclassified	unclassified
v1071	55029	81	30	0	High-quality	100	0	unclassified	unclassified
v1072	55458	91	20	2	High-quality	100	0	unclassified	unclassified
v1073	33826	62	20	3	High-quality	92.8	0	Siphoviridae	prokaryote
v1074	43680	61	16	3	High-quality	100	0	Siphoviridae	prokaryote
v1075	86996	113	27	7	High-quality	100	0	Myoviridae	prokaryote
v1076	33626	58	20	0	Medium-quality	84.53	0	Siphoviridae	prokaryote
v1077	34132	32	2	1	Medium-quality	75.45	0	unclassified	unclassified
v1078	36781	51	21	0	Medium-quality	81.41	0	Siphoviridae	prokaryote
v1079	37768	52	28	0	High-quality	90.27	0	Siphoviridae	prokaryote
v107a	76354	96	14	5	High-quality	100	0	unclassified	unclassified
v107b	62261	90	13	1	High-quality	100	0	unclassified	unclassified
v107c	34835	56	15	1	High-quality	97.71	0	unclassified	unclassified
v107d	37360	46	9	2	High-quality	100	0	Siphoviridae	prokaryote
v107e	42338	62	30	0	High-quality	99.45	0	Myoviridae	prokaryote
v107f	68626	89	15	1	High-quality	100	0	Quimbyviridae	prokaryote
v1080	39843	52	19	2	Medium-quality	85.27	0	Siphoviridae	prokaryote
v1081	19732	20	3	2	High-quality	100	23.87	unclassified	unclassified
v1082	40369	64	38	0	High-quality	100	0	Siphoviridae	prokaryote
v1083	85113	129	40	2	High-quality	100	0	unclassified	unclassified
v1084	46121	51	16	3	Medium-quality	77.81	25.69	unclassified	unclassified
v1085	36119	50	3	7	Medium-quality	73.26	0	unclassified	unclassified
v1086	19159	24	1	3	High-quality	100	0	unclassified	unclassified
v1087	40896	38	1	11	Medium-quality	54.45	0	unclassified	unclassified
v1088	159412	169	17	48	High-quality	99.8	0	unclassified	unclassified
v1089	33604	37	1	9	Medium-quality	78.01	0	unclassified	unclassified
v108a	188374	172	1	56	High-quality	100	0	unclassified	unclassified
v108b	25429	34	1	10	Medium-quality	56.06	0	unclassified	unclassified

v108c	243066	286	20	71	High-quality	100	0	unclassified	unclassified
v108d	98813	112	1	17	Complete	100	0	unclassified	unclassified
v108e	88028	93	4	27	High-quality	92.58	0	unclassified	unclassified
v108f	29977	37	1	12	Medium-quality	69.81	0	unclassified	unclassified
v1090	94319	92	8	40	Medium-quality	54.03	0	unclassified	unclassified
v1091	28175	29	23	2	Medium-quality	68.38	0	Siphoviridae	prokaryote
v1092	37990	56	47	0	High-quality	94.93	0	Podoviridae	prokaryote
v1093	44338	60	45	0	High-quality	94.55	0	Siphoviridae	prokaryote
v1094	167400	265	36	4	High-quality	94.91	0	unclassified	unclassified
v1095	41408	55	16	0	Medium-quality	50.29	0	Siphoviridae	prokaryote
v1096	45815	62	16	0	High-quality	100	0	unclassified	unclassified
v1097	41211	59	34	0	Medium-quality	87.88	0	Myoviridae	prokaryote
v1098	37794	46	29	1	Medium-quality	89.58	0	Myoviridae	prokaryote
v1099	44542	67	14	0	High-quality	100	0	unclassified	unclassified
v109a	59213	99	15	2	High-quality	100	0	unclassified	unclassified
v109b	49191	81	27	2	High-quality	97.69	0	Myoviridae	prokaryote
v109c	48122	51	9	4	High-quality	100	11.43	unclassified	unclassified
v109d	41200	51	11	2	High-quality	100	0	unclassified	unclassified
v109e	77271	71	14	6	High-quality	93.85	0	unclassified	unclassified
v109f	40448	63	31	1	High-quality	100	0	Siphoviridae	prokaryote
v10a0	39703	58	19	1	High-quality	100	0	Siphoviridae	prokaryote
v10a1	32622	18	3	0	Medium-quality	51.89	0	unclassified	unclassified
v10a2	22445	24	5	3	Medium-quality	78.25	0	unclassified	unclassified
v10a3	54680	51	6	4	High-quality	100	0	unclassified	unclassified
v10a4	33288	52	32	0	High-quality	95.05	0	Myoviridae	prokaryote
v10a5	30591	44	9	1	Medium-quality	61.49	0	unclassified	unclassified
v10a6	63577	76	8	2	High-quality	99.55	0	unclassified	unclassified
v10a7	91925	103	43	15	High-quality	100	46.12	Myoviridae	prokaryote
v10a8	200627	315	39	5	High-quality	100	0	unclassified	unclassified
v10a9	58952	76	11	3	High-quality	98.66	0	unclassified	unclassified
v10aa	33611	59	13	0	Medium-quality	73.49	0	unclassified	unclassified
v10ab	48434	57	18	2	High-quality	100	0	Siphoviridae	prokaryote
v10ac	31488	45	27	0	Medium-quality	82.17	0	Siphoviridae	prokaryote
v10ad	42094	46	15	3	Medium-quality	52.48	15.14	unclassified	unclassified
v10ae	42254	41	1	12	Medium-quality	56.85	0	unclassified	unclassified
v10af	119340	94	1	31	High-quality	100	0	unclassified	unclassified
v10b0	63298	64	3	12	High-quality	100	0	unclassified	unclassified
v10b1	44724	59	23	3	High-quality	94.54	0	Siphoviridae	prokaryote
v10b2	32017	38	12	0	Medium-quality	82.88	0	unclassified	unclassified
v10b3	107867	142	24	3	High-quality	100	0	unclassified	unclassified

v10b4	38937	63	23	0	High-quality	100	0	Myoviridae	prokaryote
v10b5	138181	224	34	6	Medium-quality	78.25	0	unclassified	unclassified
v10b6	58572	107	22	1	High-quality	99.34	0	unclassified	unclassified
v10b7	35140	50	17	0	High-quality	90.32	0	Siphoviridae	prokaryote
v10b8	36105	50	13	5	Medium-quality	72.96	14.31	unclassified	unclassified
v10b9	62653	64	8	1	High-quality	100	0	unclassified	unclassified
v10ba	78797	81	9	7	Medium-quality	88.35	0	unclassified	unclassified
v10bb	37082	50	17	2	High-quality	92.29	0	unclassified	unclassified
v10bc	35588	55	21	1	High-quality	92.09	0	Siphoviridae	prokaryote
v10bd	47188	67	9	0	High-quality	100	0	unclassified	unclassified
v10be	41257	61	24	2	High-quality	100	0	Siphoviridae	prokaryote
v10bf	121472	159	38	2	Medium-quality	73.77	0	unclassified	unclassified
v10c0	49729	68	19	0	High-quality	100	0	unclassified	unclassified
v10c1	74611	104	31	3	High-quality	100	0	Siphoviridae	prokaryote
v10c2	44983	48	16	2	Medium-quality	54.64	0	Siphoviridae	prokaryote
v10c3	79679	100	15	2	High-quality	100	0	unclassified	unclassified
v10c4	91060	132	14	3	High-quality	100	0	Quimbyviridae	prokaryote
v10c5	47094	47	3	1	High-quality	100	0	unclassified	unclassified
v10c6	51401	48	6	2	High-quality	100	0	unclassified	unclassified
v10c7	25260	33	6	2	High-quality	100	0	unclassified	unclassified
v10c8	70281	96	14	2	Medium-quality	78.52	0	unclassified	unclassified
v10c9	59023	56	6	0	High-quality	93.88	0	unclassified	unclassified
v10ca	69164	59	8	2	Medium-quality	80.93	0	unclassified	unclassified
v10cb	55601	64	26	2	Medium-quality	80.03	0	Siphoviridae	prokaryote
v10cc	62702	93	9	0	High-quality	100	0	unclassified	unclassified
v10cd	86735	134	12	4	High-quality	98.66	0	Quimbyviridae	prokaryote
v10ce	27476	40	17	1	Medium-quality	84.71	0	Siphoviridae	prokaryote
v10cf	46403	48	6	8	Medium-quality	78.98	0	unclassified	unclassified
v10d0	35407	34	0	11	Medium-quality	52.63	0	unclassified	unclassified
v10d1	61473	72	3	15	Medium-quality	83.28	0	unclassified	unclassified
v10d2	60939	65	3	10	High-quality	100	0	unclassified	unclassified
v10d3	85176	88	1	29	High-quality	100	0	unclassified	unclassified
v10d4	110340	96	1	33	High-quality	100	0	unclassified	unclassified
v10d5	56101	57	1	9	High-quality	100	0	unclassified	unclassified
v10d6	24290	33	14	0	Medium-quality	50.8	0	Myoviridae	prokaryote
v10d7	40566	60	23	0	High-quality	100	0	Siphoviridae	prokaryote
v10d8	41219	61	16	2	High-quality	100	0	Myoviridae	prokaryote
v10d9	97619	131	22	6	High-quality	100	0	unclassified	unclassified
v10da	28835	30	4	0	Medium-quality	75.82	0	unclassified	unclassified
v10db	125999	180	41	13	High-quality	100	0	Myoviridae	prokaryote

v10dc	41830	44	17	1	High-quality	100	0	Siphoviridae	prokaryote
v10dd	77282	53	2	18	High-quality	94.34	0	unclassified	unclassified
v10de	100982	111	3	33	High-quality	97.7	0	unclassified	unclassified
v10df	70592	64	7	25	High-quality	100	0	unclassified	unclassified
v10e0	23109	26	22	0	Medium-quality	58.66	0	Siphoviridae	prokaryote
v10e1	45086	45	10	3	Medium-quality	70.05	0	unclassified	unclassified
v10e2	38355	63	10	2	Medium-quality	63.41	0	Siphoviridae	prokaryote
v10e3	32830	39	32	1	Medium-quality	67.3	0	Siphoviridae	prokaryote
v10e4	29983	49	28	0	Medium-quality	78.52	0	Siphoviridae	prokaryote
v10e5	55408	74	14	2	High-quality	100	0	unclassified	unclassified
v10e6	63781	79	18	8	High-quality	92.26	0	Siphoviridae	prokaryote
v10e7	40040	50	14	5	High-quality	93.61	13.81	Siphoviridae	prokaryote
v10e8	42705	65	22	1	High-quality	90.7	0	Myoviridae	prokaryote
v10e9	17050	20	5	0	Medium-quality	59.43	0	Siphoviridae	prokaryote
v10ea	36437	58	30	0	Medium-quality	85.49	0	Myoviridae	prokaryote
v10eb	42895	53	12	4	Medium-quality	73.05	0	unclassified	unclassified
v10ec	42294	38	0	9	Medium-quality	60.07	0	unclassified	unclassified
v10ed	30945	43	1	1	Medium-quality	59.23	0	unclassified	unclassified
v10ee	227481	243	13	57	High-quality	100	0	unclassified	unclassified
v10ef	5043	6	0	0	Medium-quality	81.33	0	unclassified	unclassified
v10f0	29572	29	1	4	High-quality	100	0	unclassified	unclassified
v10f1	5569	8	0	0	Medium-quality	89.81	0	unclassified	unclassified
v10f2	6457	8	0	0	High-quality	100	0	unclassified	unclassified
v10f3	36114	35	19	0	Medium-quality	86.47	0	Siphoviridae	prokaryote
v10f4	64537	77	14	1	High-quality	95.93	0	unclassified	unclassified
v10f5	40125	45	7	3	High-quality	99.47	0	unclassified	unclassified
v10f6	32926	38	18	0	Medium-quality	79.75	0	Siphoviridae	prokaryote
v10f7	67861	78	11	4	Medium-quality	83.88	0	unclassified	unclassified
v10f8	58404	84	20	2	High-quality	95.29	0	unclassified	unclassified
v10f9	33655	54	43	0	Medium-quality	85.3	0	Podoviridae	prokaryote
v10fa	29848	27	7	1	Medium-quality	78.64	0	Siphoviridae	prokaryote
v10fb	44981	66	19	1	High-quality	100	0	Siphoviridae	prokaryote
v10fc	9824	7	1	0	Medium-quality	51.83	0	Retroviridae	eukaryote
v10fd	50296	49	1	13	High-quality	100	0	unclassified	unclassified
v10fe	183221	173	9	37	High-quality	100	0	unclassified	unclassified
v10ff	36054	37	1	9	Medium-quality	56.92	0	unclassified	unclassified
v1100	58929	68	1	19	High-quality	98.43	0	unclassified	unclassified
v1101	64478	71	1	23	Medium-quality	66.27	0	unclassified	unclassified
v1102	77084	71	4	14	High-quality	100	0	unclassified	unclassified
v1103	24975	31	4	2	Medium-quality	87.05	0	unclassified	unclassified

v1104	30618	38	14	0	Medium-quality	78.87	0	Siphoviridae	prokaryote
v1105	49409	72	11	2	High-quality	100	0	unclassified	unclassified
v1106	34799	43	6	2	Medium-quality	76.99	0	unclassified	unclassified
v1107	41397	47	32	1	High-quality	99.39	0	Siphoviridae	prokaryote
v1108	32141	55	21	0	High-quality	91.11	0	Myoviridae	prokaryote
v1109	34413	65	20	1	High-quality	94.96	0	Siphoviridae	prokaryote
v110a	29313	38	17	0	Medium-quality	73.53	0	Siphoviridae	prokaryote
v110b	42303	75	26	0	High-quality	100	0	Siphoviridae	prokaryote
v110c	42622	64	9	1	High-quality	94.92	0	unclassified	unclassified
v110d	39102	45	18	0	High-quality	94.18	0	Myoviridae	prokaryote
v110e	42208	58	18	0	High-quality	100	0	unclassified	unclassified
v110f	28280	50	13	0	Medium-quality	66.68	0	unclassified	unclassified
v1110	39700	46	18	1	High-quality	96.7	0	Siphoviridae	prokaryote
v1111	41665	57	20	0	High-quality	100	0	unclassified	unclassified
v1112	61077	84	21	7	High-quality	100	0	unclassified	unclassified
v1113	37740	46	30	0	High-quality	92.76	0	Myoviridae	prokaryote
v1114	18393	10	0	3	Medium-quality	53.04	0	unclassified	unclassified
v1115	10762	12	1	4	High-quality	100	0	Siphoviridae	prokaryote
v1116	34550	47	3	8	Medium-quality	84.65	0	unclassified	unclassified
v1117	67025	66	3	13	Medium-quality	77.01	0	unclassified	unclassified
v1118	20691	24	19	0	Medium-quality	52.33	0	Podoviridae	prokaryote
v1119	36384	54	21	0	High-quality	100	0	unclassified	unclassified
v111a	39921	53	16	0	High-quality	100	0	unclassified	unclassified
v111b	64271	82	15	4	High-quality	100	0	unclassified	unclassified
v111c	37183	50	6	0	Medium-quality	89.81	0	unclassified	unclassified
v111d	28795	49	15	0	Medium-quality	73.92	0	Siphoviridae	prokaryote
v111e	40721	64	13	1	High-quality	100	0	unclassified	unclassified
v111f	44620	61	15	0	High-quality	100	0	unclassified	unclassified
v1120	64461	86	16	10	Medium-quality	82.61	33.49	unclassified	unclassified
v1121	27362	50	17	0	Medium-quality	72.92	0	unclassified	unclassified
v1122	90538	81	2	28	High-quality	100	0	unclassified	unclassified
v1123	124849	106	4	36	High-quality	100	0	unclassified	unclassified
v1124	97997	102	5	8	High-quality	100	0	Podoviridae_crAss-like	prokaryote
v1125	32599	37	3	10	Medium-quality	71.44	0	unclassified	unclassified
v1126	102312	118	7	19	High-quality	97.1	0	unclassified	unclassified
v1127	79917	87	7	22	High-quality	100	0	unclassified	unclassified
v1128	53475	59	1	17	Medium-quality	88.76	0	unclassified	unclassified
v1129	187790	194	17	74	High-quality	100	0	unclassified	unclassified
v112a	7566	7	0	1	Medium-quality	63.72	0	unclassified	unclassified
v112b	219513	201	15	61	High-quality	99.47	0	unclassified	unclassified

v112c	99052	104	7	9	High-quality	100	0	unclassified	unclassified
v112d	118125	106	8	36	High-quality	100	0	unclassified	unclassified
v112e	218077	226	18	22	Medium-quality	88.75	0	unclassified	unclassified
v112f	70173	82	3	14	High-quality	100	0	unclassified	unclassified
v1130	78840	112	38	2	High-quality	100	0	Siphoviridae	prokaryote
v1131	54066	63	19	4	Medium-quality	84.16	0	unclassified	unclassified
v1132	35901	55	15	0	High-quality	100	0	unclassified	unclassified
v1133	63117	73	10	2	High-quality	93.84	0	unclassified	unclassified
v1134	34035	44	22	0	Medium-quality	89.17	0	Siphoviridae	prokaryote
v1135	80688	120	9	17	High-quality	100	0	unclassified	unclassified
v1136	43931	59	45	0	High-quality	100	0	Siphoviridae	prokaryote
v1137	67382	96	22	2	High-quality	100	0	Myoviridae	prokaryote
v1138	89700	132	9	7	High-quality	90.41	0	unclassified	unclassified
v1139	37803	54	22	3	High-quality	94.39	0	Siphoviridae	prokaryote
v113a	18480	25	4	1	Medium-quality	64.41	0	Siphoviridae	prokaryote
v113b	39582	81	8	3	Medium-quality	50.2	0	unclassified	unclassified
v113c	62453	92	34	1	High-quality	100	0	Siphoviridae	prokaryote
v113d	63874	67	10	1	High-quality	100	0	unclassified	unclassified
v113e	39712	44	20	0	Medium-quality	82.93	0	Siphoviridae	prokaryote
v113f	38626	64	13	0	Medium-quality	77.36	0	unclassified	unclassified
v1140	36164	51	17	0	High-quality	93.74	0	unclassified	unclassified
v1141	42031	66	12	1	Medium-quality	52.56	0	unclassified	unclassified
v1142	43310	67	9	0	High-quality	100	0	unclassified	unclassified
v1143	52260	79	47	0	High-quality	100	0	Siphoviridae	prokaryote
v1144	158621	219	46	1	High-quality	92.04	0	unclassified	unclassified
v1145	36878	56	18	0	Medium-quality	86.28	0	unclassified	unclassified
v1146	62293	58	8	7	Medium-quality	72.15	30.4	unclassified	unclassified
v1147	241122	224	14	57	High-quality	100	0	unclassified	unclassified
v1148	42394	50	7	2	High-quality	94.36	0	unclassified	unclassified
v1149	44213	76	22	0	High-quality	99.13	0	unclassified	unclassified
v114a	43266	49	21	1	High-quality	95.85	0	Siphoviridae	prokaryote
v114b	41093	73	18	0	High-quality	97.04	0	unclassified	unclassified
v114c	142845	251	23	5	High-quality	100	0	unclassified	unclassified
v114d	87006	124	19	5	High-quality	100	0	unclassified	unclassified
v114e	41345	66	21	0	High-quality	100	0	Siphoviridae	prokaryote
v114f	106495	121	11	19	Medium-quality	67.6	65.78	unclassified	unclassified
v1150	56715	69	31	1	High-quality	100	0	Myoviridae	prokaryote
v1151	22950	20	3	2	High-quality	100	0	unclassified	unclassified
v1152	27430	41	18	1	Medium-quality	85.41	0	Siphoviridae	prokaryote
v1153	46199	59	32	3	High-quality	100	0	Siphoviridae	prokaryote

v1154	48066	50	5	2	High-quality	99.3	0	unclassified	unclassified
v1155	46103	67	14	1	High-quality	100	0	unclassified	unclassified
v1156	72613	110	32	3	High-quality	100	0	Siphoviridae	prokaryote
v1157	155797	217	39	6	Medium-quality	83.68	0	unclassified	unclassified
v1158	74639	84	23	0	High-quality	90.66	0	Siphoviridae	prokaryote
v1159	119332	165	39	12	High-quality	100	0	Myoviridae	prokaryote
v115a	58591	91	36	1	High-quality	95.62	0	Siphoviridae	prokaryote
v115b	73973	95	24	4	High-quality	100	0	Myoviridae	prokaryote
v115c	46432	56	0	14	Complete	100	0	unclassified	unclassified
v115d	156854	161	2	45	Medium-quality	81.58	0	unclassified	unclassified
v115e	115440	110	3	38	High-quality	100	0	unclassified	unclassified
v115f	65164	80	1	24	High-quality	100	0	unclassified	unclassified
v1160	8260	15	0	0	Complete	100	0	unclassified	unclassified
v1161	209795	255	12	68	High-quality	100	0	unclassified	unclassified
v1162	167563	206	4	72	High-quality	100	0	unclassified	unclassified
v1163	44009	46	1	11	High-quality	100	0	unclassified	unclassified
v1164	116540	113	1	36	Medium-quality	83.43	0	unclassified	unclassified
v1165	16780	20	5	5	Medium-quality	58.49	0	Siphoviridae	prokaryote
v1166	171240	241	56	4	High-quality	95.39	0	unclassified	unclassified
v1167	48520	79	13	0	Medium-quality	76.28	0	Quimbyviridae	prokaryote
v1168	34821	49	13	0	High-quality	100	0	unclassified	unclassified
v1169	41070	49	19	1	High-quality	99.84	0	Siphoviridae	prokaryote
v116a	91395	124	46	14	High-quality	100	0	Siphoviridae	prokaryote
v116b	41369	73	26	0	High-quality	100	0	unclassified	unclassified
v116c	34455	39	16	2	Medium-quality	64.46	0	Siphoviridae	prokaryote
v116d	42160	71	18	0	High-quality	100	0	Siphoviridae	prokaryote
v116e	48740	51	14	3	Medium-quality	89.54	0	Siphoviridae	prokaryote
v116f	34169	48	17	0	High-quality	100	0	Siphoviridae	prokaryote
v1170	199729	319	44	7	High-quality	97.13	0	unclassified	unclassified
v1171	36626	52	18	1	High-quality	94.06	0	Siphoviridae	prokaryote
v1172	59416	84	19	0	High-quality	100	0	Myoviridae	prokaryote
v1173	42130	67	22	0	High-quality	100	0	Siphoviridae	prokaryote
v1174	61955	89	13	3	High-quality	97.56	0	unclassified	unclassified
v1175	34894	50	17	0	High-quality	99.53	0	Siphoviridae	prokaryote
v1176	35153	51	22	4	Medium-quality	79.97	21.22	Siphoviridae	prokaryote
v1177	71502	77	11	5	High-quality	100	0	unclassified	unclassified
v1178	16102	24	6	0	High-quality	93.65	0	unclassified	unclassified
v1179	22230	37	17	1	Medium-quality	64.37	0	Siphoviridae	prokaryote
v117a	19092	29	2	0	High-quality	100	0	unclassified	unclassified
v117b	39814	63	21	0	High-quality	99.33	0	Siphoviridae	prokaryote

v117c	127633	196	54	10	High-quality	100	0	Siphoviridae	prokaryote
v117d	63557	91	32	0	High-quality	100	0	Siphoviridae	prokaryote
v117e	54124	99	19	1	High-quality	95.2	0	unclassified	unclassified
v117f	123869	184	40	20	Medium-quality	88.01	27.25	unclassified	unclassified
v1180	32999	48	26	0	High-quality	90.18	0	Siphoviridae	prokaryote
v1181	25796	39	11	2	Medium-quality	68.22	0	unclassified	unclassified
v1182	46692	68	19	3	High-quality	100	0	Siphoviridae	prokaryote
v1183	31957	41	26	1	Medium-quality	69.72	0	Siphoviridae	prokaryote
v1184	38996	52	31	1	High-quality	99.01	0	Siphoviridae	prokaryote
v1185	47049	59	31	0	High-quality	100	0	Myoviridae	prokaryote
v1186	5076	5	1	0	High-quality	100	0	unclassified	unclassified
v1187	39112	57	14	1	High-quality	100	0	unclassified	unclassified
v1188	54090	81	19	2	High-quality	100	0	Myoviridae	prokaryote
v1189	28363	51	23	2	Medium-quality	78.26	0	Siphoviridae	prokaryote
v118a	75077	85	29	10	High-quality	100	46.18	unclassified	unclassified
v118b	36222	50	16	3	Medium-quality	81.79	18.39	unclassified	unclassified
v118c	41998	53	7	2	High-quality	100	0	unclassified	unclassified
v118d	51007	72	21	3	High-quality	100	0	Siphoviridae	prokaryote
v118e	31480	37	1	12	Medium-quality	52.23	0	unclassified	unclassified
v118f	27056	30	1	9	Medium-quality	81.01	0	unclassified	unclassified
v1190	41480	58	13	1	Medium-quality	85.7	0	unclassified	unclassified
v1191	39327	60	32	0	High-quality	100	0	Siphoviridae	prokaryote
v1192	113273	139	14	3	High-quality	100	0	unclassified	unclassified
v1193	26012	39	24	0	Medium-quality	75.4	0	Siphoviridae	prokaryote
v1194	24255	26	19	0	Medium-quality	62.19	0	Podoviridae	prokaryote
v1195	83532	124	14	2	High-quality	95.19	0	Quimbyviridae	prokaryote
v1196	88605	104	2	33	Medium-quality	77.11	0	unclassified	unclassified
v1197	19935	35	1	8	Medium-quality	50.71	0	unclassified	unclassified
v1198	70684	62	0	20	High-quality	100	0	unclassified	unclassified
v1199	60725	62	1	19	High-quality	100	0	unclassified	unclassified
v119a	54438	48	1	16	High-quality	100	0	unclassified	unclassified
v119b	46581	61	6	12	Medium-quality	77.96	0	unclassified	unclassified
v119c	139742	144	7	74	Complete	100	69.74	unclassified	unclassified
v119d	26153	47	14	0	Medium-quality	72.52	0	unclassified	unclassified
v119e	163143	227	29	10	High-quality	99.64	0	unclassified	unclassified
v119f	49780	65	29	0	High-quality	100	0	Myoviridae	prokaryote
v11a0	31190	47	18	1	Medium-quality	80.05	0	unclassified	unclassified
v11a1	97762	143	39	13	High-quality	100	34.93	unclassified	unclassified
v11a2	48328	87	17	2	High-quality	100	0	unclassified	unclassified
v11a3	10998	10	3	1	High-quality	100	28.09	Microviridae	prokaryote

v11a4	38021	50	12	1	High-quality	94.32	0	unclassified	unclassified
v11a5	45150	63	17	1	High-quality	100	0	unclassified	unclassified
v11a6	80681	76	8	5	Medium-quality	72.15	0	unclassified	unclassified
v11a7	41465	51	15	0	High-quality	100	0	Siphoviridae	prokaryote
v11a8	100137	108	17	11	High-quality	96.78	0	unclassified	unclassified
v11a9	38380	53	7	1	Medium-quality	59.12	0	unclassified	unclassified
v11aa	27474	36	2	0	Medium-quality	66.7	0	unclassified	unclassified
v11ab	114823	101	10	11	High-quality	96.33	0	unclassified	unclassified
v11ac	35152	34	0	8	Medium-quality	55.3	0	unclassified	unclassified
v11ad	126359	140	8	29	High-quality	90.26	0	unclassified	unclassified
v11ae	33148	31	1	4	Medium-quality	75.33	0	unclassified	unclassified
v11af	53764	57	3	14	Medium-quality	72.19	0	unclassified	unclassified
v11b0	72875	58	1	19	Medium-quality	84.21	0	unclassified	unclassified
v11b1	120750	121	2	42	High-quality	100	0	unclassified	unclassified
v11b2	36079	41	20	0	Medium-quality	76.66	0	Siphoviridae	prokaryote
v11b3	36219	52	27	0	High-quality	100	0	Myoviridae	prokaryote
v11b4	55265	77	23	0	High-quality	100	0	Siphoviridae	prokaryote
v11b5	36188	40	8	2	Medium-quality	74.76	0	unclassified	unclassified
v11b6	46215	54	11	2	Medium-quality	62.11	0	Siphoviridae	prokaryote
v11b7	115053	115	6	35	Medium-quality	72.5	0	unclassified	unclassified
v11b8	18036	21	5	0	High-quality	100	0	unclassified	unclassified
v11b9	40280	50	12	1	High-quality	93.65	0	unclassified	unclassified
v11ba	31571	37	10	4	Medium-quality	64.55	28.79	unclassified	unclassified
v11bb	94718	105	50	4	High-quality	100	0	Siphoviridae	prokaryote
v11bc	29185	36	22	0	Medium-quality	62.14	0	Siphoviridae	prokaryote
v11bd	94476	118	23	0	Medium-quality	74.4	0	unclassified	unclassified
v11be	40407	59	20	0	Medium-quality	84.84	0	unclassified	unclassified
v11bf	36575	52	11	1	High-quality	100	0	unclassified	unclassified
v11c0	47694	57	10	2	Medium-quality	78.03	0	unclassified	unclassified
v11c1	56488	87	20	0	Medium-quality	87.86	0	unclassified	unclassified
v11c2	25988	38	13	0	Medium-quality	72.26	0	unclassified	unclassified
v11c3	64459	65	2	22	High-quality	100	0	unclassified	unclassified
v11c4	39307	38	1	5	Medium-quality	62.6	0	unclassified	unclassified
v11c5	64245	64	4	10	High-quality	100	0	unclassified	unclassified
v11c6	82740	79	2	24	Medium-quality	85.34	0	unclassified	unclassified
v11c7	218833	217	13	72	High-quality	100	0	unclassified	unclassified
v11c8	57591	57	1	17	Medium-quality	77.39	0	unclassified	unclassified
v11c9	30447	39	1	6	Medium-quality	54.19	0	unclassified	unclassified
v11ca	31110	37	0	11	Medium-quality	51.96	0	unclassified	unclassified
v11cb	34098	44	1	1	Medium-quality	51.01	0	unclassified	unclassified

v11cc	80275	78	1	22	High-quality	100	0	unclassified	unclassified
v11cd	132975	128	2	29	High-quality	100	0	unclassified	unclassified
v11ce	46801	46	0	11	Medium-quality	60.18	0	unclassified	unclassified
v11cf	186246	178	4	50	High-quality	100	0	unclassified	unclassified
v11d0	45031	45	2	14	Medium-quality	50.81	0	unclassified	unclassified
v11d1	86641	90	7	16	High-quality	100	0	unclassified	unclassified
v11d2	81599	82	8	10	High-quality	91.87	0	unclassified	unclassified
v11d3	64927	84	9	14	Medium-quality	86.81	0	unclassified	unclassified
v11d4	45972	46	1	12	Medium-quality	69.56	0	unclassified	unclassified
v11d5	8651	8	1	2	High-quality	100	0	unclassified	unclassified
v11d6	5144	10	0	0	High-quality	100	0	Inoviridae	prokaryote
v11d7	56305	60	1	11	Medium-quality	52.4	0	unclassified	unclassified
v11d8	38479	52	18	1	High-quality	90.33	0	Siphoviridae	prokaryote
v11d9	44141	62	8	1	High-quality	100	0	unclassified	unclassified
v11da	59570	69	24	2	High-quality	100	0	unclassified	unclassified
v11db	35012	53	18	0	High-quality	91.18	0	Siphoviridae	prokaryote
v11dc	30296	53	20	0	Medium-quality	81.16	0	Siphoviridae	prokaryote
v11dd	40802	74	20	0	High-quality	96.82	0	unclassified	unclassified
v11de	81032	100	14	13	Medium-quality	56.75	60.33	unclassified	unclassified
v11df	31823	49	18	0	High-quality	93.15	0	Siphoviridae	prokaryote
v11e0	70632	66	8	5	High-quality	100	0	unclassified	unclassified
v11e1	40757	62	47	0	High-quality	97.42	0	Myoviridae	prokaryote
v11e2	34434	50	17	1	High-quality	100	0	Siphoviridae	prokaryote
v11e3	53069	48	6	1	High-quality	100	0	unclassified	unclassified
v11e4	30120	42	9	0	Medium-quality	51.57	0	unclassified	unclassified
v11e5	37239	40	9	0	Medium-quality	66.07	0	Quimbyviridae	prokaryote
v11e6	38236	52	28	0	High-quality	100	0	Siphoviridae	prokaryote
v11e7	39885	54	36	0	High-quality	99.27	0	Podoviridae	prokaryote
v11e8	73036	70	10	6	High-quality	100	0	unclassified	unclassified
v11e9	79100	104	19	8	High-quality	100	0	unclassified	unclassified
v11ea	82518	120	14	2	High-quality	93.69	0	Quimbyviridae	prokaryote
v11eb	30941	53	18	1	High-quality	100	0	Siphoviridae	prokaryote
v11ec	59969	71	15	6	Medium-quality	79.04	0	unclassified	unclassified
v11ed	54090	91	19	0	High-quality	100	0	unclassified	unclassified
v11ee	21821	15	3	0	Medium-quality	54.65	0	unclassified	unclassified
v11ef	38403	44	26	3	Medium-quality	81.29	0	Siphoviridae	prokaryote
v11f0	34430	52	14	0	Medium-quality	78.97	0	unclassified	unclassified
v11f1	47206	57	8	5	Medium-quality	80.26	31.07	unclassified	unclassified
v11f2	17704	22	4	1	Medium-quality	61.71	0	unclassified	unclassified
v11f3	42941	47	22	0	High-quality	100	0	Podoviridae	prokaryote

v11f4	35959	50	25	4	Medium-quality	76.7	0	Siphoviridae	prokaryote
v11f5	23291	37	9	0	Medium-quality	57.39	0	Siphoviridae	prokaryote
v11f6	67728	76	27	6	High-quality	98.49	16.51	Siphoviridae	prokaryote
v11f7	201711	310	55	4	High-quality	100	0	Siphoviridae	prokaryote
v11f8	64839	82	22	5	High-quality	100	0	unclassified	unclassified
v11f9	34553	51	18	1	High-quality	96.65	0	Siphoviridae	prokaryote
v11fa	66612	87	19	2	High-quality	100	0	unclassified	unclassified
v11fb	24071	39	22	0	Medium-quality	71.2	0	Siphoviridae	prokaryote
v11fc	34424	49	13	0	High-quality	98.81	0	unclassified	unclassified
v11fd	39930	56	32	0	High-quality	100	0	Siphoviridae	prokaryote
v11fe	31169	41	22	0	Medium-quality	88.76	0	Siphoviridae	prokaryote
v11ff	15194	23	4	0	Medium-quality	52.15	0	unclassified	unclassified
v1200	37919	50	13	0	High-quality	93.35	0	unclassified	unclassified
v1201	18570	31	7	0	High-quality	100	0	unclassified	unclassified
v1202	44570	53	35	0	High-quality	97.4	0	Siphoviridae	prokaryote
v1203	104605	146	31	2	High-quality	100	0	unclassified	unclassified
v1204	124081	132	10	21	High-quality	98.6	0	unclassified	unclassified
v1205	103861	102	10	32	High-quality	100	0	unclassified	unclassified
v1206	50570	58	1	15	Medium-quality	84.47	0	unclassified	unclassified
v1207	212716	308	22	30	High-quality	100	0	unclassified	unclassified
v1208	114607	101	1	23	Medium-quality	62.16	0	unclassified	unclassified
v1209	37079	37	3	7	Medium-quality	86.78	0	unclassified	unclassified
v120a	47257	59	1	18	Medium-quality	78.44	0	unclassified	unclassified
v120b	36948	56	3	3	Medium-quality	66.87	0	Myoviridae	prokaryote
v120c	152637	152	1	42	High-quality	100	0	unclassified	unclassified
v120d	81476	89	1	23	Medium-quality	58.7	0	unclassified	unclassified
v120e	62901	71	8	2	High-quality	99.8	0	unclassified	unclassified
v120f	40693	61	47	1	High-quality	100	0	Podoviridae	prokaryote
v1210	60604	92	21	0	High-quality	100	0	Myoviridae	prokaryote
v1211	42775	64	8	2	Medium-quality	88.37	0	unclassified	unclassified
v1212	29938	42	17	0	Medium-quality	67.76	0	Siphoviridae	prokaryote
v1213	31803	45	30	0	Medium-quality	78.08	0	Siphoviridae	prokaryote
v1214	31160	47	13	1	Medium-quality	78.26	0	Siphoviridae	prokaryote
v1215	70243	108	14	6	High-quality	100	0	unclassified	unclassified
v1216	37637	50	30	0	High-quality	100	0	Siphoviridae	prokaryote
v1217	105699	125	23	10	High-quality	100	0	unclassified	unclassified
v1218	60044	68	8	1	High-quality	94.66	0	unclassified	unclassified
v1219	39486	50	32	2	High-quality	97.83	12.83	Myoviridae	prokaryote
v121a	36303	44	6	1	Medium-quality	87.43	0	unclassified	unclassified
v121b	53277	59	8	1	Medium-quality	60.6	0	Quimbyviridae	prokaryote

v121c	41600	53	12	2	High-quality	100	0	unclassified	unclassified
v121d	172866	161	9	43	High-quality	100	0	unclassified	unclassified
v121e	45877	56	17	2	High-quality	100	0	unclassified	unclassified
v121f	49080	67	13	2	High-quality	100	0	unclassified	unclassified
v1220	31479	52	17	0	Medium-quality	65.81	0	Siphoviridae	prokaryote
v1221	113788	178	29	1	High-quality	100	0	unclassified	unclassified
v1222	61279	91	15	1	Medium-quality	69.68	0	Quimbyviridae	prokaryote
v1223	75855	115	18	8	High-quality	100	0	unclassified	unclassified
v1224	48514	69	8	1	Medium-quality	88.01	0	unclassified	unclassified
v1225	42081	57	8	0	High-quality	92.39	0	unclassified	unclassified
v1226	36794	58	13	0	High-quality	90.69	0	unclassified	unclassified
v1227	41208	50	13	4	Complete	100	17.64	unclassified	unclassified
v1228	41726	59	18	0	High-quality	92.53	0	unclassified	unclassified
v1229	77438	87	23	0	High-quality	94.06	0	Siphoviridae	prokaryote
v122a	38360	52	16	3	High-quality	100	0	Siphoviridae	prokaryote
v122b	99527	101	5	19	Medium-quality	80.92	0	unclassified	unclassified
v122c	45590	51	1	3	Medium-quality	65.47	0	unclassified	unclassified
v122d	26921	34	1	10	Medium-quality	81.52	0	unclassified	unclassified
v122e	30349	36	12	1	Medium-quality	62.99	0	Siphoviridae	prokaryote
v122f	47081	54	9	1	Medium-quality	69.99	0	unclassified	unclassified
v1230	34826	44	25	0	High-quality	100	0	Myoviridae	prokaryote
v1231	41636	69	49	0	Medium-quality	86.88	0	Siphoviridae	prokaryote
v1232	54243	96	17	0	High-quality	94.02	0	unclassified	unclassified
v1233	34932	47	15	1	Medium-quality	83.3	0	Siphoviridae	prokaryote
v1234	32532	48	16	0	Medium-quality	80.09	0	Siphoviridae	prokaryote
v1235	47130	72	23	0	High-quality	95.74	0	Myoviridae	prokaryote
v1236	37022	53	25	0	High-quality	94.75	0	Siphoviridae	prokaryote
v1237	66678	116	32	1	High-quality	92.51	0	Myoviridae	prokaryote
v1238	13032	25	3	0	High-quality	90.48	0	unclassified	unclassified
v1239	38577	51	32	0	High-quality	92.11	0	Myoviridae	prokaryote
v123a	48505	53	18	2	High-quality	90.35	0	unclassified	unclassified
v123b	76027	99	4	15	Medium-quality	89.35	0	unclassified	unclassified
v123c	33572	41	1	7	Medium-quality	62.63	0	unclassified	unclassified
v123d	83104	88	3	15	High-quality	100	0	unclassified	unclassified
v123e	38270	43	1	15	Medium-quality	59.94	0	unclassified	unclassified
v123f	71379	67	3	16	Complete	100	0	unclassified	unclassified
v1240	35867	56	16	0	High-quality	100	0	unclassified	unclassified
v1241	32779	51	21	0	Medium-quality	66.1	0	unclassified	unclassified
v1242	52887	58	8	4	Medium-quality	77.88	0	unclassified	unclassified
v1243	44917	59	11	1	High-quality	100	0	unclassified	unclassified

v1244	65596	95	14	1	Medium-quality	74.68	0	Quimbyviridae	prokaryote
v1245	46797	67	19	2	High-quality	100	0	Siphoviridae	prokaryote
v1246	58168	91	17	2	High-quality	100	0	unclassified	unclassified
v1247	16759	15	3	1	High-quality	100	0	unclassified	unclassified
v1248	67593	90	22	2	High-quality	100	0	Myoviridae	prokaryote
v1249	63358	79	16	8	High-quality	100	0	unclassified	unclassified
v124a	5117	7	5	0	Complete	100	0	Microviridae	prokaryote
v124b	38656	45	6	4	Medium-quality	88.7	0	unclassified	unclassified
v124c	156541	207	18	4	Complete	100	0	unclassified	unclassified
v124d	46372	59	15	1	High-quality	90.36	0	unclassified	unclassified
v124e	16309	18	6	0	Medium-quality	77.59	0	unclassified	unclassified
v124f	58797	69	8	1	Medium-quality	80.92	0	unclassified	unclassified
v1250	32312	37	3	1	Medium-quality	53.24	0	unclassified	unclassified
v1251	52544	73	13	1	Complete	100	0	Siphoviridae	prokaryote
v1252	17590	25	6	0	High-quality	92.38	0	unclassified	unclassified
v1253	83361	122	14	2	Medium-quality	76.53	0	unclassified	unclassified
v1254	80912	127	22	6	Complete	100	0	unclassified	unclassified
v1255	50848	66	42	4	Complete	100	0	Myoviridae	prokaryote
v1256	42711	72	15	0	High-quality	98.46	0	unclassified	unclassified
v1257	43811	70	15	0	Complete	100	0	unclassified	unclassified
v1258	15804	19	6	0	Medium-quality	54.29	0	unclassified	unclassified
v1259	27158	43	15	3	Medium-quality	64.16	0	unclassified	unclassified
v125a	25320	32	7	1	Medium-quality	51.44	0	Myoviridae	prokaryote
v125b	73843	85	34	7	High-quality	100	0	Siphoviridae	prokaryote
v125c	42183	54	6	0	High-quality	100	0	unclassified	unclassified
v125d	100538	104	16	1	High-quality	100	0	Podoviridae_crAss-like	prokaryote
v125e	85753	115	33	6	High-quality	100	0	Siphoviridae	prokaryote
v125f	98709	161	19	1	Complete	100	0	Podoviridae_crAss-like	prokaryote
v1260	24425	27	1	0	Medium-quality	51.89	0	unclassified	unclassified
v1261	12756	12	2	0	High-quality	100	0	unclassified	unclassified
v1262	16791	20	10	0	Medium-quality	73.18	0	Salasmaviridae	prokaryote
v1263	158183	200	17	4	Complete	100	0	unclassified	unclassified
v1264	41357	49	47	0	Complete	100	0	Autographiviridae	prokaryote
v1265	42373	51	20	0	Medium-quality	85.22	0	unclassified	unclassified
v1266	22201	30	24	0	Medium-quality	68.31	0	Siphoviridae	prokaryote
v1267	167487	244	35	4	Complete	100	0	unclassified	unclassified
v1268	30672	37	15	1	Medium-quality	74.35	0	Siphoviridae	prokaryote
v1269	6257	8	3	0	High-quality	97.76	0	Microviridae	prokaryote
v126a	42241	65	10	0	Medium-quality	64.28	0	unclassified	unclassified
v126b	43986	57	14	3	Medium-quality	78.67	0	unclassified	unclassified

v126c	29881	50	10	0	Medium-quality	50.38	0	unclassified	unclassified
v126d	39088	48	47	0	Complete	100	0	Autographiviridae	prokaryote
v126e	18355	13	1	0	Medium-quality	63.98	0	unclassified	unclassified
v126f	6123	9	2	0	Complete	100	0	unclassified	unclassified
v1270	25774	36	23	0	Medium-quality	66.25	0	Siphoviridae	prokaryote
v1271	55358	63	21	2	High-quality	100	0	Siphoviridae	prokaryote
v1272	61030	105	23	2	Complete	100	0	unclassified	unclassified
v1273	24820	50	9	0	Medium-quality	53	0	unclassified	unclassified
v1274	15877	21	6	0	Medium-quality	68.64	0	unclassified	unclassified
v1275	17221	24	8	0	Medium-quality	87.22	0	unclassified	unclassified
v1276	18701	43	10	0	Medium-quality	51.85	0	unclassified	unclassified
v1277	44757	66	20	0	High-quality	90	0	unclassified	unclassified
v1278	17923	24	10	0	Medium-quality	77.72	0	Salasmaviridae	prokaryote
v1279	100149	129	13	0	Complete	100	0	Podoviridae_crAss-like	prokaryote
v127a	36129	59	14	0	Medium-quality	58	0	unclassified	unclassified
v127b	51954	66	15	1	Complete	100	0	unclassified	unclassified
v127c	52048	91	13	0	High-quality	99.93	0	unclassified	unclassified
v127d	46552	49	27	1	Complete	100	0	Siphoviridae	prokaryote
v127e	15833	18	5	0	Medium-quality	52.71	0	unclassified	unclassified
v127f	23757	37	8	1	Medium-quality	50.81	0	unclassified	unclassified
v1280	8578	11	2	0	Medium-quality	71.82	0	unclassified	unclassified
v1281	80462	105	47	5	High-quality	100	0	Siphoviridae	prokaryote
v1282	46945	65	13	3	High-quality	100	0	unclassified	unclassified
v1283	20493	28	18	0	Medium-quality	54.1	0	Siphoviridae	prokaryote
v1284	23110	34	23	0	Medium-quality	51.58	0	Siphoviridae	prokaryote
v1285	58128	71	33	0	Complete	100	0	Siphoviridae	prokaryote
v1286	58610	64	24	2	High-quality	98.56	0	Siphoviridae	prokaryote
v1287	19337	22	16	0	Medium-quality	53.21	0	Siphoviridae	prokaryote
v1288	43938	49	12	1	High-quality	100	0	unclassified	unclassified
v1289	21387	35	19	0	Medium-quality	58.71	0	Siphoviridae	prokaryote
v128a	40504	71	15	0	Complete	100	0	unclassified	unclassified
v128b	38373	58	11	0	High-quality	94.99	0	unclassified	unclassified
v128c	80459	88	8	7	High-quality	100	0	unclassified	unclassified
v128d	81213	129	28	2	Complete	100	0	unclassified	unclassified
v128e	94244	94	11	1	Complete	100	0	Podoviridae_crAss-like	prokaryote
v128f	21964	26	10	0	Medium-quality	52.77	0	Quimbyviridae	prokaryote
v1290	45770	70	49	0	Complete	100	0	Siphoviridae	prokaryote
v1291	50690	65	18	4	High-quality	100	0	Siphoviridae	prokaryote
v1292	26467	45	31	0	Medium-quality	57.13	0	Siphoviridae	prokaryote
v1293	143360	197	45	4	High-quality	100	0	unclassified	unclassified

v1294	47626	59	25	2	High-quality	100	0	Myoviridae	prokaryote
v1295	59195	82	54	0	High-quality	100	0	Siphoviridae	prokaryote
v1296	41219	54	32	0	Medium-quality	87.29	0	Siphoviridae	prokaryote
v1297	155389	195	19	3	Complete	100	0	unclassified	unclassified
v1298	178436	316	20	2	High-quality	100	0	unclassified	unclassified
v1299	167075	211	26	0	Complete	100	0	unclassified	unclassified
v129a	12660	21	4	1	Medium-quality	55	0	unclassified	unclassified
v129b	47976	70	14	1	High-quality	100	0	unclassified	unclassified
v129c	69228	116	22	3	Medium-quality	85.79	0	unclassified	unclassified
v129d	8405	9	3	0	High-quality	100	0	unclassified	unclassified
v129e	42167	77	18	0	Complete	100	0	unclassified	unclassified
v129f	39176	55	7	0	Complete	100	0	unclassified	unclassified
v12a0	39611	47	21	2	Medium-quality	58.36	0	Siphoviridae	prokaryote
v12a1	37061	48	8	6	Medium-quality	76.57	0	unclassified	unclassified
v12a2	77127	125	17	5	Complete	100	0	unclassified	unclassified
v12a3	27735	36	30	0	Medium-quality	68.56	0	Podoviridae	prokaryote
v12a4	9932	18	2	0	Medium-quality	71.72	0	unclassified	unclassified
v12a5	27569	39	24	1	Medium-quality	59.9	0	Siphoviridae	prokaryote
v12a6	26868	35	29	0	Medium-quality	66.69	0	Podoviridae	prokaryote
v12a7	41751	63	29	0	High-quality	98.51	0	Siphoviridae	prokaryote
v12a8	42436	76	18	0	High-quality	90.46	0	unclassified	unclassified
v12a9	15874	24	8	0	Medium-quality	56.01	0	unclassified	unclassified
v12aa	45971	71	14	0	Complete	100	0	unclassified	unclassified
v12ab	46599	63	37	8	High-quality	100	0	Myoviridae	prokaryote
v12ac	41868	63	24	0	High-quality	100	0	Siphoviridae	prokaryote
v12ad	6516	8	3	0	Complete	100	0	unclassified	unclassified
v12ae	26391	36	18	1	Medium-quality	65.47	0	Siphoviridae	prokaryote
v12af	43458	70	21	4	Complete	100	0	unclassified	unclassified
v12b0	17953	25	7	0	High-quality	99.93	0	Podoviridae	prokaryote
v12b1	51396	88	18	0	Complete	100	0	unclassified	unclassified
v12b2	40807	73	19	2	Medium-quality	70.27	0	unclassified	unclassified
v12b3	69896	102	20	1	Complete	100	0	unclassified	unclassified
v12b4	55730	103	16	2	Complete	100	0	unclassified	unclassified
v12b5	25200	42	12	0	Medium-quality	68.57	0	unclassified	unclassified
v12b6	38984	57	34	2	Medium-quality	85.18	0	Siphoviridae	prokaryote
v12b7	55483	66	7	3	High-quality	91.15	0	unclassified	unclassified
v12b8	43466	76	21	0	Complete	100	0	unclassified	unclassified
v12b9	46021	80	31	0	High-quality	100	0	unclassified	unclassified
v12ba	45388	53	9	0	Complete	100	0	Siphoviridae	prokaryote
v12bb	6085	3	1	0	Complete	100	0	unclassified	unclassified

v12bc	44090	52	28	2	High-quality	100	0	Siphoviridae	prokaryote
v12bd	34443	49	9	0	Complete	100	0	unclassified	unclassified
v12be	42013	67	16	1	High-quality	94.86	0	unclassified	unclassified
v12bf	6337	7	3	0	Complete	100	0	unclassified	unclassified
v12c0	68644	84	15	2	High-quality	100	0	unclassified	unclassified
v12c1	52364	65	15	7	High-quality	100	0	unclassified	unclassified
v12c2	62391	64	7	0	Complete	100	0	unclassified	unclassified
v12c3	40089	56	23	0	High-quality	99.45	0	Siphoviridae	prokaryote
v12c4	61153	75	13	7	High-quality	100	0	Siphoviridae	prokaryote
v12c5	43678	65	20	0	Complete	100	0	unclassified	unclassified
v12c6	12706	15	6	1	Medium-quality	62	0	Podoviridae	prokaryote
v12c7	136679	166	13	6	Medium-quality	62.92	0	unclassified	unclassified
v12c8	87194	116	17	3	Complete	100	0	Flandersviridae	prokaryote
v12c9	96649	111	22	2	High-quality	99.62	0	unclassified	unclassified
v12ca	15370	22	9	0	Medium-quality	80.96	0	unclassified	unclassified
v12cb	48616	48	8	3	Medium-quality	88.2	0	unclassified	unclassified
v12cc	20053	37	5	1	Medium-quality	50.87	0	Quimbyviridae	prokaryote
v12cd	30354	46	15	1	Medium-quality	76.32	0	Siphoviridae	prokaryote
v12ce	81403	99	13	10	High-quality	94.72	0	unclassified	unclassified
v12cf	78387	107	20	9	High-quality	100	0	Myoviridae	prokaryote
v12d0	50603	82	70	0	Complete	100	0	Drexlerviridae	prokaryote
v12d1	49865	77	69	0	Complete	100	0	Drexlerviridae	prokaryote
v12d2	20203	36	17	0	Medium-quality	52.18	0	Siphoviridae	prokaryote
v12d3	144612	166	20	17	High-quality	100	0	Quimbyviridae	prokaryote
v12d4	158243	239	38	3	Complete	100	0	unclassified	unclassified
v12d5	42828	72	18	1	Complete	100	0	Siphoviridae	prokaryote
v12d6	26732	39	14	1	Medium-quality	75.67	0	Siphoviridae	prokaryote
v12d7	16898	21	8	0	Medium-quality	78.77	0	unclassified	unclassified
v12d8	46683	50	7	3	Medium-quality	84.43	0	unclassified	unclassified
v12d9	40600	68	23	5	Medium-quality	62.43	0	Siphoviridae	prokaryote
v12da	119702	139	26	23	High-quality	100	0	unclassified	unclassified
v12db	22361	25	6	1	Medium-quality	51.1	0	unclassified	unclassified
v12dc	34767	46	11	0	High-quality	91.17	0	Siphoviridae	prokaryote
v12dd	22007	31	23	0	Medium-quality	62.59	0	Myoviridae	prokaryote
v12de	38825	56	43	1	High-quality	100	0	Siphoviridae	prokaryote
v12df	6396	8	3	0	Complete	100	0	unclassified	unclassified
v12e0	42916	72	19	0	High-quality	99.48	0	unclassified	unclassified
v12e1	5046	6	1	0	High-quality	100	0	unclassified	unclassified
v12e2	66598	69	22	1	Medium-quality	79.55	0	Siphoviridae	prokaryote
v12e3	71590	112	18	4	Complete	100	0	unclassified	unclassified

v12e4	56751	74	38	2	High-quality	100	0	Siphoviridae	prokaryote
v12e5	28699	26	6	1	Medium-quality	54.3	0	Siphoviridae	prokaryote
v12e6	25012	41	14	3	Medium-quality	70.87	0	unclassified	unclassified
v12e7	38090	57	10	2	High-quality	100	0	unclassified	unclassified
v12e8	37166	61	18	2	High-quality	100	0	unclassified	unclassified
v12e9	38693	60	32	0	Complete	100	0	Siphoviridae	prokaryote
v12ea	15677	20	8	0	Medium-quality	70.25	0	unclassified	unclassified
v12eb	25692	36	6	1	Medium-quality	73.4	0	unclassified	unclassified
v12ec	39311	42	6	4	Medium-quality	81.22	0	unclassified	unclassified
v12ed	16789	24	8	0	Medium-quality	89.8	0	unclassified	unclassified
v12ee	23431	39	5	2	Medium-quality	59.91	0	unclassified	unclassified
v12ef	37771	57	26	0	Medium-quality	89.24	0	Myoviridae	prokaryote
v12f0	16660	19	8	0	Medium-quality	89.05	0	Salasmaviridae	prokaryote
v12f1	40510	48	18	1	High-quality	99.34	0	Siphoviridae	prokaryote
v12f2	26454	24	20	0	Medium-quality	70.46	0	Siphoviridae	prokaryote
v12f3	37900	63	47	0	Complete	100	0	Podoviridae	prokaryote
v12f4	37740	59	16	1	High-quality	100	0	Siphoviridae	prokaryote
v12f5	45033	67	17	0	Complete	100	0	unclassified	unclassified
v12f6	35866	51	35	0	Medium-quality	88.31	0	Podoviridae	prokaryote
v12f7	57340	78	20	5	High-quality	100	0	Siphoviridae	prokaryote
v12f8	39089	65	19	0	High-quality	95.79	0	unclassified	unclassified
v12f9	22832	29	3	2	Medium-quality	79.59	0	unclassified	unclassified
v12fa	81273	95	16	0	High-quality	99.12	0	Flandersviridae	prokaryote
v12fb	18274	22	8	0	High-quality	100	0	Podoviridae	prokaryote
v12fc	50566	68	6	3	Medium-quality	57.4	0	Quimbyviridae	prokaryote
v12fd	19584	30	7	0	High-quality	100	0	unclassified	unclassified
v12fe	85266	87	10	5	High-quality	96.63	0	unclassified	unclassified
v12ff	17607	29	6	0	Medium-quality	74.97	0	unclassified	unclassified
v1300	41097	72	13	0	High-quality	100	0	unclassified	unclassified
v1301	41694	55	16	2	High-quality	100	0	Siphoviridae	prokaryote
v1302	13631	18	5	0	High-quality	100	0	unclassified	unclassified
v1303	44861	76	17	2	High-quality	100	0	unclassified	unclassified
v1304	70625	74	8	5	High-quality	100	0	unclassified	unclassified
v1305	51694	82	22	0	Complete	100	0	unclassified	unclassified
v1306	38743	56	9	1	Complete	100	0	unclassified	unclassified
v1307	6852	7	1	0	High-quality	100	0	Siphoviridae	prokaryote
v1308	33788	46	12	0	Complete	100	0	unclassified	unclassified
v1309	43141	56	9	1	Complete	100	0	Siphoviridae	prokaryote
v130a	51989	73	45	2	High-quality	90.35	0	Siphoviridae	prokaryote
v130b	42752	68	19	0	High-quality	94.95	0	unclassified	unclassified

v130c	43721	71	16	1	High-quality	100	0	unclassified	unclassified
v130d	16504	21	6	0	Medium-quality	50.02	0	unclassified	unclassified
v130e	92657	90	9	1	High-quality	98.54	0	Podoviridae_crAss-like	prokaryote
v130f	41529	61	23	3	High-quality	99.97	0	Siphoviridae	prokaryote
v1310	47297	75	17	0	High-quality	100	0	unclassified	unclassified
v1311	38120	65	12	0	High-quality	100	0	unclassified	unclassified
v1312	209834	308	44	6	High-quality	97.41	0	unclassified	unclassified
v1313	83481	126	31	3	Complete	100	0	unclassified	unclassified
v1314	67467	78	15	2	High-quality	100	0	unclassified	unclassified
v1315	45826	50	8	2	High-quality	92.08	0	unclassified	unclassified
v1316	92628	123	20	3	Complete	100	0	unclassified	unclassified
v1317	44608	62	32	1	Complete	100	0	Siphoviridae	prokaryote
v1318	23756	43	15	1	Medium-quality	53.9	0	Siphoviridae	prokaryote
v1319	17108	31	12	0	Medium-quality	50.16	0	Siphoviridae	prokaryote
v131a	30199	37	10	1	Medium-quality	73.2	0	unclassified	unclassified
v131b	15893	20	5	0	Medium-quality	52.91	0	unclassified	unclassified
v131c	47392	54	11	0	Complete	100	0	Siphoviridae	prokaryote
v131d	42018	46	7	1	Medium-quality	73.2	0	unclassified	unclassified
v131e	24604	31	13	0	Medium-quality	51.42	0	Siphoviridae	prokaryote
v131f	61587	104	21	3	High-quality	100	0	unclassified	unclassified
v1320	52619	42	4	2	High-quality	100	0	unclassified	unclassified
v1321	146144	221	38	5	High-quality	99.8	0	unclassified	unclassified
v1322	18566	21	3	2	Medium-quality	64.71	0	unclassified	unclassified
v1323	46209	75	19	0	Complete	100	0	unclassified	unclassified
v1324	47076	57	10	0	Complete	100	0	Siphoviridae	prokaryote
v1325	46296	55	23	0	Complete	100	0	Autographiviridae	prokaryote
v1326	5674	8	1	0	High-quality	100	0	unclassified	unclassified
v1327	34574	53	17	0	Medium-quality	85.63	0	Siphoviridae	prokaryote
v1328	145578	212	38	4	Complete	100	0	unclassified	unclassified
v1329	16187	24	7	0	Medium-quality	53.49	0	unclassified	unclassified
v132a	20924	33	13	0	Medium-quality	53.17	0	Siphoviridae	prokaryote
v132b	21203	37	7	1	Medium-quality	53.85	0	unclassified	unclassified
v132c	33955	39	15	1	Medium-quality	88.25	0	Siphoviridae	prokaryote
v132d	43827	71	16	2	Complete	100	0	unclassified	unclassified
v132e	42881	66	10	1	Complete	100	0	unclassified	unclassified
v132f	102417	159	13	1	Complete	100	0	Podoviridae_crAss-like	prokaryote
v1330	103329	127	13	1	Complete	100	0	unclassified	unclassified
v1331	18212	27	13	0	Medium-quality	50.45	0	Siphoviridae	prokaryote
v1332	16783	23	6	0	Medium-quality	76.98	0	unclassified	unclassified
v1333	58727	86	15	2	High-quality	98.88	0	unclassified	unclassified

v1334	38826	50	26	0	Medium-quality	64.47	0	Siphoviridae	prokaryote
v1335	125364	206	40	5	Medium-quality	76.56	0	unclassified	unclassified
v1336	83124	126	19	1	Complete	100	0	Quimbyviridae	prokaryote
v1337	121290	169	14	3	Medium-quality	78.83	0	unclassified	unclassified
v1338	29966	34	4	2	Medium-quality	77.95	0	unclassified	unclassified
v1339	139927	191	34	5	High-quality	100	0	unclassified	unclassified
v133a	26011	34	23	0	Medium-quality	53.21	0	Siphoviridae	prokaryote
v133b	53228	80	17	7	High-quality	100	0	unclassified	unclassified
v133c	16580	24	8	0	Complete	100	0	unclassified	unclassified
v133d	33030	51	17	0	Medium-quality	80.01	0	unclassified	unclassified
v133e	47035	55	11	4	High-quality	100	0	unclassified	unclassified
v133f	40826	54	29	0	Complete	100	0	Myoviridae	prokaryote
v1340	38733	52	28	0	High-quality	94.77	0	Siphoviridae	prokaryote
v1341	10233	13	4	0	Medium-quality	76.6	0	unclassified	unclassified
v1342	87033	105	9	7	High-quality	93.94	0	unclassified	unclassified
v1343	75740	124	20	3	High-quality	98.79	0	unclassified	unclassified
v1344	21092	36	14	0	Medium-quality	51.52	0	unclassified	unclassified
v1345	74224	79	14	8	Medium-quality	86.04	0	unclassified	unclassified
v1346	180260	233	37	12	High-quality	100	0	unclassified	unclassified
v1347	38128	69	12	2	Medium-quality	66.11	0	unclassified	unclassified
v1348	67502	78	23	6	High-quality	100	0	Siphoviridae	prokaryote
v1349	45639	64	8	3	Medium-quality	66	0	unclassified	unclassified
v134a	41817	52	8	0	High-quality	92.79	0	Siphoviridae	prokaryote
v134b	40862	65	19	1	High-quality	95.25	0	unclassified	unclassified
v134c	34468	48	16	0	High-quality	97.53	0	unclassified	unclassified
v134d	150296	208	29	3	Medium-quality	88.6	0	unclassified	unclassified
v134e	36553	54	28	0	Complete	100	0	Siphoviridae	prokaryote
v134f	15361	22	7	0	Medium-quality	67.68	0	unclassified	unclassified
v1350	32808	49	19	0	Complete	100	0	Myoviridae	prokaryote
v1351	27633	46	11	0	Medium-quality	71.99	0	unclassified	unclassified
v1352	38810	57	8	1	Complete	100	0	unclassified	unclassified
v1353	72300	96	13	2	Medium-quality	76.42	0	Quimbyviridae	prokaryote
v1354	52179	86	15	1	High-quality	90.55	0	unclassified	unclassified
v1355	43607	56	14	0	Complete	100	0	unclassified	unclassified
v1356	53586	52	8	5	High-quality	100	0	unclassified	unclassified
v1357	38028	41	11	4	High-quality	96.09	0	unclassified	unclassified
v1358	26041	39	26	0	Medium-quality	61.3	0	Myoviridae	prokaryote
v1359	34117	57	17	0	Medium-quality	82.96	0	unclassified	unclassified
v135a	100868	110	20	2	High-quality	96.75	0	Gratiaviridae	prokaryote
v135b	39760	53	15	0	Complete	100	0	unclassified	unclassified

v135c	64138	61	8	7	High-quality	100	0	unclassified	unclassified
v135d	12976	20	6	0	Low-quality	43.67	0	Salasmaviridae	prokaryote
v135e	17351	24	8	0	High-quality	92.75	0	Salasmaviridae	prokaryote
v135f	75426	130	22	1	Medium-quality	70.73	0	unclassified	unclassified
v1360	33349	46	3	0	Medium-quality	80.28	0	unclassified	unclassified
v1361	45048	77	8	5	Medium-quality	56.97	0	unclassified	unclassified
v1362	6391	8	3	0	Complete	100	0	unclassified	unclassified
v1363	48000	87	16	0	Complete	100	0	unclassified	unclassified
v1364	37149	40	4	1	Low-quality	43.3	0	unclassified	unclassified
v1365	41893	57	24	0	High-quality	99.14	0	Siphoviridae	prokaryote
v1366	59498	104	25	0	Complete	100	0	unclassified	unclassified
v1367	43397	60	8	2	Complete	100	0	unclassified	unclassified
v1368	28586	47	13	0	Medium-quality	77.92	0	Siphoviridae	prokaryote
v1369	36459	56	11	1	Medium-quality	81.4	0	unclassified	unclassified
v136a	22931	39	16	1	Medium-quality	67.67	0	Siphoviridae	prokaryote
v136b	93118	140	33	3	Complete	100	0	unclassified	unclassified
v136c	34520	45	17	1	Medium-quality	70.64	0	Siphoviridae	prokaryote
v136d	41026	57	14	3	Complete	100	0	unclassified	unclassified
v136e	53870	66	5	0	Medium-quality	56.9	0	unclassified	unclassified
v136f	99702	157	11	1	Complete	100	0	Podoviridae_crAss-like	prokaryote
v1370	101323	173	19	3	Complete	100	0	Podoviridae_crAss-like	prokaryote
v1371	43060	77	17	3	Complete	100	0	unclassified	unclassified
v1372	36649	62	21	0	Complete	100	0	unclassified	unclassified
v1373	26907	49	28	0	Medium-quality	55.83	0	Siphoviridae	prokaryote
v1374	43055	62	29	0	High-quality	92.53	0	Siphoviridae	prokaryote
v1375	46919	74	27	0	Complete	100	0	Siphoviridae	prokaryote
v1376	9759	9	7	0	High-quality	100	0	Microviridae	prokaryote
v1377	113625	129	9	8	High-quality	100	0	unclassified	unclassified
v1378	62431	106	21	1	Medium-quality	77.54	0	unclassified	unclassified
v1379	17588	32	11	0	Medium-quality	51.51	0	Siphoviridae	prokaryote
v137a	34365	58	22	0	Complete	100	0	Siphoviridae	prokaryote
v137b	34783	59	20	1	Complete	100	0	Siphoviridae	prokaryote
v137c	31389	53	10	2	Medium-quality	57.17	0	unclassified	unclassified
v137d	40095	64	10	1	High-quality	98.62	0	unclassified	unclassified
v137e	20782	28	6	0	Medium-quality	64.92	0	unclassified	unclassified
v137f	34904	37	6	2	Medium-quality	82.53	0	unclassified	unclassified
v1380	49300	60	7	4	High-quality	100	0	unclassified	unclassified
v1381	60648	82	55	0	Complete	100	0	Siphoviridae	prokaryote
v1382	36508	55	10	0	Complete	100	0	unclassified	unclassified
v1383	41726	76	18	2	Complete	100	0	unclassified	unclassified

v1384	37311	48	29	0	High-quality	91.71	0	Myoviridae	prokaryote
v1385	47545	75	46	0	High-quality	100	0	Siphoviridae	prokaryote
v1386	13007	20	5	0	High-quality	93.65	0	unclassified	unclassified
v1387	37203	54	11	0	Medium-quality	83.05	0	unclassified	unclassified
v1388	62224	95	31	1	High-quality	100	0	Siphoviridae	prokaryote
v1389	28949	46	3	2	Medium-quality	89.2	0	unclassified	unclassified
v138a	31117	48	22	1	Medium-quality	89.99	0	unclassified	unclassified
v138b	41351	60	30	1	High-quality	92.73	0	Myoviridae	prokaryote
v138c	34680	50	14	1	High-quality	91.85	0	Siphoviridae	prokaryote
v138d	39400	49	34	0	Medium-quality	87.13	0	Siphoviridae	prokaryote
v138e	29997	43	16	1	High-quality	93.22	0	Siphoviridae	prokaryote
v138f	40376	67	30	1	High-quality	96.3	0	unclassified	unclassified
v1390	34536	52	6	3	Medium-quality	87.6	0	unclassified	unclassified
v1391	41172	57	6	0	Complete	100	0	unclassified	unclassified
v1392	53336	91	20	0	High-quality	99.58	0	unclassified	unclassified
v1393	56216	90	28	5	Complete	100	0	Siphoviridae	prokaryote
v1394	41985	65	20	1	Complete	100	0	Siphoviridae	prokaryote
v1395	77873	126	19	1	Complete	100	0	unclassified	unclassified
v1396	6753	13	3	0	Medium-quality	55.69	0	unclassified	unclassified
v1397	62240	101	34	0	High-quality	100	0	Siphoviridae	prokaryote
v1398	57368	61	8	0	Medium-quality	66.95	0	unclassified	unclassified
v1399	54170	69	12	2	Medium-quality	58.65	0	unclassified	unclassified
v139a	31016	49	16	0	Medium-quality	84.82	0	Siphoviridae	prokaryote
v139b	68641	117	10	2	Medium-quality	86.13	0	unclassified	unclassified
v139c	44337	73	18	0	High-quality	100	0	unclassified	unclassified
v139d	45409	57	8	1	Complete	100	0	Siphoviridae	prokaryote
v139e	39759	55	5	1	Medium-quality	58.82	0	Siphoviridae	prokaryote
v139f	31795	46	19	1	Medium-quality	88.93	0	Siphoviridae	prokaryote
v13a0	35806	46	27	0	High-quality	94.04	0	Siphoviridae	prokaryote
v13a1	42197	58	19	0	High-quality	100	0	unclassified	unclassified
v13a2	34893	47	17	0	Medium-quality	62.42	0	unclassified	unclassified
v13a3	27773	29	10	2	Medium-quality	75.81	0	Myoviridae	prokaryote
v13a4	69100	114	19	3	Medium-quality	85.93	0	unclassified	unclassified
v13a5	35609	52	26	0	Medium-quality	57.48	0	Siphoviridae	prokaryote
v13a6	38334	63	20	1	Complete	100	0	Siphoviridae	prokaryote
v13a7	53872	72	22	3	Complete	100	0	Siphoviridae	prokaryote
v13a8	24757	38	10	0	Medium-quality	82.11	0	Siphoviridae	prokaryote
v13a9	31275	44	13	2	Medium-quality	64.21	0	Myoviridae	prokaryote
v13aa	41448	63	23	0	High-quality	95.9	0	Myoviridae	prokaryote
v13ab	34152	53	20	1	High-quality	90.97	0	unclassified	unclassified

v13ac	27336	29	2	0	Medium-quality	60.58	0	unclassified	unclassified
v13ad	42839	79	20	0	Complete	100	0	unclassified	unclassified
v13ae	51620	90	16	1	High-quality	90.28	0	unclassified	unclassified
v13af	26899	25	4	1	Medium-quality	63.67	0	unclassified	unclassified
v13b0	33889	43	18	0	Medium-quality	87.06	0	Siphoviridae	prokaryote
v13b1	47530	57	11	0	Complete	100	0	Siphoviridae	prokaryote
v13b2	45935	72	29	1	High-quality	100	0	Siphoviridae	prokaryote
v13b3	14965	21	7	0	Medium-quality	77.57	0	unclassified	unclassified
v13b4	82870	135	27	3	Complete	100	0	unclassified	unclassified
v13b5	5899	8	3	0	Complete	100	0	unclassified	unclassified
v13b6	76618	107	23	3	High-quality	100	0	unclassified	unclassified
v13b7	30463	29	7	0	Medium-quality	72.72	0	unclassified	unclassified
v13b8	41476	77	18	0	Complete	100	0	unclassified	unclassified
v13b9	31445	44	8	1	Medium-quality	64.22	0	unclassified	unclassified
v13ba	130692	168	36	3	Medium-quality	82.87	0	unclassified	unclassified
v13bb	44606	70	45	1	High-quality	100	0	Siphoviridae	prokaryote
v13bc	94566	90	7	0	Complete	100	0	Podoviridae_crAss-like	prokaryote
v13bd	44700	64	16	1	Medium-quality	89.99	0	unclassified	unclassified
v13be	26246	36	11	1	Medium-quality	83.19	0	unclassified	unclassified
v13bf	45865	82	16	3	Complete	100	0	unclassified	unclassified
v13c0	6186	10	2	0	Complete	100	0	unclassified	unclassified
v13c1	70979	118	16	3	Complete	100	0	unclassified	unclassified
v13c2	32069	48	14	0	Complete	100	0	unclassified	unclassified
v13c3	40041	56	12	1	Complete	100	0	unclassified	unclassified
v13c4	35441	64	23	4	High-quality	97.8	0	Siphoviridae	prokaryote
v13c5	43346	57	9	2	Complete	100	0	unclassified	unclassified
v13c6	187325	220	27	7	Complete	100	0	unclassified	unclassified
v13c7	41974	68	17	1	High-quality	93.52	0	Myoviridae	prokaryote
v13c8	32648	49	16	1	High-quality	100	0	Siphoviridae	prokaryote
v13c9	14731	17	7	0	Medium-quality	58.52	0	unclassified	unclassified
v13ca	7983	9	1	0	High-quality	100	0	unclassified	unclassified
v13cb	23850	33	15	1	Medium-quality	52.96	0	Siphoviridae	prokaryote
v13cc	48849	66	15	3	High-quality	100	0	Myoviridae	prokaryote
v13cd	15795	24	8	0	Medium-quality	83.48	0	unclassified	unclassified
v13ce	29074	46	22	0	Medium-quality	71.67	0	Myoviridae	prokaryote
v13cf	38330	56	40	1	High-quality	100	0	Siphoviridae	prokaryote
v13d0	15577	16	8	0	Complete	100	0	unclassified	unclassified
v13d1	67359	103	22	0	Medium-quality	87.23	0	Quimbyviridae	prokaryote
v13d2	32314	52	23	0	Medium-quality	85.29	0	Siphoviridae	prokaryote
v13d3	37446	54	25	0	Medium-quality	86.77	0	Myoviridae	prokaryote

v13d4	19528	25	8	1	Medium-quality	53.88	0	Siphoviridae	prokaryote
v13d5	22067	37	15	0	Medium-quality	66.45	0	Siphoviridae	prokaryote
v13d6	17207	21	2	1	Medium-quality	60.01	0	unclassified	unclassified
v13d7	50990	69	13	1	High-quality	100	0	Siphoviridae	prokaryote
v13d8	6536	9	3	0	Complete	100	0	Microviridae	prokaryote
v13d9	10780	11	1	0	High-quality	100	0	unclassified	unclassified
v13da	46114	78	15	0	Medium-quality	55.27	0	unclassified	unclassified
v13db	66885	91	23	1	High-quality	100	0	unclassified	unclassified
v13dc	41128	56	19	0	High-quality	100	0	Siphoviridae	prokaryote
v13dd	18014	22	4	2	Medium-quality	62.79	0	unclassified	unclassified
v13de	57484	102	17	2	Complete	100	0	unclassified	unclassified
v13df	22202	31	15	0	Medium-quality	56.12	0	Siphoviridae	prokaryote
v13e0	39562	53	10	6	High-quality	100	0	unclassified	unclassified
v13e1	30091	52	25	0	Medium-quality	66.64	0	Myoviridae	prokaryote
v13e2	18128	24	11	0	Medium-quality	55.18	0	Siphoviridae	prokaryote
v13e3	15782	22	7	0	Medium-quality	69.31	0	unclassified	unclassified
v13e4	103747	134	16	5	Complete	100	0	Gratiaviridae	prokaryote
v13e5	10593	17	5	0	High-quality	90	0	unclassified	unclassified
v13e6	79995	123	26	2	High-quality	99.21	0	unclassified	unclassified
v13e7	10852	18	6	0	Medium-quality	80.14	0	unclassified	unclassified
v13e8	37896	51	20	2	High-quality	100	0	Siphoviridae	prokaryote
v13e9	30989	47	14	3	Medium-quality	88.26	0	unclassified	unclassified
v13ea	47106	71	25	0	Complete	100	0	Siphoviridae	prokaryote
v13eb	20489	37	6	3	Medium-quality	50.21	0	unclassified	unclassified
v13ec	173906	296	41	9	Medium-quality	84.64	0	unclassified	unclassified
v13ed	57282	104	16	2	Complete	100	0	unclassified	unclassified
v13ee	91170	133	13	4	Complete	100	0	Quimbyviridae	prokaryote
v13ef	39887	58	19	1	High-quality	100	0	unclassified	unclassified
v13f0	35351	48	17	1	Complete	100	0	Siphoviridae	prokaryote
v13f1	195334	237	18	12	High-quality	98.5	0	unclassified	unclassified
v13f2	45860	75	15	0	Complete	100	0	unclassified	unclassified
v13f3	44039	80	13	3	High-quality	97.67	0	unclassified	unclassified
v13f4	93386	88	10	0	High-quality	100	0	Podoviridae_crAss-like	prokaryote
v13f5	36242	38	7	3	Medium-quality	75.62	0	Siphoviridae	prokaryote
v13f6	40629	58	40	1	High-quality	100	0	Siphoviridae	prokaryote
v13f7	50948	87	22	0	High-quality	97.73	0	unclassified	unclassified
v13f8	39909	57	41	0	High-quality	100	0	Siphoviridae	prokaryote
v13f9	59927	69	16	3	High-quality	100	0	unclassified	unclassified
v13fa	23304	36	23	4	Medium-quality	50.27	0	Siphoviridae	prokaryote
v13fb	48996	73	25	3	High-quality	100	0	unclassified	unclassified

v13fc	43087	67	19	1	High-quality	100	0	unclassified	unclassified
v13fd	28926	48	18	1	Medium-quality	73.02	0	Siphoviridae	prokaryote
v13fe	34254	46	26	0	High-quality	99.41	0	Myoviridae	prokaryote
v13ff	42599	75	39	0	High-quality	99.74	0	Myoviridae	prokaryote
v1400	19649	19	9	0	Medium-quality	51.96	0	Siphoviridae	prokaryote
v1401	34482	59	23	0	High-quality	100	0	Siphoviridae	prokaryote
v1402	8945	14	1	0	Complete	100	0	unclassified	unclassified
v1403	59651	57	17	2	Complete	100	0	unclassified	unclassified
v1404	71750	116	14	1	Complete	100	0	Quimbyviridae	prokaryote
v1405	36505	51	30	0	Medium-quality	79.54	0	Siphoviridae	prokaryote
v1406	51862	73	11	0	Medium-quality	65.5	0	Flandersviridae	prokaryote
v1407	18865	24	4	2	Medium-quality	65.76	0	Siphoviridae	prokaryote
v1408	19782	26	6	1	Medium-quality	50.21	0	Siphoviridae	prokaryote
v1409	30476	34	14	1	Medium-quality	62.31	0	unclassified	unclassified
v140a	130006	156	12	2	Medium-quality	84.52	0	unclassified	unclassified
v140b	101808	175	14	1	High-quality	100	0	Podoviridae_crAss-like	prokaryote
v140c	26749	27	9	2	Medium-quality	57.26	0	Siphoviridae	prokaryote
v140d	70479	98	12	1	Medium-quality	74.5	0	Quimbyviridae	prokaryote
v140e	39060	48	18	0	Complete	100	0	Siphoviridae	prokaryote
v140f	42055	45	19	0	Complete	100	0	unclassified	unclassified
v1410	52137	77	35	0	High-quality	100	0	Siphoviridae	prokaryote
v1411	34413	47	13	0	Complete	100	0	unclassified	unclassified
v1412	30370	45	13	0	Medium-quality	84.48	0	Siphoviridae	prokaryote
v1413	99637	162	27	1	Complete	100	0	Podoviridae_crAss-like	prokaryote
v1414	58599	97	11	2	Medium-quality	66.26	0	unclassified	unclassified
v1415	10372	17	6	0	Medium-quality	78.23	0	unclassified	unclassified
v1416	6784	7	1	0	High-quality	100	0	unclassified	unclassified
v1417	57968	96	12	2	Complete	100	0	unclassified	unclassified
v1418	33198	48	18	0	High-quality	91.29	0	unclassified	unclassified
v1419	23963	41	5	2	Medium-quality	56.89	0	Siphoviridae	prokaryote
v141a	37448	61	11	0	Complete	100	0	unclassified	unclassified
v141b	47668	62	12	1	High-quality	98.61	0	unclassified	unclassified
v141c	42292	65	19	1	Complete	100	0	Siphoviridae	prokaryote
v141d	66953	84	14	1	Complete	100	0	unclassified	unclassified
v141e	104554	155	13	2	Complete	100	0	Podoviridae_crAss-like	prokaryote
v141f	50097	80	25	0	Complete	100	0	unclassified	unclassified
v1420	98498	172	15	2	High-quality	100	0	Podoviridae_crAss-like	prokaryote
v1421	55447	81	9	7	Medium-quality	66.07	0	unclassified	unclassified
v1422	155385	217	17	5	Complete	100	0	unclassified	unclassified
v1423	35345	60	21	1	High-quality	100	0	Siphoviridae	prokaryote

v1424	30894	39	23	0	Medium-quality	73.07	0	Siphoviridae	prokaryote
v1425	69476	105	18	3	Medium-quality	86.44	0	unclassified	unclassified
v1426	49359	85	16	3	Medium-quality	71.58	0	unclassified	unclassified
v1427	59337	67	14	1	Medium-quality	70.16	0	Quimbyviridae	prokaryote
v1428	16105	22	10	0	Medium-quality	69.83	0	unclassified	unclassified
v1429	41964	61	9	6	High-quality	100	0	unclassified	unclassified
v142a	39993	58	8	0	High-quality	100	0	unclassified	unclassified
v142b	14563	19	7	0	High-quality	92.04	0	unclassified	unclassified
v142c	20660	27	24	0	Medium-quality	60.97	0	Siphoviridae	prokaryote
v142d	50253	49	7	1	High-quality	100	0	unclassified	unclassified
v142e	43760	64	17	1	Complete	100	0	Myoviridae	prokaryote
v142f	79367	112	19	0	Complete	100	0	Flandersviridae	prokaryote
v1430	20367	19	10	0	Medium-quality	51.29	0	unclassified	unclassified
v1431	82937	83	27	8	High-quality	100	0	Siphoviridae	prokaryote
v1432	40513	68	18	0	Medium-quality	62.73	0	unclassified	unclassified
v1433	32614	53	23	0	Medium-quality	80.71	0	Myoviridae	prokaryote
v1434	6223	10	2	0	Complete	100	0	unclassified	unclassified
v1435	36429	46	9	1	Complete	100	0	Siphoviridae	prokaryote
v1436	47199	67	16	0	Complete	100	0	Siphoviridae	prokaryote
v1437	52664	81	13	4	Medium-quality	85.88	0	unclassified	unclassified
v1438	157493	198	18	2	Complete	100	0	unclassified	unclassified
v1439	40663	58	27	1	High-quality	90.09	0	Siphoviridae	prokaryote
v143a	53542	67	8	3	Medium-quality	84.15	0	unclassified	unclassified
v143b	73908	122	18	1	High-quality	100	0	Quimbyviridae	prokaryote
v143c	108158	132	42	2	High-quality	99.5	0	Siphoviridae	prokaryote
v143d	18931	27	8	0	Medium-quality	54.68	0	unclassified	unclassified
v143e	26042	35	15	0	Medium-quality	66.91	0	Siphoviridae	prokaryote
v143f	66028	87	3	0	Medium-quality	60.61	0	unclassified	unclassified
v1440	28693	49	28	1	Medium-quality	71.21	0	Siphoviridae	prokaryote
v1441	27811	45	10	1	Medium-quality	61.95	0	unclassified	unclassified
v1442	38811	59	23	0	Medium-quality	86.94	0	Myoviridae	prokaryote
v1443	31323	44	15	1	High-quality	92.59	0	Siphoviridae	prokaryote
v1444	29821	49	13	0	Complete	100	0	unclassified	unclassified
v1445	59081	78	15	6	High-quality	100	0	Myoviridae	prokaryote
v1446	72916	93	16	4	High-quality	100	0	unclassified	unclassified
v1447	92639	119	6	2	Medium-quality	88.9	0	unclassified	unclassified
v1448	33550	48	22	0	High-quality	92.59	0	Myoviridae	prokaryote
v1449	67641	95	10	3	High-quality	95.59	0	Quimbyviridae	prokaryote
v144a	30375	44	15	0	Medium-quality	87.25	0	Myoviridae	prokaryote
v144b	101361	119	16	1	High-quality	100	0	Podoviridae_crAss-like	prokaryote

v144c	33894	55	28	0	Medium-quality	89.35	0	Siphoviridae	prokaryote
v144d	77232	100	24	7	High-quality	100	0	Myoviridae	prokaryote
v144e	39047	50	6	3	Medium-quality	57.05	0	unclassified	unclassified
v144f	27606	52	48	0	Medium-quality	89.59	0	Siphoviridae	prokaryote
v1450	19017	35	4	2	Medium-quality	51.47	0	unclassified	unclassified
v1451	28959	54	17	1	Medium-quality	85.04	0	Siphoviridae	prokaryote
v1452	63763	63	8	4	High-quality	100	0	unclassified	unclassified
v1453	27641	42	32	0	Medium-quality	70.84	0	Podoviridae	prokaryote
v1454	62708	80	19	6	High-quality	100	0	unclassified	unclassified
v1455	112539	120	39	1	Complete	100	0	Siphoviridae	prokaryote
v1456	42542	64	21	3	High-quality	100	0	Myoviridae	prokaryote
v1457	58151	106	13	2	Complete	100	0	unclassified	unclassified
v1458	33743	39	2	0	Medium-quality	51.56	0	unclassified	unclassified
v1459	36572	56	12	1	Medium-quality	70.82	0	Myoviridae	prokaryote
v145a	29385	47	20	0	Medium-quality	88.85	0	unclassified	unclassified
v145b	34560	53	20	5	High-quality	100	0	Siphoviridae	prokaryote
v145c	41684	64	3	2	High-quality	100	0	unclassified	unclassified
v145d	46740	73	30	0	Medium-quality	77.85	0	Siphoviridae	prokaryote
v145e	78930	82	10	8	High-quality	100	0	unclassified	unclassified
v145f	39784	46	6	0	Complete	100	0	unclassified	unclassified
v1460	41591	77	19	0	High-quality	100	0	unclassified	unclassified
v1461	38107	50	7	1	Medium-quality	63.58	0	unclassified	unclassified
v1462	24814	35	14	0	Medium-quality	60.21	0	unclassified	unclassified
v1463	39603	59	11	2	Medium-quality	67.11	0	unclassified	unclassified
v1464	41440	71	15	0	Complete	100	0	unclassified	unclassified
v1465	41783	43	9	4	High-quality	90.72	0	Siphoviridae	prokaryote
v1466	49231	77	36	0	Complete	100	0	Zobellviridae	prokaryote
v1467	42569	45	6	3	Medium-quality	71.06	0	unclassified	unclassified
v1468	77738	108	27	2	High-quality	100	0	Siphoviridae	prokaryote
v1469	34018	55	10	1	High-quality	95.21	0	unclassified	unclassified
v146a	34437	45	33	0	Medium-quality	88.84	0	Siphoviridae	prokaryote
v146b	37216	43	8	1	Medium-quality	65.06	0	Quimbyviridae	prokaryote
v146c	35996	63	21	0	Complete	100	0	Siphoviridae	prokaryote
v146d	42944	49	24	0	Medium-quality	71.6	0	Siphoviridae	prokaryote
v146e	14482	19	6	0	High-quality	91.52	0	unclassified	unclassified
v146f	17321	21	20	0	Medium-quality	51.41	0	Siphoviridae	prokaryote
v1470	22960	16	10	0	Medium-quality	55.62	0	Podoviridae	prokaryote
v1471	21576	25	20	0	Medium-quality	57.13	0	Siphoviridae	prokaryote
v1472	27801	48	18	0	Medium-quality	64.39	0	Siphoviridae	prokaryote
v1473	44914	55	12	5	High-quality	98.28	0	Siphoviridae	prokaryote

v1474	168270	231	38	10	High-quality	100	0	unclassified	unclassified
v1475	69146	106	12	1	High-quality	99.82	0	Quimbyviridae	prokaryote
v1476	26605	37	12	0	Medium-quality	69.77	0	Siphoviridae	prokaryote
v1477	35372	76	13	1	Medium-quality	81.11	0	unclassified	unclassified
v1478	25219	39	17	0	Medium-quality	63.07	0	Siphoviridae	prokaryote
v1479	53590	56	30	0	Complete	100	0	Siphoviridae	prokaryote
v147a	32206	49	35	0	Medium-quality	76.19	0	Myoviridae	prokaryote
v147b	48651	92	26	2	Complete	100	0	unclassified	unclassified
v147c	21669	24	15	1	Medium-quality	53.73	0	Siphoviridae	prokaryote
v147d	140668	179	17	4	High-quality	91.43	0	unclassified	unclassified
v147e	19612	26	8	2	Medium-quality	50.74	0	Siphoviridae	prokaryote
v147f	57281	103	25	4	Complete	100	0	Siphoviridae	prokaryote
v1480	54743	66	27	1	High-quality	100	0	Siphoviridae	prokaryote
v1481	42205	55	29	1	High-quality	100	0	Siphoviridae	prokaryote
v1482	56556	102	18	1	Complete	100	0	unclassified	unclassified
v1483	43503	70	20	0	Complete	100	0	unclassified	unclassified
v1484	42789	57	16	0	High-quality	96.56	0	unclassified	unclassified
v1485	37550	67	15	0	High-quality	100	0	unclassified	unclassified
v1486	32291	52	10	1	High-quality	97.25	0	unclassified	unclassified
v1487	36625	64	17	0	Medium-quality	69.57	0	unclassified	unclassified
v1488	32292	43	7	1	Medium-quality	55.67	0	unclassified	unclassified
v1489	57467	106	13	1	Complete	100	0	unclassified	unclassified
v148a	238832	225	34	5	High-quality	100	0	unclassified	unclassified
v148b	24131	31	3	2	Medium-quality	51.26	0	unclassified	unclassified
v148c	6906	6	2	0	High-quality	100	0	unclassified	unclassified
v148d	31119	32	23	1	Medium-quality	53.96	0	Siphoviridae	prokaryote
v148e	6473	8	1	0	High-quality	100	0	unclassified	unclassified
v148f	71825	122	17	3	High-quality	94.25	0	unclassified	unclassified
v1490	36913	51	23	1	High-quality	99.56	0	Siphoviridae	prokaryote
v1491	34916	57	19	2	High-quality	100	0	unclassified	unclassified
v1492	45745	63	18	3	High-quality	100	0	Siphoviridae	prokaryote
v1493	38360	63	18	0	High-quality	100	0	unclassified	unclassified
v1494	46044	67	41	0	Complete	100	0	Siphoviridae	prokaryote
v1495	70357	94	10	4	Medium-quality	79.8	0	Quimbyviridae	prokaryote
v1496	13581	17	7	0	Medium-quality	74.12	0	Salasmaviridae	prokaryote
v1497	84949	95	18	10	High-quality	100	0	unclassified	unclassified
v1498	38467	65	18	0	Complete	100	0	unclassified	unclassified
v1499	56817	101	22	4	Complete	100	0	Siphoviridae	prokaryote
v149a	29541	34	5	2	Medium-quality	64.27	0	unclassified	unclassified
v149b	31894	34	4	3	High-quality	94.75	0	unclassified	unclassified

v149c	76422	97	16	1	High-quality	100	0	unclassified	unclassified
v149d	41595	53	30	0	Medium-quality	69.29	0	Siphoviridae	prokaryote
v149e	38930	65	26	0	High-quality	95.97	0	Myoviridae	prokaryote
v149f	62884	94	16	0	High-quality	90.76	0	unclassified	unclassified
v14a0	181334	300	22	2	Complete	100	0	unclassified	unclassified
v14a1	188113	226	33	4	Complete	100	0	unclassified	unclassified
v14a2	14844	17	4	3	Medium-quality	51.74	0	unclassified	unclassified
v14a3	57097	91	25	1	High-quality	100	0	unclassified	unclassified
v14a4	5899	8	3	0	Complete	100	0	unclassified	unclassified
v14a5	57762	78	52	0	Complete	100	0	Siphoviridae	prokaryote
v14a6	82187	114	28	0	High-quality	100	0	Myoviridae	prokaryote
v14a7	43407	61	16	2	High-quality	100	0	unclassified	unclassified
v14a8	41929	58	37	0	High-quality	100	0	Siphoviridae	prokaryote
v14a9	13072	16	5	0	High-quality	96.19	0	unclassified	unclassified
v14aa	99715	124	21	3	Complete	100	0	Gratiaviridae	prokaryote
v14ab	33494	41	10	1	Medium-quality	61.16	0	Siphoviridae	prokaryote
v14ac	45387	76	22	3	High-quality	99.26	0	unclassified	unclassified
v14ad	56728	105	16	1	Complete	100	0	unclassified	unclassified
v14ae	78447	123	18	1	Complete	100	0	Quimbyviridae	prokaryote
v14af	33011	51	17	1	High-quality	99.64	0	Siphoviridae	prokaryote
v14b0	103977	192	32	1	Complete	100	0	Podoviridae_crAss-like	prokaryote
v14b1	41071	73	22	1	Complete	100	0	Siphoviridae	prokaryote
v14b2	63355	97	19	1	Medium-quality	89.3	0	unclassified	unclassified
v14b3	38020	48	33	1	Medium-quality	84.64	0	Siphoviridae	prokaryote
v14b4	138973	181	41	2	High-quality	99.48	0	unclassified	unclassified
v14b5	88001	106	4	1	Complete	100	0	unclassified	unclassified
v14b6	14981	20	9	0	Medium-quality	75.15	0	Salasmaviridae	prokaryote
v14b7	35211	32	23	1	Medium-quality	58.09	0	Siphoviridae	prokaryote
v14b8	57674	99	14	2	Complete	100	0	unclassified	unclassified
v14b9	72844	95	25	0	Medium-quality	53.22	0	unclassified	unclassified
v14ba	104179	174	34	3	High-quality	99.73	0	Myoviridae	prokaryote
v14bb	14245	18	6	0	High-quality	100	0	unclassified	unclassified
v14bc	58548	80	19	3	High-quality	100	0	Myoviridae	prokaryote
v14bd	29728	37	9	2	Medium-quality	61.53	0	Siphoviridae	prokaryote
v14be	94062	145	19	4	High-quality	99.43	0	Quimbyviridae	prokaryote
v14bf	43494	56	39	0	High-quality	100	0	Siphoviridae	prokaryote
v14c0	88335	134	17	3	Complete	100	0	Quimbyviridae	prokaryote
v14c1	31710	43	13	0	High-quality	92.89	0	unclassified	unclassified
v14c2	52210	81	20	2	High-quality	100	0	Myoviridae	prokaryote
v14c3	43146	60	22	1	Complete	100	0	Siphoviridae	prokaryote

v14c4	43079	78	15	1	Complete	100	0	unclassified	unclassified
v14c5	41753	57	19	0	High-quality	100	0	unclassified	unclassified
v14c6	36808	41	9	4	Medium-quality	78.71	0	unclassified	unclassified
v14c7	42278	57	38	2	High-quality	100	0	Podoviridae	prokaryote
v14c8	226536	241	124	0	Complete	100	0	Myoviridae	prokaryote
v14c9	31927	49	27	0	Complete	100	0	Siphoviridae	prokaryote
v14ca	26164	43	10	1	Medium-quality	58.05	0	unclassified	unclassified
v14cb	89386	136	20	4	High-quality	100	0	unclassified	unclassified
v14cc	6159	10	2	0	Complete	100	0	unclassified	unclassified
v14cd	43439	75	13	2	Medium-quality	71.36	0	Myoviridae	prokaryote
v14ce	67690	99	25	2	Complete	100	0	Siphoviridae	prokaryote
v14cf	35739	54	28	0	High-quality	100	0	Myoviridae	prokaryote
v14d0	96588	97	13	0	High-quality	100	0	Podoviridae_crAss-like	prokaryote
v14d1	66801	76	4	3	High-quality	100	0	unclassified	unclassified
v14d2	40013	52	49	0	Complete	100	0	Autographiviridae	prokaryote
v14d3	34223	56	16	1	Medium-quality	57.51	0	unclassified	unclassified
v14d4	43965	62	9	1	Complete	100	0	unclassified	unclassified
v14d5	30427	56	13	0	Medium-quality	67.49	0	unclassified	unclassified
v14d6	33269	57	16	0	Complete	100	0	unclassified	unclassified
v14d7	106363	125	9	2	Complete	100	0	unclassified	unclassified
v14d8	44914	69	20	0	Complete	100	0	unclassified	unclassified
v14d9	34915	44	28	0	Medium-quality	75.16	0	Siphoviridae	prokaryote
v14da	44203	85	16	0	High-quality	100	0	unclassified	unclassified
v14db	42032	62	20	1	Complete	100	0	unclassified	unclassified
v14dc	23784	32	10	0	Medium-quality	61.54	0	unclassified	unclassified
v14dd	44011	73	19	0	Complete	100	0	unclassified	unclassified
v14de	47013	65	9	2	Medium-quality	60.77	0	unclassified	unclassified
v14df	39554	43	10	1	Medium-quality	52.46	0	unclassified	unclassified
v14e0	60815	80	11	2	High-quality	100	0	unclassified	unclassified
v14e1	45544	52	5	4	High-quality	100	0	unclassified	unclassified
v14e2	50938	42	6	5	High-quality	96.55	0	unclassified	unclassified
v14e3	97126	99	12	2	High-quality	95.27	0	Podoviridae_crAss-like	prokaryote
v14e4	31799	40	17	0	High-quality	98.03	0	Siphoviridae	prokaryote
v14e5	18768	26	7	1	Medium-quality	58.42	0	unclassified	unclassified
v14e6	87091	106	12	2	High-quality	91.07	0	unclassified	unclassified
v14e7	148449	187	27	8	Complete	100	0	unclassified	unclassified
v14e8	55352	79	16	3	High-quality	90.09	0	unclassified	unclassified
v14e9	32365	39	26	0	Medium-quality	65.6	0	Siphoviridae	prokaryote
v14ea	15987	19	8	0	Medium-quality	75.75	0	Salasmaviridae	prokaryote
v14eb	44215	72	29	0	Complete	100	0	unclassified	unclassified

v14ec	60184	108	16	1	Complete	100	0	unclassified	unclassified
v14ed	96389	103	20	0	Complete	100	0	Podoviridae_crAss-like	prokaryote
v14ee	40860	65	19	0	High-quality	100	0	Siphoviridae	prokaryote
v14ef	70965	76	10	3	Complete	100	0	unclassified	unclassified
v14f0	36466	57	21	0	Medium-quality	87.61	0	Myoviridae	prokaryote
v14f1	46351	72	15	1	Complete	100	0	unclassified	unclassified
v14f2	77696	116	18	3	Complete	100	0	unclassified	unclassified
v14f3	20951	23	12	2	Medium-quality	51.97	0	unclassified	unclassified
v14f4	29366	46	15	0	Complete	100	0	Siphoviridae	prokaryote
v14f5	16890	25	9	0	Medium-quality	73.21	0	unclassified	unclassified
v14f6	29238	58	5	0	Medium-quality	74.88	0	unclassified	unclassified
v14f7	25894	36	16	1	Medium-quality	63.42	0	Siphoviridae	prokaryote
v14f8	39856	65	20	0	High-quality	97.52	0	unclassified	unclassified
v14f9	13369	18	5	0	Medium-quality	74.42	0	Podoviridae	prokaryote
v14fa	23392	29	6	0	Medium-quality	52.13	0	unclassified	unclassified
v14fb	52017	66	9	3	High-quality	100	0	unclassified	unclassified
v14fc	45005	64	31	1	High-quality	91.1	0	Myoviridae	prokaryote
v14fd	42339	67	22	2	Complete	100	0	Siphoviridae	prokaryote
v14fe	21799	27	2	1	Medium-quality	51.24	0	unclassified	unclassified
v14ff	22109	30	8	0	Medium-quality	55.76	0	unclassified	unclassified
v1500	56494	100	15	2	Complete	100	0	unclassified	unclassified
v1501	61610	101	21	1	High-quality	100	0	unclassified	unclassified
v1502	57660	105	19	1	High-quality	94.15	0	unclassified	unclassified
v1503	41961	55	24	2	High-quality	100	0	Siphoviridae	prokaryote
v1504	58303	98	9	1	Complete	100	0	unclassified	unclassified
v1505	56398	66	6	5	High-quality	100	0	unclassified	unclassified
v1506	34974	60	34	0	Medium-quality	82.08	0	Myoviridae	prokaryote
v1507	94991	132	34	1	High-quality	100	0	unclassified	unclassified
v1508	31542	47	12	1	High-quality	90.54	0	Siphoviridae	prokaryote
v1509	69305	88	7	2	Medium-quality	79.84	0	Quimbyviridae	prokaryote
v150a	39963	47	18	0	High-quality	96.22	0	Siphoviridae	prokaryote
v150b	43714	52	18	1	Medium-quality	64.64	0	unclassified	unclassified
v150c	37435	56	20	0	High-quality	100	0	Siphoviridae	prokaryote
v150d	39068	69	25	0	Complete	100	0	Siphoviridae	prokaryote
v150e	34056	32	7	2	Medium-quality	51.69	0	unclassified	unclassified
v150f	44479	58	20	1	High-quality	100	0	Siphoviridae	prokaryote
v1510	37999	65	29	0	Medium-quality	58.21	0	Myoviridae	prokaryote
v1511	47553	58	21	1	Complete	100	0	Siphoviridae	prokaryote
v1512	41560	72	21	0	High-quality	99.31	0	Siphoviridae	prokaryote
v1513	45857	77	17	1	Complete	100	0	unclassified	unclassified

v1514	58384	98	18	2	Complete	100	0	unclassified	unclassified
v1515	65283	90	22	2	Complete	100	0	unclassified	unclassified
v1516	49630	54	9	0	High-quality	100	0	Siphoviridae	prokaryote
v1517	50123	65	20	1	High-quality	100	0	Siphoviridae	prokaryote
v1518	32005	39	9	2	Medium-quality	81.56	0	unclassified	unclassified
v1519	70737	79	5	2	Medium-quality	88.81	0	unclassified	unclassified
v151a	28769	32	7	1	Medium-quality	57.76	0	Siphoviridae	prokaryote
v151b	105184	177	20	2	High-quality	100	0	Podoviridae_crAss-like	prokaryote
v151c	57013	100	20	0	Complete	100	0	unclassified	unclassified
v151d	17594	21	5	4	Medium-quality	61.33	0	unclassified	unclassified
v151e	61679	64	8	5	Medium-quality	69.8	0	unclassified	unclassified
v151f	31797	40	20	1	Medium-quality	84.67	0	Siphoviridae	prokaryote
v1520	83110	111	14	0	High-quality	97.34	0	Flandersviridae	prokaryote
v1521	33780	44	8	0	Medium-quality	80.69	0	unclassified	unclassified
v1522	76823	114	29	4	Complete	100	0	Siphoviridae	prokaryote
v1523	46069	76	17	0	High-quality	100	0	unclassified	unclassified
v1524	40529	76	17	0	Complete	100	0	unclassified	unclassified
v1525	45580	58	40	0	Complete	100	0	Autographiviridae	prokaryote
v1526	24775	31	17	0	Medium-quality	65.04	0	Siphoviridae	prokaryote
v1527	22996	17	2	0	Medium-quality	66.95	0	unclassified	unclassified
v1528	16254	20	3	0	Medium-quality	89.99	0	Retroviridae	eukaryote
v1529	12049	18	1	0	Medium-quality	63.57	0	unclassified	unclassified
v152a	13297	19	2	0	High-quality	100	0	unclassified	unclassified
v152b	12260	11	2	0	High-quality	93.8	0	unclassified	unclassified
v152c	11548	11	1	0	Medium-quality	88.76	0	Retroviridae	eukaryote
v152d	14708	9	4	0	High-quality	91.2	0	Retroviridae	eukaryote
v152e	12736	16	1	0	High-quality	100	0	unclassified	unclassified
v152f	20198	21	2	0	High-quality	100	0	unclassified	unclassified
v1530	16002	15	1	0	High-quality	100	0	unclassified	unclassified
v1531	14126	17	3	0	High-quality	100	0	Retroviridae	eukaryote
v1532	10340	8	3	0	High-quality	97.74	0	Retroviridae	eukaryote
v1533	7067	5	1	0	Medium-quality	69.78	0	Retroviridae	eukaryote
v1534	31416	27	3	0	High-quality	100	0	unclassified	unclassified
v1535	33805	19	2	0	High-quality	100	0	unclassified	unclassified
v1536	23474	22	3	0	High-quality	100	0	unclassified	unclassified
v1537	14686	15	3	0	High-quality	100	0	Retroviridae	eukaryote
v1538	13042	10	3	0	Medium-quality	87.75	0	Retroviridae	eukaryote
v1539	15921	12	2	0	High-quality	100	0	Retroviridae	eukaryote
v153a	8241	11	2	0	Medium-quality	53.4	0	unclassified	unclassified
v153b	20689	20	2	0	High-quality	100	0	unclassified	unclassified

v153c	5993	6	1	0	Medium-quality	70.3	0	Retroviridae	eukaryote
v153d	14141	22	3	0	High-quality	100	0	Retroviridae	eukaryote
v153e	8183	11	1	0	High-quality	100	0	unclassified	unclassified
v153f	7846	11	3	0	High-quality	94.52	0	Retroviridae	eukaryote
v1540	7011	10	1	0	High-quality	100	0	unclassified	unclassified
v1541	24871	13	1	0	High-quality	100	0	Retroviridae	eukaryote
v1542	5683	10	1	0	High-quality	91.65	0	unclassified	unclassified
v1543	10019	14	2	0	High-quality	100	0	Retroviridae	eukaryote
v1544	35254	29	1	0	High-quality	100	0	unclassified	unclassified
v1545	7850	10	1	0	Medium-quality	68.77	0	Retroviridae	eukaryote
v1546	10774	13	1	0	High-quality	100	0	unclassified	unclassified
v1547	10357	14	4	0	High-quality	99.93	0	Retroviridae	eukaryote
v1548	23323	29	2	0	High-quality	100	0	unclassified	unclassified
v1549	10728	10	1	0	Medium-quality	56.6	0	unclassified	unclassified
v154a	7969	11	1	0	High-quality	100	0	unclassified	unclassified
v154b	9092	7	1	0	Medium-quality	79.65	0	unclassified	unclassified
v154c	38808	26	2	0	High-quality	100	0	unclassified	unclassified
v154d	16503	18	3	0	High-quality	99.4	0	unclassified	unclassified
v154e	18019	11	1	0	High-quality	100	0	unclassified	unclassified
v154f	15529	15	2	0	High-quality	100	0	unclassified	unclassified
v1550	10119	4	1	0	High-quality	100	0	Retroviridae	eukaryote
v1551	13333	14	2	0	Medium-quality	86.71	0	unclassified	unclassified
v1552	26548	22	2	0	High-quality	96.91	0	unclassified	unclassified
v1553	11364	9	2	0	High-quality	100	0	unclassified	unclassified
v1554	17354	10	2	0	High-quality	100	0	unclassified	unclassified
v1555	21695	21	3	0	High-quality	100	0	unclassified	unclassified
v1556	13290	12	1	0	High-quality	100	0	Retroviridae	eukaryote
v1557	11006	12	3	0	High-quality	100	0	Retroviridae	eukaryote
v1558	6261	3	1	0	Medium-quality	75.48	0	Retroviridae	eukaryote
v1559	11963	19	3	0	High-quality	100	0	Retroviridae	eukaryote
v155a	13841	11	1	0	High-quality	100	0	unclassified	unclassified
v155b	6058	8	1	0	High-quality	97.69	0	unclassified	unclassified
v155c	11973	15	5	0	High-quality	97.43	0	Retroviridae	eukaryote
v155d	7945	14	2	0	Medium-quality	69.6	0	Retroviridae	eukaryote
v155e	19023	17	1	0	High-quality	100	0	unclassified	unclassified
v155f	8522	8	1	0	High-quality	100	0	unclassified	unclassified
v1560	5668	2	1	0	Medium-quality	66.49	0	Retroviridae	eukaryote
v1561	13109	13	1	0	High-quality	100	0	unclassified	unclassified
v1562	10083	6	2	0	High-quality	98.77	0	Retroviridae	eukaryote
v1563	34885	37	2	0	High-quality	100	0	unclassified	unclassified

v1564	18532	11	1	0	High-quality	100	0	unclassified	unclassified
v1565	14514	7	4	0	High-quality	100	0	Retroviridae	eukaryote
v1566	15035	12	2	0	High-quality	100	0	unclassified	unclassified
v1567	7676	12	1	0	High-quality	90.04	0	unclassified	unclassified
v1568	10736	8	1	0	High-quality	100	0	unclassified	unclassified
v1569	16253	14	2	0	Medium-quality	89.99	0	unclassified	unclassified
v156a	12709	18	1	0	High-quality	100	0	unclassified	unclassified
v156b	12550	13	2	0	High-quality	100	0	unclassified	unclassified
v156c	18026	23	3	0	High-quality	95.1	0	unclassified	unclassified
v156d	17046	18	4	0	Medium-quality	86.89	0	Retroviridae	eukaryote
v156e	16945	10	3	0	High-quality	100	0	Retroviridae	eukaryote
v156f	13249	12	2	0	High-quality	100	0	unclassified	unclassified
v1570	8985	12	4	0	Medium-quality	58.22	0	Retroviridae	eukaryote
v1571	16519	23	1	0	High-quality	100	0	unclassified	unclassified
v1572	21990	25	2	0	High-quality	100	0	unclassified	unclassified
v1573	7297	8	1	0	Medium-quality	68.03	0	Retroviridae	eukaryote
v1574	12711	17	2	0	High-quality	100	0	Retroviridae	eukaryote
v1575	10977	12	1	0	High-quality	100	0	unclassified	unclassified
v1576	8479	7	1	0	Medium-quality	79.05	0	unclassified	unclassified
v1577	11010	12	2	0	High-quality	97.94	0	Retroviridae	eukaryote
v1578	9528	13	4	0	Medium-quality	83.47	0	unclassified	unclassified
v1579	11721	11	3	0	High-quality	98.12	0	Retroviridae	eukaryote
v157a	18606	18	2	0	High-quality	99.91	0	Retroviridae	eukaryote
v157b	29964	32	3	0	High-quality	100	0	Retroviridae	eukaryote
v157c	11167	19	6	0	High-quality	97.06	0	Retroviridae	eukaryote
v157d	17179	11	2	0	High-quality	100	0	unclassified	unclassified
v157e	9459	12	1	0	Medium-quality	72.37	0	unclassified	unclassified
v157f	17257	25	6	0	Medium-quality	88.65	0	Podoviridae	prokaryote
v1580	18766	37	5	1	Medium-quality	50.26	0	Siphoviridae	prokaryote
v1581	11220	9	1	0	High-quality	100	0	unclassified	unclassified
v1582	7181	11	3	0	Medium-quality	83.87	0	Retroviridae	eukaryote
v1583	5941	6	1	0	Medium-quality	55.39	0	Retroviridae	eukaryote
v1584	7226	4	2	0	Medium-quality	67.37	0	Retroviridae	eukaryote
v1585	9858	10	4	0	High-quality	100	0	Retroviridae	eukaryote
v1586	6679	7	1	0	High-quality	100	0	unclassified	unclassified
v1587	6663	7	1	0	Medium-quality	78.16	0	Retroviridae	eukaryote
v1588	18952	25	4	0	High-quality	100	0	unclassified	unclassified
v1589	6439	4	1	0	High-quality	100	0	Retroviridae	eukaryote
v158a	12321	13	1	0	High-quality	100	0	unclassified	unclassified
v158b	12318	13	3	0	Medium-quality	80.69	0	unclassified	unclassified

v158c	11940	11	1	0	Medium-quality	62.99	0	unclassified	unclassified
v158d	9988	11	1	0	High-quality	93.12	0	unclassified	unclassified
v158e	5074	7	1	0	Medium-quality	81.83	0	unclassified	unclassified
v158f	23109	13	1	0	High-quality	100	0	Retroviridae	eukaryote
v1590	9362	7	1	0	Medium-quality	71.96	0	Retroviridae	eukaryote
v1591	7906	13	2	0	High-quality	100	0	unclassified	unclassified
v1592	11457	8	2	0	Medium-quality	72.21	0	Retroviridae	eukaryote
v1593	13527	18	3	0	High-quality	100	0	Retroviridae	eukaryote
v1594	11299	12	1	0	High-quality	100	0	unclassified	unclassified
v1595	42772	41	4	0	Medium-quality	88.07	0	unclassified	unclassified
v1596	11099	8	2	0	High-quality	100	0	Retroviridae	eukaryote
v1597	5233	10	1	0	Medium-quality	84.39	0	unclassified	unclassified
v1598	7015	6	1	0	High-quality	100	0	unclassified	unclassified
v1599	7538	12	1	0	Medium-quality	88.43	0	unclassified	unclassified
v159a	22520	12	2	0	High-quality	100	0	unclassified	unclassified
v159b	16628	14	1	0	Medium-quality	87.73	0	Retroviridae	eukaryote
v159c	9767	9	3	0	High-quality	97.62	0	Retroviridae	eukaryote
v159d	14650	11	2	0	Medium-quality	77.29	0	unclassified	unclassified
v159e	8047	6	1	0	High-quality	94.4	0	unclassified	unclassified
v159f	14160	14	3	0	High-quality	100	0	unclassified	unclassified
v15a0	20297	13	2	0	High-quality	100	0	unclassified	unclassified
v15a1	10115	13	1	0	High-quality	94.3	0	unclassified	unclassified
v15a2	13575	14	4	0	High-quality	100	0	Retroviridae	eukaryote
v15a3	7345	15	1	0	Medium-quality	86.16	0	Retroviridae	eukaryote
v15a4	6242	7	1	0	High-quality	100	0	unclassified	unclassified
v15a5	10864	11	2	0	High-quality	100	0	Retroviridae	eukaryote
v15a6	11136	7	2	0	High-quality	97.55	0	unclassified	unclassified
v15a7	13888	13	2	0	Medium-quality	81.22	0	Retroviridae	eukaryote
v15a8	30835	21	1	0	High-quality	100	0	unclassified	unclassified
v15a9	15343	7	1	0	High-quality	100	0	unclassified	unclassified
v15aa	13705	9	2	0	High-quality	100	0	unclassified	unclassified
v15ab	10586	7	1	0	High-quality	98.69	0	Retroviridae	eukaryote
v15ac	23393	19	3	0	High-quality	100	0	Retroviridae	eukaryote
v15ad	20719	15	2	0	High-quality	100	0	Retroviridae	eukaryote
v15ae	50603	53	3	0	High-quality	100	0	unclassified	unclassified
v15af	18457	8	1	0	High-quality	100	0	unclassified	unclassified
v15b0	10902	15	1	0	Medium-quality	57.52	0	unclassified	unclassified
v15b1	16520	12	1	0	Medium-quality	87.16	0	unclassified	unclassified
v15b2	15948	12	1	0	Medium-quality	84.14	0	unclassified	unclassified
v15b3	22765	23	7	0	High-quality	97.77	0	Retroviridae	eukaryote

v15b4	7576	11	2	0	Medium-quality	88.48	0	Retroviridae	eukaryote
v15b5	20861	15	3	0	High-quality	100	0	unclassified	unclassified
v15b6	13559	13	2	0	High-quality	100	0	unclassified	unclassified
v15b7	54549	30	4	0	High-quality	100	0	unclassified	unclassified
v15b8	7513	11	2	0	Medium-quality	85.75	0	Retroviridae	eukaryote
v15b9	15674	11	1	0	High-quality	100	0	unclassified	unclassified
v15ba	16382	20	4	0	High-quality	100	0	Retroviridae	eukaryote
v15bb	14309	9	1	0	Medium-quality	75.49	0	unclassified	unclassified
v15bc	11019	8	1	0	Medium-quality	58.13	0	unclassified	unclassified
v15bd	43139	33	2	0	High-quality	100	0	unclassified	unclassified
v15be	16228	13	2	0	Medium-quality	89.9	0	unclassified	unclassified
v15bf	17426	14	1	0	High-quality	100	0	unclassified	unclassified
v15c0	5736	3	1	0	Medium-quality	67.29	0	Retroviridae	eukaryote
v15c1	10506	11	1	0	High-quality	100	0	unclassified	unclassified
v15c2	11481	10	2	0	High-quality	100	0	unclassified	unclassified
v15c3	17459	13	1	0	High-quality	92.11	0	unclassified	unclassified
v15c4	18559	18	2	0	High-quality	100	0	unclassified	unclassified
v15c5	6465	9	1	0	High-quality	100	0	unclassified	unclassified
v15c6	13598	14	1	0	High-quality	100	0	unclassified	unclassified
v15c7	16398	16	1	0	High-quality	100	0	unclassified	unclassified
v15c8	6012	10	1	0	High-quality	96.95	0	unclassified	unclassified
v15c9	16799	10	1	0	High-quality	100	0	unclassified	unclassified
v15ca	15944	10	3	0	High-quality	100	0	Retroviridae	eukaryote
v15cb	8903	12	1	0	High-quality	100	0	unclassified	unclassified
v15cc	11338	16	4	0	Medium-quality	78.55	0	unclassified	unclassified
v15cd	13598	11	2	0	Medium-quality	80.15	0	unclassified	unclassified
v15ce	13273	14	2	0	High-quality	100	0	Retroviridae	eukaryote
v15cf	10390	7	1	0	High-quality	96.87	0	Retroviridae	eukaryote
v15d0	8928	11	2	0	Medium-quality	83.24	0	Retroviridae	eukaryote
v15d1	7663	3	1	0	Medium-quality	89.89	0	unclassified	unclassified
v15d2	33682	26	1	0	High-quality	100	0	unclassified	unclassified
v15d3	9266	4	1	0	High-quality	100	0	unclassified	unclassified
v15d4	6647	9	4	0	Medium-quality	78.69	0	Retroviridae	eukaryote
v15d5	16208	11	1	0	High-quality	100	0	Retroviridae	eukaryote
v15d6	22296	15	1	0	High-quality	100	0	unclassified	unclassified
v15d7	8286	8	2	0	High-quality	97.2	0	Retroviridae	eukaryote
v15d8	15709	9	1	0	High-quality	100	0	unclassified	unclassified
v15d9	10042	12	2	0	High-quality	100	0	unclassified	unclassified
v15da	19855	10	1	0	High-quality	100	0	unclassified	unclassified
v15db	8048	4	1	0	Medium-quality	75.03	0	unclassified	unclassified

v15dc	5889	9	1	0	Medium-quality	54.9	0	unclassified	unclassified
v15dd	9997	13	4	0	Medium-quality	87.58	0	Retroviridae	eukaryote
v15de	8241	10	3	0	High-quality	96.25	0	Retroviridae	eukaryote
v15df	11899	15	3	0	High-quality	100	0	Retroviridae	eukaryote
v15e0	7585	5	1	0	Medium-quality	74.32	0	unclassified	unclassified
v15e1	7621	4	1	0	Medium-quality	71.05	0	Retroviridae	eukaryote
v15e2	16029	18	2	0	Medium-quality	89.16	0	Retroviridae	eukaryote
v15e3	11743	9	1	0	Medium-quality	61.95	0	unclassified	unclassified
v15e4	26945	21	1	0	High-quality	100	0	unclassified	unclassified
v15e5	14076	13	3	0	High-quality	98.76	0	Retroviridae	eukaryote
v15e6	9588	10	1	0	High-quality	100	0	unclassified	unclassified
v15e7	13286	11	3	0	High-quality	98.53	0	Retroviridae	eukaryote
v15e8	18226	23	2	0	High-quality	100	0	unclassified	unclassified
v15e9	10083	7	1	0	High-quality	94	0	unclassified	unclassified
v15ea	16698	13	6	0	High-quality	100	0	Retroviridae	eukaryote
v15eb	11489	11	1	0	High-quality	100	0	unclassified	unclassified
v15ec	15084	12	1	0	Medium-quality	79.58	0	Retroviridae	eukaryote
v15ed	9342	9	2	0	High-quality	100	0	unclassified	unclassified
v15ee	8522	6	1	0	Medium-quality	65.2	0	Retroviridae	eukaryote
v15ef	59272	69	10	8	High-quality	100	0	unclassified	unclassified
v15f0	37149	56	15	0	High-quality	95.36	0	unclassified	unclassified
v15f1	34421	53	21	0	Complete	100	0	Siphoviridae	prokaryote
v15f2	39834	54	16	0	Complete	100	0	unclassified	unclassified
v15f3	32870	45	34	0	Medium-quality	83.45	0	Siphoviridae	prokaryote
v15f4	23883	34	15	0	Medium-quality	62.09	0	Myoviridae	prokaryote
v15f5	119793	194	31	3	Medium-quality	74.35	0	unclassified	unclassified
v15f6	32194	52	21	0	Complete	100	0	Siphoviridae	prokaryote
v15f7	5755	4	1	0	Medium-quality	53.65	0	Retroviridae	eukaryote
v15f8	11802	9	2	0	Medium-quality	86.64	0	Retroviridae	eukaryote
v15f9	23478	30	21	0	Medium-quality	59.61	0	Siphoviridae	prokaryote
v15fa	24639	29	10	0	Medium-quality	55.52	0	Siphoviridae	prokaryote
v15fb	42393	71	16	0	High-quality	100	0	unclassified	unclassified
v15fc	42578	69	13	0	Complete	100	0	unclassified	unclassified
v15fd	9409	9	2	0	Medium-quality	76.54	0	Retroviridae	eukaryote
v15fe	21413	23	1	0	High-quality	100	0	unclassified	unclassified
v15ff	22132	22	4	0	High-quality	100	0	unclassified	unclassified
v1600	21541	14	2	0	High-quality	100	0	unclassified	unclassified
v1601	17029	17	5	0	High-quality	99.54	0	Retroviridae	eukaryote
v1602	7366	10	1	0	Medium-quality	72.18	0	unclassified	unclassified
v1603	34920	41	1	0	High-quality	100	0	unclassified	unclassified

v1604	10175	10	1	0	High-quality	94.86	0	unclassified	unclassified
v1605	7796	7	3	0	Medium-quality	54.66	0	Retroviridae	eukaryote
v1606	13619	12	4	0	High-quality	100	0	Retroviridae	eukaryote
v1607	15540	11	2	0	High-quality	91.51	0	Retroviridae	eukaryote
v1608	6268	7	1	0	Medium-quality	58.44	0	unclassified	unclassified
v1609	5264	5	1	0	Medium-quality	61.75	0	unclassified	unclassified
v160a	17535	15	2	0	High-quality	100	0	unclassified	unclassified
v160b	29757	23	2	0	High-quality	100	0	unclassified	unclassified
v160c	7940	11	4	0	Medium-quality	69.56	0	unclassified	unclassified
v160d	11704	10	1	0	High-quality	100	0	Retroviridae	eukaryote
v160e	12417	10	1	0	High-quality	100	0	unclassified	unclassified
v160f	11608	10	2	0	High-quality	98.1	0	unclassified	unclassified
v1610	13544	12	2	0	High-quality	100	0	unclassified	unclassified
v1611	23302	26	3	0	High-quality	100	0	unclassified	unclassified
v1612	16740	20	1	0	High-quality	100	0	unclassified	unclassified
v1613	31899	18	5	0	High-quality	100	0	unclassified	unclassified
v1614	16589	11	1	0	High-quality	100	0	unclassified	unclassified
v1615	10898	12	2	0	High-quality	100	0	Retroviridae	eukaryote
v1616	21828	15	1	0	High-quality	100	0	unclassified	unclassified
v1617	21020	18	1	0	High-quality	100	0	unclassified	unclassified
v1618	17246	13	1	0	High-quality	90.99	0	unclassified	unclassified
v1619	39283	42	1	0	High-quality	100	0	unclassified	unclassified
v161a	19762	14	2	0	High-quality	100	0	Retroviridae	eukaryote
v161b	32283	30	2	0	High-quality	100	0	unclassified	unclassified
v161c	15397	12	1	0	Medium-quality	81.23	0	unclassified	unclassified
v161d	16726	10	1	0	High-quality	100	0	unclassified	unclassified
v161e	8004	11	1	0	High-quality	100	0	unclassified	unclassified
v161f	16149	16	2	0	High-quality	100	0	Retroviridae	eukaryote
v1620	32601	36	4	0	High-quality	100	0	unclassified	unclassified
v1621	8176	9	1	0	Medium-quality	76.22	0	Retroviridae	eukaryote
v1622	16173	16	1	0	High-quality	100	0	Retroviridae	eukaryote
v1623	11515	12	2	0	High-quality	99.37	0	Retroviridae	eukaryote
v1624	16725	20	3	0	Medium-quality	88.24	0	unclassified	unclassified
v1625	15080	17	6	0	High-quality	100	0	unclassified	unclassified
v1626	34588	27	1	0	High-quality	100	0	unclassified	unclassified
v1627	11496	8	1	0	High-quality	100	0	Retroviridae	eukaryote
v1628	13447	11	2	0	Medium-quality	79.58	0	unclassified	unclassified
v1629	44981	45	2	0	High-quality	100	0	unclassified	unclassified
v162a	32867	21	1	0	High-quality	100	0	unclassified	unclassified
v162b	15124	16	3	0	High-quality	99.03	0	Retroviridae	eukaryote

v162c	7707	13	1	0	High-quality	100	0	unclassified	unclassified
v162d	13318	12	4	0	High-quality	98.64	0	Retroviridae	eukaryote
v162e	21580	27	3	0	High-quality	100	0	Retroviridae	eukaryote
v162f	34812	28	2	0	High-quality	100	0	unclassified	unclassified
v1630	20808	22	2	0	High-quality	100	0	unclassified	unclassified
v1631	7873	17	1	0	High-quality	100	0	unclassified	unclassified
v1632	16011	12	1	0	High-quality	100	0	unclassified	unclassified
v1633	13609	13	3	0	High-quality	96.94	0	Retroviridae	eukaryote
v1634	10852	12	1	0	High-quality	100	0	unclassified	unclassified
v1635	15936	14	2	0	High-quality	100	0	Retroviridae	eukaryote
v1636	19340	11	3	0	High-quality	100	0	Retroviridae	eukaryote
v1637	12878	16	1	0	High-quality	100	0	unclassified	unclassified
v1638	12443	10	1	0	High-quality	100	0	unclassified	unclassified
v1639	16472	12	5	0	High-quality	97.35	0	unclassified	unclassified
v163a	39844	38	3	0	High-quality	100	0	unclassified	unclassified
v163b	12942	12	6	0	High-quality	100	0	unclassified	unclassified
v163c	10542	11	1	0	High-quality	100	0	unclassified	unclassified
v163d	12286	13	3	0	High-quality	98.31	0	Retroviridae	eukaryote
v163e	30888	29	6	0	High-quality	100	0	unclassified	unclassified
v163f	33985	19	1	0	High-quality	100	0	unclassified	unclassified
v1640	7961	10	1	0	Medium-quality	69.74	0	Retroviridae	eukaryote
v1641	9047	7	3	0	High-quality	97.16	0	unclassified	unclassified
v1642	7962	6	1	0	High-quality	93.4	0	unclassified	unclassified
v1643	13909	12	5	0	High-quality	98.72	0	Retroviridae	eukaryote
v1644	16746	19	2	0	High-quality	91.82	0	unclassified	unclassified
v1645	20350	14	4	0	High-quality	100	0	unclassified	unclassified
v1646	25321	28	7	0	High-quality	100	0	Retroviridae	eukaryote
v1647	7411	7	2	0	Medium-quality	64.92	0	unclassified	unclassified
v1648	12074	17	2	0	High-quality	100	0	unclassified	unclassified
v1649	14593	18	5	0	High-quality	100	0	Retroviridae	eukaryote
v164a	7051	8	1	0	High-quality	100	0	unclassified	unclassified
v164b	12901	16	1	0	High-quality	100	0	unclassified	unclassified
v164c	20640	19	4	0	High-quality	100	0	Retroviridae	eukaryote
v164d	9647	8	1	0	Medium-quality	89.94	0	Retroviridae	eukaryote
v164e	14034	16	2	0	Medium-quality	81.76	0	unclassified	unclassified
v164f	46234	31	1	0	High-quality	100	0	unclassified	unclassified
v1650	16924	20	2	0	High-quality	100	0	unclassified	unclassified
v1651	30547	25	6	0	Medium-quality	88.7	0	Retroviridae	eukaryote
v1652	8757	8	1	0	Medium-quality	81.64	0	Retroviridae	eukaryote
v1653	16556	13	3	0	High-quality	99.38	0	Retroviridae	eukaryote

v1654	16085	9	1	0	High-quality	100	0	unclassified	unclassified
v1655	17724	11	1	0	High-quality	100	0	unclassified	unclassified
v1656	6265	7	1	0	High-quality	100	0	unclassified	unclassified
v1657	24963	20	4	0	High-quality	100	0	unclassified	unclassified
v1658	16873	13	5	0	High-quality	99.46	0	unclassified	unclassified
v1659	15576	11	1	0	High-quality	100	0	unclassified	unclassified
v165a	11696	9	1	0	High-quality	100	0	unclassified	unclassified
v165b	22506	18	6	0	Low-quality	28.06	0	unclassified	unclassified
v165c	16564	11	2	0	High-quality	100	0	Retroviridae	eukaryote
v165d	7472	7	1	0	High-quality	100	0	unclassified	unclassified
v165e	18515	18	1	0	High-quality	100	0	unclassified	unclassified
v165f	13431	9	1	0	High-quality	100	0	unclassified	unclassified
v1660	21923	21	3	0	High-quality	100	0	unclassified	unclassified
v1661	17962	21	4	0	Medium-quality	86.93	0	unclassified	unclassified
v1662	10033	13	1	0	High-quality	100	0	unclassified	unclassified
v1663	21715	18	1	0	High-quality	100	0	unclassified	unclassified
v1664	7002	7	1	0	High-quality	100	0	unclassified	unclassified
v1665	12704	11	1	0	High-quality	100	0	Retroviridae	eukaryote
v1666	6831	11	1	0	High-quality	100	0	unclassified	unclassified
v1667	10110	9	2	0	High-quality	94.26	0	unclassified	unclassified
v1668	9468	15	1	0	High-quality	92.77	0	unclassified	unclassified
v1669	7553	8	2	0	Medium-quality	88.21	0	Retroviridae	eukaryote
v166a	22007	15	1	0	High-quality	100	0	unclassified	unclassified
v166b	29444	23	4	0	High-quality	100	0	Retroviridae	eukaryote
v166c	11748	9	2	0	High-quality	100	0	unclassified	unclassified
v166d	17524	18	3	0	High-quality	100	0	unclassified	unclassified
v166e	15147	16	2	0	High-quality	100	0	Retroviridae	eukaryote
v166f	19898	16	2	0	High-quality	100	0	unclassified	unclassified
v1670	20797	17	2	0	High-quality	100	0	unclassified	unclassified
v1671	10355	13	1	0	High-quality	96.54	0	unclassified	unclassified
v1672	12720	16	2	0	High-quality	100	0	Retroviridae	eukaryote
v1673	8704	10	1	0	Medium-quality	81.15	0	Retroviridae	eukaryote
v1674	25546	20	5	0	High-quality	100	0	unclassified	unclassified
v1675	21247	16	2	0	High-quality	100	0	unclassified	unclassified
v1676	9698	11	3	0	High-quality	96.4	0	unclassified	unclassified
v1677	26333	40	8	0	High-quality	100	0	Retroviridae	eukaryote
v1678	9946	6	1	0	High-quality	92.73	0	Retroviridae	eukaryote
v1679	15020	18	4	0	High-quality	100	0	Retroviridae	eukaryote
v167a	15235	16	3	0	High-quality	99.06	0	Retroviridae	eukaryote
v167b	5958	9	1	0	High-quality	96.08	0	unclassified	unclassified

v167c	26497	16	2	0	High-quality	100	0	unclassified	unclassified
v167d	18993	10	3	0	High-quality	100	0	Retroviridae	eukaryote
v167e	11026	7	1	0	High-quality	100	0	Retroviridae	eukaryote
v167f	8332	6	1	0	Medium-quality	81.64	0	unclassified	unclassified
v1680	8936	8	1	0	High-quality	100	0	unclassified	unclassified
v1681	17114	23	1	0	High-quality	100	0	unclassified	unclassified
v1682	37285	20	4	0	High-quality	100	0	Retroviridae	eukaryote
v1683	38687	38	5	0	High-quality	100	0	unclassified	unclassified
v1684	17308	14	2	0	High-quality	100	0	unclassified	unclassified
v1685	8597	10	2	0	Medium-quality	55.71	0	unclassified	unclassified
v1686	16013	12	1	0	High-quality	100	0	unclassified	unclassified
v1687	23730	16	1	0	High-quality	100	0	unclassified	unclassified
v1688	7301	10	1	0	High-quality	100	0	Retroviridae	eukaryote
v1689	15032	15	1	0	Medium-quality	79.31	0	unclassified	unclassified
v168a	26240	27	2	0	High-quality	100	0	unclassified	unclassified
v168b	20158	19	6	0	High-quality	100	0	Retroviridae	eukaryote
v168c	6611	7	2	0	Medium-quality	77.55	0	unclassified	unclassified
v168d	10472	10	1	0	High-quality	97.63	0	Retroviridae	eukaryote
v168e	29614	21	1	0	High-quality	100	0	unclassified	unclassified
v168f	12030	7	1	0	High-quality	100	0	unclassified	unclassified
v1690	12928	7	1	0	High-quality	100	0	unclassified	unclassified
v1691	13346	15	1	0	Medium-quality	70.41	0	unclassified	unclassified
v1692	9447	9	3	0	High-quality	92.31	0	unclassified	unclassified
v1693	11960	10	2	0	High-quality	100	0	unclassified	unclassified
v1694	7245	12	3	0	Medium-quality	63.47	0	Retroviridae	eukaryote
v1695	11007	13	1	0	High-quality	100	0	unclassified	unclassified
v1696	12253	9	1	0	Medium-quality	64.65	0	unclassified	unclassified
v1697	6381	5	1	0	High-quality	100	0	unclassified	unclassified
v1698	16898	13	5	0	High-quality	99.49	0	Retroviridae	eukaryote
v1699	5860	8	1	0	Medium-quality	54.63	0	unclassified	unclassified
v169a	26646	21	2	0	High-quality	100	0	unclassified	unclassified
v169b	11387	15	1	0	High-quality	100	0	unclassified	unclassified
v169c	20087	16	2	0	High-quality	96.84	0	unclassified	unclassified
v169d	10491	13	1	0	High-quality	100	0	unclassified	unclassified
v169e	15400	9	1	0	High-quality	100	0	unclassified	unclassified
v169f	19074	12	1	0	High-quality	100	0	unclassified	unclassified
v16a0	6127	10	1	0	High-quality	98.81	0	unclassified	unclassified
v16a1	16449	18	2	0	High-quality	100	0	Retroviridae	eukaryote
v16a2	8445	7	2	0	Medium-quality	78.73	0	Retroviridae	eukaryote
v16a3	22334	23	1	0	High-quality	100	0	unclassified	unclassified

v16a4	16639	12	1	0	High-quality	100	0	unclassified	unclassified
v16a5	9379	13	1	0	High-quality	100	0	unclassified	unclassified
v16a6	7553	10	1	0	High-quality	100	0	unclassified	unclassified
v16a7	18523	15	2	0	High-quality	100	0	unclassified	unclassified
v16a8	19128	13	3	0	High-quality	100	0	Retroviridae	eukaryote
v16a9	14849	11	1	0	High-quality	100	0	unclassified	unclassified
v16aa	14919	19	3	0	High-quality	100	0	unclassified	unclassified
v16ab	14386	12	1	0	High-quality	100	0	unclassified	unclassified
v16ac	18045	13	2	0	High-quality	100	0	Retroviridae	eukaryote
v16ad	5555	6	1	0	Medium-quality	89.58	0	unclassified	unclassified
v16ae	11639	4	1	0	High-quality	100	0	unclassified	unclassified
v16af	7618	12	1	0	Medium-quality	71.02	0	unclassified	unclassified
v16b0	20279	20	4	0	High-quality	100	0	Retroviridae	eukaryote
v16b1	11887	21	1	0	High-quality	100	0	unclassified	unclassified
v16b2	17603	21	5	0	High-quality	100	0	unclassified	unclassified
v16b3	6802	9	1	0	Medium-quality	63.41	0	unclassified	unclassified
v16b4	8559	5	2	0	Medium-quality	74.98	0	Retroviridae	eukaryote
v16b5	11151	12	2	0	High-quality	100	0	Retroviridae	eukaryote
v16b6	11135	12	1	0	High-quality	100	0	Retroviridae	eukaryote
v16b7	11839	16	1	0	High-quality	100	0	unclassified	unclassified
v16b8	6061	10	1	0	Medium-quality	56.51	0	unclassified	unclassified
v16b9	9723	11	1	0	High-quality	90.65	0	unclassified	unclassified
v16ba	7841	8	3	0	High-quality	94.73	0	Retroviridae	eukaryote
v16bb	9804	10	1	0	Medium-quality	85.88	0	Retroviridae	eukaryote
v16bc	9751	5	2	0	High-quality	93.74	0	unclassified	unclassified
v16bd	6386	8	1	0	High-quality	100	0	Retroviridae	eukaryote
v16be	20908	20	1	0	High-quality	100	0	Retroviridae	eukaryote
v16bf	14905	13	2	0	High-quality	100	0	unclassified	unclassified
v16c0	29464	17	1	0	High-quality	100	0	unclassified	unclassified
v16c1	43739	80	22	0	Complete	100	0	Siphoviridae	prokaryote
v16c2	37483	47	25	3	Medium-quality	81.98	0	Myoviridae	prokaryote
v16c3	90015	119	11	2	Medium-quality	58.51	0	unclassified	unclassified
v16c4	40445	56	7	2	Complete	100	0	unclassified	unclassified
v16c5	27956	37	6	0	Medium-quality	64.79	0	unclassified	unclassified
v16c6	44255	68	16	1	High-quality	100	0	unclassified	unclassified
v16c7	34182	50	8	0	Complete	100	0	unclassified	unclassified
v16c8	219712	334	46	4	Complete	100	0	unclassified	unclassified
v16c9	55094	63	13	4	High-quality	100	0	unclassified	unclassified
v16ca	64248	80	12	2	Complete	100	0	unclassified	unclassified
v16cb	104218	150	29	1	Complete	100	0	Podoviridae_crAss-like	prokaryote

v16cc	93456	101	18	7	High-quality	100	0	unclassified	unclassified
v16cd	34108	56	9	0	Medium-quality	53.06	0	Quimbyviridae	prokaryote
v16ce	56824	59	9	4	Medium-quality	80.43	0	unclassified	unclassified
v16cf	26084	26	14	0	Medium-quality	65.59	0	unclassified	unclassified
v16d0	37590	58	17	0	High-quality	100	0	unclassified	unclassified
v16d1	77626	112	11	2	Medium-quality	81.99	0	Quimbyviridae	prokaryote
v16d2	37916	56	34	1	Medium-quality	85.3	0	Siphoviridae	prokaryote
v16d3	20394	30	11	0	Medium-quality	53.3	0	Siphoviridae	prokaryote
v16d4	40329	57	8	1	Medium-quality	60.97	0	unclassified	unclassified
v16d5	11251	12	1	0	High-quality	100	0	unclassified	unclassified
v16d6	41278	50	19	0	High-quality	100	0	Siphoviridae	prokaryote
v16d7	77114	102	27	2	High-quality	99.94	0	unclassified	unclassified
v16d8	23712	26	13	0	Medium-quality	59.53	0	Siphoviridae	prokaryote
v16d9	53129	78	15	2	Medium-quality	88.95	0	unclassified	unclassified
v16da	35737	60	21	1	High-quality	98.93	0	Siphoviridae	prokaryote
v16db	49361	64	12	1	Complete	100	0	unclassified	unclassified
v16dc	27205	25	10	0	Medium-quality	65.87	0	unclassified	unclassified
v16dd	43466	53	20	1	High-quality	100	0	Siphoviridae	prokaryote
v16de	37316	54	32	0	High-quality	95.78	0	Siphoviridae	prokaryote
v16df	38643	67	33	0	Medium-quality	54.24	0	Myoviridae	prokaryote
v16e0	55303	65	26	2	High-quality	96.83	0	Siphoviridae	prokaryote
v16e1	29175	33	2	1	Medium-quality	64.48	0	unclassified	unclassified
v16e2	43901	53	40	1	High-quality	100	0	Siphoviridae	prokaryote
v16e3	20947	22	18	0	Medium-quality	60.3	0	Siphoviridae	prokaryote
v16e4	34088	50	14	0	High-quality	99.83	0	unclassified	unclassified
v16e5	42025	49	18	0	High-quality	100	0	Siphoviridae	prokaryote
v16e6	33244	39	27	0	Medium-quality	81.95	0	Myoviridae	prokaryote
v16e7	19026	23	4	3	Medium-quality	66.32	0	unclassified	unclassified
v16e8	18303	21	18	0	Medium-quality	55.63	0	Siphoviridae	prokaryote
v16e9	40786	53	30	0	Complete	100	0	Myoviridae	prokaryote
v16ea	38313	61	35	0	High-quality	100	0	Siphoviridae	prokaryote
v16eb	34720	44	21	0	Medium-quality	89.24	0	Siphoviridae	prokaryote
v16ec	17162	14	4	0	Medium-quality	50.41	0	unclassified	unclassified
v16ed	40494	54	30	0	Complete	100	0	Myoviridae	prokaryote
v16ee	40777	49	20	0	High-quality	97.84	0	Siphoviridae	prokaryote
v16ef	67626	82	21	8	High-quality	100	0	Siphoviridae	prokaryote
v16f0	45981	72	16	1	Complete	100	0	unclassified	unclassified
v16f1	40772	61	7	0	High-quality	98.48	0	unclassified	unclassified
v16f2	60501	112	27	0	Complete	100	0	unclassified	unclassified
v16f3	37412	62	16	0	Medium-quality	78.07	0	Myoviridae	prokaryote

v16f4	37955	62	23	0	Complete	100	0	Siphoviridae	prokaryote
v16f5	38132	47	11	1	Medium-quality	84.41	0	Siphoviridae	prokaryote
v16f6	28487	35	8	0	Medium-quality	83.47	0	unclassified	unclassified
v16f7	102518	102	15	1	Complete	100	0	Podoviridae_crAss-like	prokaryote
v16f8	42522	66	12	1	High-quality	100	0	Siphoviridae	prokaryote
v16f9	13382	16	4	0	Complete	100	0	unclassified	unclassified
v16fa	102690	187	21	2	Complete	100	0	Podoviridae_crAss-like	prokaryote
v16fb	75658	80	10	1	High-quality	100	0	unclassified	unclassified
v16fc	37644	66	18	2	Complete	100	0	unclassified	unclassified
v16fd	42078	56	17	3	High-quality	100	0	unclassified	unclassified
v16fe	58240	93	33	1	Complete	100	0	Myoviridae	prokaryote
v16ff	12956	16	5	0	High-quality	95.17	0	unclassified	unclassified
v1700	97108	131	6	2	High-quality	99.94	0	unclassified	unclassified
v1701	13135	16	4	0	Complete	100	0	unclassified	unclassified
v1702	48896	91	18	0	Complete	100	0	unclassified	unclassified
v1703	153495	191	16	5	Complete	100	0	unclassified	unclassified
v1704	39889	58	22	1	High-quality	100	0	Siphoviridae	prokaryote
v1705	83039	133	32	4	High-quality	100	0	unclassified	unclassified
v1706	102776	117	17	2	High-quality	98.59	0	Gratiaviridae	prokaryote
v1707	26487	35	13	0	Medium-quality	77.58	0	unclassified	unclassified
v1708	36637	56	18	0	Medium-quality	81.63	0	Myoviridae	prokaryote
v1709	5503	6	1	0	High-quality	100	0	Myoviridae	prokaryote
v170a	106577	113	13	0	Complete	100	0	Podoviridae_crAss-like	prokaryote
v170b	57347	86	12	2	Complete	100	0	unclassified	unclassified
v170c	77914	131	20	6	Complete	100	0	unclassified	unclassified
v170d	55791	75	16	2	High-quality	100	0	Myoviridae	prokaryote
v170e	49064	62	12	0	Complete	100	0	Siphoviridae	prokaryote
v170f	47246	62	31	0	High-quality	100	0	Myoviridae	prokaryote
v1710	47022	72	16	0	High-quality	100	0	Siphoviridae	prokaryote
v1711	47238	74	12	1	High-quality	100	0	unclassified	unclassified
v1712	57399	98	19	1	Complete	100	0	unclassified	unclassified
v1713	76261	116	23	3	Complete	100	0	unclassified	unclassified
v1714	60847	67	9	3	High-quality	100	0	unclassified	unclassified
v1715	112829	133	18	5	High-quality	100	0	unclassified	unclassified
v1716	80038	78	7	6	High-quality	100	0	unclassified	unclassified
v1717	30280	45	14	0	Medium-quality	52.47	0	unclassified	unclassified
v1718	39618	40	4	3	Medium-quality	84.16	0	unclassified	unclassified
v1719	21668	34	21	0	Medium-quality	50.81	0	Myoviridae	prokaryote
v171a	58870	99	16	2	Complete	100	0	unclassified	unclassified
v171b	34036	58	11	1	Complete	100	0	Siphoviridae	prokaryote

v171c	41954	64	12	2	Complete	100	0	unclassified	unclassified
v171d	35933	66	14	0	High-quality	97.64	0	unclassified	unclassified
v171e	47789	93	23	1	Complete	100	0	unclassified	unclassified
v171f	53495	61	6	1	Medium-quality	79.54	0	unclassified	unclassified
v1720	5666	8	2	0	Complete	100	0	unclassified	unclassified
v1721	5962	9	4	0	Complete	100	0	Microviridae	prokaryote
v1722	60270	62	14	13	High-quality	100	0	unclassified	unclassified
v1723	17239	22	8	0	Medium-quality	80.81	0	unclassified	unclassified
v1724	60993	72	25	4	High-quality	100	0	Myoviridae	prokaryote
v1725	54005	42	9	2	Medium-quality	81.6	0	unclassified	unclassified
v1726	5735	9	6	0	Complete	100	0	Microviridae	prokaryote
v1727	46251	43	6	5	High-quality	95.55	0	unclassified	unclassified
v1728	65617	114	21	1	Complete	100	0	unclassified	unclassified
v1729	47430	77	30	2	High-quality	100	0	Siphoviridae	prokaryote
v172a	6170	7	3	0	Complete	100	0	unclassified	unclassified
v172b	14362	18	5	0	High-quality	100	0	unclassified	unclassified
v172c	28474	50	21	0	Medium-quality	85.86	0	Siphoviridae	prokaryote
v172d	112815	173	33	3	Medium-quality	63.36	0	unclassified	unclassified
v172e	43678	82	17	0	High-quality	100	0	unclassified	unclassified
v172f	54657	87	7	2	Medium-quality	50.17	0	unclassified	unclassified
v1730	34175	41	16	4	High-quality	93.65	0	Siphoviridae	prokaryote
v1731	101943	157	11	2	Complete	100	0	Podoviridae_crAss-like	prokaryote
v1732	44459	71	20	0	Complete	100	0	unclassified	unclassified
v1733	27952	42	19	0	Medium-quality	68.74	0	Siphoviridae	prokaryote
v1734	15822	22	6	0	Medium-quality	68.9	0	unclassified	unclassified
v1735	191675	336	27	3	Complete	100	0	unclassified	unclassified
v1736	12843	17	8	1	Complete	100	0	Siphoviridae	prokaryote
v1737	68255	81	12	3	High-quality	100	0	unclassified	unclassified
v1738	47721	56	10	5	High-quality	100	0	unclassified	unclassified
v1739	153172	221	40	2	High-quality	94.54	0	unclassified	unclassified
v173a	15381	21	6	0	Medium-quality	67.89	0	unclassified	unclassified
v173b	12900	19	6	0	Medium-quality	56.86	0	Salasmaviridae	prokaryote
v173c	27035	35	24	1	Medium-quality	84.65	0	Myoviridae	prokaryote
v173d	5727	9	5	0	Complete	100	0	Microviridae	prokaryote
v173e	44460	79	17	3	Medium-quality	55.14	0	unclassified	unclassified
v173f	25206	50	5	1	Medium-quality	64.54	0	unclassified	unclassified
v1740	195150	227	39	8	Complete	100	0	unclassified	unclassified
v1741	30437	38	17	0	Medium-quality	73.78	0	Siphoviridae	prokaryote
v1742	34809	42	13	0	Medium-quality	84.42	0	unclassified	unclassified
v1743	44690	76	18	0	High-quality	100	0	unclassified	unclassified

v1744	55415	86	15	2	High-quality	100	0	Myoviridae	prokaryote
v1745	46554	77	19	0	Complete	100	0	unclassified	unclassified
v1746	34555	39	19	2	High-quality	98.25	0	Siphoviridae	prokaryote
v1747	139502	184	43	1	Complete	100	0	unclassified	unclassified
v1748	42831	73	19	0	High-quality	100	0	unclassified	unclassified
v1749	15127	21	7	0	High-quality	90.87	0	unclassified	unclassified
v174a	32933	38	16	0	Complete	100	0	Siphoviridae	prokaryote
v174b	13240	16	4	0	High-quality	99.11	0	unclassified	unclassified
v174c	124813	172	8	6	High-quality	100	0	unclassified	unclassified
v174d	66591	72	24	4	High-quality	100	0	Siphoviridae	prokaryote
v174e	58791	97	12	2	Complete	100	0	unclassified	unclassified
v174f	42921	73	20	0	Complete	100	0	unclassified	unclassified
v1750	36949	53	7	3	Medium-quality	51.73	0	Myoviridae	prokaryote
v1751	61693	76	11	4	Complete	100	0	unclassified	unclassified
v1752	22466	33	8	0	Medium-quality	51.67	0	unclassified	unclassified
v1753	41981	54	10	0	High-quality	100	0	unclassified	unclassified
v1754	16032	22	8	0	Medium-quality	84.44	0	unclassified	unclassified
v1755	150110	205	19	3	Medium-quality	83.27	0	unclassified	unclassified
v1756	15981	22	8	0	Medium-quality	85.53	0	unclassified	unclassified
v1757	24829	32	15	0	Medium-quality	53.33	0	Siphoviridae	prokaryote
v1758	39031	59	17	0	Complete	100	0	Siphoviridae	prokaryote
v1759	17033	21	2	1	High-quality	100	0	unclassified	unclassified
v175a	44771	75	18	0	High-quality	99.24	0	unclassified	unclassified
v175b	82839	93	6	5	High-quality	100	0	unclassified	unclassified
v175c	37299	53	15	3	High-quality	98.22	0	unclassified	unclassified
v175d	39326	43	17	3	Medium-quality	73.43	0	Siphoviridae	prokaryote
v175e	34198	47	13	1	Medium-quality	60.85	0	unclassified	unclassified
v175f	101451	172	19	1	Complete	100	0	Podoviridae_crAss-like	prokaryote
v1760	57666	108	23	0	Complete	100	0	unclassified	unclassified
v1761	84524	138	26	2	Complete	100	0	unclassified	unclassified
v1762	47231	80	18	3	Medium-quality	78.15	0	Quimbyviridae	prokaryote
v1763	44469	56	28	4	Medium-quality	83.12	0	Siphoviridae	prokaryote
v1764	122428	165	32	5	Medium-quality	71.94	0	unclassified	unclassified
v1765	24053	35	27	0	Medium-quality	52.94	0	Siphoviridae	prokaryote
v1766	8217	15	1	0	Medium-quality	84.2	0	unclassified	unclassified
v1767	36095	48	19	2	Medium-quality	71.43	0	Siphoviridae	prokaryote
v1768	57988	100	14	2	Complete	100	0	unclassified	unclassified
v1769	49449	72	23	1	Complete	100	0	Siphoviridae	prokaryote
v176a	32074	47	9	2	Medium-quality	55.31	0	unclassified	unclassified
v176b	43959	58	8	1	Complete	100	0	unclassified	unclassified

v176c	35059	58	11	0	Medium-quality	63.7	0	unclassified	unclassified
v176d	35812	56	13	0	Medium-quality	62.14	0	unclassified	unclassified
v176e	46536	77	19	0	Complete	100	0	unclassified	unclassified
v176f	37691	62	17	0	High-quality	100	0	unclassified	unclassified
v1770	21586	22	5	2	Medium-quality	75.26	0	unclassified	unclassified
v1771	26727	31	7	2	Medium-quality	59.12	0	Siphoviridae	prokaryote
v1772	5077	7	6	0	Complete	100	0	Microviridae	prokaryote
v1773	55174	97	15	0	High-quality	100	0	unclassified	unclassified
v1774	52393	86	15	1	Medium-quality	89.31	0	unclassified	unclassified
v1775	47291	80	22	1	High-quality	100	0	unclassified	unclassified
v1776	30984	53	13	1	Complete	100	0	unclassified	unclassified
v1777	24995	31	12	0	Medium-quality	72.42	0	unclassified	unclassified
v1778	40082	61	49	0	High-quality	100	0	Podoviridae	prokaryote
v1779	46557	73	16	0	Complete	100	0	unclassified	unclassified
v177a	38360	48	23	0	High-quality	94.2	0	Myoviridae	prokaryote
v177b	15022	17	7	0	Medium-quality	64.16	0	Salasmaviridae	prokaryote
v177c	33800	53	11	0	Complete	100	0	unclassified	unclassified
v177d	38771	55	11	0	High-quality	95.93	0	unclassified	unclassified
v177e	89253	153	38	0	High-quality	100	0	unclassified	unclassified
v177f	16241	27	2	1	High-quality	100	0	unclassified	unclassified
v1780	39515	56	21	1	High-quality	100	0	Siphoviridae	prokaryote
v1781	78994	128	24	2	High-quality	98.02	0	unclassified	unclassified
v1782	32112	35	16	1	Medium-quality	69.12	0	Siphoviridae	prokaryote
v1783	47397	58	18	2	High-quality	100	0	Siphoviridae	prokaryote
v1784	41753	68	18	1	Medium-quality	86.89	0	Myoviridae	prokaryote
v1785	21542	38	27	0	Medium-quality	54.9	0	Siphoviridae	prokaryote
v1786	29661	42	16	1	Medium-quality	83.79	0	Siphoviridae	prokaryote
v1787	44960	71	14	1	Medium-quality	55.77	0	unclassified	unclassified
v1788	103531	120	26	1	Complete	100	0	unclassified	unclassified
v1789	65897	106	11	2	Medium-quality	77.71	0	unclassified	unclassified
v178a	26779	27	4	3	High-quality	93.34	0	unclassified	unclassified
v178b	90984	141	27	6	Complete	100	0	unclassified	unclassified
v178c	46085	67	21	1	Medium-quality	84.74	0	Myoviridae	prokaryote
v178d	12915	20	6	1	High-quality	100	0	unclassified	unclassified
v178e	18776	35	6	0	Medium-quality	57.03	0	Siphoviridae	prokaryote
v178f	29484	50	20	0	Complete	100	0	Siphoviridae	prokaryote
v1790	50594	48	10	2	Medium-quality	56.51	0	unclassified	unclassified
v1791	39861	62	16	0	Complete	100	0	unclassified	unclassified
v1792	44811	67	10	3	Medium-quality	79.4	0	unclassified	unclassified
v1793	42742	77	19	1	Complete	100	0	Siphoviridae	prokaryote

v1794	62468	72	9	5	High-quality	100	0	unclassified	unclassified
v1795	102996	136	8	3	High-quality	100	0	unclassified	unclassified
v1796	34523	47	12	0	Complete	100	0	Siphoviridae	prokaryote
v1797	37191	50	13	1	Medium-quality	60.61	0	Quimbyviridae	prokaryote
v1798	28643	36	20	1	High-quality	94.04	0	Myoviridae	prokaryote
v1799	31824	49	22	1	Complete	100	0	Siphoviridae	prokaryote
v179a	50581	70	20	0	Medium-quality	83.34	0	unclassified	unclassified
v179b	35769	49	32	0	Medium-quality	88.17	0	Siphoviridae	prokaryote
v179c	38771	72	14	1	Complete	100	0	unclassified	unclassified
v179d	5579	8	2	0	Medium-quality	82.66	0	unclassified	unclassified
v179e	93828	154	17	2	Medium-quality	62.05	0	unclassified	unclassified
v179f	29472	44	36	0	Medium-quality	71.58	0	Siphoviridae	prokaryote
v17a0	80894	115	17	2	High-quality	100	0	unclassified	unclassified
v17a1	59535	80	54	0	Complete	100	0	Siphoviridae	prokaryote
v17a2	26238	39	20	2	Medium-quality	63.16	0	Siphoviridae	prokaryote
v17a3	89184	103	14	2	Medium-quality	59.18	0	unclassified	unclassified
v17a4	55372	84	21	1	High-quality	100	0	Myoviridae	prokaryote
v17a5	40636	65	18	1	Medium-quality	82.24	0	unclassified	unclassified
v17a6	15201	26	6	0	Medium-quality	79.67	0	unclassified	unclassified
v17a7	38986	53	3	2	High-quality	100	0	unclassified	unclassified
v17a8	17255	21	3	2	Medium-quality	60.14	0	unclassified	unclassified
v17a9	28258	28	9	1	Medium-quality	59.32	0	Siphoviridae	prokaryote
v17aa	91068	142	27	2	Medium-quality	70.93	0	unclassified	unclassified
v17ab	56495	103	19	1	Complete	100	0	unclassified	unclassified
v17ac	39308	52	26	0	Medium-quality	65.27	0	Siphoviridae	prokaryote
v17ad	16860	23	11	0	Medium-quality	71.11	0	Salasmaviridae	prokaryote
v17ae	73069	96	11	2	Complete	100	0	unclassified	unclassified
v17af	97491	94	11	0	Complete	100	0	Podoviridae_crAss-like	prokaryote
v17b0	40115	52	6	0	Complete	100	0	unclassified	unclassified
v17b1	5969	10	2	0	Complete	100	0	unclassified	unclassified
v17b2	16778	19	7	0	Medium-quality	88.32	0	Salasmaviridae	prokaryote
v17b3	26498	43	24	0	Medium-quality	71.57	0	Siphoviridae	prokaryote
v17b4	42214	64	16	1	Medium-quality	89.63	0	Myoviridae	prokaryote
v17b5	41277	59	26	0	High-quality	100	0	Siphoviridae	prokaryote
v17b6	42891	73	14	0	Complete	100	0	unclassified	unclassified
v17b7	79654	119	12	0	Complete	100	0	Flandersviridae	prokaryote
v17b8	5625	6	3	0	Complete	100	0	unclassified	unclassified
v17b9	16241	26	6	0	Medium-quality	68.53	0	unclassified	unclassified
v17ba	39998	54	30	0	High-quality	98.31	0	Myoviridae	prokaryote
v17bb	42302	73	22	1	High-quality	100	0	Siphoviridae	prokaryote

v17bc	121120	166	28	3	Complete	100	0	unclassified	unclassified
v17bd	63670	103	25	1	Complete	100	0	unclassified	unclassified
v17be	15319	18	7	0	Medium-quality	65.73	0	unclassified	unclassified
v17bf	16077	25	6	0	Medium-quality	70.66	0	unclassified	unclassified
v17c0	38747	69	16	3	Complete	100	0	unclassified	unclassified
v17c1	34651	42	28	0	Medium-quality	85.18	0	Myoviridae	prokaryote
v17c2	16349	19	8	0	Medium-quality	53.52	0	unclassified	unclassified
v17c3	55143	82	30	1	High-quality	96.05	0	Siphoviridae	prokaryote
v17c4	59958	87	16	3	Complete	100	0	Quimbyviridae	prokaryote
v17c5	17170	21	7	0	Medium-quality	74.14	0	unclassified	unclassified
v17c6	9402	7	1	0	High-quality	100	0	unclassified	unclassified
v17c7	31733	57	21	1	High-quality	92.45	0	Siphoviridae	prokaryote
v17c8	30707	44	14	0	Medium-quality	83.45	0	unclassified	unclassified
v17c9	35404	66	14	3	Medium-quality	82.62	0	unclassified	unclassified
v17ca	26421	33	13	2	Medium-quality	77.77	0	Siphoviridae	prokaryote
v17cb	39122	58	17	1	Complete	100	0	Siphoviridae	prokaryote
v17cc	41754	56	48	0	Complete	100	0	Podoviridae	prokaryote
v17cd	41114	66	12	1	Complete	100	0	unclassified	unclassified
v17ce	28462	46	16	0	Medium-quality	53.96	0	unclassified	unclassified
v17cf	13278	17	7	0	High-quality	95.6	0	unclassified	unclassified
v17d0	53726	53	11	7	Medium-quality	61.38	0	unclassified	unclassified
v17d1	16989	25	7	0	Complete	100	0	unclassified	unclassified
v17d2	51398	55	9	2	Complete	100	0	unclassified	unclassified
v17d3	44033	52	21	2	Medium-quality	82.32	0	Siphoviridae	prokaryote
v17d4	68327	99	8	4	Medium-quality	81.62	0	Quimbyviridae	prokaryote
v17d5	67943	105	29	2	High-quality	100	0	unclassified	unclassified
v17d6	38554	56	24	0	High-quality	90.6	0	Myoviridae	prokaryote
v17d7	98965	147	21	2	Complete	100	0	Quimbyviridae	prokaryote
v17d8	59325	102	20	3	High-quality	98.94	0	unclassified	unclassified
v17d9	34213	47	12	0	Complete	100	0	unclassified	unclassified
v17da	48318	63	33	1	High-quality	100	0	Myoviridae	prokaryote
v17db	58767	98	17	2	Complete	100	0	unclassified	unclassified
v17dc	29142	38	16	0	Medium-quality	83.1	0	Siphoviridae	prokaryote
v17dd	112358	121	34	1	Complete	100	0	Siphoviridae	prokaryote
v17de	65681	85	23	2	Medium-quality	72.85	0	Myoviridae	prokaryote
v17df	36558	50	14	2	High-quality	100	0	unclassified	unclassified
v17e0	36462	45	9	0	Medium-quality	73.53	0	Siphoviridae	prokaryote
v17e1	37373	62	17	0	Medium-quality	89.1	0	unclassified	unclassified
v17e2	50842	69	31	1	Complete	100	0	Siphoviridae	prokaryote
v17e3	40858	63	11	1	High-quality	100	0	unclassified	unclassified

v17e4	36457	43	6	5	High-quality	100	0	unclassified	unclassified
v17e5	21527	25	14	0	Medium-quality	52.16	0	Siphoviridae	prokaryote
v17e6	31993	54	15	0	Medium-quality	89.86	0	unclassified	unclassified
v17e7	66377	116	27	0	High-quality	100	0	unclassified	unclassified
v17e8	79463	120	15	1	High-quality	100	0	Quimbyviridae	prokaryote
v17e9	24191	29	15	0	Medium-quality	55.91	0	Myoviridae	prokaryote
v17ea	80978	123	17	1	Medium-quality	85.5	0	Quimbyviridae	prokaryote
v17eb	40268	59	15	0	Complete	100	0	unclassified	unclassified
v17ec	86222	102	7	6	High-quality	100	0	unclassified	unclassified
v17ed	42271	74	20	0	High-quality	100	0	Siphoviridae	prokaryote
v17ee	39313	37	9	0	Medium-quality	51.79	0	unclassified	unclassified
v17ef	35497	50	26	0	Complete	100	0	Siphoviridae	prokaryote
v17f0	9583	15	1	0	Medium-quality	69.91	0	unclassified	unclassified
v17f1	6937	10	1	0	High-quality	100	0	unclassified	unclassified
v17f2	38395	73	14	0	High-quality	96.06	0	unclassified	unclassified
v17f3	23366	45	12	1	High-quality	100	0	unclassified	unclassified
v17f4	252855	283	36	5	High-quality	100	0	Podoviridae_crAss-like	prokaryote
v17f5	74629	128	18	2	Medium-quality	51.61	0	unclassified	unclassified
v17f6	195012	300	53	18	High-quality	100	0	unclassified	unclassified
v17f7	80940	142	23	4	Complete	100	0	unclassified	unclassified
v17f8	15928	21	6	0	Medium-quality	89.34	0	unclassified	unclassified
v17f9	29661	39	22	0	Medium-quality	73.49	0	Siphoviridae	prokaryote
v17fa	54923	101	14	0	High-quality	100	0	unclassified	unclassified
v17fb	38905	70	19	0	High-quality	92.65	0	unclassified	unclassified
v17fc	22396	35	14	1	Medium-quality	53.94	0	Siphoviridae	prokaryote
v17fd	6445	7	4	0	High-quality	100	0	unclassified	unclassified
v17fe	58746	106	18	2	High-quality	100	0	unclassified	unclassified
v17ff	99205	107	23	14	High-quality	100	0	Siphoviridae	prokaryote
v1800	98785	156	21	1	Complete	100	0	Podoviridae_crAss-like	prokaryote
v1801	152430	197	36	4	High-quality	100	0	unclassified	unclassified
v1802	20907	28	3	2	Medium-quality	54.26	0	Siphoviridae	prokaryote
v1803	13957	19	6	0	High-quality	100	0	unclassified	unclassified
v1804	42646	71	18	0	High-quality	100	0	unclassified	unclassified
v1805	40341	67	17	0	Complete	100	0	unclassified	unclassified
v1806	37175	61	47	0	Medium-quality	87.94	0	Siphoviridae	prokaryote
v1807	61332	109	19	3	Complete	100	0	unclassified	unclassified
v1808	78683	118	16	5	High-quality	100	0	unclassified	unclassified
v1809	145206	196	39	3	High-quality	100	0	unclassified	unclassified
v180a	52414	88	21	1	High-quality	100	0	unclassified	unclassified
v180b	95617	169	18	1	Complete	100	0	Podoviridae_crAss-like	prokaryote

v180c	100446	155	44	0	High-quality	100	0	Myoviridae	prokaryote
v180d	37226	70	23	1	Medium-quality	89.74	0	Siphoviridae	prokaryote
v180e	39139	49	5	4	Medium-quality	65.89	0	unclassified	unclassified
v180f	42876	68	15	0	Medium-quality	74.37	0	unclassified	unclassified
v1810	31197	39	15	2	Medium-quality	58.18	0	unclassified	unclassified
v1811	131987	237	28	6	Complete	100	0	unclassified	unclassified
v1812	87614	136	18	4	High-quality	100	0	unclassified	unclassified
v1813	41308	55	37	0	Medium-quality	73.4	0	Myoviridae	prokaryote
v1814	48030	37	4	0	Medium-quality	76.4	0	unclassified	unclassified
v1815	44985	75	49	0	High-quality	90.56	0	Siphoviridae	prokaryote
v1816	38025	60	11	1	High-quality	93	0	unclassified	unclassified
v1817	36815	49	7	0	Medium-quality	81.06	0	unclassified	unclassified
v1818	36799	46	21	0	High-quality	95.96	0	Siphoviridae	prokaryote
v1819	44917	74	17	0	Complete	100	0	unclassified	unclassified
v181a	28959	38	19	0	Medium-quality	68.43	0	Siphoviridae	prokaryote
v181b	37586	59	27	0	Complete	100	0	Siphoviridae	prokaryote
v181c	140239	160	15	4	High-quality	100	0	unclassified	unclassified
v181d	57947	95	19	2	High-quality	100	0	unclassified	unclassified
v181e	30632	57	9	1	Medium-quality	62.56	0	unclassified	unclassified
v181f	44777	72	17	0	Complete	100	0	unclassified	unclassified
v1820	28533	28	17	0	Medium-quality	71	0	Siphoviridae	prokaryote
v1821	37389	52	11	0	Medium-quality	87.57	0	unclassified	unclassified
v1822	121829	175	25	13	Medium-quality	88.41	0	unclassified	unclassified
v1823	82296	119	23	3	Complete	100	0	unclassified	unclassified
v1824	54861	101	19	1	High-quality	96.01	0	unclassified	unclassified
v1825	46147	69	18	1	High-quality	100	0	unclassified	unclassified
v1826	78394	115	10	1	Medium-quality	82.8	0	Quimbyviridae	prokaryote
v1827	42121	71	16	1	High-quality	100	0	unclassified	unclassified
v1828	133755	163	37	2	Complete	100	0	unclassified	unclassified
v1829	18981	24	11	1	Medium-quality	84.14	0	unclassified	unclassified
v182a	53149	94	12	3	High-quality	92.26	0	unclassified	unclassified
v182b	38805	60	7	1	Complete	100	0	unclassified	unclassified
v182c	40797	50	18	3	High-quality	100	0	Siphoviridae	prokaryote
v182d	179406	145	28	2	Medium-quality	82.31	0	unclassified	unclassified
v182e	41783	57	26	0	High-quality	92.83	0	Myoviridae	prokaryote
v182f	31311	48	14	0	Medium-quality	63.94	0	Siphoviridae	prokaryote
v1830	26638	54	5	0	Medium-quality	65.55	0	unclassified	unclassified
v1831	23241	40	15	0	Medium-quality	63.68	0	Siphoviridae	prokaryote
v1832	55811	82	19	0	Medium-quality	72.28	0	unclassified	unclassified
v1833	27714	53	13	0	Medium-quality	65.88	0	unclassified	unclassified

v1834	19911	40	5	1	Medium-quality	51.73	0	unclassified	unclassified
v1835	40949	70	20	0	Complete	100	0	unclassified	unclassified
v1836	23116	28	21	0	Medium-quality	66.67	0	Siphoviridae	prokaryote
v1837	41553	69	18	1	Complete	100	0	Siphoviridae	prokaryote
v1838	53163	97	18	0	Complete	100	0	unclassified	unclassified
v1839	39988	68	22	2	Complete	100	0	Siphoviridae	prokaryote
v183a	44183	66	22	3	High-quality	100	0	Siphoviridae	prokaryote
v183b	47656	65	20	1	High-quality	97.82	0	Siphoviridae	prokaryote
v183c	70646	84	14	7	Medium-quality	87.36	0	unclassified	unclassified
v183d	62538	69	6	5	High-quality	100	0	unclassified	unclassified
v183e	38795	48	8	0	High-quality	99.09	0	unclassified	unclassified
v183f	41904	78	16	1	High-quality	100	0	unclassified	unclassified
v1840	38790	65	17	4	Complete	100	0	unclassified	unclassified
v1841	19394	24	2	1	High-quality	100	0	unclassified	unclassified
v1842	42155	74	19	0	High-quality	99.75	0	unclassified	unclassified
v1843	33485	52	16	0	Medium-quality	83.64	0	Siphoviridae	prokaryote
v1844	25262	39	26	0	Medium-quality	59.51	0	Myoviridae	prokaryote
v1845	44908	69	17	0	High-quality	100	0	unclassified	unclassified
v1846	38432	58	14	0	High-quality	99.26	0	Siphoviridae	prokaryote
v1847	45802	72	23	0	High-quality	100	0	unclassified	unclassified
v1848	56500	101	20	3	High-quality	94.22	0	unclassified	unclassified
v1849	26332	41	11	0	Medium-quality	56.35	0	unclassified	unclassified
v184a	75902	81	17	9	High-quality	100	0	unclassified	unclassified
v184b	42256	70	17	0	Complete	100	0	unclassified	unclassified
v184c	37625	66	21	1	Complete	100	0	unclassified	unclassified
v184d	32052	48	8	5	Medium-quality	52.23	0	unclassified	unclassified
v184e	42525	51	19	1	High-quality	100	0	Siphoviridae	prokaryote
v184f	44740	71	42	0	High-quality	100	0	Myoviridae	prokaryote
v1850	190141	323	28	2	Complete	100	0	unclassified	unclassified
v1851	51988	46	9	7	High-quality	100	0	unclassified	unclassified
v1852	20293	34	4	1	Complete	100	0	unclassified	unclassified
v1853	50434	69	7	1	Medium-quality	59.54	0	Quimbyviridae	prokaryote
v1854	101633	172	15	1	Complete	100	0	Podoviridae_crAss-like	prokaryote
v1855	34476	50	19	1	Complete	100	0	Siphoviridae	prokaryote
v1856	52506	92	18	0	Medium-quality	86.18	0	unclassified	unclassified
v1857	42204	84	13	0	Complete	100	0	unclassified	unclassified
v1858	149113	208	42	6	Complete	100	0	unclassified	unclassified
v1859	31651	42	25	2	Medium-quality	67.7	0	Siphoviridae	prokaryote
v185a	71297	89	12	5	Medium-quality	83.45	0	unclassified	unclassified
v185b	48356	64	21	0	High-quality	100	0	unclassified	unclassified

v185c	38627	42	6	1	High-quality	95.62	0	unclassified	unclassified
v185d	83692	120	18	3	Complete	100	0	unclassified	unclassified
v185e	49981	67	33	1	High-quality	100	0	Myoviridae	prokaryote
v185f	46907	73	27	0	Medium-quality	72.61	0	Myoviridae	prokaryote
v1860	16528	18	6	0	Medium-quality	55.63	0	unclassified	unclassified
v1861	216825	345	39	6	Complete	100	0	unclassified	unclassified
v1862	35923	53	5	1	Medium-quality	53.39	0	unclassified	unclassified
v1863	16752	25	8	0	Complete	100	0	Siphoviridae	prokaryote
v1864	102018	124	14	3	High-quality	100	0	Flandersviridae	prokaryote
v1865	48779	78	23	1	High-quality	100	0	Siphoviridae	prokaryote
v1866	58547	80	11	2	High-quality	100	0	unclassified	unclassified
v1867	16071	20	7	0	Medium-quality	50.45	0	unclassified	unclassified
v1868	78743	123	20	4	Complete	100	0	unclassified	unclassified
v1869	20115	16	2	1	Medium-quality	51.74	0	unclassified	unclassified
v186a	43965	69	8	0	Complete	100	0	unclassified	unclassified
v186b	45319	45	11	0	Complete	100	0	unclassified	unclassified
v186c	93371	116	35	0	Medium-quality	68.39	0	Siphoviridae	prokaryote
v186d	44939	71	15	1	High-quality	99.92	0	unclassified	unclassified
v186e	36127	58	17	0	Medium-quality	80.11	0	unclassified	unclassified
v186f	41458	80	12	0	Complete	100	0	unclassified	unclassified
v1870	45017	75	19	0	Complete	100	0	unclassified	unclassified
v1871	37461	53	13	2	High-quality	99.64	0	unclassified	unclassified
v1872	41783	77	18	0	Complete	100	0	unclassified	unclassified
v1873	52618	66	7	1	Medium-quality	67.16	0	Quimbyviridae	prokaryote
v1874	31692	53	10	1	High-quality	95.45	0	unclassified	unclassified
v1875	42873	62	25	1	High-quality	100	0	unclassified	unclassified
v1876	45249	83	21	1	High-quality	100	0	unclassified	unclassified
v1877	39058	52	17	0	Medium-quality	88.14	0	unclassified	unclassified
v1878	36237	54	14	1	Complete	100	0	unclassified	unclassified
v1879	59197	101	15	2	Complete	100	0	unclassified	unclassified
v187a	39327	66	19	1	Complete	100	0	unclassified	unclassified
v187b	36563	63	17	3	Complete	100	0	Myoviridae	prokaryote
v187c	42249	72	19	0	Complete	100	0	unclassified	unclassified
v187d	46970	72	23	1	High-quality	100	0	Siphoviridae	prokaryote
v187e	5824	8	4	0	Complete	100	0	unclassified	unclassified
v187f	44295	63	9	0	Complete	100	0	unclassified	unclassified
v1880	67659	107	29	4	Complete	100	0	unclassified	unclassified
v1881	10455	13	5	0	Medium-quality	75.28	0	unclassified	unclassified
v1882	41077	68	21	0	High-quality	98.75	0	Siphoviridae	prokaryote
v1883	29427	42	14	0	Medium-quality	75.59	0	Siphoviridae	prokaryote

v1884	27501	46	10	2	Medium-quality	71.66	0	Siphoviridae	prokaryote
v1885	29999	32	11	0	Medium-quality	72.64	0	unclassified	unclassified
v1886	54837	93	15	1	Complete	100	0	unclassified	unclassified
v1887	37477	54	15	0	Complete	100	0	Siphoviridae	prokaryote
v1888	17155	21	4	0	Medium-quality	80.86	0	unclassified	unclassified
v1889	27025	27	26	0	Medium-quality	67.2	0	Autographiviridae	prokaryote
v188a	79922	111	10	2	Medium-quality	73.34	0	unclassified	unclassified
v188b	5438	8	1	0	High-quality	100	0	unclassified	unclassified
v188c	156605	196	23	2	Complete	100	0	unclassified	unclassified
v188d	44166	70	18	0	High-quality	98.25	0	unclassified	unclassified
v188e	52026	92	11	1	High-quality	90.3	0	unclassified	unclassified
v188f	71216	106	15	3	High-quality	97.19	0	unclassified	unclassified
v1890	44455	87	18	1	Complete	100	0	unclassified	unclassified
v1891	45106	78	20	0	High-quality	100	0	unclassified	unclassified
v1892	21379	31	23	0	Medium-quality	53.73	0	Myoviridae	prokaryote
v1893	157087	206	18	3	Complete	100	0	unclassified	unclassified
v1894	15968	19	5	0	Medium-quality	53.15	0	unclassified	unclassified
v1895	27482	31	9	2	Medium-quality	56.17	0	Siphoviridae	prokaryote
v1896	52471	69	16	0	High-quality	100	0	unclassified	unclassified
v1897	47701	43	6	1	Medium-quality	70.9	0	unclassified	unclassified
v1898	40965	68	18	0	Complete	100	0	unclassified	unclassified
v1899	52835	96	32	1	Complete	100	0	Siphoviridae	prokaryote
v189a	41021	58	6	1	High-quality	99.16	0	unclassified	unclassified
v189b	37747	58	49	0	High-quality	93.4	0	Podoviridae	prokaryote
v189c	20678	28	6	1	Low-quality	42.07	0	unclassified	unclassified
v189d	73707	107	12	8	High-quality	100	0	unclassified	unclassified
v189e	60168	71	20	6	High-quality	100	0	Siphoviridae	prokaryote
v189f	44475	68	16	1	High-quality	100	0	Siphoviridae	prokaryote
v18a0	74227	113	12	3	Medium-quality	84.32	0	Quimbyviridae	prokaryote
v18a1	46980	68	18	0	Complete	100	0	Siphoviridae	prokaryote
v18a2	59830	84	12	0	Complete	100	0	unclassified	unclassified
v18a3	40032	51	18	1	Medium-quality	78.62	0	unclassified	unclassified
v18a4	41068	70	20	0	High-quality	98.82	0	unclassified	unclassified
v18a5	39855	60	24	0	High-quality	99.19	0	Siphoviridae	prokaryote
v18a6	42379	73	17	0	Complete	100	0	unclassified	unclassified
v18a7	21214	25	20	0	Medium-quality	53.78	0	Podoviridae	prokaryote
v18a8	16851	23	6	0	Medium-quality	82.06	0	unclassified	unclassified
v18a9	57237	98	20	1	Complete	100	0	unclassified	unclassified
v18aa	86804	117	14	2	Complete	100	0	Flandersviridae	prokaryote
v18ab	39582	63	35	0	Complete	100	0	Siphoviridae	prokaryote

v18ac	42628	63	16	4	Medium-quality	55.66	0	unclassified	unclassified
v18ad	41122	60	15	0	Complete	100	0	unclassified	unclassified
v18ae	96025	138	23	1	High-quality	100	0	unclassified	unclassified
v18af	40263	51	48	0	Complete	100	0	Autographiviridae	prokaryote
v18b0	124960	194	29	2	Medium-quality	70.86	0	unclassified	unclassified
v18b1	59371	81	16	0	Complete	100	0	unclassified	unclassified
v18b2	78422	131	16	3	Complete	100	0	unclassified	unclassified
v18b3	6541	9	4	0	Complete	100	0	Microviridae	prokaryote
v18b4	16148	20	9	0	Medium-quality	70.62	0	Salasmaviridae	prokaryote
v18b5	9770	11	5	0	Medium-quality	52.96	0	unclassified	unclassified
v18b6	71141	109	18	1	High-quality	100	0	unclassified	unclassified
v18b7	15299	20	5	0	Medium-quality	50.92	0	unclassified	unclassified
v18b8	40294	47	46	0	Complete	100	0	Autographiviridae	prokaryote
v18b9	24125	37	11	0	Medium-quality	55.35	0	unclassified	unclassified
v18ba	171620	287	38	3	Complete	100	0	unclassified	unclassified
v18bb	30242	44	14	0	Medium-quality	80.25	0	unclassified	unclassified
v18bc	24074	34	3	2	Medium-quality	55.17	0	unclassified	unclassified
v18bd	35262	60	8	3	Medium-quality	53.28	0	Quimbyviridae	prokaryote
v18be	16158	21	5	0	Medium-quality	53.78	0	unclassified	unclassified
v18bf	41505	68	18	1	Complete	100	0	Siphoviridae	prokaryote
v18c0	45225	67	17	0	High-quality	100	0	unclassified	unclassified
v18c1	42319	73	19	1	High-quality	93.9	0	unclassified	unclassified
v18c2	44103	60	7	1	Complete	100	0	unclassified	unclassified
v18c3	54125	85	22	0	High-quality	99.75	0	Myoviridae	prokaryote
v18c4	25079	41	6	2	Medium-quality	59.25	0	unclassified	unclassified
v18c5	28641	27	4	0	Medium-quality	66.68	0	unclassified	unclassified
v18c6	43146	44	4	2	High-quality	100	0	unclassified	unclassified
v18c7	37008	44	13	0	Medium-quality	74.42	0	Siphoviridae	prokaryote
v18c8	59321	95	14	2	High-quality	100	0	unclassified	unclassified
v18c9	93923	102	14	0	Complete	100	0	Podoviridae_crAss-like	prokaryote
v18ca	5523	10	6	0	High-quality	100	0	Microviridae	prokaryote
v18cb	66610	122	18	2	High-quality	100	0	unclassified	unclassified
v18cc	42490	70	13	1	High-quality	100	0	unclassified	unclassified
v18cd	41284	66	21	2	High-quality	100	0	Siphoviridae	prokaryote
v18ce	44249	64	29	1	High-quality	91.68	0	unclassified	unclassified
v18cf	13814	18	5	0	High-quality	100	0	unclassified	unclassified
v18d0	31993	46	8	1	Medium-quality	71.38	0	unclassified	unclassified
v18d1	22207	32	19	2	Medium-quality	64.3	0	Siphoviridae	prokaryote
v18d2	32689	55	19	1	Complete	100	0	Siphoviridae	prokaryote
v18d3	39594	45	20	1	High-quality	97.36	0	Siphoviridae	prokaryote

v18d4	25295	28	17	1	Medium-quality	62.76	0	Siphoviridae	prokaryote
v18d5	38829	55	17	1	High-quality	99.97	0	Siphoviridae	prokaryote
v18d6	43030	75	21	0	Complete	100	0	unclassified	unclassified
v18d7	37973	55	11	2	Complete	100	0	Siphoviridae	prokaryote
v18d8	52878	90	14	1	High-quality	92.48	0	unclassified	unclassified
v18d9	54324	58	12	7	High-quality	100	0	Siphoviridae	prokaryote
v18da	16539	20	5	0	Medium-quality	55.06	0	unclassified	unclassified
v18db	42899	80	17	0	Complete	100	0	unclassified	unclassified
v18dc	28616	43	20	1	Medium-quality	85.84	0	Myoviridae	prokaryote
v18dd	41821	69	10	2	Complete	100	0	unclassified	unclassified
v18de	34813	58	18	0	Complete	100	0	unclassified	unclassified
v18df	24269	33	7	0	Medium-quality	52.56	0	unclassified	unclassified
v18e0	35325	48	14	0	Medium-quality	89.89	0	Siphoviridae	prokaryote
v18e1	37184	57	15	0	Complete	100	0	Siphoviridae	prokaryote
v18e2	16737	21	8	0	High-quality	90.81	0	unclassified	unclassified
v18e3	58929	70	31	5	High-quality	100	0	Siphoviridae	prokaryote
v18e4	103217	177	19	2	Complete	100	0	Podoviridae_crAss-like	prokaryote
v18e5	39571	60	18	1	High-quality	100	0	Siphoviridae	prokaryote
v18e6	27824	26	10	1	Medium-quality	59.81	0	Siphoviridae	prokaryote
v18e7	66153	105	25	1	High-quality	99.27	0	unclassified	unclassified
v18e8	50128	75	15	3	Medium-quality	68.03	0	unclassified	unclassified
v18e9	27042	26	14	2	Medium-quality	50.52	0	Siphoviridae	prokaryote
v18ea	91234	93	17	13	High-quality	100	0	unclassified	unclassified
v18eb	46025	69	49	1	High-quality	100	0	Siphoviridae	prokaryote
v18ec	25217	36	11	3	Medium-quality	61.84	0	Siphoviridae	prokaryote
v18ed	43040	70	19	0	High-quality	100	0	Siphoviridae	prokaryote
v18ee	24272	41	7	1	Medium-quality	57.88	0	unclassified	unclassified
v18ef	44456	79	19	0	High-quality	100	0	unclassified	unclassified
v18f0	53659	78	8	3	High-quality	100	0	unclassified	unclassified
v18f1	107134	132	12	2	Complete	100	0	unclassified	unclassified
v18f2	8514	13	1	0	High-quality	100	0	Inoviridae	prokaryote
v18f3	56531	70	13	2	High-quality	100	0	unclassified	unclassified
v18f4	44570	58	38	2	High-quality	100	0	Siphoviridae	prokaryote
v18f5	46637	60	20	5	High-quality	97.63	0	Siphoviridae	prokaryote
v18f6	24859	21	9	1	Medium-quality	60.16	0	unclassified	unclassified
v18f7	138360	201	32	6	Complete	100	0	unclassified	unclassified
v18f8	118609	234	24	7	Medium-quality	57.73	0	unclassified	unclassified
v18f9	37655	63	14	0	Medium-quality	83.71	0	unclassified	unclassified
v18fa	45876	42	4	1	Medium-quality	68.21	0	unclassified	unclassified
v18fb	35738	60	11	0	High-quality	93.16	0	unclassified	unclassified

v18fc	41116	69	18	2	High-quality	100	0	unclassified	unclassified
v18fd	56951	82	33	0	Complete	100	0	Siphoviridae	prokaryote
v18fe	37030	48	16	0	High-quality	100	0	Siphoviridae	prokaryote
v18ff	45215	71	6	4	Medium-quality	60.51	0	unclassified	unclassified
v1900	153541	186	23	4	Complete	100	0	unclassified	unclassified
v1901	45538	79	19	1	Complete	100	0	unclassified	unclassified
v1902	28588	33	10	1	Medium-quality	52.38	0	Siphoviridae	prokaryote
v1903	31853	41	22	0	Medium-quality	52.95	0	Siphoviridae	prokaryote
v1904	59939	61	15	6	High-quality	100	0	Siphoviridae	prokaryote
v1905	11227	9	1	0	High-quality	94	0	unclassified	unclassified
v1906	35419	44	6	3	Medium-quality	75.24	0	unclassified	unclassified
v1907	68351	86	15	7	High-quality	97.64	0	unclassified	unclassified
v1908	37386	56	7	1	Medium-quality	60.97	0	unclassified	unclassified
v1909	60580	101	19	4	Medium-quality	75.15	0	unclassified	unclassified
v190a	44252	78	15	0	Complete	100	0	unclassified	unclassified
v190b	32994	50	28	0	Complete	100	0	Siphoviridae	prokaryote
v190c	68534	102	9	4	High-quality	100	0	unclassified	unclassified
v190d	21041	34	12	1	Medium-quality	55.11	0	unclassified	unclassified
v190e	37172	56	14	1	High-quality	94.3	0	unclassified	unclassified
v190f	81341	108	33	5	High-quality	100	0	Siphoviridae	prokaryote
v1910	20306	24	4	3	Medium-quality	70.78	0	unclassified	unclassified
v1911	25647	37	15	0	Medium-quality	64.04	0	Siphoviridae	prokaryote
v1912	45993	71	19	2	High-quality	100	0	unclassified	unclassified
v1913	34766	39	13	0	Medium-quality	72.04	0	Siphoviridae	prokaryote
v1914	5185	9	4	0	Complete	100	0	Microviridae	prokaryote
v1915	59936	83	53	0	High-quality	100	0	Siphoviridae	prokaryote
v1916	50171	60	20	5	High-quality	92.82	0	Siphoviridae	prokaryote
v1917	41627	64	13	0	Complete	100	0	unclassified	unclassified
v1918	19014	22	7	0	Medium-quality	52.23	0	unclassified	unclassified
v1919	52209	78	22	1	High-quality	100	0	unclassified	unclassified
v191a	46579	63	12	5	High-quality	100	0	unclassified	unclassified
v191b	62647	88	20	4	High-quality	100	0	Myoviridae	prokaryote
v191c	56654	74	11	6	High-quality	100	0	unclassified	unclassified
v191d	26289	44	25	0	Medium-quality	66.29	0	Siphoviridae	prokaryote
v191e	34684	48	35	1	Medium-quality	67.25	0	Siphoviridae	prokaryote
v191f	57689	73	23	3	High-quality	100	0	Siphoviridae	prokaryote
v1920	80891	109	13	1	Complete	100	0	Flandersviridae	prokaryote
v1921	42135	71	17	0	Complete	100	0	unclassified	unclassified
v1922	32646	52	16	2	Medium-quality	88.28	0	Siphoviridae	prokaryote
v1923	122866	179	28	2	Complete	100	0	unclassified	unclassified

v1924	33574	35	10	1	Medium-quality	65.53	0	Siphoviridae	prokaryote
v1925	69122	76	17	8	High-quality	100	0	unclassified	unclassified
v1926	15635	20	6	0	Medium-quality	52.62	0	unclassified	unclassified
v1927	235281	383	43	9	High-quality	100	0	unclassified	unclassified
v1928	60297	98	26	0	High-quality	100	0	Myoviridae	prokaryote
v1929	5176	9	6	0	Complete	100	0	Microviridae	prokaryote
v192a	119944	150	11	8	High-quality	100	0	unclassified	unclassified
v192b	35712	63	16	0	Medium-quality	77.24	0	unclassified	unclassified
v192c	75858	123	19	4	Complete	100	0	unclassified	unclassified
v192d	55803	93	16	1	Complete	100	0	unclassified	unclassified
v192e	206266	311	47	11	High-quality	100	0	unclassified	unclassified
v192f	49107	65	11	1	Complete	100	0	unclassified	unclassified
v1930	21688	32	8	0	Medium-quality	54.3	0	Siphoviridae	prokaryote
v1931	16036	17	5	0	Medium-quality	53.38	0	unclassified	unclassified
v1932	40355	60	30	2	High-quality	91.02	0	Siphoviridae	prokaryote
v1933	57997	97	24	3	Complete	100	0	unclassified	unclassified
v1934	15613	24	6	0	Complete	100	0	unclassified	unclassified
v1935	82514	132	17	2	High-quality	94.02	0	Quimbyviridae	prokaryote
v1936	38691	55	20	1	Complete	100	0	unclassified	unclassified
v1937	42988	60	21	1	High-quality	100	0	Siphoviridae	prokaryote
v1938	45691	77	26	0	High-quality	100	0	Myoviridae	prokaryote
v1939	40704	63	12	0	Complete	100	0	unclassified	unclassified
v193a	50189	65	16	4	High-quality	100	0	Myoviridae	prokaryote
v193b	83102	104	25	0	Low-quality	49.39	0	unclassified	unclassified
v193c	48343	63	42	2	High-quality	100	0	Siphoviridae	prokaryote
v193d	211925	317	46	9	High-quality	100	0	unclassified	unclassified
v193e	46563	60	8	1	Complete	100	0	Siphoviridae	prokaryote
v193f	5906	7	3	0	Complete	100	0	unclassified	unclassified
v1940	21230	31	10	0	Medium-quality	57.75	0	unclassified	unclassified
v1941	101431	122	7	2	High-quality	97.35	0	unclassified	unclassified
v1942	157750	207	18	5	High-quality	100	0	unclassified	unclassified
v1943	27987	50	6	1	Medium-quality	62.36	0	Myoviridae	prokaryote
v1944	33710	58	26	0	Medium-quality	73.25	0	Myoviridae	prokaryote
v1945	23296	27	4	1	Medium-quality	51.96	0	unclassified	unclassified
v1946	30943	31	9	2	Medium-quality	65.33	0	Siphoviridae	prokaryote
v1947	42492	75	20	0	Complete	100	0	unclassified	unclassified
v1948	12844	18	4	0	Complete	100	0	unclassified	unclassified
v1949	44313	60	7	2	High-quality	100	0	unclassified	unclassified
v194a	13464	16	2	1	High-quality	100	0	unclassified	unclassified
v194b	54124	53	1	13	Medium-quality	64.45	0	unclassified	unclassified

v194c	40855	29	2	4	Medium-quality	54.71	0	unclassified	unclassified
v194d	58884	81	1	24	Medium-quality	66.1	0	unclassified	unclassified
v194e	35808	52	33	0	Medium-quality	88.14	0	Siphoviridae	prokaryote
v194f	21776	32	3	1	High-quality	100	0	unclassified	unclassified
v1950	143813	212	26	2	High-quality	100	0	unclassified	unclassified
v1951	54926	81	11	3	High-quality	91.36	0	unclassified	unclassified
v1952	46893	68	32	1	High-quality	100	0	Siphoviridae	prokaryote
v1953	33923	54	24	0	High-quality	98.94	0	Siphoviridae	prokaryote
v1954	72874	110	21	5	High-quality	100	0	unclassified	unclassified
v1955	63594	91	35	1	High-quality	100	0	Siphoviridae	prokaryote
v1956	40789	46	3	1	High-quality	98.19	0	unclassified	unclassified
v1957	30829	42	26	0	Medium-quality	87.6	0	Siphoviridae	prokaryote
v1958	41291	71	29	0	Medium-quality	89.05	0	Myoviridae	prokaryote
v1959	77571	127	28	2	High-quality	96.2	0	unclassified	unclassified
v195a	39312	61	44	0	Medium-quality	87.03	0	Siphoviridae	prokaryote
v195b	46045	66	28	2	High-quality	97.95	0	Siphoviridae	prokaryote
v195c	29132	52	7	0	Medium-quality	55.29	0	Myoviridae	prokaryote
v195d	30936	36	6	5	Medium-quality	67.15	0	Siphoviridae	prokaryote
v195e	56178	82	15	3	High-quality	100	0	unclassified	unclassified
v195f	36826	59	43	0	High-quality	93.41	0	Podoviridae	prokaryote
v1960	107420	136	29	9	High-quality	100	0	Myoviridae	prokaryote
v1961	41925	51	30	1	Medium-quality	85.66	0	Myoviridae	prokaryote
v1962	23081	33	26	0	Medium-quality	58.59	0	Siphoviridae	prokaryote
v1963	16964	22	5	1	Medium-quality	54.47	0	unclassified	unclassified
v1964	8403	16	5	1	Medium-quality	75.43	0	unclassified	unclassified
v1965	79418	135	18	5	High-quality	100	0	unclassified	unclassified
v1966	38176	51	8	2	High-quality	94.17	0	unclassified	unclassified
v1967	32353	35	16	1	Medium-quality	77.07	0	Autographiviridae	prokaryote
v1968	229623	316	68	21	High-quality	100	17.3	Siphoviridae	prokaryote
v1969	33602	38	26	0	Medium-quality	78.88	0	Myoviridae	prokaryote
v196a	41605	67	21	0	High-quality	100	0	Siphoviridae	prokaryote
v196b	25343	34	14	0	Medium-quality	69.03	0	unclassified	unclassified
v196c	8525	10	3	2	High-quality	100	31.78	Microviridae	prokaryote
v196d	35621	50	14	0	High-quality	100	0	unclassified	unclassified
v196e	109553	142	19	12	High-quality	100	0	unclassified	unclassified
v196f	31648	50	16	1	High-quality	90.79	0	unclassified	unclassified
v1970	81952	112	23	1	High-quality	100	0	unclassified	unclassified
v1971	106712	144	31	7	High-quality	100	0	unclassified	unclassified
v1972	41080	68	15	0	High-quality	97.58	0	unclassified	unclassified
v1973	149527	207	30	6	High-quality	93.32	0	unclassified	unclassified

v1974	5075	5	1	0	High-quality	100	0	unclassified	unclassified
v1975	46887	72	13	1	High-quality	100	0	unclassified	unclassified
v1976	125187	125	8	23	High-quality	97.12	62.45	unclassified	unclassified
v1977	28107	41	28	0	Medium-quality	86.45	0	Siphoviridae	prokaryote
v1978	40240	68	23	2	High-quality	100	0	Siphoviridae	prokaryote
v1979	28628	46	21	0	Medium-quality	79.71	0	Siphoviridae	prokaryote
v197a	48181	64	42	2	High-quality	100	0	Siphoviridae	prokaryote
v197b	30217	36	1	11	Medium-quality	76.36	0	unclassified	unclassified
v197c	36116	37	0	7	Medium-quality	71.85	0	unclassified	unclassified
v197d	37873	40	1	2	Medium-quality	57.31	0	unclassified	unclassified
v197e	300473	328	8	85	High-quality	96.78	0	unclassified	unclassified
v197f	27156	28	1	8	Medium-quality	81.31	0	unclassified	unclassified
v1980	14640	23	1	1	Medium-quality	63.93	0	unclassified	unclassified
v1981	50869	47	6	6	Medium-quality	59.59	0	unclassified	unclassified
v1982	8376	14	0	0	High-quality	100	0	Inoviridae	prokaryote
v1983	220579	229	16	20	High-quality	97.16	0	unclassified	unclassified
v1984	15350	18	1	4	High-quality	100	0	unclassified	unclassified
v1985	43427	38	1	11	High-quality	100	0	unclassified	unclassified
v1986	50069	89	21	1	High-quality	100	0	unclassified	unclassified
v1987	71834	105	19	5	High-quality	100	0	Myoviridae	prokaryote
v1988	68992	65	5	8	High-quality	100	0	unclassified	unclassified
v1989	30954	45	19	1	Medium-quality	64.56	0	Siphoviridae	prokaryote
v198a	43871	57	22	0	High-quality	100	0	Myoviridae	prokaryote
v198b	26876	32	5	0	Medium-quality	63.84	0	unclassified	unclassified
v198c	42654	70	17	0	High-quality	94.6	0	unclassified	unclassified
v198d	48072	65	30	2	High-quality	100	0	Myoviridae	prokaryote
v198e	35544	52	19	0	High-quality	94.15	0	Siphoviridae	prokaryote
v198f	22097	33	19	1	Medium-quality	65.12	0	Siphoviridae	prokaryote
v1990	24190	28	1	6	High-quality	100	0	unclassified	unclassified
v1991	52523	79	18	0	High-quality	92.43	0	unclassified	unclassified
v1992	21276	30	13	1	Medium-quality	55.39	0	unclassified	unclassified
v1993	35479	54	32	0	High-quality	100	0	Myoviridae	prokaryote
v1994	49848	49	8	3	Medium-quality	64.13	0	unclassified	unclassified
v1995	34853	42	19	0	High-quality	90.16	0	Siphoviridae	prokaryote
v1996	41702	49	20	0	High-quality	100	0	Siphoviridae	prokaryote
v1997	60388	76	12	8	High-quality	100	0	unclassified	unclassified
v1998	44995	35	0	10	Medium-quality	54.53	0	unclassified	unclassified
v1999	43129	65	22	2	Medium-quality	61.44	0	Siphoviridae	prokaryote
v199a	47220	76	19	0	High-quality	100	0	unclassified	unclassified
v199b	82151	98	13	4	High-quality	100	0	unclassified	unclassified

v199c	34812	51	13	4	Medium-quality	69.98	15.39	unclassified	unclassified
v199d	47580	67	16	2	High-quality	100	0	unclassified	unclassified
v199e	50304	80	33	0	High-quality	100	0	Myoviridae	prokaryote
v199f	7758	11	0	2	Medium-quality	65.31	0	unclassified	unclassified
v19a0	133112	198	24	1	High-quality	99.6	0	unclassified	unclassified
v19a1	52768	76	21	2	High-quality	100	0	unclassified	unclassified
v19a2	39018	66	22	0	High-quality	98	0	Siphoviridae	prokaryote
v19a3	52534	74	16	0	Medium-quality	65.31	0	unclassified	unclassified
v19a4	30989	36	7	1	Medium-quality	76.44	0	unclassified	unclassified
v19a5	62336	91	32	3	High-quality	100	0	Siphoviridae	prokaryote
v19a6	31362	58	22	0	Medium-quality	79.39	0	Siphoviridae	prokaryote
v19a7	63625	102	28	0	High-quality	100	0	unclassified	unclassified
v19a8	41776	69	31	0	High-quality	100	0	Siphoviridae	prokaryote
v19a9	44718	76	49	0	High-quality	95.35	0	Siphoviridae	prokaryote
v19aa	34652	51	21	2	High-quality	100	0	Siphoviridae	prokaryote
v19ab	36171	63	52	0	Medium-quality	68.74	0	Siphoviridae	prokaryote
v19ac	43959	64	19	0	High-quality	100	0	Siphoviridae	prokaryote
v19ad	18880	27	12	1	Medium-quality	51.7	0	Siphoviridae	prokaryote
v19ae	74642	76	1	25	High-quality	100	0	unclassified	unclassified
v19af	28313	29	3	8	Medium-quality	61.9	0	unclassified	unclassified
v19b0	114929	173	31	2	Medium-quality	64.5	0	unclassified	unclassified
v19b1	219459	321	48	19	High-quality	100	0	unclassified	unclassified
v19b2	29668	33	6	3	High-quality	100	0	unclassified	unclassified
v19b3	61148	91	7	2	Medium-quality	73.07	0	Quimbyviridae	prokaryote
v19b4	68054	84	25	2	High-quality	100	0	Siphoviridae	prokaryote
v19b5	75632	100	14	7	Medium-quality	86.97	0	unclassified	unclassified
v19b6	41980	55	14	2	High-quality	100	0	Siphoviridae	prokaryote
v19b7	39419	47	4	2	High-quality	100	0	unclassified	unclassified
v19b8	134402	174	27	5	High-quality	94.69	0	unclassified	unclassified
v19b9	76016	92	24	1	High-quality	100	0	Siphoviridae	prokaryote
v19ba	39788	56	19	0	High-quality	100	0	unclassified	unclassified
v19bb	54499	86	28	0	High-quality	100	0	Siphoviridae	prokaryote
v19bc	50903	70	34	0	High-quality	100	0	Myoviridae	prokaryote
v19bd	40569	59	14	1	High-quality	100	0	unclassified	unclassified
v19be	123258	133	5	43	High-quality	93.99	0	unclassified	unclassified
v19bf	37039	54	33	0	Medium-quality	82.29	0	Siphoviridae	prokaryote
v19c0	79753	107	13	2	High-quality	100	0	unclassified	unclassified
v19c1	51092	58	6	1	High-quality	100	0	unclassified	unclassified
v19c2	41186	60	26	0	High-quality	100	0	Siphoviridae	prokaryote
v19c3	40012	60	22	0	High-quality	100	0	Siphoviridae	prokaryote

v19c4	54566	86	21	0	Medium-quality	87.75	0	unclassified	unclassified
v19c5	36277	30	1	4	Medium-quality	53.77	0	unclassified	unclassified
v19c6	70514	66	2	22	Medium-quality	51.13	0	unclassified	unclassified
v19c7	127382	137	12	20	High-quality	100	0	unclassified	unclassified
v19c8	35892	58	23	0	Medium-quality	89.95	0	Siphoviridae	prokaryote
v19c9	34934	57	23	4	Medium-quality	84.08	0	Siphoviridae	prokaryote
v19ca	41553	48	19	2	High-quality	93.69	7.3	Siphoviridae	prokaryote
v19cb	33987	42	17	1	Medium-quality	75.78	0	Autographiviridae	prokaryote
v19cc	37889	67	29	0	Medium-quality	54.37	0	Myoviridae	prokaryote
v19cd	47954	63	28	2	Medium-quality	89.82	0	Siphoviridae	prokaryote
v19ce	53477	72	14	4	High-quality	100	0	Siphoviridae	prokaryote
v19cf	35869	57	17	0	Medium-quality	84.24	0	Siphoviridae	prokaryote
v19d0	45965	65	9	0	High-quality	100	0	unclassified	unclassified
v19d1	73085	88	22	8	High-quality	100	0	unclassified	unclassified
v19d2	28378	34	26	0	Medium-quality	70.38	0	Podoviridae	prokaryote
v19d3	33587	52	20	0	Medium-quality	89.7	0	Siphoviridae	prokaryote
v19d4	85140	130	81	4	High-quality	100	0	Siphoviridae	prokaryote
v19d5	63352	85	18	3	High-quality	100	0	Myoviridae	prokaryote
v19d6	63995	67	1	20	Medium-quality	73.84	0	unclassified	unclassified
v19d7	49935	62	39	2	High-quality	96	11.54	Siphoviridae	prokaryote
v19d8	26038	37	24	0	Medium-quality	66.35	0	Siphoviridae	prokaryote
v19d9	31122	47	19	1	Medium-quality	67.96	0	Siphoviridae	prokaryote
v19da	72880	78	11	8	Medium-quality	81.97	0	unclassified	unclassified
v19db	38849	51	13	0	High-quality	95.51	0	unclassified	unclassified
v19dc	62185	66	12	0	High-quality	97.83	0	unclassified	unclassified
v19dd	39881	54	19	1	High-quality	97.08	0	Siphoviridae	prokaryote
v19de	37001	52	28	0	High-quality	95.35	0	Siphoviridae	prokaryote
v19df	37415	68	25	0	Medium-quality	86.53	0	unclassified	unclassified
v19e0	41272	64	26	0	High-quality	100	0	Siphoviridae	prokaryote
v19e1	78680	95	37	4	High-quality	100	0	Siphoviridae	prokaryote
v19e2	53338	81	28	0	High-quality	100	0	unclassified	unclassified
v19e3	100911	80	7	17	High-quality	100	0	unclassified	unclassified
v19e4	47494	60	5	12	Medium-quality	82.04	0	unclassified	unclassified
v19e5	190239	224	16	32	High-quality	100	0	unclassified	unclassified
v19e6	39484	52	19	0	High-quality	100	0	Siphoviridae	prokaryote
v19e7	25475	26	2	0	Medium-quality	56.98	0	unclassified	unclassified
v19e8	17793	20	5	3	Medium-quality	52.22	15.79	unclassified	unclassified
v19e9	35405	54	29	0	Medium-quality	79	0	Siphoviridae	prokaryote
v19ea	42702	70	15	0	Medium-quality	72.26	0	unclassified	unclassified
v19eb	113774	115	5	32	High-quality	100	0	unclassified	unclassified

v19ec	58769	73	2	3	High-quality	100	0	unclassified	unclassified
v19ed	56745	52	1	17	High-quality	100	0	unclassified	unclassified
v19ee	131728	134	13	22	High-quality	100	0	unclassified	unclassified
v19ef	109334	119	7	17	High-quality	100	0	unclassified	unclassified
v19f0	86606	94	3	26	Medium-quality	63.58	0	unclassified	unclassified
v19f1	66057	74	2	10	Medium-quality	66.77	0	unclassified	unclassified
v19f2	27112	39	3	5	Medium-quality	56.01	0	unclassified	unclassified
v19f3	80449	83	3	14	High-quality	96.06	0	unclassified	unclassified
v19f4	93363	102	1	30	Medium-quality	56.82	0	unclassified	unclassified
v19f5	41181	59	2	2	Medium-quality	74.71	0	unclassified	unclassified
v19f6	44521	48	1	13	High-quality	100	0	unclassified	unclassified
v19f7	89720	89	5	13	High-quality	100	0	unclassified	unclassified
v19f8	59994	66	3	18	High-quality	100	0	unclassified	unclassified
v19f9	118246	133	14	34	High-quality	100	0	unclassified	unclassified
v19fa	59629	60	1	16	Medium-quality	85.63	0	unclassified	unclassified
v19fb	29817	23	1	6	Medium-quality	66.9	0	unclassified	unclassified
v19fc	22021	27	1	6	Medium-quality	54.87	0	unclassified	unclassified
v19fd	9621	12	1	1	High-quality	100	0	Myoviridae	prokaryote
v19fe	107624	85	2	29	High-quality	100	0	unclassified	unclassified
v19ff	30552	40	4	4	Medium-quality	63.12	0	unclassified	unclassified
v1a00	37027	37	4	0	High-quality	97.14	0	Siphoviridae	prokaryote
v1a01	42298	53	15	1	High-quality	97.9	0	Siphoviridae	prokaryote
v1a02	43111	52	5	3	Medium-quality	70.89	0	unclassified	unclassified
v1a03	34739	55	30	0	Medium-quality	85.67	0	Siphoviridae	prokaryote
v1a04	39185	50	9	2	Medium-quality	63.91	0	unclassified	unclassified
v1a05	34180	47	25	0	High-quality	99.17	0	Siphoviridae	prokaryote
v1a06	36179	54	17	1	Medium-quality	85.81	0	unclassified	unclassified
v1a07	38564	70	32	0	Medium-quality	82.55	0	Myoviridae	prokaryote
v1a08	32326	34	9	1	High-quality	100	0	unclassified	unclassified
v1a09	75400	67	6	3	High-quality	100	0	unclassified	unclassified
v1a0a	38606	59	10	0	Medium-quality	65.72	0	unclassified	unclassified
v1a0b	42902	53	37	0	High-quality	100	0	Podoviridae	prokaryote
v1a0c	38700	50	5	1	High-quality	93.36	0	unclassified	unclassified
v1a0d	43560	80	19	1	High-quality	99.96	0	unclassified	unclassified
v1a0e	51506	56	9	2	High-quality	100	0	unclassified	unclassified
v1a0f	49197	73	16	1	High-quality	100	0	unclassified	unclassified
v1a10	38355	63	21	2	High-quality	100	0	Siphoviridae	prokaryote
v1a11	48702	60	33	1	High-quality	100	0	Siphoviridae	prokaryote
v1a12	44003	52	28	1	High-quality	100	0	Siphoviridae	prokaryote
v1a13	40657	62	18	1	High-quality	100	0	Myoviridae	prokaryote

v1a14	32963	47	15	0	High-quality	96.37	0	Siphoviridae	prokaryote
v1a15	39218	47	20	0	High-quality	100	0	Siphoviridae	prokaryote
v1a16	51526	43	6	1	Medium-quality	83.16	0	unclassified	unclassified
v1a17	59499	77	15	2	High-quality	100	0	unclassified	unclassified
v1a18	43330	69	29	0	High-quality	100	0	unclassified	unclassified
v1a19	42632	58	8	2	Medium-quality	71.13	0	unclassified	unclassified
v1a1a	58114	82	15	5	High-quality	100	0	unclassified	unclassified
v1a1b	47210	59	29	1	High-quality	100	0	Siphoviridae	prokaryote
v1a1c	45907	58	4	2	High-quality	100	0	unclassified	unclassified
v1a1d	57171	100	17	1	High-quality	100	0	unclassified	unclassified
v1a1e	46242	55	24	1	High-quality	100	0	Siphoviridae	prokaryote
v1a1f	27391	48	15	0	Medium-quality	82.94	0	Siphoviridae	prokaryote
v1a20	36114	55	23	2	High-quality	98.05	0	Siphoviridae	prokaryote
v1a21	42675	58	21	1	High-quality	100	0	Siphoviridae	prokaryote
v1a22	37713	58	6	0	High-quality	91.17	0	unclassified	unclassified
v1a23	39910	61	42	0	High-quality	100	0	Podoviridae	prokaryote
v1a24	40463	71	23	1	High-quality	97.44	0	Siphoviridae	prokaryote
v1a25	35458	59	18	0	High-quality	100	0	unclassified	unclassified
v1a26	37974	56	21	0	High-quality	100	0	Siphoviridae	prokaryote
v1a27	60321	80	18	7	High-quality	100	0	Myoviridae	prokaryote
v1a28	37915	71	18	1	High-quality	92.71	0	unclassified	unclassified
v1a29	69460	91	15	7	High-quality	100	0	unclassified	unclassified
v1a2a	35800	54	35	0	Medium-quality	76.53	0	Siphoviridae	prokaryote
v1a2b	74900	78	8	8	Medium-quality	66.74	46.08	unclassified	unclassified
v1a2c	163388	208	40	7	High-quality	100	0	unclassified	unclassified
v1a2d	47185	59	23	1	High-quality	98.04	0	Siphoviridae	prokaryote
v1a2e	47773	83	8	0	Medium-quality	75.38	0	Quimbyviridae	prokaryote
v1a2f	48028	56	11	3	High-quality	100	0	unclassified	unclassified
v1a30	27745	31	1	9	Medium-quality	58.94	0	unclassified	unclassified
v1a31	10379	13	1	2	High-quality	100	0	unclassified	unclassified
v1a32	59608	94	17	4	Medium-quality	80.34	0	unclassified	unclassified
v1a33	32572	40	14	1	Medium-quality	82.1	0	Siphoviridae	prokaryote
v1a34	16218	17	3	2	High-quality	100	0	unclassified	unclassified
v1a35	43278	69	28	0	High-quality	92.63	0	unclassified	unclassified
v1a36	43550	79	20	0	High-quality	100	0	Siphoviridae	prokaryote
v1a37	28242	41	13	0	High-quality	100	0	Siphoviridae	prokaryote
v1a38	46678	47	7	2	High-quality	90.27	0	unclassified	unclassified
v1a39	26296	37	15	0	Medium-quality	65.63	0	Siphoviridae	prokaryote
v1a3a	46643	59	9	1	Medium-quality	86.46	0	unclassified	unclassified
v1a3b	37318	51	11	2	High-quality	100	0	unclassified	unclassified

v1a3c	33850	62	21	1	High-quality	91.88	0	unclassified	unclassified
v1a3d	39224	58	18	1	High-quality	100	0	unclassified	unclassified
v1a3e	31829	53	14	0	High-quality	98.84	0	unclassified	unclassified
v1a3f	51419	54	6	1	Medium-quality	76.44	0	unclassified	unclassified
v1a40	23023	22	3	2	Medium-quality	56.18	0	unclassified	unclassified
v1a41	33058	60	20	1	Medium-quality	89.7	0	unclassified	unclassified
v1a42	98756	109	6	14	High-quality	100	0	unclassified	unclassified
v1a43	28045	30	1	3	Medium-quality	62.33	0	unclassified	unclassified
v1a44	39393	42	1	8	High-quality	94.34	0	unclassified	unclassified
v1a45	63349	62	6	6	High-quality	100	0	unclassified	unclassified
v1a46	109093	115	3	37	High-quality	96.72	0	unclassified	unclassified
v1a47	7002	11	1	1	High-quality	100	0	unclassified	unclassified
v1a48	173761	187	6	13	Medium-quality	80.58	0	unclassified	unclassified
v1a49	30781	32	0	4	Medium-quality	61.5	0	unclassified	unclassified
v1a4a	33845	35	9	0	Medium-quality	85.29	0	Siphoviridae	prokaryote
v1a4b	35706	46	23	0	Medium-quality	88.64	0	Siphoviridae	prokaryote
v1a4c	31466	47	17	0	Medium-quality	78.7	0	Siphoviridae	prokaryote
v1a4d	67984	116	23	2	High-quality	100	0	unclassified	unclassified
v1a4e	62340	103	32	1	High-quality	100	0	Myoviridae	prokaryote
v1a4f	37462	61	35	0	Medium-quality	83.35	0	Siphoviridae	prokaryote
v1a50	50446	54	10	7	High-quality	91.61	47.61	unclassified	unclassified
v1a51	38801	62	10	0	High-quality	99.1	0	unclassified	unclassified
v1a52	36382	48	19	1	High-quality	94.76	0	Siphoviridae	prokaryote
v1a53	28645	41	11	1	Medium-quality	68	0	unclassified	unclassified
v1a54	14633	18	3	0	High-quality	100	0	unclassified	unclassified
v1a55	37367	60	33	0	High-quality	95.64	0	Myoviridae	prokaryote
v1a56	39035	51	21	0	High-quality	99.87	0	Siphoviridae	prokaryote
v1a57	30897	41	24	0	Medium-quality	87.63	0	Myoviridae	prokaryote
v1a58	173889	224	41	4	High-quality	100	0	unclassified	unclassified
v1a59	20481	30	7	0	High-quality	100	0	Siphoviridae	prokaryote
v1a5a	50513	71	15	3	High-quality	100	0	Myoviridae	prokaryote
v1a5b	42905	60	28	0	High-quality	96.81	0	Myoviridae	prokaryote
v1a5c	30291	38	20	0	Medium-quality	53.37	0	Siphoviridae	prokaryote
v1a5d	40451	48	18	1	High-quality	97.32	0	Siphoviridae	prokaryote
v1a5e	43511	49	20	1	High-quality	100	0	unclassified	unclassified
v1a5f	91766	161	41	0	High-quality	100	0	unclassified	unclassified
v1a60	32312	45	22	2	Medium-quality	76.67	0	Siphoviridae	prokaryote
v1a61	21995	19	3	2	Medium-quality	53.81	0	unclassified	unclassified
v1a62	112588	145	28	3	Medium-quality	74.77	0	unclassified	unclassified
v1a63	40698	50	6	0	High-quality	100	0	Siphoviridae	prokaryote

v1a64	33667	56	25	0	High-quality	96.12	0	Siphoviridae	prokaryote
v1a65	65174	109	34	0	High-quality	99.84	0	Siphoviridae	prokaryote
v1a66	44134	66	23	0	High-quality	94.66	0	Siphoviridae	prokaryote
v1a67	39412	68	43	0	High-quality	94.43	0	Siphoviridae	prokaryote
v1a68	41321	54	19	0	High-quality	100	0	Siphoviridae	prokaryote
v1a69	86104	113	18	3	Medium-quality	73.91	0	unclassified	unclassified
v1a6a	118962	126	14	42	Medium-quality	86.83	45.18	unclassified	unclassified
v1a6b	53351	72	23	0	High-quality	96.46	0	Myoviridae	prokaryote
v1a6c	48597	70	41	0	High-quality	100	0	Siphoviridae	prokaryote
v1a6d	48181	77	17	0	High-quality	100	0	unclassified	unclassified
v1a6e	29942	51	14	0	Medium-quality	70.33	0	unclassified	unclassified
v1a6f	38331	60	25	0	Medium-quality	82.5	0	unclassified	unclassified
v1a70	6418	9	6	0	High-quality	100	0	Microviridae	prokaryote
v1a71	52342	77	7	5	Medium-quality	76.49	0	unclassified	unclassified
v1a72	60634	57	1	17	Medium-quality	72.2	0	unclassified	unclassified
v1a73	93148	98	3	30	Medium-quality	89.58	0	unclassified	unclassified
v1a74	62473	58	2	18	Medium-quality	64.44	0	unclassified	unclassified
v1a75	33058	47	23	0	Medium-quality	85.83	0	Siphoviridae	prokaryote
v1a76	41790	56	12	1	High-quality	96.78	0	unclassified	unclassified
v1a77	34708	57	17	0	Medium-quality	82.03	0	unclassified	unclassified
v1a78	32504	46	12	0	High-quality	95.37	0	unclassified	unclassified
v1a79	40645	60	13	1	High-quality	98.52	0	unclassified	unclassified
v1a7a	50984	69	43	0	High-quality	100	0	Siphoviridae	prokaryote
v1a7b	42617	52	1	18	Medium-quality	77.25	0	unclassified	unclassified
v1a7c	13122	16	0	0	Medium-quality	80.53	0	unclassified	unclassified
v1a7d	30331	31	3	13	Medium-quality	53.28	0	unclassified	unclassified
v1a7e	77734	83	8	11	High-quality	100	0	unclassified	unclassified
v1a7f	23129	19	1	6	Medium-quality	55.84	0	unclassified	unclassified
v1a80	36351	51	16	0	High-quality	96.17	0	unclassified	unclassified
v1a81	114127	191	26	6	Medium-quality	64.67	0	unclassified	unclassified
v1a82	46027	55	13	0	Medium-quality	75.47	0	unclassified	unclassified
v1a83	59814	66	4	5	High-quality	100	0	unclassified	unclassified
v1a84	22484	23	16	0	Medium-quality	55.94	0	Siphoviridae	prokaryote
v1a85	52206	76	18	7	High-quality	100	0	Siphoviridae	prokaryote
v1a86	168768	272	42	3	High-quality	95.66	0	unclassified	unclassified
v1a87	37993	72	21	1	Medium-quality	62.94	0	Siphoviridae	prokaryote
v1a88	48627	75	28	0	High-quality	97.99	0	Siphoviridae	prokaryote
v1a89	41535	57	32	0	High-quality	100	0	Siphoviridae	prokaryote
v1a8a	36586	61	28	1	Medium-quality	81.99	0	Myoviridae	prokaryote
v1a8b	35592	59	21	0	High-quality	94.16	0	Siphoviridae	prokaryote

v1a8c	44018	59	38	1	High-quality	100	0	Podoviridae	prokaryote
v1a8d	128425	190	64	3	High-quality	100	0	Siphoviridae	prokaryote
v1a8e	39799	65	24	0	High-quality	99.29	0	Siphoviridae	prokaryote
v1a8f	59818	81	16	2	High-quality	100	0	unclassified	unclassified
v1a90	33655	42	21	0	Medium-quality	74.49	0	Siphoviridae	prokaryote
v1a91	60311	89	26	2	High-quality	100	0	Myoviridae	prokaryote
v1a92	42601	49	20	0	Medium-quality	82.29	0	Myoviridae	prokaryote
v1a93	47374	66	15	0	High-quality	100	0	Siphoviridae	prokaryote
v1a94	42169	50	16	2	High-quality	100	8.21	Siphoviridae	prokaryote
v1a95	33054	40	3	0	Medium-quality	73.07	0	unclassified	unclassified
v1a96	35551	53	14	4	High-quality	100	0	unclassified	unclassified
v1a97	124237	147	13	35	High-quality	100	0	unclassified	unclassified
v1a98	115668	137	2	46	High-quality	100	0	unclassified	unclassified
v1a99	94504	120	16	21	High-quality	100	0	unclassified	unclassified
v1a9a	105923	120	6	18	High-quality	91.14	0	unclassified	unclassified
v1a9b	52931	50	1	13	Medium-quality	63.38	0	unclassified	unclassified
v1a9c	111936	112	2	11	High-quality	100	0	unclassified	unclassified
v1a9d	79652	87	6	23	Medium-quality	85.27	0	unclassified	unclassified
v1a9e	96895	87	2	30	High-quality	100	0	unclassified	unclassified
v1a9f	18354	19	1	2	High-quality	100	0	unclassified	unclassified
v1aa0	32414	27	3	12	Medium-quality	56.94	0	unclassified	unclassified
v1aa1	120564	149	10	28	High-quality	100	0	unclassified	unclassified
v1aa2	49716	68	17	1	High-quality	100	0	Siphoviridae	prokaryote
v1aa3	39024	65	21	0	High-quality	97.53	0	Siphoviridae	prokaryote
v1aa4	46536	53	13	3	High-quality	100	0	unclassified	unclassified
v1aa5	32409	47	20	0	Medium-quality	80.15	0	Siphoviridae	prokaryote
v1aa6	45415	59	14	0	High-quality	100	0	unclassified	unclassified
v1aa7	39002	66	21	0	High-quality	97.28	0	Siphoviridae	prokaryote
v1aa8	88996	118	15	4	High-quality	100	0	Quimbyviridae	prokaryote
v1aa9	43978	68	20	2	High-quality	100	0	Siphoviridae	prokaryote
v1aaa	54078	73	11	0	High-quality	98.11	0	unclassified	unclassified
v1aab	75875	85	10	1	Medium-quality	83.2	0	unclassified	unclassified
v1aac	39465	63	8	1	High-quality	99.55	0	unclassified	unclassified
v1aad	43402	78	25	0	High-quality	100	0	unclassified	unclassified
v1aae	34595	43	30	0	Medium-quality	80.81	0	Myoviridae	prokaryote
v1aaf	45915	60	19	0	High-quality	100	0	unclassified	unclassified
v1ab0	117253	142	13	12	High-quality	100	0	unclassified	unclassified
v1ab1	28250	30	4	3	Medium-quality	68.79	0	unclassified	unclassified
v1ab2	46386	67	16	0	Medium-quality	80.68	0	unclassified	unclassified
v1ab3	43532	63	24	0	High-quality	98.47	0	Myoviridae	prokaryote

v1ab4	38169	52	23	1	High-quality	94.95	0	unclassified	unclassified
v1ab5	34619	55	16	0	High-quality	90.63	0	unclassified	unclassified
v1ab6	45976	54	20	0	Medium-quality	76.63	0	unclassified	unclassified
v1ab7	69516	108	18	1	High-quality	100	0	unclassified	unclassified
v1ab8	47200	81	21	1	High-quality	90.54	0	unclassified	unclassified
v1ab9	26752	42	17	0	Medium-quality	79.54	0	Siphoviridae	prokaryote
v1aba	65472	92	19	4	High-quality	100	0	unclassified	unclassified
v1abb	49259	63	43	1	High-quality	97.03	0	Siphoviridae	prokaryote
v1abc	48512	58	25	1	High-quality	100	0	Autographiviridae	prokaryote
v1abd	42841	57	21	4	Medium-quality	56.61	0	Siphoviridae	prokaryote
v1abe	41835	57	38	0	High-quality	100	0	Podoviridae	prokaryote
v1abf	37733	58	12	1	High-quality	96.85	0	Siphoviridae	prokaryote
v1ac0	96046	118	33	10	High-quality	100	0	unclassified	unclassified
v1ac1	44698	80	18	1	High-quality	100	0	unclassified	unclassified
v1ac2	36125	43	9	1	Medium-quality	81.53	0	unclassified	unclassified
v1ac3	28374	42	17	0	Medium-quality	73.31	0	Siphoviridae	prokaryote
v1ac4	37681	56	17	1	High-quality	94.69	0	unclassified	unclassified
v1ac5	16400	19	4	2	Medium-quality	57.16	0	unclassified	unclassified
v1ac6	40441	62	12	1	Medium-quality	65.28	0	unclassified	unclassified
v1ac7	42150	50	9	3	Medium-quality	60.53	13.19	unclassified	unclassified
v1ac8	40504	64	30	0	High-quality	93.46	0	Myoviridae	prokaryote
v1ac9	40357	56	42	0	Medium-quality	72.57	0	Myoviridae	prokaryote
v1aca	51960	73	31	1	High-quality	100	0	Myoviridae	prokaryote
v1acb	39375	50	18	3	High-quality	91.91	0	Siphoviridae	prokaryote
v1acc	147273	161	15	31	High-quality	100	0	unclassified	unclassified
v1acd	67269	98	19	10	High-quality	100	0	unclassified	unclassified
v1ace	43990	48	11	3	High-quality	100	0	unclassified	unclassified
v1acf	43964	64	17	1	High-quality	100	0	unclassified	unclassified
v1ad0	34580	55	18	0	Medium-quality	86.07	0	Siphoviridae	prokaryote
v1ad1	39738	59	32	0	High-quality	100	0	Siphoviridae	prokaryote
v1ad2	66801	89	11	2	High-quality	100	0	unclassified	unclassified
v1ad3	30443	31	1	2	Medium-quality	72.9	0	unclassified	unclassified
v1ad4	39459	33	1	5	Medium-quality	84.88	0	unclassified	unclassified
v1ad5	43925	55	32	1	High-quality	100	0	Siphoviridae	prokaryote
v1ad6	36797	63	22	1	High-quality	100	0	Siphoviridae	prokaryote
v1ad7	38402	44	19	0	High-quality	93.35	0	Siphoviridae	prokaryote
v1ad8	91210	137	13	4	High-quality	100	0	Quimbyviridae	prokaryote
v1ad9	54286	72	7	9	Medium-quality	64.92	0	unclassified	unclassified
v1ada	25210	27	2	3	Medium-quality	60.77	0	unclassified	unclassified
v1adb	72300	59	5	15	High-quality	100	0	unclassified	unclassified

v1adc	88405	93	6	10	High-quality	100	0	unclassified	unclassified
v1add	35339	38	4	19	Medium-quality	76.24	0	unclassified	unclassified
v1ade	38091	55	21	0	High-quality	100	0	Siphoviridae	prokaryote
v1adf	86036	126	20	5	Medium-quality	79.32	0	unclassified	unclassified
v1ae0	50052	79	12	1	High-quality	94.28	0	unclassified	unclassified
v1ae1	37999	50	15	1	High-quality	100	0	unclassified	unclassified
v1ae2	172554	263	64	25	High-quality	100	23.38	unclassified	unclassified
v1ae3	30309	37	9	0	Medium-quality	80.22	0	unclassified	unclassified
v1ae4	41191	48	20	0	High-quality	99.11	0	Myoviridae	prokaryote
v1ae5	124137	177	29	2	Medium-quality	68.28	0	unclassified	unclassified
v1ae6	56483	93	19	1	High-quality	98	0	unclassified	unclassified
v1ae7	180801	300	37	4	High-quality	100	0	unclassified	unclassified
v1ae8	55593	69	23	2	High-quality	100	0	Siphoviridae	prokaryote
v1ae9	22231	27	11	0	Medium-quality	51.94	0	Siphoviridae	prokaryote
v1aea	32997	62	21	0	Medium-quality	87.25	0	Siphoviridae	prokaryote
v1aeb	9383	14	3	1	Medium-quality	78.56	0	unclassified	unclassified
v1aec	44794	61	9	0	Medium-quality	52.07	0	Quimbyviridae	prokaryote
v1aed	34930	64	22	2	High-quality	93.71	0	Siphoviridae	prokaryote
v1aee	36373	64	16	0	High-quality	94.46	0	unclassified	unclassified
v1aef	62111	105	20	2	High-quality	100	0	unclassified	unclassified
v1af0	41497	57	41	0	Medium-quality	73.5	0	Myoviridae	prokaryote
v1af1	13033	12	3	1	High-quality	100	0	unclassified	unclassified
v1af2	117054	154	25	3	Medium-quality	87.19	0	unclassified	unclassified
v1af3	46929	67	19	4	High-quality	100	0	unclassified	unclassified
v1af4	61073	83	14	1	Medium-quality	70.13	0	unclassified	unclassified
v1af5	38353	32	3	2	High-quality	100	0	unclassified	unclassified
v1af6	89206	124	16	2	High-quality	100	0	Quimbyviridae	prokaryote
v1af7	42744	74	22	0	Medium-quality	65.36	0	Siphoviridae	prokaryote
v1af8	34756	57	19	0	High-quality	91.49	0	Siphoviridae	prokaryote
v1af9	24387	44	17	0	Medium-quality	64.86	0	Myoviridae	prokaryote
v1afa	5070	6	1	0	High-quality	100	0	Inoviridae	prokaryote
v1afb	47321	54	6	3	Medium-quality	79.7	0	unclassified	unclassified
v1afc	34010	43	28	1	Medium-quality	75.21	0	Myoviridae	prokaryote
v1afd	32698	53	21	0	Medium-quality	82.13	0	Siphoviridae	prokaryote
v1afe	41707	52	14	0	High-quality	100	0	unclassified	unclassified
v1aff	41708	73	18	4	High-quality	100	0	unclassified	unclassified
v1b00	24208	43	16	0	Medium-quality	68.69	0	Siphoviridae	prokaryote
v1b01	36109	70	21	0	High-quality	95.48	0	unclassified	unclassified
v1b02	30128	37	19	2	Medium-quality	80.74	0	Siphoviridae	prokaryote
v1b03	46620	50	6	2	High-quality	100	0	unclassified	unclassified

v1b04	29723	49	24	1	Medium-quality	87.01	0	Siphoviridae	prokaryote
v1b05	32703	48	19	0	Medium-quality	84.54	0	Siphoviridae	prokaryote
v1b06	38559	57	24	0	High-quality	97.32	0	Siphoviridae	prokaryote
v1b07	70169	80	15	5	High-quality	100	0	unclassified	unclassified
v1b08	32529	33	4	1	Medium-quality	66.88	0	unclassified	unclassified
v1b09	36383	54	40	1	High-quality	92.59	0	Podoviridae	prokaryote
v1b0a	34436	52	29	0	High-quality	100	0	Siphoviridae	prokaryote
v1b0b	39400	57	32	0	High-quality	100	0	Myoviridae	prokaryote
v1b0c	16463	25	7	0	High-quality	96.24	0	unclassified	unclassified
v1b0d	27432	32	5	2	Medium-quality	60.95	0	unclassified	unclassified
v1b0e	30323	32	0	7	Medium-quality	50.65	0	unclassified	unclassified
v1b0f	8216	17	0	0	High-quality	100	0	unclassified	unclassified
v1b10	68378	75	6	10	Medium-quality	82.83	0	unclassified	unclassified
v1b11	12544	12	1	1	High-quality	90.73	0	unclassified	unclassified
v1b12	42251	55	18	0	High-quality	100	0	unclassified	unclassified
v1b13	52249	56	6	1	Medium-quality	59.48	0	Quimbyviridae	prokaryote
v1b14	42213	64	23	0	High-quality	91.36	0	Myoviridae	prokaryote
v1b15	93380	166	36	1	High-quality	100	0	Siphoviridae	prokaryote
v1b16	34033	41	29	1	Medium-quality	74.41	0	Siphoviridae	prokaryote
v1b17	33534	50	15	1	Medium-quality	86.78	0	Siphoviridae	prokaryote
v1b18	193577	331	42	9	High-quality	100	0	unclassified	unclassified
v1b19	82647	111	41	5	High-quality	100	0	Siphoviridae	prokaryote
v1b1a	43498	56	23	2	High-quality	100	0	unclassified	unclassified
v1b1b	38239	60	24	1	High-quality	98.68	0	Siphoviridae	prokaryote
v1b1c	38670	50	33	2	High-quality	98.39	10.58	Myoviridae	prokaryote
v1b1d	40597	51	21	0	High-quality	100	0	Siphoviridae	prokaryote
v1b1e	35338	52	21	0	Medium-quality	89.66	0	Siphoviridae	prokaryote
v1b1f	21108	31	11	0	Medium-quality	57.44	0	unclassified	unclassified
v1b20	77008	110	19	4	High-quality	100	0	unclassified	unclassified
v1b21	59558	87	32	0	High-quality	98.53	0	Siphoviridae	prokaryote
v1b22	127292	174	34	3	High-quality	92.66	0	unclassified	unclassified
v1b23	43645	59	37	0	High-quality	100	0	Myoviridae	prokaryote
v1b24	41709	64	15	1	High-quality	100	0	unclassified	unclassified
v1b25	38731	50	15	2	High-quality	92.78	9.51	unclassified	unclassified
v1b26	33830	42	9	1	Medium-quality	89.54	0	unclassified	unclassified
v1b27	35743	55	19	0	High-quality	100	0	unclassified	unclassified
v1b28	190522	296	34	5	High-quality	100	0	unclassified	unclassified
v1b29	53792	83	19	1	High-quality	100	0	Myoviridae	prokaryote
v1b2a	110070	168	50	13	High-quality	100	0	Siphoviridae	prokaryote
v1b2b	35780	53	18	0	High-quality	100	0	Siphoviridae	prokaryote

v1b2c	60756	71	6	2	High-quality	94.47	0	unclassified	unclassified
v1b2d	91112	120	21	8	High-quality	100	0	Myoviridae	prokaryote
v1b2e	42862	65	27	1	High-quality	98.07	0	Myoviridae	prokaryote
v1b2f	35302	54	21	0	Medium-quality	73.41	0	Myoviridae	prokaryote
v1b30	41295	67	17	0	High-quality	100	0	unclassified	unclassified
v1b31	58086	63	33	6	High-quality	100	0	Siphoviridae	prokaryote
v1b32	35136	57	19	0	High-quality	90.19	0	Siphoviridae	prokaryote
v1b33	37654	52	22	0	High-quality	100	0	Siphoviridae	prokaryote
v1b34	52789	85	29	0	Medium-quality	87.13	0	Siphoviridae	prokaryote
v1b35	35083	42	13	1	Medium-quality	82.78	0	unclassified	unclassified
v1b36	29002	44	20	0	Medium-quality	84.14	0	Siphoviridae	prokaryote
v1b37	28669	32	22	0	Medium-quality	61.29	0	Siphoviridae	prokaryote
v1b38	42032	56	23	0	High-quality	100	0	Siphoviridae	prokaryote
v1b39	100510	103	5	18	Medium-quality	54.95	0	unclassified	unclassified
v1b3a	64979	58	5	14	High-quality	96.48	0	unclassified	unclassified
v1b3b	31390	42	1	13	Medium-quality	67.32	0	unclassified	unclassified
v1b3c	58197	67	1	23	Medium-quality	83.57	0	unclassified	unclassified
v1b3d	51465	68	9	12	High-quality	100	0	Siphoviridae	prokaryote
v1b3e	467445	487	6	240	Complete	100	0	unclassified	unclassified
v1b3f	255016	284	19	45	High-quality	100	0	unclassified	unclassified
v1b40	83842	99	7	16	Medium-quality	63.56	0	unclassified	unclassified
v1b41	8057	11	0	0	Complete	100	0	Inoviridae	prokaryote
v1b42	147973	140	2	49	High-quality	100	0	unclassified	unclassified
v1b43	64384	77	3	26	Medium-quality	81.14	0	unclassified	unclassified
v1b44	38159	50	24	1	Medium-quality	81.76	0	Myoviridae	prokaryote
v1b45	9869	7	2	1	Medium-quality	58.59	7.53	unclassified	unclassified
v1b46	37247	55	45	0	Medium-quality	68.32	0	Myoviridae	prokaryote
v1b47	60092	84	13	2	High-quality	99.16	0	unclassified	unclassified
v1b48	61770	95	34	1	High-quality	100	0	Siphoviridae	prokaryote
v1b49	183108	294	50	7	High-quality	100	0	unclassified	unclassified
v1b4a	57370	82	12	2	Medium-quality	69.1	0	unclassified	unclassified
v1b4b	37576	48	15	1	High-quality	96.55	0	Siphoviridae	prokaryote
v1b4c	194453	303	39	3	High-quality	100	0	unclassified	unclassified
v1b4d	34011	64	10	1	High-quality	97.13	0	unclassified	unclassified
v1b4e	40398	52	12	0	High-quality	97.23	0	unclassified	unclassified
v1b4f	42392	63	27	0	High-quality	100	0	Siphoviridae	prokaryote
v1b50	40042	64	27	0	High-quality	100	0	Siphoviridae	prokaryote
v1b51	35147	49	14	2	High-quality	97.82	0	Siphoviridae	prokaryote
v1b52	73318	106	15	4	Medium-quality	89.92	0	unclassified	unclassified
v1b53	47093	51	12	7	Medium-quality	58.06	41.78	Siphoviridae	prokaryote

v1b54	42291	50	20	1	High-quality	100	0	Siphoviridae	prokaryote
v1b55	38487	49	15	2	Medium-quality	89.43	0	unclassified	unclassified
v1b56	33966	46	14	1	Medium-quality	88.01	0	Siphoviridae	prokaryote
v1b57	36530	56	16	0	High-quality	93.49	0	Siphoviridae	prokaryote
v1b58	42477	68	20	1	High-quality	100	0	unclassified	unclassified
v1b59	36253	60	18	0	High-quality	90.46	0	Siphoviridae	prokaryote
v1b5a	64687	103	32	1	High-quality	100	0	Siphoviridae	prokaryote
v1b5b	194511	307	39	4	High-quality	90.26	0	unclassified	unclassified
v1b5c	37092	52	28	0	Medium-quality	83.26	0	Myoviridae	prokaryote
v1b5d	62495	86	12	1	Medium-quality	71.17	0	Quimbyviridae	prokaryote
v1b5e	34525	59	15	0	High-quality	91.29	0	Siphoviridae	prokaryote
v1b5f	38286	56	28	1	High-quality	96.42	0	Siphoviridae	prokaryote
v1b60	35754	50	27	0	Medium-quality	89.44	0	Siphoviridae	prokaryote
v1b61	28636	47	18	0	Medium-quality	76.04	0	Siphoviridae	prokaryote
v1b62	48400	65	29	0	High-quality	100	0	Siphoviridae	prokaryote
v1b63	39019	62	28	1	High-quality	97.87	0	Siphoviridae	prokaryote
v1b64	36630	53	19	1	High-quality	98.94	0	Siphoviridae	prokaryote
v1b65	36412	51	28	0	Medium-quality	86.31	0	Siphoviridae	prokaryote
v1b66	31110	49	25	0	Medium-quality	82.47	0	Siphoviridae	prokaryote
v1b67	39188	50	22	3	High-quality	100	8.95	Siphoviridae	prokaryote
v1b68	20595	39	15	0	Medium-quality	61.09	0	Siphoviridae	prokaryote
v1b69	41209	73	16	1	High-quality	91.44	0	unclassified	unclassified
v1b6a	129568	195	26	2	Medium-quality	72.78	0	unclassified	unclassified
v1b6b	64273	65	5	0	High-quality	100	0	unclassified	unclassified
v1b6c	56283	70	14	0	High-quality	100	0	unclassified	unclassified
v1b6d	45583	68	17	0	High-quality	100	0	unclassified	unclassified
v1b6e	37265	59	15	0	Medium-quality	87.59	0	unclassified	unclassified
v1b6f	93865	126	20	1	Medium-quality	53.27	0	unclassified	unclassified
v1b70	36867	60	20	0	High-quality	100	0	Siphoviridae	prokaryote
v1b71	43423	51	4	14	Medium-quality	53.96	0	unclassified	unclassified
v1b72	76589	73	7	24	High-quality	100	0	unclassified	unclassified
v1b73	42948	55	0	18	Medium-quality	82.62	0	Myoviridae	prokaryote
v1b74	265613	296	8	88	High-quality	100	0	unclassified	unclassified
v1b75	37859	38	0	6	Medium-quality	52.8	0	unclassified	unclassified
v1b76	162787	148	4	45	High-quality	99.2	0	unclassified	unclassified
v1b77	62408	64	1	21	High-quality	100	0	unclassified	unclassified
v1b78	40433	26	1	8	Medium-quality	89.38	0	unclassified	unclassified
v1b79	80238	86	2	11	Medium-quality	58.8	0	unclassified	unclassified
v1b7a	39890	37	0	9	Medium-quality	54.25	0	unclassified	unclassified
v1b7b	59911	71	7	11	Medium-quality	79.55	0	unclassified	unclassified

v1b7c	23685	37	3	5	Medium-quality	66.47	0	unclassified	unclassified
v1b7d	170201	188	1	62	High-quality	100	0	unclassified	unclassified
v1b7e	50726	65	22	2	High-quality	100	0	Myoviridae	prokaryote
v1b7f	11130	11	3	1	High-quality	100	0	unclassified	unclassified
v1b80	27173	52	19	0	Medium-quality	66.61	0	Siphoviridae	prokaryote
v1b81	29976	52	20	0	High-quality	92.75	0	Siphoviridae	prokaryote
v1b82	35409	32	9	0	Medium-quality	85.3	0	unclassified	unclassified
v1b83	28436	45	17	0	Medium-quality	78.77	0	Siphoviridae	prokaryote
v1b84	38602	42	4	2	Medium-quality	85.34	0	unclassified	unclassified
v1b85	40618	66	20	0	High-quality	100	0	unclassified	unclassified
v1b86	40706	58	28	0	High-quality	100	0	Myoviridae	prokaryote
v1b87	32327	44	21	0	Medium-quality	78.2	0	Siphoviridae	prokaryote
v1b88	57754	87	34	2	High-quality	97.56	0	unclassified	unclassified
v1b89	184862	254	36	8	High-quality	100	0	unclassified	unclassified
v1b8a	163577	242	28	8	High-quality	100	0	unclassified	unclassified
v1b8b	30217	51	18	1	Medium-quality	85.45	0	Siphoviridae	prokaryote
v1b8c	38457	33	6	0	Medium-quality	71.73	0	unclassified	unclassified
v1b8d	163559	239	36	5	High-quality	97.63	0	unclassified	unclassified
v1b8e	39644	56	28	0	Medium-quality	56.92	0	Siphoviridae	prokaryote
v1b8f	37034	52	17	0	High-quality	96.47	0	Siphoviridae	prokaryote
v1b90	31197	42	30	0	Medium-quality	81.39	0	Siphoviridae	prokaryote
v1b91	24827	41	11	1	Medium-quality	76.51	0	unclassified	unclassified
v1b92	36114	57	14	0	High-quality	98.56	0	unclassified	unclassified
v1b93	149903	227	43	3	High-quality	96.74	0	unclassified	unclassified
v1b94	41986	60	22	1	High-quality	100	0	Siphoviridae	prokaryote
v1b95	37879	45	6	1	High-quality	100	0	unclassified	unclassified
v1b96	41022	62	26	0	High-quality	94.08	0	Siphoviridae	prokaryote
v1b97	40835	60	21	4	High-quality	100	0	Siphoviridae	prokaryote
v1b98	56280	58	16	3	High-quality	100	0	Siphoviridae	prokaryote
v1b99	44405	51	24	3	Medium-quality	78.83	0	Siphoviridae	prokaryote
v1b9a	65182	72	13	1	High-quality	96.88	0	unclassified	unclassified
v1b9b	56711	89	9	4	Medium-quality	75.88	0	unclassified	unclassified
v1b9c	50523	70	7	2	Medium-quality	58.04	0	Quimbyviridae	prokaryote
v1b9d	44277	50	25	4	Medium-quality	86.56	26.49	Siphoviridae	prokaryote
v1b9e	67122	83	11	2	High-quality	99.74	0	unclassified	unclassified
v1b9f	38893	65	15	0	Medium-quality	86.14	0	unclassified	unclassified
v1ba0	40493	53	16	1	High-quality	100	0	Siphoviridae	prokaryote
v1ba1	35014	51	28	0	Medium-quality	88.35	0	Siphoviridae	prokaryote
v1ba2	40004	62	27	0	High-quality	100	0	Siphoviridae	prokaryote
v1ba3	18449	27	2	0	High-quality	96.81	0	unclassified	unclassified

v1ba4	29127	44	16	0	Medium-quality	85.5	0	unclassified	unclassified
v1ba5	56622	51	1	17	Medium-quality	79.65	0	unclassified	unclassified
v1ba6	38133	46	1	16	Medium-quality	56.22	0	unclassified	unclassified
v1ba7	9858	11	1	1	High-quality	100	0	unclassified	unclassified
v1ba8	78300	82	3	23	High-quality	100	0	unclassified	unclassified
v1ba9	58069	43	2	13	High-quality	100	0	unclassified	unclassified
v1baa	60719	62	1	16	High-quality	100	0	unclassified	unclassified
v1bab	72658	75	4	15	High-quality	100	0	unclassified	unclassified
v1bac	25371	34	2	6	Medium-quality	52.42	0	unclassified	unclassified
v1bad	30992	45	14	1	Medium-quality	88.96	0	Siphoviridae	prokaryote
v1bae	40438	63	30	0	High-quality	100	0	Siphoviridae	prokaryote
v1baf	42684	55	5	0	High-quality	100	0	unclassified	unclassified
v1bb0	41253	61	21	0	High-quality	100	0	Siphoviridae	prokaryote
v1bb1	36350	54	22	0	Medium-quality	89.45	0	Siphoviridae	prokaryote
v1bb2	44288	58	28	0	High-quality	100	0	Myoviridae	prokaryote
v1bb3	45009	69	29	0	High-quality	100	0	Siphoviridae	prokaryote
v1bb4	54829	79	35	1	High-quality	90.16	0	Siphoviridae	prokaryote
v1bb5	58362	102	29	2	High-quality	94.99	0	Myoviridae	prokaryote
v1bb6	34836	68	15	2	High-quality	93.07	0	Siphoviridae	prokaryote
v1bb7	35851	44	32	1	Medium-quality	72.45	0	Siphoviridae	prokaryote
v1bb8	35776	55	12	3	High-quality	97.1	0	unclassified	unclassified
v1bb9	37214	53	28	1	Medium-quality	86.87	0	Siphoviridae	prokaryote
v1bba	57659	99	14	1	High-quality	100	0	unclassified	unclassified
v1bbb	69261	96	21	3	High-quality	100	0	unclassified	unclassified
v1bbc	85651	118	14	3	High-quality	97.24	0	Quimbyviridae	prokaryote
v1bbd	41881	56	17	0	High-quality	100	0	unclassified	unclassified
v1bbe	45021	66	29	2	High-quality	96.01	0	Myoviridae	prokaryote
v1bbf	37325	55	19	0	High-quality	93.49	0	Siphoviridae	prokaryote
v1bc0	41929	56	44	0	High-quality	100	0	Siphoviridae	prokaryote
v1bc1	49667	82	22	5	High-quality	100	0	Siphoviridae	prokaryote
v1bc2	37347	56	32	0	High-quality	94.54	0	Siphoviridae	prokaryote
v1bc3	22005	33	19	0	Medium-quality	58.48	0	Siphoviridae	prokaryote
v1bc4	40081	68	16	1	High-quality	100	0	unclassified	unclassified
v1bc5	50752	70	11	1	High-quality	100	0	unclassified	unclassified
v1bc6	34274	54	25	0	High-quality	91.9	0	Siphoviridae	prokaryote
v1bc7	28192	44	23	0	Medium-quality	61.5	0	Siphoviridae	prokaryote
v1bc8	48941	55	11	2	Medium-quality	66.2	0	unclassified	unclassified
v1bc9	39348	62	26	0	Medium-quality	87.73	0	Myoviridae	prokaryote
v1bca	23480	28	12	0	Medium-quality	57.7	0	Siphoviridae	prokaryote
v1bcb	33448	45	20	0	Medium-quality	88.86	0	Siphoviridae	prokaryote

v1bcc	43891	57	33	0	High-quality	96.69	0	Siphoviridae	prokaryote
v1bcd	165413	293	32	11	High-quality	97.24	0	unclassified	unclassified
v1bce	28759	41	17	1	Medium-quality	75.29	0	unclassified	unclassified
v1bcf	28640	50	19	0	Medium-quality	84.06	0	Siphoviridae	prokaryote
v1bd0	42840	49	24	2	Medium-quality	80.08	0	Siphoviridae	prokaryote
v1bd1	31701	43	14	1	High-quality	96.31	0	Siphoviridae	prokaryote
v1bd2	39189	40	12	0	High-quality	98.17	0	Siphoviridae	prokaryote
v1bd3	35454	50	27	0	Medium-quality	83.95	0	Siphoviridae	prokaryote
v1bd4	38338	57	15	0	High-quality	94.11	0	Siphoviridae	prokaryote
v1bd5	42361	58	42	0	High-quality	92.02	0	Siphoviridae	prokaryote
v1bd6	78558	112	27	4	High-quality	99.38	0	Myoviridae	prokaryote
v1bd7	34851	60	19	0	Medium-quality	89.23	0	unclassified	unclassified
v1bd8	41253	58	28	0	Medium-quality	88.18	0	unclassified	unclassified
v1bd9	53204	78	33	0	Medium-quality	87.14	0	Siphoviridae	prokaryote
v1bda	44919	52	21	4	High-quality	95.54	0	Siphoviridae	prokaryote
v1bdb	38814	49	36	1	High-quality	100	0	Siphoviridae	prokaryote
v1bdc	48355	56	22	2	High-quality	100	0	Autographiviridae	prokaryote
v1bdd	153183	243	40	2	High-quality	95.14	0	unclassified	unclassified
v1bde	38735	55	23	0	High-quality	97.3	0	Siphoviridae	prokaryote
v1bdf	38939	60	23	3	High-quality	99.52	0	Siphoviridae	prokaryote
v1be0	65934	73	15	2	High-quality	97.98	0	unclassified	unclassified
v1be1	45564	52	21	2	High-quality	96.81	0	Siphoviridae	prokaryote
v1be2	21868	32	18	0	Medium-quality	52.82	0	Siphoviridae	prokaryote
v1be3	160207	257	32	5	High-quality	100	0	unclassified	unclassified
v1be4	44454	45	1	8	High-quality	98.27	0	unclassified	unclassified
v1be5	67274	87	1	25	High-quality	99.19	0	unclassified	unclassified
v1be6	37202	51	20	2	High-quality	100	0	Siphoviridae	prokaryote
v1be7	55393	72	5	3	High-quality	96	0	unclassified	unclassified
v1be8	39352	61	31	1	Medium-quality	84.63	0	Siphoviridae	prokaryote
v1be9	33670	52	12	1	Medium-quality	80.65	0	unclassified	unclassified
v1bea	164406	209	39	6	High-quality	100	0	unclassified	unclassified
v1beb	32019	52	17	0	Medium-quality	81.27	0	Siphoviridae	prokaryote
v1bec	50155	67	10	2	Medium-quality	57	0	Quimbyviridae	prokaryote
v1bed	30470	35	11	0	Medium-quality	56.89	0	unclassified	unclassified
v1bee	48761	64	18	1	High-quality	100	0	unclassified	unclassified
v1bef	10316	16	0	0	High-quality	100	0	unclassified	unclassified
v1bf0	44203	53	24	1	High-quality	100	0	Autographiviridae	prokaryote
v1bf1	54666	79	20	7	High-quality	100	0	Siphoviridae	prokaryote
v1bf2	87678	98	24	13	High-quality	91.04	2.37	unclassified	unclassified
v1bf3	22756	34	12	0	Medium-quality	61.93	0	Siphoviridae	prokaryote

v1bf4	41853	60	17	0	Medium-quality	84.28	0	unclassified	unclassified
v1bf5	35724	51	21	0	High-quality	95.4	0	Siphoviridae	prokaryote
v1bf6	39325	55	17	0	High-quality	100	0	Siphoviridae	prokaryote
v1bf7	47227	60	15	1	Medium-quality	82.97	0	Myoviridae	prokaryote
v1bf8	42062	51	14	6	Medium-quality	75.86	25.29	unclassified	unclassified
v1bf9	117779	142	33	6	High-quality	99.76	0	unclassified	unclassified
v1bfa	18791	24	0	3	High-quality	100	0	unclassified	unclassified
v1bfb	91061	127	5	10	Medium-quality	73.48	0	unclassified	unclassified
v1bfc	92017	88	4	26	Medium-quality	82.99	0	unclassified	unclassified
v1bfd	37668	58	18	0	High-quality	92.89	0	Siphoviridae	prokaryote
v1bfe	43505	47	24	0	High-quality	99.34	0	Autographiviridae	prokaryote
v1bff	38522	58	10	2	Medium-quality	63.03	0	unclassified	unclassified
v1c00	100269	158	60	2	High-quality	100	0	Siphoviridae	prokaryote
v1c01	125992	182	26	7	High-quality	100	0	unclassified	unclassified
v1c02	33591	60	19	0	Medium-quality	67.16	0	Siphoviridae	prokaryote
v1c03	32370	51	21	0	Medium-quality	86.99	0	Siphoviridae	prokaryote
v1c04	47456	58	6	2	High-quality	100	0	unclassified	unclassified
v1c05	38515	57	22	0	Medium-quality	80.11	0	Myoviridae	prokaryote
v1c06	50202	48	8	1	Medium-quality	82.47	0	unclassified	unclassified
v1c07	52190	79	17	4	Medium-quality	87.23	0	unclassified	unclassified
v1c08	75774	62	4	33	Complete	100	0	unclassified	unclassified
v1c09	35114	47	2	15	High-quality	93.22	0	unclassified	unclassified
v1c0a	32198	49	16	1	High-quality	99.35	0	Siphoviridae	prokaryote
v1c0b	44959	71	53	0	High-quality	100	0	Siphoviridae	prokaryote
v1c0c	59693	53	6	0	High-quality	94.95	0	unclassified	unclassified
v1c0d	39785	52	17	0	High-quality	99.58	0	Siphoviridae	prokaryote
v1c0e	29995	43	22	0	Medium-quality	70.08	0	Siphoviridae	prokaryote
v1c0f	34606	52	28	1	High-quality	94.58	0	Siphoviridae	prokaryote
v1c10	49220	71	13	0	Medium-quality	68.07	0	unclassified	unclassified
v1c11	28367	38	17	0	Medium-quality	80.39	0	unclassified	unclassified
v1c12	36086	65	15	3	High-quality	91.17	0	Siphoviridae	prokaryote
v1c13	33797	50	30	1	High-quality	97.05	0	Myoviridae	prokaryote
v1c14	33461	60	34	0	Medium-quality	72.76	0	Siphoviridae	prokaryote
v1c15	34681	60	21	0	High-quality	100	0	Myoviridae	prokaryote
v1c16	40856	52	30	1	High-quality	93.75	0	Myoviridae	prokaryote
v1c17	41364	66	25	0	High-quality	100	0	Siphoviridae	prokaryote
v1c18	56421	60	4	7	High-quality	100	0	Myoviridae	prokaryote
v1c19	38681	44	3	4	Medium-quality	61.05	0	unclassified	unclassified
v1c1a	119422	141	7	32	High-quality	100	0	unclassified	unclassified
v1c1b	79785	88	3	23	High-quality	100	0	unclassified	unclassified

v1c1c	14387	25	0	0	High-quality	100	0	unclassified	unclassified
v1c1d	5758	15	0	0	High-quality	92.84	0	unclassified	unclassified
v1c1e	61178	63	1	20	High-quality	100	0	unclassified	unclassified
v1c1f	122248	119	5	20	High-quality	100	0	unclassified	unclassified
v1c20	43957	63	16	1	High-quality	100	0	unclassified	unclassified
v1c21	38307	55	18	0	High-quality	100	0	Siphoviridae	prokaryote
v1c22	38580	68	21	0	Medium-quality	86.68	0	unclassified	unclassified
v1c23	50646	61	18	3	High-quality	97.25	0	Myoviridae	prokaryote
v1c24	53334	50	23	5	Medium-quality	73.96	30.11	Siphoviridae	prokaryote
v1c25	44078	60	14	1	Medium-quality	71.69	0	unclassified	unclassified
v1c26	55048	73	39	0	High-quality	100	0	Siphoviridae	prokaryote
v1c27	41573	61	37	1	High-quality	100	0	Myoviridae	prokaryote
v1c28	159793	199	31	9	High-quality	100	0	unclassified	unclassified
v1c29	6453	8	1	0	High-quality	100	0	unclassified	unclassified
v1c2a	41022	55	8	1	Medium-quality	72.86	0	unclassified	unclassified
v1c2b	11111	17	2	0	Medium-quality	87.49	0	unclassified	unclassified
v1c2c	21361	31	25	0	Medium-quality	67.21	0	Myoviridae	prokaryote
v1c2d	35454	61	16	0	High-quality	97.07	0	Siphoviridae	prokaryote
v1c2e	34789	56	21	0	Medium-quality	89.73	0	Siphoviridae	prokaryote
v1c2f	50357	71	20	9	Medium-quality	59.39	22.19	Siphoviridae	prokaryote
v1c30	37151	48	19	2	High-quality	98.88	0	Siphoviridae	prokaryote
v1c31	38296	50	13	1	High-quality	100	0	unclassified	unclassified
v1c32	83816	90	15	14	High-quality	100	12.26	unclassified	unclassified
v1c33	226961	215	16	72	High-quality	100	0	unclassified	unclassified
v1c34	92832	109	1	12	Medium-quality	58.81	0	unclassified	unclassified
v1c35	68120	64	3	20	High-quality	100	0	unclassified	unclassified
v1c36	33844	40	1	6	Medium-quality	56.53	0	unclassified	unclassified
v1c37	148526	155	18	82	High-quality	100	0	unclassified	unclassified
v1c38	37939	44	0	9	Medium-quality	63.37	0	unclassified	unclassified
v1c39	71846	82	7	15	High-quality	90.16	0	unclassified	unclassified
v1c3a	7330	7	0	2	Medium-quality	61.73	0	unclassified	unclassified
v1c3b	32942	51	26	0	Medium-quality	72.91	0	Myoviridae	prokaryote
v1c3c	40307	50	24	5	Medium-quality	84.33	27.03	Siphoviridae	prokaryote
v1c3d	142313	192	40	6	High-quality	100	0	unclassified	unclassified
v1c3e	10230	10	3	1	High-quality	100	25.5	unclassified	unclassified
v1c3f	43044	54	22	0	High-quality	91.11	0	Siphoviridae	prokaryote
v1c40	33327	42	16	1	Medium-quality	88.11	0	Siphoviridae	prokaryote
v1c41	40304	47	23	2	Medium-quality	85.99	0	Siphoviridae	prokaryote
v1c42	33402	53	18	0	Medium-quality	89.97	0	Siphoviridae	prokaryote
v1c43	29582	46	18	1	Medium-quality	78.13	0	Myoviridae	prokaryote

v1c44	50142	76	16	1	Medium-quality	78.91	0	Siphoviridae	prokaryote
v1c45	45106	72	15	0	High-quality	100	0	unclassified	unclassified
v1c46	79151	116	15	8	Medium-quality	53.38	0	unclassified	unclassified
v1c47	28737	47	13	0	Medium-quality	72.53	0	Siphoviridae	prokaryote
v1c48	41261	51	21	0	High-quality	100	0	Autographiviridae	prokaryote
v1c49	37675	58	27	1	High-quality	96.47	0	Myoviridae	prokaryote
v1c4a	25550	34	17	0	Medium-quality	72.34	0	Siphoviridae	prokaryote
v1c4b	43797	66	26	0	High-quality	100	0	Siphoviridae	prokaryote
v1c4c	49798	76	18	0	High-quality	100	0	unclassified	unclassified
v1c4d	25982	45	22	1	Medium-quality	76.68	0	Siphoviridae	prokaryote
v1c4e	24034	28	14	2	Medium-quality	53.6	0	Siphoviridae	prokaryote
v1c4f	43214	72	10	0	High-quality	100	0	unclassified	unclassified
v1c50	33310	50	20	0	Medium-quality	78.97	0	unclassified	unclassified
v1c51	126722	174	32	2	Medium-quality	69.99	0	unclassified	unclassified
v1c52	36860	57	15	0	High-quality	96.11	0	Siphoviridae	prokaryote
v1c53	44999	68	18	1	Medium-quality	68.53	0	Siphoviridae	prokaryote
v1c54	43929	54	26	2	High-quality	96.5	18.7	Siphoviridae	prokaryote
v1c55	34069	47	18	1	High-quality	90.04	0	Siphoviridae	prokaryote
v1c56	42191	64	19	1	High-quality	100	0	unclassified	unclassified
v1c57	40628	68	21	0	High-quality	100	0	Siphoviridae	prokaryote
v1c58	53034	81	32	0	High-quality	100	0	unclassified	unclassified
v1c59	43188	65	26	1	High-quality	100	0	Siphoviridae	prokaryote
v1c5a	34524	46	19	1	Medium-quality	80.54	0	Siphoviridae	prokaryote
v1c5b	26672	35	23	0	Medium-quality	56.82	0	Siphoviridae	prokaryote
v1c5c	41688	50	25	1	High-quality	100	0	Autographiviridae	prokaryote
v1c5d	82844	100	33	12	High-quality	100	36.4	unclassified	unclassified
v1c5e	44329	58	11	4	High-quality	99.96	0	unclassified	unclassified
v1c5f	37610	58	18	0	High-quality	99.02	0	unclassified	unclassified
v1c60	47013	51	25	2	Medium-quality	78.17	14.1	Siphoviridae	prokaryote
v1c61	88865	121	39	1	High-quality	100	0	unclassified	unclassified
v1c62	19231	34	17	0	Medium-quality	54.72	0	Siphoviridae	prokaryote
v1c63	42176	66	23	0	High-quality	99.11	0	Myoviridae	prokaryote
v1c64	28098	37	4	3	High-quality	97.93	0	unclassified	unclassified
v1c65	44675	77	18	0	High-quality	100	0	unclassified	unclassified
v1c66	30545	54	15	0	Medium-quality	89.95	0	Siphoviridae	prokaryote
v1c67	51159	70	17	4	High-quality	100	0	unclassified	unclassified
v1c68	47342	92	35	2	High-quality	100	0	Myoviridae	prokaryote
v1c69	31865	37	26	0	Medium-quality	78.83	0	Podoviridae	prokaryote
v1c6a	37781	59	22	0	High-quality	100	0	Siphoviridae	prokaryote
v1c6b	38736	46	16	0	High-quality	100	0	unclassified	unclassified

v1c6c	88694	123	16	7	High-quality	100	0	Myoviridae	prokaryote
v1c6d	36883	53	19	1	High-quality	92.41	0	Siphoviridae	prokaryote
v1c6e	36867	58	22	0	Medium-quality	58.2	0	Siphoviridae	prokaryote
v1c6f	46853	82	17	0	High-quality	100	0	unclassified	unclassified
v1c70	57966	80	16	1	High-quality	100	0	unclassified	unclassified
v1c71	37945	54	13	1	High-quality	100	0	unclassified	unclassified
v1c72	37268	43	22	0	Medium-quality	79.7	0	Siphoviridae	prokaryote
v1c73	39693	51	31	0	High-quality	95.94	0	Siphoviridae	prokaryote
v1c74	34585	51	24	0	High-quality	98.07	0	Siphoviridae	prokaryote
v1c75	36837	61	16	1	High-quality	99.59	0	Siphoviridae	prokaryote
v1c76	40260	70	23	3	Medium-quality	67.06	0	Siphoviridae	prokaryote
v1c77	48115	80	22	0	High-quality	100	0	unclassified	unclassified
v1c78	39109	50	18	7	Complete	100	22.49	Siphoviridae	prokaryote
v1c79	35430	61	27	0	High-quality	92.94	0	Siphoviridae	prokaryote
v1c7a	39631	52	20	0	High-quality	95.97	0	unclassified	unclassified
v1c7b	37608	50	15	1	Medium-quality	82.44	0	unclassified	unclassified
v1c7c	70064	79	22	4	High-quality	100	0	Siphoviridae	prokaryote
v1c7d	37157	53	26	1	High-quality	92.13	0	Siphoviridae	prokaryote
v1c7e	38003	52	40	0	High-quality	100	0	Siphoviridae	prokaryote
v1c7f	43799	54	10	1	High-quality	100	0	unclassified	unclassified
v1c80	36162	50	22	0	High-quality	94.56	0	Siphoviridae	prokaryote
v1c81	64085	116	26	1	High-quality	100	0	unclassified	unclassified
v1c82	103137	129	29	8	High-quality	100	0	unclassified	unclassified
v1c83	26886	29	8	0	Medium-quality	67.78	0	unclassified	unclassified
v1c84	23101	36	7	0	Medium-quality	61.14	0	unclassified	unclassified
v1c85	51456	74	29	1	High-quality	100	0	unclassified	unclassified
v1c86	31314	44	11	0	High-quality	95.45	0	unclassified	unclassified
v1c87	17950	27	7	1	High-quality	100	0	unclassified	unclassified
v1c88	44863	58	22	3	High-quality	100	0	Siphoviridae	prokaryote
v1c89	36204	50	19	1	High-quality	100	0	Siphoviridae	prokaryote
v1c8a	34878	36	18	0	Medium-quality	84.76	0	Myoviridae	prokaryote
v1c8b	36715	49	26	0	Medium-quality	82.66	0	Siphoviridae	prokaryote
v1c8c	45195	50	25	7	Medium-quality	58.75	25.6	Siphoviridae	prokaryote
v1c8d	39480	62	39	1	Medium-quality	89.28	0	Siphoviridae	prokaryote
v1c8e	37904	52	26	0	High-quality	93.53	0	Myoviridae	prokaryote
v1c8f	38548	46	22	0	High-quality	91.49	0	Siphoviridae	prokaryote
v1c90	21902	33	21	0	Medium-quality	53.85	0	Podoviridae	prokaryote
v1c91	40202	64	13	0	Medium-quality	66.98	0	Quimbyviridae	prokaryote
v1c92	28150	36	29	0	Medium-quality	56.78	0	Siphoviridae	prokaryote
v1c93	41200	59	32	0	High-quality	100	0	Siphoviridae	prokaryote

v1c94	32482	47	25	0	High-quality	96.25	0	Siphoviridae	prokaryote
v1c95	39993	61	16	2	Medium-quality	77.46	0	Siphoviridae	prokaryote
v1c96	31385	41	28	0	Medium-quality	75.44	0	Siphoviridae	prokaryote
v1c97	36055	51	37	0	High-quality	90.03	0	Siphoviridae	prokaryote
v1c98	59006	81	34	0	High-quality	100	0	Myoviridae	prokaryote
v1c99	54008	72	4	7	High-quality	100	0	unclassified	unclassified
v1c9a	34131	59	27	0	Medium-quality	85.02	0	Siphoviridae	prokaryote
v1c9b	50270	56	9	3	Medium-quality	78.65	0	unclassified	unclassified
v1c9c	39714	57	42	0	High-quality	100	0	Podoviridae	prokaryote
v1c9d	20127	24	18	0	Medium-quality	50.17	0	Podoviridae	prokaryote
v1c9e	31561	44	20	0	Medium-quality	78.1	0	Siphoviridae	prokaryote
v1c9f	38627	47	24	3	Medium-quality	82.4	0	Siphoviridae	prokaryote
v1ca0	48458	48	9	5	Medium-quality	75.29	0	unclassified	unclassified
v1ca1	32104	54	27	1	Medium-quality	72.6	0	Siphoviridae	prokaryote
v1ca2	43266	64	33	0	High-quality	100	0	Siphoviridae	prokaryote
v1ca3	21767	32	0	9	Medium-quality	54.66	0	unclassified	unclassified
v1ca4	27723	37	26	0	Medium-quality	68.3	0	Siphoviridae	prokaryote
v1ca5	40993	62	16	1	High-quality	100	0	Siphoviridae	prokaryote
v1ca6	39406	43	4	0	High-quality	97.92	0	unclassified	unclassified
v1ca7	37121	49	22	0	Medium-quality	71.92	0	Myoviridae	prokaryote
v1ca8	36725	43	28	1	Medium-quality	86.2	0	Siphoviridae	prokaryote
v1ca9	34238	57	49	0	Medium-quality	65.55	0	Myoviridae	prokaryote
v1caa	41586	48	20	1	High-quality	100	0	Siphoviridae	prokaryote
v1cab	38595	64	22	0	High-quality	94.94	0	Siphoviridae	prokaryote
v1cac	20487	29	14	0	Medium-quality	53.63	0	Siphoviridae	prokaryote
v1cad	54745	80	28	2	High-quality	100	0	unclassified	unclassified
v1cae	36601	50	23	0	High-quality	96.77	0	Siphoviridae	prokaryote
v1caf	36926	50	30	0	High-quality	100	0	Siphoviridae	prokaryote
v1cb0	43607	57	36	2	Medium-quality	83.45	0	Myoviridae	prokaryote
v1cb1	32657	50	1	3	Medium-quality	61.96	0	Myoviridae	prokaryote
v1cb2	40531	37	1	5	Medium-quality	86.1	0	unclassified	unclassified
v1cb3	100109	96	1	33	High-quality	100	0	unclassified	unclassified
v1cb4	67544	90	3	5	Medium-quality	59.68	0	unclassified	unclassified
v1cb5	38793	43	1	8	Medium-quality	86.39	0	unclassified	unclassified
v1cb6	83644	86	4	18	High-quality	100	0	unclassified	unclassified
v1cb7	59087	62	1	21	High-quality	92.27	0	unclassified	unclassified
v1cb8	41137	39	2	9	Medium-quality	54.08	0	unclassified	unclassified
v1cb9	35983	32	1	9	Medium-quality	54.45	0	unclassified	unclassified
v1cba	47150	52	1	17	High-quality	100	0	unclassified	unclassified
v1cbb	226525	291	61	11	High-quality	100	0	unclassified	unclassified

v1cbc	27179	41	10	0	Medium-quality	79.6	0	unclassified	unclassified
v1cbd	21303	36	7	0	Medium-quality	60.03	0	unclassified	unclassified
v1cbe	39612	53	29	1	High-quality	100	0	Siphoviridae	prokaryote
v1cbf	57566	63	7	5	High-quality	100	0	unclassified	unclassified
v1cc0	38216	70	27	1	Medium-quality	82.38	0	Myoviridae	prokaryote
v1cc1	29655	37	9	2	Medium-quality	62.81	0	Siphoviridae	prokaryote
v1cc2	44048	60	22	3	High-quality	100	0	Siphoviridae	prokaryote
v1cc3	82738	107	21	8	High-quality	98.01	0	Myoviridae	prokaryote
v1cc4	45346	46	4	2	High-quality	100	0	unclassified	unclassified
v1cc5	61659	104	29	2	High-quality	100	0	unclassified	unclassified
v1cc6	50188	73	16	2	Medium-quality	50.48	0	Siphoviridae	prokaryote
v1cc7	46442	44	9	8	Complete	100	38.16	unclassified	unclassified
v1cc8	37357	56	9	1	High-quality	100	0	unclassified	unclassified
v1cc9	44157	72	29	2	High-quality	91.97	0	unclassified	unclassified
v1cca	6038	11	6	0	High-quality	100	0	Microviridae	prokaryote
v1ccb	43580	50	4	2	High-quality	97.25	0	unclassified	unclassified
v1ccc	32665	40	18	0	Medium-quality	89.13	0	Siphoviridae	prokaryote
v1ccd	66944	105	26	2	High-quality	100	0	Myoviridae	prokaryote
v1cce	77115	123	12	1	High-quality	98.34	0	Quimbyviridae	prokaryote
v1ccf	47316	68	15	0	High-quality	100	0	unclassified	unclassified
v1cd0	22072	24	21	0	Medium-quality	55.5	0	Siphoviridae	prokaryote
v1cd1	40446	58	34	0	High-quality	100	0	Siphoviridae	prokaryote
v1cd2	59363	82	31	1	High-quality	100	0	Siphoviridae	prokaryote
v1cd3	53464	78	12	1	High-quality	100	0	unclassified	unclassified
v1cd4	39991	54	8	2	High-quality	98.64	0	unclassified	unclassified
v1cd5	15060	18	3	0	Medium-quality	52.5	0	unclassified	unclassified
v1cd6	76957	116	22	4	High-quality	100	0	unclassified	unclassified
v1cd7	42565	55	22	0	High-quality	98.49	0	Myoviridae	prokaryote
v1cd8	46588	84	12	0	High-quality	100	0	unclassified	unclassified
v1cd9	18380	18	1	1	High-quality	100	0	unclassified	unclassified
v1cda	87105	82	1	28	High-quality	100	0	unclassified	unclassified
v1cdb	94125	86	2	29	High-quality	100	0	unclassified	unclassified
v1cdc	116914	179	15	19	High-quality	100	0	unclassified	unclassified
v1cdd	36002	39	5	0	High-quality	95	0	unclassified	unclassified
v1cde	38626	58	21	0	High-quality	95.19	0	unclassified	unclassified
v1cdf	35978	51	20	0	High-quality	97.83	0	Siphoviridae	prokaryote
v1ce0	97832	174	36	0	High-quality	100	0	unclassified	unclassified
v1ce1	23310	32	13	0	Medium-quality	60.15	0	Siphoviridae	prokaryote
v1ce2	30437	42	23	0	Medium-quality	76.45	0	Siphoviridae	prokaryote
v1ce3	38436	51	21	0	High-quality	99.06	0	Siphoviridae	prokaryote

v1ce4	29445	54	37	0	Medium-quality	64.08	0	Siphoviridae	prokaryote
v1ce5	45290	62	26	0	High-quality	100	0	Siphoviridae	prokaryote
v1ce6	33822	46	20	1	Medium-quality	84.05	0	unclassified	unclassified
v1ce7	37540	53	14	0	High-quality	100	0	Siphoviridae	prokaryote
v1ce8	56012	65	23	1	High-quality	100	0	Siphoviridae	prokaryote
v1ce9	42552	55	32	1	High-quality	100	0	Myoviridae	prokaryote
v1cea	34112	50	13	2	High-quality	97.92	0	unclassified	unclassified
v1ceb	25798	40	14	0	Medium-quality	73.14	0	unclassified	unclassified
v1cec	42050	49	7	2	High-quality	100	0	unclassified	unclassified
v1ced	30744	52	16	0	Medium-quality	86.53	0	unclassified	unclassified
v1cee	40262	65	20	1	High-quality	97.01	0	Siphoviridae	prokaryote
v1cef	38006	50	13	2	High-quality	98.72	10.35	Siphoviridae	prokaryote
v1cf0	32247	41	19	0	Medium-quality	83.77	0	Siphoviridae	prokaryote
v1cf1	58334	90	24	0	High-quality	97.09	0	unclassified	unclassified
v1cf2	34979	50	15	1	Medium-quality	89.83	0	Siphoviridae	prokaryote
v1cf3	78216	64	6	3	High-quality	100	0	unclassified	unclassified
v1cf4	35725	50	9	0	Medium-quality	88.12	0	unclassified	unclassified
v1cf5	6727	8	1	0	High-quality	100	0	unclassified	unclassified
v1cf6	14656	16	4	2	Medium-quality	51.09	0	unclassified	unclassified
v1cf7	155275	158	23	106	High-quality	90.2	0	unclassified	unclassified
v1cf8	71820	76	2	22	Medium-quality	57.32	0	unclassified	unclassified
v1cf9	38135	36	0	10	Medium-quality	54.04	0	unclassified	unclassified
v1cfa	63630	72	1	24	Medium-quality	57.94	0	unclassified	unclassified
v1cfb	48665	47	2	16	High-quality	95.03	0	unclassified	unclassified
v1cfc	86949	119	19	4	High-quality	100	0	unclassified	unclassified
v1cfd	55106	94	18	1	High-quality	100	0	unclassified	unclassified
v1cfe	44073	52	18	5	High-quality	97.91	30.49	Siphoviridae	prokaryote
v1cff	27588	41	23	0	Medium-quality	78.71	0	Siphoviridae	prokaryote
v1d00	166038	220	11	5	Medium-quality	76.44	0	unclassified	unclassified
v1d01	40328	70	22	0	High-quality	96.89	0	Myoviridae	prokaryote
v1d02	29600	40	6	0	Medium-quality	73.38	0	unclassified	unclassified
v1d03	69797	75	11	3	High-quality	100	0	unclassified	unclassified
v1d04	35795	58	46	0	Medium-quality	88.33	0	Podoviridae	prokaryote
v1d05	41531	65	14	1	High-quality	97.62	0	unclassified	unclassified
v1d06	42300	70	15	0	High-quality	100	0	unclassified	unclassified
v1d07	42144	67	21	0	High-quality	100	0	Siphoviridae	prokaryote
v1d08	37404	49	25	1	High-quality	100	0	Siphoviridae	prokaryote
v1d09	36224	49	20	3	Medium-quality	89.27	14.29	unclassified	unclassified
v1d0a	34089	49	17	2	High-quality	93.4	0	unclassified	unclassified
v1d0b	36887	60	28	0	Medium-quality	83.01	0	Myoviridae	prokaryote

v1d0c	34805	52	27	1	High-quality	98.73	0	Siphoviridae	prokaryote
v1d0d	39399	62	16	0	Medium-quality	87.33	0	unclassified	unclassified
v1d0e	37112	38	10	4	High-quality	100	0	unclassified	unclassified
v1d0f	44199	52	3	1	High-quality	97.71	0	unclassified	unclassified
v1d10	52075	65	9	0	Medium-quality	77.39	0	unclassified	unclassified
v1d11	44972	60	26	3	High-quality	100	20.72	Siphoviridae	prokaryote
v1d12	38699	52	12	0	High-quality	99.47	0	unclassified	unclassified
v1d13	37537	53	22	0	Medium-quality	89.72	0	Siphoviridae	prokaryote
v1d14	43971	68	20	0	High-quality	97.31	0	unclassified	unclassified
v1d15	40966	62	8	2	High-quality	100	0	unclassified	unclassified
v1d16	30221	41	25	0	Medium-quality	84.74	0	Siphoviridae	prokaryote
v1d17	22746	21	3	2	Medium-quality	55.23	0	unclassified	unclassified
v1d18	78061	132	25	4	High-quality	100	0	unclassified	unclassified
v1d19	24205	30	19	0	Medium-quality	59.57	0	Siphoviridae	prokaryote
v1d1a	36909	57	27	0	High-quality	90.95	0	Myoviridae	prokaryote
v1d1b	31268	42	17	0	Medium-quality	69.42	0	Siphoviridae	prokaryote
v1d1c	44398	53	18	4	High-quality	100	0	unclassified	unclassified
v1d1d	36367	55	19	2	High-quality	100	0	Siphoviridae	prokaryote
v1d1e	58405	85	18	3	High-quality	100	0	unclassified	unclassified
v1d1f	35729	48	8	1	Medium-quality	56.99	0	unclassified	unclassified
v1d20	52448	76	18	1	Medium-quality	78.62	0	unclassified	unclassified
v1d21	55206	50	8	3	Medium-quality	60.3	16.19	unclassified	unclassified
v1d22	53670	95	20	3	High-quality	93.99	0	unclassified	unclassified
v1d23	14411	21	6	0	Medium-quality	85.95	0	unclassified	unclassified
v1d24	46058	59	2	9	Medium-quality	62.04	0	unclassified	unclassified
v1d25	26300	39	2	11	Medium-quality	66.46	0	unclassified	unclassified
v1d26	30917	38	2	11	Medium-quality	63.23	0	unclassified	unclassified
v1d27	57068	57	1	11	High-quality	100	0	unclassified	unclassified
v1d28	99725	118	1	40	High-quality	100	0	unclassified	unclassified
v1d29	227529	246	16	74	High-quality	100	0	unclassified	unclassified
v1d2a	38024	44	1	13	Medium-quality	50.01	0	unclassified	unclassified
v1d2b	37219	35	1	10	Medium-quality	68.34	0	unclassified	unclassified
v1d2c	62591	71	1	18	Medium-quality	70.26	0	unclassified	unclassified
v1d2d	19753	24	1	7	Medium-quality	58.1	0	unclassified	unclassified
v1d2e	126927	110	3	28	Medium-quality	86.36	0	unclassified	unclassified
v1d2f	41618	72	13	4	Medium-quality	73.78	0	unclassified	unclassified
v1d30	48712	74	19	0	High-quality	100	0	unclassified	unclassified
v1d31	64495	82	8	4	High-quality	100	0	unclassified	unclassified
v1d32	35657	50	12	0	High-quality	93.76	0	unclassified	unclassified
v1d33	33536	47	22	0	Medium-quality	79.99	0	unclassified	unclassified

v1d34	46958	70	28	1	High-quality	100	0	Siphoviridae	prokaryote
v1d35	93289	118	38	15	High-quality	100	0	unclassified	unclassified
v1d36	41580	67	22	0	High-quality	100	0	unclassified	unclassified
v1d37	37147	64	26	1	High-quality	94.76	0	unclassified	unclassified
v1d38	49206	72	31	0	High-quality	90.45	0	unclassified	unclassified
v1d39	35375	54	31	0	High-quality	100	0	Myoviridae	prokaryote
v1d3a	33408	33	3	2	Medium-quality	79.84	0	unclassified	unclassified
v1d3b	23642	41	10	1	Medium-quality	63.08	0	Siphoviridae	prokaryote
v1d3c	40545	42	4	2	Medium-quality	83.77	0	unclassified	unclassified
v1d3d	45860	51	13	4	Medium-quality	89.52	0	unclassified	unclassified
v1d3e	34071	46	13	1	Medium-quality	84.18	0	unclassified	unclassified
v1d3f	21207	36	5	1	Medium-quality	59.27	0	unclassified	unclassified
v1d40	63276	114	20	0	High-quality	100	0	unclassified	unclassified
v1d41	133345	123	10	61	Complete	100	77.34	unclassified	unclassified
v1d42	36230	57	13	0	Medium-quality	86.81	0	unclassified	unclassified
v1d43	46769	61	25	0	High-quality	100	0	Siphoviridae	prokaryote
v1d44	26340	36	14	0	Medium-quality	58.44	0	unclassified	unclassified
v1d45	54673	92	19	1	High-quality	92.83	0	unclassified	unclassified
v1d46	51340	66	15	3	High-quality	100	0	unclassified	unclassified
v1d47	52108	66	31	3	High-quality	94.03	0	Siphoviridae	prokaryote
v1d48	74758	100	14	3	High-quality	100	0	Quimbyviridae	prokaryote
v1d49	62810	78	13	1	High-quality	100	0	unclassified	unclassified
v1d4a	55341	84	15	2	Medium-quality	51.75	0	unclassified	unclassified
v1d4b	88370	95	12	10	Medium-quality	65.02	54.64	unclassified	unclassified
v1d4c	40502	51	24	3	Medium-quality	86.95	25.9	Siphoviridae	prokaryote
v1d4d	35100	55	20	0	Medium-quality	87.6	0	Siphoviridae	prokaryote
v1d4e	34012	26	1	9	Medium-quality	73.16	0	unclassified	unclassified
v1d4f	97145	108	3	23	Medium-quality	65.31	0	unclassified	unclassified
v1d50	59919	48	2	14	High-quality	100	0	unclassified	unclassified
v1d51	51654	57	1	16	Medium-quality	78.16	0	unclassified	unclassified
v1d52	80709	77	2	34	Complete	100	0	unclassified	unclassified
v1d53	58548	67	3	20	Medium-quality	66.23	0	unclassified	unclassified
v1d54	68709	87	12	15	High-quality	100	0	Siphoviridae	prokaryote
v1d55	67071	82	10	11	High-quality	100	0	unclassified	unclassified
v1d56	169316	180	14	38	High-quality	100	0	unclassified	unclassified
v1d57	56712	56	0	11	High-quality	94.73	0	unclassified	unclassified
v1d58	53348	50	4	20	High-quality	100	0	unclassified	unclassified
v1d59	31830	44	1	8	Medium-quality	56.65	0	unclassified	unclassified
v1d5a	173519	169	3	59	High-quality	100	0	unclassified	unclassified
v1d5b	9186	14	0	0	High-quality	100	0	Inoviridae	prokaryote

v1d5c	5461	11	0	0	Complete	100	0	Inoviridae	prokaryote
v1d5d	141120	138	5	44	High-quality	93.75	0	unclassified	unclassified
v1d5e	39463	42	1	14	Medium-quality	65.5	0	unclassified	unclassified
v1d5f	32897	43	2	2	Medium-quality	72.92	0	unclassified	unclassified
v1d60	50817	65	15	1	High-quality	99.03	0	unclassified	unclassified
v1d61	46138	66	4	2	Medium-quality	59.45	0	unclassified	unclassified
v1d62	37786	62	21	0	High-quality	94.42	0	Siphoviridae	prokaryote
v1d63	35749	50	13	0	High-quality	100	0	unclassified	unclassified
v1d64	41079	60	20	2	High-quality	98.71	0	Siphoviridae	prokaryote
v1d65	52229	74	15	2	High-quality	93.1	0	unclassified	unclassified
v1d66	159202	256	39	4	High-quality	94.52	0	unclassified	unclassified
v1d67	77912	99	21	1	High-quality	100	0	Siphoviridae	prokaryote
v1d68	36502	55	14	0	High-quality	91.83	0	Siphoviridae	prokaryote
v1d69	16009	19	4	1	Medium-quality	55.8	0	unclassified	unclassified
v1d6a	43598	50	11	5	Medium-quality	69.12	23.88	unclassified	unclassified
v1d6b	51035	55	25	1	High-quality	100	0	Siphoviridae	prokaryote
v1d6c	44337	64	18	2	Medium-quality	77.08	0	unclassified	unclassified
v1d6d	63084	107	22	0	High-quality	100	0	unclassified	unclassified
v1d6e	21846	26	8	1	High-quality	100	0	unclassified	unclassified
v1d6f	44262	60	21	0	High-quality	100	0	Siphoviridae	prokaryote
v1d70	35279	46	19	0	High-quality	93.6	0	Siphoviridae	prokaryote
v1d71	57773	83	22	3	High-quality	100	0	Myoviridae	prokaryote
v1d72	155829	212	38	6	High-quality	100	0	unclassified	unclassified
v1d73	42007	66	41	0	High-quality	91.95	0	Siphoviridae	prokaryote
v1d74	52432	85	17	0	High-quality	100	0	unclassified	unclassified
v1d75	37307	60	18	0	High-quality	100	0	Siphoviridae	prokaryote
v1d76	64742	91	29	0	High-quality	100	0	unclassified	unclassified
v1d77	37518	55	21	2	Medium-quality	84.45	0	unclassified	unclassified
v1d78	29564	43	2	0	Medium-quality	64.5	0	unclassified	unclassified
v1d79	156360	237	37	3	High-quality	94.98	0	unclassified	unclassified
v1d7a	34680	48	18	1	High-quality	95.54	0	Myoviridae	prokaryote
v1d7b	31221	50	26	0	Medium-quality	66.09	0	Myoviridae	prokaryote
v1d7c	62122	100	20	0	High-quality	99.43	0	Quimbyviridae	prokaryote
v1d7d	54404	87	17	0	High-quality	100	0	Myoviridae	prokaryote
v1d7e	38874	57	21	1	High-quality	100	0	Siphoviridae	prokaryote
v1d7f	36921	50	19	0	High-quality	96.07	0	unclassified	unclassified
v1d80	208895	215	69	107	High-quality	100	65.57	Myoviridae	prokaryote
v1d81	47944	67	9	1	Medium-quality	54.72	0	Quimbyviridae	prokaryote
v1d82	106273	141	27	2	High-quality	100	0	Siphoviridae	prokaryote
v1d83	61533	105	18	1	High-quality	98.87	0	unclassified	unclassified

v1d84	92611	137	42	2	High-quality	100	0	Myoviridae	prokaryote
v1d85	158550	244	38	11	High-quality	97.57	0	unclassified	unclassified
v1d86	35910	51	17	1	High-quality	97.75	0	unclassified	unclassified
v1d87	48766	82	14	3	Medium-quality	66.38	0	unclassified	unclassified
v1d88	52670	58	13	1	Medium-quality	80.55	0	Siphoviridae	prokaryote
v1d89	37171	49	25	1	High-quality	100	0	Siphoviridae	prokaryote
v1d8a	56424	99	18	0	High-quality	100	0	unclassified	unclassified
v1d8b	16080	20	3	0	Medium-quality	56.05	0	unclassified	unclassified
v1d8c	46689	76	15	1	Medium-quality	77.72	0	unclassified	unclassified
v1d8d	83592	124	42	2	High-quality	100	0	Myoviridae	prokaryote
v1d8e	55441	72	9	7	High-quality	95.22	0	unclassified	unclassified
v1d8f	33736	50	19	0	High-quality	100	0	Siphoviridae	prokaryote
v1d90	40005	66	18	1	High-quality	96.48	0	unclassified	unclassified
v1d91	46721	61	14	0	Medium-quality	74.96	0	unclassified	unclassified
v1d92	110019	117	2	36	Medium-quality	78.27	0	unclassified	unclassified
v1d93	118977	126	1	43	High-quality	100	0	unclassified	unclassified
v1d94	5358	10	0	0	Medium-quality	86.33	0	unclassified	unclassified
v1d95	121717	124	3	36	High-quality	90.58	0	unclassified	unclassified
v1d96	28095	28	1	8	Medium-quality	51.65	0	unclassified	unclassified
v1d97	82338	84	11	13	High-quality	92.02	0	unclassified	unclassified
v1d98	28615	29	1	9	Medium-quality	58.66	0	unclassified	unclassified
v1d99	96118	96	6	24	Medium-quality	76.06	0	unclassified	unclassified
v1d9a	58055	60	0	18	Medium-quality	76.19	0	unclassified	unclassified
v1d9b	36828	38	1	6	Complete	100	0	unclassified	unclassified
v1d9c	35599	30	0	5	Medium-quality	59.46	0	unclassified	unclassified
v1d9d	37147	44	0	11	Medium-quality	62.05	0	unclassified	unclassified
v1d9e	46285	55	7	1	Medium-quality	53.18	0	Quimbyviridae	prokaryote
v1d9f	38384	65	18	2	High-quality	91.03	0	unclassified	unclassified
v1da0	47052	69	11	1	Medium-quality	76.95	0	unclassified	unclassified
v1da1	52454	90	20	0	High-quality	100	0	unclassified	unclassified
v1da2	43204	74	27	0	High-quality	97.04	0	unclassified	unclassified
v1da3	31155	56	19	1	High-quality	92.92	0	Siphoviridae	prokaryote
v1da4	43866	71	15	0	High-quality	100	0	unclassified	unclassified
v1da5	44258	51	16	1	High-quality	100	0	Siphoviridae	prokaryote
v1da6	40791	54	12	0	High-quality	100	0	unclassified	unclassified
v1da7	37772	52	29	0	High-quality	91.53	0	Siphoviridae	prokaryote
v1da8	57722	80	42	1	High-quality	100	0	Siphoviridae	prokaryote
v1da9	40998	61	42	0	Medium-quality	86.29	0	Siphoviridae	prokaryote
v1daa	62235	68	14	5	Medium-quality	80.62	0	unclassified	unclassified
v1dab	23508	34	20	0	Medium-quality	57.57	0	Siphoviridae	prokaryote

v1dac	31355	35	13	0	High-quality	94.11	0	Siphoviridae	prokaryote
v1dad	140709	213	26	7	Medium-quality	68.44	0	unclassified	unclassified
v1dae	113646	177	18	5	Medium-quality	71.82	0	unclassified	unclassified
v1daf	28435	42	31	0	Medium-quality	82.22	0	Siphoviridae	prokaryote
v1db0	40290	53	12	0	High-quality	98.8	0	unclassified	unclassified
v1db1	152658	236	41	5	High-quality	100	0	unclassified	unclassified
v1db2	37984	56	17	1	High-quality	96.58	0	unclassified	unclassified
v1db3	46071	60	31	0	High-quality	100	0	Myoviridae	prokaryote
v1db4	31311	51	24	0	Medium-quality	79.87	0	unclassified	unclassified
v1db5	96585	104	6	13	High-quality	100	0	unclassified	unclassified
v1db6	31713	51	39	0	Medium-quality	80.97	0	Podoviridae	prokaryote
v1db7	33649	44	14	0	High-quality	93.61	0	unclassified	unclassified
v1db8	41672	69	21	0	High-quality	100	0	Siphoviridae	prokaryote
v1db9	35700	52	17	0	Medium-quality	86.59	0	Siphoviridae	prokaryote
v1dba	51369	64	24	2	Medium-quality	88.19	0	Siphoviridae	prokaryote
v1dbb	44308	59	20	1	High-quality	100	0	Siphoviridae	prokaryote
v1dbc	36002	57	17	0	Medium-quality	89.72	0	Siphoviridae	prokaryote
v1dbd	38732	49	16	0	High-quality	93.9	0	unclassified	unclassified
v1dbe	45481	69	20	5	High-quality	100	0	Siphoviridae	prokaryote
v1dbf	69238	85	19	7	High-quality	100	0	unclassified	unclassified
v1dc0	55882	99	12	2	Medium-quality	50.12	0	unclassified	unclassified
v1dc1	34919	50	32	0	Medium-quality	81.48	0	Siphoviridae	prokaryote
v1dc2	34286	49	30	0	Medium-quality	86.79	0	Siphoviridae	prokaryote
v1dc3	42786	63	37	0	Medium-quality	88.99	0	Siphoviridae	prokaryote
v1dc4	44634	65	18	1	High-quality	100	0	unclassified	unclassified
v1dc5	51770	77	24	0	High-quality	100	0	Myoviridae	prokaryote
v1dc6	47216	51	4	2	High-quality	100	0	unclassified	unclassified
v1dc7	45215	74	27	3	High-quality	100	0	unclassified	unclassified
v1dc8	24994	30	26	0	Medium-quality	60.11	0	Myoviridae	prokaryote
v1dc9	9078	6	1	0	Medium-quality	62.88	0	unclassified	unclassified
v1dca	37107	47	17	0	High-quality	98.23	0	Siphoviridae	prokaryote
v1dcb	39515	66	19	0	High-quality	99.11	0	unclassified	unclassified
v1dcc	40472	61	13	2	High-quality	100	0	unclassified	unclassified
v1dcd	66144	54	13	5	High-quality	100	0	unclassified	unclassified
v1dce	59288	63	8	4	High-quality	98.25	0	unclassified	unclassified
v1dcf	34467	34	1	6	Medium-quality	51.56	0	unclassified	unclassified
v1dd0	74233	80	3	12	High-quality	100	0	unclassified	unclassified
v1dd1	62343	65	8	20	Medium-quality	80.52	0	unclassified	unclassified
v1dd2	44477	66	29	0	Medium-quality	89.98	0	unclassified	unclassified
v1dd3	34511	51	17	0	High-quality	97.3	0	Siphoviridae	prokaryote

v1dd4	40053	53	8	2	High-quality	96.18	0	unclassified	unclassified
v1dd5	61052	110	25	0	Complete	100	0	unclassified	unclassified
v1dd6	68471	108	28	1	High-quality	100	0	unclassified	unclassified
v1dd7	26634	31	3	1	Medium-quality	69.92	0	unclassified	unclassified
v1dd8	38118	50	9	1	High-quality	96.69	0	unclassified	unclassified
v1dd9	35246	59	23	0	High-quality	99.46	0	unclassified	unclassified
v1dda	59313	69	15	3	Medium-quality	82.36	0	unclassified	unclassified
v1ddb	18221	31	16	0	Medium-quality	53.37	0	Siphoviridae	prokaryote
v1ddc	35854	57	51	0	Medium-quality	72.24	0	Siphoviridae	prokaryote
v1ddd	44135	66	33	0	High-quality	97.06	0	Myoviridae	prokaryote
v1dde	27146	36	2	9	Medium-quality	51.07	0	unclassified	unclassified
v1ddf	31429	31	1	10	Medium-quality	73.67	0	unclassified	unclassified
v1de0	62820	70	5	7	Medium-quality	76.16	0	unclassified	unclassified
v1de1	8674	15	0	0	High-quality	100	0	Inoviridae	prokaryote
v1de2	33913	45	2	4	Medium-quality	88.9	0	unclassified	unclassified
v1de3	40875	50	3	3	Medium-quality	89.16	0	unclassified	unclassified
v1de4	6279	9	0	1	High-quality	100	0	unclassified	unclassified
v1de5	72865	125	23	4	Medium-quality	77.76	0	unclassified	unclassified
v1de6	41500	46	20	0	High-quality	99.3	0	Siphoviridae	prokaryote
v1de7	65795	74	9	0	High-quality	100	0	unclassified	unclassified
v1de8	34616	50	5	2	Medium-quality	53.08	0	unclassified	unclassified
v1de9	37545	50	30	0	Medium-quality	86.18	0	Myoviridae	prokaryote
v1dea	38763	40	20	0	High-quality	93.79	0	Siphoviridae	prokaryote
v1deb	49074	64	17	2	High-quality	100	0	unclassified	unclassified
v1dec	159543	244	37	6	Medium-quality	89.81	0	unclassified	unclassified
v1ded	34477	48	18	1	High-quality	100	0	Siphoviridae	prokaryote
v1dee	48878	65	41	0	High-quality	100	0	Siphoviridae	prokaryote
v1def	36444	39	19	1	Medium-quality	88.13	0	Autographiviridae	prokaryote
v1df0	13367	19	5	1	High-quality	100	0	unclassified	unclassified
v1df1	22122	31	22	1	Medium-quality	68.05	0	Myoviridae	prokaryote
v1df2	110454	168	30	4	High-quality	100	0	unclassified	unclassified
v1df3	103537	75	1	23	Medium-quality	88.47	0	unclassified	unclassified
v1df4	109582	124	5	39	Medium-quality	78.4	0	unclassified	unclassified
v1df5	21956	28	17	0	Medium-quality	57.28	0	Siphoviridae	prokaryote
v1df6	38131	60	29	1	Medium-quality	87.08	0	Myoviridae	prokaryote
v1df7	21279	26	11	0	Medium-quality	57.43	0	Siphoviridae	prokaryote
v1df8	39263	57	48	1	High-quality	98.23	0	Podoviridae	prokaryote
v1df9	58970	106	21	1	High-quality	100	0	unclassified	unclassified
v1dfa	40835	60	17	1	Medium-quality	60.02	0	unclassified	unclassified
v1dfb	33800	42	22	0	Medium-quality	82.68	0	Siphoviridae	prokaryote

v1dfc	34773	49	3	0	High-quality	96.61	0	unclassified	unclassified
v1dfd	27897	31	21	0	Medium-quality	60.5	0	Siphoviridae	prokaryote
v1dfe	39741	63	17	0	High-quality	98.77	0	unclassified	unclassified
v1dff	18878	28	3	0	High-quality	99.06	0	unclassified	unclassified
v1e00	32076	23	5	1	Medium-quality	50.48	0	unclassified	unclassified
v1e01	90501	98	3	15	High-quality	100	0	unclassified	unclassified
v1e02	157739	180	6	58	High-quality	92.61	0	unclassified	unclassified
v1e03	28100	38	2	3	Medium-quality	69.98	0	unclassified	unclassified
v1e04	37535	25	1	7	Medium-quality	61.62	0	unclassified	unclassified
v1e05	46640	53	7	2	High-quality	100	0	unclassified	unclassified
v1e06	42097	53	38	0	High-quality	100	0	Podoviridae	prokaryote
v1e07	152600	208	41	7	High-quality	96.21	0	unclassified	unclassified
v1e08	54874	79	16	0	High-quality	98.05	0	unclassified	unclassified
v1e09	35230	50	16	1	High-quality	91.2	0	Siphoviridae	prokaryote
v1e0a	20562	36	8	0	Medium-quality	53.13	0	Siphoviridae	prokaryote
v1e0b	10232	13	2	1	Medium-quality	51.18	0	Siphoviridae	prokaryote
v1e0c	22876	28	21	0	Medium-quality	57.2	0	Podoviridae	prokaryote
v1e0d	47490	75	37	1	High-quality	100	0	Siphoviridae	prokaryote
v1e0e	61076	93	35	1	High-quality	100	0	Siphoviridae	prokaryote
v1e0f	61552	89	14	1	High-quality	100	0	unclassified	unclassified
v1e10	37748	55	29	0	High-quality	91.65	0	Siphoviridae	prokaryote
v1e11	36979	55	25	0	High-quality	91.86	0	Siphoviridae	prokaryote
v1e12	55719	77	15	0	High-quality	91.8	0	unclassified	unclassified
v1e13	59230	89	32	1	High-quality	99.15	0	Myoviridae	prokaryote
v1e14	44713	82	22	1	High-quality	100	0	unclassified	unclassified
v1e15	159983	207	45	6	High-quality	100	0	unclassified	unclassified
v1e16	65527	97	11	2	Medium-quality	74.55	0	Quimbyviridae	prokaryote
v1e17	40643	48	7	1	Medium-quality	72.52	0	unclassified	unclassified
v1e18	36766	45	7	0	High-quality	94.9	0	unclassified	unclassified
v1e19	56918	57	3	5	High-quality	100	0	unclassified	unclassified
v1e1a	42725	47	1	13	High-quality	100	0	unclassified	unclassified
v1e1b	144097	134	8	21	High-quality	100	0	unclassified	unclassified
v1e1c	19683	29	1	10	Medium-quality	51.61	0	unclassified	unclassified
v1e1d	24923	25	1	3	Medium-quality	51.07	0	unclassified	unclassified
v1e1e	6761	11	0	0	High-quality	100	0	unclassified	unclassified
v1e1f	32240	29	7	0	Medium-quality	84.43	0	unclassified	unclassified
v1e20	143031	147	12	40	High-quality	100	0	unclassified	unclassified
v1e21	27842	46	11	0	Medium-quality	85.82	0	unclassified	unclassified
v1e22	126160	197	52	10	High-quality	100	0	unclassified	unclassified
v1e23	134273	209	30	1	Medium-quality	79.99	0	unclassified	unclassified

v1e24	38881	52	37	0	Medium-quality	68.77	0	Myoviridae	prokaryote
v1e25	38768	48	31	0	High-quality	96.25	0	Podoviridae	prokaryote
v1e26	46389	76	30	0	Medium-quality	74.24	0	Myoviridae	prokaryote
v1e27	49946	55	7	4	Medium-quality	83.75	0	unclassified	unclassified
v1e28	31076	45	24	3	Medium-quality	77.04	0	Siphoviridae	prokaryote
v1e29	30989	31	15	0	High-quality	91.09	0	Siphoviridae	prokaryote
v1e2a	56464	44	7	2	High-quality	100	0	unclassified	unclassified
v1e2b	38893	57	19	0	Medium-quality	77.07	0	unclassified	unclassified
v1e2c	20180	18	1	5	High-quality	100	0	unclassified	unclassified
v1e2d	103633	110	5	27	High-quality	100	0	unclassified	unclassified
v1e2e	35836	55	22	0	Medium-quality	86.8	0	Siphoviridae	prokaryote
v1e2f	40577	53	29	0	High-quality	94.12	0	Myoviridae	prokaryote
v1e30	58610	58	10	2	Medium-quality	76.54	0	unclassified	unclassified
v1e31	54235	73	47	0	High-quality	100	0	Siphoviridae	prokaryote
v1e32	55429	63	23	3	Medium-quality	87.6	0	Siphoviridae	prokaryote
v1e33	32352	41	14	0	Medium-quality	82.48	0	Siphoviridae	prokaryote
v1e34	21750	21	13	2	Medium-quality	50.04	0	Siphoviridae	prokaryote
v1e35	38662	48	9	1	Medium-quality	79.88	0	unclassified	unclassified
v1e36	37095	57	12	0	Medium-quality	61.77	0	unclassified	unclassified
v1e37	40913	50	10	1	High-quality	100	0	unclassified	unclassified
v1e38	218302	221	13	67	High-quality	100	0	unclassified	unclassified
v1e39	39848	31	0	8	Medium-quality	55.04	0	unclassified	unclassified
v1e3a	95814	119	17	20	High-quality	100	0	unclassified	unclassified
v1e3b	26302	26	0	5	Medium-quality	53.53	0	unclassified	unclassified
v1e3c	40321	49	1	1	Medium-quality	54.18	0	unclassified	unclassified
v1e3d	37706	51	20	0	High-quality	90.72	0	unclassified	unclassified
v1e3e	34151	34	8	0	Medium-quality	81.76	0	Siphoviridae	prokaryote
v1e3f	38317	60	18	0	High-quality	100	0	Myoviridae	prokaryote
v1e40	40956	63	18	0	High-quality	97.48	0	Siphoviridae	prokaryote
v1e41	43137	52	14	5	Medium-quality	68.53	36.36	Siphoviridae	prokaryote
v1e42	25312	38	7	1	Medium-quality	64.78	0	Siphoviridae	prokaryote
v1e43	21582	33	10	0	Medium-quality	55.55	0	Siphoviridae	prokaryote
v1e44	26419	30	21	0	Medium-quality	56.98	0	Siphoviridae	prokaryote
v1e45	40414	64	51	0	High-quality	100	0	Podoviridae	prokaryote
v1e46	66213	90	30	7	High-quality	100	0	Myoviridae	prokaryote
v1e47	62711	58	2	18	Medium-quality	74.67	0	unclassified	unclassified
v1e48	98468	104	3	27	High-quality	100	0	unclassified	unclassified
v1e49	49451	55	5	9	High-quality	100	0	unclassified	unclassified
v1e4a	24566	28	2	2	Medium-quality	62.37	0	Siphoviridae	prokaryote
v1e4b	72100	92	5	17	Medium-quality	64.11	0	unclassified	unclassified

v1e4c	6951	14	0	0	High-quality	100	0	Inoviridae	prokaryote
v1e4d	42993	65	26	0	High-quality	90.89	0	Myoviridae	prokaryote
v1e4e	30696	39	27	1	High-quality	93.56	0	Myoviridae	prokaryote
v1e4f	10557	16	6	3	High-quality	100	25.73	Microviridae	prokaryote
v1e50	37712	55	23	0	High-quality	90.57	0	Siphoviridae	prokaryote
v1e51	45760	66	3	1	High-quality	100	0	unclassified	unclassified
v1e52	41440	66	35	0	High-quality	96.99	0	Myoviridae	prokaryote
v1e53	36223	54	13	1	High-quality	100	0	unclassified	unclassified
v1e54	40405	58	25	0	Medium-quality	81.77	0	Myoviridae	prokaryote
v1e55	37766	46	7	2	Medium-quality	86.12	0	unclassified	unclassified
v1e56	51522	55	14	1	Medium-quality	61.85	0	Quimbyviridae	prokaryote
v1e57	12420	19	5	1	High-quality	100	0	unclassified	unclassified
v1e58	38983	61	46	0	High-quality	96.5	0	Podoviridae	prokaryote
v1e59	36217	51	13	0	Medium-quality	71.53	0	unclassified	unclassified
v1e5a	47317	65	17	3	High-quality	100	0	unclassified	unclassified
v1e5b	36877	60	17	0	High-quality	100	0	unclassified	unclassified
v1e5c	30093	43	12	1	Medium-quality	55.27	0	Siphoviridae	prokaryote
v1e5d	53792	52	7	7	Medium-quality	88.07	0	unclassified	unclassified
v1e5e	23274	31	1	5	Medium-quality	58.82	0	unclassified	unclassified
v1e5f	58899	68	1	18	Medium-quality	70.13	0	unclassified	unclassified
v1e60	10946	20	0	0	Medium-quality	52.56	0	unclassified	unclassified
v1e61	59584	53	6	7	High-quality	100	0	unclassified	unclassified
v1e62	9043	10	0	3	Medium-quality	76.15	0	unclassified	unclassified
v1e63	40115	43	1	14	Medium-quality	60.7	0	unclassified	unclassified
v1e64	110849	135	7	1	High-quality	100	0	unclassified	unclassified
v1e65	36441	51	14	1	High-quality	100	0	Siphoviridae	prokaryote
v1e66	43945	64	26	0	High-quality	97.95	0	Myoviridae	prokaryote
v1e67	18298	22	4	1	Medium-quality	63.78	0	unclassified	unclassified
v1e68	64651	101	38	0	High-quality	100	0	Siphoviridae	prokaryote
v1e69	30764	40	22	1	Medium-quality	87.02	0	Siphoviridae	prokaryote
v1e6a	33847	48	17	0	High-quality	92.43	0	Siphoviridae	prokaryote
v1e6b	34428	42	10	2	Medium-quality	86.44	0	Siphoviridae	prokaryote
v1e6c	91669	117	11	8	High-quality	100	0	unclassified	unclassified
v1e6d	33530	39	8	3	Medium-quality	57.48	0	unclassified	unclassified
v1e6e	43657	57	26	1	Complete	100	0	unclassified	unclassified
v1e6f	80538	130	26	3	Complete	100	0	unclassified	unclassified
v1e70	34431	52	5	2	Medium-quality	52.41	0	unclassified	unclassified
v1e71	21237	33	8	2	Medium-quality	52.62	0	Siphoviridae	prokaryote
v1e72	32020	43	30	1	High-quality	94.97	0	Myoviridae	prokaryote
v1e73	38914	51	28	1	High-quality	99.86	0	Siphoviridae	prokaryote

v1e74	42113	49	18	1	High-quality	100	0	Autographiviridae	prokaryote
v1e75	59122	76	12	2	Medium-quality	78.59	0	unclassified	unclassified
v1e76	32836	43	3	2	Medium-quality	75.9	0	unclassified	unclassified
v1e77	25856	29	3	2	Medium-quality	62.11	0	unclassified	unclassified
v1e78	16907	17	7	0	Medium-quality	52.04	0	unclassified	unclassified
v1e79	45925	72	34	2	Complete	100	0	Myoviridae	prokaryote
v1e7a	12634	18	11	0	Medium-quality	85.77	0	Siphoviridae	prokaryote
v1e7b	84846	114	13	5	High-quality	100	0	unclassified	unclassified
v1e7c	55810	70	14	0	High-quality	91.18	0	unclassified	unclassified
v1e7d	36894	49	29	1	High-quality	100	0	Siphoviridae	prokaryote
v1e7e	16110	18	9	0	Medium-quality	70.08	0	Salasmaviridae	prokaryote
v1e7f	33478	39	17	0	Medium-quality	86.26	0	Siphoviridae	prokaryote
v1e80	78907	107	9	6	Medium-quality	89.64	0	Quimbyviridae	prokaryote
v1e81	53383	91	20	1	Complete	100	0	unclassified	unclassified
v1e82	19990	40	13	0	Medium-quality	56.9	0	Siphoviridae	prokaryote
v1e83	30617	55	6	0	Medium-quality	51.28	0	Quimbyviridae	prokaryote
v1e84	23886	28	6	0	Medium-quality	51.58	0	Siphoviridae	prokaryote
v1e85	38246	54	15	1	Medium-quality	77.34	0	unclassified	unclassified
v1e86	46605	65	12	1	High-quality	100	0	unclassified	unclassified
v1e87	45145	68	17	0	High-quality	98.05	0	Siphoviridae	prokaryote
v1e88	26861	29	19	0	Medium-quality	58.38	0	Siphoviridae	prokaryote
v1e89	13316	21	6	1	High-quality	100	0	Siphoviridae	prokaryote
v1e8a	19968	26	11	0	Medium-quality	57.96	0	unclassified	unclassified
v1e8b	28005	42	14	1	Medium-quality	64.63	0	unclassified	unclassified
v1e8c	27021	30	12	0	Medium-quality	60.89	0	Siphoviridae	prokaryote
v1e8d	13294	16	4	0	Complete	100	0	unclassified	unclassified
v1e8e	99598	141	28	1	Medium-quality	61.69	0	unclassified	unclassified
v1e8f	45305	60	27	2	High-quality	94.46	0	Myoviridae	prokaryote
v1e90	41319	75	18	0	Complete	100	0	Siphoviridae	prokaryote
v1e91	24402	24	3	1	Medium-quality	51.84	0	unclassified	unclassified
v1e92	38321	50	25	3	Medium-quality	89.1	0	Myoviridae	prokaryote
v1e93	132606	136	11	9	High-quality	100	0	unclassified	unclassified
v1e94	36165	58	26	0	High-quality	94.58	0	unclassified	unclassified
v1e95	103110	137	27	5	High-quality	100	0	unclassified	unclassified
v1e96	29200	53	33	0	Medium-quality	77.85	0	Siphoviridae	prokaryote
v1e97	35818	55	23	0	Complete	100	0	Siphoviridae	prokaryote
v1e98	35935	37	3	1	Medium-quality	68.79	0	unclassified	unclassified
v1e99	34500	54	16	0	High-quality	100	0	Siphoviridae	prokaryote
v1e9a	25989	37	25	0	Medium-quality	67.63	0	Siphoviridae	prokaryote
v1e9b	162979	207	23	4	Complete	100	0	unclassified	unclassified

v1e9c	16297	20	5	0	Medium-quality	54.25	0	unclassified	unclassified
v1e9d	84101	147	22	1	Complete	100	0	Quimbyviridae	prokaryote
v1e9e	16412	19	5	0	Medium-quality	54.63	0	unclassified	unclassified
v1e9f	26018	46	12	2	Medium-quality	62.24	0	Siphoviridae	prokaryote
v1ea0	154777	199	15	2	Complete	100	0	unclassified	unclassified
v1ea1	49831	64	12	2	High-quality	100	0	unclassified	unclassified
v1ea2	162007	245	27	7	High-quality	96.05	0	unclassified	unclassified
v1ea3	186320	217	24	6	Complete	100	0	unclassified	unclassified
v1ea4	40382	56	22	3	High-quality	100	0	Siphoviridae	prokaryote
v1ea5	32244	37	6	0	Medium-quality	65.13	0	unclassified	unclassified
v1ea6	38516	60	21	0	Complete	100	0	unclassified	unclassified
v1ea7	62792	83	16	7	High-quality	100	0	unclassified	unclassified
v1ea8	17562	27	6	0	Complete	100	0	unclassified	unclassified
v1ea9	17030	19	7	0	Medium-quality	88.47	0	Salasmaviridae	prokaryote
v1eaa	61569	92	14	9	Medium-quality	83.28	0	Quimbyviridae	prokaryote
v1eab	15739	22	7	0	Medium-quality	62.53	0	unclassified	unclassified
v1eac	37904	60	8	0	Medium-quality	66.29	0	unclassified	unclassified
v1ead	58169	98	13	2	Complete	100	0	unclassified	unclassified
v1eae	62435	76	8	1	Complete	100	0	unclassified	unclassified
v1eaf	73340	96	10	7	Medium-quality	86.03	0	unclassified	unclassified
v1eb0	25485	38	13	0	Medium-quality	71.44	0	Siphoviridae	prokaryote
v1eb1	50695	60	24	1	High-quality	100	0	Siphoviridae	prokaryote
v1eb2	212406	334	37	7	Complete	100	0	unclassified	unclassified
v1eb3	23712	25	4	2	Medium-quality	63.26	0	unclassified	unclassified
v1eb4	79673	121	25	2	Complete	100	0	unclassified	unclassified
v1eb5	42986	72	25	3	Complete	100	0	unclassified	unclassified
v1eb6	78082	128	14	1	High-quality	91.93	0	unclassified	unclassified
v1eb7	40132	56	26	0	Medium-quality	66.64	0	Siphoviridae	prokaryote
v1eb8	31593	28	5	4	High-quality	100	0	unclassified	unclassified
v1eb9	40511	46	44	0	Complete	100	0	Autographiviridae	prokaryote
v1eba	45298	62	8	1	Complete	100	0	Siphoviridae	prokaryote
v1ebb	39831	68	20	0	High-quality	99.44	0	Siphoviridae	prokaryote
v1ebc	35668	41	11	8	Medium-quality	66.58	0	Siphoviridae	prokaryote
v1ebd	56328	94	14	2	Complete	100	0	unclassified	unclassified
v1ebe	61794	70	9	6	High-quality	100	0	unclassified	unclassified
v1ebf	43973	75	21	0	Complete	100	0	unclassified	unclassified
v1ec0	40242	42	13	0	Medium-quality	73.45	0	unclassified	unclassified
v1ec1	59037	108	20	0	High-quality	100	0	unclassified	unclassified
v1ec2	296209	345	46	9	High-quality	100	0	unclassified	unclassified
v1ec3	58011	97	14	1	High-quality	100	0	unclassified	unclassified

v1ec4	31996	46	14	2	Medium-quality	89.65	0	unclassified	unclassified
v1ec5	36854	46	6	2	Medium-quality	66.86	0	unclassified	unclassified
v1ec6	45717	70	21	0	High-quality	100	0	unclassified	unclassified
v1ec7	16571	23	7	0	Medium-quality	70.18	0	unclassified	unclassified
v1ec8	15344	15	6	0	Medium-quality	51.65	0	unclassified	unclassified
v1ec9	45857	81	24	1	High-quality	100	0	unclassified	unclassified
v1eca	15857	17	5	0	Medium-quality	52.78	0	unclassified	unclassified
v1ecb	48639	62	12	1	High-quality	100	0	unclassified	unclassified
v1ecc	64976	83	20	0	Complete	100	0	unclassified	unclassified
v1ecd	46083	71	17	0	Complete	100	0	unclassified	unclassified
v1ece	39625	57	8	5	Medium-quality	69.82	0	unclassified	unclassified
v1ecf	48540	73	17	1	Medium-quality	57.15	0	unclassified	unclassified
v1ed0	40634	67	11	1	High-quality	99.57	0	unclassified	unclassified
v1ed1	33967	50	22	1	High-quality	94.17	0	unclassified	unclassified
v1ed2	26171	33	23	0	Medium-quality	68.83	0	Siphoviridae	prokaryote
v1ed3	33391	60	26	0	Complete	100	0	Siphoviridae	prokaryote
v1ed4	42355	66	18	2	Medium-quality	88.7	0	Myoviridae	prokaryote
v1ed5	28398	45	3	1	Medium-quality	58.67	0	unclassified	unclassified
v1ed6	15525	23	7	0	Medium-quality	68.4	0	unclassified	unclassified
v1ed7	53749	70	11	4	High-quality	100	0	unclassified	unclassified
v1ed8	78280	124	37	1	High-quality	100	0	unclassified	unclassified
v1ed9	258447	284	43	14	High-quality	98.23	0	unclassified	unclassified
v1eda	40766	59	17	0	High-quality	92.19	0	Siphoviridae	prokaryote
v1edb	28451	37	25	0	Medium-quality	59.39	0	Siphoviridae	prokaryote
v1edc	29505	36	8	1	Medium-quality	58.01	0	unclassified	unclassified
v1edd	39031	66	22	0	High-quality	97.53	0	Siphoviridae	prokaryote
v1ede	16737	23	9	0	Medium-quality	87.45	0	unclassified	unclassified
v1edf	85459	79	10	4	Medium-quality	77.11	0	unclassified	unclassified
v1ee0	42406	68	18	0	Medium-quality	66.27	0	unclassified	unclassified
v1ee1	43048	58	19	2	High-quality	100	0	unclassified	unclassified
v1ee2	16209	23	8	0	Medium-quality	86.58	0	unclassified	unclassified
v1ee3	40545	74	21	0	Complete	100	0	Siphoviridae	prokaryote
v1ee4	47156	66	15	0	Complete	100	0	Siphoviridae	prokaryote
v1ee5	28154	40	17	0	Medium-quality	69.51	0	Siphoviridae	prokaryote
v1ee6	47105	81	15	0	Complete	100	0	unclassified	unclassified
v1ee7	45314	71	22	0	High-quality	100	0	unclassified	unclassified
v1ee8	5304	10	4	0	High-quality	97.81	0	Microviridae	prokaryote
v1ee9	62436	88	13	2	High-quality	100	0	unclassified	unclassified
v1eea	73419	98	14	0	High-quality	92.75	0	Flandersviridae	prokaryote
v1eeb	40482	49	5	0	Medium-quality	54.64	0	unclassified	unclassified

v1eec	50205	70	7	3	Complete	100	0	unclassified	unclassified
v1eed	44291	67	14	1	High-quality	97.73	0	unclassified	unclassified
v1eee	124531	166	13	10	High-quality	100	0	Quimbyviridae	prokaryote
v1eef	15694	17	7	0	Medium-quality	82.62	0	Salasmaviridae	prokaryote
v1ef0	31133	49	13	1	Complete	100	0	unclassified	unclassified
v1ef1	36648	56	18	0	Complete	100	0	unclassified	unclassified
v1ef2	37377	44	5	3	Medium-quality	62.43	0	unclassified	unclassified
v1ef3	42095	65	17	1	Medium-quality	58.35	0	unclassified	unclassified
v1ef4	15757	26	6	0	High-quality	91.76	0	unclassified	unclassified
v1ef5	60723	94	23	3	Medium-quality	75.36	0	unclassified	unclassified
v1ef6	64426	78	14	0	High-quality	100	0	unclassified	unclassified
v1ef7	60097	85	13	0	Medium-quality	61	0	Podoviridae_crAss-like	prokaryote
v1ef8	16057	21	6	0	Medium-quality	72.83	0	unclassified	unclassified
v1ef9	30701	51	19	0	Medium-quality	77.17	0	Siphoviridae	prokaryote
v1efa	61741	100	33	0	High-quality	100	0	Siphoviridae	prokaryote
v1efb	12819	14	4	0	High-quality	95.96	0	unclassified	unclassified
v1efc	98033	83	8	0	Complete	100	0	Podoviridae_crAss-like	prokaryote
v1efd	46521	69	26	2	Complete	100	0	Myoviridae	prokaryote
v1efe	66502	93	11	2	High-quality	100	0	unclassified	unclassified
v1eff	179042	198	32	4	Complete	100	0	unclassified	unclassified
v1f00	37621	57	19	1	Complete	100	0	Siphoviridae	prokaryote
v1f01	40772	63	16	1	Complete	100	0	unclassified	unclassified
v1f02	41537	68	22	0	Complete	100	0	Siphoviridae	prokaryote
v1f03	43378	78	12	0	Complete	100	0	unclassified	unclassified
v1f04	63102	63	7	0	Complete	100	0	unclassified	unclassified
v1f05	22457	38	10	1	Medium-quality	58.79	0	unclassified	unclassified
v1f06	43041	74	21	0	Complete	100	0	Siphoviridae	prokaryote
v1f07	33555	45	9	0	Medium-quality	55.65	0	unclassified	unclassified
v1f08	30187	39	22	0	Medium-quality	50.17	0	Siphoviridae	prokaryote
v1f09	13019	25	2	1	High-quality	100	0	unclassified	unclassified
v1f0a	33648	43	23	1	Medium-quality	85.75	0	Siphoviridae	prokaryote
v1f0b	96265	165	16	1	Complete	100	0	Podoviridae_crAss-like	prokaryote
v1f0c	80595	129	26	3	Complete	100	0	unclassified	unclassified
v1f0d	5524	9	6	0	Complete	100	0	Microviridae	prokaryote
v1f0e	122398	155	34	5	Complete	100	0	unclassified	unclassified
v1f0f	66434	65	4	0	Medium-quality	57.1	0	unclassified	unclassified
v1f10	7832	10	5	0	Medium-quality	56.45	0	unclassified	unclassified
v1f11	16201	20	7	0	Medium-quality	50.85	0	unclassified	unclassified
v1f12	82502	124	17	1	High-quality	100	0	Quimbyviridae	prokaryote
v1f13	60131	80	13	6	High-quality	100	0	Myoviridae	prokaryote

v1f14	60011	99	14	1	Complete	100	0	unclassified	unclassified
v1f15	80525	128	19	3	High-quality	99.86	0	unclassified	unclassified
v1f16	25539	33	23	0	Medium-quality	57.59	0	Siphoviridae	prokaryote
v1f17	31766	40	10	4	High-quality	100	0	unclassified	unclassified
v1f18	28121	38	20	0	Medium-quality	80.25	0	Siphoviridae	prokaryote
v1f19	24966	36	11	0	Medium-quality	61.77	0	unclassified	unclassified
v1f1a	37255	55	28	0	Medium-quality	69.2	0	Myoviridae	prokaryote
v1f1b	60630	72	11	0	High-quality	96.92	0	unclassified	unclassified
v1f1c	42487	79	14	0	Complete	100	0	unclassified	unclassified
v1f1d	76809	118	17	4	High-quality	100	0	unclassified	unclassified
v1f1e	54760	86	22	0	Complete	100	0	Myoviridae	prokaryote
v1f1f	38242	67	21	1	High-quality	97.28	0	Siphoviridae	prokaryote
v1f20	65806	96	17	1	High-quality	92.68	0	unclassified	unclassified
v1f21	37464	49	20	1	Medium-quality	63.3	0	unclassified	unclassified
v1f22	17494	22	11	0	Medium-quality	52.51	0	Siphoviridae	prokaryote
v1f23	31541	36	10	0	High-quality	100	0	unclassified	unclassified
v1f24	17134	27	8	0	High-quality	99.52	0	unclassified	unclassified
v1f25	45005	76	22	0	Complete	100	0	unclassified	unclassified
v1f26	45116	71	24	0	High-quality	100	0	unclassified	unclassified
v1f27	73166	104	7	2	Medium-quality	75.8	0	unclassified	unclassified
v1f28	42997	59	6	1	High-quality	100	0	unclassified	unclassified
v1f29	30582	51	16	0	Medium-quality	77.43	0	Siphoviridae	prokaryote
v1f2a	27571	27	1	0	Medium-quality	61.85	0	unclassified	unclassified
v1f2b	46471	68	14	0	High-quality	100	0	unclassified	unclassified
v1f2c	61784	84	18	0	Complete	100	0	unclassified	unclassified
v1f2d	34496	55	15	1	Medium-quality	76.73	0	Siphoviridae	prokaryote
v1f2e	39307	61	7	1	High-quality	98.48	0	unclassified	unclassified
v1f2f	5897	5	1	0	High-quality	100	0	Siphoviridae	prokaryote
v1f30	44682	60	10	0	High-quality	100	0	unclassified	unclassified
v1f31	31652	51	15	0	Medium-quality	74.87	0	unclassified	unclassified
v1f32	86891	111	30	3	Medium-quality	65.14	0	unclassified	unclassified
v1f33	56845	80	20	1	High-quality	100	0	Myoviridae	prokaryote
v1f34	39777	78	18	0	Complete	100	0	unclassified	unclassified
v1f35	43916	56	8	1	Complete	100	0	unclassified	unclassified
v1f36	34929	59	20	1	High-quality	100	0	unclassified	unclassified
v1f37	42958	57	22	0	High-quality	99.49	0	unclassified	unclassified
v1f38	33495	51	17	1	Medium-quality	85.72	0	Siphoviridae	prokaryote
v1f39	21770	29	12	0	Medium-quality	73.37	0	unclassified	unclassified
v1f3a	28387	40	15	0	Medium-quality	72.96	0	Siphoviridae	prokaryote
v1f3b	73103	110	21	1	Complete	100	0	unclassified	unclassified

v1f3c	22510	27	14	0	Medium-quality	61.78	0	Siphoviridae	prokaryote
v1f3d	39311	47	11	0	Medium-quality	58.99	0	unclassified	unclassified
v1f3e	40192	60	7	1	Complete	100	0	unclassified	unclassified
v1f3f	26526	37	19	0	Medium-quality	58.68	0	Myoviridae	prokaryote
v1f40	29373	46	10	0	Medium-quality	65.14	0	unclassified	unclassified
v1f41	42496	79	15	1	Complete	100	0	unclassified	unclassified
v1f42	75743	75	10	7	High-quality	94.55	0	unclassified	unclassified
v1f43	21787	24	8	1	Medium-quality	52.63	0	unclassified	unclassified
v1f44	38097	72	14	0	Medium-quality	89.71	0	unclassified	unclassified
v1f45	36675	55	39	0	Medium-quality	67.97	0	Siphoviridae	prokaryote
v1f46	71719	115	18	2	Complete	100	0	unclassified	unclassified
v1f47	30288	30	5	0	Medium-quality	54.41	0	Quimbyviridae	prokaryote
v1f48	29739	55	8	1	Medium-quality	53.24	0	unclassified	unclassified
v1f49	33362	44	29	1	Medium-quality	67.67	0	Myoviridae	prokaryote
v1f4a	82608	127	17	2	High-quality	100	0	unclassified	unclassified
v1f4b	44788	57	11	0	High-quality	100	0	unclassified	unclassified
v1f4c	103023	164	16	1	Complete	100	0	Podoviridae_crAss-like	prokaryote
v1f4d	51567	82	21	0	High-quality	95.27	0	Myoviridae	prokaryote
v1f4e	41537	47	20	2	High-quality	98.94	0	Autographiviridae	prokaryote
v1f4f	63041	79	6	0	Medium-quality	85.17	0	unclassified	unclassified
v1f50	69888	72	8	2	High-quality	100	0	unclassified	unclassified
v1f51	123549	124	12	1	High-quality	100	0	unclassified	unclassified
v1f52	32804	54	15	0	Complete	100	0	unclassified	unclassified
v1f53	56719	81	15	0	Medium-quality	73.15	0	unclassified	unclassified
v1f54	28994	48	18	0	Medium-quality	70.31	0	unclassified	unclassified
v1f55	37842	46	18	0	Medium-quality	59.24	0	unclassified	unclassified
v1f56	27556	38	25	0	Medium-quality	70.22	0	Siphoviridae	prokaryote
v1f57	39310	76	16	3	High-quality	100	0	unclassified	unclassified
v1f58	44848	73	23	2	Complete	100	0	unclassified	unclassified
v1f59	42489	80	18	1	High-quality	100	0	unclassified	unclassified
v1f5a	103011	154	16	1	Complete	100	0	Podoviridae_crAss-like	prokaryote
v1f5b	39999	66	6	4	Medium-quality	53.58	0	unclassified	unclassified
v1f5c	63825	72	8	5	Medium-quality	78.71	0	unclassified	unclassified
v1f5d	35021	51	26	0	Medium-quality	79.87	0	Myoviridae	prokaryote
v1f5e	6265	10	4	0	High-quality	100	0	unclassified	unclassified
v1f5f	104351	157	29	1	Complete	100	0	Podoviridae_crAss-like	prokaryote
v1f60	31948	50	12	0	High-quality	92.85	0	Siphoviridae	prokaryote
v1f61	32532	47	25	0	Medium-quality	89.03	0	Myoviridae	prokaryote
v1f62	55188	101	23	1	High-quality	100	0	unclassified	unclassified
v1f63	49649	75	23	2	High-quality	100	0	Myoviridae	prokaryote

v1f64	9699	19	1	0	High-quality	100	0	unclassified	unclassified
v1f65	44180	77	18	0	Complete	100	0	unclassified	unclassified
v1f66	16593	22	8	0	Medium-quality	87.03	0	unclassified	unclassified
v1f67	14549	18	7	0	Medium-quality	76.28	0	unclassified	unclassified
v1f68	97717	170	19	1	Complete	100	0	Podoviridae_crAss-like	prokaryote
v1f69	38589	65	23	0	Complete	100	0	unclassified	unclassified
v1f6a	28191	50	15	0	Medium-quality	79.25	0	unclassified	unclassified
v1f6b	60909	105	16	2	Complete	100	0	unclassified	unclassified
v1f6c	43603	71	22	0	High-quality	97.76	0	Myoviridae	prokaryote
v1f6d	52486	69	12	1	Complete	100	0	Siphoviridae	prokaryote
v1f6e	55958	72	12	2	High-quality	100	0	unclassified	unclassified
v1f6f	22975	33	8	1	Medium-quality	64.25	0	unclassified	unclassified
v1f70	44529	75	22	1	Complete	100	0	unclassified	unclassified
v1f71	48724	72	6	4	Medium-quality	76.67	0	unclassified	unclassified
v1f72	5144	6	1	0	High-quality	100	0	unclassified	unclassified
v1f73	30380	45	13	0	High-quality	90.1	0	unclassified	unclassified
v1f74	46228	77	19	1	Complete	100	0	unclassified	unclassified
v1f75	42279	62	46	0	High-quality	100	0	Podoviridae	prokaryote
v1f76	63545	95	21	2	High-quality	100	0	Myoviridae	prokaryote
v1f77	6232	7	2	0	High-quality	100	0	unclassified	unclassified
v1f78	25658	35	11	0	Medium-quality	57.05	0	Siphoviridae	prokaryote
v1f79	28398	37	22	0	Medium-quality	62.63	0	Myoviridae	prokaryote
v1f7a	41375	78	18	0	High-quality	94.92	0	unclassified	unclassified
v1f7b	38665	54	28	0	Complete	100	0	Siphoviridae	prokaryote
v1f7c	41868	77	18	1	High-quality	100	0	Siphoviridae	prokaryote
v1f7d	61368	47	8	0	Medium-quality	61.31	0	Podoviridae_crAss-like	prokaryote
v1f7e	19278	27	8	0	Medium-quality	55.68	0	unclassified	unclassified
v1f7f	102938	116	22	1	Complete	100	0	Podoviridae_crAss-like	prokaryote
v1f80	86770	130	12	1	High-quality	100	0	unclassified	unclassified
v1f81	74557	121	16	3	Complete	100	0	unclassified	unclassified
v1f82	181725	299	32	8	Complete	100	0	unclassified	unclassified
v1f83	26789	32	24	0	Medium-quality	56.95	0	Siphoviridae	prokaryote
v1f84	33738	45	12	0	Complete	100	0	unclassified	unclassified
v1f85	40419	52	19	0	Medium-quality	89.92	0	Siphoviridae	prokaryote
v1f86	42825	71	19	0	High-quality	100	0	unclassified	unclassified
v1f87	54199	68	12	3	High-quality	100	0	unclassified	unclassified
v1f88	31272	37	21	0	Medium-quality	88.13	0	Siphoviridae	prokaryote
v1f89	61219	77	18	0	High-quality	100	0	unclassified	unclassified
v1f8a	33646	59	12	1	Complete	100	0	unclassified	unclassified
v1f8b	156969	199	14	2	Complete	100	0	unclassified	unclassified

v1f8c	44702	73	16	1	Complete	100	0	unclassified	unclassified
v1f8d	13569	19	5	0	Complete	100	0	unclassified	unclassified
v1f8e	44388	78	17	1	Medium-quality	68.77	0	unclassified	unclassified
v1f8f	39282	66	15	2	Complete	100	0	unclassified	unclassified
v1f90	79230	135	20	3	High-quality	100	0	unclassified	unclassified
v1f91	59775	69	17	4	High-quality	100	0	unclassified	unclassified
v1f92	85652	129	17	5	Complete	100	0	unclassified	unclassified
v1f93	26177	39	9	0	Medium-quality	58.06	0	Siphoviridae	prokaryote
v1f94	32136	49	31	0	Medium-quality	71.41	0	Siphoviridae	prokaryote
v1f95	44566	73	19	0	Complete	100	0	unclassified	unclassified
v1f96	79334	138	18	5	Complete	100	0	unclassified	unclassified
v1f97	45363	64	22	1	Complete	100	0	Siphoviridae	prokaryote
v1f98	43999	76	18	0	Complete	100	0	unclassified	unclassified
v1f99	44021	72	43	1	High-quality	100	0	Myoviridae	prokaryote
v1f9a	43868	79	17	1	Complete	100	0	unclassified	unclassified
v1f9b	47787	63	12	1	Complete	100	0	unclassified	unclassified
v1f9c	42114	62	7	0	Complete	100	0	unclassified	unclassified
v1f9d	37650	49	9	0	Medium-quality	52.72	0	unclassified	unclassified
v1f9e	76776	89	13	8	High-quality	100	0	unclassified	unclassified
v1f9f	7622	14	1	0	Complete	100	0	Inoviridae	prokaryote
v1fa0	39887	53	9	1	Medium-quality	62.43	0	unclassified	unclassified
v1fa1	36108	63	25	1	Complete	100	0	Siphoviridae	prokaryote
v1fa2	40862	71	31	2	High-quality	97.48	0	Siphoviridae	prokaryote
v1fa3	41864	69	17	1	Medium-quality	87.09	0	Myoviridae	prokaryote
v1fa4	23495	34	8	0	Medium-quality	57.68	0	unclassified	unclassified
v1fa5	34244	47	12	0	Complete	100	0	unclassified	unclassified
v1fa6	41372	66	45	0	Complete	100	0	Myoviridae	prokaryote
v1fa7	15549	19	7	0	Medium-quality	53.65	0	unclassified	unclassified
v1fa8	47430	54	7	5	Medium-quality	56.92	0	unclassified	unclassified
v1fa9	41250	66	21	0	High-quality	100	0	unclassified	unclassified
v1faa	59943	82	14	0	Complete	100	0	unclassified	unclassified
v1fab	56762	91	17	2	High-quality	98.46	0	unclassified	unclassified
v1fac	40694	52	26	1	Medium-quality	67.19	0	Siphoviridae	prokaryote
v1fad	68135	97	24	2	High-quality	100	0	unclassified	unclassified
v1fae	8487	10	1	0	High-quality	100	0	unclassified	unclassified
v1faf	118718	115	16	2	Medium-quality	50.78	0	unclassified	unclassified
v1fb0	45435	52	3	2	High-quality	100	0	unclassified	unclassified
v1fb1	40843	52	17	1	High-quality	100	0	Siphoviridae	prokaryote
v1fb2	90781	107	33	4	High-quality	100	0	Siphoviridae	prokaryote
v1fb3	30384	40	24	1	Medium-quality	87.98	0	Siphoviridae	prokaryote

v1fb4	19920	31	5	0	Medium-quality	50.46	0	Myoviridae	prokaryote
v1fb5	47336	61	12	0	Complete	100	0	Siphoviridae	prokaryote
v1fb6	5795	9	5	0	Complete	100	0	Microviridae	prokaryote
v1fb7	76404	134	16	3	High-quality	96.82	0	unclassified	unclassified
v1fb8	38619	29	7	3	Medium-quality	51.7	0	unclassified	unclassified
v1fb9	66176	62	13	10	High-quality	100	0	unclassified	unclassified
v1fba	35230	65	19	0	Complete	100	0	Siphoviridae	prokaryote
v1fbb	38261	46	10	2	Medium-quality	70.58	0	unclassified	unclassified
v1fbc	28419	43	12	0	Medium-quality	65.52	0	unclassified	unclassified
v1fbd	29258	39	15	1	Medium-quality	69.4	0	unclassified	unclassified
v1fbe	71668	84	5	4	High-quality	100	0	unclassified	unclassified
v1fbf	39228	53	28	1	High-quality	90.04	0	Siphoviridae	prokaryote
v1fc0	80722	123	16	1	Medium-quality	85.24	0	Quimbyviridae	prokaryote
v1fc1	39078	62	17	1	Complete	100	0	unclassified	unclassified
v1fc2	35752	60	20	0	High-quality	100	0	Siphoviridae	prokaryote
v1fc3	32271	47	11	0	High-quality	94.57	0	unclassified	unclassified
v1fc4	40780	57	21	0	High-quality	98.73	0	unclassified	unclassified
v1fc5	37370	46	8	0	High-quality	95.36	0	unclassified	unclassified
v1fc6	44543	62	17	0	High-quality	100	0	Siphoviridae	prokaryote
v1fc7	16619	22	19	0	Medium-quality	50.22	0	Siphoviridae	prokaryote
v1fc8	9661	10	1	0	High-quality	100	0	unclassified	unclassified
v1fc9	36364	59	19	2	High-quality	95.37	0	unclassified	unclassified
v1fca	24478	49	9	0	Medium-quality	57.82	0	unclassified	unclassified
v1fcb	51255	84	22	2	Complete	100	0	unclassified	unclassified
v1fcc	42022	55	15	1	High-quality	100	0	Myoviridae	prokaryote
v1fcd	47034	62	6	5	Medium-quality	79.25	0	unclassified	unclassified
v1fce	33783	51	7	0	Complete	100	0	unclassified	unclassified
v1fcf	38460	52	22	2	High-quality	100	0	Siphoviridae	prokaryote
v1fd0	46727	56	11	4	High-quality	100	0	unclassified	unclassified
v1fd1	40944	63	18	0	High-quality	100	0	Siphoviridae	prokaryote
v1fd2	11700	15	5	0	Complete	100	0	Podoviridae	prokaryote
v1fd3	51953	79	21	0	High-quality	95.87	0	Myoviridae	prokaryote
v1fd4	41031	69	22	0	Complete	100	0	Siphoviridae	prokaryote
v1fd5	41874	55	14	1	High-quality	100	0	unclassified	unclassified
v1fd6	45387	65	10	1	High-quality	100	0	unclassified	unclassified
v1fd7	20305	22	9	0	Medium-quality	51.1	0	unclassified	unclassified
v1fd8	157785	194	18	3	High-quality	100	0	unclassified	unclassified
v1fd9	122119	147	34	3	Complete	100	0	unclassified	unclassified
v1fda	39491	39	12	2	Medium-quality	81.13	0	unclassified	unclassified
v1fdb	147906	173	35	5	Complete	100	0	unclassified	unclassified

v1fdc	39492	50	23	0	High-quality	90.19	0	Siphoviridae	prokaryote
v1fdd	127055	199	57	9	High-quality	100	0	Siphoviridae	prokaryote
v1fde	42059	72	19	0	Complete	100	0	unclassified	unclassified
v1fdf	29794	37	14	0	Medium-quality	78.73	0	Siphoviridae	prokaryote
v1fe0	16051	17	3	2	Medium-quality	55.95	0	unclassified	unclassified
v1fe1	40842	40	23	1	High-quality	100	0	Siphoviridae	prokaryote
v1fe2	31372	43	15	2	Medium-quality	75.91	0	Siphoviridae	prokaryote
v1fe3	52155	71	15	2	Complete	100	0	Siphoviridae	prokaryote
v1fe4	26516	47	12	0	Medium-quality	68.96	0	unclassified	unclassified
v1fe5	44241	77	23	5	Complete	100	0	unclassified	unclassified
v1fe6	133789	186	50	2	Complete	100	0	unclassified	unclassified
v1fe7	52690	71	12	1	Complete	100	0	Siphoviridae	prokaryote
v1fe8	40508	53	29	0	Complete	100	0	Myoviridae	prokaryote
v1fe9	46328	71	15	1	Complete	100	0	unclassified	unclassified
v1fea	78045	126	20	6	High-quality	100	0	Quimbyviridae	prokaryote
v1feb	52867	81	27	1	Complete	100	0	Myoviridae	prokaryote
v1fec	42736	62	17	0	Complete	100	0	Siphoviridae	prokaryote
v1fed	99372	148	27	1	Medium-quality	67.27	0	unclassified	unclassified
v1fee	16774	24	8	0	Medium-quality	77.47	0	unclassified	unclassified
v1fef	41259	45	2	0	High-quality	94.81	0	unclassified	unclassified
v1ff0	84928	126	19	1	High-quality	100	0	unclassified	unclassified
v1ff1	33290	51	19	1	High-quality	91.61	0	Siphoviridae	prokaryote
v1ff2	10438	11	3	1	High-quality	100	0	unclassified	unclassified
v1ff3	5906	8	3	0	Complete	100	0	unclassified	unclassified
v1ff4	29622	44	18	1	Medium-quality	73.31	0	Siphoviridae	prokaryote
v1ff5	40232	69	24	0	High-quality	100	0	Siphoviridae	prokaryote
v1ff6	23293	28	24	0	Medium-quality	57.27	0	Podoviridae	prokaryote
v1ff7	43737	76	15	2	High-quality	98.61	0	unclassified	unclassified
v1ff8	39729	63	10	0	Complete	100	0	unclassified	unclassified
v1ff9	16746	19	8	0	Medium-quality	88.11	0	Salasmaviridae	prokaryote
v1ffa	24724	48	8	0	Medium-quality	55.54	0	unclassified	unclassified
v1ffb	52453	76	23	1	High-quality	100	0	unclassified	unclassified
v1ffc	29878	46	23	0	Medium-quality	62.4	0	Siphoviridae	prokaryote
v1ffd	11401	12	7	0	Medium-quality	59.09	0	Salasmaviridae	prokaryote
v1ffe	58253	100	20	2	Complete	100	0	unclassified	unclassified
v1fff	43640	71	19	0	Complete	100	0	unclassified	unclassified
v2000	90687	143	20	0	Medium-quality	55.84	0	unclassified	unclassified
v2001	55259	93	23	0	Complete	100	0	unclassified	unclassified
v2002	34151	48	11	0	Complete	100	0	unclassified	unclassified
v2003	46281	79	12	0	Complete	100	0	unclassified	unclassified

v2004	77203	95	15	1	Complete	100	0	unclassified	unclassified
v2005	15207	26	1	0	Complete	100	0	unclassified	unclassified
v2006	60286	95	5	3	Medium-quality	55.37	0	unclassified	unclassified
v2007	49148	75	5	0	Medium-quality	51.48	0	Podoviridae_crAss-like	prokaryote
v2008	45402	77	15	0	Medium-quality	77.01	0	unclassified	unclassified
v2009	24938	38	24	0	Medium-quality	54.75	0	Siphoviridae	prokaryote
v200a	36373	64	17	0	Complete	100	0	unclassified	unclassified
v200b	65468	94	15	3	High-quality	100	0	Myoviridae	prokaryote
v200c	27218	34	13	0	Medium-quality	58.14	0	unclassified	unclassified
v200d	29926	38	20	0	Medium-quality	76.43	0	Siphoviridae	prokaryote
v200e	88445	116	14	3	Complete	100	0	Quimbyviridae	prokaryote
v200f	35904	40	5	2	Medium-quality	59.76	0	Quimbyviridae	prokaryote
v2010	30342	40	23	0	Medium-quality	58.62	0	Siphoviridae	prokaryote
v2011	22012	29	1	0	Medium-quality	51.24	0	unclassified	unclassified
v2012	42620	70	17	0	Complete	100	0	unclassified	unclassified
v2013	66502	94	23	0	Medium-quality	75.75	0	unclassified	unclassified
v2014	54113	72	14	4	Medium-quality	72.19	0	unclassified	unclassified
v2015	90943	127	41	2	High-quality	100	0	Myoviridae	prokaryote
v2016	12404	7	1	0	High-quality	100	0	unclassified	unclassified
v2017	77439	130	23	5	Complete	100	0	unclassified	unclassified
v2018	24626	38	13	0	Medium-quality	61.67	0	unclassified	unclassified
v2019	55453	60	7	5	High-quality	100	0	unclassified	unclassified
v201a	30207	32	7	3	Medium-quality	65.49	0	Siphoviridae	prokaryote
v201b	36943	54	23	0	High-quality	100	0	Siphoviridae	prokaryote
v201c	35149	54	19	1	High-quality	100	0	Siphoviridae	prokaryote
v201d	50272	87	23	0	Complete	100	0	unclassified	unclassified
v201e	68440	90	13	2	Medium-quality	77.34	0	Quimbyviridae	prokaryote
v201f	160423	239	42	5	High-quality	100	0	unclassified	unclassified
v2020	32628	47	11	0	Medium-quality	53.97	0	unclassified	unclassified
v2021	29160	28	8	1	Medium-quality	60.81	0	Siphoviridae	prokaryote
v2022	41745	63	16	2	High-quality	100	0	Siphoviridae	prokaryote
v2023	36767	44	9	0	Medium-quality	53.65	0	Quimbyviridae	prokaryote
v2024	56022	58	7	2	Medium-quality	77.18	0	unclassified	unclassified
v2025	22579	29	17	0	Medium-quality	61.03	0	Siphoviridae	prokaryote
v2026	25010	39	6	1	High-quality	100	0	unclassified	unclassified
v2027	42075	62	25	0	Medium-quality	67.76	0	Myoviridae	prokaryote
v2028	110194	172	35	2	High-quality	100	0	unclassified	unclassified
v2029	30405	50	19	0	Medium-quality	74.62	0	unclassified	unclassified
v202a	90150	141	35	3	High-quality	100	0	unclassified	unclassified
v202b	38883	53	26	0	High-quality	96.83	0	Siphoviridae	prokaryote

v202c	27203	28	15	0	Medium-quality	52.82	0	Siphoviridae	prokaryote
v202d	78361	128	25	3	Complete	100	0	unclassified	unclassified
v202e	29843	41	17	0	Medium-quality	74.46	0	Siphoviridae	prokaryote
v202f	12975	16	2	0	Medium-quality	55.71	0	unclassified	unclassified
v2030	44282	64	32	0	Medium-quality	73.51	0	Siphoviridae	prokaryote
v2031	88752	142	28	4	Complete	100	0	unclassified	unclassified
v2032	115017	211	30	3	Medium-quality	63.14	0	unclassified	unclassified
v2033	19347	28	2	0	High-quality	100	0	unclassified	unclassified
v2034	40886	55	3	2	High-quality	94.37	0	unclassified	unclassified
v2035	31658	36	26	1	Medium-quality	61.95	0	Myoviridae	prokaryote
v2036	105096	135	13	1	Complete	100	0	unclassified	unclassified
v2037	57969	83	24	2	Medium-quality	86.99	0	unclassified	unclassified
v2038	29240	46	16	1	Medium-quality	84.92	0	Siphoviridae	prokaryote
v2039	48787	62	24	0	High-quality	100	0	Siphoviridae	prokaryote
v203a	46234	72	16	0	Complete	100	0	unclassified	unclassified
v203b	67900	83	14	2	High-quality	100	0	unclassified	unclassified
v203c	32528	48	17	1	High-quality	100	0	Siphoviridae	prokaryote
v203d	33514	52	21	0	Medium-quality	85.86	0	Siphoviridae	prokaryote
v203e	173771	247	23	7	High-quality	99.23	0	unclassified	unclassified
v203f	35310	66	13	0	Medium-quality	84.25	0	unclassified	unclassified
v2040	38227	38	2	1	Medium-quality	87.22	0	unclassified	unclassified
v2041	38604	65	20	3	Complete	100	0	unclassified	unclassified
v2042	103115	99	16	0	Complete	100	0	Podoviridae_crAss-like	prokaryote
v2043	28914	49	7	0	Medium-quality	71.32	0	unclassified	unclassified
v2044	74998	97	13	2	Complete	100	0	unclassified	unclassified
v2045	90107	117	30	1	Medium-quality	54.02	0	unclassified	unclassified
v2046	40233	54	49	0	Complete	100	0	Autographiviridae	prokaryote
v2047	35428	42	9	0	Medium-quality	71.38	0	Siphoviridae	prokaryote
v2048	37579	58	17	0	Complete	100	0	unclassified	unclassified
v2049	18107	27	7	0	High-quality	100	0	unclassified	unclassified
v204a	103916	144	32	2	Medium-quality	60.05	0	unclassified	unclassified
v204b	63677	65	7	1	High-quality	100	0	unclassified	unclassified
v204c	44560	70	24	0	Complete	100	0	Siphoviridae	prokaryote
v204d	102845	117	19	2	Complete	100	0	Gratiaviridae	prokaryote
v204e	37581	56	17	0	High-quality	100	0	Siphoviridae	prokaryote
v204f	10416	18	5	0	Medium-quality	85.19	0	Siphoviridae	prokaryote
v2050	34962	52	30	0	High-quality	99.23	0	Myoviridae	prokaryote
v2051	25928	42	31	0	Medium-quality	65.25	0	Siphoviridae	prokaryote
v2052	44206	71	22	0	High-quality	99.21	0	unclassified	unclassified
v2053	35142	45	8	0	Medium-quality	75.84	0	Siphoviridae	prokaryote

v2054	110434	169	26	3	Medium-quality	70.69	0	unclassified	unclassified
v2055	13522	14	3	1	High-quality	100	0	unclassified	unclassified
v2056	6554	7	3	0	High-quality	100	0	unclassified	unclassified
v2057	27140	44	12	0	Medium-quality	75.89	0	Siphoviridae	prokaryote
v2058	54754	73	6	2	Medium-quality	64.6	0	Quimbyviridae	prokaryote
v2059	12833	17	5	0	High-quality	94.09	0	unclassified	unclassified
v205a	57478	88	26	1	Complete	100	0	unclassified	unclassified
v205b	46379	79	20	1	High-quality	100	0	unclassified	unclassified
v205c	31051	42	31	0	Medium-quality	81.24	0	Siphoviridae	prokaryote
v205d	67652	97	14	2	High-quality	93.25	0	unclassified	unclassified
v205e	50134	82	24	2	Medium-quality	62.18	0	unclassified	unclassified
v205f	23839	19	5	0	Medium-quality	53.96	0	unclassified	unclassified
v2060	23989	38	10	1	Medium-quality	57.43	0	Siphoviridae	prokaryote
v2061	176057	312	46	14	High-quality	100	0	unclassified	unclassified
v2062	144490	160	43	2	High-quality	100	0	unclassified	unclassified
v2063	42026	59	15	1	Complete	100	0	unclassified	unclassified
v2064	35624	52	26	0	Medium-quality	79.48	0	Myoviridae	prokaryote
v2065	55399	67	8	0	Medium-quality	59.65	0	unclassified	unclassified
v2066	41977	49	15	2	Complete	100	0	Siphoviridae	prokaryote
v2067	28521	44	8	0	Medium-quality	66.17	0	unclassified	unclassified
v2068	23192	34	10	0	Medium-quality	62.53	0	Siphoviridae	prokaryote
v2069	29329	39	26	0	Medium-quality	77.09	0	Siphoviridae	prokaryote
v206a	40903	72	23	5	Complete	100	0	Myoviridae	prokaryote
v206b	58478	81	14	2	Complete	100	0	unclassified	unclassified
v206c	67348	59	8	4	Medium-quality	76.85	0	unclassified	unclassified
v206d	60741	80	9	1	Complete	100	0	unclassified	unclassified
v206e	60148	66	7	6	High-quality	100	0	unclassified	unclassified
v206f	41035	51	8	7	Medium-quality	70.69	0	Myoviridae	prokaryote
v2070	78529	89	16	11	High-quality	100	0	unclassified	unclassified
v2071	37630	59	31	0	High-quality	100	0	Myoviridae	prokaryote
v2072	40076	58	24	0	High-quality	91.7	0	Myoviridae	prokaryote
v2073	69426	118	14	3	Medium-quality	88.01	0	unclassified	unclassified
v2074	49816	85	16	5	High-quality	90.91	0	Siphoviridae	prokaryote
v2075	59855	91	33	2	High-quality	99.14	0	Siphoviridae	prokaryote
v2076	69490	115	15	5	Complete	100	0	unclassified	unclassified
v2077	66054	79	12	3	Complete	100	0	unclassified	unclassified
v2078	37843	53	15	0	High-quality	96.85	0	unclassified	unclassified
v2079	109928	188	16	6	Medium-quality	65.54	0	unclassified	unclassified
v207a	53622	56	9	2	High-quality	100	0	unclassified	unclassified
v207b	72290	94	24	3	High-quality	100	0	unclassified	unclassified

v207c	23765	26	19	0	Medium-quality	51.67	0	Siphoviridae	prokaryote
v207d	107782	152	18	3	Medium-quality	52.46	0	unclassified	unclassified
v207e	25260	42	12	1	Medium-quality	65.91	0	Siphoviridae	prokaryote
v207f	32498	47	31	1	Medium-quality	81.83	0	Siphoviridae	prokaryote
v2080	41602	63	22	3	Complete	100	0	Siphoviridae	prokaryote
v2081	7909	8	1	0	High-quality	100	0	unclassified	unclassified
v2082	31235	52	17	1	Medium-quality	80.07	0	Siphoviridae	prokaryote
v2083	46092	75	19	0	Complete	100	0	unclassified	unclassified
v2084	42268	57	26	0	High-quality	96.99	0	Myoviridae	prokaryote
v2085	29103	56	18	0	Medium-quality	72.41	0	Siphoviridae	prokaryote
v2086	18434	22	4	0	High-quality	100	0	unclassified	unclassified
v2087	78185	124	24	3	Complete	100	0	unclassified	unclassified
v2088	144725	167	33	4	High-quality	90.1	0	unclassified	unclassified
v2089	39431	61	6	1	Complete	100	0	unclassified	unclassified
v208a	41171	55	11	1	Medium-quality	83.16	0	unclassified	unclassified
v208b	74256	78	14	7	Medium-quality	84.18	0	unclassified	unclassified
v208c	36976	55	16	1	High-quality	100	0	unclassified	unclassified
v208d	30926	41	34	0	High-quality	98.69	0	Myoviridae	prokaryote
v208e	54627	93	11	3	Medium-quality	69.2	0	unclassified	unclassified
v208f	55349	71	35	4	High-quality	100	0	Siphoviridae	prokaryote
v2090	14552	19	2	0	Medium-quality	63.65	0	unclassified	unclassified
v2091	100784	173	15	1	Complete	100	0	Podoviridae_crAss-like	prokaryote
v2092	85438	132	14	2	Complete	100	0	Quimbyviridae	prokaryote
v2093	129812	165	35	4	Medium-quality	89.52	0	unclassified	unclassified
v2094	36880	50	28	0	High-quality	90.62	0	Siphoviridae	prokaryote
v2095	39896	62	25	1	High-quality	98.71	0	Siphoviridae	prokaryote
v2096	72878	128	11	3	Medium-quality	58.02	0	unclassified	unclassified
v2097	28994	50	11	0	Medium-quality	76.95	0	unclassified	unclassified
v2098	55778	83	29	1	Complete	100	0	Myoviridae	prokaryote
v2099	20717	23	10	0	Medium-quality	53.98	0	unclassified	unclassified
v209a	55878	85	30	1	High-quality	100	0	Siphoviridae	prokaryote
v209b	79716	119	28	2	Complete	100	0	unclassified	unclassified
v209c	47882	63	13	5	High-quality	100	0	unclassified	unclassified
v209d	40746	83	18	0	High-quality	100	0	Siphoviridae	prokaryote
v209e	152907	270	35	7	Medium-quality	89	0	unclassified	unclassified
v209f	41306	67	14	1	High-quality	99.7	0	unclassified	unclassified
v20a0	56850	104	24	4	Complete	100	0	Siphoviridae	prokaryote
v20a1	87078	111	13	2	Complete	100	0	Flandersviridae	prokaryote
v20a2	35789	54	13	0	High-quality	100	0	unclassified	unclassified
v20a3	26269	31	13	0	Medium-quality	64.54	0	unclassified	unclassified

v20a4	33729	44	25	1	High-quality	94.64	0	Siphoviridae	prokaryote
v20a5	39125	61	12	1	Complete	100	0	unclassified	unclassified
v20a6	36877	46	15	1	Medium-quality	55.29	0	Myoviridae	prokaryote
v20a7	42488	56	20	2	High-quality	100	0	unclassified	unclassified
v20a8	70669	121	16	3	Medium-quality	87.87	0	unclassified	unclassified
v20a9	45372	51	8	5	Medium-quality	76.78	0	unclassified	unclassified
v20aa	35193	51	15	3	High-quality	100	0	unclassified	unclassified
v20ab	103048	107	16	0	Medium-quality	89.62	0	Podoviridae_crAss-like	prokaryote
v20ac	72437	116	14	1	Medium-quality	84.69	0	unclassified	unclassified
v20ad	8101	12	1	0	High-quality	91.56	0	unclassified	unclassified
v20ae	43528	65	31	0	High-quality	100	0	Myoviridae	prokaryote
v20af	32704	55	18	1	Medium-quality	85.98	0	Siphoviridae	prokaryote
v20b0	75589	102	28	2	Complete	100	0	unclassified	unclassified
v20b1	38327	54	17	1	High-quality	93.94	0	Siphoviridae	prokaryote
v20b2	41266	55	23	2	High-quality	100	0	Siphoviridae	prokaryote
v20b3	34932	60	28	1	Medium-quality	86.69	0	unclassified	unclassified
v20b4	26858	43	11	1	Medium-quality	61.47	0	unclassified	unclassified
v20b5	36745	58	15	3	High-quality	96.2	0	unclassified	unclassified
v20b6	130438	204	25	6	Medium-quality	63.32	0	unclassified	unclassified
v20b7	40076	51	11	0	Medium-quality	64.43	0	unclassified	unclassified
v20b8	53384	65	14	3	Complete	100	0	Siphoviridae	prokaryote
v20b9	29556	47	14	1	Medium-quality	78.18	0	Siphoviridae	prokaryote
v20ba	23959	36	13	3	Medium-quality	70.67	0	Siphoviridae	prokaryote
v20bb	53827	70	13	0	Medium-quality	74.07	0	unclassified	unclassified
v20bc	35242	45	9	1	Medium-quality	88.05	0	unclassified	unclassified
v20bd	65123	50	8	2	High-quality	100	0	unclassified	unclassified
v20be	84717	116	13	5	High-quality	96.17	0	Quimbyviridae	prokaryote
v20bf	36224	54	20	0	Medium-quality	80.71	0	Myoviridae	prokaryote
v20c0	39767	56	16	3	High-quality	100	0	unclassified	unclassified
v20c1	34861	49	7	1	Medium-quality	89.26	0	unclassified	unclassified
v20c2	46368	60	18	4	High-quality	100	0	Siphoviridae	prokaryote
v20c3	26037	33	17	1	Medium-quality	61.71	0	Siphoviridae	prokaryote
v20c4	26654	43	14	0	Medium-quality	89.97	0	unclassified	unclassified
v20c5	165669	212	39	7	High-quality	100	0	unclassified	unclassified
v20c6	44901	74	17	0	Complete	100	0	unclassified	unclassified
v20c7	45646	77	22	0	Complete	100	0	unclassified	unclassified
v20c8	39636	70	16	1	Complete	100	0	Siphoviridae	prokaryote
v20c9	42429	63	7	3	Medium-quality	87.66	0	unclassified	unclassified
v20ca	50487	68	14	1	Medium-quality	71.06	0	unclassified	unclassified
v20cb	41019	68	15	0	Complete	100	0	unclassified	unclassified

v20cc	98041	98	10	0	Complete	100	0	Podoviridae_crAss-like	prokaryote
v20cd	35017	58	11	0	Medium-quality	70.41	0	unclassified	unclassified
v20ce	144627	231	37	2	High-quality	99.81	0	unclassified	unclassified
v20cf	21478	26	6	0	Medium-quality	52.45	0	Siphoviridae	prokaryote
v20d0	39821	38	5	0	Complete	100	0	unclassified	unclassified
v20d1	19786	27	20	0	Medium-quality	60.86	0	Siphoviridae	prokaryote
v20d2	84960	131	20	3	High-quality	100	0	Quimbyviridae	prokaryote
v20d3	41427	66	26	3	High-quality	100	0	Myoviridae	prokaryote
v20d4	40335	65	12	1	Complete	100	0	unclassified	unclassified
v20d5	36502	57	13	2	High-quality	90.46	0	unclassified	unclassified
v20d6	24824	32	13	0	Medium-quality	66.48	0	unclassified	unclassified
v20d7	37668	56	22	0	Complete	100	0	Siphoviridae	prokaryote
v20d8	53757	93	20	0	High-quality	100	0	unclassified	unclassified
v20d9	22129	35	2	1	High-quality	100	0	unclassified	unclassified
v20da	23749	43	11	0	Medium-quality	56.13	0	Siphoviridae	prokaryote
v20db	31972	40	5	1	Medium-quality	76.97	0	unclassified	unclassified
v20dc	47662	64	19	1	High-quality	95.85	0	Siphoviridae	prokaryote
v20dd	35992	61	17	1	High-quality	95.82	0	unclassified	unclassified
v20de	72917	117	20	3	Complete	100	0	unclassified	unclassified
v20df	37379	65	14	0	Complete	100	0	unclassified	unclassified
v20e0	61236	85	27	3	Complete	100	0	Siphoviridae	prokaryote
v20e1	25841	38	12	0	Medium-quality	57.51	0	unclassified	unclassified
v20e2	41691	55	25	4	Medium-quality	77.96	0	Siphoviridae	prokaryote
v20e3	42344	59	5	1	Complete	100	0	unclassified	unclassified
v20e4	38847	55	16	4	High-quality	97.33	0	Siphoviridae	prokaryote
v20e5	43960	70	17	0	High-quality	100	0	unclassified	unclassified
v20e6	62821	92	18	3	Medium-quality	69.18	0	unclassified	unclassified
v20e7	41067	67	17	1	High-quality	97.99	0	Myoviridae	prokaryote
v20e8	49755	49	4	1	Medium-quality	67.12	0	unclassified	unclassified
v20e9	41751	59	7	0	Complete	100	0	unclassified	unclassified
v20ea	27922	28	6	0	Medium-quality	66.66	0	unclassified	unclassified
v20eb	30111	39	6	0	Medium-quality	72.2	0	unclassified	unclassified
v20ec	24779	42	17	0	Medium-quality	70.88	0	Siphoviridae	prokaryote
v20ed	22947	42	18	0	Medium-quality	56.6	0	Siphoviridae	prokaryote
v20ee	90540	117	28	2	Medium-quality	66.31	0	Siphoviridae	prokaryote
v20ef	16710	20	3	2	Medium-quality	58.24	0	Siphoviridae	prokaryote
v20f0	28812	27	6	2	Medium-quality	57.86	0	unclassified	unclassified
v20f1	38705	52	15	2	High-quality	99.85	0	unclassified	unclassified
v20f2	41054	50	47	0	Complete	100	0	Autographiviridae	prokaryote
v20f3	38460	57	44	1	High-quality	95.1	0	Podoviridae	prokaryote

v20f4	22224	32	16	0	Medium-quality	55.04	0	Siphoviridae	prokaryote
v20f5	39176	59	20	1	High-quality	98.43	0	Siphoviridae	prokaryote
v20f6	43617	62	8	0	High-quality	100	0	unclassified	unclassified
v20f7	12461	20	3	1	High-quality	98.15	0	unclassified	unclassified
v20f8	27942	46	10	1	Medium-quality	62.78	0	unclassified	unclassified
v20f9	60539	91	32	0	Complete	100	0	Siphoviridae	prokaryote
v20fa	47828	73	14	2	High-quality	100	0	unclassified	unclassified
v20fb	37835	56	48	0	High-quality	95.14	0	Siphoviridae	prokaryote
v20fc	30154	47	22	0	Medium-quality	82.53	0	Siphoviridae	prokaryote
v20fd	38222	51	18	0	Medium-quality	76.87	0	unclassified	unclassified
v20fe	36490	58	19	1	Medium-quality	89.34	0	unclassified	unclassified
v20ff	44056	72	15	2	High-quality	100	0	unclassified	unclassified
v2100	19730	30	3	1	High-quality	100	0	unclassified	unclassified
v2101	39081	63	21	0	Medium-quality	86.79	0	unclassified	unclassified
v2102	9017	10	1	0	High-quality	100	0	unclassified	unclassified
v2103	47709	69	34	1	Complete	100	0	Myoviridae	prokaryote
v2104	32228	54	21	1	Medium-quality	75.82	0	Myoviridae	prokaryote
v2105	44506	58	13	3	Medium-quality	58.07	0	unclassified	unclassified
v2106	89969	118	18	4	Medium-quality	51.75	0	unclassified	unclassified
v2107	41246	50	11	3	Complete	100	0	unclassified	unclassified
v2108	101328	177	22	2	Complete	100	0	Podoviridae_crAss-like	prokaryote
v2109	17684	31	15	1	Medium-quality	50.12	0	Siphoviridae	prokaryote
v210a	29983	43	12	1	Medium-quality	78.05	0	unclassified	unclassified
v210b	67620	120	13	3	Medium-quality	85.65	0	unclassified	unclassified
v210c	9470	10	1	0	High-quality	100	0	unclassified	unclassified
v210d	45865	64	28	0	Medium-quality	73.48	0	Siphoviridae	prokaryote
v210e	47370	68	64	0	High-quality	95.29	0	Drexlerviridae	prokaryote
v210f	44139	73	19	0	Complete	100	0	unclassified	unclassified
v2110	53787	49	3	0	Medium-quality	72.59	0	unclassified	unclassified
v2111	73422	92	11	5	Medium-quality	83.67	0	Quimbyviridae	prokaryote
v2112	26455	27	6	0	Medium-quality	64.13	0	unclassified	unclassified
v2113	20531	29	21	0	Medium-quality	60.36	0	Siphoviridae	prokaryote
v2114	27219	43	13	0	Medium-quality	71.53	0	unclassified	unclassified
v2115	38978	57	29	0	Medium-quality	55.96	0	Siphoviridae	prokaryote
v2116	12005	15	6	0	High-quality	100	0	unclassified	unclassified
v2117	31771	43	6	3	Medium-quality	71.88	0	unclassified	unclassified
v2118	36685	51	13	0	High-quality	90.23	0	Siphoviridae	prokaryote
v2119	40617	47	7	1	Medium-quality	71.95	0	unclassified	unclassified
v211a	45254	77	18	0	Complete	100	0	unclassified	unclassified
v211b	173674	221	47	5	High-quality	100	0	unclassified	unclassified

v211c	22747	34	28	0	Medium-quality	56.93	0	Siphoviridae	prokaryote
v211d	29422	48	18	0	Medium-quality	84.14	0	unclassified	unclassified
v211e	29033	47	15	3	Medium-quality	69.57	0	Siphoviridae	prokaryote
v211f	91615	141	17	2	Complete	100	0	unclassified	unclassified
v2120	16020	19	5	0	Medium-quality	53.33	0	unclassified	unclassified
v2121	46604	65	9	1	Complete	100	0	Siphoviridae	prokaryote
v2122	43605	56	22	0	Complete	100	0	Autographiviridae	prokaryote
v2123	38051	64	17	0	High-quality	100	0	unclassified	unclassified
v2124	38737	58	14	2	Medium-quality	81.84	0	unclassified	unclassified
v2125	36241	46	15	0	High-quality	90.4	0	unclassified	unclassified
v2126	58192	87	12	1	Medium-quality	64.16	0	unclassified	unclassified
v2127	43813	77	22	1	High-quality	99.81	0	Siphoviridae	prokaryote
v2128	24938	31	11	1	Medium-quality	59.01	0	Siphoviridae	prokaryote
v2129	42010	59	35	0	High-quality	100	0	Siphoviridae	prokaryote
v212a	44584	68	18	0	Complete	100	0	unclassified	unclassified
v212b	33617	47	13	1	Medium-quality	75.01	0	unclassified	unclassified
v212c	119725	231	26	7	Medium-quality	62.62	0	unclassified	unclassified
v212d	79811	117	35	2	High-quality	100	0	Siphoviridae	prokaryote
v212e	39998	77	17	0	Complete	100	0	unclassified	unclassified
v212f	90991	149	31	3	Complete	100	0	unclassified	unclassified
v2130	40308	53	30	1	High-quality	95.68	0	Myoviridae	prokaryote
v2131	36862	47	21	2	Medium-quality	66.68	0	Siphoviridae	prokaryote
v2132	29158	39	17	1	Medium-quality	68.42	0	Siphoviridae	prokaryote
v2133	23772	38	12	0	Medium-quality	58.01	0	Siphoviridae	prokaryote
v2134	6097	10	1	0	High-quality	100	0	Inoviridae	prokaryote
v2135	19431	38	9	1	Medium-quality	67.06	0	unclassified	unclassified
v2136	13144	16	5	0	Medium-quality	67.88	0	Podoviridae	prokaryote
v2137	38299	65	17	0	Complete	100	0	Siphoviridae	prokaryote
v2138	43254	76	18	1	High-quality	100	0	unclassified	unclassified
v2139	73418	80	33	1	Complete	100	0	Schitoviridae	prokaryote
v213a	87678	123	14	2	Complete	100	0	unclassified	unclassified
v213b	40738	70	19	0	High-quality	95.96	0	Siphoviridae	prokaryote
v213c	42624	73	18	0	Complete	100	0	unclassified	unclassified
v213d	34851	56	15	1	Medium-quality	86.78	0	unclassified	unclassified
v213e	40260	65	25	1	Medium-quality	87.06	0	Myoviridae	prokaryote
v213f	167271	267	42	2	High-quality	94.86	0	unclassified	unclassified
v2140	54059	72	27	3	Medium-quality	80.53	0	Myoviridae	prokaryote
v2141	23378	28	14	0	Medium-quality	58.56	0	Siphoviridae	prokaryote
v2142	61917	100	21	0	High-quality	100	0	unclassified	unclassified
v2143	55715	68	28	3	High-quality	100	0	Myoviridae	prokaryote

v2144	81831	126	27	5	Complete	100	0	unclassified	unclassified
v2145	26343	36	13	0	Medium-quality	58.41	0	unclassified	unclassified
v2146	34769	44	5	4	Medium-quality	86.08	0	unclassified	unclassified
v2147	76334	124	25	2	High-quality	94.7	0	unclassified	unclassified
v2148	44079	76	19	2	Complete	100	0	unclassified	unclassified
v2149	43540	77	12	1	Complete	100	0	unclassified	unclassified
v214a	26940	34	18	0	Medium-quality	63.72	0	Siphoviridae	prokaryote
v214b	25957	44	16	0	Medium-quality	63.53	0	unclassified	unclassified
v214c	97893	99	12	0	Complete	100	0	Podoviridae_crAss-like	prokaryote
v214d	79646	126	16	2	High-quality	93.97	0	unclassified	unclassified
v214e	40227	64	21	0	Complete	100	0	Siphoviridae	prokaryote
v214f	8457	7	1	0	High-quality	100	0	unclassified	unclassified
v2150	33167	55	11	2	High-quality	99.91	0	unclassified	unclassified
v2151	34224	49	10	0	Medium-quality	56.97	0	unclassified	unclassified
v2152	45759	74	17	0	Complete	100	0	unclassified	unclassified
v2153	62163	92	34	2	High-quality	100	0	Siphoviridae	prokaryote
v2154	35948	46	21	1	High-quality	100	0	Siphoviridae	prokaryote
v2155	20366	33	25	0	Medium-quality	53.84	0	Siphoviridae	prokaryote
v2156	35815	51	13	0	High-quality	100	0	unclassified	unclassified
v2157	34154	53	22	0	Medium-quality	87.68	0	unclassified	unclassified
v2158	43074	72	21	0	Complete	100	0	Siphoviridae	prokaryote
v2159	80845	128	19	2	High-quality	100	0	unclassified	unclassified
v215a	59799	104	12	2	Medium-quality	72.01	0	unclassified	unclassified
v215b	58192	70	23	3	High-quality	100	0	Siphoviridae	prokaryote
v215c	8657	16	5	0	Medium-quality	80.36	0	Siphoviridae	prokaryote
v215d	29072	41	12	0	Medium-quality	66.41	0	unclassified	unclassified
v215e	66803	80	12	2	Complete	100	0	unclassified	unclassified
v215f	35757	52	16	1	Medium-quality	85.87	0	unclassified	unclassified
v2160	41716	54	36	1	High-quality	100	0	Siphoviridae	prokaryote
v2161	18958	27	2	0	High-quality	99.48	0	unclassified	unclassified
v2162	131766	186	24	3	Medium-quality	76.68	0	unclassified	unclassified
v2163	40322	58	21	3	High-quality	100	0	Siphoviridae	prokaryote
v2164	28896	49	9	2	Medium-quality	66.01	0	Siphoviridae	prokaryote
v2165	49507	64	22	1	High-quality	100	0	Siphoviridae	prokaryote
v2166	55341	95	5	2	Medium-quality	50.84	0	unclassified	unclassified
v2167	42095	51	15	0	Complete	100	0	unclassified	unclassified
v2168	25458	32	11	1	Medium-quality	56.48	0	Siphoviridae	prokaryote
v2169	108282	122	24	10	High-quality	100	0	unclassified	unclassified
v216a	50824	75	19	0	High-quality	100	0	unclassified	unclassified
v216b	25237	31	11	3	Medium-quality	54.1	0	Siphoviridae	prokaryote

v216c	95253	173	15	3	High-quality	97.17	0	Podoviridae_crAss-like	prokaryote
v216d	38316	46	26	1	High-quality	94.95	0	Siphoviridae	prokaryote
v216e	39532	45	7	3	Medium-quality	78.2	0	unclassified	unclassified
v216f	21336	21	15	1	Medium-quality	52.9	0	Siphoviridae	prokaryote
v2170	52956	80	25	1	Complete	100	0	Siphoviridae	prokaryote
v2171	25716	27	23	0	Medium-quality	68.82	0	Siphoviridae	prokaryote
v2172	30645	45	8	0	Medium-quality	68.14	0	unclassified	unclassified
v2173	68382	81	13	2	High-quality	100	0	unclassified	unclassified
v2174	56663	65	10	2	Medium-quality	64.44	0	Quimbyviridae	prokaryote
v2175	42904	56	7	0	Medium-quality	64.67	0	Quimbyviridae	prokaryote
v2176	143330	265	29	5	Medium-quality	74.36	0	unclassified	unclassified
v2177	25288	32	17	1	Medium-quality	62.37	0	Podoviridae	prokaryote
v2178	62577	62	15	11	High-quality	100	0	unclassified	unclassified
v2179	27708	51	16	1	Medium-quality	69.45	0	Siphoviridae	prokaryote
v217a	56312	91	17	1	High-quality	90.38	0	unclassified	unclassified
v217b	32124	60	17	0	Medium-quality	83.18	0	unclassified	unclassified
v217c	139912	205	34	3	Medium-quality	86.47	0	unclassified	unclassified
v217d	21532	26	22	0	Medium-quality	61.04	0	Siphoviridae	prokaryote
v217e	30219	47	18	0	Medium-quality	76.79	0	Siphoviridae	prokaryote
v217f	41354	54	28	1	High-quality	100	0	Siphoviridae	prokaryote
v2180	88595	119	10	5	High-quality	100	0	unclassified	unclassified
v2181	42000	68	18	0	Complete	100	0	unclassified	unclassified
v2182	31745	46	15	0	High-quality	90.23	0	Siphoviridae	prokaryote
v2183	30604	51	28	0	Medium-quality	66.18	0	Myoviridae	prokaryote
v2184	26016	36	14	0	Medium-quality	65.6	0	Siphoviridae	prokaryote
v2185	120193	150	31	1	Medium-quality	74.73	0	unclassified	unclassified
v2186	23159	31	23	0	Medium-quality	62.43	0	Myoviridae	prokaryote
v2187	59543	81	10	1	Medium-quality	67.73	0	Quimbyviridae	prokaryote
v2188	20131	20	20	0	Medium-quality	53.81	0	Siphoviridae	prokaryote
v2189	22985	28	16	1	Medium-quality	57.29	0	Siphoviridae	prokaryote
v218a	25173	24	12	0	Medium-quality	50.63	0	Siphoviridae	prokaryote
v218b	43487	71	16	2	High-quality	97.32	0	unclassified	unclassified
v218c	89944	129	51	0	High-quality	100	0	Siphoviridae	prokaryote
v218d	39637	61	18	1	High-quality	100	0	unclassified	unclassified
v218e	56934	60	22	1	High-quality	100	0	Siphoviridae	prokaryote
v218f	40018	64	19	0	High-quality	95.49	0	Siphoviridae	prokaryote
v2190	57465	101	24	4	Complete	100	0	Siphoviridae	prokaryote
v2191	34667	51	15	0	High-quality	90.65	0	unclassified	unclassified
v2192	56578	72	14	3	High-quality	100	0	unclassified	unclassified
v2193	42084	36	9	5	Medium-quality	89.19	0	Siphoviridae	prokaryote

v2194	29909	34	10	6	Medium-quality	58.55	0	Siphoviridae	prokaryote
v2195	70303	104	19	2	High-quality	99.01	0	unclassified	unclassified
v2196	20138	30	3	0	High-quality	100	0	unclassified	unclassified
v2197	34534	50	23	1	High-quality	99.95	0	Siphoviridae	prokaryote
v2198	33708	46	36	0	Medium-quality	75.99	0	Siphoviridae	prokaryote
v2199	45954	71	20	0	High-quality	100	0	unclassified	unclassified
v219a	44222	53	23	1	High-quality	100	0	Siphoviridae	prokaryote
v219b	26775	28	2	0	Medium-quality	59.53	0	unclassified	unclassified
v219c	56121	85	22	1	High-quality	100	0	Myoviridae	prokaryote
v219d	25994	39	6	1	High-quality	100	0	unclassified	unclassified
v219e	28969	48	14	0	Medium-quality	64.26	0	unclassified	unclassified
v219f	57922	104	14	2	Complete	100	0	unclassified	unclassified
v21a0	43342	66	20	1	Medium-quality	71	0	unclassified	unclassified
v21a1	145360	173	16	1	High-quality	94.5	0	unclassified	unclassified
v21a2	39308	53	17	1	Medium-quality	86.91	0	Myoviridae	prokaryote
v21a3	77414	114	14	3	High-quality	98.62	0	Quimbyviridae	prokaryote
v21a4	40958	78	18	0	Complete	100	0	unclassified	unclassified
v21a5	138826	160	31	2	Medium-quality	87.32	0	unclassified	unclassified
v21a6	21966	35	2	1	Medium-quality	61.37	0	unclassified	unclassified
v21a7	39019	53	15	0	High-quality	98.36	0	unclassified	unclassified
v21a8	22158	35	12	0	Medium-quality	56.79	0	Siphoviridae	prokaryote
v21a9	47665	71	27	3	High-quality	100	0	Siphoviridae	prokaryote
v21aa	35699	51	21	1	High-quality	100	0	Siphoviridae	prokaryote
v21ab	32070	48	24	0	Medium-quality	80.42	0	Siphoviridae	prokaryote
v21ac	41141	57	37	2	Medium-quality	74.32	0	Myoviridae	prokaryote
v21ad	34008	51	15	1	Medium-quality	76.67	0	Myoviridae	prokaryote
v21ae	24855	37	17	1	Medium-quality	68.06	0	Siphoviridae	prokaryote
v21af	106443	141	19	3	Complete	100	0	Gratiaviridae	prokaryote
v21b0	31932	59	12	2	Medium-quality	64.45	0	Siphoviridae	prokaryote
v21b1	39729	63	18	1	High-quality	100	0	Siphoviridae	prokaryote
v21b2	14186	20	5	2	High-quality	100	0	Siphoviridae	prokaryote
v21b3	38511	66	25	0	High-quality	95.96	0	Siphoviridae	prokaryote
v21b4	23202	25	4	1	Medium-quality	55.64	0	Siphoviridae	prokaryote
v21b5	37486	59	18	0	High-quality	95.98	0	unclassified	unclassified
v21b6	44554	56	18	5	High-quality	98.38	0	unclassified	unclassified
v21b7	14939	19	3	1	Medium-quality	52.08	0	unclassified	unclassified
v21b8	37453	62	23	0	Medium-quality	82.57	0	Siphoviridae	prokaryote
v21b9	212654	273	38	13	High-quality	100	0	unclassified	unclassified
v21ba	175296	232	41	12	Complete	100	0	unclassified	unclassified
v21bb	48933	73	15	9	High-quality	100	0	unclassified	unclassified

v21bc	30945	48	19	0	Medium-quality	51.54	0	Siphoviridae	prokaryote
v21bd	26842	19	3	1	Medium-quality	74.44	0	unclassified	unclassified
v21be	27874	49	18	0	Medium-quality	73.99	0	Siphoviridae	prokaryote
v21bf	62504	92	29	1	High-quality	100	0	Myoviridae	prokaryote
v21c0	29934	48	16	0	Medium-quality	76.84	0	Siphoviridae	prokaryote
v21c1	98375	147	16	1	Complete	100	0	Podoviridae_crAss-like	prokaryote
v21c2	22801	48	8	1	Medium-quality	50.79	0	unclassified	unclassified
v21c3	38998	58	17	0	Medium-quality	63.63	0	Siphoviridae	prokaryote
v21c4	111018	116	25	20	High-quality	100	0	Siphoviridae	prokaryote
v21c5	49867	68	17	1	Medium-quality	58.01	0	unclassified	unclassified
v21c6	84186	146	29	3	High-quality	100	0	unclassified	unclassified
v21c7	49596	76	21	0	High-quality	91.66	0	Myoviridae	prokaryote
v21c8	109629	164	12	5	Complete	100	0	unclassified	unclassified
v21c9	46306	64	31	0	High-quality	100	0	Siphoviridae	prokaryote
v21ca	21382	24	3	2	Medium-quality	74.53	0	unclassified	unclassified
v21cb	40667	57	21	0	High-quality	99.94	0	Siphoviridae	prokaryote
v21cc	16980	23	2	1	Complete	100	0	unclassified	unclassified
v21cd	110997	138	36	10	High-quality	100	0	Siphoviridae	prokaryote
v21ce	88260	120	24	0	Medium-quality	70.73	0	unclassified	unclassified
v21cf	54623	62	3	1	Medium-quality	64.71	0	unclassified	unclassified
v21d0	41660	73	30	2	High-quality	96.7	0	unclassified	unclassified
v21d1	28860	37	13	3	Medium-quality	70	0	Siphoviridae	prokaryote
v21d2	86424	93	16	3	Medium-quality	51.58	0	unclassified	unclassified
v21d3	24240	38	14	1	Medium-quality	59.75	0	Siphoviridae	prokaryote
v21d4	15419	19	4	1	Medium-quality	66.21	0	unclassified	unclassified
v21d5	16900	21	7	0	High-quality	100	0	unclassified	unclassified
v21d6	76605	97	12	7	High-quality	100	0	Quimbyviridae	prokaryote
v21d7	37167	52	25	0	Complete	100	0	Siphoviridae	prokaryote
v21d8	42797	74	8	0	Medium-quality	61.59	0	Quimbyviridae	prokaryote
v21d9	42475	51	10	0	High-quality	100	0	unclassified	unclassified
v21da	42367	64	23	1	High-quality	100	0	Siphoviridae	prokaryote
v21db	29750	46	21	0	Medium-quality	68.12	0	Myoviridae	prokaryote
v21dc	9765	13	1	0	High-quality	100	0	unclassified	unclassified
v21dd	76071	88	21	5	High-quality	100	0	Myoviridae	prokaryote
v21de	40966	55	14	2	Complete	100	0	Siphoviridae	prokaryote
v21df	162360	226	34	7	High-quality	96.91	0	unclassified	unclassified
v21e0	35662	42	7	0	High-quality	90.1	0	unclassified	unclassified
v21e1	15660	8	1	0	High-quality	100	0	unclassified	unclassified
v21e2	5523	2	1	0	Medium-quality	51.49	0	Retroviridae	eukaryote
v21e3	7076	6	3	0	Medium-quality	80.46	0	Retroviridae	eukaryote

v21e4	6384	6	1	0	Medium-quality	74.89	0	Retroviridae	eukaryote
v21e5	21841	25	20	0	Medium-quality	53.5	0	Siphoviridae	prokaryote
v21e6	74291	94	25	8	High-quality	100	0	Myoviridae	prokaryote
v21e7	113238	122	40	2	High-quality	100	0	Siphoviridae	prokaryote
v21e8	29103	44	23	0	Medium-quality	73.12	0	Siphoviridae	prokaryote
v21e9	37779	59	17	1	High-quality	100	0	Siphoviridae	prokaryote
v21ea	5090	6	2	0	Medium-quality	59.45	0	unclassified	unclassified
v21eb	17120	24	6	1	Medium-quality	77.55	0	unclassified	unclassified
v21ec	6171	8	1	0	High-quality	99.52	0	unclassified	unclassified
v21ed	11379	13	1	0	High-quality	100	0	unclassified	unclassified
v21ee	6655	10	1	0	High-quality	100	0	unclassified	unclassified
v21ef	9484	8	1	0	High-quality	100	0	unclassified	unclassified
v21f0	9695	10	2	0	High-quality	99.79	0	Retroviridae	eukaryote
v21f1	6199	6	1	0	High-quality	99.97	0	unclassified	unclassified
v21f2	43866	70	49	0	High-quality	93.16	0	Myoviridae	prokaryote
v21f3	6121	8	1	0	High-quality	98.71	0	unclassified	unclassified
v21f4	48498	71	24	3	Medium-quality	82.09	0	Siphoviridae	prokaryote
v21f5	6597	10	2	0	High-quality	100	0	unclassified	unclassified
v21f6	5115	10	1	0	Medium-quality	82.49	0	unclassified	unclassified
v21f7	35181	50	17	0	Complete	100	0	unclassified	unclassified
v21f8	45799	77	23	0	Complete	100	0	unclassified	unclassified
v21f9	11125	15	1	0	High-quality	100	0	unclassified	unclassified
v21fa	9994	6	1	0	High-quality	100	0	unclassified	unclassified
v21fb	17670	22	6	0	Medium-quality	81.06	0	unclassified	unclassified
v21fc	11922	9	1	0	Medium-quality	62.9	0	unclassified	unclassified
v21fd	7545	7	2	0	Medium-quality	88.51	0	unclassified	unclassified
v21fe	8284	4	2	0	Medium-quality	63.68	0	Retroviridae	eukaryote
v21ff	26446	44	25	0	Medium-quality	66.94	0	Siphoviridae	prokaryote
v2200	32007	45	30	1	Medium-quality	68.79	0	Siphoviridae	prokaryote
v2201	51906	50	15	1	Medium-quality	57.56	0	Myoviridae	prokaryote
v2202	44361	62	15	3	High-quality	100	0	unclassified	unclassified
v2203	10589	10	3	0	High-quality	98.98	0	Retroviridae	eukaryote
v2204	10835	12	1	0	High-quality	100	0	unclassified	unclassified
v2205	5100	5	1	0	Medium-quality	82.24	0	Retroviridae	eukaryote
v2206	6307	7	1	0	Medium-quality	73.99	0	unclassified	unclassified
v2207	9946	9	1	0	High-quality	92.73	0	unclassified	unclassified
v2208	6767	14	1	0	High-quality	100	0	unclassified	unclassified
v2209	12442	10	2	0	High-quality	98.31	0	unclassified	unclassified
v220a	11639	16	1	0	High-quality	100	0	unclassified	unclassified
v220b	7632	9	1	0	High-quality	100	0	unclassified	unclassified

v220c	6258	4	1	0	Medium-quality	73.41	0	unclassified	unclassified
v220d	8481	6	1	0	High-quality	100	0	unclassified	unclassified
v220e	30802	19	1	0	High-quality	100	0	unclassified	unclassified
v220f	33964	50	32	0	Medium-quality	75.57	0	Siphoviridae	prokaryote
v2210	5129	4	1	0	Medium-quality	60.17	0	Retroviridae	eukaryote
v2211	21373	28	11	0	Medium-quality	52.82	0	unclassified	unclassified
v2212	47514	68	34	1	High-quality	100	0	Siphoviridae	prokaryote
v2213	32774	37	14	0	Medium-quality	79.39	0	unclassified	unclassified
v2214	10795	8	1	0	High-quality	100	0	Retroviridae	eukaryote
v2215	6514	6	1	0	High-quality	100	0	unclassified	unclassified
v2216	11569	7	1	0	High-quality	100	0	Retroviridae	eukaryote
v2217	9527	9	2	0	Medium-quality	74.57	0	Bornaviridae	eukaryote
v2218	27371	54	27	0	Medium-quality	65.85	0	Siphoviridae	prokaryote
v2219	22534	31	24	0	Medium-quality	56.32	0	Siphoviridae	prokaryote
v221a	26148	35	21	0	Medium-quality	64.45	0	Myoviridae	prokaryote
v221b	5461	8	1	0	Medium-quality	88.07	0	unclassified	unclassified
v221c	5436	8	5	0	Medium-quality	64.17	0	Retroviridae	eukaryote
v221d	5869	12	1	0	High-quality	94.65	0	unclassified	unclassified
v221e	6010	7	1	0	Medium-quality	56.03	0	unclassified	unclassified
v221f	5043	7	1	0	Medium-quality	81.33	0	unclassified	unclassified
v2220	27145	43	32	0	Medium-quality	81.66	0	Siphoviridae	prokaryote
v2221	60180	101	53	0	Complete	100	0	Siphoviridae	prokaryote
v2222	8825	14	2	0	High-quality	100	0	unclassified	unclassified
v2223	23075	34	16	0	Medium-quality	59.24	0	Siphoviridae	prokaryote
v2224	10830	13	2	1	High-quality	90.67	0	unclassified	unclassified
v2225	50925	88	15	0	High-quality	93.9	0	unclassified	unclassified
v2226	41734	49	20	0	High-quality	100	0	Siphoviridae	prokaryote
v2227	7567	11	4	0	Medium-quality	88.77	0	unclassified	unclassified
v2228	33293	39	10	3	Medium-quality	73.33	0	Siphoviridae	prokaryote
v2229	10463	4	1	0	High-quality	91.44	0	unclassified	unclassified
v222a	15138	10	2	0	High-quality	100	0	Retroviridae	eukaryote
v222b	11531	12	2	0	High-quality	100	0	unclassified	unclassified
v222c	17874	24	9	0	Complete	100	0	Salasmaviridae	prokaryote
v222d	25461	32	18	0	Medium-quality	61.8	0	Siphoviridae	prokaryote
v222e	32802	56	41	2	Medium-quality	63.42	0	Siphoviridae	prokaryote
v222f	57513	86	18	0	High-quality	95.62	0	unclassified	unclassified
v2230	15945	22	6	0	Medium-quality	73.27	0	unclassified	unclassified
v2231	10464	12	1	0	High-quality	100	0	unclassified	unclassified
v2232	5602	7	2	0	Medium-quality	65.71	0	Retroviridae	eukaryote
v2233	7194	8	1	0	High-quality	100	0	unclassified	unclassified

v2234	8093	7	1	0	High-quality	94.94	0	unclassified	unclassified
v2235	7123	8	1	0	Medium-quality	66.41	0	Retroviridae	eukaryote
v2236	13555	7	1	0	High-quality	100	0	unclassified	unclassified
v2237	7071	11	2	0	Medium-quality	82.59	0	Retroviridae	eukaryote
v2238	5819	9	1	0	High-quality	93.84	0	unclassified	unclassified
v2239	41531	55	35	0	Complete	100	0	Siphoviridae	prokaryote
v223a	5903	7	1	0	High-quality	95.19	0	unclassified	unclassified
v223b	52712	78	20	1	High-quality	90.86	0	unclassified	unclassified
v223c	55853	98	17	3	High-quality	96.84	0	unclassified	unclassified
v223d	59186	87	49	1	Complete	100	0	Siphoviridae	prokaryote
v223e	52048	86	18	2	High-quality	100	0	unclassified	unclassified
v223f	19779	23	5	4	Medium-quality	68.94	0	Siphoviridae	prokaryote
v2240	48905	77	34	0	Complete	100	0	Zobellviridae	prokaryote
v2241	40522	39	14	2	Medium-quality	53.93	0	Siphoviridae	prokaryote
v2242	77516	121	16	4	High-quality	100	0	unclassified	unclassified
v2243	54762	94	20	2	High-quality	100	0	unclassified	unclassified
v2244	38512	55	16	2	High-quality	100	0	Myoviridae	prokaryote
v2245	25300	25	8	1	Medium-quality	62.41	0	unclassified	unclassified
v2246	24395	41	33	0	Medium-quality	60.06	0	Podoviridae	prokaryote
v2247	43906	57	13	1	Complete	100	0	unclassified	unclassified
v2248	5177	9	5	0	Complete	100	0	Microviridae	prokaryote
v2249	34546	51	11	4	High-quality	99.16	0	unclassified	unclassified
v224a	34939	48	14	2	High-quality	100	0	Siphoviridae	prokaryote
v224b	14732	17	4	2	Medium-quality	51.35	0	unclassified	unclassified
v224c	22996	27	9	0	Medium-quality	54.51	0	Siphoviridae	prokaryote
v224d	26831	38	20	0	Medium-quality	55.95	0	Siphoviridae	prokaryote
v224e	79528	104	12	2	Complete	100	0	unclassified	unclassified
v224f	56016	85	18	1	High-quality	100	0	Myoviridae	prokaryote
v2250	45662	60	16	2	High-quality	100	0	unclassified	unclassified
v2251	14352	20	5	0	High-quality	100	0	unclassified	unclassified
v2252	43892	81	21	1	Complete	100	0	unclassified	unclassified
v2253	36736	54	18	1	Complete	100	0	unclassified	unclassified
v2254	95250	112	14	13	High-quality	94.31	0	unclassified	unclassified
v2255	33503	45	15	0	Medium-quality	82.55	0	unclassified	unclassified
v2256	38256	51	21	3	High-quality	93.35	0	Myoviridae	prokaryote
v2257	11621	13	7	0	Medium-quality	63.15	0	unclassified	unclassified
v2258	127201	189	49	1	High-quality	100	0	unclassified	unclassified
v2259	72628	102	22	1	Complete	100	0	unclassified	unclassified
v225a	23249	27	11	0	Medium-quality	58.64	0	unclassified	unclassified
v225b	60722	78	28	0	High-quality	100	0	Siphoviridae	prokaryote

v225c	39171	60	14	1	High-quality	100	0	unclassified	unclassified
v225d	15089	17	4	0	High-quality	100	0	unclassified	unclassified
v225e	5953	9	6	0	Complete	100	0	Microviridae	prokaryote
v225f	25922	29	13	0	Medium-quality	58.31	0	Siphoviridae	prokaryote
v2260	37295	62	15	0	High-quality	100	0	unclassified	unclassified
v2261	7078	8	1	0	High-quality	100	0	unclassified	unclassified
v2262	93829	134	15	3	High-quality	100	0	Quimbyviridae	prokaryote
v2263	64010	103	25	1	Complete	100	0	unclassified	unclassified
v2264	43676	51	8	5	High-quality	100	0	unclassified	unclassified
v2265	36293	43	12	6	Medium-quality	80.92	0	unclassified	unclassified
v2266	39154	54	34	0	Complete	100	0	Siphoviridae	prokaryote
v2267	33494	50	30	0	Complete	100	0	Siphoviridae	prokaryote
v2268	27989	42	19	0	Medium-quality	64.44	0	Myoviridae	prokaryote
v2269	34287	58	14	2	Complete	100	0	unclassified	unclassified
v226a	17086	22	10	0	Medium-quality	73	0	unclassified	unclassified
v226b	32234	50	13	1	Complete	100	0	unclassified	unclassified
v226c	27648	28	1	0	Medium-quality	56.36	0	unclassified	unclassified
v226d	36642	40	11	0	Medium-quality	73.65	0	Siphoviridae	prokaryote
v226e	42169	53	19	0	Medium-quality	84.81	0	Siphoviridae	prokaryote
v226f	42944	51	22	0	High-quality	100	0	unclassified	unclassified
v2270	16449	17	7	0	Medium-quality	87.43	0	unclassified	unclassified
v2271	52347	78	29	0	High-quality	91.18	0	Siphoviridae	prokaryote
v2272	35487	43	29	0	Medium-quality	86.2	0	Siphoviridae	prokaryote
v2273	15858	19	7	0	High-quality	100	0	unclassified	unclassified
v2274	10935	12	2	1	High-quality	91.55	0	unclassified	unclassified
v2275	34591	40	14	1	Medium-quality	56.6	0	Myoviridae	prokaryote
v2276	18722	30	11	0	Medium-quality	50.64	0	Siphoviridae	prokaryote
v2277	34394	43	12	0	Medium-quality	87.36	0	unclassified	unclassified
v2278	66217	78	14	2	High-quality	98.46	0	unclassified	unclassified
v2279	19839	31	13	0	Medium-quality	52.43	0	Siphoviridae	prokaryote
v227a	6903	12	5	1	High-quality	100	0	Microviridae	prokaryote
v227b	36140	57	14	0	High-quality	100	0	Siphoviridae	prokaryote
v227c	95099	107	20	6	High-quality	94.29	0	unclassified	unclassified
v227d	24970	33	10	0	Medium-quality	55.94	0	unclassified	unclassified
v227e	36921	55	18	3	High-quality	100	0	unclassified	unclassified
v227f	87954	98	25	6	High-quality	100	0	Siphoviridae	prokaryote
v2280	31244	46	9	1	Medium-quality	54.21	0	unclassified	unclassified
v2281	49631	80	19	0	High-quality	100	0	unclassified	unclassified
v2282	41185	73	20	1	High-quality	98	0	unclassified	unclassified
v2283	10544	18	5	0	Medium-quality	89.58	0	Siphoviridae	prokaryote

v2284	151610	196	50	5	High-quality	99.33	0	unclassified	unclassified
v2285	87804	134	15	2	High-quality	99.63	0	Quimbyviridae	prokaryote
v2286	13633	18	7	0	Complete	100	0	unclassified	unclassified
v2287	56859	104	17	1	Complete	100	0	unclassified	unclassified
v2288	59363	83	8	1	High-quality	100	0	unclassified	unclassified
v2289	15368	23	8	0	Medium-quality	70.97	0	unclassified	unclassified
v228a	14517	22	4	0	Complete	100	0	unclassified	unclassified
v228b	13528	17	6	0	Medium-quality	74.93	0	unclassified	unclassified
v228c	10530	7	5	0	High-quality	100	0	unclassified	unclassified
v228d	6153	9	2	0	High-quality	100	0	unclassified	unclassified
v228e	18168	24	10	0	Medium-quality	83.26	0	unclassified	unclassified
v228f	6174	6	3	0	High-quality	100	0	unclassified	unclassified
v2290	5920	10	4	0	High-quality	100	0	Microviridae	prokaryote
v2291	5593	10	6	0	High-quality	100	0	Microviridae	prokaryote
v2292	103240	140	35	2	Complete	100	0	unclassified	unclassified
v2293	46473	94	23	1	High-quality	100	0	unclassified	unclassified
v2294	13397	15	4	0	High-quality	100	0	Podoviridae	prokaryote
v2295	10375	13	3	0	Medium-quality	74.7	0	unclassified	unclassified
v2296	6121	8	3	0	Complete	100	0	unclassified	unclassified
v2297	62308	81	26	5	High-quality	100	0	Podoviridae_crAss-like	prokaryote
v2298	39902	56	22	2	Complete	100	0	Siphoviridae	prokaryote
v2299	12887	17	5	0	Complete	100	0	unclassified	unclassified
v229a	6675	7	2	0	High-quality	100	0	unclassified	unclassified
v229b	5874	7	6	0	High-quality	100	0	Microviridae	prokaryote
v229c	43053	75	24	1	High-quality	97.13	0	unclassified	unclassified
v229d	15799	18	8	0	Medium-quality	83.88	0	Salasmaviridae	prokaryote
v229e	37590	45	16	0	High-quality	92.24	0	Siphoviridae	prokaryote
v229f	33357	45	12	0	Complete	100	0	unclassified	unclassified
v22a0	15262	22	8	0	Medium-quality	79.19	0	unclassified	unclassified
v22a1	15179	20	9	0	Medium-quality	65.81	0	Salasmaviridae	prokaryote
v22a2	5357	7	4	0	Complete	100	0	Microviridae	prokaryote
v22a3	5253	9	4	0	High-quality	92.81	0	Microviridae	prokaryote
v22a4	44931	80	20	0	High-quality	100	0	unclassified	unclassified
v22a5	25817	46	20	0	Medium-quality	78.31	0	Siphoviridae	prokaryote
v22a6	17091	22	8	0	Complete	100	0	unclassified	unclassified
v22a7	73455	101	42	0	Complete	100	0	Siphoviridae	prokaryote
v22a8	33899	42	13	1	High-quality	99.34	0	Siphoviridae	prokaryote
v22a9	26261	50	14	0	Medium-quality	63.1	0	Siphoviridae	prokaryote
v22aa	17542	27	8	0	Complete	100	0	unclassified	unclassified
v22ab	13439	20	4	0	Complete	100	0	unclassified	unclassified

v22ac	11603	17	9	0	High-quality	100	0	Microviridae	prokaryote
v22ad	50019	72	30	1	Complete	100	0	unclassified	unclassified
v22ae	44845	71	19	0	Complete	100	0	unclassified	unclassified
v22af	11615	13	7	0	Medium-quality	83.63	0	unclassified	unclassified
v22b0	6140	5	3	0	High-quality	96.83	0	Microviridae	prokaryote
v22b1	43068	58	4	1	Complete	100	0	unclassified	unclassified
v22b2	8581	13	4	0	High-quality	100	0	Microviridae	prokaryote
v22b3	5859	9	4	0	High-quality	100	0	Microviridae	prokaryote
v22b4	16836	24	6	0	Medium-quality	88.34	0	unclassified	unclassified
v22b5	12459	17	4	0	Complete	100	0	unclassified	unclassified
v22b6	11892	18	4	0	High-quality	100	0	unclassified	unclassified
v22b7	11537	14	5	0	Complete	100	0	Podoviridae	prokaryote
v22b8	6198	10	2	0	High-quality	100	0	unclassified	unclassified
v22b9	5135	9	4	0	Medium-quality	85.71	0	unclassified	unclassified
v22ba	59459	98	35	1	High-quality	96.93	0	Siphoviridae	prokaryote
v22bb	6292	7	3	0	Complete	100	0	unclassified	unclassified
v22bc	100582	166	54	0	Complete	100	0	unclassified	unclassified
v22bd	13414	18	8	0	Medium-quality	69.57	0	Salasmaviridae	prokaryote
v22be	5852	9	4	0	High-quality	94.28	0	unclassified	unclassified
v22bf	44298	77	16	1	High-quality	98.34	0	unclassified	unclassified
v22c0	22808	25	13	1	Medium-quality	56.8	0	Siphoviridae	prokaryote
v22c1	7392	9	4	0	High-quality	100	0	unclassified	unclassified
v22c2	6892	10	3	0	High-quality	100	0	unclassified	unclassified
v22c3	151694	196	50	5	High-quality	99.38	0	unclassified	unclassified
v22c4	11878	15	5	0	High-quality	100	0	Podoviridae	prokaryote
v22c5	6822	7	3	0	Complete	100	0	unclassified	unclassified
v22c6	6155	9	2	0	High-quality	100	0	unclassified	unclassified
v22c7	29885	49	7	1	Medium-quality	72.67	0	unclassified	unclassified
v22c8	96366	165	15	1	Complete	100	0	Podoviridae_crAss-like	prokaryote
v22c9	112399	149	13	4	High-quality	100	0	unclassified	unclassified
v22ca	34962	42	20	2	Medium-quality	74.72	0	Siphoviridae	prokaryote
v22cb	39609	60	30	0	Medium-quality	63.19	0	Myoviridae	prokaryote
v22cc	157453	203	14	3	Complete	100	0	unclassified	unclassified
v22cd	138243	182	41	3	Complete	100	0	unclassified	unclassified
v22ce	58652	97	17	2	Complete	100	0	unclassified	unclassified
v22cf	42444	61	20	1	High-quality	100	0	Siphoviridae	prokaryote
v22d0	33453	45	21	0	Medium-quality	75.21	0	Siphoviridae	prokaryote
v22d1	119847	153	41	2	High-quality	100	0	unclassified	unclassified
v22d2	44076	61	24	1	High-quality	100	0	Siphoviridae	prokaryote
v22d3	218677	353	42	4	High-quality	100	0	unclassified	unclassified

v22d4	28229	43	10	0	Medium-quality	61.73	0	Siphoviridae	prokaryote
v22d5	39069	75	16	2	Complete	100	0	unclassified	unclassified
v22d6	38817	73	16	0	Medium-quality	86.15	0	unclassified	unclassified
v22d7	124451	164	35	5	Medium-quality	85.51	0	unclassified	unclassified
v22d8	40318	80	19	0	High-quality	100	0	unclassified	unclassified
v22d9	155126	190	17	3	Complete	100	0	unclassified	unclassified
v22da	35690	53	18	5	High-quality	95.01	0	Siphoviridae	prokaryote
v22db	39000	54	24	1	High-quality	100	0	Siphoviridae	prokaryote
v22dc	105699	138	20	4	Complete	100	0	Gratiaviridae	prokaryote
v22dd	38455	65	20	0	High-quality	96	0	Siphoviridae	prokaryote
v22de	96672	163	15	1	Complete	100	0	Podoviridae_crAss-like	prokaryote
v22df	178116	273	23	7	Complete	100	0	unclassified	unclassified
v22e0	64066	74	8	0	Medium-quality	69.49	0	unclassified	unclassified
v22e1	142343	176	33	1	Complete	100	0	unclassified	unclassified
v22e2	35365	51	18	0	High-quality	90.71	0	Siphoviridae	prokaryote
v22e3	46331	56	11	0	Complete	100	0	Siphoviridae	prokaryote
v22e4	34178	49	25	0	Medium-quality	79.59	0	Myoviridae	prokaryote
v22e5	43649	73	18	1	High-quality	96.62	0	unclassified	unclassified
v22e6	97566	130	26	1	Complete	100	0	unclassified	unclassified
v22e7	40901	74	33	0	High-quality	95.08	0	Myoviridae	prokaryote
v22e8	55490	69	8	3	Medium-quality	63.06	0	Quimbyviridae	prokaryote
v22e9	68750	66	15	9	High-quality	100	0	Siphoviridae	prokaryote
v22ea	42901	57	18	1	High-quality	100	0	Siphoviridae	prokaryote
v22eb	159002	199	16	4	Complete	100	0	unclassified	unclassified
v22ec	35219	61	18	0	High-quality	98.7	0	unclassified	unclassified
v22ed	43498	72	21	1	Complete	100	0	unclassified	unclassified
v22ee	31238	46	13	0	Medium-quality	69.6	0	Myoviridae	prokaryote
v22ef	15866	20	6	0	Medium-quality	54.5	0	unclassified	unclassified
v22f0	35862	48	23	1	High-quality	100	0	Siphoviridae	prokaryote
v22f1	79411	115	13	0	Complete	100	0	Flandersviridae	prokaryote
v22f2	77579	111	15	0	Complete	100	0	Flandersviridae	prokaryote
v22f3	26421	36	14	0	Medium-quality	63.35	0	Siphoviridae	prokaryote
v22f4	11284	16	4	0	Medium-quality	88.88	0	unclassified	unclassified
v22f5	12708	15	6	1	Medium-quality	62.01	0	Podoviridae	prokaryote
v22f6	29561	44	5	0	Medium-quality	84.57	0	unclassified	unclassified
v22f7	17097	22	8	0	Medium-quality	75.14	0	unclassified	unclassified
v22f8	98734	162	18	1	Complete	100	0	Podoviridae_crAss-like	prokaryote
v22f9	59476	59	15	5	High-quality	100	0	Siphoviridae	prokaryote
v22fa	46222	61	5	2	Medium-quality	52.36	0	Quimbyviridae	prokaryote
v22fb	92525	113	11	2	High-quality	96.6	0	unclassified	unclassified

v22fc	27392	42	19	0	Medium-quality	67.5	0	Siphoviridae	prokaryote
v22fd	55246	79	26	7	Medium-quality	82.62	0	Myoviridae	prokaryote
v22fe	33797	52	16	2	High-quality	98.87	0	Siphoviridae	prokaryote
v22ff	48185	57	9	7	High-quality	100	0	unclassified	unclassified
v2300	37485	42	5	4	Medium-quality	60.68	0	unclassified	unclassified
v2301	41981	50	24	3	Medium-quality	78.54	0	Siphoviridae	prokaryote
v2302	16056	18	5	0	Medium-quality	53.45	0	unclassified	unclassified
v2303	37550	65	13	1	Medium-quality	83.21	0	unclassified	unclassified
v2304	15372	25	6	0	Medium-quality	82.44	0	unclassified	unclassified
v2305	55972	77	21	3	High-quality	100	0	Myoviridae	prokaryote
v2306	40374	66	11	1	High-quality	98.9	0	unclassified	unclassified
v2307	26519	33	3	0	Medium-quality	61.08	0	unclassified	unclassified
v2308	89095	104	12	4	High-quality	100	0	unclassified	unclassified
v2309	40892	60	12	0	High-quality	97.97	0	unclassified	unclassified
v230a	41773	45	20	0	High-quality	99.96	0	Siphoviridae	prokaryote
v230b	8645	11	3	0	Medium-quality	55.23	0	unclassified	unclassified
v230c	40193	60	15	0	High-quality	100	0	unclassified	unclassified
v230d	52073	78	18	1	High-quality	98.61	0	unclassified	unclassified
v230e	17180	24	6	0	Medium-quality	77.93	0	unclassified	unclassified
v230f	42346	79	13	0	Complete	100	0	unclassified	unclassified
v2310	41076	59	24	1	High-quality	100	0	Siphoviridae	prokaryote
v2311	56046	101	19	1	High-quality	100	0	unclassified	unclassified
v2312	22290	36	11	0	Medium-quality	62.28	0	Myoviridae	prokaryote
v2313	17599	23	3	2	Medium-quality	61.34	0	Siphoviridae	prokaryote
v2314	41364	62	20	1	High-quality	92.16	0	Myoviridae	prokaryote
v2315	38891	69	18	0	High-quality	94.05	0	unclassified	unclassified
v2316	55444	98	20	1	Complete	100	0	unclassified	unclassified
v2317	40156	74	17	0	High-quality	94.95	0	unclassified	unclassified
v2318	30413	37	24	0	Medium-quality	57.83	0	Myoviridae	prokaryote
v2319	31508	52	21	1	Medium-quality	71.71	0	unclassified	unclassified
v231a	40516	77	21	0	Complete	100	0	Siphoviridae	prokaryote
v231b	27297	43	15	1	Medium-quality	73.1	0	Siphoviridae	prokaryote
v231c	31626	50	8	0	High-quality	90.56	0	unclassified	unclassified
v231d	75964	121	20	7	Complete	100	0	unclassified	unclassified
v231e	87690	115	15	2	Complete	100	0	Flandersviridae	prokaryote
v231f	133214	162	38	4	High-quality	100	0	unclassified	unclassified
v2320	41263	53	30	2	High-quality	99.87	0	Siphoviridae	prokaryote
v2321	26242	34	17	0	Medium-quality	63.78	0	Siphoviridae	prokaryote
v2322	40723	49	46	0	Complete	100	0	Autographiviridae	prokaryote
v2323	86231	116	31	13	High-quality	100	0	unclassified	unclassified

v2324	88977	119	28	0	Medium-quality	61	0	Siphoviridae	prokaryote
v2325	61717	79	17	1	High-quality	93.95	0	unclassified	unclassified
v2326	180466	219	26	2	Complete	100	0	unclassified	unclassified
v2327	41866	76	18	0	Complete	100	0	unclassified	unclassified
v2328	36868	52	16	2	High-quality	91.5	0	Siphoviridae	prokaryote
v2329	41872	56	16	2	Medium-quality	76	0	Myoviridae	prokaryote
v232a	33561	30	4	3	Medium-quality	72.83	0	unclassified	unclassified
v232b	47615	80	11	2	Medium-quality	78.58	0	unclassified	unclassified
v232c	35181	63	17	0	High-quality	100	0	unclassified	unclassified
v232d	41463	74	19	0	High-quality	98.05	0	unclassified	unclassified
v232e	49510	50	22	3	Medium-quality	87.36	0	Siphoviridae	prokaryote
v232f	43882	63	12	2	Medium-quality	59.17	0	unclassified	unclassified
v2330	35618	40	10	0	Medium-quality	74.07	0	unclassified	unclassified
v2331	15852	24	6	0	Medium-quality	69.15	0	unclassified	unclassified
v2332	73886	121	21	4	High-quality	96.46	0	unclassified	unclassified
v2333	8584	3	1	0	Medium-quality	83.4	0	unclassified	unclassified
v2334	400748	535	29	8	Complete	100	0	unclassified	unclassified
v2335	86857	116	22	6	Complete	100	0	unclassified	unclassified
v2336	43663	77	19	0	High-quality	100	0	unclassified	unclassified
v2337	69467	86	5	4	High-quality	100	0	unclassified	unclassified
v2338	76673	127	17	5	Complete	100	0	unclassified	unclassified
v2339	14487	15	6	0	Low-quality	48.76	0	Salasmaviridae	prokaryote
v233a	25535	32	2	1	Medium-quality	56.73	0	unclassified	unclassified
v233b	40646	62	18	0	Complete	100	0	Siphoviridae	prokaryote
v233c	59337	91	21	1	High-quality	98.64	0	unclassified	unclassified
v233d	79197	119	14	7	High-quality	100	0	unclassified	unclassified
v233e	36420	66	21	1	Complete	100	0	unclassified	unclassified
v233f	42923	79	28	0	Complete	100	0	Myoviridae	prokaryote
v2340	110583	145	23	5	Complete	100	0	Gratiaviridae	prokaryote
v2341	46196	77	32	2	High-quality	100	0	Myoviridae	prokaryote
v2342	47410	69	18	1	High-quality	100	0	unclassified	unclassified
v2343	31075	46	10	0	High-quality	91.25	0	unclassified	unclassified
v2344	45568	71	20	0	High-quality	100	0	unclassified	unclassified
v2345	40392	65	15	2	High-quality	100	0	Siphoviridae	prokaryote
v2346	45243	74	18	0	Complete	100	0	unclassified	unclassified
v2347	43365	67	16	1	Complete	100	0	unclassified	unclassified
v2348	12082	16	7	0	Medium-quality	56.07	0	unclassified	unclassified
v2349	45781	79	20	0	Complete	100	0	unclassified	unclassified
v234a	59256	99	14	2	Complete	100	0	unclassified	unclassified
v234b	33762	64	12	0	Complete	100	0	unclassified	unclassified

v234c	36991	47	11	0	Medium-quality	74.48	0	unclassified	unclassified
v234d	89415	146	18	3	High-quality	100	0	unclassified	unclassified
v234e	73766	133	17	4	High-quality	93.48	0	unclassified	unclassified
v234f	20628	27	13	0	Medium-quality	51.5	0	Siphoviridae	prokaryote
v2350	36848	36	10	3	Medium-quality	78.21	0	Siphoviridae	prokaryote
v2351	31209	41	22	0	Medium-quality	51.79	0	Siphoviridae	prokaryote
v2352	21027	18	13	0	Low-quality	48.35	0	Siphoviridae	prokaryote
v2353	30601	53	14	1	Medium-quality	67.91	0	unclassified	unclassified
v2354	42527	62	20	0	High-quality	94.25	0	unclassified	unclassified
v2355	36866	60	11	1	Complete	100	0	unclassified	unclassified
v2356	55666	97	20	1	Complete	100	0	unclassified	unclassified
v2357	44000	52	31	0	High-quality	100	0	Autographiviridae	prokaryote
v2358	29345	34	10	0	Complete	100	0	unclassified	unclassified
v2359	29650	36	9	4	Medium-quality	72.78	0	Siphoviridae	prokaryote
v235a	15321	20	8	0	Medium-quality	64.66	0	Salasmaviridae	prokaryote
v235b	48526	70	9	1	Medium-quality	82.68	0	unclassified	unclassified
v235c	72816	105	22	10	High-quality	100	0	unclassified	unclassified
v235d	53135	82	35	4	Complete	100	0	Myoviridae	prokaryote
v235e	47347	75	21	1	Complete	100	0	unclassified	unclassified
v235f	109387	161	133	0	Complete	100	0	Demerecviridae	prokaryote
v2360	72134	96	11	3	High-quality	100	0	unclassified	unclassified
v2361	38626	55	11	1	High-quality	100	0	unclassified	unclassified
v2362	34755	50	17	3	High-quality	100	0	Siphoviridae	prokaryote
v2363	44001	77	7	0	Medium-quality	55.57	0	Flandersviridae	prokaryote
v2364	37041	52	16	0	Complete	100	0	Siphoviridae	prokaryote
v2365	37240	61	12	1	Complete	100	0	unclassified	unclassified
v2366	45998	76	16	1	Complete	100	0	unclassified	unclassified
v2367	70755	95	17	2	High-quality	100	0	Siphoviridae	prokaryote
v2368	17019	20	10	0	Medium-quality	74.01	0	Salasmaviridae	prokaryote
v2369	61523	95	35	1	High-quality	100	0	Siphoviridae	prokaryote
v236a	44866	62	13	0	High-quality	99.09	0	Siphoviridae	prokaryote
v236b	47730	85	29	1	Complete	100	0	Myoviridae	prokaryote
v236c	94362	124	32	2	Medium-quality	77.1	0	Siphoviridae	prokaryote
v236d	60781	91	14	2	High-quality	100	0	unclassified	unclassified
v236e	34225	46	12	1	High-quality	90.66	0	unclassified	unclassified
v236f	45592	72	17	2	High-quality	100	0	unclassified	unclassified
v2370	9398	18	1	0	Complete	100	0	unclassified	unclassified
v2371	36307	61	17	0	Complete	100	0	unclassified	unclassified
v2372	45233	53	7	4	High-quality	100	0	unclassified	unclassified
v2373	41199	80	14	0	Complete	100	0	unclassified	unclassified

v2374	179745	230	20	5	High-quality	99.94	0	unclassified	unclassified
v2375	40349	52	8	2	Medium-quality	53.85	0	unclassified	unclassified
v2376	16181	19	8	0	Medium-quality	84.24	0	Salasmaviridae	prokaryote
v2377	62384	81	33	4	High-quality	100	0	Myoviridae	prokaryote
v2378	54774	101	15	0	High-quality	93.41	0	unclassified	unclassified
v2379	64249	99	21	1	High-quality	100	0	Myoviridae	prokaryote
v237a	49390	78	13	0	Medium-quality	80.57	0	unclassified	unclassified
v237b	82039	79	24	6	High-quality	100	0	Siphoviridae	prokaryote
v237c	41852	66	11	1	Medium-quality	52.06	0	unclassified	unclassified
v237d	73590	117	15	4	Complete	100	0	unclassified	unclassified
v237e	5668	8	2	0	Complete	100	0	unclassified	unclassified
v237f	43076	71	18	0	High-quality	100	0	unclassified	unclassified
v2380	8940	13	1	0	High-quality	100	0	unclassified	unclassified
v2381	25109	38	19	0	Medium-quality	65.5	0	Siphoviridae	prokaryote
v2382	88749	107	32	3	Complete	100	0	Myoviridae	prokaryote
v2383	50311	79	9	3	Medium-quality	74.78	0	unclassified	unclassified
v2384	75118	115	16	1	Medium-quality	79.38	0	Quimbyviridae	prokaryote
v2385	16673	26	7	0	Complete	100	0	unclassified	unclassified
v2386	29761	40	15	1	Medium-quality	75.07	0	Siphoviridae	prokaryote
v2387	23453	39	10	2	Complete	100	0	unclassified	unclassified
v2388	13939	18	5	0	High-quality	100	0	unclassified	unclassified
v2389	55717	80	3	2	High-quality	100	0	unclassified	unclassified
v238a	59079	76	19	6	High-quality	100	0	unclassified	unclassified
v238b	61341	82	10	0	High-quality	100	0	unclassified	unclassified
v238c	51432	65	12	1	High-quality	95.87	0	unclassified	unclassified
v238d	43135	66	16	1	Complete	100	0	unclassified	unclassified
v238e	38393	76	18	0	Complete	100	0	Siphoviridae	prokaryote
v238f	59991	77	13	5	High-quality	100	0	Myoviridae	prokaryote
v2390	41894	61	10	0	High-quality	98.18	0	unclassified	unclassified
v2391	19478	32	4	0	Medium-quality	53.31	0	unclassified	unclassified
v2392	61025	89	17	0	Medium-quality	70.83	0	unclassified	unclassified
v2393	14169	27	3	1	Medium-quality	67.28	0	unclassified	unclassified
v2394	42120	50	17	2	High-quality	100	0	Siphoviridae	prokaryote
v2395	31540	42	24	0	Medium-quality	52.41	0	Siphoviridae	prokaryote
v2396	87482	137	23	3	High-quality	100	0	unclassified	unclassified
v2397	38573	59	9	1	Complete	100	0	unclassified	unclassified
v2398	45906	83	19	0	High-quality	100	0	unclassified	unclassified
v2399	41441	79	13	0	Complete	100	0	unclassified	unclassified
v239a	41884	81	16	0	High-quality	100	0	unclassified	unclassified
v239b	45268	79	22	1	Complete	100	0	unclassified	unclassified

v239c	57218	89	24	1	High-quality	100	0	unclassified	unclassified
v239d	175851	208	41	7	Complete	100	0	unclassified	unclassified
v239e	44807	78	14	3	Complete	100	0	unclassified	unclassified
v239f	42735	65	15	1	High-quality	100	0	unclassified	unclassified
v23a0	35101	51	11	1	Complete	100	0	unclassified	unclassified
v23a1	51238	55	26	3	Medium-quality	86.02	0	Siphoviridae	prokaryote
v23a2	38142	72	19	2	Complete	100	0	unclassified	unclassified
v23a3	7663	14	3	0	Medium-quality	55.51	0	unclassified	unclassified
v23a4	35252	59	15	0	High-quality	98.87	0	Siphoviridae	prokaryote
v23a5	24113	29	18	0	Medium-quality	71.15	0	Siphoviridae	prokaryote
v23a6	77843	131	27	2	Complete	100	0	unclassified	unclassified
v23a7	39977	62	18	3	High-quality	96.25	0	Siphoviridae	prokaryote
v23a8	39027	51	25	0	High-quality	92.33	0	Siphoviridae	prokaryote
v23a9	39060	52	36	1	High-quality	97	0	Podoviridae	prokaryote
v23aa	34880	57	13	0	Complete	100	0	unclassified	unclassified
v23ab	45684	74	17	1	Complete	100	0	unclassified	unclassified
v23ac	35077	69	15	1	High-quality	97.26	0	unclassified	unclassified
v23ad	16579	19	7	0	Medium-quality	87.22	0	Salasmaviridae	prokaryote
v23ae	53255	93	19	0	Complete	100	0	unclassified	unclassified
v23af	15430	20	7	0	Medium-quality	80.08	0	unclassified	unclassified
v23b0	41401	50	9	4	Medium-quality	50.29	0	Siphoviridae	prokaryote
v23b1	110204	162	34	0	Complete	100	0	unclassified	unclassified
v23b2	43014	81	16	1	Complete	100	0	unclassified	unclassified
v23b3	38546	65	13	1	Complete	100	0	unclassified	unclassified
v23b4	33673	50	27	0	Complete	100	0	Siphoviridae	prokaryote
v23b5	6605	7	4	0	Complete	100	0	unclassified	unclassified
v23b6	48795	64	11	1	Complete	100	0	unclassified	unclassified
v23b7	115968	186	22	8	Medium-quality	69.25	0	unclassified	unclassified
v23b8	113407	146	18	7	Complete	100	0	Gratiaviridae	prokaryote
v23b9	28075	42	13	0	Medium-quality	61.87	0	unclassified	unclassified
v23ba	38336	60	21	0	High-quality	90.48	0	unclassified	unclassified
v23bb	52828	78	13	1	Medium-quality	71.51	0	unclassified	unclassified
v23bc	80045	128	19	1	Complete	100	0	unclassified	unclassified
v23bd	58642	88	9	2	High-quality	100	0	unclassified	unclassified
v23be	34988	37	10	0	Medium-quality	89.16	0	unclassified	unclassified
v23bf	102162	129	20	1	Complete	100	0	Gratiaviridae	prokaryote
v23c0	41231	56	8	0	Complete	100	0	unclassified	unclassified
v23c1	45886	80	18	1	Complete	100	0	unclassified	unclassified
v23c2	25487	34	9	1	Medium-quality	54.02	0	Siphoviridae	prokaryote
v23c3	5633	7	2	0	Complete	100	0	unclassified	unclassified

v23c4	39137	53	9	0	Complete	100	0	unclassified	unclassified
v23c5	39728	60	14	0	Medium-quality	67.55	0	unclassified	unclassified
v23c6	25569	35	15	0	Medium-quality	64.28	0	Siphoviridae	prokaryote
v23c7	36505	49	33	0	High-quality	92.57	0	Siphoviridae	prokaryote
v23c8	41000	59	32	1	Complete	100	0	Siphoviridae	prokaryote
v23c9	47449	75	55	0	High-quality	97.72	0	Myoviridae	prokaryote
v23ca	13962	20	6	0	Medium-quality	73.26	0	Salasmaviridae	prokaryote
v23cb	41377	58	8	2	Medium-quality	83.55	0	unclassified	unclassified
v23cc	43288	74	18	3	High-quality	100	0	unclassified	unclassified
v23cd	49930	57	12	1	Complete	100	0	unclassified	unclassified
v23ce	35595	59	16	0	Complete	100	0	Siphoviridae	prokaryote
v23cf	24104	27	21	0	Medium-quality	59.58	0	Siphoviridae	prokaryote
v23d0	42926	58	19	0	Medium-quality	86.32	0	Siphoviridae	prokaryote
v23d1	16475	24	7	0	Medium-quality	76.13	0	unclassified	unclassified
v23d2	45868	63	16	0	Complete	100	0	Siphoviridae	prokaryote
v23d3	48826	68	14	2	Medium-quality	83.92	0	unclassified	unclassified
v23d4	44036	66	22	4	High-quality	100	0	Siphoviridae	prokaryote
v23d5	27345	37	7	0	Medium-quality	59.94	0	unclassified	unclassified
v23d6	23610	29	5	3	Medium-quality	82.3	0	unclassified	unclassified
v23d7	47372	46	9	2	Medium-quality	61.96	0	unclassified	unclassified
v23d8	84034	123	38	1	Complete	100	0	Siphoviridae	prokaryote
v23d9	5427	9	4	0	Complete	100	0	Microviridae	prokaryote
v23da	10655	14	4	0	Medium-quality	80.4	0	unclassified	unclassified
v23db	99265	171	36	6	High-quality	100	0	unclassified	unclassified
v23dc	29843	49	18	1	Medium-quality	72.31	0	Siphoviridae	prokaryote
v23dd	43792	76	17	0	Complete	100	0	unclassified	unclassified
v23de	5669	8	2	0	Complete	100	0	unclassified	unclassified
v23df	38423	53	21	4	Complete	100	0	Siphoviridae	prokaryote
v23e0	104790	115	21	2	Complete	100	0	Gratiaviridae	prokaryote
v23e1	24361	36	16	0	Medium-quality	74.1	0	Siphoviridae	prokaryote
v23e2	42315	60	26	1	High-quality	97.29	0	unclassified	unclassified
v23e3	46082	60	25	6	High-quality	96.45	0	Siphoviridae	prokaryote
v23e4	57679	60	8	5	High-quality	100	0	unclassified	unclassified
v23e5	41539	65	24	1	Complete	100	0	Siphoviridae	prokaryote
v23e6	14767	19	7	0	Medium-quality	79.21	0	Salasmaviridae	prokaryote
v23e7	35115	63	21	0	High-quality	100	0	Siphoviridae	prokaryote
v23e8	16230	18	8	0	Medium-quality	85.8	0	Salasmaviridae	prokaryote
v23e9	45257	75	18	0	High-quality	100	0	unclassified	unclassified
v23ea	100399	121	42	0	Medium-quality	73.42	0	unclassified	unclassified
v23eb	36043	48	15	0	High-quality	95.42	0	Siphoviridae	prokaryote

v23ec	71560	110	17	1	High-quality	100	0	unclassified	unclassified
v23ed	35355	61	15	0	High-quality	96.01	0	Siphoviridae	prokaryote
v23ee	92994	93	6	0	High-quality	100	0	Podoviridae_crAss-like	prokaryote
v23ef	35153	57	19	1	Complete	100	0	Siphoviridae	prokaryote
v23f0	38796	50	10	1	Medium-quality	63.09	0	unclassified	unclassified
v23f1	16497	19	9	0	Medium-quality	74.97	0	Guelinviridae	prokaryote
v23f2	37190	50	31	0	Complete	100	0	Siphoviridae	prokaryote
v23f3	41861	65	23	2	Complete	100	0	unclassified	unclassified
v23f4	35763	60	19	0	Complete	100	0	Siphoviridae	prokaryote
v23f5	45703	74	40	1	Complete	100	0	Siphoviridae	prokaryote
v23f6	14520	15	1	0	High-quality	100	0	unclassified	unclassified
v23f7	43793	70	18	0	Complete	100	0	unclassified	unclassified
v23f8	5393	9	7	0	High-quality	100	0	Microviridae	prokaryote
v23f9	51725	100	13	1	Medium-quality	65.25	0	unclassified	unclassified
v23fa	37503	56	21	0	High-quality	100	0	Siphoviridae	prokaryote
v23fb	34508	57	20	0	Complete	100	0	Siphoviridae	prokaryote
v23fc	51990	73	14	0	Medium-quality	76.01	0	unclassified	unclassified
v23fd	38167	60	8	1	High-quality	100	0	unclassified	unclassified
v23fe	46331	72	13	2	Medium-quality	79.75	0	unclassified	unclassified
v23ff	38402	67	33	0	Complete	100	0	Siphoviridae	prokaryote
v2400	13263	17	2	1	Complete	100	0	unclassified	unclassified
v2401	29032	47	10	5	Medium-quality	83.04	0	Siphoviridae	prokaryote
v2402	5977	9	2	0	Complete	100	0	unclassified	unclassified
v2403	15205	19	7	0	Medium-quality	81.35	0	Salasmaviridae	prokaryote
v2404	38205	56	19	1	Complete	100	0	Siphoviridae	prokaryote
v2405	26684	36	9	1	Medium-quality	59.19	0	unclassified	unclassified
v2406	52775	84	15	0	High-quality	99.39	0	unclassified	unclassified
v2407	65212	71	10	1	Complete	100	0	unclassified	unclassified
v2408	63221	88	21	2	High-quality	99.44	0	Myoviridae	prokaryote
v2409	102804	153	20	3	High-quality	100	0	Quimbyviridae	prokaryote
v240a	146315	228	33	3	Medium-quality	77.7	0	unclassified	unclassified
v240b	111971	145	22	8	High-quality	100	0	Gratiaviridae	prokaryote
v240c	16604	21	4	0	Medium-quality	53.39	0	unclassified	unclassified
v240d	24972	43	16	0	Medium-quality	72.94	0	Myoviridae	prokaryote
v240e	45485	58	20	2	High-quality	100	0	Siphoviridae	prokaryote
v240f	31113	48	26	0	Medium-quality	73.09	0	Myoviridae	prokaryote
v2410	34435	49	17	1	High-quality	100	0	Siphoviridae	prokaryote
v2411	61401	84	33	0	Complete	100	0	Siphoviridae	prokaryote
v2412	42464	71	12	0	Medium-quality	73.7	0	unclassified	unclassified
v2413	152993	195	16	2	Complete	100	0	unclassified	unclassified

v2414	30923	51	13	0	Medium-quality	64	0	unclassified	unclassified
v2415	96235	98	12	5	Complete	100	0	Podoviridae_crAss-like	prokaryote
v2416	50620	81	26	0	High-quality	96.75	0	unclassified	unclassified
v2417	41158	51	50	0	Complete	100	0	Autographiviridae	prokaryote
v2418	25294	34	20	0	Medium-quality	73.49	0	Siphoviridae	prokaryote
v2419	50686	87	18	1	High-quality	100	0	Myoviridae	prokaryote
v241a	22578	27	18	0	Medium-quality	51.56	0	Myoviridae	prokaryote
v241b	28214	42	10	2	Medium-quality	64.45	0	unclassified	unclassified
v241c	31324	52	14	0	Medium-quality	73.62	0	Siphoviridae	prokaryote
v241d	38383	63	19	1	High-quality	96.43	0	Siphoviridae	prokaryote
v241e	41818	74	20	1	Complete	100	0	Siphoviridae	prokaryote
v241f	44896	72	13	0	Complete	100	0	unclassified	unclassified
v2420	31580	40	7	3	Medium-quality	81.83	0	Siphoviridae	prokaryote
v2421	41623	77	18	2	High-quality	100	0	Siphoviridae	prokaryote
v2422	150616	225	31	3	Complete	100	0	unclassified	unclassified
v2423	39238	49	12	1	High-quality	94.76	0	unclassified	unclassified
v2424	5900	10	5	0	Complete	100	0	Microviridae	prokaryote
v2425	33758	59	20	4	Complete	100	0	Siphoviridae	prokaryote
v2426	49142	60	3	2	Medium-quality	86.8	0	unclassified	unclassified
v2427	46752	77	19	3	Complete	100	0	unclassified	unclassified
v2428	33431	51	15	0	Complete	100	0	unclassified	unclassified
v2429	19271	25	10	0	Medium-quality	50.96	0	unclassified	unclassified
v242a	46782	77	20	1	Complete	100	0	unclassified	unclassified
v242b	28749	39	10	0	Medium-quality	65.4	0	unclassified	unclassified
v242c	65104	110	28	0	High-quality	100	0	unclassified	unclassified
v242d	15282	20	7	0	Medium-quality	64.76	0	unclassified	unclassified
v242e	17168	22	8	0	Medium-quality	88.03	0	Salasmaviridae	prokaryote
v242f	6630	9	3	0	Complete	100	0	unclassified	unclassified
v2430	58119	76	9	2	Medium-quality	67.55	0	Quimbyviridae	prokaryote
v2431	28874	38	25	0	Medium-quality	63	0	Siphoviridae	prokaryote
v2432	38258	49	11	2	Medium-quality	74.79	0	unclassified	unclassified
v2433	42473	58	12	0	High-quality	94.08	0	Siphoviridae	prokaryote
v2434	39643	47	7	3	High-quality	98.28	0	unclassified	unclassified
v2435	72325	81	19	6	High-quality	95.57	0	Myoviridae	prokaryote
v2436	40073	60	9	3	Medium-quality	65.25	0	unclassified	unclassified
v2437	45590	74	18	0	Complete	100	0	unclassified	unclassified
v2438	35682	52	21	1	Complete	100	0	Siphoviridae	prokaryote
v2439	37241	66	15	0	Complete	100	0	unclassified	unclassified
v243a	155871	206	18	4	Complete	100	0	unclassified	unclassified
v243b	12191	13	3	2	High-quality	100	0	Microviridae	prokaryote

v243c	48292	61	5	4	Medium-quality	58.12	0	unclassified	unclassified
v243d	45579	81	23	1	Complete	100	0	unclassified	unclassified
v243e	57617	98	18	2	Complete	100	0	unclassified	unclassified
v243f	55864	99	12	2	Complete	100	0	unclassified	unclassified
v2440	61186	98	22	0	High-quality	100	0	unclassified	unclassified
v2441	42499	59	9	0	Complete	100	0	unclassified	unclassified
v2442	21949	24	22	1	Medium-quality	55.29	0	Siphoviridae	prokaryote
v2443	25251	40	9	0	Medium-quality	69.86	0	Siphoviridae	prokaryote
v2444	55676	64	11	3	Medium-quality	60.28	0	Siphoviridae	prokaryote
v2445	49185	52	23	2	High-quality	100	0	Siphoviridae	prokaryote
v2446	39081	56	14	0	Complete	100	0	unclassified	unclassified
v2447	36222	58	23	0	Complete	100	0	Siphoviridae	prokaryote
v2448	54381	58	8	1	Medium-quality	80.83	0	unclassified	unclassified
v2449	36890	49	24	2	High-quality	91.51	0	Siphoviridae	prokaryote
v244a	37813	54	19	1	Medium-quality	65.47	0	Myoviridae	prokaryote
v244b	42982	58	40	0	High-quality	93.56	0	Siphoviridae	prokaryote
v244c	39451	56	24	1	Complete	100	0	Siphoviridae	prokaryote
v244d	57128	78	56	0	Complete	100	0	Siphoviridae	prokaryote
v244e	65106	85	31	1	Complete	100	0	Siphoviridae	prokaryote
v244f	23913	25	15	1	Medium-quality	55.29	0	Siphoviridae	prokaryote
v2450	27242	42	8	2	Medium-quality	71.07	0	unclassified	unclassified
v2451	31341	39	24	4	Medium-quality	62.42	0	Siphoviridae	prokaryote
v2452	28113	43	8	1	Medium-quality	61.49	0	unclassified	unclassified
v2453	47002	66	15	2	High-quality	98.6	0	unclassified	unclassified
v2454	37248	59	16	0	Complete	100	0	unclassified	unclassified
v2455	29271	39	15	2	Medium-quality	72.6	0	Siphoviridae	prokaryote
v2456	5707	7	1	0	High-quality	100	0	unclassified	unclassified
v2457	39660	83	21	1	Complete	100	0	Siphoviridae	prokaryote
v2458	158500	225	31	8	High-quality	94.53	0	unclassified	unclassified
v2459	31125	46	22	2	Medium-quality	77.27	0	Siphoviridae	prokaryote
v245a	38160	63	19	1	Complete	100	0	Siphoviridae	prokaryote
v245b	76661	108	22	2	High-quality	100	0	unclassified	unclassified
v245c	68616	83	20	7	High-quality	100	0	unclassified	unclassified
v245d	38892	71	13	0	Complete	100	0	unclassified	unclassified
v245e	47087	77	24	0	Complete	100	0	unclassified	unclassified
v245f	41288	63	26	1	High-quality	91.47	0	Siphoviridae	prokaryote
v2460	13483	16	7	0	High-quality	98.83	0	unclassified	unclassified
v2461	43465	57	20	0	Complete	100	0	unclassified	unclassified
v2462	30645	42	7	3	High-quality	96.81	0	unclassified	unclassified
v2463	22326	28	22	1	Medium-quality	50.58	0	Myoviridae	prokaryote

v2464	39585	44	6	1	High-quality	93.37	0	unclassified	unclassified
v2465	186306	226	30	3	Complete	100	0	unclassified	unclassified
v2466	15731	25	8	0	Medium-quality	52.39	0	unclassified	unclassified
v2467	40224	53	30	0	Complete	100	0	Siphoviridae	prokaryote
v2468	42665	58	11	1	Complete	100	0	unclassified	unclassified
v2469	9022	10	1	0	High-quality	100	0	unclassified	unclassified
v246a	46959	54	25	1	High-quality	100	0	unclassified	unclassified
v246b	22946	28	8	2	Medium-quality	56.97	0	unclassified	unclassified
v246c	72527	114	17	0	High-quality	100	0	unclassified	unclassified
v246d	6554	8	3	0	High-quality	100	0	unclassified	unclassified
v246e	17118	25	6	0	Medium-quality	89.9	0	unclassified	unclassified
v246f	68241	80	11	9	Medium-quality	88.82	0	unclassified	unclassified
v2470	38214	55	30	0	Complete	100	0	Myoviridae	prokaryote
v2471	25024	34	26	0	Medium-quality	58.67	0	Myoviridae	prokaryote
v2472	42528	61	8	1	Medium-quality	50.86	0	Quimbyviridae	prokaryote
v2473	38659	64	21	2	Complete	100	0	Siphoviridae	prokaryote
v2474	62724	71	5	3	Medium-quality	86.57	0	unclassified	unclassified
v2475	40469	64	16	1	Complete	100	0	Siphoviridae	prokaryote
v2476	10372	4	1	0	High-quality	100	0	unclassified	unclassified
v2477	62662	93	31	1	High-quality	100	0	Siphoviridae	prokaryote
v2478	37127	59	22	1	High-quality	95.31	0	unclassified	unclassified
v2479	52600	56	10	2	High-quality	100	0	unclassified	unclassified
v247a	20243	30	13	0	Medium-quality	52.25	0	Siphoviridae	prokaryote
v247b	37949	62	16	3	Complete	100	0	unclassified	unclassified
v247c	13343	16	4	0	Complete	100	0	unclassified	unclassified
v247d	38899	61	12	0	Complete	100	0	unclassified	unclassified
v247e	67494	83	13	2	High-quality	100	0	unclassified	unclassified
v247f	35273	57	21	2	High-quality	100	0	Siphoviridae	prokaryote
v2480	54800	95	17	1	Complete	100	0	unclassified	unclassified
v2481	15785	24	6	0	Medium-quality	67.58	0	unclassified	unclassified
v2482	104918	160	32	2	High-quality	100	0	unclassified	unclassified
v2483	73282	118	11	2	Medium-quality	62.44	0	unclassified	unclassified
v2484	11725	14	5	0	High-quality	98.92	0	Podoviridae	prokaryote
v2485	19685	29	18	0	Medium-quality	55.55	0	Myoviridae	prokaryote
v2486	47856	68	18	0	High-quality	100	0	unclassified	unclassified
v2487	44933	80	22	4	Complete	100	0	unclassified	unclassified
v2488	43594	73	16	1	High-quality	100	0	unclassified	unclassified
v2489	22811	31	15	0	Medium-quality	59.33	0	Siphoviridae	prokaryote
v248a	33522	42	19	0	Medium-quality	74.36	0	Siphoviridae	prokaryote
v248b	63097	67	11	1	High-quality	99.29	0	unclassified	unclassified

v248c	85876	135	15	4	Medium-quality	51.15	0	unclassified	unclassified
v248d	58701	106	21	0	Complete	100	0	unclassified	unclassified
v248e	43621	57	30	2	High-quality	100	0	Siphoviridae	prokaryote
v248f	40479	54	21	1	Complete	100	0	Siphoviridae	prokaryote
v2490	29861	40	20	0	Medium-quality	86.68	0	Siphoviridae	prokaryote
v2491	41394	61	12	2	Medium-quality	73.74	0	unclassified	unclassified
v2492	41074	76	16	1	Complete	100	0	unclassified	unclassified
v2493	38796	65	14	3	Complete	100	0	unclassified	unclassified
v2494	46456	75	12	1	Complete	100	0	unclassified	unclassified
v2495	48132	72	10	1	High-quality	100	0	unclassified	unclassified
v2496	23167	33	16	0	Medium-quality	64.01	0	unclassified	unclassified
v2497	43346	68	19	1	Complete	100	0	unclassified	unclassified
v2498	32643	41	6	1	Medium-quality	59.84	0	unclassified	unclassified
v2499	39749	54	24	0	Medium-quality	79.82	0	Myoviridae	prokaryote
v249a	45892	58	18	3	Medium-quality	50.89	0	Myoviridae	prokaryote
v249b	59221	80	16	7	High-quality	100	0	unclassified	unclassified
v249c	101402	163	15	1	Complete	100	0	Podoviridae_crAss-like	prokaryote
v249d	28273	41	26	1	Medium-quality	61.9	0	Myoviridae	prokaryote
v249e	30699	49	7	0	Complete	100	0	unclassified	unclassified
v249f	41485	64	25	2	Complete	100	0	Siphoviridae	prokaryote
v24a0	8035	9	1	0	High-quality	100	0	unclassified	unclassified
v24a1	25113	26	4	2	Medium-quality	51.53	0	Siphoviridae	prokaryote
v24a2	37245	38	13	7	Medium-quality	69.69	0	Siphoviridae	prokaryote
v24a3	12697	14	4	0	High-quality	100	0	Podoviridae	prokaryote
v24a4	42710	67	15	2	Complete	100	0	Myoviridae	prokaryote
v24a5	65870	84	20	3	Complete	100	0	Siphoviridae	prokaryote
v24a6	47550	55	18	3	High-quality	100	0	Siphoviridae	prokaryote
v24a7	36419	59	36	0	Complete	100	0	Siphoviridae	prokaryote
v24a8	33763	45	23	0	High-quality	90.95	0	Siphoviridae	prokaryote
v24a9	16280	22	7	0	Medium-quality	72.05	0	unclassified	unclassified
v24aa	26894	38	20	0	Medium-quality	57.35	0	Myoviridae	prokaryote
v24ab	77804	90	15	2	High-quality	100	0	unclassified	unclassified
v24ac	45866	62	29	1	High-quality	100	0	Myoviridae	prokaryote
v24ad	51820	66	16	1	High-quality	96.56	0	unclassified	unclassified
v24ae	46441	71	24	2	Complete	100	0	unclassified	unclassified
v24af	44484	81	17	0	Complete	100	0	unclassified	unclassified
v24b0	106084	135	21	3	Complete	100	0	Gratiaviridae	prokaryote
v24b1	88366	117	16	2	Complete	100	0	Flandersviridae	prokaryote
v24b2	79310	140	22	2	Medium-quality	50.85	0	unclassified	unclassified
v24b3	41854	55	28	0	High-quality	96.12	0	Siphoviridae	prokaryote

v24b4	22439	44	12	0	Medium-quality	58.89	0	Siphoviridae	prokaryote
v24b5	36149	51	17	0	High-quality	100	0	Siphoviridae	prokaryote
v24b6	52930	64	24	2	High-quality	100	0	Siphoviridae	prokaryote
v24b7	148800	216	36	4	Complete	100	0	unclassified	unclassified
v24b8	65548	96	32	2	High-quality	100	0	Siphoviridae	prokaryote
v24b9	24506	34	2	0	Medium-quality	66.85	0	unclassified	unclassified
v24ba	36453	56	23	0	High-quality	97.2	0	Siphoviridae	prokaryote
v24bb	44624	59	4	2	High-quality	100	0	unclassified	unclassified
v24bc	21962	34	4	2	Medium-quality	51.52	0	unclassified	unclassified
v24bd	18405	28	12	0	Medium-quality	53.93	0	unclassified	unclassified
v24be	41833	59	11	2	High-quality	94.65	0	unclassified	unclassified
v24bf	92625	119	32	2	High-quality	100	0	Myoviridae	prokaryote
v24c0	23368	31	10	0	Medium-quality	60.3	0	Quimbyviridae	prokaryote
v24c1	63477	112	17	4	High-quality	100	0	unclassified	unclassified
v24c2	34016	50	16	1	Medium-quality	84.22	0	Siphoviridae	prokaryote
v24c3	26589	34	11	0	Medium-quality	60.49	0	unclassified	unclassified
v24c4	39083	68	20	2	Complete	100	0	unclassified	unclassified
v24c5	38498	68	33	0	Medium-quality	55.75	0	Myoviridae	prokaryote
v24c6	49477	78	30	0	High-quality	100	0	Siphoviridae	prokaryote
v24c7	24410	37	17	0	Low-quality	49.88	0	Myoviridae	prokaryote
v24c8	45166	63	22	0	Complete	100	0	Siphoviridae	prokaryote
v24c9	22986	27	5	2	Medium-quality	65.98	0	unclassified	unclassified
v24ca	64793	76	13	2	High-quality	100	0	unclassified	unclassified
v24cb	34829	49	14	2	Medium-quality	65.94	0	unclassified	unclassified
v24cc	5818	9	1	0	Complete	100	0	unclassified	unclassified
v24cd	5457	9	6	0	Complete	100	0	Microviridae	prokaryote
v24ce	238993	227	35	3	High-quality	100	0	unclassified	unclassified
v24cf	40216	46	17	0	High-quality	100	0	unclassified	unclassified
v24d0	55061	89	21	0	Complete	100	0	unclassified	unclassified
v24d1	26954	62	25	0	Medium-quality	50.11	0	Siphoviridae	prokaryote
v24d2	57005	64	5	2	High-quality	97.01	0	unclassified	unclassified
v24d3	59117	86	33	1	High-quality	96.23	0	Siphoviridae	prokaryote
v24d4	11779	13	4	0	Medium-quality	85.13	0	unclassified	unclassified
v24d5	5497	11	4	0	Complete	100	0	Microviridae	prokaryote
v24d6	31717	51	10	5	High-quality	91.04	0	Siphoviridae	prokaryote
v24d7	239231	260	95	2	Complete	100	0	Myoviridae	prokaryote
v24d8	64772	78	7	3	Medium-quality	73.5	0	Quimbyviridae	prokaryote
v24d9	35465	50	8	1	Medium-quality	62.61	0	unclassified	unclassified
v24da	38667	48	12	2	High-quality	95.24	0	unclassified	unclassified
v24db	38120	63	17	4	High-quality	100	0	unclassified	unclassified

v24dc	31858	57	8	3	Medium-quality	53.01	0	Siphoviridae	prokaryote
v24dd	52070	57	3	2	High-quality	100	0	unclassified	unclassified
v24de	34928	40	20	0	Medium-quality	56.9	0	Siphoviridae	prokaryote
v24df	20429	29	15	0	Medium-quality	51.08	0	Siphoviridae	prokaryote
v24e0	25380	45	6	2	Medium-quality	67.17	0	unclassified	unclassified
v24e1	14629	19	11	0	Medium-quality	63.14	0	Salasmaviridae	prokaryote
v24e2	80751	141	24	2	Complete	100	0	unclassified	unclassified
v24e3	36161	43	14	0	Medium-quality	81.61	0	unclassified	unclassified
v24e4	38964	69	20	3	High-quality	99.99	0	unclassified	unclassified
v24e5	38107	44	18	1	High-quality	92.63	0	Siphoviridae	prokaryote
v24e6	24824	35	11	0	Medium-quality	66.64	0	unclassified	unclassified
v24e7	27720	50	18	0	Medium-quality	56.77	0	Myoviridae	prokaryote
v24e8	146511	240	34	2	Medium-quality	89.41	0	unclassified	unclassified
v24e9	20874	24	19	0	Medium-quality	53.04	0	Podoviridae	prokaryote
v24ea	114428	151	39	5	High-quality	100	0	Siphoviridae	prokaryote
v24eb	26515	33	22	0	Medium-quality	57.13	0	Siphoviridae	prokaryote
v24ec	38469	43	15	0	Complete	100	0	Siphoviridae	prokaryote
v24ed	6431	8	3	0	Complete	100	0	Microviridae	prokaryote
v24ee	40645	76	18	1	High-quality	99.35	0	unclassified	unclassified
v24ef	13486	15	1	0	High-quality	100	0	Myoviridae	prokaryote
v24f0	36610	46	14	3	High-quality	95.7	0	unclassified	unclassified
v24f1	38550	56	11	1	Medium-quality	63.04	0	unclassified	unclassified
v24f2	57054	84	17	4	High-quality	100	0	Myoviridae	prokaryote
v24f3	27594	36	27	0	Medium-quality	68.01	0	Podoviridae	prokaryote
v24f4	20911	30	7	1	Medium-quality	52.17	0	Podoviridae	prokaryote
v24f5	21145	26	12	0	Medium-quality	56.58	0	Siphoviridae	prokaryote
v24f6	83612	123	25	1	High-quality	98.46	0	unclassified	unclassified
v24f7	24157	28	10	0	Medium-quality	57.7	0	unclassified	unclassified
v24f8	21656	15	4	3	Medium-quality	75.49	0	Siphoviridae	prokaryote
v24f9	61183	89	15	0	Complete	100	0	unclassified	unclassified
v24fa	63454	97	20	0	High-quality	100	0	Myoviridae	prokaryote
v24fb	33506	48	2	1	Medium-quality	87.83	0	unclassified	unclassified
v24fc	38971	52	9	1	Medium-quality	65.22	0	unclassified	unclassified
v24fd	21087	22	19	0	Medium-quality	52.53	0	Podoviridae	prokaryote
v24fe	11194	12	3	2	High-quality	100	0	unclassified	unclassified
v24ff	49391	73	33	0	High-quality	100	0	unclassified	unclassified
v2500	47604	66	25	1	Complete	100	0	Siphoviridae	prokaryote
v2501	23346	29	16	0	Medium-quality	64.36	0	Siphoviridae	prokaryote
v2502	24243	32	22	0	Medium-quality	59.7	0	Siphoviridae	prokaryote
v2503	47538	62	6	2	Medium-quality	87.75	0	unclassified	unclassified

v2504	46834	58	10	1	High-quality	100	0	Siphoviridae	prokaryote
v2505	48151	59	16	3	High-quality	100	0	Myoviridae	prokaryote
v2506	5264	7	4	0	High-quality	91.51	0	Microviridae	prokaryote
v2507	29303	34	23	0	Medium-quality	73.03	0	Podoviridae	prokaryote
v2508	36524	55	17	0	Complete	100	0	Siphoviridae	prokaryote
v2509	144397	219	36	6	Medium-quality	86.48	0	unclassified	unclassified
v250a	16827	21	7	0	Medium-quality	75.8	0	unclassified	unclassified
v250b	37867	56	24	0	Complete	100	0	Siphoviridae	prokaryote
v250c	34205	54	16	0	Medium-quality	88.41	0	unclassified	unclassified
v250d	37054	56	4	3	Medium-quality	56.34	0	unclassified	unclassified
v250e	11986	14	5	0	Complete	100	0	Podoviridae	prokaryote
v250f	39653	55	8	1	Medium-quality	84.29	0	unclassified	unclassified
v2510	37439	52	40	0	Complete	100	0	Podoviridae	prokaryote
v2511	30661	50	12	0	Medium-quality	60.29	0	unclassified	unclassified
v2512	80588	129	25	10	Medium-quality	55.73	0	unclassified	unclassified
v2513	41819	59	7	1	Complete	100	0	unclassified	unclassified
v2514	41108	53	26	0	Medium-quality	89.39	0	unclassified	unclassified
v2515	63147	106	21	3	Complete	100	0	unclassified	unclassified
v2516	102788	176	19	3	Complete	100	0	Podoviridae_crAss-like	prokaryote
v2517	27102	37	25	0	Medium-quality	59.21	0	Siphoviridae	prokaryote
v2518	16462	24	7	0	Medium-quality	86.7	0	unclassified	unclassified
v2519	40902	68	13	0	High-quality	92.6	0	unclassified	unclassified
v251a	41214	43	10	0	High-quality	99.79	0	unclassified	unclassified
v251b	27939	34	25	1	Medium-quality	64.5	0	Myoviridae	prokaryote
v251c	15668	24	11	1	High-quality	100	0	Siphoviridae	prokaryote
v251d	102895	164	17	2	Complete	100	0	Podoviridae_crAss-like	prokaryote
v251e	31624	52	15	0	Medium-quality	82.62	0	unclassified	unclassified
v251f	41208	56	26	1	Medium-quality	86.86	0	Myoviridae	prokaryote
v2520	43267	58	27	0	High-quality	100	0	Myoviridae	prokaryote
v2521	39671	57	14	2	High-quality	100	0	Siphoviridae	prokaryote
v2522	40983	60	29	2	High-quality	90.54	0	Myoviridae	prokaryote
v2523	79682	134	20	3	Complete	100	0	unclassified	unclassified
v2524	35787	61	28	1	Medium-quality	82.42	0	Myoviridae	prokaryote
v2525	53771	76	11	4	Medium-quality	89.02	0	unclassified	unclassified
v2526	26955	30	11	2	Medium-quality	58.25	0	Siphoviridae	prokaryote
v2527	40172	68	50	0	High-quality	95.74	0	Siphoviridae	prokaryote
v2528	41551	42	5	1	High-quality	100	0	Siphoviridae	prokaryote
v2529	36530	58	17	0	Medium-quality	86.31	0	unclassified	unclassified
v252a	38300	64	11	2	Medium-quality	81.8	0	unclassified	unclassified
v252b	54646	95	18	1	Complete	100	0	unclassified	unclassified

v252c	74048	107	36	0	Complete	100	0	unclassified	unclassified
v252d	42283	67	16	3	Complete	100	0	unclassified	unclassified
v252e	146048	180	18	3	High-quality	94.93	0	unclassified	unclassified
v252f	36570	68	21	0	High-quality	98.49	0	Siphoviridae	prokaryote
v2530	24393	36	11	0	Medium-quality	67.16	0	unclassified	unclassified
v2531	60384	82	18	1	Complete	100	0	Siphoviridae	prokaryote
v2532	31163	43	6	0	Medium-quality	72.42	0	unclassified	unclassified
v2533	85337	126	49	1	High-quality	100	0	unclassified	unclassified
v2534	46118	60	17	0	Complete	100	0	Siphoviridae	prokaryote
v2535	38824	60	20	1	Medium-quality	56.29	0	Siphoviridae	prokaryote
v2536	26355	47	5	0	Medium-quality	61.81	0	unclassified	unclassified
v2537	64485	75	12	3	High-quality	99.26	0	unclassified	unclassified
v2538	43806	66	16	1	High-quality	100	0	unclassified	unclassified
v2539	5613	8	2	0	Complete	100	0	unclassified	unclassified
v253a	17922	39	6	2	Medium-quality	52.92	0	Siphoviridae	prokaryote
v253b	25313	35	21	0	Medium-quality	56.39	0	Myoviridae	prokaryote
v253c	25780	28	13	2	Medium-quality	52	0	Siphoviridae	prokaryote
v253d	36600	54	12	2	Complete	100	0	unclassified	unclassified
v253e	44423	53	4	3	High-quality	100	0	unclassified	unclassified
v253f	41844	66	18	1	Medium-quality	88.91	0	Myoviridae	prokaryote
v2540	19566	31	11	1	Medium-quality	53.77	0	Siphoviridae	prokaryote
v2541	43504	74	18	0	High-quality	97.56	0	unclassified	unclassified
v2542	91152	95	9	2	High-quality	100	0	Podoviridae_crAss-like	prokaryote
v2543	44808	75	16	0	Complete	100	0	unclassified	unclassified
v2544	48283	77	25	1	High-quality	100	0	Myoviridae	prokaryote
v2545	74828	122	15	5	Complete	100	0	unclassified	unclassified
v2546	41260	53	14	2	High-quality	93.28	0	unclassified	unclassified
v2547	33604	48	17	0	High-quality	98.41	0	unclassified	unclassified
v2548	51388	73	21	2	High-quality	100	0	Myoviridae	prokaryote
v2549	38107	52	32	1	Medium-quality	66.34	0	Podoviridae	prokaryote
v254a	25332	37	11	0	Medium-quality	64.93	0	unclassified	unclassified
v254b	43362	59	29	2	High-quality	100	0	Siphoviridae	prokaryote
v254c	29949	37	13	0	Medium-quality	59.9	0	unclassified	unclassified
v254d	41895	79	17	0	Complete	100	0	unclassified	unclassified
v254e	31561	41	17	0	Medium-quality	60.26	0	Myoviridae	prokaryote
v254f	21565	37	10	1	Medium-quality	62.79	0	Siphoviridae	prokaryote
v2550	150457	223	32	4	Medium-quality	76.58	0	unclassified	unclassified
v2551	34666	48	5	3	Medium-quality	64.9	0	unclassified	unclassified
v2552	45414	86	18	0	Complete	100	0	unclassified	unclassified
v2553	39159	67	20	1	Medium-quality	84.48	0	Siphoviridae	prokaryote

v2554	39729	57	22	1	High-quality	94.83	0	Siphoviridae	prokaryote
v2555	66109	107	22	3	Medium-quality	81.98	0	unclassified	unclassified
v2556	16429	23	9	0	Medium-quality	86.33	0	unclassified	unclassified
v2557	34995	54	12	0	High-quality	100	0	Siphoviridae	prokaryote
v2558	6014	6	4	0	High-quality	98.56	0	unclassified	unclassified
v2559	63568	97	15	4	Medium-quality	82.93	0	unclassified	unclassified
v255a	23020	29	25	0	Medium-quality	59.59	0	Siphoviridae	prokaryote
v255b	31466	45	25	0	Medium-quality	73.89	0	Myoviridae	prokaryote
v255c	18131	30	20	0	Medium-quality	63.81	0	Siphoviridae	prokaryote
v255d	24680	28	2	1	High-quality	100	0	unclassified	unclassified
v255e	95491	105	9	0	Complete	100	0	Podoviridae_crAss-like	prokaryote
v255f	13031	20	2	1	Medium-quality	84.97	0	unclassified	unclassified
v2560	35770	51	15	2	High-quality	95.98	0	unclassified	unclassified
v2561	8191	11	1	0	High-quality	100	0	unclassified	unclassified
v2562	63875	90	20	1	Medium-quality	80.58	0	unclassified	unclassified
v2563	33337	51	14	1	Complete	100	0	Siphoviridae	prokaryote
v2564	41655	62	14	1	Medium-quality	65.95	0	unclassified	unclassified
v2565	50143	85	22	1	High-quality	100	0	Siphoviridae	prokaryote
v2566	30752	30	9	1	Medium-quality	51.62	0	Siphoviridae	prokaryote
v2567	39489	51	7	1	Medium-quality	69.17	0	unclassified	unclassified
v2568	39627	45	16	1	Medium-quality	80.35	0	Siphoviridae	prokaryote
v2569	41285	64	28	0	High-quality	91.1	0	Myoviridae	prokaryote
v256a	45456	73	16	4	Medium-quality	56.37	0	unclassified	unclassified
v256b	14571	23	7	0	Medium-quality	86.84	0	Siphoviridae	prokaryote
v256c	50497	65	5	3	High-quality	100	0	Siphoviridae	prokaryote
v256d	30966	28	14	0	Medium-quality	74.33	0	Podoviridae	prokaryote
v256e	46588	78	19	0	High-quality	100	0	Siphoviridae	prokaryote
v256f	24570	38	26	0	Medium-quality	53.8	0	Siphoviridae	prokaryote
v2570	56125	78	38	0	High-quality	99.53	0	Siphoviridae	prokaryote
v2571	48994	79	37	1	High-quality	100	0	Myoviridae	prokaryote
v2572	6754	9	3	0	Complete	100	0	unclassified	unclassified
v2573	6080	7	3	0	Complete	100	0	unclassified	unclassified
v2574	94264	142	29	1	Complete	100	0	unclassified	unclassified
v2575	6321	8	2	0	High-quality	100	0	unclassified	unclassified
v2576	14730	8	3	0	High-quality	100	0	Retroviridae	eukaryote
v2577	6410	8	3	0	High-quality	97.91	0	unclassified	unclassified
v2578	57755	78	16	2	High-quality	95.65	0	unclassified	unclassified
v2579	74867	119	12	2	Complete	100	0	Quimbyviridae	prokaryote
v257a	111119	170	24	2	Medium-quality	82.02	0	unclassified	unclassified
v257b	38576	56	45	0	Complete	100	0	Podoviridae	prokaryote

v257c	16333	23	6	0	Medium-quality	72.48	0	unclassified	unclassified
v257d	61423	99	23	1	High-quality	94.62	0	unclassified	unclassified
v257e	99115	104	23	0	High-quality	98.07	0	Podoviridae_crAss-like	prokaryote
v257f	37562	58	30	0	Complete	100	0	Siphoviridae	prokaryote
v2580	44782	71	18	1	Complete	100	0	unclassified	unclassified
v2581	13261	19	8	0	Medium-quality	56.64	0	Salasmaviridae	prokaryote
v2582	5540	8	3	0	Complete	100	0	unclassified	unclassified
v2583	5721	10	3	0	High-quality	96.71	0	unclassified	unclassified
v2584	44820	58	8	1	Complete	100	0	Siphoviridae	prokaryote
v2585	112269	135	34	1	Medium-quality	82.77	0	Siphoviridae	prokaryote
v2586	53959	98	21	0	High-quality	100	0	unclassified	unclassified
v2587	14065	21	5	0	High-quality	100	0	unclassified	unclassified
v2588	97679	117	25	4	Medium-quality	89.96	0	Gratiaviridae	prokaryote
v2589	193424	327	41	8	Complete	100	0	unclassified	unclassified
v258a	6131	8	3	0	Complete	100	0	unclassified	unclassified
v258b	6567	9	4	0	Complete	100	0	Microviridae	prokaryote
v258c	6195	7	4	0	Complete	100	0	unclassified	unclassified
v258d	6633	9	3	0	Complete	100	0	unclassified	unclassified
v258e	5604	7	5	0	High-quality	100	0	Microviridae	prokaryote
v258f	5673	7	1	0	Complete	100	0	unclassified	unclassified
v2590	6228	8	2	0	Complete	100	0	unclassified	unclassified
v2591	5623	5	1	0	Complete	100	0	unclassified	unclassified
v2592	6615	7	4	0	High-quality	100	0	unclassified	unclassified
v2593	46953	52	18	0	Medium-quality	52.79	0	unclassified	unclassified
v2594	13047	12	3	0	High-quality	100	0	Retroviridae	eukaryote
v2595	7914	9	4	0	High-quality	100	0	Microviridae	prokaryote
v2596	6498	7	3	0	Complete	100	0	Microviridae	prokaryote
v2597	44132	80	14	2	Complete	100	0	unclassified	unclassified
v2598	36223	62	18	1	Medium-quality	89.2	0	unclassified	unclassified
v2599	55938	97	15	2	Complete	100	0	unclassified	unclassified
v259a	91947	161	30	2	Complete	100	0	unclassified	unclassified
v259b	16387	22	6	0	Medium-quality	75.09	0	unclassified	unclassified
v259c	86049	115	13	2	Complete	100	0	Flandersviridae	prokaryote
v259d	29630	39	16	0	Medium-quality	77.92	0	Siphoviridae	prokaryote
v259e	40418	69	18	2	Complete	100	0	unclassified	unclassified
v259f	36104	61	16	3	High-quality	96.04	0	Siphoviridae	prokaryote
v25a0	36288	53	18	1	Medium-quality	86.59	0	Siphoviridae	prokaryote
v25a1	6610	9	3	0	Complete	100	0	unclassified	unclassified
v25a2	33001	44	7	1	Medium-quality	87.34	0	unclassified	unclassified
v25a3	43556	57	12	0	Complete	100	0	Siphoviridae	prokaryote

v25a4	7648	12	3	0	Medium-quality	59.55	0	unclassified	unclassified
v25a5	45349	74	28	0	Complete	100	0	Myoviridae	prokaryote
v25a6	30807	39	23	0	Medium-quality	51.03	0	Siphoviridae	prokaryote
v25a7	31913	43	19	1	Medium-quality	78.43	0	Siphoviridae	prokaryote
v25a8	32683	37	24	0	Medium-quality	54.24	0	Siphoviridae	prokaryote
v25a9	99014	160	29	1	High-quality	99.74	0	Podoviridae_crAss-like	prokaryote
v25aa	6714	9	3	0	Complete	100	0	Microviridae	prokaryote
v25ab	99724	95	11	0	Complete	100	0	Podoviridae_crAss-like	prokaryote
v25ac	6313	9	2	0	Complete	100	0	unclassified	unclassified
v25ad	48458	82	12	0	Medium-quality	85.15	0	unclassified	unclassified
v25ae	6501	9	3	0	Complete	100	0	Microviridae	prokaryote
v25af	40458	62	27	0	High-quality	93.44	0	unclassified	unclassified
v25b0	38169	49	17	0	Medium-quality	86.83	0	Siphoviridae	prokaryote
v25b1	97390	89	9	0	High-quality	100	0	Podoviridae_crAss-like	prokaryote
v25b2	44688	74	28	1	Complete	100	0	unclassified	unclassified
v25b3	5237	8	7	0	Complete	100	0	Microviridae	prokaryote
v25b4	40249	60	17	0	Medium-quality	88.75	0	unclassified	unclassified
v25b5	42729	71	20	0	High-quality	99.91	0	unclassified	unclassified
v25b6	14789	19	4	0	High-quality	100	0	unclassified	unclassified
v25b7	57181	97	15	1	Complete	100	0	unclassified	unclassified
v25b8	5839	9	2	0	Complete	100	0	unclassified	unclassified
v25b9	37775	44	22	2	High-quality	100	0	Siphoviridae	prokaryote
v25ba	44298	82	15	2	Complete	100	0	unclassified	unclassified
v25bb	57901	107	25	1	Complete	100	0	unclassified	unclassified
v25bc	21573	30	12	0	Medium-quality	64.36	0	Myoviridae	prokaryote
v25bd	46248	75	16	0	Complete	100	0	unclassified	unclassified
v25be	38536	66	19	0	Medium-quality	89.92	0	Siphoviridae	prokaryote
v25bf	16698	24	8	0	Medium-quality	74.11	0	unclassified	unclassified
v25c0	165611	186	19	0	Complete	100	0	unclassified	unclassified
v25c1	69002	81	18	0	High-quality	100	0	unclassified	unclassified
v25c2	37380	59	15	0	Complete	100	0	Siphoviridae	prokaryote
v25c3	34822	50	16	0	Complete	100	0	Siphoviridae	prokaryote
v25c4	62024	116	21	0	Complete	100	0	unclassified	unclassified
v25c5	36963	60	11	2	High-quality	96.25	0	unclassified	unclassified
v25c6	41566	74	34	0	High-quality	100	0	Siphoviridae	prokaryote
v25c7	6228	10	2	0	Complete	100	0	unclassified	unclassified
v25c8	10425	14	4	0	Medium-quality	88.82	0	Podoviridae	prokaryote
v25c9	94470	94	8	0	Complete	100	0	Podoviridae_crAss-like	prokaryote
v25ca	15109	22	1	0	Complete	100	0	unclassified	unclassified
v25cb	49890	61	5	2	Medium-quality	57.26	0	Quimbyviridae	prokaryote

v25cc	40045	67	23	1	Medium-quality	71.9	0	Siphoviridae	prokaryote
v25cd	76750	131	19	6	Complete	100	0	unclassified	unclassified
v25ce	8191	9	4	0	High-quality	100	0	unclassified	unclassified
v25cf	6027	9	2	0	Complete	100	0	unclassified	unclassified
v25d0	5760	7	3	0	High-quality	94.42	0	unclassified	unclassified
v25d1	36207	50	23	1	Complete	100	0	Siphoviridae	prokaryote
v25d2	85169	108	25	0	Medium-quality	51.39	0	unclassified	unclassified
v25d3	37469	61	29	0	Complete	100	0	Siphoviridae	prokaryote
v25d4	17534	29	6	0	High-quality	90.62	0	Podoviridae	prokaryote
v25d5	50204	93	20	0	Complete	100	0	unclassified	unclassified
v25d6	12509	21	5	0	High-quality	91.68	0	unclassified	unclassified
v25d7	7578	10	4	0	High-quality	100	0	unclassified	unclassified
v25d8	6140	9	3	0	Complete	100	0	unclassified	unclassified
v25d9	42403	59	29	0	High-quality	100	0	Siphoviridae	prokaryote
v25da	29938	43	5	0	Medium-quality	54.31	0	unclassified	unclassified
v25db	39002	51	24	0	Medium-quality	64.77	0	Siphoviridae	prokaryote
v25dc	44112	82	14	0	Complete	100	0	unclassified	unclassified
v25dd	6657	7	3	0	Complete	100	0	unclassified	unclassified
v25de	87651	110	4	2	Medium-quality	79.17	0	unclassified	unclassified
v25df	70786	107	14	4	Complete	100	0	unclassified	unclassified
v25e0	32527	56	11	2	High-quality	98.05	0	unclassified	unclassified
v25e1	51141	85	22	0	High-quality	100	0	unclassified	unclassified
v25e2	55809	105	19	0	Complete	100	0	unclassified	unclassified
v25e3	41861	80	21	1	Complete	100	0	unclassified	unclassified
v25e4	6475	7	3	0	Complete	100	0	Microviridae	prokaryote
v25e5	34806	55	19	2	Medium-quality	79.52	0	unclassified	unclassified
v25e6	38317	74	15	1	High-quality	92.9	0	unclassified	unclassified
v25e7	53986	98	15	0	Complete	100	0	unclassified	unclassified
v25e8	58956	98	22	2	Complete	100	0	unclassified	unclassified
v25e9	40182	77	15	2	High-quality	98.05	0	unclassified	unclassified
v25ea	10504	15	2	0	Complete	100	0	unclassified	unclassified
v25eb	13994	26	5	0	High-quality	100	0	unclassified	unclassified
v25ec	16834	21	6	0	Medium-quality	56.66	0	unclassified	unclassified
v25ed	6152	10	4	0	Complete	100	0	unclassified	unclassified
v25ee	37799	55	11	0	High-quality	95.68	0	unclassified	unclassified
v25ef	5425	8	3	0	Complete	100	0	unclassified	unclassified
v25f0	41493	77	18	1	High-quality	95.67	0	unclassified	unclassified
v25f1	52709	91	17	1	High-quality	100	0	unclassified	unclassified
v25f2	41254	68	51	0	High-quality	98.46	0	Siphoviridae	prokaryote
v25f3	85840	155	22	2	Complete	100	0	Quimbyviridae	prokaryote

v25f4	6321	7	3	0	Complete	100	0	unclassified	unclassified
v25f5	5758	9	6	0	Complete	100	0	Microviridae	prokaryote
v25f6	41153	68	17	1	High-quality	92.13	0	unclassified	unclassified
v25f7	43066	72	23	0	Complete	100	0	unclassified	unclassified
v25f8	16260	22	7	0	Medium-quality	83.23	0	unclassified	unclassified
v25f9	51826	83	21	1	Complete	100	0	unclassified	unclassified
v25fa	33733	38	7	4	Medium-quality	75.5	0	unclassified	unclassified
v25fb	6593	9	3	0	Complete	100	0	unclassified	unclassified
v25fc	16847	17	8	0	High-quality	100	0	unclassified	unclassified
v25fd	58514	101	20	0	Complete	100	0	unclassified	unclassified
v25fe	41022	71	18	1	Complete	100	0	unclassified	unclassified
v25ff	60933	113	23	0	Complete	100	0	unclassified	unclassified
v2600	6014	8	2	0	High-quality	100	0	unclassified	unclassified
v2601	19455	31	8	0	Complete	100	0	Guelinviridae	prokaryote
v2602	39965	60	7	2	Medium-quality	55.09	0	unclassified	unclassified
v2603	38860	61	31	1	Complete	100	0	Siphoviridae	prokaryote
v2604	31139	49	19	0	Medium-quality	85.67	0	unclassified	unclassified
v2605	5141	8	6	0	Complete	100	0	Microviridae	prokaryote
v2606	36355	58	11	1	High-quality	100	0	unclassified	unclassified
v2607	27761	48	10	0	Medium-quality	62.8	0	unclassified	unclassified
v2608	6671	4	3	0	Medium-quality	79.45	0	Retroviridae	eukaryote
v2609	47406	65	18	0	Complete	100	0	Siphoviridae	prokaryote
v260a	53595	80	23	0	High-quality	95.58	0	unclassified	unclassified
v260b	35072	51	15	0	Medium-quality	57.57	0	Siphoviridae	prokaryote
v260c	45169	73	11	0	High-quality	100	0	unclassified	unclassified
v260d	15351	20	8	0	Medium-quality	79.9	0	unclassified	unclassified
v260e	42157	56	9	0	High-quality	100	0	unclassified	unclassified
v260f	5143	9	6	0	Complete	100	0	Microviridae	prokaryote
v2610	5398	8	4	0	Complete	100	0	Microviridae	prokaryote
v2611	24394	32	15	0	Medium-quality	50.05	0	Siphoviridae	prokaryote
v2612	67482	112	17	0	Medium-quality	68.61	0	Podoviridae_crAss-like	prokaryote
v2613	25862	45	12	0	Medium-quality	66.88	0	unclassified	unclassified
v2614	36842	42	6	0	High-quality	90.34	0	unclassified	unclassified
v2615	16158	19	4	0	Medium-quality	56.13	0	unclassified	unclassified
v2616	5209	8	7	0	Complete	100	0	Microviridae	prokaryote
v2617	40760	48	5	4	Medium-quality	67.47	0	unclassified	unclassified
v2618	15776	20	7	0	Medium-quality	74.68	0	Salasmaviridae	prokaryote
v2619	13804	18	6	0	High-quality	99.39	0	unclassified	unclassified
v261a	15854	18	6	0	Medium-quality	82.95	0	Salasmaviridae	prokaryote
v261b	6442	10	4	0	Complete	100	0	unclassified	unclassified

v261c	6148	9	2	0	Complete	100	0	unclassified	unclassified
v261d	43786	74	16	1	High-quality	99.19	0	unclassified	unclassified
v261e	25246	35	14	1	Medium-quality	73.52	0	Siphoviridae	prokaryote
v261f	35616	47	10	1	Medium-quality	78.53	0	unclassified	unclassified
v2620	6209	7	3	0	Complete	100	0	unclassified	unclassified
v2621	45985	85	14	1	Medium-quality	79.79	0	unclassified	unclassified
v2622	9418	12	1	0	Medium-quality	87.8	0	unclassified	unclassified
v2623	39388	62	47	0	Complete	100	0	Podoviridae	prokaryote
v2624	45212	76	26	0	High-quality	96.43	0	unclassified	unclassified
v2625	5522	7	3	0	Complete	100	0	unclassified	unclassified
v2626	37899	62	11	2	Medium-quality	86.94	0	unclassified	unclassified
v2627	100211	176	18	1	Complete	100	0	Podoviridae_crAss-like	prokaryote
v2628	25252	42	12	0	Medium-quality	58.86	0	Siphoviridae	prokaryote
v2629	6538	10	3	0	Complete	100	0	Microviridae	prokaryote
v262a	48641	77	22	0	High-quality	98.26	0	unclassified	unclassified
v262b	30252	54	35	0	Medium-quality	64.27	0	Siphoviridae	prokaryote
v262c	112123	141	8	3	Complete	100	0	unclassified	unclassified
v262d	8410	15	9	0	High-quality	100	0	Microviridae	prokaryote
v262e	5671	10	5	0	Complete	100	0	Microviridae	prokaryote
v262f	6630	7	3	0	Complete	100	0	unclassified	unclassified
v2630	41314	59	31	0	High-quality	90.43	0	Myoviridae	prokaryote
v2631	87178	114	16	3	Complete	100	0	Quimbyviridae	prokaryote
v2632	40490	59	11	3	High-quality	95.85	0	unclassified	unclassified
v2633	61288	70	24	0	High-quality	98.24	0	Siphoviridae	prokaryote
v2634	140855	187	41	12	Complete	100	0	unclassified	unclassified
v2635	63654	78	13	0	Complete	100	0	unclassified	unclassified
v2636	33846	47	15	1	Medium-quality	89.78	0	Siphoviridae	prokaryote
v2637	26943	38	10	4	Medium-quality	65.9	0	unclassified	unclassified
v2638	50910	66	17	3	Complete	100	0	unclassified	unclassified
v2639	93574	138	28	2	High-quality	100	0	unclassified	unclassified
v263a	6896	10	4	0	High-quality	100	0	Microviridae	prokaryote
v263b	27277	48	41	0	Medium-quality	67.34	0	Podoviridae	prokaryote
v263c	29164	36	30	0	Medium-quality	72.04	0	Podoviridae	prokaryote
v263d	88319	127	29	2	Complete	100	0	unclassified	unclassified
v263e	75474	121	13	1	High-quality	93.46	0	unclassified	unclassified
v263f	55265	93	14	2	Complete	100	0	unclassified	unclassified
v2640	62483	90	15	1	Complete	100	0	Quimbyviridae	prokaryote
v2641	46729	78	15	1	Complete	100	0	unclassified	unclassified
v2642	45009	62	23	0	Complete	100	0	Siphoviridae	prokaryote
v2643	28455	45	10	0	Medium-quality	63.84	0	unclassified	unclassified

v2644	23836	50	34	0	Medium-quality	59.8	0	Podoviridae	prokaryote
v2645	42145	66	17	0	Complete	100	0	Siphoviridae	prokaryote
v2646	37788	57	31	0	Complete	100	0	Siphoviridae	prokaryote
v2647	48332	76	32	1	Complete	100	0	Myoviridae	prokaryote
v2648	91837	147	32	3	High-quality	100	0	unclassified	unclassified
v2649	5526	7	3	0	Complete	100	0	unclassified	unclassified
v264a	5176	9	5	0	Complete	100	0	Microviridae	prokaryote
v264b	45931	86	20	2	Medium-quality	57.13	0	unclassified	unclassified
v264c	41280	61	46	0	Complete	100	0	Podoviridae	prokaryote
v264d	114355	133	10	3	High-quality	100	0	unclassified	unclassified
v264e	43306	78	17	2	High-quality	100	0	unclassified	unclassified
v264f	22413	32	5	1	Medium-quality	52.97	0	Siphoviridae	prokaryote
v2650	42400	74	15	0	Complete	100	0	unclassified	unclassified
v2651	28804	45	16	0	Medium-quality	72.71	0	Siphoviridae	prokaryote
v2652	36400	62	23	0	Complete	100	0	Siphoviridae	prokaryote
v2653	40706	69	12	0	Complete	100	0	unclassified	unclassified
v2654	39751	69	49	0	High-quality	100	0	Podoviridae	prokaryote
v2655	40257	59	41	1	Complete	100	0	Podoviridae	prokaryote
v2656	29875	45	27	1	Medium-quality	74.91	0	Siphoviridae	prokaryote
v2657	36775	55	28	0	High-quality	93.72	0	Siphoviridae	prokaryote
v2658	37983	69	16	0	Complete	100	0	Siphoviridae	prokaryote
v2659	41281	76	19	1	High-quality	97.56	0	unclassified	unclassified
v265a	35592	55	21	0	Complete	100	0	unclassified	unclassified
v265b	98471	116	18	2	Complete	100	0	Gratiaviridae	prokaryote
v265c	70637	90	22	2	Medium-quality	79.16	0	unclassified	unclassified
v265d	30232	40	12	0	Medium-quality	67.1	0	unclassified	unclassified
v265e	5179	6	2	0	Medium-quality	59.6	0	Retroviridae	eukaryote
v265f	6229	7	4	0	Complete	100	0	unclassified	unclassified
v2660	42484	66	19	1	Complete	100	0	unclassified	unclassified
v2661	36004	59	17	0	Medium-quality	89.23	0	unclassified	unclassified
v2662	43395	77	20	0	Complete	100	0	unclassified	unclassified
v2663	47088	71	26	0	Complete	100	0	unclassified	unclassified
v2664	104028	104	16	1	Complete	100	0	Podoviridae_crAss-like	prokaryote
v2665	5148	8	6	0	Complete	100	0	Microviridae	prokaryote
v2666	5325	9	1	0	Medium-quality	85.87	0	Retroviridae	eukaryote
v2667	60536	86	32	0	High-quality	100	0	Siphoviridae	prokaryote
v2668	36469	61	25	0	High-quality	100	0	Siphoviridae	prokaryote
v2669	49605	91	26	0	Complete	100	0	unclassified	unclassified
v266a	22598	31	10	0	Medium-quality	51.59	0	unclassified	unclassified
v266b	22288	30	9	0	Medium-quality	56.42	0	unclassified	unclassified

v266c	10598	11	5	0	High-quality	100	0	unclassified	unclassified
v266d	9186	10	5	0	High-quality	100	0	Retroviridae	eukaryote
v266e	41642	82	20	0	Complete	100	0	unclassified	unclassified
v266f	69769	108	15	0	High-quality	100	0	Quimbyviridae	prokaryote
v2670	24480	26	23	0	Medium-quality	62.4	0	Podoviridae	prokaryote
v2671	9986	10	5	0	High-quality	100	0	unclassified	unclassified
v2672	6407	8	4	0	Complete	100	0	Microviridae	prokaryote
v2673	21636	39	7	0	High-quality	93.58	0	unclassified	unclassified
v2674	43572	57	7	0	Complete	100	0	Siphoviridae	prokaryote
v2675	37914	54	28	0	Medium-quality	62.88	0	Siphoviridae	prokaryote
v2676	37854	54	19	0	High-quality	90.77	0	Siphoviridae	prokaryote
v2677	26270	34	25	0	Medium-quality	65.93	0	Siphoviridae	prokaryote
v2678	5413	10	5	0	Complete	100	0	Microviridae	prokaryote
v2679	113810	143	22	5	Complete	100	0	Gratiaviridae	prokaryote
v267a	44096	63	17	0	Complete	100	0	unclassified	unclassified
v267b	41144	72	18	0	Complete	100	0	unclassified	unclassified
v267c	37542	53	20	1	High-quality	90.21	0	Siphoviridae	prokaryote
v267d	170166	228	21	3	High-quality	100	0	unclassified	unclassified
v267e	21468	22	19	0	Medium-quality	52.14	0	Siphoviridae	prokaryote
v267f	25741	36	11	0	Medium-quality	67.42	0	Siphoviridae	prokaryote
v2680	23474	35	30	0	Medium-quality	60.11	0	Podoviridae	prokaryote
v2681	59527	79	14	3	Medium-quality	79.66	0	unclassified	unclassified
v2682	5910	8	3	0	High-quality	97.26	0	unclassified	unclassified
v2683	59989	104	20	2	High-quality	100	0	unclassified	unclassified
v2684	34114	68	5	1	Medium-quality	64.81	0	unclassified	unclassified
v2685	26411	45	14	0	High-quality	96.81	0	unclassified	unclassified
v2686	8750	9	4	0	High-quality	100	0	unclassified	unclassified
v2687	21589	30	9	1	Medium-quality	52.94	0	Siphoviridae	prokaryote
v2688	36488	62	21	0	High-quality	100	0	unclassified	unclassified
v2689	28757	49	16	1	Medium-quality	68.29	0	Siphoviridae	prokaryote
v268a	26118	47	8	1	Medium-quality	60.43	0	unclassified	unclassified
v268b	40653	56	17	1	Complete	100	0	Siphoviridae	prokaryote
v268c	46842	75	27	2	High-quality	100	0	Myoviridae	prokaryote
v268d	70210	101	8	4	Medium-quality	84.58	0	unclassified	unclassified
v268e	5024	7	3	0	High-quality	100	0	Microviridae	prokaryote
v268f	8689	8	4	0	High-quality	100	0	unclassified	unclassified
v2690	8039	12	4	0	High-quality	100	0	unclassified	unclassified
v2691	6415	6	1	0	High-quality	100	0	unclassified	unclassified
v2692	11297	10	1	0	High-quality	100	0	unclassified	unclassified
v2693	5292	8	6	0	Complete	100	0	Microviridae	prokaryote

v2694	6016	9	4	0	Complete	100	0	unclassified	unclassified
v2695	55534	53	14	1	Medium-quality	74.48	0	unclassified	unclassified
v2696	26924	44	20	1	Medium-quality	79.68	0	Siphoviridae	prokaryote
v2697	37297	50	42	1	Complete	100	0	Podoviridae	prokaryote
v2698	6691	8	3	0	Complete	100	0	unclassified	unclassified
v2699	11063	11	1	0	High-quality	100	0	unclassified	unclassified
v269a	40679	51	36	0	Complete	100	0	Podoviridae	prokaryote
v269b	5366	9	7	0	Complete	100	0	Microviridae	prokaryote
v269c	32334	51	37	0	Medium-quality	68.45	0	Myoviridae	prokaryote
v269d	18375	22	6	0	Medium-quality	59.24	0	unclassified	unclassified
v269e	36669	49	23	0	High-quality	94.93	0	Siphoviridae	prokaryote
v269f	44713	78	11	1	Medium-quality	56.11	0	unclassified	unclassified
v26a0	174741	273	189	0	Complete	100	0	Myoviridae	prokaryote
v26a1	11374	16	6	0	High-quality	100	0	Microviridae	prokaryote
v26a2	6101	7	3	0	Complete	100	0	unclassified	unclassified
v26a3	26451	28	16	0	Medium-quality	74.67	0	unclassified	unclassified
v26a4	6096	9	3	0	Complete	100	0	unclassified	unclassified
v26a5	6982	10	4	0	High-quality	100	0	unclassified	unclassified
v26a6	37040	76	15	1	High-quality	100	0	Siphoviridae	prokaryote
v26a7	5665	8	3	0	Complete	100	0	unclassified	unclassified
v26a8	6594	9	3	0	Complete	100	0	unclassified	unclassified
v26a9	49616	93	21	0	Complete	100	0	unclassified	unclassified
v26aa	49724	71	27	0	High-quality	100	0	unclassified	unclassified
v26ab	71136	128	13	0	Medium-quality	70.5	0	Podoviridae_crAss-like	prokaryote
v26ac	38603	61	13	0	Medium-quality	84.7	0	unclassified	unclassified
v26ad	93214	158	14	3	Medium-quality	69.65	0	unclassified	unclassified
v26ae	40252	57	27	1	Complete	100	0	Siphoviridae	prokaryote
v26af	56255	92	16	1	High-quality	98.41	0	unclassified	unclassified
v26b0	34354	58	15	1	Medium-quality	83.88	0	unclassified	unclassified
v26b1	84935	94	8	4	High-quality	100	0	unclassified	unclassified
v26b2	106863	148	23	8	High-quality	100	0	unclassified	unclassified
v26b3	5416	9	5	0	Complete	100	0	Microviridae	prokaryote
v26b4	41739	71	14	0	High-quality	90.41	0	unclassified	unclassified
v26b5	10036	14	4	0	Medium-quality	78.06	0	unclassified	unclassified
v26b6	6551	8	3	0	Complete	100	0	unclassified	unclassified
v26b7	6144	8	3	0	Complete	100	0	unclassified	unclassified
v26b8	6659	10	4	0	Complete	100	0	Microviridae	prokaryote
v26b9	67952	82	15	5	High-quality	100	0	unclassified	unclassified
v26ba	43826	73	14	0	High-quality	98.98	0	unclassified	unclassified
v26bb	41652	69	18	1	Complete	100	0	Siphoviridae	prokaryote

v26bc	6133	7	4	0	Complete	100	0	unclassified	unclassified
v26bd	26702	34	3	1	Medium-quality	55.23	0	unclassified	unclassified
v26be	5021	7	7	0	Complete	100	0	Microviridae	prokaryote
v26bf	46862	56	15	0	Medium-quality	67.72	0	unclassified	unclassified
v26c0	5512	11	4	0	Complete	100	0	unclassified	unclassified
v26c1	50420	65	8	5	High-quality	100	0	unclassified	unclassified
v26c2	77864	100	26	0	High-quality	91.48	0	unclassified	unclassified
v26c3	6357	8	3	0	Complete	100	0	Microviridae	prokaryote
v26c4	34435	58	19	1	High-quality	99.87	0	Siphoviridae	prokaryote
v26c5	152718	191	17	3	High-quality	99.27	0	unclassified	unclassified
v26c6	42278	75	18	0	High-quality	100	0	unclassified	unclassified
v26c7	5638	8	6	0	Complete	100	0	Microviridae	prokaryote
v26c8	40147	67	22	0	High-quality	98.82	0	unclassified	unclassified
v26c9	32683	30	7	1	Medium-quality	55.96	0	unclassified	unclassified
v26ca	21620	41	8	0	Medium-quality	51.33	0	unclassified	unclassified
v26cb	6195	8	3	0	Complete	100	0	unclassified	unclassified
v26cc	41764	65	7	1	Medium-quality	64	0	unclassified	unclassified
v26cd	42890	57	26	0	Complete	100	0	Siphoviridae	prokaryote
v26ce	42401	69	22	0	High-quality	100	0	Siphoviridae	prokaryote
v26cf	54693	68	11	0	Medium-quality	86.06	0	unclassified	unclassified
v26d0	62582	78	18	0	High-quality	100	0	unclassified	unclassified
v26d1	70599	100	12	4	Medium-quality	80.25	0	Quimbyviridae	prokaryote
v26d2	13243	16	6	0	Complete	100	0	unclassified	unclassified
v26d3	43605	75	18	0	Complete	100	0	unclassified	unclassified
v26d4	33291	50	7	2	Medium-quality	62.32	0	unclassified	unclassified
v26d5	35388	46	13	1	High-quality	92.59	0	Siphoviridae	prokaryote
v26d6	37976	58	16	2	High-quality	99.25	0	Siphoviridae	prokaryote
v26d7	71151	109	16	5	High-quality	94.95	0	unclassified	unclassified
v26d8	45616	83	20	0	Complete	100	0	unclassified	unclassified
v26d9	92592	97	16	1	High-quality	90.83	0	Podoviridae_crAss-like	prokaryote
v26da	34949	63	18	0	Complete	100	0	unclassified	unclassified
v26db	6555	9	3	0	Complete	100	0	unclassified	unclassified
v26dc	5400	9	6	0	Complete	100	0	Microviridae	prokaryote
v26dd	14037	20	2	0	Medium-quality	50.5	0	unclassified	unclassified
v26de	12948	17	6	0	Low-quality	43.58	0	unclassified	unclassified
v26df	67068	78	11	3	Complete	100	0	unclassified	unclassified
v26e0	34338	45	25	0	Medium-quality	57.28	0	Siphoviridae	prokaryote
v26e1	13903	19	5	0	High-quality	100	0	unclassified	unclassified
v26e2	47067	49	8	1	Medium-quality	54.52	0	Quimbyviridae	prokaryote
v26e3	144809	195	10	6	Complete	100	0	unclassified	unclassified

v26e4	6136	10	2	0	Complete	100	0	unclassified	unclassified
v26e5	23655	40	14	0	Medium-quality	71.93	0	unclassified	unclassified
v26e6	40901	65	18	1	High-quality	95.93	0	unclassified	unclassified
v26e7	5240	7	5	0	High-quality	100	0	Polyomaviridae	eukaryote
v26e8	33125	67	17	1	Medium-quality	83.67	0	Siphoviridae	prokaryote
v26e9	159574	208	16	3	Complete	100	0	unclassified	unclassified
v26ea	6141	10	3	0	Complete	100	0	unclassified	unclassified
v26eb	7480	11	6	0	High-quality	100	0	Microviridae	prokaryote
v26ec	5607	8	4	0	High-quality	99.2	0	Microviridae	prokaryote
v26ed	103350	152	29	1	Complete	100	0	unclassified	unclassified
v26ee	16590	19	6	0	Medium-quality	55.84	0	unclassified	unclassified
v26ef	41250	72	16	1	Complete	100	0	unclassified	unclassified
v26f0	41784	56	13	0	Complete	100	0	unclassified	unclassified
v26f1	76454	111	22	1	Complete	100	0	unclassified	unclassified
v26f2	58735	102	20	0	Complete	100	0	unclassified	unclassified
v26f3	24756	26	3	0	Medium-quality	50.01	0	unclassified	unclassified
v26f4	56434	95	16	2	High-quality	97.97	0	unclassified	unclassified
v26f5	51348	92	20	1	Complete	100	0	unclassified	unclassified
v26f6	62042	73	12	0	Complete	100	0	unclassified	unclassified
v26f7	33452	37	9	0	Medium-quality	54.2	0	unclassified	unclassified
v26f8	40388	73	21	1	Complete	100	0	unclassified	unclassified
v26f9	97207	83	7	0	Complete	100	0	Podoviridae_crAss-like	prokaryote
v26fa	75837	96	11	3	High-quality	100	0	unclassified	unclassified
v26fb	6252	11	3	0	Complete	100	0	unclassified	unclassified
v26fc	6485	7	3	0	High-quality	100	0	unclassified	unclassified
v26fd	36653	56	10	0	Medium-quality	89.97	0	unclassified	unclassified
v26fe	30097	51	8	0	Medium-quality	82.47	0	unclassified	unclassified
v26ff	39622	49	10	1	Complete	100	0	unclassified	unclassified
v2700	98093	105	16	0	Complete	100	0	Podoviridae_crAss-like	prokaryote
v2701	5884	9	4	0	Complete	100	0	Microviridae	prokaryote
v2702	112907	148	34	4	Complete	100	0	unclassified	unclassified
v2703	5299	8	7	0	Complete	100	0	Microviridae	prokaryote
v2704	79034	105	24	0	Medium-quality	59.62	0	unclassified	unclassified
v2705	41072	64	10	0	Complete	100	0	unclassified	unclassified
v2706	33454	55	11	0	High-quality	100	0	unclassified	unclassified
v2707	6135	11	5	0	Complete	100	0	Microviridae	prokaryote
v2708	19413	26	11	0	Medium-quality	53.56	0	Siphoviridae	prokaryote
v2709	6695	9	4	0	Complete	100	0	unclassified	unclassified
v270a	31471	42	28	0	Medium-quality	78.02	0	Podoviridae	prokaryote
v270b	41703	77	19	1	High-quality	90.1	0	unclassified	unclassified

v270c	60978	76	39	1	Complete	100	0	Siphoviridae	prokaryote
v270d	40382	64	15	1	High-quality	99.02	0	Siphoviridae	prokaryote
v270e	41329	44	8	2	High-quality	99.04	0	unclassified	unclassified
v270f	49814	79	36	0	Complete	100	0	Myoviridae	prokaryote
v2710	29135	33	11	0	Medium-quality	78.71	0	unclassified	unclassified
v2711	35137	62	19	2	Complete	100	0	unclassified	unclassified
v2712	6146	9	3	0	Complete	100	0	unclassified	unclassified
v2713	46642	70	31	0	Complete	100	0	unclassified	unclassified
v2714	17438	23	8	0	Medium-quality	76.28	0	Salasmaviridae	prokaryote
v2715	59200	102	14	2	High-quality	100	0	unclassified	unclassified
v2716	55425	102	17	1	Complete	100	0	unclassified	unclassified
v2717	6121	9	2	0	Complete	100	0	unclassified	unclassified
v2718	38230	71	13	0	High-quality	90.1	0	unclassified	unclassified
v2719	71535	112	18	0	Complete	100	0	Quimbyviridae	prokaryote
v271a	40635	67	47	0	Complete	100	0	Podoviridae	prokaryote
v271b	21307	29	20	0	Medium-quality	50.11	0	Myoviridae	prokaryote
v271c	42915	82	15	0	High-quality	100	0	unclassified	unclassified
v271d	6380	8	4	0	Complete	100	0	unclassified	unclassified
v271e	19830	32	13	0	Medium-quality	55.98	0	unclassified	unclassified
v271f	5677	10	6	0	Complete	100	0	Microviridae	prokaryote
v2720	41120	55	6	0	Complete	100	0	unclassified	unclassified
v2721	6079	9	2	0	Complete	100	0	unclassified	unclassified
v2722	6833	8	3	0	High-quality	100	0	unclassified	unclassified
v2723	110889	143	9	3	Complete	100	0	unclassified	unclassified
v2724	20488	39	29	0	Medium-quality	52.35	0	Podoviridae	prokaryote
v2725	145614	194	9	8	Complete	100	0	unclassified	unclassified
v2726	40765	52	34	0	Complete	100	0	Podoviridae	prokaryote
v2727	30502	39	31	0	Medium-quality	75.46	0	Podoviridae	prokaryote
v2728	49497	67	15	0	Medium-quality	71.53	0	unclassified	unclassified
v2729	56363	63	7	3	Medium-quality	81.45	0	unclassified	unclassified
v272a	25034	32	8	1	Medium-quality	65.82	0	Siphoviridae	prokaryote
v272b	15434	17	5	0	Medium-quality	51.38	0	unclassified	unclassified
v272c	58907	79	15	0	High-quality	98.11	0	unclassified	unclassified
v272d	6469	10	4	0	High-quality	100	0	unclassified	unclassified
v272e	57865	102	16	1	Complete	100	0	unclassified	unclassified
v272f	42563	74	15	1	High-quality	96.39	0	unclassified	unclassified
v2730	24912	26	10	1	Medium-quality	64.85	0	Siphoviridae	prokaryote
v2731	5908	8	3	0	Complete	100	0	unclassified	unclassified
v2732	6952	6	3	0	High-quality	100	0	unclassified	unclassified
v2733	26170	41	18	1	Medium-quality	75.48	0	Siphoviridae	prokaryote

v2734	95351	140	22	2	Medium-quality	74.09	0	unclassified	unclassified
v2735	16274	21	7	0	Medium-quality	85.4	0	unclassified	unclassified
v2736	16203	19	7	0	Medium-quality	55.9	0	unclassified	unclassified
v2737	5734	10	6	0	Complete	100	0	Microviridae	prokaryote
v2738	6261	9	2	0	High-quality	97.39	0	unclassified	unclassified
v2739	33317	63	14	1	Medium-quality	82.02	0	unclassified	unclassified
v273a	37191	53	9	0	Complete	100	0	unclassified	unclassified
v273b	59482	103	17	1	High-quality	100	0	unclassified	unclassified
v273c	18515	33	10	0	Medium-quality	56.09	0	Siphoviridae	prokaryote
v273d	13642	19	8	0	Medium-quality	58.2	0	Salasmaviridae	prokaryote
v273e	19954	29	9	0	Medium-quality	61.71	0	unclassified	unclassified
v273f	84207	89	18	6	Medium-quality	50.15	0	unclassified	unclassified
v2740	43796	78	20	0	Complete	100	0	Siphoviridae	prokaryote
v2741	44399	79	20	2	Complete	100	0	unclassified	unclassified
v2742	6150	10	4	0	Complete	100	0	unclassified	unclassified
v2743	16691	18	7	0	Medium-quality	86.48	0	Salasmaviridae	prokaryote
v2744	48201	78	22	1	Complete	100	0	unclassified	unclassified
v2745	18851	19	9	0	High-quality	100	0	unclassified	unclassified
v2746	47280	69	40	0	High-quality	100	0	Siphoviridae	prokaryote

Supplementary Table 3| Comparison of the gut virome between IBD patients and healthy controls at the family level.

	VLP					bulk				
	Avg. abundance in IBD patients	Avg. abundance in controls	p-value	q	Enriched in	Avg. abundance in IBD patients	Avg. abundance in controls	pvalue	q	Enriched in
Siphoviridae	0.198391977	0.056673392	2.01552E-06	4.03104E-05	IBD	0.169210452	0.089397096	0.000154065	0.000616258	IBD
Myoviridae	0.031488214	0.006990531	0.000116559	0.00077706	IBD	0.092625978	0.062605173	0.110338041	0.140495988	
Retroviridae	2.69349E-05	0.000114507	0.676584481	0.676584481		0.0268287	7.26769E-06	1.23509E-07	1.23509E-06	IBD
Microviridae	0.083712561	0.178061714	5.64506E-05	0.000564506	control	0.023976921	0.007488114	0.580395269	0.580395269	
Anelloviridae	0.027816079	0.003844749	0.000253574	0.00126787	IBD	0.013169841	0.024396916	5.91149E-06	2.99539E-05	control
Inoviridae	0.001910658	0.028327271	0.00186307	0.006210234	control	0.011190485	0.003354334	0.001573766	0.003285999	IBD
Podoviridae	0.031805002	0.04021445	0.147788896	0.184736121		0.010429919	0.001789752	0.00144656	0.003285999	IBD
Salasmaviridae	0.033364746	0.001592639	0.001350509	0.005402035	IBD	0.0093186	0.00038269	0.478365228	0.503542345	
p-crAss-like	0.004372719	0.025536292	0.003237076	0.007193503	control	0.004741233	0.004870861	0.000253734	0.000732303	control
Iridoviridae	0	0	NA	NA		0.002807981	3.93878E-07	0.000256306	0.000732303	IBD
Quimbyviridae	0.012017853	0.022240197	0.013559083	0.024652878	control	0.002712981	0.008856453	5.99078E-06	2.99539E-05	control
Flandersviridae	0.004365752	0.001107626	0.161563886	0.19007516		0.001829439	0.001083047	0.004447843	0.008086987	IBD
Metaviridae	1.28965E-05	3.39674E-06	0.062107699	0.088741508		0.001018288	5.70365E-08	0.268415881	0.315783389	
Autographiviridae	0.002331066	0.017665169	0.032056706	0.053427843		0.000175826	0.000310681	0.103933214	0.140495988	
Circoviridae	0.000221123	0.00116148	0.188947027	0.209941141		4.79554E-05	0.000184326	0.001642999	0.003285999	control
Drexlerviridae	0.000669345	0.000357392	0.062119056	0.088741508		2.477E-05	4.14016E-05	0.014589573	0.024315955	control
Gratiaviridae	3.23006E-05	0.000752306	0.010216928	0.020433857	control	7.84481E-06	2.55131E-05	0.066193348	0.10183592	
Genomoviridae	0.005076133	0.001851907	0.002364273	0.006755066	IBD	5.50203E-06	9.07775E-07	0.112396791	0.140495988	
Virgaviridae	0.002324448	6.26744E-06	0.083236549	0.110982065		2.88661E-07	0	0.30401002	0.337788911	
Adenoviridae	0	0.003753606	0.002948297	0.007193503	control	0	0	NA	NA	

Supplementary Table 4| Detailed information of 139 IBD-associated vOTUs.

vOTU ID	VLP				bulk				Enriched in	Taxonomic assignment (family level)	Host assignment
	Avg. abundance in IBD patients	Avg. abundance in controls	p-value	q	Avg. abundance in IBD patients	Avg. abundance in controls	pvalue	q			
v0057	0	0.001943093	0.001748136	0.012793179	0	2.7561E-05	0.005490385	0.014279061	CON	Myoviridae	Sutterella_unknown; Sutterella_wadsworthensis
v00f5	6.84909E-07	0.000337949	0.011734865	0.049658256	0	0.000115085	0.016991487	0.039320759	CON	p-crAss-like	Prevotella_unknown
v012f	7.39253E-05	0.000304503	0.000247306	0.003412821	0.000429693	0.007033842	2.62968E-05	0.000129823	CON	unclassified	Prevotella_copri
v0154	4.10277E-06	1.78794E-05	0.007621196	0.035971051	0.000158134	0.002675408	4.56888E-05	0.000205381	CON	unclassified	Prevotella_copri
v0247	2.42714E-05	0.001336697	8.32278E-05	0.00156426	0.000159489	0.007394404	1.38251E-06	9.92446E-06	CON	unclassified	Prevotella_unknown; Prevotella_copri; Prevotella_sp.
v02ca	0	0.005073371	0.000355518	0.004402958	2.49518E-08	0.000355819	0.000380512	0.001307429	CON	unclassified	unclassified
v02dc	3.06171E-07	7.02319E-05	0.000245628	0.003412821	4.81565E-05	0.006545307	2.64707E-09	5.47709E-08	CON	unclassified	Prevotella_unknown; Prevotella_copri
v02e0	0	0.000241618	0.001748136	0.012793179	0	0.000310846	0.005490385	0.014279061	CON	Quimbyviridae	CAG-611_unknown
v02e6	4.40913E-05	6.85546E-05	0.001918754	0.013772015	0.000520692	0.005474699	0.001317061	0.004011049	CON	unclassified	Prevotella_sp.; Prevotella_unknown
v0308	4.75196E-09	7.6313E-06	0.002458267	0.016881652	0.000135279	0.000452755	1.1148E-08	1.70491E-07	CON	unclassified	CAG-103_sp.
v0312	1.21714E-06	3.67155E-05	0.011754853	0.049658256	6.36543E-05	0.000536382	7.824E-09	1.2951E-07	CON	unclassified	Ruminococcus_sp.; CAG-110_unknown; UBA1417_sp.
v04f1	0.000653852	6.9669E-06	4.36656E-06	0.000162235	0.000253739	6.92342E-05	0.000129034	0.000502989	IBD	unclassified	Dialister_invisus
v04f2	0.002367263	5.68489E-07	2.51629E-05	0.000628638	1.60295E-05	0	0.005000626	0.013175806	IBD	unclassified	unclassified
v04f6	0.000963798	0	0.001492854	0.011885414	1.98084E-05	0	0.001345361	0.004070803	IBD	Siphoviridae	unclassified
v04fd	0.009080298	7.39177E-07	3.21687E-12	4.66125E-09	0.000212575	0	0.005000626	0.013175806	IBD	Microviridae	unclassified
v0503	0.001012229	3.84707E-05	7.09438E-06	0.000206566	0.001030579	0.000483795	0.019292067	0.043085616	IBD	unclassified	Salmonella_enterica
v054f	0.000767723	3.02345E-07	0.000144604	0.002332074	3.05931E-05	0	0.01855263	0.041566163	IBD	Siphoviridae	unclassified
v05af	0.012734495	1.15919E-08	6.53604E-10	3.15691E-07	3.22886E-05	0	0.009623394	0.023589051	IBD	unclassified	unclassified
v05c9	0.00710777	0	5.18822E-09	1.25296E-06	2.12013E-05	0	0.005000626	0.013175806	IBD	Microviridae	Parasutterella_sp.
v0606	0.005828558	4.00943E-07	2.05981E-06	9.32706E-05	1.48317E-05	0	0.01855263	0.041566163	IBD	Microviridae	Faecalibacterium_sp.
v0686	0.000635667	6.46536E-07	0.00962519	0.043179258	4.86553E-05	0	0.01855263	0.041566163	IBD	Myoviridae	Dakarella_massiliensis; Dakarella_unknown
v06fa	0.000453643	3.33954E-07	0.003319473	0.02046773	0.000355613	0	0.000694547	0.002251675	IBD	Siphoviridae	unclassified
v06ff	0.00873219	6.19796E-06	0.000144849	0.002332074	0.001784485	5.79794E-07	0.004699672	0.012476299	IBD	Siphoviridae	unclassified
v072c	0.002547298	9.43363E-07	0.004708592	0.026044183	6.88274E-06	2.45513E-09	0.010543182	0.025664804	IBD	unclassified	Clostridium_citroniae
v0735	0.002953457	5.1385E-06	2.33415E-05	0.000593365	4.87691E-05	0	0.009623394	0.023589051	IBD	unclassified	unclassified
v0749	0.000472071	5.53852E-07	0.007782823	0.03626145	6.46702E-06	0	0.01855263	0.041566163	IBD	unclassified	Veillonella_unknown;
v086b	0	0.005444375	0.000607348	0.006470932	0	0.000296702	0.016991487	0.039320759	CON	unclassified	CAG-170_sp.
v09dc	0.000435464	4.59595E-07	0.00040106	0.004827933	5.17042E-05	0	0.002596791	0.007396124	IBD	unclassified	unclassified
v0b4d	0.000976397	0	0.000346327	0.004402958	7.8199E-05	0	0.01855263	0.041566163	IBD	unclassified	unclassified
v0b75	0.012220808	0	7.57911E-07	4.06745E-05	0.000574487	0	0.01855263	0.041566163	IBD	unclassified	unclassified
v0bb8	0.00064668	3.7222E-06	0.001104025	0.009522216	0.000116409	2.83173E-08	0.010827426	0.026220634	IBD	unclassified	unclassified
v0bd8	3.69982E-07	1.45666E-05	0.007496142	0.035496435	3.91794E-07	0.002258571	6.28645E-05	0.000272995	CON	unclassified	Prevotella_sp.; Prevotella_unknown
v0bdb	1.8867E-07	9.28375E-05	5.69421E-05	0.001178701	3.39015E-06	0.00120222	1.75825E-07	1.84617E-06	CON	unclassified	Prevotella_unknown; Prevotella_copri; Prevotella_sp.
v0be4	0	2.94072E-05	2.19863E-06	9.654E-05	0	0.000463137	3.53478E-07	3.14775E-06	CON	unclassified	Prevotella_unknown; Prevotella_oralis
v0c00	1.38755E-07	1.40577E-05	0.001079654	0.009481326	4.45341E-07	0.001405847	1.20032E-06	8.93572E-06	CON	unclassified	Prevotella_sp.; Prevotella_unknown
v0c0f	0.000406079	7.26803E-06	0.004102471	0.023873417	0.00300761	0.001245929	0.001425105	0.00428445	IBD	Myoviridae	Escherichia_coli; Escherichia_sp.; Citrobacter_farmeri; Cronobacter_sakazakii
v0c18	0	0.00052823	0.000607348	0.006470932	0	0.000211727	5.32207E-05	0.000234005	CON	Quimbyviridae	Prevotella_sp.
v0c24	1.91159E-07	0.000215002	0.008297132	0.038010627	0.000126189	0.000458739	2.26111E-09	4.97091E-08	CON	unclassified	unclassified
v0c59	8.06007E-05	0.000623139	0.000900082	0.008350704	0.001035212	0.00130922	7.05168E-06	4.20411E-05	CON	Myoviridae	Lawsonibacter_sp.; Clostridium_jeddahense; CAG-110_unknown; Acutalibacteraceae_unknown; Peptococcaceae_unknown

v0c6d	1.32773E-06	1.20087E-05	0.000904804	0.008350704	5.04613E-05	0.000684189	5.54534E-06	3.37762E-05	CON	Siphoviridae	Ruminococcus_bromii; Ruminococcus_unknown; CAG-964_sp.; Ruminococcus_sp.
v0c76	4.52446E-06	0.000169667	0.001100653	0.009522216	0.000107523	0.000236946	2.00177E-07	2.03701E-06	CON	Myoviridae	Agathobaculum_butyriciproducens; Lawsonibacter_sp.; Clostridium_jeddahense; CAG-110_unknown; Acutalibacteraceae_unknown; Peptococcaceae_unknown
v0ca6	0.000395109	6.37686E-06	0.000930773	0.008536011	0.003225272	0.001569194	0.011273724	0.027254519	IBD	Myoviridae	Escherichia_coli; Escherichia_sp.; Escherichia_albertii; Escherichia_marmotae; Citrobacter_koseri; Kluyvera_ascorbata; Citrobacter_portucalensis; Enterobacter_timonensis; Pantoea_sp.; RIT-PI-d_unknown
v0cf8	2.626E-07	2.01948E-07	0.010784581	0.047354117	0.003692938	0.00043679	0.00689829	0.017583142	IBD	unclassified	Bifidobacterium_infantis
v0d1b	0.000118492	4.55181E-07	0.006770526	0.033369105	0.002659108	0.000663292	0.003674429	0.010038684	IBD	Myoviridae	Escherichia_coli
v0d26	3.30203E-07	2.3808E-06	0.004100528	0.023873417	0.000334248	0.000580343	3.15157E-07	2.89821E-06	CON	unclassified	unclassified
v0d28	1.35551E-06	7.96257E-10	0.001455485	0.011782112	0.000480125	1.33425E-05	1.4033E-08	2.05672E-07	IBD	Siphoviridae	Streptococcus_parasanguinis
v0d50	0.000360048	4.99354E-06	0.004753012	0.026087556	0.002428113	0.000941329	0.005096263	0.013377691	IBD	Siphoviridae	Escherichia_coli; Escherichia_fergusonii
v0d5d	4.6668E-05	3.20717E-08	0.011734865	0.049658256	0.000408601	2.94422E-06	1.4973E-10	6.19616E-09	IBD	Siphoviridae	Streptococcus_parasanguinis; Streptococcus_unknown
v0d62	0.001224909	0	0.000720785	0.007202875	0.008921394	2.16898E-06	8.93017E-08	1.03841E-06	IBD	Siphoviridae	Lactobacillus_salivarius
v0d6e	0.018249367	4.55894E-06	1.30513E-10	9.45563E-08	0.003057134	7.23581E-06	1.99101E-05	0.000105275	IBD	Siphoviridae	Enterococcus_faecium
v0da4	9.40235E-07	4.63265E-05	0.007496142	0.035496435	0	0.000467268	9.66662E-05	0.000390714	CON	unclassified	unclassified
v0df5	0.0002933	0	0.00634656	0.032042387	0.00074368	1.22934E-06	0.019839432	0.04416785	IBD	Siphoviridae	Klebsiella_grimontii; Klebsiella_pneumoniae
v0e93	7.21468E-07	0	0.00634656	0.032042387	0.001814509	2.09206E-05	0.008873644	0.022097728	IBD	Myoviridae	Klebsiella_pneumoniae
v0ea0	2.53372E-05	3.43509E-05	0.002732135	0.018243608	0.000462578	0.004551932	6.15642E-10	1.8046E-08	CON	unclassified	Bacteroides_thetaiotaomicron; Bacteroides_unknown; Prevotella_unknown; Butyricimonas_synergistica
v0edf	3.49203E-06	0.000396092	1.74529E-08	3.61274E-06	0.000174807	0.00883737	4.12146E-09	7.77687E-08	CON	unclassified	Prevotella_sp.; Prevotella_unknown; Prevotella_copri
v0ee2	9.38095E-09	0.000112777	4.21522E-05	0.000935861	1.71847E-05	0.002757768	6.1241E-08	7.36462E-07	CON	unclassified	UBA11471_sp.; Prevotella_copri; Prevotella_unknown
v0ee4	3.86254E-07	1.07514E-06	0.006784854	0.033369105	0.000147793	0.000633035	1.45937E-09	3.54023E-08	CON	unclassified	Fournierella_massiliensis; Agathobaculum_butyriciproducens; Faecalibacterium_prausnitzii; ER4_sp.; Lawsonibacter_sp.; Hungatella_unknown; Clostridium_saccharolyticum; Oscillibacter_sp.; Pseudoflavonifractor_capillosus; Lawsonibacter_unknown; Gemmiger_variabile; Absiella_innocuum; Intestinimonas_massiliensis;
v0ee5	9.88073E-07	9.11652E-06	0.001423005	0.0115839	4.12999E-05	0.000573456	1.60267E-05	8.57398E-05	CON	unclassified	Ruminococcus_bromii
v0efd	1.1442E-08	2.3676E-05	0.00026631	0.003606387	1.15153E-05	0.001692528	1.39124E-07	1.48294E-06	CON	unclassified	Prevotella_unknown; Prevotella_copri
v0f0a	0.001085372	0	0.00634656	0.032042387	0.000327367	4.15146E-06	0.000417805	0.00141651	IBD	unclassified	unclassified
v0f15	7.89085E-08	3.61243E-06	0.007887534	0.036631526	3.13261E-05	0.00112252	5.04037E-06	3.16598E-05	CON	unclassified	Prevotella_sp.
v0f3a	3.99855E-10	3.03593E-05	0.000428435	0.005088538	3.36174E-06	0.000820294	2.74501E-07	2.62737E-06	CON	unclassified	Prevotella_unknown; Prevotella_copri

v0f4a	1.38889E-06	2.3318E-06	0.001213215	0.010161549	0.000265653	0.00109583	4.34348E-09	7.93673E-08	CON	unclassified	Fournierella_massiliensis; Agathobaculum_butyriciproducens; Faecalibacterium_prausnitzii; ER4_sp.; Lawsonibacter_sp.; Hungatella_unknown; Clostridium_saccharolyticum; Oscillibacter_sp.; Pseudoflavonifractor_capillosus; Lawsonibacter_unknown; Gemmiger_variabile; Absiella_innocuum; Intestinimonas_massiliensis;
v0f83	6.96666E-07	1.7818E-06	0.000569207	0.006393646	0.00020437	0.001086297	4.1634E-10	1.3266E-08	CON	unclassified	Fournierella_massiliensis; Agathobaculum_butyriciproducens; Faecalibacterium_prausnitzii; ER4_sp.; Lawsonibacter_sp.; Hungatella_unknown; Clostridium_saccharolyticum; Oscillibacter_sp.; Pseudoflavonifractor_capillosus; Lawsonibacter_unknown; Gemmiger_variabile; Absiella_innocuum; Intestinimonas_massiliensis;
v0f95	0	1.80176E-06	0.002948297	0.01890302	5.0192E-05	0.000431966	9.75842E-10	2.69218E-08	CON	unclassified	UBA1777_unknown
v0fa6	9.23106E-07	1.05561E-05	6.73177E-06	0.000206566	0.000430288	0.002340701	3.54563E-10	1.21676E-08	CON	unclassified	Faecalicatena_torques; Faecalicatena_gnavus; Emergencia_timonensis; Roseburia_unknown; Marseille-P4683_sp.; UBA9502_unknown; Faecalicatena_fissicatena; Clostridium_bolteae; Clostridium_saccharolyticum; UBA7096_unknown; Anaerotignum_sp.; Dorea_scindens; Eisenbergiella_unknown; Eisenbergiella_massiliensis; Agathobaculum_sp.; Lachnospiraceae_unknown; Dorea_sp.; Clostridium_sp.; GCA-900066755_unknown; Eisenbergiella_tayi; Sellimonas_intestinalis; Eubacterium_limosum; CAG-353_sp.;
v0ffb	4.74566E-05	6.28184E-05	0.003961857	0.023431552	0.000752057	0.007476577	0.000138137	0.000526716	CON	unclassified	Prevotella_copri
v1004	7.13273E-06	1.17173E-05	0.006462114	0.032288284	0.000187913	0.001794345	5.10803E-06	3.18009E-05	CON	unclassified	Prevotella_unknown; Prevotella_copri
v100b	2.60713E-06	4.20411E-06	0.006561874	0.032674073	9.25668E-05	0.000751774	7.74816E-05	0.000326397	CON	unclassified	unclassified
v100d	2.43199E-07	1.73622E-06	0.001919908	0.013772015	0.000313677	0.000696118	2.60682E-06	1.75492E-05	CON	unclassified	Catenibacterium_mitsuokai; GCA-900066995_sp.; UBA7160_unknown; Eubacterium_sp.; Dorea_sp.; TF01-11_sp.; Coprobacillus_cateniformis; Christensenella_minuta; Acetatifactor_unknown; Ruminococcus_bromii; CAG-110_unknown; Bacteroides_pectinophilus

v102d	1.94194E-07	7.25431E-06	6.35774E-06	0.000206566	7.99071E-06	0.001791994	2.97878E-10	1.16421E-08	CON	unclassified	CAG-611_unknown; Parabacteroides_sp.; Prevotella_unknown; Prevotella_oris
v1059	0	9.8345E-06	0.000607348	0.006470932	0	0.000523257	6.78559E-07	5.45561E-06	CON	unclassified	Prevotellamassilia_unknown; Prevotella_unknown
v108a	7.06029E-05	0.00031525	0.00290852	0.01890302	0.000585518	0.004407962	7.46063E-05	0.000316178	CON	unclassified	Prevotella_sp.; UBA11471_sp.; Prevotella_copri
v108e	8.85484E-06	2.10185E-05	0.011185081	0.048379649	0.000917443	0.002191919	3.79766E-09	7.3196E-08	CON	unclassified	KLE1615_sp.; Dorea_longicatena; Acetatifactor_sp.; Coprococcus_sp.; Blautia_unknown; Agathobaculum_butyriciproducens; Agathobacter_faecis; Roseburia_intestinalis; Roseburia_hominis; Hungatella_effluvii; Lawsonibacter_sp.; Blautia_sp.; Agathobacter_sp.; Lachnospiraceae_unknown; Faecalicatena_sp.; UBA9502_unknown; RUG563_unknown; Absiella_sp.; Faecalicatena_fissicatena;
v1090	0.000914957	1.56968E-05	3.63272E-05	0.000850745	0.010029736	0.003323562	0.002175489	0.006324201	IBD	unclassified	Escherichia_coli; Escherichia_fergusonii; Escherichia_sp.; Escherichia_albertii; Escherichia_marmotae; Citrobacter_koseri; Citrobacter_portucalensis
v10ae	7.29008E-08	1.13157E-06	0.004534579	0.025566554	0	0.000573083	1.82703E-07	1.89017E-06	CON	unclassified	Prevotella_unknown; Prevotella_sp.; Prevotella_jejuni
v10af	9.92724E-09	4.23469E-06	0.00026631	0.003606387	6.21478E-06	0.001014528	2.29266E-08	3.13181E-07	CON	unclassified	unclassified
v10b0	8.90785E-10	1.18985E-05	0.002210771	0.015550519	3.85834E-06	0.000576115	2.66628E-07	2.57033E-06	CON	unclassified	Prevotella_unknown; Prevotella_copri
v10cf	4.32758E-05	9.40147E-05	0.000671164	0.006946542	0.000793438	0.006306011	0.000109172	0.000436379	CON	unclassified	Prevotella_copri
v10ff	1.06645E-07	1.09723E-05	2.84422E-06	0.000121214	2.85408E-05	0.006321679	1.24601E-12	1.08234E-10	CON	unclassified	Prevotella_unknown; Prevotellamassilia_timonensis; Prevotella_sp.
v1114	0	4.93722E-06	0.001748136	0.012793179	3.47445E-05	0.001076647	8.24227E-07	6.37191E-06	CON	unclassified	Prevotella_sp.; Prevotella_unknown
v1115	1.3947E-06	1.25929E-05	0.000567756	0.006393646	6.49696E-05	0.000863628	2.34889E-05	0.000119014	CON	Siphoviridae	Ruminococcus_bromii; Coprobacter_unknown
v1122	5.38215E-05	7.73092E-05	0.001525933	0.012016721	0.000934518	0.007326108	0.000501811	0.001669146	CON	unclassified	Prevotella_copri
v1151	3.60537E-07	0.000168803	3.13825E-06	0.000126314	2.64302E-06	0.001393384	4.03952E-08	5.12036E-07	CON	unclassified	Prevotella_copri; Prevotella_unknown
v119c	1.96174E-06	1.38954E-05	0.001161338	0.009840807	7.71655E-05	0.000971604	2.74011E-05	0.000134332	CON	unclassified	Ruminococcus_bromii
v11af	4.64262E-07	1.02356E-06	0.004046345	0.023737465	0.000241242	0.001017327	4.24286E-10	1.3266E-08	CON	unclassified	Fournierella_massiliensis; Agathobaculum_butyriciproducens; Faecalibacterium_prausnitzii; ER4_sp.; ER4_unknown; Lawsonibacter_sp.; Hungatella_unknown; Clostridium_saccharolyticum; Anaerovoracaceae_unknown; Oscillibacter_sp.; Pseudoflavonifractor_capillosus; Lawsonibacter_unknown; Gemmiger_variabile; Absiella_innocuum; Intestinimonas_massiliensis;
v11b0	2.10758E-05	3.99905E-05	0.002709886	0.018178817	0.000549906	0.004414518	0.000256715	0.000909818	CON	unclassified	Prevotella_unknown

v11d2	7.58889E-09	1.33072E-06	0.007337073	0.035320329	7.27671E-06	0.000668678	5.15544E-12	3.02238E-10	CON	unclassified	Prevotella_sp.; Prevotella_stercorea; Prevotellamassilia_timonensis
v11d3	2.08501E-05	2.1973E-05	0.004167975	0.024157586	0.00029867	0.002124912	7.09104E-05	0.000302336	CON	unclassified	unclassified
v1209	4.37129E-06	9.59296E-05	9.64041E-07	4.98891E-05	0.000156389	0.006003778	9.32555E-08	1.0755E-06	CON	unclassified	UBA11471_sp.; Prevotella_copri; Prevotella_lascolaii; Prevotella_unknown
v122a	1.97094E-07	9.5869E-06	0.000846198	0.008120137	7.06005E-05	0.000699683	1.59448E-12	1.24635E-10	CON	Siphoviridae	Faecalicatena_gnavus
v1247	4.84263E-07	0.001200183	3.93388E-05	0.000890655	1.17149E-06	0.000852272	1.55493E-06	1.10494E-05	CON	unclassified	Prevotella_copri
v1248	1.76312E-05	0.000869823	8.63636E-05	0.00156426	8.01528E-05	0.000151764	1.40832E-06	1.00584E-05	CON	Myoviridae	Faecalibacterium_prausnitzii; Lawsonibacter_sp.; Clostridium_jeddahense; CAG-110_unknown; Acutalibacteraceae_unknown; Peptococcaceae_unknown
v1260	3.49712E-07	9.58873E-06	2.99823E-06	0.000124127	1.22863E-05	0.002589643	7.11863E-11	3.12997E-09	CON	unclassified	Prevotella_sp.; Prevotella_unknown
v1356	1.68489E-06	0.001881218	1.26812E-07	1.20731E-05	1.57536E-05	0.00283079	3.87038E-10	1.26642E-08	CON	unclassified	CAG-279_sp.; Prevotellamassilia_unknown; Prevotella_unknown;
v151e	1.64042E-07	1.86611E-06	0.00474821	0.026087556	6.23826E-05	0.001177299	4.38993E-08	5.46605E-07	CON	unclassified	Prevotella_sp.
v1738	3.80571E-06	4.1663E-06	1.57044E-05	0.00041374	9.67451E-05	0.000862405	1.95087E-12	1.33542E-10	CON	unclassified	unclassified
v1794	1.00281E-05	2.13935E-05	0.000106905	0.001825233	0.000157525	0.001484639	6.08343E-12	3.42375E-10	CON	unclassified	Bacteroides_coprophilus; Bacteroides_coprocola; Bacteroides_plebeius; Prevotella_sp.; Bacteroides_unknown; Paraprevotella_clara; Prevotella_copri; Parabacteroides_unknown;
v1994	0	2.89576E-06	0.004957032	0.026310399	5.33197E-07	0.000492611	1.33894E-06	9.71076E-06	CON	unclassified	Prevotella_unknown
v19ae	2.0036E-05	9.41098E-05	0.007471084	0.035496435	0.000310515	0.000692313	1.00881E-09	2.69605E-08	CON	unclassified	Faecalibacterium_prausnitzii
v19f6	3.38215E-06	1.29739E-05	0.006928818	0.03386691	0.000223484	0.000500749	9.82982E-08	1.11537E-06	CON	unclassified	CAG-103_sp.
v1a30	8.11203E-08	3.03159E-06	0.002660252	0.017994326	3.16201E-06	0.000790913	3.53533E-10	1.21676E-08	CON	unclassified	Prevotellamassilia_timonensis; Prevotellamassilia_unknown; Prevotella_colorans
v1a34	5.13376E-08	3.31834E-05	4.55201E-06	0.000164896	1.75666E-06	0.0008701	4.45448E-08	5.49777E-07	CON	unclassified	Prevotella_unknown
v1a54	1.4471E-05	2.94153E-05	0.000703556	0.007179241	8.55737E-05	0.000699187	5.14825E-06	3.19101E-05	CON	unclassified	Prevotella_unknown; Prevotella_copri
v1a9e	4.76461E-09	2.14114E-05	4.66229E-05	0.001008308	6.04805E-06	0.001031518	2.11666E-07	2.12724E-06	CON	unclassified	Prevotella_unknown; Prevotella_copri
v1ab1	1.28256E-05	1.76338E-05	0.008459111	0.038423988	0.000313813	0.000561041	5.82942E-13	6.835E-11	CON	unclassified	Faecalibacterium_unknown; Faecalibacterium_prausnitzii; Faecalibacterium_sp.; Eubacterium_sp.; Absiella_unknown; KLE1615_sp.; Emergencia_timonensis; UBA7182_unknown; Holdemanella_sp.; Lachnospiraceae_unknown;
v1ad3	4.26609E-07	1.13515E-06	0.000829347	0.008020599	0.00010774	0.000521164	4.45866E-11	2.16322E-09	CON	unclassified	CAG-103_sp.
v1b3a	0.00051213	6.0361E-06	0.003208138	0.019922726	0.003112726	0.001074383	0.005330589	0.013914914	IBD	unclassified	Escherichia_coli; Escherichia_fergusonii; Escherichia_albertii; Escherichia_marmotae; Citrobacter_koseri
v1b6b	9.64776E-10	0.000271303	0.010873675	0.047535764	0	0.000210348	0.000560242	0.001837438	CON	unclassified	unclassified
v1b78	1.06264E-07	7.01736E-05	0.000379237	0.004617768	2.32422E-05	0.001464081	1.57083E-06	1.10508E-05	CON	unclassified	Prevotellamassilia_unknown; Prevotella_copri; Prevotella_unknown
v1ba8	2.5329E-08	1.48096E-05	0.00016016	0.00239721	1.72639E-05	0.002269928	3.69466E-08	4.76917E-07	CON	unclassified	Prevotella_copri
v1bab	3.33735E-08	1.60036E-05	0.000518507	0.006010533	5.65592E-07	0.000924778	1.26895E-05	7.1132E-05	CON	unclassified	Prevotella_unknown
v1bac	4.96027E-05	0.001006477	0.004709162	0.026044183	0.000275219	0.000641374	0.000917047	0.002873686	CON	unclassified	Bacteroides_plebeius
v1bf8	6.7615E-07	6.58008E-06	0.00010707	0.001825233	2.34467E-05	0.000347916	8.75835E-05	0.000364726	CON	unclassified	unclassified
v1c08	2.8971E-06	5.63605E-06	0.001960838	0.013878759	0.000491932	0.000675785	1.42106E-11	7.40532E-10	CON	unclassified	Gemmiger_unknown
v1c37	3.21206E-06	2.34528E-05	0.000348296	0.004402958	8.76546E-05	0.001030022	4.6477E-06	2.93243E-05	CON	unclassified	Ruminococcus_bromii

v1c6b	0.00060136	2.09841E-07	0.002669966	0.017994326	3.70852E-05	0	0.01855263	0.041566163	IBD	unclassified	unclassified
v1cd0	0.000593719	0	0.00634656	0.032042387	1.12895E-05	0	0.001345361	0.004070803	IBD	Siphoviridae	unclassified
v1cda	4.40834E-06	1.02989E-05	0.003217335	0.019922726	0.000257818	0.001285886	1.18231E-13	1.84834E-11	CON	unclassified	ER4_sp.
v1cdb	2.65926E-08	4.38355E-05	8.50677E-05	0.00156426	1.02095E-05	0.001805559	2.00014E-07	2.03701E-06	CON	unclassified	Prevotella_unknown; Prevotella_copri
v1cdc	1.1787E-05	1.40913E-05	4.24978E-06	0.000162051	9.91477E-06	0.000823402	1.01632E-08	1.6067E-07	CON	unclassified	Prevotella_unknown; Prevotella_sp.
v1cf7	0.000740191	1.55714E-05	0.001961417	0.013878759	0.006078573	0.002451803	0.003997917	0.010734866	IBD	unclassified	Escherichia_coli; Escherichia_fergusonii; Escherichia_sp.; Escherichia_albertii; Escherichia_marmotae
v1cf9	0	5.06261E-06	0.001748136	0.012793179	1.27572E-05	0.000670615	1.5661E-06	1.10508E-05	CON	unclassified	Ruminococcus_bromii; Ruminococcus_unknown
v1d2c	7.21428E-09	9.13E-07	0.001511353	0.011966943	0.000112183	0.000690845	2.11895E-13	2.98137E-11	CON	unclassified	Flavonifractor_plautii; ER4_sp.; Lawsonibacter_sp.; Lawsonibacter_unknown
v1d4e	6.57994E-07	1.39073E-05	0.000453272	0.005298215	5.31573E-06	0.000864162	7.78124E-07	6.15067E-06	CON	unclassified	Prevotella_sp.
v1d53	2.01131E-07	2.32019E-06	0.000558587	0.006373167	0.000249801	0.001462422	7.62041E-05	0.000321979	CON	unclassified	Eubacterium_sp.; Absiella_unknown; KLE1615_sp.; Emergencia_timonensis; UBA7182_unknown; Holdemanella_sp.; Lachnospiraceae_unknown;
v1d54	4.35356E-07	3.45212E-06	0.003582124	0.021627075	0.000218538	0.000844838	0.000245679	0.00087957	CON	Siphoviridae	Eubacterium_sp.; Absiella_unknown; KLE1615_sp.; Emergencia_timonensis; UBA7182_unknown; Holdemanella_sp.;
v1d57	2.05625E-06	1.34382E-05	0.000183541	0.002628175	0.000292015	0.000907589	2.24929E-07	2.2287E-06	CON	unclassified	CAG-41_sp.; Clostridium_clostridioforme; Eubacterium_sp.; Intestinimonas_massiliensis; Eisenbergiella_unknown; UBA737_unknown; Monoglobus_pectinilyticus
v1d6c	7.71587E-07	9.65823E-06	0.000453401	0.005298215	3.43859E-05	0.000518809	2.10674E-05	0.000108578	CON	unclassified	Ruminococcus_bromii
v1d95	9.32643E-06	1.02184E-05	0.000116658	0.001941312	0.000519523	0.002063565	1.81698E-09	4.26082E-08	CON	unclassified	unclassified
v1e19	1.16114E-07	2.78071E-06	5.09672E-05	0.001070311	1.08426E-05	0.000582331	6.16695E-11	2.8923E-09	CON	unclassified	Prevotella_sp.
v1e30	3.81108E-08	2.47678E-05	6.47422E-06	0.000206566	1.45174E-05	0.001361468	4.14546E-09	7.77687E-08	CON	unclassified	Prevotella_unknown
v1e3a	0	9.928E-07	0.001032625	0.009179596	3.05121E-06	0.000557834	2.54308E-09	5.43195E-08	CON	unclassified	Prevotella_sp.; UBA11471_sp.; Prevotellamassilia_timonensis; Prevotella_unknown
v1eb3	1.73433E-06	7.26914E-06	0.006441803	0.032288284	0.000202066	0.003306197	2.18676E-06	1.50086E-05	CON	unclassified	Prevotella_sp.; Prevotella_unknown
v1f42	3.88487E-07	5.59241E-06	4.26272E-05	0.000935861	2.97838E-05	0.00096569	1.91153E-09	4.40906E-08	CON	unclassified	Prevotella_sp.; Prevotella_unknown
v1fc2	0.000436149	2.98781E-07	0.005427758	0.028495731	0.000341836	0	0.009623394	0.023589051	IBD	Siphoviridae	Eubacterium_unknown
v2055	1.70661E-07	1.98162E-05	6.15452E-05	0.001256042	1.28346E-06	0.000438485	7.28052E-07	5.7874E-06	CON	unclassified	Prevotella_copri
v24d2	1.83605E-05	0	0.000165439	0.00239721	0.000460782	7.28692E-06	0.005225963	0.013685212	IBD	unclassified	Parabacteroides_unknown; Bacteroides_mediterraneensis; Bacteroides_fragilis
v26b8	0.003843149	1.09524E-07	1.20392E-06	6.01543E-05	2.1129E-05	0	0.005000626	0.013175806	IBD	Microviridae	unclassified

Supplementary Table 5| External validation cohorts for this study.

Project	Country	No. of IBD patients	No. of healthy controls
**Bulk metagenomes**			
Federic et al. (2020)	France (FRA)	42 (CD), 44 (UC)	25
	Germany (GER)	68 (UC)	123
	Israel (ISR)	54 (CD), 32 (UC)	30
	United States (USA)	24 (CD), 44 (UC)	51
Franzosa et al. (2019)	Netherlands (NED)	20 (CD), 23 (UC)	22
	United States (USA)	68 (CD), 53 (UC)	34
He et al. (2017)	China (CHN)	49 (CD)	53
Weng et al. (2019)	China (CHN)	41 (CD), 25 (UC)	15
**VLP metagenomes**			
Stockdale et al. (2023)	Israel (ISR)	55 (CD), 59 (UC)	117
**Total**		701	470
